# Multi-omics analysis reveals NNMT as a master metabolic regulator of metastasis in esophageal squamous cell carcinoma

**DOI:** 10.1038/s41698-024-00509-w

**Published:** 2024-01-30

**Authors:** Qi Huang, Haiming Chen, Dandan Yin, Jie Wang, Shaodong Wang, Feng Yang, Jiawei Li, Teng Mu, Jilun Li, Jia Zhao, Rong Yin, Wei Li, Mantang Qiu, Erbao Zhang, Xiangnan Li

**Affiliations:** 1 Department of Thoracic Surgery, The First Affiliated Hospital of Zhengzhou University, Zhengzhou, 450003 China; 2 Department of Thoracic Surgery, Peking University People’s Hospital, Beijing, 100044 China; 3 Thoracic Oncology Institute, Peking University People’s Hospital, Beijing, 100044 China; 4 Clinical Research Center, The Second Hospital of Nanjing, Nanjing University of Chinese Medicine, Zhong Fu Road, Gulou District, Nanjing, 210003 China; 5 Department of Thoracic Surgery, Jiangsu Key Laboratory of Molecular and Translational Cancer Research, Jiangsu Cancer Hospital and Nanjing Medical University Affiliated Cancer Hospital and Jiangsu Institute of Cancer Research, Nanjing, 21009 China; 6 Department of Science and Technology, Jiangsu Cancer Hospital and Nanjing Medical University Affiliated Cancer Hospital and Jiangsu Institute of Cancer Research, Nanjing, 21009 China; 7 Biobank of Lung Cancer, Jiangsu Biobank of Clinical Resources, Nanjing, 21009 China; 8 Department of Oncology, First Affiliated Hospital of Nanjing Medical University, Nanjing, Jiangsu PR China; 9 Department of Epidemiology, Center for Global Health, School of Public Health, Jiangsu Key Lab of Cancer Biomarkers, Prevention and Treatment, Collaborative Innovation Center for Cancer Personalized Medicine, Nanjing Medical University, Nanjing, 211166 China

**Keywords:** Cancer metabolism, Cancer microenvironment, Cancer epigenetics

## Abstract

Metabolic reprogramming has been observed in cancer metastasis, whereas metabolic changes required for malignant cells during lymph node metastasis of esophageal squamous cell carcinoma (ESCC) are still poorly understood. Here, we performed single-cell RNA sequencing (scRNA-seq) of paired ESCC tumor tissues and lymph nodes to uncover the reprogramming of tumor microenvironment (TME) and metabolic pathways. By integrating analyses of scRNA-seq data with metabolomics of ESCC tumor tissues and plasma samples, we found nicotinate and nicotinamide metabolism pathway was dysregulated in ESCC patients with lymph node metastasis (LN^+^), exhibiting as significantly increased 1-methylnicotinamide (MNA) in both tumors and plasma. Further data indicated high expression of N-methyltransferase (NNMT), which converts active methyl groups from the universal methyl donor, S-adenosylmethionine (SAM), to stable MNA, contributed to the increased MNA in LN^+^ ESCC. NNMT promotes epithelial–mesenchymal transition (EMT) and metastasis of ESCC in vitro and in vivo by inhibiting E-cadherin expression. Mechanically, high NNMT expression consumed too much active methyl group and decreased H3K4me3 modification at E-cadherin promoter and inhibited m6A modification of E-cadherin mRNA, therefore inhibiting E-cadherin expression at both transcriptional and post-transcriptional level. Finally, a detection method of lymph node metastasis was build based on the dysregulated metabolites, which showed good performance among ESCC patients. For lymph node metastasis of ESCC, this work supports NNMT is a master regulator of the cross-talk between cellular metabolism and epigenetic modifications, which may be a therapeutic target.

## Introduction

Esophageal squamous cell carcinoma (ESCC), the dominant subtype of esophageal cancer, is a common and deadly cancer worldwide, especially in East Asia^1,2^. Regional lymph node metastasis is the key indication of tumor cell dissemination, which is a strong predictor for poor survival of patients with ESCC^3^. A better understanding of tumor microenvironment and metabolism reprograming underlying ESCC lymph node metastasis is urgently needed to develop promising diagnostic and therapeutic strategies.

Metabolic reprogramming has been considered a hallmark of cancer for nearly a decade^4^. Recent works have also revealed the vital role of metabolic reprogramming of malignant cells during cancer metastasis^5,6^. Tasdogan et al. found that higher levels of MCT1 potentiated melanoma metastasis through increased activity of oxidative pentose phosphate pathway^5^. These results indicate that metabolic properties and preferences of malignant cells have been altered during cancer metastasis. Specifically, lymph node metastasis requires the transient activation of cellular programs enabling dissemination and seeding of malignant cells in regional lymph node^7^. In addition, recent data support the concept that metabolic reprogramming in malignant cells is driven by many biochemical changes, including activation of oncogenic and inactivation of tumor suppressive metabolic enzymes^8,9^, which suggests signaling and transcriptional pathways can be regulated by metabolism^8^. Large-scale profiling studies have uncovered numerous metabolic enzymes with altered expression in cancers^10,11^. However, how metabolic reprogramming of malignant cells affects lymph node metastasis is poorly understood in ESCC.

Metabolomics is a promising approach for the identification of metabolites of cells, tissues, biofluids, and other samples, which may provide insights into early diagnosis and therapeutic approaches of cancer^12,13^. Single-cell RNA sequencing (scRNA-seq) profiles gene expression network at the single-cell level, enabling high-resolution characterization of cellular heterogeneity, development, and differentiation states in diverse tumors^14–16^. In our previous study^17^, we discovered lipid metabolism dysregulation in tumor microenvironment (TME) of lung cancer and developed a highly sensitive method for lung cancer detection by integrating scRNA-seq and metabolomics.

In current study, scRNA-seq of paired tumor tissues and lymph nodes of ESCC was performed and aberrant cellular metabolism was observed in various cell types. Then, metabolomics analyses of ESCC tumor tissues and plasma revealed various dysregulated metabolites and metabolic pathways during lymph node metastasis of ESCC. Integrating analyses of scRNA-seq and metabolomic data showed that the highly expressed nicotinamide N-methyltransferase (NNMT) is the key enzyme contributing to the metabolic changes, which decreases H3K4me3 and m6A modification of E-cadherin, thereby promoting epithelial–mesenchymal transition (EMT) and metastasis ability of ESCC cells. Furthermore, based on metabolites of NNMT-related pathway, we developed a sensitive method to detect lymph node metastasis of ESCC patients.

## Results

### Tumor microenvironment of ESCC with lymph node metastasis

**Fig. 1:**
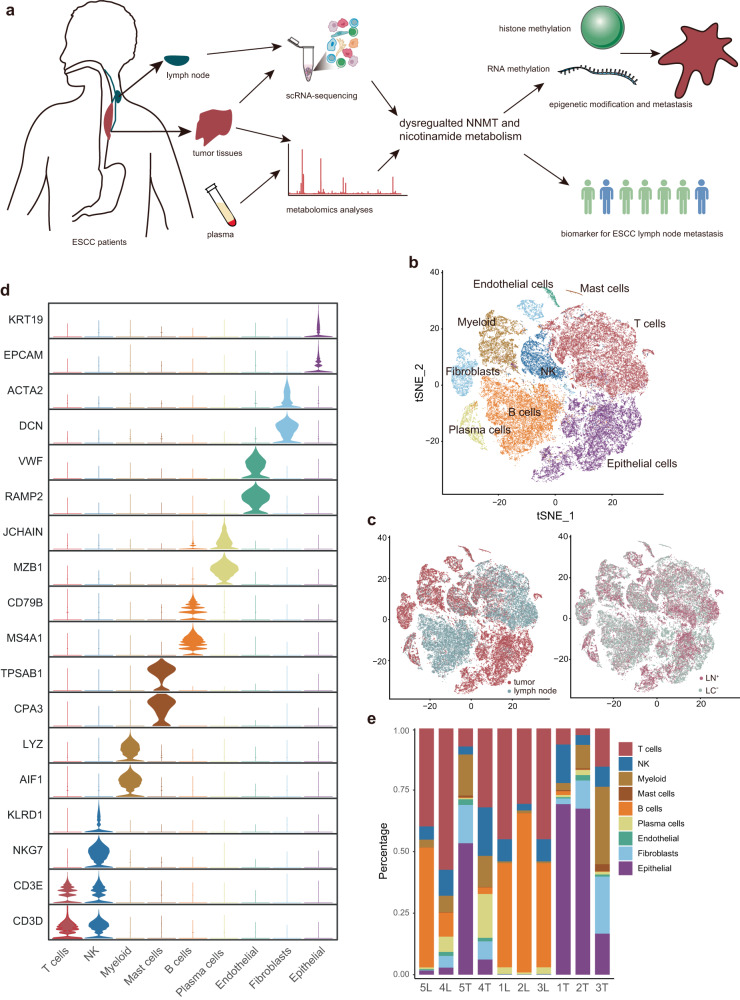
Cellular atlas of ESCC tumor tissues and paired lymph nodes. **a** Schematic illustration of scRNA-seq analyses and metabolomics. **b** Cell populations identified in ESCC tumor tissues and paired lymph node samples. The t Stochastic neighbour Embedding (tSNE) plots for 66,864 high-quality cells from tumor tissues (*n* = 5) and lymph node samples (*n* = 5), and nine major cell clusters identified are labeled. Each dot corresponds to a single cell and is colored based on its cell type. **c** TSNE plots colored by sample origin (left), and lymph node metastatic groups (right). **d** Violin plots showing expression distribution of marker genes in nine cell types. **e** The proportion of each cell type in 10 samples. Among all samples, 5 L and 4 L represent mLN; 1 L, 2 L, and 3 L represent nLN; 5 T and 4 T represent LN^+^ ESCC; 1 T, 2 T and 3 T represent LN^−^ ESCC.

To investigate the reprogramming of TME at the single-cell level, five ESCC primary tumors and five matched lymph nodes with (*n* = 2) or without (*n* = 3) metastasis were analyzed by scRNA-seq (Fig. 1a–d and Supplementary Fig. 1a–d). The clinical characteristics of these enrolled participants were shown in Supplementary Table 1. After quality control and removal of the batch effect between batches (Supplementary Fig. 1c, see “Methods”), our dataset included 66,864 total cells, covering various malignant and non-malignant cell types. We identified 9 major cell types based on marker genes expression (Fig. 1d, Supplementary Fig. 1d and Supplementary Table 2): T cells (marked with CD3D and CD2), natural killer (NK) cells (marked with KLRD1), myeloid cells (marked with LYZ and AIF1), mast cells (marked with TPSAB1), B cells (marked with CD79B and MS4A1), plasma cells (marked with JCHAIN and MZB1), endothelial cells (marked with VWF and RAMP2), fibroblasts (marked with DCN and ACTA2), epithelial cells (marked with EPCAM and KRT19). The proportion of each cell types varies greatly among different samples (Fig. 1e). The relative abundance of myeloid cells, T cells and plasma cells tended to be higher in LN^+^ group than in non-lymph node metastasis (LN^−^) group (Supplementary Fig. 1b). In addition, epithelial cells can be found in lymph node with metastasis (mLN), while the relative proportion of that in lymph node without metastasis (nLN) was little (Supplementary Fig. 1b).

### Metabolic reprogramming of immune cells in tumor microenvironment

**Fig. 2:**
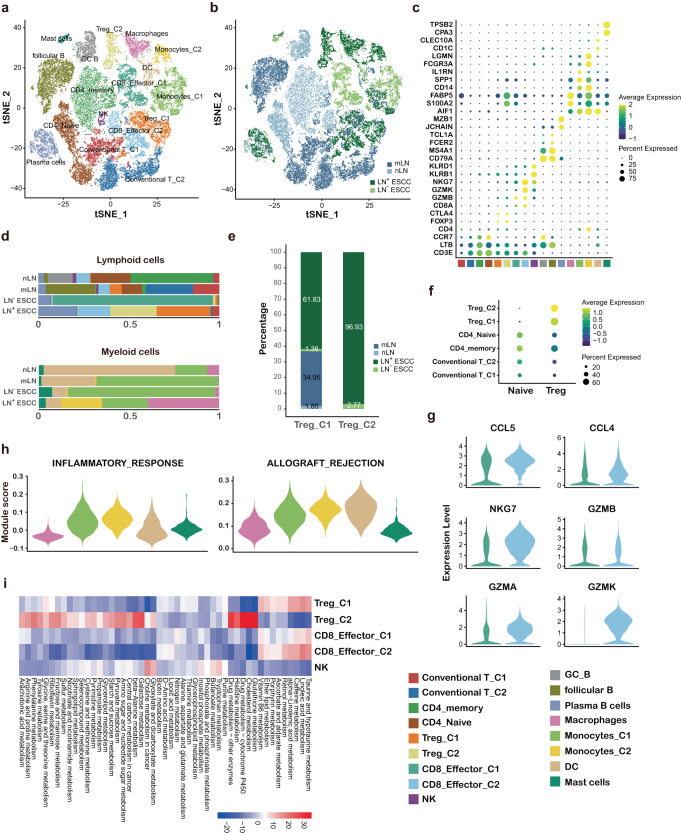
Composition of the immune microenvironment of ESCC. **a** TSNE plot of all immune cells colored by cell subtypes. **b** TSNE plot of immune cells colored by tissue types. **c** Average gene expression of selected marker genes for immune cell subtypes, for cell subtype color code see (**a**). **d** The relative quantification of lymphoid and myeloid cell subtypes in each tissue type. **e** The relative quantification of Treg cells in each tissue type, color code for tissue type is consistent with (**b**). **f** Dot plot of representative naïve and Treg signatures in conventional and CD4 T cell subtypes. **g** Violin plot showing the CCL5, CCL4, NKG7, GZMB, GZMA, and GZMK in CD8-effector-C1 and CD8-effector-C2 cells. **h** Module scores of gene signatures related to inflammation of different macrophage subtypes, for cell cluster color code see (**a**). **i** Heatmap showing differences in metabolic pathways scored per cell by GSVA among Treg, CD8 T and NK cells.

We re-clustered lymphocytes and identified different subtypes of T, NK, B, and plasma cells (Fig. 2a–e) based on typical marker genes (Fig. 2c). Specifically, 23,424 (75.28%) lymphocytes were obtained from lymph nodes (Fig. 2b and Supplementary Fig. 2a). Although lymphocytes usually encompassed both LN^+^ and LN^−^ tissue-derived transcriptomes, we noted quantitative shifts in the cellular composition of the tumor immune microenvironment (TIME) (Fig. 2d). To investigate gene networks in CD8 T cells and CD4 T cells, we used the public naïve, Treg, and cytotoxic signatures^18^ (Supplementary Table 3) and applied these signatures to CD8 and CD4 subtypes and computed transcriptional scores (Fig. 2f and Supplementary Fig. 2b). In CD8 T cells, CD8_effector_C1 and CD8_effector_C2 both expressed CCL5, CCL4 and effector molecular such as NKG7, GZMA and GZMB, while CD8_effector_C2 expressed a higher level of GZMK than CD8_effector_C1 (Fig. 2g), which indicated that distinct states of CD8 T cells in TIME^19^. Dr. Hornburg and colleagues have reported that CD8 + GZMK T cells represent pre-dysfunctional effector memory cells in ovarian cancer^19^. For CD4 T cells, Treg formed two distinct clusters, including Treg_C1 and Treg_C2, consistent with the Treg signatures as described previous (Fig. 2e, f). We observed that Treg_C1, marked by expression of FOXP3 and CTLA4, was enriched in LN^+^ ESCC and mLN (Fig. 2e), indicating the more suppressive TIME in LN^+^ ESCC. Intriguingly, Treg_C2, marked by high level expression of FABP5, was almost solely detected from the LN^+^ ESCC tissues (96.93%) (Fig. 2e). Lipid chaperones, such as fatty acid binding proteins (FABPs), are critical in cellular metabolism^20^. In addition, FABP5, one of the most highly expressed FABPs in T cells^21^, is essential for mitochondrial oxidative phosphorylation and lipid metabolism in Treg. Recent study revealed that Tregs may augment FABP5 in response to low-lipid environments^22^, which supports that lipid metabolism has been altered in TIME for LN^+^ ESCC. Further comprehensive dissection of metabolic profiles suggested that Treg_C2 had distinct metabolic patterns compared to CD8 T and NK cells, as expected. Specifically, some metabolic pathways were highly expressed in Treg_C2, including glutathione metabolism; histidine metabolism; and galactose metabolism. Collectively, these data indicate that metabolic reprogramming is widely occurred in TIME of LN^+^ ESCC, especially lipid metabolism and glutathione metabolism.

In addition, B cells have been revealed to enormously impact molding cancer immune response and are closely related to prognosis of patients^23^. A total of 7347 B cells were analyzed, and three subtypes were identified: follicular B cells, germinal center B cells and plasma B cells (Fig. 2a). The relative abundance of plasma B cells increased stepwise from nLN, mLN to LN^−^ ESCC and LN^+^ ESCC (Fig. 2d). Gene set variation analysis (GSVA) revealed that epithelial–mesenchymal transition (EMT) and angiogenesis were upregulated in plasma B cells (Supplementary Fig. 2c), which suggests plasma B cells may participate in LN metastasis of ESCC.

Since myeloid cells have been shown to be fundamental in regulating both innate and adaptive immune responses and facilitating tumor invasion and metastasis^24^, we detected five subtypes of myeloid cells, including macrophages, monocyte_C1, monocyte_C2, DC cells and mast cells (Fig. 2a). Intriguingly, monocyte_C2 was almost solely observed from LN^+^ ESCC, compared to other tissues (Fig. 2d). In addition, monocyte_C2 also showed high scores of immune response related signature^25^, together with monocyte_C1 and DC cells^26^ (Fig. 2h), which indicates that they represent proinflammatory functional state among all of the myeloid subtypes. Analysis of metabolic pathway gene signature highlighted monocyte_C2 had distinct metabolic pattern compared to other subtypes (Supplementary Fig. 2d). Specifically, some metabolic pathways were exclusively upregulated in monocyte_C2, such as nicotinate and nicotinamide metabolism; arginine and proline metabolism; and histidine metabolism, which further confirm that metabolic reprogramming in LN^+^ ESCC.

### Myofibroblasts are highly abundant in LN^+^ ESCC

Then, stromal cells of ESCC TME and lymph nodes were examined. We detected two subtypes of ECs, including tumor ECs and lymphatic ECs (Supplementary Fig. 3a, b). GSVA revealed that p53 pathway was enriched in ECs from LN^+^ group compared with LN^−^ group (Supplementary Fig. 3c).

**Fig. 3:**
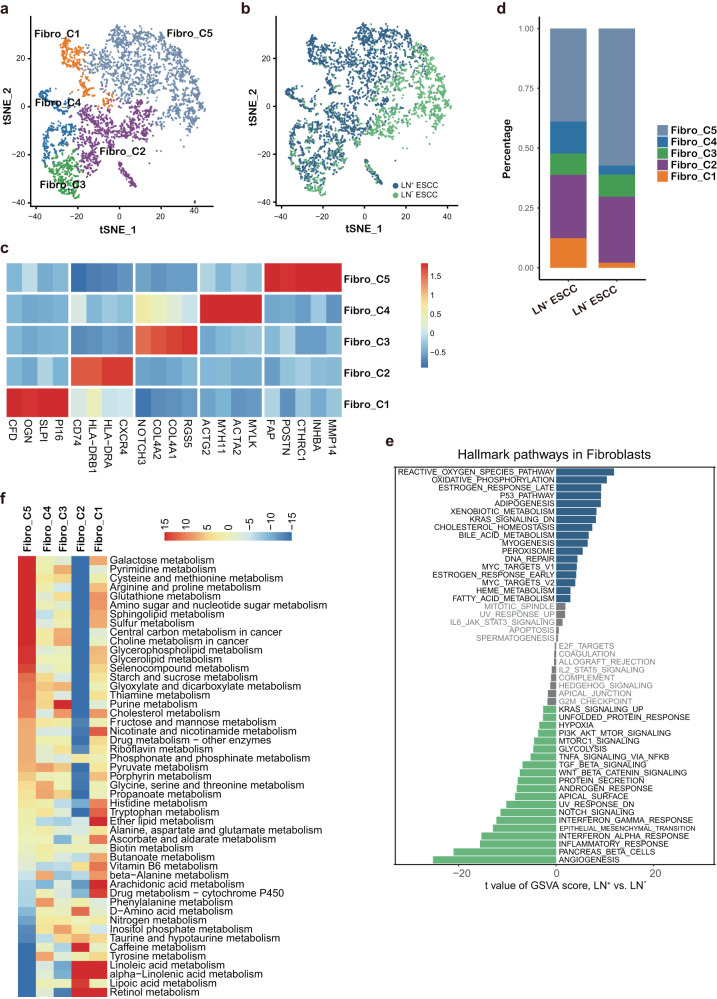
Fibroblasts in the TME of ESCC. **a** TSNE plot of fibroblasts, showing the composition of five main subtypes. **b** TSNE plot showing fibroblast from LN^+^ and LN^−^ groups. **c** Heatmap of marker genes expression in fibroblasts. **d** Average proportion of each fibroblast subtype between LN^+^ and LN^−^ groups. **e** Differences in pathway activities scored per cell by GSVA between LN^+^ and LN^−^ group. **f** Metabolic pathways are scored per cell by GSVA among five fibroblast subtypes. The relative activity scores were obtained from a linear model by limma and sorted by pathway activity in Fibro_C5 cells.

Among 4151 fibroblasts, we identified 5 subtypes of fibroblasts based on marker genes, including normal fibroblasts (Fibro_C1), immune-modulatory fibroblasts (Fibro_C2), pericytes (Fibro_C3), myofibroblasts (Fibro_C4), cancer-associated fibroblasts (CAFs) (Fibro_C5) (Fig. 3a–c and Supplementary Fig. 3d–h). CAFs play important roles in creating extracellular matrix structure and metabolic reprogramming of TME^27^. In addition to collagens broadly expressed in all subtypes, CAFs uniquely expressed collagens X and XI (Supplementary Fig. 3g), suggesting functional specialization of tumor-supported collagens. CAFs was also characterized by high activity of TGFβ and hypoxia-induced pathways (Supplementary Fig. 3h), which are known features of CAFs^28^. Our scRNA-Seq data showed that myofibroblasts was more abundant in LN^+^ group than LN^−^ group (Fig. 3d), which was also observed in other cancers that myofibroblasts could promote extensive tissue angiogenesis^29^ and tumor progression^27,30^. Besides, JAK/STAT signaling was also upregulated in myofibroblasts (Supplementary Fig. 3h), as it has been reported that activation of JAK/STAT cascade in cancer-associated myofibroblasts play an important role in promoting the invasion and metastasis of cancer^28,31^. A direct comparison of fibroblasts from LN^+^ group and LN^−^ group revealed that many metabolic pathways were significantly up-regulated in fibroblasts of LN^+^ group, including oxidative phosphorylation; and bile acid metabolism; and fatty acid metabolism; and HEME metabolism (Fig. 3e). Further comprehensive dissection of metabolic profiles suggested that CAFs showed a opposite metabolic pattern to Fibro_C2 cells (Fig. 3f), which may explain their distinguish contribution to ESCC progression and further support metabolomic reprogramming in LN^+^ group.

### Metabolic reprograming of malignant cells in LN^+^ ESCC

**Fig. 4:**
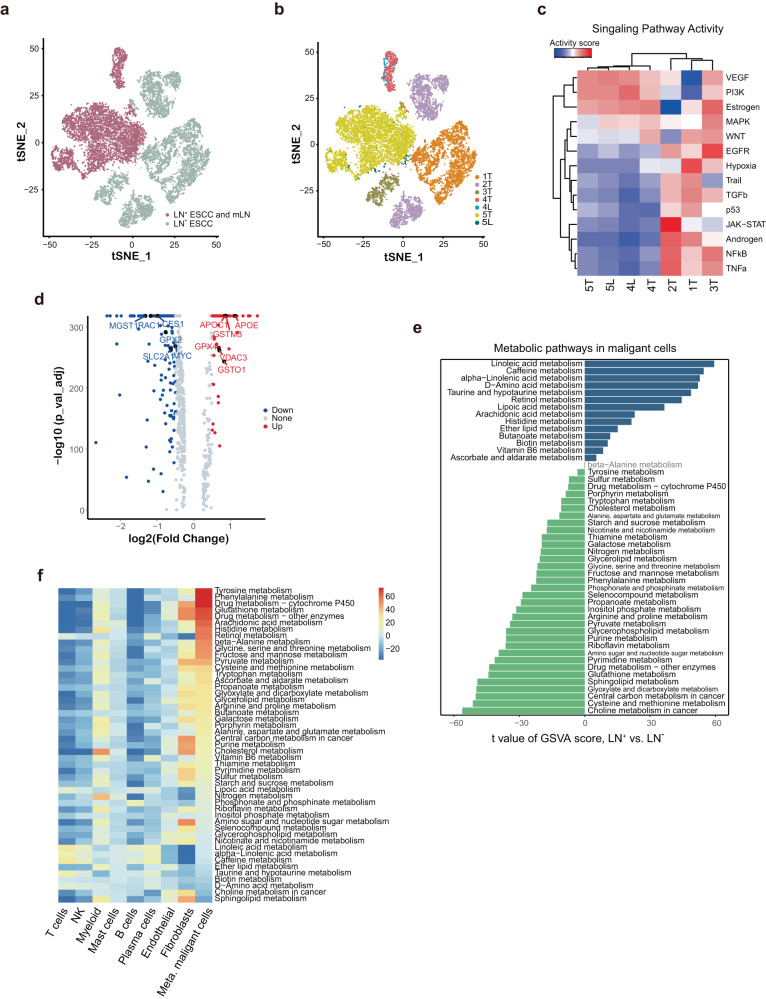
Metabolic pathways were broadly altered in LN^+^ESCC. **a**, **b** The TSNE projection of cells from five ESCC patients, showing LN^+^ group and LN^−^ group. **a** Cancer cell identification was inferred by CNV sub-clusters and was highly patient-specific. **b** Each dot corresponds to a single cell, with each sample represented by a different color. **c** Mean pathway activity scores of tumor epithelial cells grouped by sample. **d** The volcano plot shows DEGs between LN^+^ ESCC and LN^−^ ESCC maligant cells. The genes related to metabolic pathways are labeled with their gene names. The red and blue points indicate up and down-regulated DEGs in maligant cells, respectively. **e** Differences in pathway activities scored per cell by GSVA between LN^+^ and non-metastasis groups. **f** Metabolic pathway expression profiles in the LN^+^ group. For each pathway, the fold change in malignant cells was calculated by comparing to other cell types and corrected for sample of origin. Pathways were ordered by log-fold change in malignant cells, respectively.

By analyzing the transcriptome patterns of epithelial cells in 7 samples that had > 100 epithelial cells (Fig. 1e and Supplementary Table 4), we distinguished malignant cells from normal stromal and immune cells by inferring largescale copy number variations (CNVs)^32^ from expression profiles as reported (Fig. 4a, b and Supplementary Fig. 4a). We found that malignant cells formed clusters according to sample origin, suggesting a high degree of inter-tumor heterogeneity (Fig. 4b). Next, we quantified oncogenic pathways expression between LN^+^ and LN^−^ groups, and results showed high activities of TNFα, TGFβ, and NF-kB signaling in LN^+^ group and high activities of VEGF and PI3K signaling in LN^−^ group (Fig. 4c). This indicated high intra-group similarity in LN^+^ group and significant distinction between LN^+^ group and LN^−^ ESCC. A direct comparison of malignant cells from LN^+^ ESCC and LN^−^ ESCC showed that many differentially expressed genes (DEGs) were involved in metabolism related processes (Fig. 4d and Supplementary Fig. 4b). Furthermore, we compared LN^+^ ESCC and paired LN, and result showed that the DEGs were also enriched in several metabolic process, such as ATP metabolic process and quinone metabolic process (Supplementary Fig. 4c, d). Then, we characterized global metabolic changes in malignant cells by comparing with other cell types within LN^+^ group and LN^−^ ESCC, respectively. Results suggested that malignant cells in LN^+^ group and LN^−^ ESCC showed distinct metabolic patterns (Fig. 4e, f and Supplementary Fig. 4e). Specifically, linoleic metabolism, and histidine metabolism were upregulated in LN^+^ malignant cells, whereas glutathione metabolism was enriched in LN^−^ malignant cells (Fig. 4e). Together, these analyses demonstrate that metabolism was widely dysregulated in malignant cells of lymph node metastatic ESCC.

### Increased nicotinate and nicotinamide metabolism in LN^+^ ESCC

**Fig. 5:**
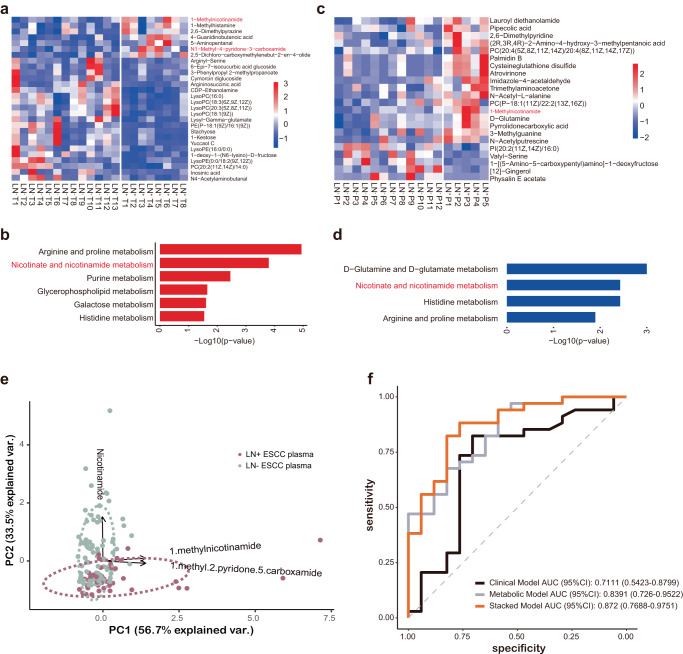
Increased nicotinate and nicotinamide metabolism in LN^+^ ESCC. **a** Heatmap of normalized metabolite abundance in LN^+^ and LN^−^ ESCC tissues. **b** The enriched pathway of differentiated metabolites in tissue samples of ESCC. **c** Heatmap of normalized metabolite abundance in plasma from LN^+^ and LN^−^ ESCC patients. **d** The enriched pathway of differentiated metabolites in plasma samples from LN+ and LN^−^ ESCC patients. **e** Principal component analysis showed stratification of plasma samples based on abundance 3 metabolites. PC1 and PC2 values represent the top two principal coordinates. Different sample types were denoted by color code. **f** Performance of various predictive models in forms of receiver operating characteristic (ROC) curves and area under curve (AUC) scores, based on the 51-patient test set. The performance of various predictive models based on different feature sets, namely clinical, metabolic, and integrated model, respectively. The ROC curves of models are shown as lines of different colors. AUC and the 95% CI of each model are shown in the legend.

Untargeted metabolomics was performed to directly investigate reprogramming of LN^+^ ESCC at metabolites level. Tumor tissues of 8 LN^+^ and 13 LN^−^ ESCC (Fig. 5a and Supplementary Fig. 5a) were analyzed, and results suggested 1-MNA and its downstream product, 1-methyl-4-pyridone-5-carboxamide (4-PYR), were both increased in tumor tissues of LN^+^ ESCC. Accordingly, pathway analysis revealed that nicotinate and nicotinamide metabolism was highly upregulated in LN^+^ ESCC (Fig. 5b). Consistent with scRNA-seq data, histidine metabolism was also enriched in LN^+^ ESCC tissues (Fig. 5b). In a large-scale scRNA-seq dataset^33^ including 21,788 cells (69.16%) originated from 28 LN^+^ ESCC patients and 9,718 cells (30.84%) from 16 LN^−^ ESCC patients (Supplementary Fig. 5c–e), differentially expressed genes of malignant cells from LN^+^ ESCC were also significantly enriched in nicotinate and nicotinamide metabolism and pathways, further convincing the untargeted metabolomics results of ESCC tissues.

Then, we used untargeted metabolomics to detect plasma of LN^+^ and LN^−^ ESCC patients. Intriguingly, significant upregulation of 1-MNA was found in plasma of LN^+^ ESCC compared with that of LN^−^ ESCC (Fig. 5c and Supplementary Fig. 5b). In addition, pathway analysis revealed that nicotinate and nicotinamide metabolism was highly upregulated in LN^+^ ESCC, consistent with metabolomics data from tumor tissue samples (Fig. 5d). Thus, both metabolomics data of tissues and plasma demonstrated that nicotinate and nicotinamide metabolism was upregulated in LN^+^ ESCC.

Since lymph node metastasis is a critical factor for clinical decision^34^, we then test whether metabolites of nicotinate and nicotinamide metabolism pathway could detect LN metastasis of ESCC. Nicotinamide, MNA, and 1-methyl-2-pyridone-5-carboxamide (2-PYR) were finally selected to construct a targeted metabolomic detection method and the detecting performance was tested in a cohort of 130 ESCC patients (43 LN^+^ ESCC and 87 LN^−^ ESCC, Supplementary Table 9). As shown in Fig. 5e, based on the 3 metabolites, LN^+^ ESCC could be distinguished from LN^−^ ESCC. Then, the 130 ESCC patients were split into a training set (79-patient) and a test set (51-patient). In the training set, a partial least squares (PLS) model with the 3 metabolites-based biomarkers was trained, which achieved a high accuracy in LN^+^ ESCC prediction in the test set (area under receiver operating characteristic curve (AUC) = 0.8391), which contained 17 LN^+^ ESCC patients and 34 LN^−^ ESCC patients (Fig. 5f). Clinical information of ESCC patients (age, sex, smoking history, alcohol intake history, tumors history, and pathologic tumor stage (T stage)) had AUC of 0.7111 in the test set. Specifically, T stage showed statistical significance among the 6 clinical features in differentiating LN^+^ ESCC and LN^−^ ESCC groups (Supplementary Table 9) and univariate prediction AUC = 0.7279. Next, we combined the metabolic model based on 3 selected metabolites and T stage to construct an integrated model. As expected, the integrated model achieved improved performance (AUC = 0.872, Fig. 5f) in the test set, with sensitivity 0.7647 and specificity 0.8824 (Supplementary Table 10).

### NNMT promotes ESCC cell metastasis in vitro and in vivo

**Fig. 6:**
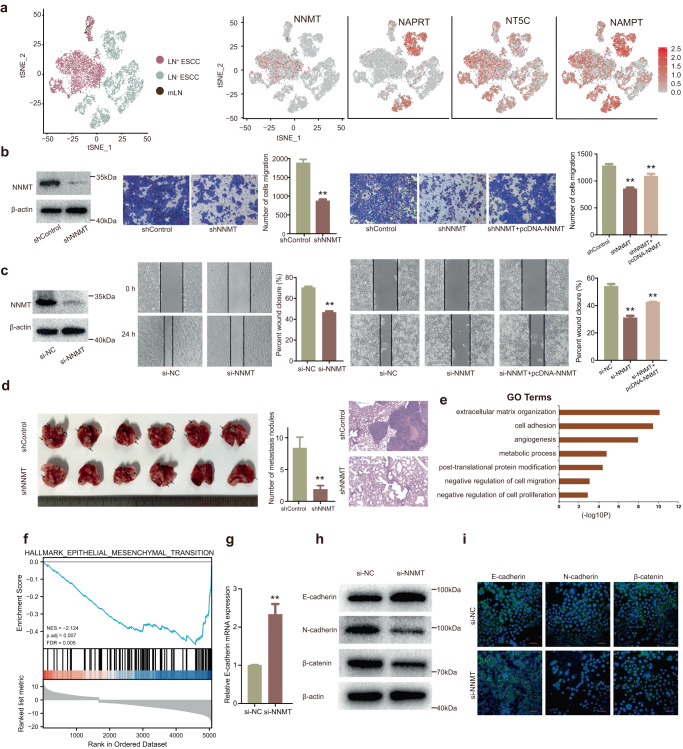
NNMT promotes ESCC cell metastasis in vitro and in vivo. **a** The tSNE plot of tumor cells, colored by tissues (left) and expression of four key enzymes of nicotinate and nicotinamide metabolism pathway (right). **b** NNMT was analyzed by western blotting after knockdown of NNMT in Eca-109 cells. Then transwell was used to investigate the cell migration ability after corresponding transfection. **c** After corresponding transfection, wound healing assays were used to analyze the migration ability of Eca-109 cells. **d** Eca-109 cells with NNMT knockdown were injected into mice through the tail vein to analyze animal models of tumor metastasis. **e** GO analysis for all genes with altered expression after knockdown of NNMT in Eca-109 cells. **f** GSEA showed that genes in response to NNMT knockdown were enriched for gene sets significantly related to the epithelial mesenchymal transition. **g** The mRNA level of E-cadherin was confirmed by qRT-PCR in Eca-109 cells with NNMT knockdown. **h** The protein levels of E-cadherin, N-cadherin and β-catenin were detected by western blot in Eca-109 cells. **i** Immunofluorescence staining of E-cadherin, N-cadherin, and β-catenin (green) in the Eca-109 cells after treatment. The blue signal represents the nuclear DNA staining by 4′,6-diamidino-2-phenylindole.

Since both metabolomics data from tumor tissues and plasma confirmed nicotinate and nicotinamide metabolism was upregulated in LN^+^ ESCC, we then tried to discover the underlying mechanism. We first retrieved key enzymes involved in nicotinate and nicotinamide metabolism pathway from database. Then, we reviewed the relevant literature on these enzymes and identified NAMPT^35^, NAPRT^36,37^, NNMT^11,38^, and NT5C^39^ as key enzymes for further investigation. Among them, high expression of NNMT was mainly found in malignant cells of LN^+^ ESCC and barely no expression was observed in LN^−^ ESCC (Fig. 6a). NNMT is a metabolic enzyme transferring methyl group from S-adenosyl-L-methionine (SAM) to nicotinamide (NA) and generating S-adenosyl homocysteine (SAH) and 1-MNA, which is consistent with scRNA-seq and metabolomics findings. Additionally, this finding was also validated in external scRNA-sea data (Supplementary Fig. 5d). Thus, we proposed high expression NNMT contribute to nicotinate and nicotinamide metabolism reprograming in LN^+^ ESCC.

To investigate the biological role of NNMT in LN^+^ ESCC, validated small interfering RNA (siRNA) sequences specifically targeting NNMT were used^40^. As shown in Supplementary Fig. 6a, the expression of NNMT was obviously inhibited. Transwell and wound-healing assays showed that downregulation of NNMT expression could significantly inhibit cell migration ability in Eca-109 cells (Fig. 6b, c). Furthermore, we found that overexpression of NNMT could partly reverse NNMT knockdown-mediated migration suppression (Fig. 6b, c, right panel). The overexpression efficiency of NNMT was shown in Supplementary Fig. 6b. To further verify the metastasis-promoting effects of NNMT in vivo, Eca-109 cells stably transfected with sh-NNMT and control vector were injected into the tail vein of mice to establish a metastasis model. Compared with the control group, the number of metastatic nodules at the lung surface was reduced after NNMT knockdown (Fig. 6d). In addition, as shown in Supplementary Fig. 6c–f, knockdown of NNMT also significantly inhibited proliferation of ESCC cells. And overexpression of NNMT could partly reverse si-NNMT-mediated proliferation suppression. Moreover, knockdown of NNMT could also inhibit proliferation of ESCC in vivo (Supplementary Fig. 6g).

### NNMT promotes EMT in ESCC by inhibiting expression of E-cadherin expression at transcriptional and post-transcriptional level

To ascertain the mechanism of NNMT and identify the key downstream gene of NNMT in ESCC, we performed RNA-sequencing after NNMT knockdown (Supplementary Table 12). GO analysis found that the most upregulated biological processes were extracellular matrix organization, cell adhesion, angiogenesis, metabolic process, post-translational protein modification et al. (Fig. 6e). Moreover, gene set enrichment analysis (GSEA) revealed that gene sets related to the epithelial mesenchymal transition (EMT) were significantly enriched (Fig. 6f). Importantly, the key gene of the EMT pathway, E-cadherin, was upregulated after NNMT knockdown (Supplementary Table 12), which was validated by qRT-PCR (Fig. 6g). Furthermore, both western blot and immunofluorescent staining assays confirm that knockdown of NNMT upregulated E-cadherin expression and inhibited N-cadherin and β-catenin (Fig. 6h, i). Thus, we speculated that NNMT may promote metastasis of ESCC by inducing EMT.

**Fig. 7:**
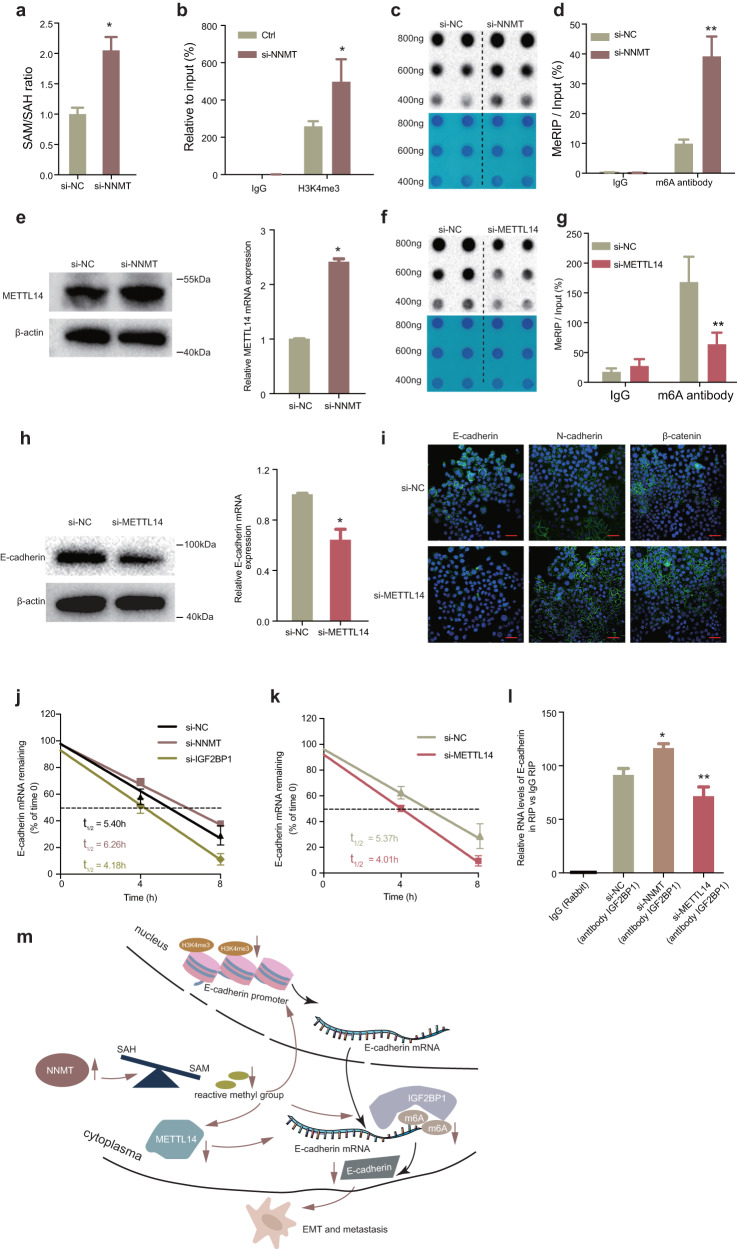
NNMT promotes EMT in ESCC by inhibiting E-cadherin expression transcriptionally and post-transcriptionally. **a** The SAM/SAH ratio significantly increased after silencing NNMT in Eca-109 cells. **b** ChIP-qPCR of H3K4me3 of the promoter region of the E-cadherin locus after siRNA treatment targeting si-NNMT in Eca-109 cells. Antibody enrichment was quantified relative to the amount of input DNA. An antibody directed against IgG was used as a negative control. **c** The m6A dot blot assay was used to investigate the global m6A abundance after knockdown of NNMT compared with the control group in Eca-109 cells. **d** MeRIP-qPCR was performed to quantify the relative m6A modification level of E-cadherin upon NNMT knockdown in Eca-109 cells. **e** qRT-PCR assays and western blot assays detected the mRNA and protein levels of METTL14 after knockdown of NNMT expression in Eca-109 cells. **f** The m6A dot blot assay was used to investigate the global m6A abundance after silencing METTL14 compared with the control group in Eca-109 cells. **g** MeRIP-qPCR was performed to quantify the relative m6A modification level of E-cadherin after knockdown of METTL14 in Eca-109 cells. **h** qRT-PCR and western blot assays detected the mRNA and protein levels of E-cadherin after siRNA treatment of METTL14 in Eca-109 cells. **i** Immunofluorescence staining of E-cadherin, N-cadherin, and β-catenin (green) in the Eca-109 cells expressing after knockdown of METTL14. **j** Lifetime of E-cadherin mRNA levels in Eca-109 cells with NNMT and IGF2BP1 knockdown. **k** Lifetime of E-cadherin mRNA levels after silencing METTL14 in Eca-109 cells. **l** A RIP experiment for IGF2BP1 was performed in Eca-109 cells, and the coprecipitated RNA was subjected to qRT-PCR for E-cadherin after transfection of si-NC, si-NNMT and si-METTL14. The fold enrichment of E-cadherin in RIP is relative to its matching IgG control RIP. *P < 0.05, **P < 0.01. **m** Schematic map showing that NNMT promotes EMT of ESCC via decreasing H3K4me3 in E-cadherin promoter region at transcriptional level and inhibiting m6A modification of E-cadherin in an m6A-dependent manner at post-transcriptional level.

Then we assessed SAM and SAH by ELISA in Eca-109 cell and found NNMT knockdown indeed increased the SAM/SAH ratio (Fig. 7a). Using SAM as methyl donor, NNMT catalyzes N-methylation of nicotinamide to generate 1-methylnicotinamide (MNA) and SAH^38^. NNMT converts active methyl donor (SAM) to stable methylated metabolite (MNA), thus linking cellular metabolism and epigenetic modifications. Previous researchers have found that NNMT could transcriptionally regulate gene expression by affecting H3K4me3 in gene promoter regions^11^. Then we performed chromatin immunoprecipitation (ChIP) assays to examine whether NNMT was associated with H3K4me3 occupy at E-cadherin promoter. The results showed that the E-cadherin promoter was enriched with endogenous H3K4me3, and knockdown of NNMT expression increased H3K4me3 modification (Fig. 7b).

In addition, as a post-transcriptional modification, m6A RNA methylation is the most prevalent chemical modification at the epigenetic level of RNA, which also consumes methyl donor^41^ and belongs to SAM (as the methyl donor)-dependent process^42^. Therefore, we hypothesized that NNMT may participate in m6A modification and modulate E-cadherin expression at post-transcriptional level by tuning intracellular methyl groups. Firstly, we analyzed the m6A modification sites in the E-cadherin mRNA sequences by SRAMP^43^. As shown in Supplementary Fig. 7a, many predicted m6A sites existed in E-cadherin mRNA with very high confidence, indicating m6A modification may participate in the expression of E-cadherin. M6A dot blot assay conformed the increased overall m6A level after NNMT knockdown, indicating that NNMT could affect the overall m6A modification level in Eca-109 cells (Fig. 7c). Combining the above results, we speculated that NNMT might suppress E-cadherin through m6A modification, thus promoting EMT of ESCC. To further verify the mechanism between NNMT and E-cadherin, we performed methylated RNA immunoprecipitation-qPCR (MeRIP-qPCR) assay and found a consistently increase m6A level in the CDS region of E-cadherin after NNMT knockdown (Fig. 7d). Thus, we proved that NNMT inhibits expression of E-cadherin not only by histone modification dependent manner, but also m6A modification dependent manner.

M6A modification requires the participation of RNA methyltransferases. M6A methyltransferase, Methyltransferase-like 3 (METTL3), METTL14, Wilms tumor 1-associated protein (WTAP) and KIAA1429 (also known as VIRMA), termed “writers”, catalyze the formation of m6A RNA^44^. Since NNMT is activated in ESCC, it is suggested that it may consume more methyl groups, resulting in less methyl groups for m6A modification process, and m6A modification is at a disadvantaged level in cells. This implies that m6A modification mediated by “writers” in dialogue with NNMT is attenuated. We analyzed these four “writers” and found that only METTL14 was downregulated in ESCC (Supplementary Fig. 7b, c). Therefore, we speculate that NNMT may post-transcriptionally regulate E-cadherin in a m6A-modified manner by communicating with METTL14. Then we found that knockdown of NNMT indeed upregulated METTL14 expression at the RNA and protein levels (Fig. 7e). Dot blot assay showed that METTL14 was essential for m6A modification since the overall m6A level dramatically declined upon METTL14 silence in ESCC (Fig. 7f and Supplementary Fig. 7d). M6A level in the CDS region of E-cadherin was downregulated after METTL14 knockdown (Fig. 7g) and knockdown of METTL14 could indeed inhibit the expression of E-cadherin (Fig. 7h). Therefore, these data suggest that METTL14 can post-transcriptionally regulate E-cadherin expression in an m6A modification-dependent manner.

To further clarify the functional role of METTL14 in the EMT process, immunofluorescent staining assays found knockdown of METTL14 inhibited the expression of E-cadherin and increased N-cadherin and β-catenin expression (Fig. 7i). Since m6A modification requires the cooperative participation of writer and reader, next, we investigated how m6A affected expression of E-cadherin. IGF2BP1 has been reported as a m6A “reader” proteins to selectively recognize m6A modifications and enhance m6A-containing mRNA stability^45^. This led to hypothesize that m6A-containing in mRNA of E-cadherin might be recognized and bound by IGF2BP1. To test whether IGF2BP1 could recognize m6A modification of E-cadherin, we used siRNA to knockdown IGF2BP1 (Supplementary Fig. 7e). To further verify the influence of NNMT, IGF2BP1 and METTL14 on the RNA stability of E-cadherin, we treated Eca-109 cells with the transcription inhibitor actinomycin D after knockdown of NNMT, IGF2BP1 and METTL14. Indeed, we found that knockdown of NNMT increased the half-life of E-cadherin mRNA, whereas knockdown of IGF2BP1 promoted mRNA degradation of E-cadherin (Fig. 7j). Conversely, the half-life of E-cadherin transcripts was significantly decreased after silencing METTL14 (Fig. 7k). These results indicated that the m6A modification of E-cadherin mRNA could be recognized and bound by IGF2BP1. Moreover, RIP assay further confirmed that the IGF2BP1 protein could directly bind to E-cadherin mRNA. The binding abundance of IGF2BP1 on E-cadherin mRNA increased significantly after silencing NNMT but inhibited by METTL14 knockdown (Fig. 7I). Taken together, these results showed that abnormal histone modification and m6A modification mediated inhibition of E-cadherin expression to promote metastasis of ESCC through NNMT-mediated regulation of EMT (Fig. 7m).

## Discussion

Increasing studies have revealed that metabolism of different cancer types is of high heterogeneity^46,47^. Specifically, metabolic pattern of tumor cells has been found as the major contributor to metabolic heterogeneity in TME^47^. Here, we dissected metabolic reprogramming of ESCC TME during lymph node metastasis at single-cell resolution. In this study, we uncovered the oncogenic role of NNMT in ESCC lymph node metastasis by promoting nicotinate and nicotinamide metabolism and exerting broad influence to epigenetic landscape of cancer cells. We observed that Tregs had greater metabolic activities of glutathione metabolism^48^ than CD8 T and NK cells, especially in the LN^+^ group. This reinforces the emerging theme that immunosuppressive cells undergo remarkable reprogramming and have higher levels of metabolic activities^47,49^. Furthermore, we constructed a integrate model based on 3 selected metabolites and clinical information, and this method can precisely detect lymph node metastasis among ESCC patients.

scRNA-seq help us to understand TME composition and metabolic patterns of each type of cells at single-cell resolution, which is a powerful tool to discover metabolism reprogramming during cancer progression. scRNA-seq can find alterations of metabolism pathways in each cell at transcriptional level. Metabolomics identify and quantify low molecular weight metabolites in a biosystem, which are endpoint products of each pathway. Thus, integrating scRNA-seq and metabolomics can help better understand metabolic reprograming of TME, and metabolomics is also validation of scRNA-seq findings to some degree. In addition to nicotinate and nicotinamide metabolism, both our scRNA-seq and metabolomics data suggest that histidine metabolism is significantly enriched in LN^+^ ESCC. This finding suggest histidine metabolism might play critical roles in ESCC and the underling molecular mechanism need further investigation. Another advance of our study is successful development of a precise method for lymph node metastasis detection based on targeted metabolomics. Since lymph node metastasis is a key factor for clinical decision, this metabolomics detecting method will promote precise treatment of ESCC.

Our data revealed that NNMT could regulate not only the histone methylation modification of E-cadherin at the transcription level, but also m6A RNA methylation at the post-transcriptional level, thus promoting EMT of ESCC. M6A RNA methylation is the most prevalent chemical modification at the epigenetic level of RNA, which plays as important regulatory role in tumorigenesis and metabolic remodeling^41,50^. It follows that NNMT can directly communicate between histone methylation and RNA methylation, thus shaping vital roles in epigenetic modification. These findings indicate NNMT is key enzyme bridging cellular metabolism and epigenetic modifications, and might be a promising biomarker and therapeutic target of ESCC. Our data also confirmed the fact that metabolism reprogramming observed in cancer not only serve to fulfill the proliferative demand of tumor cells but also play a significant role in driving cancer development.

The results of our pathway enrichment analysis revealed significant disturbances in many interconnected pathways, including but not limited to nicotinate and nicotinamide metabolism; arginine and proline metabolism; and histidine metabolism (Fig. 5b, d). These findings fit well with the previous greater literature. For instance, we observed arginine and proline metabolism was enriched in the LN^+^ group. This reinforce the increased arginase expression and activity in many tumors, such as head and neck^51^, kidney^52^, breast^53^, hepatocellular^54^, and prostate^55^.

Limitations of this study should be acknowledged. Due to the limited patient number fitting the inclusion criteria, only five patients were recruited in our scRNA analysis. To compensate this, we have also applied large-scale externalscRNA dataset as an important supplementary data to validate our findings. Additionally, we built a targeted detection method using nicotinamide, MNA, and 2-PYR, since we failed to develop targeted detection method of 4-PYR or nicotinate. It is reasonable to believe that a targeted method with more metabolites of nicotinamide metabolism pathway may have better diagnostic performance for ESCC LN metastasis.

In summary, we provided evidence for metabolic reprogramming of ESCC with lymph node metastasis by scRNA-seq and metabolomics, and identified NNMT as the key regulator that links metabolic reprograming and epigenetic remodeling. These findings not only deepen our understanding into the metabolic heterogeneity of ESCC, but also guided the development of a useful method for ESCC precise treatment. Our findings provide unique insights into the biology of cancer metastasis and raise the possibility to target metabolism pathways in cancer metastasis.

## Methods

### Human biospecimen collection

Tumor tissues, lymph nodes, and blood samples of ESCC patients who underwent surgery at the Department of Thoracic Surgery of Jiangsu Cancer Hospital and the First Affiliated Hospital of Zhengzhou University were enrolled for scRNA-seq and metabolomics analyses. None of the patients had been treated with chemotherapy, radiation, or any other anti-tumor medicines prior to tumor resection. Detailed patient characteristics are shown in Supplementary Tables 1, 8 and 9. Sex or gender was not considered in the study design. This study was approved by the Ethics Committee Board of Peking University People’s Hospital (2020PHB191-01), and it was performed in compliance with the Declaration of Helsinki Principles. The informed consent was obtained from all patients.

### Sample collection and dissociation for scRNA-seq

For scRNA-seq, five primary tumors and five matched lymph nodes with (*n* = 2) or without (*n* = 3) metastasis were analyzed. Primary tumor tissue and lymph node samples were transported in ice-cold H1640 (Gibco, Life Technologies) immediately after surgical resection. All samples were rinsed with phosphate-buffered saline (PBS; Thermo Fisher Scientific), minced into ~1 mm cubic piece, and ground with a UTTD (ULTRA-TURRAX® Tube Drive) disperser (IKA, Germany). The samples were digested by 0.25% trypsin (Gibco, Life Technologies), terminated by H1640 supplemented with 10% fetal bovine serum (Gibco, Life Technologies), and then transferred to 10 ml of digestion medium containing collagenase IV (100 U/ml; Gibco, Life Technologies) and dispase (0.6 U/ml; Gibco, Life Technologies). The digested samples were filtered through a 70 µm nylon mesh. After centrifuging, the pelleted cells were suspended with ice-cold red blood cell lysis buffer (Solarbio) and filtered with a 40 µm nylon mesh. At last, the pelleted cells were suspended with 1 ml of Dulbecco’s PBS (Solarbio), and the concentrations of live cells and clumped cells were determined using an automated cell counter (Countstar).

### Droplet-based single-cell sequencing

Using the Single Cell 3′ Library and Gel Bead Kit V2 (10X Genomics) and Chromium Single Cell A Chip Kit (10X Genomics), the cell suspension was loaded onto the Chromium single-cell controller (10X Genomics) to generate single-cell gel beads in the emulsion (GEMs) according to the manufacturer’s protocol. In brief, single cells were suspended in PBS containing 0.04% bovine serum albumin. About 10,000 cells were added to each channel, and the target number of cells to be recovered was estimated to be about 6000 cells. The captured cells were lysed, and the released RNA was barcoded via reverse transcription in individual GEMs. Reverse transcription was performed at 53 °C for 45 min, followed by 85 °C for 5 min, after which the temperature was held at 4 °C. Complementary DNA was generated and amplified, after which its quality was assessed using an Agilent 4200. According to the manufacturer’s instructions, scRNA-seq libraries were constructed using the Single Cell 3′ Library Gel Bead Kit V2. Finally, the libraries were sequenced using an Illumina NovaSeq 6000 with a paired-end 150–base pair (PE150) reading strategy (performed by CapitalBio Technology, Beijing).

### scRNA-seq data analysis

Raw gene expression matrices were generated for each sample using the Cell Ranger (version 3.0.2) with reference transcriptome GRCh38. After removal of empty droplets using the Scruble^56^ package (version 0.2.3), the output-filtered gene expression matrices were analyzed by R software (version 4.0.5) with the Seurat^57^ package (version 4.1.0). Single-cell gene expression data of all samples were merged, and transcriptomes were filtered for cells with 200–6000 genes detected, 500–50,000 UMIs counted, fraction of mitochondrial reads < 10%, and fraction of hemoglobin reads < 5%. After removal of low-quality cells, UMI counts were variance-stabilized using scTransform^58^ with 3000 variable features, while regressing out number of UMIs and fraction of mitochondrial reads. To remove batch effects and perform integrated analysis, we used the harmony algorithm^59^ to integrate 10 datasets. The details of the integration methods are described at https://github.com/immunogenomics/harmony.

### Dimension reduction and identification of major cell clusters

To reduce the dimension of the integrated object, the resulting variably expressed genes were summarized by the RunPCA() function with default parameters. A subset of significant PCs was identified according to the results of the ElbowPlot(), DimHeatmap() and JackStrawPlot() functions. Next, we clustered cells using the FindNeighbors() and FindClusters() functions and performed nonlinear dimensional reduction with the RunTSNE() function with default settings. Last, we used the FindAllMarkers function in Seurat to find markers for each of the identified clusters and annotated clusters on the basis of expression of canonical markers of particular cell types. For epithelial cell clusters, the InferCNV package5 was used to detect the CNVs in EPCAM+ cells and to recognize ESCC cancer cells with default parameters. Stromal cells and immune cells were used as the control group, and their CNV estimates were used to define a baseline.

### Subclustering of the major cell types

To identify subclusters within major cell types, we reanalyzed cells belonging to each of these major cell types separately. Based on the integrated assay, the ScaleData() and RunPCA() functions were performed. The pipelines of significant PC selection, cell clustering and TSNE visualization were the same as those described above. Finally, we identified each cell sub-cluster based on the expression of canonical markers.

### Differential expression genes (DEGs) identification

Differential expression analysis comparing cells from metastasis or non-metastasis groups was performed using the FindMarkers function with the following parameters: fraction of expressing cells inside the cluster ≥ 0.25, log fold change between cells inside and outside the cluster ≥ 0.25. |log2(fold-change)| > 0.5 and adj.p.val < 0.01 were used as the cut-off criteria. Enrichment analysis for the functions of the DEGs was performed using the Metascape webtool (www.metascape.org).

### Gene set variation analysis (GSVA)

Pathway analyses were performed using Hallmark gene sets from v7.5.1 of the MSigDB repository (https://www.gsea-msigdb.org/gsea/msigdb). We also assessed metabolic pathway activities using metabolic pathways from a previously described curated dataset^46^. The gsva() function in GSVA R package (version 1.38.2) was implemented to estimate the pathway enrichment scores of individual cells. The differential activities of pathways between clusters or conditions were calculated using Limma R package (version 3.46.0). Gene sets with adj.p.val < 0.05 were considered significant.

### Functional analysis

To evaluate the activities of functional expression programs in immune cells, we calculated the gene signature expression scores using the AddModuleScore() function in Seurat. Signature gene lists for naïve, cytotoxicity and Treg have been previously described in ref. ^60^ and were provided in Supplementary Table 3. Oncogenic signaling pathway activity scores were calculated using the progeny R package^61^. Enrichment analysis for the functions of the DEGs was performed using the Metascape webtool (www.metascape.org).

### Plasma collection

For the plasma metabolomics cohort, 4 mL of peripheral blood was collected from all participants before surgery with tubes containing EDTA. All participants had fasted at least 8 h (h) before blood collection. Whole blood was centrifuged at 1600 g for 10 min and the obtained supernatant was centrifuged at 16,000 g for 10 min. Next, plasma aliquots were transferred into cryovials and stored at −80 °C.

### Sample preparation for metabolomics

For untargeted metabolomic, metabolites were extracted from plasma samples^62^ and tissue samples^63^ as previously described. Briefly, 25 mg of tissue samples were mixed with 500 μL of acetonitrile/methanol/water (2: 2: 1, V/V/V). After 30 s vortex, the tissue samples were homogenized at 35 Hz for 4 min and sonicated for 5 min in ice-water bath. For plasma samples, 100 μL of plasma mixed with 400 μL of acetonitrile/methanol (1:1, V/V). Then, tissue and plasma samples were incubated for 1 h at −40 °C to precipitate proteins, and centrifuged at 12,000 rpm for 15 min at 4 °C. The resulting supernatant was transferred to a fresh glass vial for analysis. For targeted metabolomics, plasma samples were thawed on the ice, followed by vortexed for 30 s. The mixture was centrifuged at 12,000 rpm for 1 h at 4 °C. The clear supernatant was transferred to an auto-sampler vial for subsequent analysis.

### LC-MS/MS metabolite profiling

For untargeted metabolomics, an ultra-high-performance liquid chromatography (UHPLC) system (Vanquish, Thermo Fisher Scientific) coupled with Q-Exactive MS (Thermo Scientific) was used for metabolites separation and detection. The mobile phase consisted of 25 mmol/L ammonium acetate and 25 ammonia hydroxide in water (pH = 9.75) (A) and acetonitrile (B). The analysis was carried with elution gradient as follows: 0 ~ 0.5 min, 95% B; 0.5 ~ 7.0 min, 95% ~ 65% B; 7.0 ~ 8.0 min, 65% ~ 40% B; 8.0 ~ 9.0 min, 40% B; 9.0 ~ 9.1 min, 40% ~ 95% B; 9.1 ~ 12.0 min, 95% B. The column temperature was maintained at 30 °C and the auto-sampler temperature was 4 °C. The injection volume was 3 μL. Next analysis was performed using the Q-Exactive MS (Thermo Scientific) equipped with an ESI ion source in information-dependent acquisition (IDA) mode (performed by Shanghai Biotree Biomedical Technology CO., LTD). The ESI source conditions were set as following: sheath gas flow rate as 50 Arb, aux gas flow rate as 10 Arb, capillary temperature 320 °C, full MS resolution as 60,000, MS/MS resolution as 7500, collision energy as 10/30/60 in NCE mode, spray Voltage as 3.5 kV (positive) or −3.2 kV (negative), respectively. The acquired raw data were converted to the mzXML format using ProteoWizard software (http://proteowizard.sourceforge.net). R package XCMS was used for peak detection, extraction, alignment, and integration^64^. Then an in-house MS2 database (BiotreeDB) was applied in metabolite annotation set cutoff annotation at 0.3^64^. For targeted metabolomics, the UHPLC separation was carried out using an ACQUITY UPLC-I/CLASS (Waters), equipped with an ACQUITY UPLC HSS T3 column (100 × 2.1 mm, 1.8 μm, Waters). The mobile phase A was 10 mmol/L ammonium formate and 0.02% formic acid in water and the mobile phase B was methanol. Waters Xevo TQ-S triple quadrupole mass spectrometer (Waters) was applied for assay development. Typical ion source parameters were: capillary voltages = 2.5 kV, cone voltages = 30 V, desolvation temperature = 550 °C, desolvation gas flow = 1100 (L/Hr), cone gas flow=150 (L/Hr), nebuliser gas flow =7.0 (Bar). The column temperature was set at 40 °C and the auto-sampler temperature was set at 10 °C. The injection volume was 10 μL. The quantification assay was performed on a Waters Xevo TQ-S triple quadrupole mass spectrometer (Waters) in multiple reaction monitoring (MRM) mode. One to four transitions were selected for each target metabolites, and collision energy were optimized for each transition to achieve maximum sensitivity. In total, 10 transitions (1-methylnicotinamide, 4 transitions; 1-methyl-2-pyridone-5-carboxamide, 4 transitions; Nicotinamide, 2 transitions) representing 3 target metabolites were acquired in a LC-MS run in positive-ion mode. Typical ion source parameters were: capillary voltages, 2.5 kV; cone voltages, 30 V; desolvation temperature, 550 °C; desolvation gas flow, 1100 (L/Hr); cone gas flow, 150 (L/Hr); and nebuliser gas flow, 7.0 (Bar). Chromatograms of each metabolite were extracted and quantified using the Agilent MassHunter Qualitative Analysis software (Agilent Technologies). Then acquired MRM data was processed by MultiQuant software (AB Sciex), areas of the XICs of targeted lipids were calculated and normalized with internal standards.

### Machine learning model in evaluating diagnostic power

Machine learning models with the abundance of the 3 targeted metabolites and clinical features as input were constructed with the caret package (https://cran.r-project.org/web/packages/caret/). Then, the diagnostic capacity of the targeted metabolites in the panel was evaluated with AUC and accuracy through the R packages pROC (https://cran.r-project.org/web/packages/pROC/index.html) in discriminating LN^+^ ESCC from LN^−^ ESCC. The model was trained by 60% of samples through fivefold cross-validation, and the rest samples were used as a testing dataset to assess the diagnostic capability.

### Cell culture

The Eca-109 and KYSE-30 cell lines were purchased from the Cell library of Shanghai Institutes for Biological Sciences, Chinese Academy of Sciences (Shanghai, China). Eca-109 and KYSE-30 were cultured in DMEM medium (Gibco, Carlsbad, CA, USA) supplemented with 10% FBS, 100 U/mL penicillin, and 100 U/mL streptomycin. The cells were cultured in an incubator (Thermo Scientific, Carlsbad, CA, USA) at a temperature of 37 °C and CO_2_ concentration of 5%.

### Transfection

The siRNAs were transfected into Eca-109 cell using Lipofectamine2000 (Invitrogen, San Francisco, CA, USA) according to the manufacturer’s instructions. Scrambled negative control siRNA (si‐NC) were purchased from Invitrogen (San Francisco, CA, USA). The sequences for siRNAs are listed in Supplementary Table 11. Typically, the cells were evenly added to 6-well culture plates at a certain concentration and transfected with siRNA on the next day.

### RNA extraction, reverse transcription, and the quantitative real-time PCR

TRIzol reagent was used to lyse Eca-109 cell and extract RNA. For qRT‐PCR, 1 μg of RNA from each sample was reverse transcribed to complementary DNA (cDNA) by using a reverse transcription kit (Takara, Beijing, China). qRT-PCR analyses were performed with TB Green (Takara, Beijing, China) according to the manufacturer’s instructions. The results were normalized with β-actin.

### Western blot assay

The cells were lysed using Radio-Immunoprecipitation Assay (RIPA) buffer (Beyotime Biotechnology, Shanghai, China) supplemented with protease inhibitors cocktail (Roche Applied Science, Indianapolis, IN, USA, dilution ratio, 1:100) and phenylmethanesulfonyl fluoride (PMSF) (Roche Applied Science, Indianapolis, IN, USA, dilution ratio, 1:1000). The protein extractions were separated by 10% SDS-PAGE transferred to 0.45 μm polyvinylidene difluoride (PVDF) membranes (Sigma Aldrich, St. Louis, MO, USA) and incubated overnight with specific primary antibody. The membrane was washed with wash buffer and probed with a secondary antibody (Beyotime Biotechnology, Shanghai, China). The autoradiograms were quantified by a developing instrument (Bio-Rad, Universal Hood II, CA, USA). The information of primary antibody is as follows: NNMT antibody (1:1000, ab119758, abcam, UK); E-cadherin antibody (1:1000, ab40772, abcam, UK); N-cadherin antibody (1:1000, ab76011, abcam, UK); β-catenin antibody (1:1000, ab32572, abcam, UK); METTL14 antibody (1:1000, ab309096, abcam, UK); IGF2BP1 antibody (1:1000, #8482, Cell Signaling Technology, USA). Results were normalized to the expression of β-actin. All blots originate from the same experiment and have undergone parallel processing. All uncropped blots were included in Supplementary Fig. 8.

### Cell migration assays

For the migration assays, after transfection, the cells in serum-free media were placed into the upper chamber of an insert (8-μm pore size; Millipore, Billerica, MA, USA). Medium containing 10% FBS was added to the lower chamber. After incubation for 24 h, the cells remaining on the upper membrane were removed with cotton wool. Cells that had migrated through the membrane were stained with methanol and 0.1% crystal violet, imaged, and counted using an IX71 inverted microscope (Olympus, Tokyo, Japan).

### Wound healing assays

Eca-109 cells were inoculated in six‐well culture medium until the confluence reached 90%. Before transfection, a sterile pipette tip was used to scratch a wound line across the surface of each dish. Then, the suspension cells were removed with phosphate belanced solution (PBS), and a new culture medium was added in a humidified 5% CO_2_ incubator at 37 °C. Images were taken with a microscope (Olympus, Tokyo, Japan) at 24 h intervals. Image Pro Plus 6.0 software (Media Cybernetics, Bethesda, MD, USA) was used to measure and calculate the distance that the cells had migrated.

### Tumor formation assay in mice model

Five-week-old male athymic BALB/c mice were purchased from GemPharmatech (Nanjing, China) and maintained under specific pathogen-free conditions. For the in vivo cell proliferation assay, Eca-109 cells were stably transfected and subcutaneously injected into either side of the posterior flanks of the mouse. The tumor volume was measured every few days (length × width^2^ × 0.5). At the end of the experiment, the mice were euthanized, and the tumors were removed, weighed, and then fixed for hematoxylin and eosin (H&E) and Ki-67 immunostaining analysis. To observe tumor metastasis in the lungs, treated Eca-109 cells were injected into the mice’s tail vein. After few days, the mice were euthanized, and the lungs were removed. Images of the lungs were taken for recording and used for H&E immunostaining analysis, and the nodules on the lung were counted. This study was conducted in strict accordance with the Guide for the Care and Use of Laboratory Animals of the National Institutes of Health. The study protocol was approved by the Committee on the Ethics of Animal Experiments of Nanjing Medical University.

### Transcriptome sequencing

Total RNA was isolated from NNMT knockdown and control Eca-109 cells. The concentration of each sample was measured using a NanoDrop 2000 (Thermo Scientific, Carlsbad, CA, USA). The quality was evaluated by an Agilent 2200 (Agilent, Palo Alto, CA, USA). TruSeq Stranded mRNA Library Prep Kit (Illumina, Inc.) was used to establish the sequencing library of each RNA sample. The sequencing data was presented in Supplementary Table 12.

### Gene set enrichment analysis (GSEA)

The differentially expressed genes (DEGs) responsive to NNMT knockdown were used in GSEA analyses against the Molecular Signatures Database (MSigDB) Hallmark gene sets and KEGG gene sets (http://software.broadinstitute.org/gsea/msigdb). GSEA was performed using GSEA software 4.0.3 by the Broad Institute (http://software.broadinstitute.org).

### Immunofluorescence microscopy

Cells grown on coverslip (24 mm × 24 mm) were fixed with 4% paraformaldehyde for 15 min, washed with 150 mM glycine in PBS, and permeabilized with 0.3% Triton X-100 in PBS for 10 min at room temperature. After blocking with 5% BSA, the cell smears were incubated with the indicated primary antibodies overnight at 4 °C, washed, and Alexa Flour® 488/647-labeled secondary antibody (life technologies) in PBS was added to the cell smears. Images were taken by a laser scanning confocal microscope (Nikon, Japan).

### ChIP assays

ChIP assays were performed using EZ-CHIPKIT according to the manufacturer’s instructions (Millipore, Billerica, MA, USA). The H3K4me3 antibody was obtained from Abcam (1:1000, ab8580, abcam, UK). Quantification of immunoprecipitated DNA was performed using qPCR with TB Green Mix. ChIP data were calculated as a percentage relative to the input DNA.

### Enzyme-Linked Immunosorbent Assay (ELISA)

To quantify cell SAM and SAH levels, direct ELISA was developed by immobilizing antigens to a solid plate, followed by the addition of SAM or SAH and HRP-labeled anti-SAM or anti-SAH antibodies. Antigens from a sample or standards competed with the corresponding fixed amount of immobilized antigens for binding to specific HRP-labeled antibodies. The final HRP substrate absorption values at 450 nM were inversely proportional to the amount of detected antigen.

### RNA m6A dot blot assays

Total RNA was first loaded onto a nitrocellulose membrane (Amersham, GE Healthcare, USA) installed in a BioDot apparatus (Bio-Rad, Hercules, CA, USA) with ice-cold 20×Saline-Sodium Citrate (SSC) buffer (Sigma Aldrich, St. Louis, MO, USA). First, the membrane was stained with 0.02% methylene blue (MB) (Beyotime Biotechnology, Shanghai, China) in 0.3 mol/L sodium acetate (pH 5.2) (Sigma Aldrich, St. Louis, MO, USA) to ensure the consistency of the baseline between the different groups. Then, the membrane was cross-linked using ultraviolet (UV), blocked, incubated with m6A antibody (1:2000, #202003, Synaptic Systems, Germany) overnight at 4 °C and subsequently incubated with Horseradish Peroxidase (HRP)-conjugated goat anti-mouse immunoglobin G (IgG) (Beyotime Biotechnology, Shanghai, China) for 1 h. Finally, the membrane was visualized by an imaging system (Bio-Rad, Universal Hood II, CA, USA).

### Methylated RNA immunoprecipitation (MeRIP) assay and qRT-PCR

Total RNA was isolated from stable NNMT knockdown Eca-109 cells and controls. Chemically fragmented RNA ( ~ 100 nt) was incubated with a m6A antibody for immunoprecipitation according to the standard protocol of the Magna MeRIP™ m6A Kit (Merck Millipore, Billerica, MA, USA). Briefly, total RNA was preheated at 94 °C for 10 min, Ethylene Diamine Tetraacetic Acid (EDTA) (Sigma Aldrich, St. Louis, MO, USA) was immediately added, 3 mol/L sodium acetate (Sigma Aldrich, St. Louis, MO, USA), glycogen (Sigma Aldrich, St. Louis, MO, USA) and 100% ethanol (Sigma Aldrich, St. Louis, MO, USA) were added, and the samples were incubated at −80 °C overnight. The next day, magnetic beads were prepared, and the MeRIP reaction mixture was prepared and incubated with m6A antibody at 4 °C for 2 h. The last m6A RNA was eluted with 10 mg of m6A 5’-monophosphate sodium salt (Merck Millipore, Billerica, MA, USA) at 4 °C for 1 h. Enrichment of m6A was analyzed using qRT-PCR.

### RNA stability assays

After siRNA transfection of Eca1-09 cells for 24 h, the cells were treated with 15 μg/mL actinomycin D (MedChemExpress, Newark, NJ, USA), then collected at 0, 4, and 8 h after treatment. Total RNA was extracted and detected by qRT-PCR.

### RNA immunoprecipitation (RIP) assays

The RIP experiment was performed using the Magna RIP™ RNA binding protein immunoprecipitation kit (CAT.17-701, Millipore, Billerica, MA, USA), and all operations were performed following the manufacturer’s instructions. The antibody against IGF2BP1 for the RIP assay was purchased from Cell Signaling Technology (7ug per sample, #8482, Cell Signaling Technology, USA).

### Statistical analysis

SPSS R26.0.0, Prism Graphpad v6.02, and R v4.0.2 software was used for statistical analyses. For data from metabolomic assays, PCA analysis was conducted with R using the prcomp function; Pathway enrichment, was performed using the MetaboAnalyst statistical analysis tool web service (https://www.metaboanalyst.ca). R package (pheatmap v1.0.12) and MetaboAnalyst statistical analysis tool web service was used for hierarchy cluster analysis. Statistical tools, methods and thresholds for each analysis of single-cell RNA-seq are explicitly described in the Results section or detailed in Figure Legends and the Methods sections.

### Reporting summary

Further information on research design is available in the Nature Research Reporting Summary linked to this article.

### Supplementary information

Supplementary Information

Reporting Summary

## Data Availability

The raw sequence data reported in this paper have been deposited in the Genome Sequence Archive of the Beijing Institute of Genomics (BIG) Data Center, BIG, Chinese Academy of Sciences, under accession code HRA003417 and are publicly accessible at http://bigd.big.ac.cn/gsa-human. Other supporting raw data are available from the corresponding author upon reasonable request.

## Code availability

The code that supports the findings of this study are available from the corresponding authors on reasonable request.
