# Supplementary material for: Multi-omics analysis reveals NNMT as a master metabolic regulator of metastasis in esophageal squamous cell carcinoma
Source: NPJ Precis Oncol. 2024 Jan 30;8:24. doi: 10.1038/s41698-024-00509-w (PMC10828394; doi:10.1038/s41698-024-00509-w)
Supplement: Supplementary file 1 — Supplementary Information [file 41698_2024_509_MOESM1_ESM.pdf]

Multi-omics analysis reveals NNMT as a master metabolic regulator of metastasis in esophageal squamous cell carcinoma

Correspondence

Mantang Qiu, Department of Thoracic Surgery, Thoracic Oncology Institute, Peking University People's Hospital, No. 11 Xizhimen South Street, Beijing 100044, China; Tel: 86-10-88325952; Fax: 86-10-68349763; E-mail: qiumantang@163.com

Supplementary Materials

Supplementary Figures

Supplementary Figure 1. Overview of major cell types and quality control of single cell sequencing data.

Supplementary Figure 2. Characterization of immune cell subtypes.

Supplementary Figure 3. Characterization of stromal cell subtypes.

Supplementary Figure 4. Malignant cells identified based on CNV-inferred method.

Supplementary Figure 5. Validation of scRNA-seq metabolism findings in external scRNA-seq dataset.

Supplementary Figure 6. NNMT promotes ESCC cell proliferation in *vitro* and in *vivo*.

Supplementary Figure 7. M6A modification in ESCC.

Supplementary Figure 8. Uncropped western blot gels presented in Fig.6-7 and Supplementary Fig. 6-7.

Supplementary Tables

Supplementary Table 1. Clinical characteristics of the 5 ESCC patients for scRNA-seq.

Supplementary Table 2. Canonical markers for 9 major cell types related to Fig. 1.

Supplementary Table 3. The public naïve, cytotoxic and Treg signatures related to Fig. 2f and Extended Data Fig. 2b.

Supplementary Table 4. The number of epithelial cells in each sample

Supplementary Table 5. Clinical characteristics of 44 patients in the external scRNA-seq cohort.

Supplementary Table 6. Differentially expressed genes in malignant cells between LN+ ESCC and LN- ESCC related to Fig. 4c

Supplementary Table 7. Differentially expressed genes in malignant cells between mLN and LN+ ESCC related to Extended Data Fig. 4c.

Supplementary Table 8. Clinical characteristics of untargeted metabolomics cohort.

Supplementary Table 9. Clinical characteristics of targeted metabolomics cohort.

Supplementary Table 10. Detecting performances of each model in test set related to Fig. 5f.

Supplementary Table 11: Primers and oligos sequences

Supplementary Table 12: Differentially expressed genes after NNMT knockdown

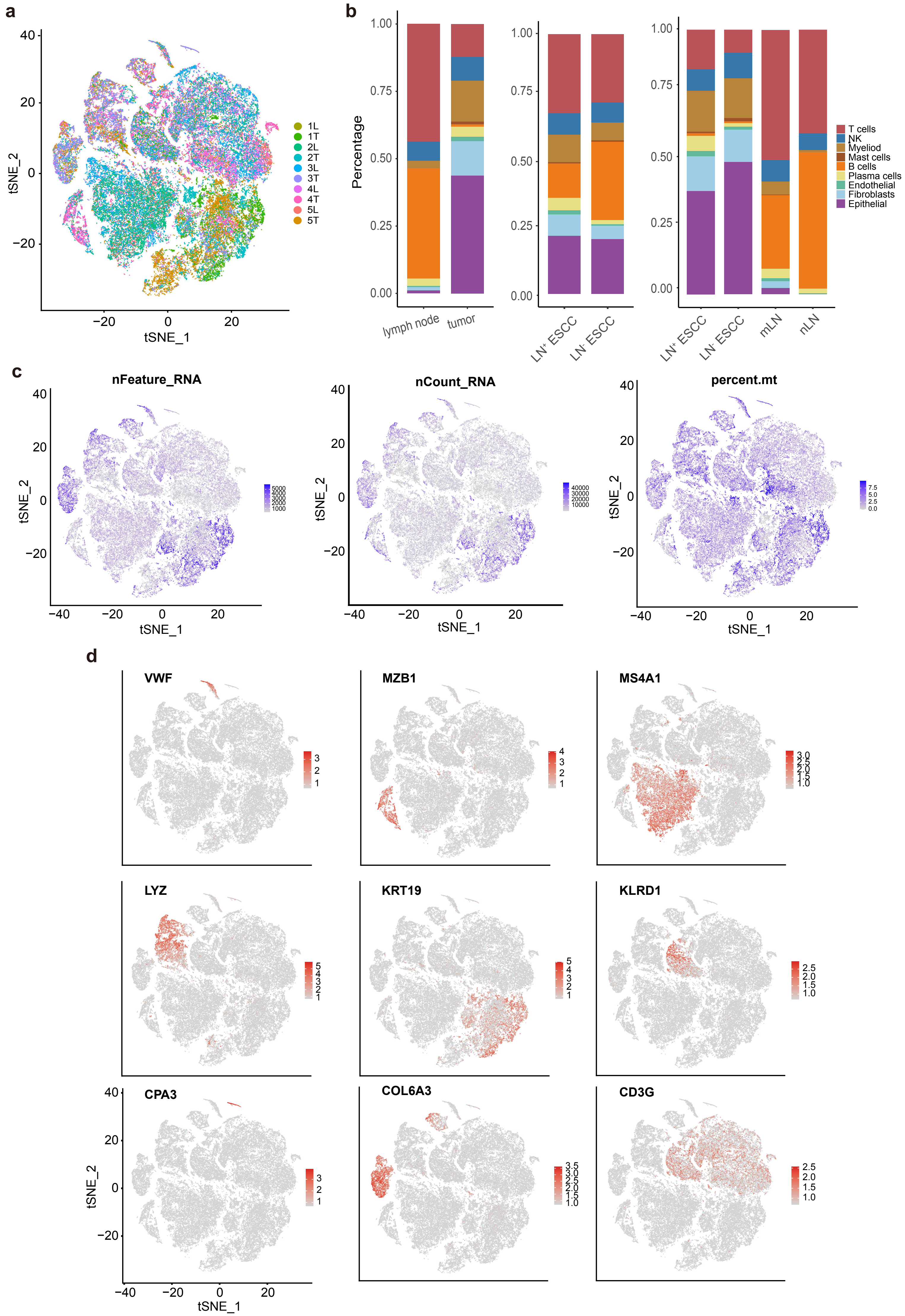

a
b
c
d
tSNE_1
tSNE_2
1L
1T
2L
2T
3L
3T
4L
4T
5L
5T
Percentage
lymph node
tumor
LN+ ESCC
LN- ESCC
mLN
nLN
T cells
NK
Myeliod
Mast cells
B cells
Plasma cells
Endothelial
Fibroblasts
Epithelial
nFeature_RNA
nCount_RNA
percent.mt
VWF
MZB1
MS4A1
LYZ
KRT19
KLRD1
CPA3
COL6A3
CD3G

Supplementary Figure 1. Overview of major cell types and quality control of single cell sequencing data.

a, TSNE plot of single cells colored by samples. **b,** The proportion of each cell type in lymph node samples and tumor tissues (left), LN^+ and LN^- groups (middle), and tissue types (right). **c,** TSNE plots of cells colored by number of genes detected, UMIs RNA, and percentage of mitochondrial genes. **d,** Expression of marker genes for major cell types. Nine major cell types were identified: endothelial cells (VWF+); fibroblasts (COL6A3+); plasma cells (MZB1+); B cells (MS4A1+); myeloid cells (LYZ+); mast cells (CPA3+); T cells (CD3G+); NK (KLRD1+); and epithelial cells (KRT19+).

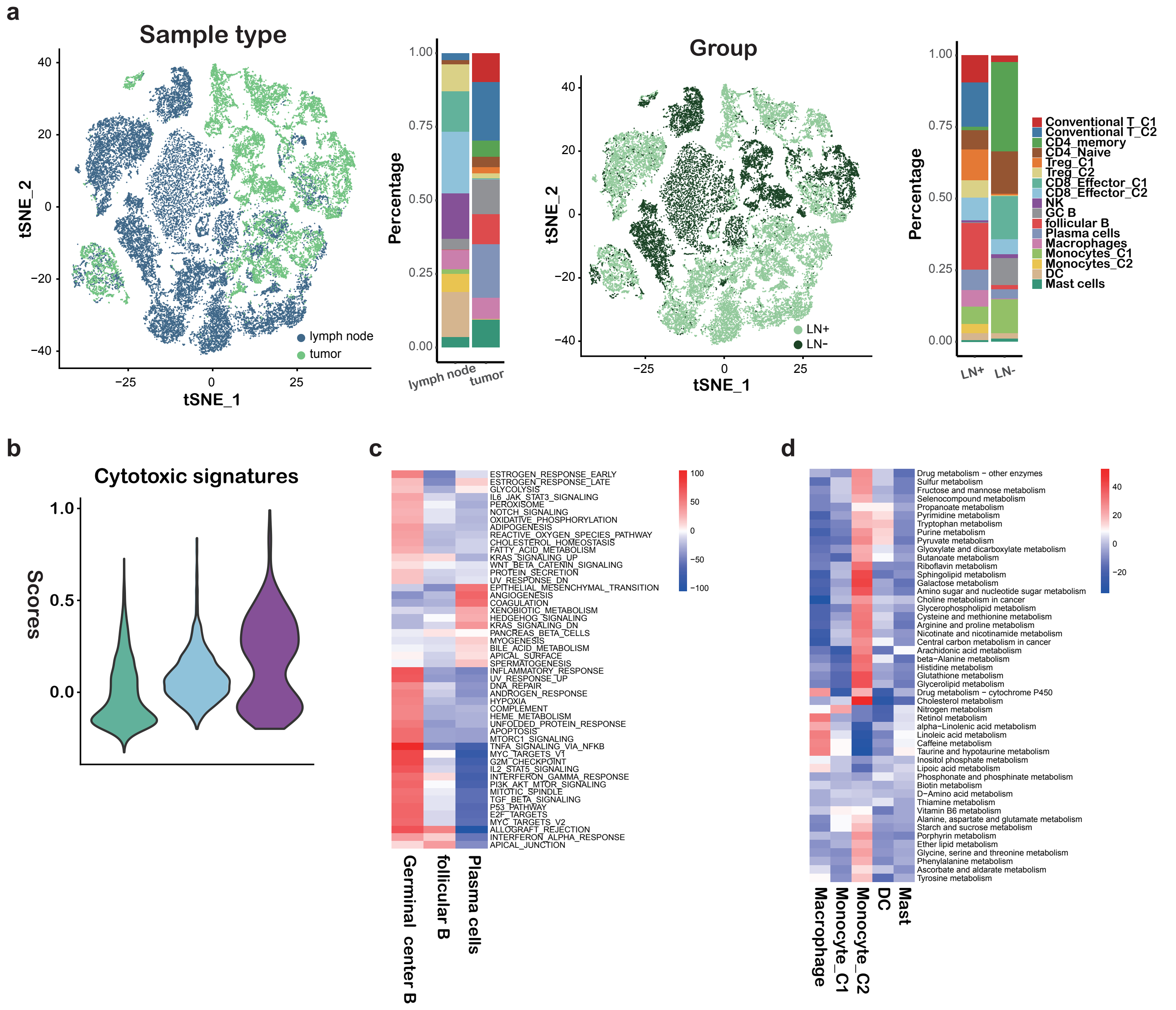

a
Sample type
tSNE_2
tSNE_1
lymph node
tumor
Percentage
lymph node
tumor
Group
LN+
LN-
Percentage
LN+
LN-
Conventional T_C1
Conventional T_C2
CD4_memory
CD4_Naive
Treg_C1
Treg_C2
CD8_Effector_C1
CD8_Effector_C2
NK
GC B
follicular B
Plasma cells
Macrophages
Monocytes_C1
Monocytes_C2
DC
Mast cells
b
Cytotoxic signatures
Scores
c
ESTROGEN_RESPONSE_EARLY
ESTROGEN_RESPONSE_LATE
GLYCOLYSIS
IL6_JAK_STAT3_SIGNALING
PEROXISOME
NOTCH_SIGNALING
OXIDATIVE_PHOSPHORYLATION
ADIPOGENESIS
REACTIVE_OXYGEN_SPECIES_PATHWAY
CHOLESTEROL_HOMEOSTASIS
FATTY_ACID_METABOLISM
KRAS_SIGNALING_UP
WNT_BETA_CATENIN_SIGNALING
PROTEIN_SECRETION
UV_RESPONSE_DN
EPITHELIAL_MESENCHYMAL_TRANSITION
ANGIOGENESIS
COAGULATION
XENOBIOTIC_METABOLISM
HEDGEHOG_SIGNALING
KRAS_SIGNALING_DN
PANCREAS_BETA_CELLS
MYOGENESIS
BILE_ACID_METABOLISM
APICAL_SURFACE
SPERMATOGENESIS
INFLAMMATORY_RESPONSE
UV_RESPONSE_UP
DNA_REPAIR
ANDROGEN_RESPONSE
HYPOXIA
COMPLEMENT
HEME_METABOLISM
UNFOLDED_PROTEIN_RESPONSE
APOPTOSIS
MTORC1_SIGNALING
TNFA_SIGNALING_VIA_NFKB
MYC_TARGETS_V1
G2M_CHECKPOINT
IL2_STAT5_SIGNALING
INTERFERON_GAMMA_RESPONSE
PI3K_AKT_MTOR_SIGNALING
MITOTIC_SPINDLE
TGF_BETA_SIGNALING
P53_PATHWAY
E2F_TARGETS
MYC_TARGETS_V2
ALLOGRAFT_REJECTION
INTERFERON_ALPHA_RESPONSE
APICAL_JUNCTION
Germinal center B
follicular B
Plasma cells
d
Drug metabolism − other enzymes
Sulfur metabolism
Fructose and mannose metabolism
Selenocompound metabolism
Propanoate metabolism
Pyrimidine metabolism
Tryptophan metabolism
Purine metabolism
Pyruvate metabolism
Glyoxylate and dicarboxylate metabolism
Butanoate metabolism
Riboflavin metabolism
Sphingolipid metabolism
Galactose metabolism
Amino sugar and nucleotide sugar metabolism
Choline metabolism in cancer
Glycerophospholipid metabolism
Cysteine and methionine metabolism
Arginine and proline metabolism
Nicotinate and nicotinamide metabolism
Central carbon metabolism in cancer
Arachidonic acid metabolism
beta−Alanine metabolism
Histidine metabolism
Glutathione metabolism
Glycerolipid metabolism
Drug metabolism − cytochrome P450
Cholesterol metabolism
Nitrogen metabolism
Retinol metabolism
alpha−Linolenic acid metabolism
Linoleic acid metabolism
Caffeine metabolism
Taurine and hypotaurine metabolism
Inositol phosphate metabolism
Lipoic acid metabolism
Phosphonate and phosphinate metabolism
Biotin metabolism
D−Amino acid metabolism
Thiamine metabolism
Vitamin B6 metabolism
Alanine, aspartate and glutamate metabolism
Starch and sucrose metabolism
Porphyrin metabolism
Ether lipid metabolism
Glycine, serine and threonine metabolism
Phenylalanine metabolism
Ascorbate and aldarate metabolism
Tyrosine metabolism
Macrophage
Monocyte_C1
Monocyte_C2
DC
Mast

Supplementary Figure 2. Characterization of immune cell subtypes.

a, Visualization and associated cell type proportions across immune cells of sample types (left) and lymph node metastatic groups (right). **b,** Violin plot of representative cytotoxic signatures in NK and CD8 T cell subtypes. **c,** Differentially expressed pathways are scored per cell by GSVA among three B cell subtypes. The relative activity scores were obtained from a linear model by limma. **d,** Heatmap showing differences in metabolic pathways scored per cell by GSVA among five myeloid cell subtypes.

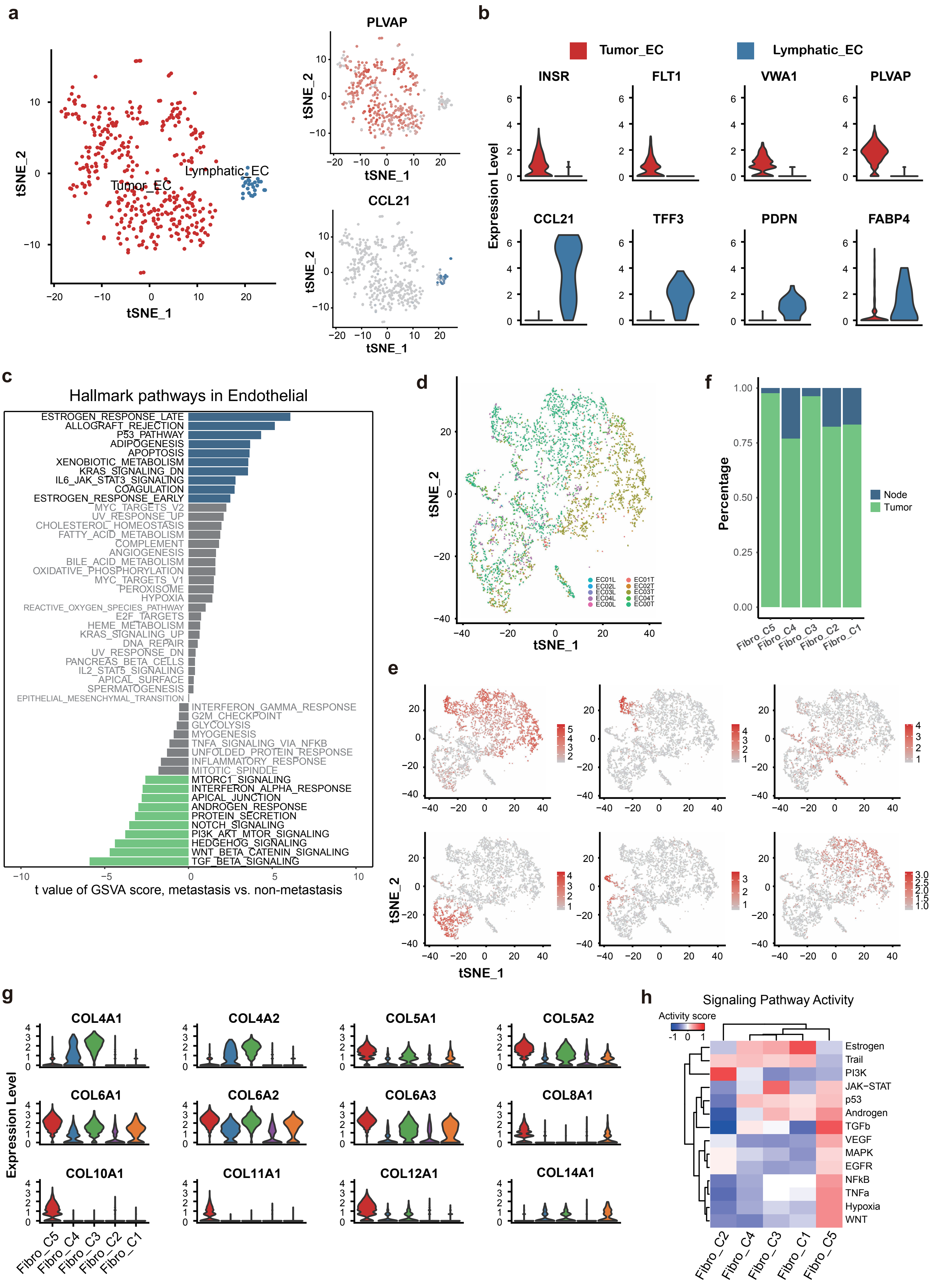
a
tSNE_2
tSNE_1
Tumor_EC
Lymphatic_EC
PLVAP
CCL21
b
Tumor_EC
Lymphatic_EC
Expression Level
INSR
FLT1
VWA1
PLVAP
CCL21
TFF3
PDPN
FABP4
c
Hallmark pathways in Endothelial
ESTROGEN_RESPONSE_LATE
ALLOGRAFT_REJECTION
P53_PATHWAY
ADIPOGENESIS
APOPTOSIS
XENOBIOTIC_METABOLISM
KRAS_SIGNALING_DN
IL6_JAK_STAT3_SIGNALING
COAGULATION
ESTROGEN_RESPONSE_EARLY
MYC_TARGETS_V2
UV_RESPONSE_UP
CHOLESTEROL_HOMEOSTASIS
FATTY_ACID_METABOLISM
COMPLEMENT
ANGIOGENESIS
BILE_ACID_METABOLISM
OXIDATIVE_PHOSPHORYLATION
MYC_TARGETS_V1
PEROXISOME
HYPOXIA
REACTIVE_OXYGEN_SPECIES_PATHWAY
E2F_TARGETS
HEME_METABOLISM
KRAS_SIGNALING_UP
DNA_REPAIR
UV_RESPONSE_DN
PANCREAS_BETA_CELLS
IL2_STAT5_SIGNALING
APICAL_SURFACE
SPERMATOGENESIS
EPITHELIAL_MESENCHYMAL_TRANSITION
INTERFERON_GAMMA_RESPONSE
G2M_CHECKPOINT
GLYCOLYSIS
MYOGENESIS
TNFA_SIGNALING_VIA_NFKB
UNFOLDED_PROTEIN_RESPONSE
INFLAMMATORY_RESPONSE
MITOTIC_SPINDLE
MTORC1_SIGNALING
INTERFERON_ALPHA_RESPONSE
APICAL_JUNCTION
ANDROGEN_RESPONSE
PROTEIN_SECRETION
NOTCH_SIGNALING
PI3K_AKT_MTOR_SIGNALING
HEDGEHOG_SIGNALING
WNT_BETA_CATENIN_SIGNALING
TGF_BETA_SIGNALING
t value of GSVA score, metastasis vs. non-metastasis
d
tSNE_2
tSNE_1
EC01L
EC02L
EC03L
EC04L
EC00L
EC01T
EC02T
EC03T
EC04T
EC00T
f
Percentage
Node
Tumor
Fibro_C5
Fibro_C4
Fibro_C3
Fibro_C2
Fibro_C1
e
tSNE_2
tSNE_1
g
Expression Level
COL4A1
COL4A2
COL5A1
COL5A2
COL6A1
COL6A2
COL6A3
COL8A1
COL10A1
COL11A1
COL12A1
COL14A1
Fibro_C5
Fibro_C4
Fibro_C3
Fibro_C2
Fibro_C1
h
Signaling Pathway Activity
Activity score
Estrogen
Trail
PI3K
JAK-STAT
p53
Androgen
TGFb
VEGF
MAPK
EGFR
NFkB
TNFa
Hypoxia
WNT
Fibro_C2
Fibro_C4
Fibro_C3
Fibro_C1
Fibro_C5

Supplementary Figure 3. Characterization of stromal cell subtypes.

a, TSNE plot of endothelial cells colored by two cell subtypes, and selected marker genes. **b,** Violin plots showing the expression of selected marker genes of different endothelial subtypes. **c,** Metabolic pathways scored per cell by GSVA between LN^{+} and LN^{-} group. **d,** TSNE plot of fibroblasts according to different samples. **e,** Marker genes were used to identify fibroblast subtypes. **f,** The proportion of fibroblasts in lymph node and tumor tissues. g, Mean pathway activity scores of different fibroblast cell subtypes. h, Violin plots showing the expression of collagens in different fibroblast subtypes.

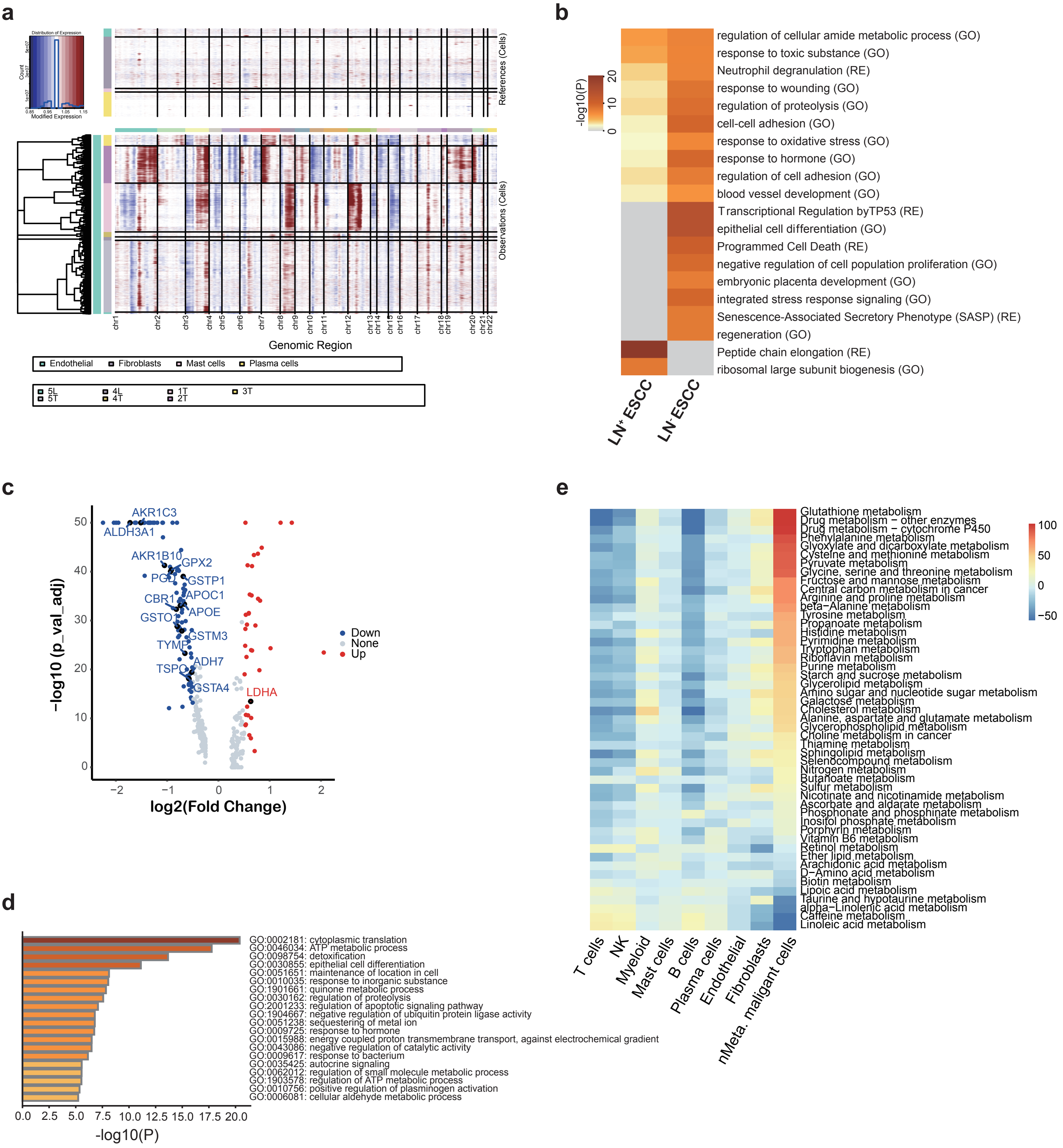
a
Distribution of Expression
Count
Modified Expression
References (Cells)
Observations (Cells)
chr1
chr2
chr3
chr4
chr5
chr6
chr7
chr8
chr9
chr10
chr11
chr12
chr13
chr14
chr15
chr16
chr17
chr18
chr19
chr20
chr21
chr22
Genomic Region
Endothelial
Fibroblasts
Mast cells
Plasma cells
5L
5T
4L
4T
1T
2T
3T
b
-log10(P)
regulation of cellular amide metabolic process (GO)
response to toxic substance (GO)
Neutrophil degranulation (RE)
response to wounding (GO)
regulation of proteolysis (GO)
cell-cell adhesion (GO)
response to oxidative stress (GO)
response to hormone (GO)
regulation of cell adhesion (GO)
blood vessel development (GO)
Transcriptional Regulation byTP53 (RE)
epithelial cell differentiation (GO)
Programmed Cell Death (RE)
negative regulation of cell population proliferation (GO)
embryonic placenta development (GO)
integrated stress response signaling (GO)
Senescence-Associated Secretory Phenotype (SASP) (RE)
regeneration (GO)
Peptide chain elongation (RE)
ribosomal large subunit biogenesis (GO)
LN+ ESCC
LN- ESCC
c
AKR1C3
ALDH3A1
AKR1B10
GPX2
PGD
GSTP1
CBR1
APOC1
GSTO
APOE
GSTM3
TYMP
ADH7
TSPO
GSTA4
LDHA
−log10 (p_val_adj)
log2(Fold Change)
Down
None
Up
d
GO:0002181: cytoplasmic translation
GO:0046034: ATP metabolic process
GO:0098754: detoxification
GO:0030855: epithelial cell differentiation
GO:0051651: maintenance of location in cell
GO:0010035: response to inorganic substance
GO:1901661: quinone metabolic process
GO:0030162: regulation of proteolysis
GO:2001233: regulation of apoptotic signaling pathway
GO:1904667: negative regulation of ubiquitin protein ligase activity
GO:0051238: sequestering of metal ion
GO:0009725: response to hormone
GO:0015988: energy coupled proton transmembrane transport, against electrochemical gradient
GO:0043086: negative regulation of catalytic activity
GO:0009617: response to bacterium
GO:0035425: autocrine signaling
GO:0062012: regulation of small molecule metabolic process
GO:1903578: regulation of ATP metabolic process
GO:0010756: positive regulation of plasminogen activation
GO:0006081: cellular aldehyde metabolic process
-log10(P)
e
Glutathione metabolism
Drug metabolism − other enzymes
Drug metabolism − cytochrome P450
Phenylalanine metabolism
Glyoxylate and dicarboxylate metabolism
Cysteine and methionine metabolism
Pyruvate metabolism
Glycine, serine and threonine metabolism
Fructose and mannose metabolism
Central carbon metabolism in cancer
Arginine and proline metabolism
beta−Alanine metabolism
Tyrosine metabolism
Propanoate metabolism
Histidine metabolism
Pyrimidine metabolism
Tryptophan metabolism
Riboflavin metabolism
Purine metabolism
Starch and sucrose metabolism
Glycerolipid metabolism
Amino sugar and nucleotide sugar metabolism
Galactose metabolism
Cholesterol metabolism
Alanine, aspartate and glutamate metabolism
Glycerophospholipid metabolism
Choline metabolism in cancer
Thiamine metabolism
Sphingolipid metabolism
Selenocompound metabolism
Nitrogen metabolism
Butanoate metabolism
Sulfur metabolism
Nicotinate and nicotinamide metabolism
Ascorbate and aldarate metabolism
Phosphonate and phosphinate metabolism
Inositol phosphate metabolism
Porphyrin metabolism
Vitamin B6 metabolism
Retinol metabolism
Ether lipid metabolism
Arachidonic acid metabolism
D−Amino acid metabolism
Biotin metabolism
Lipoic acid metabolism
Taurine and hypotaurine metabolism
alpha−Linolenic acid metabolism
Caffeine metabolism
Linoleic acid metabolism
T cells
NK
Myeloid
Mast cells
B cells
Plasma cells
Endothelial
Fibroblasts
nMeta. maligant cells

Supplementary Figure 4. Malignant cells identified based on CNV-inferred method.

a, Copy number variations (CNVs) evaluated per cell by InferCNV. Four cell types were used as control, including endothelial, fibroblasts, plasma cells and mast cells. **b,** Hierarchical clustering of the p values of the functional enrichment terms of the DEGs between LN^{+} ESCC and LN^{-} ESCC. The functional categories are from GO Biological Processes (GO), and Reactome (RE). For gene lists see Supplementary Table 6. **c,** The volcano plot shows DEGs between mLN and LN^{+} ESCC. The genes related to metabolism are labeled with their gene names. The red and blue points indicate up and down- regulated DEGs in malignant cells, respectively. **d,** Gene enrichment analyses of the DEGs between mLN and LN^{+} ESCC. GO terms are labeled with names and ids and are sorted by -log10 (P) value. A darker color indicates a smaller p-value. The top 20 enriched GO terms are shown. **e,** Metabolic pathway expression profiles in LN^{-} ESCC. For each pathway, the fold change in malignant cells was calculated by comparing to other cell types and corrected for sample of origin. Pathways were ordered by log-fold change.

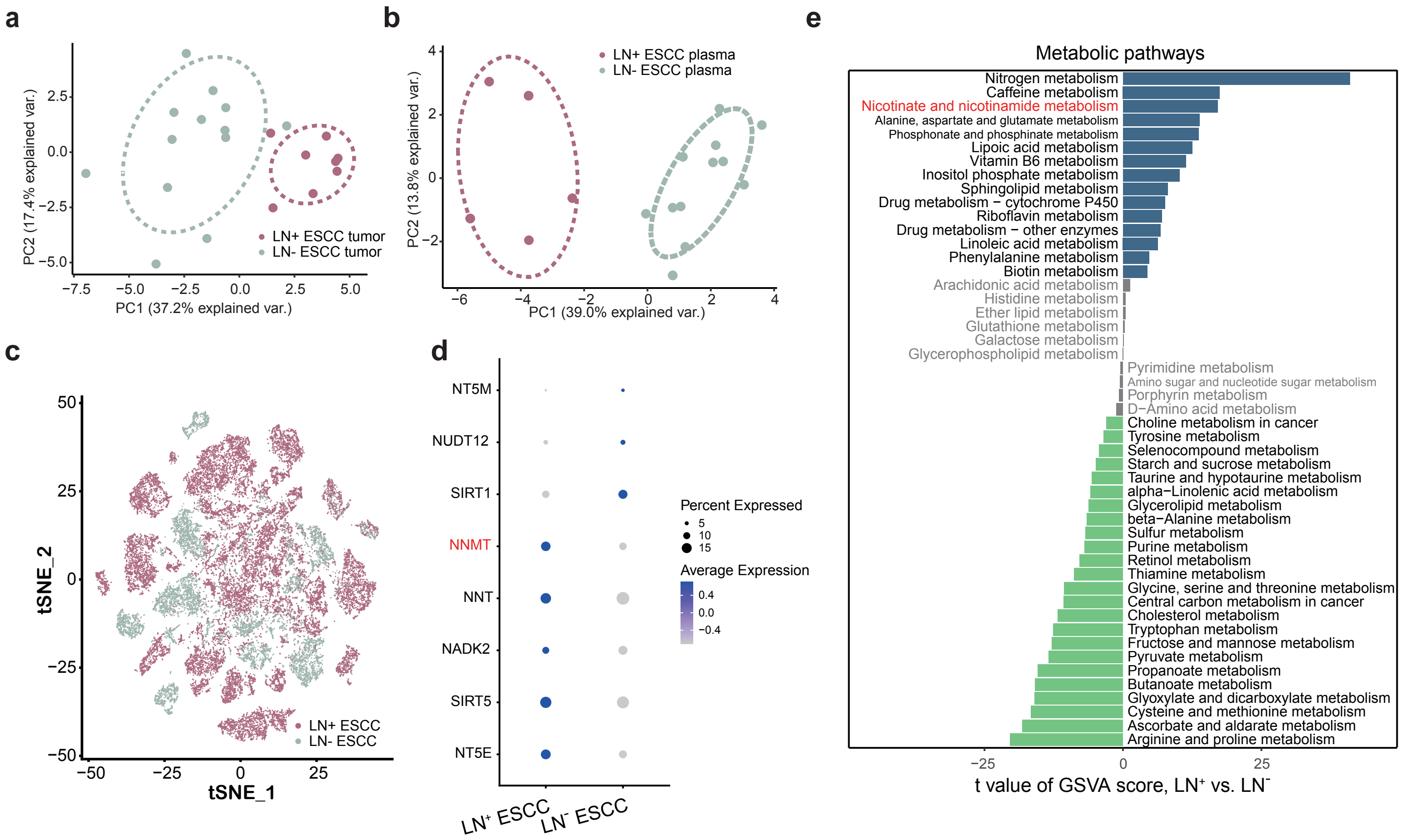

a
PC2 (17.4% explained var.)
PC1 (37.2% explained var.)
LN+ ESCC tumor
LN- ESCC tumor
b
PC2 (13.8% explained var.)
PC1 (39.0% explained var.)
LN+ ESCC plasma
LN- ESCC plasma
c
tSNE_2
tSNE_1
LN+ ESCC
LN- ESCC
d
NT5M
NUDT12
SIRT1
NNMT
NNT
NADK2
SIRT5
NT5E
Percent Expressed
Average Expression
LN+ ESCC
LN- ESCC
e
Metabolic pathways
Nitrogen metabolism
Caffeine metabolism
Nicotinate and nicotinamide metabolism
Alanine, aspartate and glutamate metabolism
Phosphonate and phosphinate metabolism
Lipoic acid metabolism
Vitamin B6 metabolism
Inositol phosphate metabolism
Sphingolipid metabolism
Drug metabolism − cytochrome P450
Riboflavin metabolism
Drug metabolism − other enzymes
Linoleic acid metabolism
Phenylalanine metabolism
Biotin metabolism
Arachidonic acid metabolism
Histidine metabolism
Ether lipid metabolism
Glutathione metabolism
Galactose metabolism
Glycerophospholipid metabolism
Pyrimidine metabolism
Amino sugar and nucleotide sugar metabolism
Porphyrin metabolism
D−Amino acid metabolism
Choline metabolism in cancer
Tyrosine metabolism
Selenocompound metabolism
Starch and sucrose metabolism
Taurine and hypotaurine metabolism
alpha−Linolenic acid metabolism
Glycerolipid metabolism
beta−Alanine metabolism
Sulfur metabolism
Purine metabolism
Retinol metabolism
Thiamine metabolism
Glycine, serine and threonine metabolism
Central carbon metabolism in cancer
Cholesterol metabolism
Tryptophan metabolism
Fructose and mannose metabolism
Pyruvate metabolism
Propanoate metabolism
Butanoate metabolism
Glyoxylate and dicarboxylate metabolism
Cysteine and methionine metabolism
Ascorbate and aldarate metabolism
Arginine and proline metabolism
t value of GSVA score, LN+ vs. LN-

Supplementary Figure 5. Validation of scRNA-seq metabolism findings in external scRNA-seq dataset.

(a-b) Principal component analysis showed stratification of tissue samples (a) and plasma samples (b) based on untargeted metabolomics. PC1 and PC2 values represent the top two principal coordinates. Different sample types were denoted by color code. **c,** The t-SNE plot of tumor cells in external scRNA dataset, grouped by LN^+ ESCC and LN^- ESCC groups. **d,** The dot plot showing selected genes of nicotinate and nicotinamide metabolism in tumor cells between LN^+ ESCC and LN^- ESCC groups. **e,** Differences in metabolic pathway activities scored per cell by GSVA between LN^+ ESCC and LN^- ESCC groups.

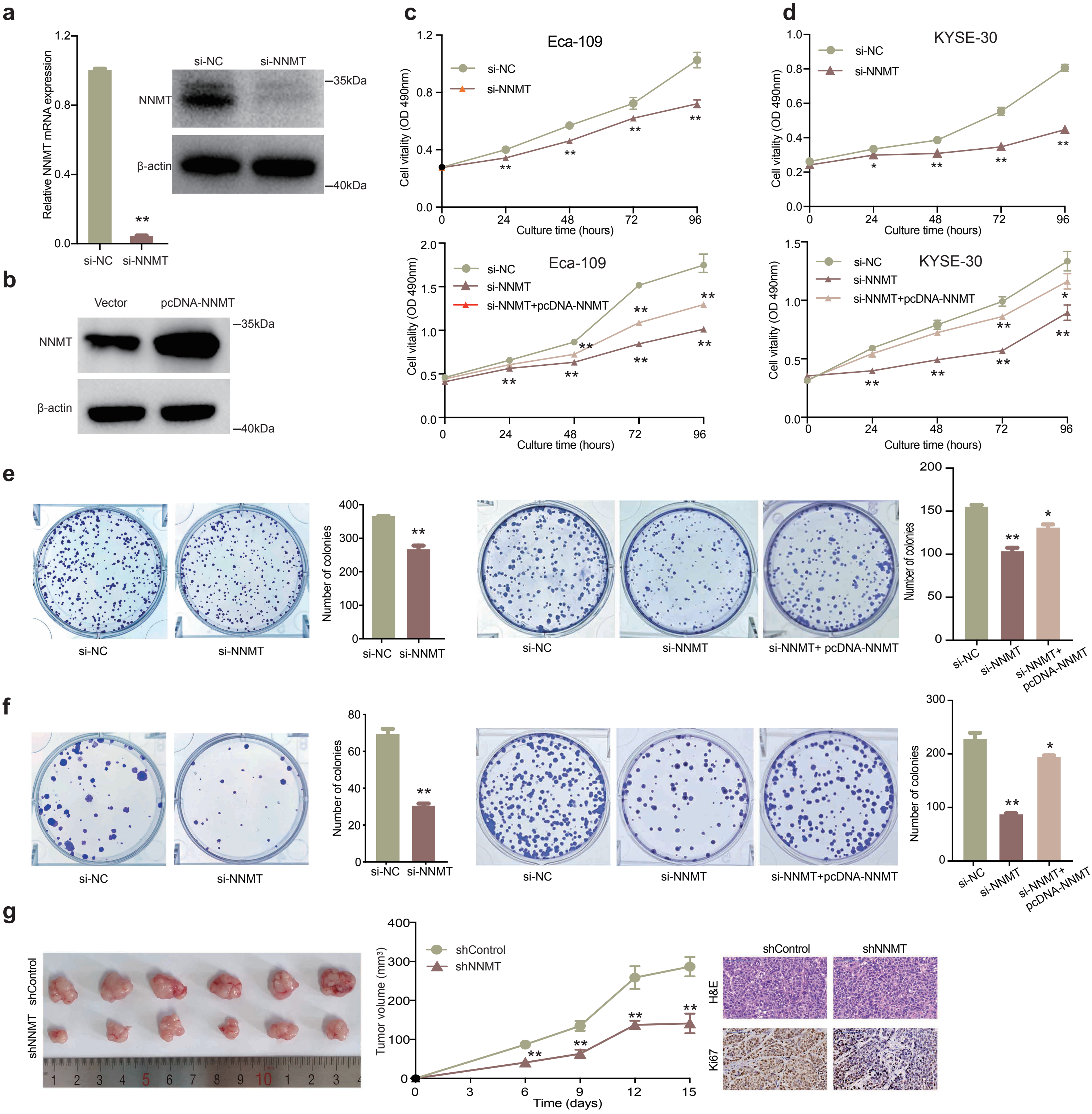

a
Relative NNMT mRNA expression
si-NC
si-NNMT
NNMT
β-actin
35kDa
40kDa
b
Vector
pcDNA-NNMT
c
Eca-109
Cell vitality (OD 490nm)
Culture time (hours)
si-NNMT+pcDNA-NNMT
d
KYSE-30
e
Number of colonies
si-NNMT+ pcDNA-NNMT
f
g
shControl
shNNMT
Tumor volume (mm3)
Time (days)
H&E
Ki67

Supplementary Figure 6. NNMT promotes ESCC cell proliferation in *vitro* and in *vivo*.

a, NNMT expression was analyzed by western blotting after transfection of si-NNMT in Eca-109 cell. **b,** NNMT expression was analyzed by western blotting after overexpression of NNMT in Eca-109 cell. (**c-d**) MTT assays were used to analyze the cell viability after transfection in Eca-109 cells and KYSE-30 cells. (**e-f**) After corresponding transfection, colony formation assays were used to investigate the colony ability of Eca-109 and KYSE-30 cells. (**g**) Eca-109 cells transfected with shControl and shNNMT were injected into nude mice. Tumor volumes were measured at fixed intervals. H&E staining and IHC staining (Ki-67) were performed to detect tumor sections.

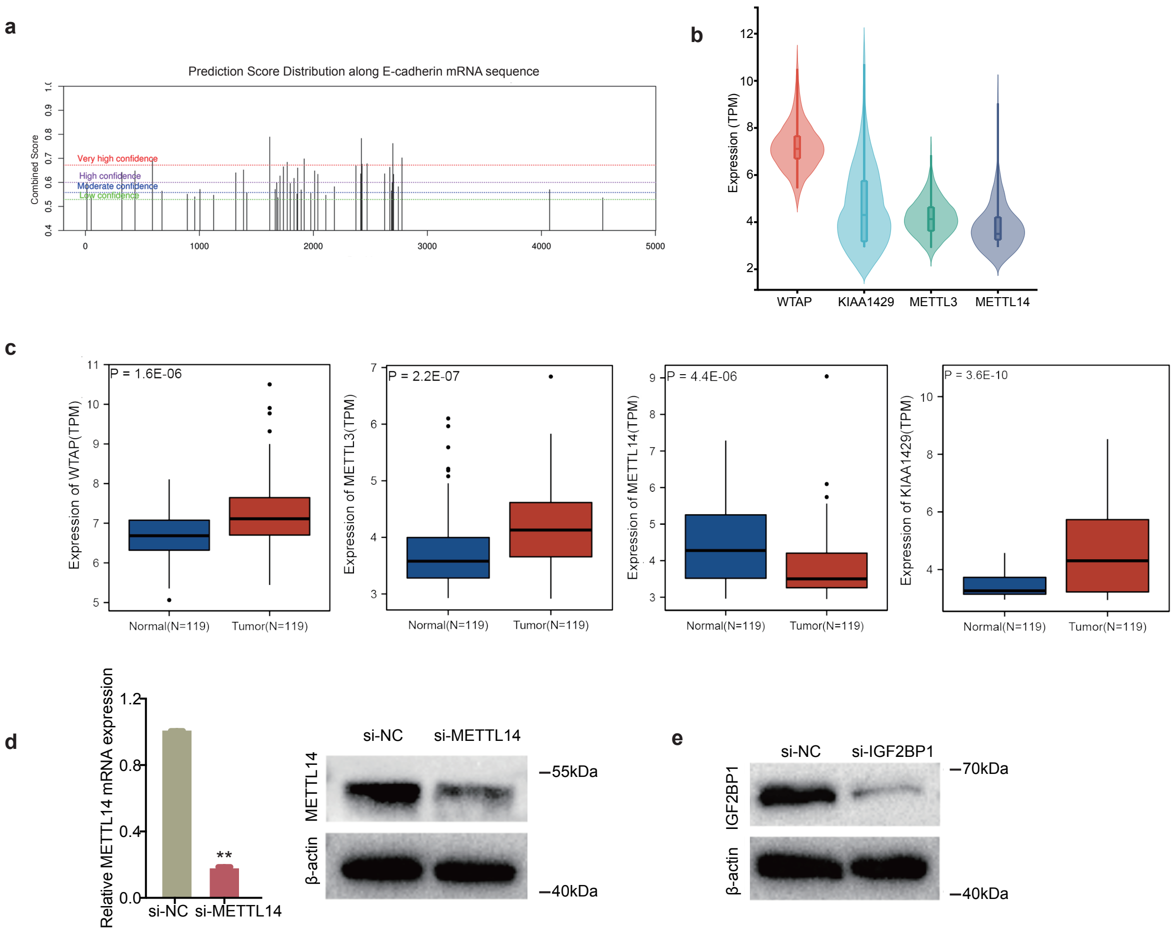
a
Prediction Score Distribution along E-cadherin mRNA sequence
Combined Score
Very high confidence
High confidence
Moderate confidence
Low confidence
b
Expression (TPM)
WTAP
KIAA1429
METTL3
METTL14
c
P = 1.6E-06
Expression of WTAP(TPM)
P = 2.2E-07
Expression of METTL3(TPM)
P = 4.4E-06
Expression of METTL14(TPM)
P = 3.6E-10
Expression of KIAA1429(TPM)
Normal(N=119)
Tumor(N=119)
d
Relative METTL14 mRNA expression
si-NC
si-METTL14
METTL14
β-actin
55kDa
40kDa
e
si-NC
si-IGF2BP1
IGF2BP1
β-actin
70kDa
40kDa

Supplementary Figure 7. M6A modification in ESCC.

a, M6A modification sites in the E-cadherin mRNA sequences were predicted by SRAMP. The combined scores were divided into four degrees (very high confidence, high confidence, moderate confidence, low confidence). (**b-c**) the expression of 4 "writers" in ESCC. **d,** qRT-PCR assays and western blot assays detected the mRNA and protein levels of METTL14 after si-METTL14 treatment in Eca-109 cells. **e,** Western blot assays detected the protein level of IGF2BP1 after si-IGF2BP1 treatment in Eca-109 cells.

Figure 6b

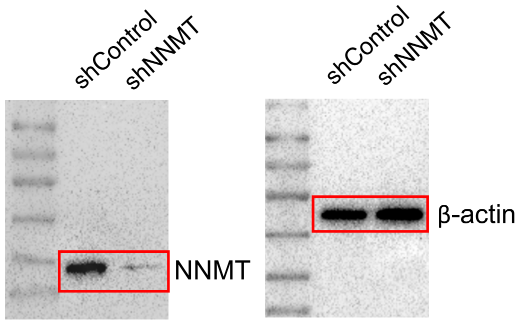

Figure 6c

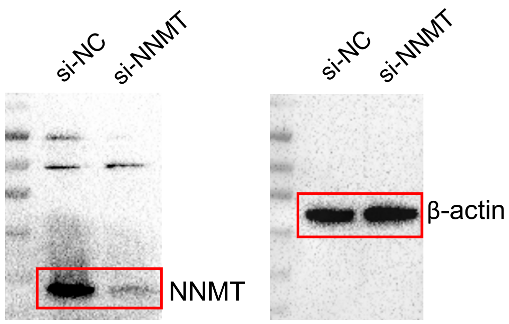

Figure 6h

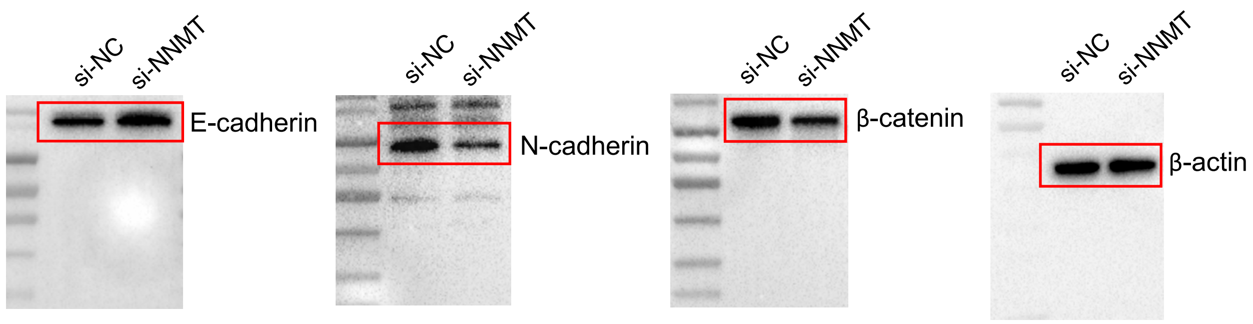

Figure 7e

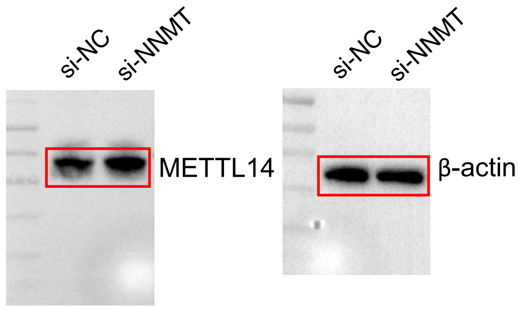

Figure 7h

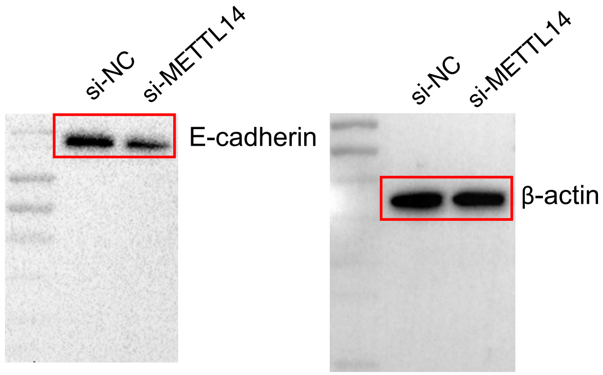

Figure S6a

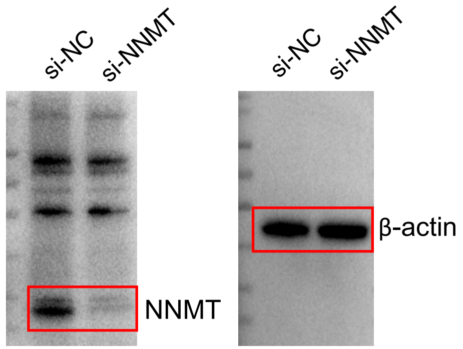

Figure S6b

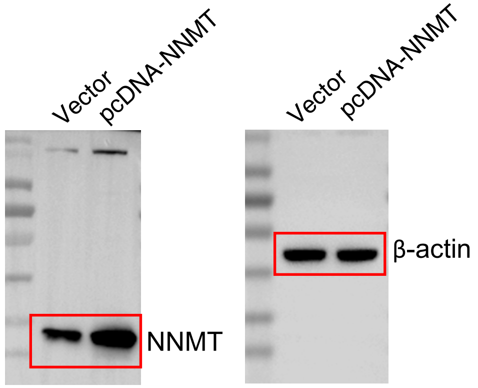

Figure S7d

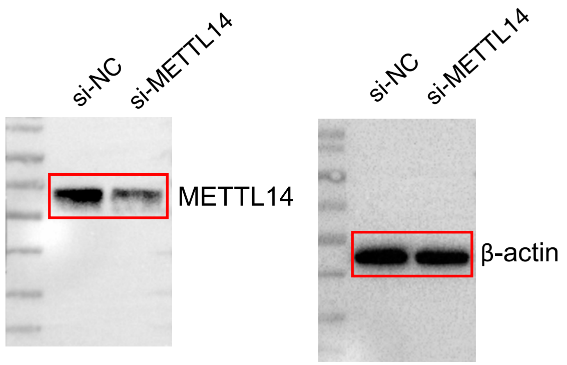

Figure S7e

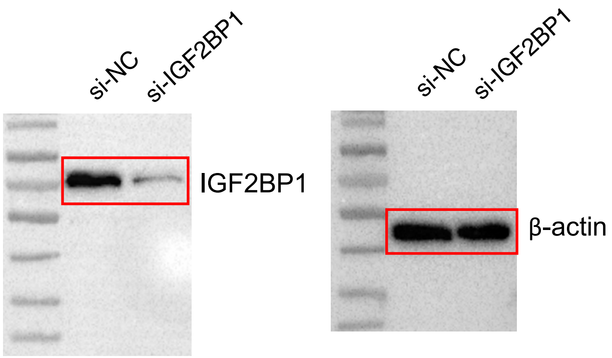

Supplementary Figure 8. Uncropped western blot gels presented in Fig.6-7 and Supplementary Fig. 6-7.

Supplementary Table 1: Clinical characteristics of the 5 ESCC patients for scRNA-seq

Patient ID	Gender	Age	Smoking status	Drinking status	TNM	Stage
1	Male	72	Non-smoker	Non-drinker	T3N0M0	‖ A
2	Female	78	Non-smoker	Non-drinker	T2N0M0	‖ A
3	Female	53	Non-smoker	Non-drinker	T3N0M0	‖ A
4	Male	64	Non-smoker	Non-drinker	T3N2M0	|||B
5	Male	64	Non-smoker	Non-drinker	T4aN1M0	|||B

Supplementary Table 2: Canonical markers for 9 major cell types related to Fig 1

Cell type	Marker genes
T cells	CD3D, CD3E, CD3G, CD2
NK	KLRD1, KLRB1, NKG7, GNLY
Myeloid	LYZ, AIF1, CD68
Mast cells	TPSAB1, TPSB2, KIT, CPA3
B cells	MS4A1, CD79A, CD79B, BANK1
Plasma cells	MZB1, JCHAIN, IGHG2
Endothelial	RAMP2, VWF, CLDN5
Fibroblasts	DCN, ACTA2, COL1A1, COL6A3
Epithelial	EPCAM, KRT19, KRT14, KRT17

Supplementary Table 3: The public naïve, cytotoxic and Treg signatures related to Fig. 2f and Extended Data Fig. 2b

Naïve signature	Cytotoxic signature	Treg signautre
CCR7	PRF1	NT5E
TCF7	IFNG	CD3D
LEF1	GNLY	CD3G
SELL	NKG7	CD3E
	GZMB	CD4
	GZMA	CD5
	HZMH	ENTPD1
	KLRK1	CTLA4
	KLRKB1	IZUMO1R
	KLRD1	TNFRSF18
	CTSW	IL2RA
	CST7	ITGAE
	CX3CR1	LAG3
	FGFBP2	TGFB1
	S1PR5	LRRC32
	FCGR3A	TNFRSF4
	PLAC8	SELL
	C1orf21	FOXP3
	TGFBR3	STAT5A
	PLEK	STAT5B
	FGR	LGALS1
	KLRF1	IL10
	SPON2	IL12A
	CD300A	EBI3
	S1PR1	TGFB1
	FAM65B	
	EFHD2	
	STK38	
	C1orf162	
	SORL1	
	EMP3	
	ARL4C	
	BIN2	
	CCND3	
	FCRL6	
	SAMD3	
	TRDC	
	TYROBP	

	GNLY	
	KLRG1	

Supplementary Table 4: The number of epithelial cells in each sample

**sample**	**cell numbers**
EC04T	345
EC05T	4944
EC04L	237
EC05L	114
EC01T	3360
EC02T	2571
EC03T	744
EC01L	5
EC02L	2
EC03L	5

Supplementary Table 5: Clinical characteristics of 44 patients in the external scRNA-seq cohort

Sample ID	Smoking status	Drinking status	Pathologic stage	T stage	N stage	M stage
P10	Smoker	Non-drinker	II	3	0	0
P104	Smoker	Drinker	III	3	2	0
P11	Non-smoker	Non-drinker	II	3	0	0
P12	Smoker	Non-drinker	II	3	0	0
P126	Smoker	Drinker	III	3	1	0
P127	Smoker	Non-drinker	III	3	2	0
P128	Non-smoker	Non-drinker	II	2	1	0
P130	Smoker	Non-drinker	II	3	0	0
P15	Non-smoker	Non-drinker	II	3	0	0
P19	Non-smoker	Non-drinker	II	3	0	0
P20	Smoker	Drinker	III	3	2	0
P21	Non-smoker	Non-drinker	III	3	2	0
P22	Smoker	Drinker	II	3	0	0
P23	Smoker	Drinker	III	3	3	0
P24	Smoker	Non-drinker	III	3	1	0
P26	Non-smoker	Non-drinker	III	3	2	0
P27	Smoker	Drinker	II	3	0	0
P28	Smoker	Drinker	III	3	1	0
P32	Smoker	Non-drinker	II	3	0	0
P36	Non-smoker	Non-drinker	II	3	0	0
P37	Non-smoker	Non-drinker	III	3	1	0
P38	Smoker	Non-drinker	II	3	0	0
P39	Smoker	Non-drinker	II	2	1	0
P40	Smoker	Non-drinker	III	2	2	0
P42	Smoker	Drinker	II	1	1	0
P44	Smoker	Non-drinker	III	3	2	0
P47	Smoker	Drinker	III	3	2	0
P5	Non-smoker	Non-drinker	III	3	1	0
P54	Non-smoker	Non-drinker	II	3	0	0
P57	Smoker	Drinker	III	3	1	0
P61	Non-smoker	Non-drinker	II	3	0	0
P62	Non-smoker	Non-drinker	III	3	1	0
P63	Non-smoker	Non-drinker	III	4	3	0
P74	Smoker	Non-drinker	II	3	0	0
P75	Smoker	Drinker	III	3	2	0
P8	Non-smoker	Non-drinker	III	4	1	0
P80	Non-smoker	Non-drinker	III	3	2	0

P83	Smoker	Non-drinker	III	3	3	0
P84	Non-smoker	Non-drinker	III	3	1	0
P87	Smoker	Drinker	III	3	2	0
P89	Smoker	Drinker	III	3	1	0
P9	Smoker	Drinker	III	3	1	0
P91	Smoker	Drinker	II	3	0	0
P94	Non-smoker	Non-drinker	III	3	1	0

Supplementary Table 6: Differential genes expression between LN+ ESCC and LN- ESCC related to Fig. 4c

	p_val	avg_log2 FC	pct.1	pct.2	p_val_adj	Sig
RPL11	0	0.8895	1	0.995	0	Up
RPS8	0	0.7371	1	0.998	0	Up
GSTM3	0	0.905	0.639	0.321	0	Up
S100A9	0	0.829	0.857	0.371	0	Up
S100A8	0	1.1805	0.833	0.32	0	Up
IGKC	0	1.3939	0.679	0.182	0	Up
DAPL1	0	1.787	0.613	0.158	0	Up
COL3A1	0	0.5812	0.332	0.036	0	Up
RPL24	0	0.8255	1	0.993	0	Up
RBP1	0	1.0003	0.569	0.235	0	Up
RPL9	0	0.9728	1	0.988	0	Up
RPL34	0	0.7737	1	0.996	0	Up
CD74	0	0.8261	0.644	0.156	0	Up
RPS14	0	0.5403	1	0.997	0	Up
SPARC	0	0.5059	0.277	0.035	0	Up
RACK1	0	0.8095	0.998	0.926	0	Up
HLA-DRA	0	0.7313	0.569	0.147	0	Up
HLA-DRB1	0	0.5814	0.353	0.06	0	Up
RPS18	0	0.6512	1	1	0	Up
RPL10A	0	0.5913	1	0.994	0	Up
COL1A2	0	0.5814	0.327	0.038	0	Up
TIMP1	0	0.6329	0.368	0.072	0	Up
FNTA	0	0.6817	0.468	0.156	0	Up
FABP5	0	1.0236	0.977	0.919	0	Up
LY6D	0	1.5858	0.82	0.36	0	Up
RPS6	0	0.8551	1	0.997	0	Up
RPL12	0	0.6522	1	0.998	0	Up
RPS13	0	0.676	0.999	0.972	0	Up
VIM	0	0.6939	0.398	0.082	0	Up
MGP	0	0.6222	0.308	0.003	0	Up
PFDN5	0	0.6532	0.933	0.759	0	Up
RPL6	0	0.5375	1	0.992	0	Up
RPLP0	0	0.62	1	0.991	0	Up
RPL21	0	1.1734	1	0.997	0	Up
TPT1	0	1.083	1	0.993	0	Up
RPL4	0	0.9426	0.988	0.847	0	Up

RPLP1	0	0.7168	1	1	0	Up
RPS17	0	0.7256	0.999	0.986	0	Up
MT1E	0	1.1935	0.522	0.06	0	Up
RPL26	0	0.6663	1	0.99	0	Up
KRT15	0	1.2463	0.737	0.46	0	Up
COL1A1	0	0.6916	0.392	0.037	0	Up
ACTG1	0	0.6906	0.992	0.959	0	Up
SERPINB3	0	1.3936	0.406	0.05	0	Up
ZFAS1	0	0.668	0.906	0.831	0	Up
CIRBP	0	0.7056	0.607	0.265	0	Up
APOE	0	1.1494	0.577	0.035	0	Up
APOC1	0	0.8786	0.437	0.026	0	Up
RPL18	0	0.6997	1	0.998	0	Up
LGALS1	0	0.6901	0.67	0.279	0	Up
RPS2	1.17E-303	0.504	1	0.999	2.66E-299	Up
MIB2	8.41E-302	0.5727	0.403	0.125	1.91E-297	Up
VDAC3	3.52E-286	0.7155	0.55	0.29	7.99E-282	Up
GPX4	5.32E-283	0.6951	0.735	0.495	1.21E-278	Up
SNHG7	2.15E-280	0.6272	0.397	0.135	4.89E-276	Up
GSTO1	7.56E-280	0.8615	0.619	0.422	1.72E-275	Up
SMIM19	2.44E-275	0.563	0.413	0.146	5.53E-271	Up
RPL29	1.94E-257	0.5054	0.999	0.986	4.40E-253	Up
MT-ND3	8.88E-230	0.7215	0.969	0.838	2.02E-225	Up
EEF2	2.99E-224	0.5503	0.941	0.888	6.78E-220	Up
SH3BGRL3	4.54E-216	0.5151	0.804	0.579	1.03E-211	Up
RPL17	3.34E-163	0.6123	0.888	0.721	7.59E-159	Up
UPK1B	2.30E-152	0.5317	0.421	0.203	5.23E-148	Up
LGALS7B	9.60E-151	0.6077	0.381	0.19	2.18E-146	Up
PKM	1.21E-149	0.5228	0.764	0.748	2.75E-145	Up
ALDH3A1	1.40E-149	0.7638	0.742	0.649	3.17E-145	Up
TACSTD2	0	-1.1106	0.225	0.59	0	Down
S100A10	0	-0.6027	0.912	0.982	0	Down
S100A11	0	-0.7045	0.986	0.998	0	Down
S100A6	0	-2.312	0.958	1	0	Down
EFNA1	0	-0.5839	0.162	0.503	0	Down
LINC01133	0	-0.7842	0.053	0.382	0	Down
F11R	0	-0.5404	0.117	0.495	0	Down
TMCO1	0	-0.5578	0.166	0.517	0	Down
CREG1	0	-0.7518	0.11	0.529	0	Down

MPC2	0	-0.9731	0.104	0.636	0	Down
ATP1B1	0	-1.3287	0.163	0.723	0	Down
PRDX6	0	-0.8052	0.311	0.787	0	Down
GLUL	0	-0.8361	0.178	0.63	0	Down
ELF3	0	-1.3787	0.089	0.557	0	Down
BTG2	0	-0.8066	0.104	0.483	0	Down
RPS7	0	-0.6083	0.996	0.998	0	Down
YWHAQ	0	-0.8002	0.282	0.735	0	Down
LAPTM4A	0	-0.5389	0.229	0.609	0	Down
SDC1	0	-0.7114	0.299	0.718	0	Down
EPCAM	0	-0.8197	0.271	0.668	0	Down
RND3	0	-0.8092	0.057	0.359	0	Down
IGFBP2	0	-1.4966	0.139	0.57	0	Down
PTMA	0	-0.8022	0.999	0.999	0	Down
CD47	0	-0.5227	0.072	0.37	0	Down
H1FX	0	-0.916	0.088	0.464	0	Down
RNF7	0	-0.575	0.421	0.806	0	Down
ATP1B3	0	-0.9059	0.568	0.91	0	Down
RAP2B	0	-0.7272	0.035	0.396	0	Down
SOX2	0	-0.7918	0.137	0.475	0	Down
MAP3K13	0	-0.6357	0.253	0.608	0	Down
CLDN1	0	-0.6876	0.08	0.396	0	Down
HES1	0	-1.3515	0.166	0.526	0	Down
FGFBP1	0	-1.1455	0.055	0.491	0	Down
UCHL1	0	-1.0473	0.014	0.544	0	Down
DSP	0	-0.6634	0.128	0.495	0	Down
DEK	0	-0.6867	0.151	0.503	0	Down
SGK1	0	-0.6147	0.115	0.46	0	Down
PERP	0	-1.7169	0.664	0.97	0	Down
RAC1	0	-1.1569	0.51	0.859	0	Down
KDELR2	0	-0.6274	0.178	0.554	0	Down
CBX3	0	-0.6766	0.182	0.515	0	Down
CLDN4	0	-1.3116	0.023	0.517	0	Down
LAPTM4B	0	-0.5282	0.157	0.499	0	Down
PABPC1	0	-0.8386	0.916	0.957	0	Down
YWHAZ	0	-0.5878	0.424	0.825	0	Down
ZNF706	0	-0.6037	0.346	0.769	0	Down
MAL2	0	-0.6689	0.068	0.464	0	Down
CDKN2A	0	-1.6682	0.002	0.65	0	Down
ANXA1	0	-1.1681	0.32	0.725	0	Down

SET	0	-0.6472	0.313	0.714	0	Down
NEAT1	0	-2.0886	0.486	0.724	0	Down
CTSC	0	-0.7727	0.169	0.563	0	Down
TMEM12 3	0	-0.6565	0.096	0.532	0	Down
CRYAB	0	-0.7869	0.037	0.38	0	Down
APLP2	0	-0.5758	0.108	0.51	0	Down
FAM213 A	0	-0.9222	0.244	0.698	0	Down
SCD	0	-0.7489	0.038	0.385	0	Down
YBX3	0	-1.1267	0.293	0.742	0	Down
MGST1	0	-1.3174	0.198	0.759	0	Down
KRT6B	0	-1.027	0.078	0.372	0	Down
KRT6A	0	-1.6784	0.64	0.954	0	Down
NXPH4	0	-0.7384	0.023	0.378	0	Down
SHMT2	0	-0.5514	0.092	0.413	0	Down
R3HDM2	0	-0.6947	0.024	0.365	0	Down
DDIT3	0	-1.6396	0.114	0.521	0	Down
DCTN2	0	-0.9169	0.215	0.536	0	Down
OS9	0	-0.7137	0.077	0.459	0	Down
CDK4	0	-1.0969	0.16	0.682	0	Down
TSFM	0	-0.5023	0.075	0.366	0	Down
TXNRD1	0	-0.6553	0.121	0.489	0	Down
TMED2	0	-0.6532	0.117	0.585	0	Down
AL16143 1.1	0	-1.0551	0.061	0.545	0	Down
ZFP36L1	0	-0.6233	0.16	0.492	0	Down
IFI27	0	-1.9368	0.268	0.732	0	Down
CES1	0	-0.9815	0.138	0.533	0	Down
KRT19	0	-2.3946	0.04	0.781	0	Down
KRT17	0	-1.3954	0.903	0.954	0	Down
VMP1	0	-0.7564	0.069	0.468	0	Down
SUMO2	0	-0.5683	0.82	0.975	0	Down
SNRPD1	0	-0.6035	0.264	0.67	0	Down
DSC3	0	-0.7598	0.047	0.495	0	Down
DSC2	0	-0.6028	0.013	0.306	0	Down
DSG3	0	-0.9768	0.022	0.514	0	Down
DSG2	0	-0.5524	0.023	0.364	0	Down
PRNP	0	-0.5848	0.117	0.494	0	Down
DSTN	0	-0.7512	0.661	0.913	0	Down
CHMP4B	0	-0.5251	0.135	0.475	0	Down
CEBPB	0	-0.692	0.064	0.393	0	Down
PPDPF	0	-0.7657	0.714	0.944	0	Down

BST2	0	-0.5997	0.041	0.316	0	Down
FXYD3	0	-0.856	0.778	0.925	0	Down
SPINT2	0	-0.736	0.35	0.784	0	Down
C19orf33	0	-0.8127	0.127	0.598	0	Down
KLK11	0	-0.678	0.091	0.507	0	Down
RPS4Y1	0	-1.5149	0.066	0.462	0	Down
CXADR	0	-0.8081	0.043	0.517	0	Down
KRTCAP2	2.66E-306	-0.5061	0.205	0.552	6.04E-302	Down
PFN2	3.40E-305	-0.543	0.391	0.743	7.72E-301	Down
COX17	1.75E-303	-0.528	0.381	0.75	3.97E-299	Down
PPP1CB	1.94E-302	-0.6296	0.222	0.564	4.40E-298	Down
SDC4	2.88E-302	-0.5026	0.046	0.319	6.53E-298	Down
NTRK2	1.24E-300	-0.6313	0.259	0.606	2.81E-296	Down
ZCRB1	6.41E-298	-0.6782	0.234	0.54	1.46E-293	Down
LMNA	3.56E-296	-0.7758	0.419	0.747	8.07E-292	Down
RPA3	9.19E-294	-0.6105	0.143	0.454	2.08E-289	Down
HIST1H1C	7.77E-293	-1.5161	0.043	0.3	1.76E-288	Down
CLU	1.51E-285	-0.5949	0.113	0.408	3.43E-281	Down
KLF5	5.21E-282	-0.6982	0.154	0.444	1.18E-277	Down
HIST1H2AC	6.02E-282	-0.5925	0.031	0.276	1.37E-277	Down
ATF3	4.44E-280	-0.8937	0.151	0.447	1.01E-275	Down
LYPD3	3.26E-275	-0.75	0.079	0.348	7.40E-271	Down
CEBPD	8.06E-272	-0.8737	0.191	0.479	1.83E-267	Down
MALAT1	1.56E-269	-2.1261	0.993	0.95	3.55E-265	Down
ARPC5	1.72E-267	-0.6275	0.204	0.514	3.91E-263	Down
BNIP3	1.09E-261	-0.5664	0.131	0.423	2.48E-257	Down
SLC2A1	1.79E-259	-0.631	0.208	0.512	4.06E-255	Down
NDUFA4	2.42E-258	-0.7702	0.814	0.905	5.50E-254	Down
MYC	8.79E-258	-0.5051	0.149	0.443	1.99E-253	Down
CCNL1	1.31E-257	-0.5383	0.158	0.454	2.98E-253	Down
CYCS	2.67E-255	-0.6487	0.662	0.854	6.07E-251	Down
GPX2	6.18E-255	-0.7277	0.635	0.847	1.40E-250	Down
DDIT4	3.27E-251	-1.111	0.246	0.503	7.42E-247	Down
PCNA	1.02E-250	-0.7959	0.143	0.415	2.31E-246	Down
GJB6	2.37E-250	-0.6913	0.147	0.426	5.37E-246	Down
CSNK1A1	2.91E-247	-0.619	0.249	0.545	6.60E-243	Down
GPNMB	8.25E-244	-1.1898	0.455	0.731	1.87E-239	Down
LSM5	7.38E-237	-0.5384	0.288	0.594	1.68E-232	Down
H2AFV	5.96E-232	-0.6886	0.201	0.471	1.35E-227	Down
BRI3	4.08E-229	-0.5366	0.103	0.35	9.25E-225	Down

CXCL14	3.11E-221	-1.2181	0.376	0.634	7.07E-217	Down
SRP9	5.81E-221	-0.5574	0.376	0.673	1.32E-216	Down
CLEC2B	9.76E-218	-0.7869	0.109	0.351	2.22E-213	Down
ITM2B	3.87E-214	-0.6216	0.791	0.92	8.78E-210	Down
SOX4	1.52E-208	-0.647	0.167	0.42	3.45E-204	Down
PSMA2	1.11E-203	-0.5079	0.38	0.662	2.51E-199	Down
IGFBP3	2.42E-199	-0.8846	0.064	0.273	5.50E-195	Down
AKR1C1	6.77E-194	-0.8496	0.655	0.829	1.54E-189	Down
TNFSF10	8.48E-192	-0.5529	0.169	0.424	1.92E-187	Down
KRT13	3.36E-186	-0.8536	0.213	0.468	7.62E-182	Down
FGFBP2	4.72E-180	-2.0123	0.19	0.385	1.07E-175	Down
PTGR1	1.83E-177	-0.6163	0.373	0.605	4.16E-173	Down
SNRPB	3.22E-173	-0.6377	0.412	0.618	7.30E-169	Down
MT-CO1	6.45E-172	-1.0352	0.978	0.92	1.46E-167	Down
IFI6	2.20E-168	-0.8985	0.14	0.351	4.99E-164	Down
ZFP36	9.36E-166	-0.7237	0.425	0.598	2.13E-161	Down
TXN	7.09E-164	-0.8262	0.96	0.989	1.61E-159	Down
TM4SF1	8.64E-160	-0.522	0.266	0.502	1.96E-155	Down
BTG1	9.55E-158	-0.6464	0.688	0.767	2.17E-153	Down
KRT8	3.13E-154	-0.5718	0.285	0.519	7.10E-150	Down
FOS	3.11E-142	-0.6335	0.707	0.769	7.05E-138	Down
ADM	2.93E-140	-0.6009	0.292	0.516	6.66E-136	Down
SLC25A6	7.81E-140	-1.2588	0.876	0.853	1.77E-135	Down
HIST1H4C	3.42E-139	-0.7662	0.123	0.321	7.76E-135	Down
SPP1	1.20E-138	-0.764	0.289	0.501	2.73E-134	Down
GSN	3.19E-135	-0.5257	0.285	0.518	7.25E-131	Down
DBI	2.57E-131	-0.5261	0.713	0.875	5.82E-127	Down
ACTB	1.47E-125	-0.6957	0.994	0.972	3.34E-121	Down
NDUFA4L2	1.74E-113	-2.7022	0.41	0.507	3.96E-109	Down
SAT1	1.42E-112	-0.6073	0.583	0.699	3.23E-108	Down
UBE2S	1.98E-112	-0.6351	0.131	0.302	4.49E-108	Down
JUNB	2.11E-103	-0.5843	0.581	0.658	4.78E-99	Down
MT1X	4.62E-92	-1.2742	0.688	0.751	1.05E-87	Down
NDRG1	3.88E-91	-0.6515	0.359	0.515	8.81E-87	Down
JUN	2.48E-77	-0.5372	0.61	0.66	5.64E-73	Down
ISG15	1.35E-55	-0.8739	0.241	0.355	3.06E-51	Down
FTL	1.98E-50	-1.0466	1	0.995	4.49E-46	Down
CD9	3.28E-49	-1.2452	0.738	0.64	7.45E-45	Down
SPRR1B	7.00E-48	-0.8074	0.408	0.256	1.59E-43	Down
KRT16	2.60E-46	-1.8066	0.327	0.42	5.90E-42	Down
H2AFZ	5.05E-25	-0.5039	0.588	0.621	1.15E-20	Down

KRT14	4.60E-10	-0.7363	0.705	0.561	1.04E-05	Down

Supplementary Table 7: Differential genes expression between mLN and LN+ ESCC related to Extended Data Fig. 4c

gene	p_val	avg_log2FC	pct.1	pct.2	p_val_adj	Sig
S100A9	4.12E-141	-1.99581	0.162	0.857	9.35E-137	Down
S100A8	6.55E-138	-2.25072	0.064	0.833	1.49E-133	Down
KRT19	2.37E-122	1.212077	0.344	0.04	5.37E-118	Up
MT-ND1	5.90E-115	-1.95182	0.791	0.967	1.34E-110	Down
MT-CO3	7.29E-113	-1.61214	0.852	0.981	1.65E-108	Down
FABP5	1.25E-105	-2.03853	0.813	0.977	2.84E-101	Down
FTL	7.60E-103	-1.2752	0.997	1	1.72E-98	Down
MT-ND3	7.56E-100	-1.44089	0.788	0.969	1.72E-95	Down
MT-ND2	9.58E-97	-1.45838	0.838	0.98	2.17E-92	Down
MT-ND4	1.23E-93	-1.45197	0.846	0.981	2.80E-89	Down
LY6D	1.61E-90	-1.63072	0.204	0.82	3.66E-86	Down
MT-CO2	1.09E-89	-1.37056	0.863	0.98	2.46E-85	Down
MT-CYB	1.31E-83	-1.34414	0.779	0.961	2.97E-79	Down
S100A6	5.86E-82	-1.36236	0.779	0.958	1.33E-77	Down
AKR1C3	9.39E-82	-1.51342	0.039	0.613	2.13E-77	Down
ALDH3A1	2.00E-77	-1.72335	0.198	0.742	4.55E-73	Down
MT-ATP6	1.48E-74	-1.19268	0.813	0.966	3.35E-70	Down
CSTB	1.11E-72	-1.09281	0.732	0.925	2.51E-68	Down
MT-CO1	3.20E-72	-1.31861	0.849	0.978	7.26E-68	Down
MRPL21	2.37E-71	1.431125	0.601	0.252	5.39E-67	Up
FTH1	5.43E-69	-0.80805	1	1	1.23E-64	Down
KRT15	1.27E-61	-1.47685	0.265	0.737	2.88E-57	Down
HSPB1	5.22E-61	-1.08259	0.972	0.997	1.19E-56	Down
KRT17	1.93E-60	-1.24857	0.578	0.903	4.38E-56	Down
DAPL1	4.95E-60	-1.60723	0.145	0.613	1.12E-55	Down
S100A1	8.39E-60	-	0.911	0.986	1.90E-55	Down

1		0.89407				
S100A 2	1.45E-55	- 1.33713	0.631	0.929	3.28E-51	Down
IGHG3	3.41E-55	0.52725 7	0.416	0.112	7.74E-51	Up
RBP1	4.25E-52	- 1.08149	0.156	0.569	9.66E-48	Down
RPS7	5.70E-50	0.84468 4	1	0.996	1.29E-45	Up
BTF3	1.68E-49	- 0.72684	0.911	0.977	3.81E-45	Down
RPL5	9.69E-49	0.76981 4	1	0.988	2.20E-44	Up
RPS3A	1.79E-48	0.69218 8	1	1	4.05E-44	Up
RPL23 A	2.22E-46	0.56884 8	1	0.999	5.03E-42	Up
AKR1 B10	2.36E-46	- 1.05118	0.159	0.577	5.37E-42	Down
RPL32	3.30E-46	0.65497 5	1	0.999	7.49E-42	Up
CALM L3	4.53E-46	- 0.94312	0.182	0.595	1.03E-41	Down
PRDX1	1.47E-45	- 0.79169	0.841	0.958	3.33E-41	Down
GPX2	1.90E-45	- 0.91667	0.204	0.635	4.32E-41	Down
FXYD3	2.51E-45	- 0.80462	0.369	0.778	5.70E-41	Down
COL1A 1	3.08E-45	- 0.77087	0.011	0.392	6.99E-41	Down
PGD	3.87E-45	- 0.93188	0.162	0.538	8.78E-41	Down
CTSD	1.40E-44	- 0.84664	0.095	0.488	3.17E-40	Down
SERPI NB3	3.12E-44	- 1.43827	0.022	0.406	7.08E-40	Down
GSTP1	4.19E-44	- 0.68648	0.961	0.992	9.52E-40	Down
SFN	6.90E-44	- 0.96389	0.341	0.728	1.57E-39	Down
CSTA	1.07E-42	- 0.84296	0.349	0.773	2.42E-38	Down
ATP5M E	2.02E-42	- 0.65863	0.67	0.888	4.58E-38	Down
SPRR1 B	1.31E-41	- 0.68543	0.031	0.408	2.96E-37	Down
UBC	2.51E-41	- 0.64757	0.891	0.972	5.69E-37	Down
TXN	8.36E-41	- 0.67043	0.852	0.96	1.90E-36	Down
TALD O1	9.44E-41	- 0.81555	0.332	0.689	2.14E-36	Down
RPL8	2.11E-40	0.61124 4	1	1	4.79E-36	Up
MT2A	3.13E-40	-	0.564	0.828	7.11E-36	Down

		0.68029				
RPS16	3.22E-40	0.647319	1	1	7.30E-36	Up
RPSA	1.57E-39	0.773519	0.992	0.985	3.57E-35	Up
KRT5	1.60E-39	-0.7433	0.517	0.864	3.64E-35	Down
BNIP3	3.83E-39	0.81258	0.363	0.131	8.70E-35	Up
SPRR3	4.55E-39	-0.68374	0	0.339	1.03E-34	Down
AP2M1	5.57E-39	-0.77027	0.307	0.652	1.26E-34	Down
APOE	1.99E-38	-0.66346	0.196	0.577	4.51E-34	Down
APOC1	3.93E-38	-0.742	0.078	0.437	8.92E-34	Down
AKR1C2	1.31E-37	-0.88609	0.196	0.587	2.97E-33	Down
CBR1	2.15E-37	-0.82063	0.198	0.571	4.88E-33	Down
S100A10	2.37E-37	-0.70671	0.751	0.912	5.37E-33	Down
AKR1C1	1.00E-36	-0.71266	0.243	0.655	2.27E-32	Down
RPL14	1.29E-36	0.593275	0.997	0.99	2.93E-32	Up
RPL39	1.71E-36	0.590171	1	1	3.88E-32	Up
RPS19	2.96E-36	0.533964	1	1	6.71E-32	Up
LGALS3	5.59E-36	-0.78121	0.142	0.504	1.27E-31	Down
COL3A1	5.75E-35	-0.63959	0.014	0.332	1.30E-30	Down
NUPR1	7.71E-35	-0.79782	0.209	0.577	1.75E-30	Down
CLTB	1.08E-34	-0.71177	0.126	0.463	2.46E-30	Down
RPS20	2.99E-34	0.557015	1	1	6.78E-30	Up
SNRPD2	4.56E-34	0.721567	0.941	0.862	1.03E-29	Up
GSTO1	7.18E-34	-0.80406	0.288	0.619	1.63E-29	Down
CTSB	7.79E-34	-0.77048	0.154	0.507	1.77E-29	Down
NFE2L2	2.02E-33	-0.74682	0.215	0.588	4.59E-29	Down
RPL27	2.30E-33	0.525966	1	0.999	5.21E-29	Up
NQO1	3.32E-33	-0.6628	0.17	0.533	7.53E-29	Down
COX7C	4.00E-33	-0.51942	0.947	0.981	9.08E-29	Down
MT1E	4.41E-33	-0.7835	0.17	0.522	1.00E-28	Down
GSTM3	6.02E-33	-0.71296	0.291	0.639	1.37E-28	Down

ATP1B 3	5.04E-32	- 0.76207	0.218	0.568	1.14E-27	Down
MT- ND5	1.13E-31	- 0.78565	0.592	0.763	2.55E-27	Down
KRT6A	1.16E-31	- 0.62217	0.24	0.64	2.64E-27	Down
S100A 16	7.96E-31	- 0.58117	0.302	0.674	1.81E-26	Down
PTMA	6.44E-30	0.52246	1	0.999	1.46E-25	Up
COL1A 2	1.35E-29	- 0.56297	0.036	0.327	3.05E-25	Down
IGKC	2.04E-29	1.01604 5	0.858	0.679	4.62E-25	Up
RPL17	5.00E-29	0.63761 5	0.966	0.888	1.13E-24	Up
RPL29	6.05E-29	0.65653 7	1	0.999	1.37E-24	Up
IGLC2	1.53E-28	2.05468 5	0.472	0.221	3.47E-24	Up
TYMP	2.21E-28	- 0.65328	0.115	0.416	5.02E-24	Down
MGP	2.37E-28	- 0.53573	0.028	0.308	5.39E-24	Down
RPL23	1.24E-27	0.54728 9	1	0.997	2.82E-23	Up
MSRB 1	1.60E-27	- 0.52663	0.047	0.321	3.64E-23	Down
LGALS 7B	3.74E-27	- 0.75771	0.092	0.381	8.48E-23	Down
UQCR1 1	1.67E-25	- 0.51117	0.64	0.825	3.80E-21	Down
SPP1	4.30E-25	- 0.61538	0.031	0.289	9.76E-21	Down
HMGB 2	6.39E-25	0.79918 5	0.327	0.142	1.45E-20	Up
ADH7	1.63E-24	- 0.51959	0.064	0.328	3.71E-20	Down
ANXA 2	3.95E-24	- 0.55575	0.545	0.786	8.97E-20	Down
RPL31	4.33E-24	0.51079 7	1	1	9.83E-20	Up
GPNM B	9.86E-24	- 0.60737	0.165	0.455	2.24E-19	Down
ADIRF	1.71E-23	- 0.57655	0.182	0.475	3.89E-19	Down
TSPO	3.05E-23	- 0.58057	0.327	0.584	6.93E-19	Down
GSTA4	3.63E-23	- 0.57342	0.159	0.426	8.23E-19	Down
LMNA	4.90E-23	- 0.56428	0.151	0.419	1.11E-18	Down
ATP5M C3	2.97E-22	-0.5349	0.64	0.812	6.75E-18	Down
UPK1B	3.90E-22	- 0.57414	0.14	0.421	8.85E-18	Down
LAMP	6.51E-22	-0.5772	0.092	0.334	1.48E-17	Down

1						
ITM2B	1.28E-21	-0.67678	0.698	0.791	2.92E-17	Down
DBI	1.57E-21	-0.5157	0.503	0.713	3.57E-17	Down
KRT16	7.40E-21	-0.56208	0.078	0.327	1.68E-16	Down
ATP5F1D	1.71E-20	-0.5306	0.416	0.651	3.88E-16	Down
TKT	2.31E-19	-0.53496	0.304	0.541	5.25E-15	Down
LGALS7	4.83E-19	-0.52584	0.067	0.286	1.10E-14	Down
LDHA	1.50E-18	0.630165	0.777	0.68	3.41E-14	Up
HLA-B	2.83E-18	-0.502	0.777	0.871	6.42E-14	Down
SUB1	1.82E-17	0.560196	0.67	0.5	4.12E-13	Up
CXCL14	1.91E-17	-0.70078	0.142	0.376	4.34E-13	Down
IFI27	3.63E-17	-0.96298	0.067	0.268	8.24E-13	Down
TRMT112	8.64E-16	0.536225	0.601	0.467	1.96E-11	Up
DDIT4	1.07E-15	0.578776	0.408	0.246	2.44E-11	Up
HSP90AA1	3.66E-15	0.639193	0.986	0.959	8.31E-11	Up
ENO1	7.69E-14	0.535831	0.925	0.873	1.75E-09	Up
CFL1	1.11E-13	0.528268	0.964	0.939	2.52E-09	Up
TMSB4X	1.23E-11	0.603525	0.978	0.99	2.80E-07	Up
PGK1	5.25E-11	0.633568	0.508	0.396	1.19E-06	Up
CD74	2.15E-08	0.706594	0.749	0.644	0.00048867	Up

Supplementary Table 8: Clinical data of untargeted metabolomics cohort

**Non-metastasis Group**						
Patient ID	Sex	Age	Smoking status	Drinking status	TNM	Stage
1	Female	55	Non-smoker	Non-drinker	T1bN0M0	Ⅰ B
2	Male	54	Smoker	Drinker	T1bN0M1	Ⅰ B
3	Male	57	Non-smoker	Non-drinker	T2N0M0	Ⅱ A
4	Male	63	Non-smoker	Non-drinker	T2N0M1	Ⅰ B
5	Male	61	Smoker	Non-drinker	TisN0M0	0
6	Female	64	Non-smoker	Non-drinker	T1bN0M1	Ⅰ B
7	Male	71	Smoker	Drinker	T2N0M0	Ⅱ A
8	Male	65	Smoker	Non-drinker	T1bN0M1	Ⅰ B
9	Male	68	Non-smoker	Non-drinker	T2N0M0	Ⅱ A
10	Male	77	Non-smoker	Non-drinker	T2N0M0	Ⅱ A
11	Male	52	Smoker	Drinker	T1aN0M1	Ⅰ B
12	Male	66	Smoker	Drinker	T1bN0M1	Ⅰ B
13	Female	70	Non-smoker	Non-drinker	T2N0M0	Ⅱ A

**Metastasis Group**						
Patient ID	Sex	Age	Smoking status	Drinking status	TNM	Stage
1	Male	72	Non-smoker	Non-drinker	T3N1M0	Ⅲ B
2	Male	50	Non-smoker	Non-drinker	T2N1M0	Ⅲ A
3	Female	74	Non-smoker	Non-drinker	T2N1M0	Ⅲ A
4	Female	68	Non-smoker	Non-drinker	T2N1M0	Ⅲ A
5	Male	75	Non-smoker	Non-drinker	T2N1M0	Ⅲ A
6	Female	58	Non-smoker	Non-drinker	T2N2M0	Ⅲ B
7	Male	76	Smoker	Non-drinker	T2N1M0	Ⅲ A
8	Male	68	Non-smoker	Drinker	T2N1M0	Ⅲ A

Supplementary Table 9: Clinical data of targeted metabolomics cohort

Demographic variables	Characteristics	Train set				Test set		
		**metastasis group**	**non-metastasis group**	**p**		**metastasis group**	**non-metastasis group**	**p**
Age (years)	Mean±SD	63.7±8.3	63.5±9.0	0.909		63.2±10.8	63.3±8.4	0.975
Sex	Female	11	19	0.578		3	11	0.267
	Male	15	34			14	23	
Alcohol intake history	Yes	6	11	0.813		3	13	0.135
	no	20	42			14	21	
Smoking history	Smoker	6	16	0.508		3	11	0.437
	Nonsmoker	20	37			14	23	
Tumors history	Yes	1	2	0.987		1	2	0.998
	No	25	51			16	32	
T stage	T3	17	14	0.003		13	11	0.01
	T2	6	13			1	11	
	T1	3	21			3	9	
	Tis	0	5			0	3	

Supplementary Table 10: Detecting performances of each model in test set related to Fig. 5f

**Model**	**Accuracy (95% CI)**	**Sensitivity**	**Specificity**	**PPV**	**NPV**
Clinical model	0.7255 (0.5826, 0.8411)	0.7647	0.7059	0.5652	0.8571
Metabolic model	0.7647 (0.6251, 0.8721)	0.29412	1	1	0.73913
Stacked model	0.8235 (0.6913-0.916)	0.7647	0.8529	0.7222	0.8788

Supplementary Table 11: Primers and oligos sequences

**Primers**	F	R
NNMT	AGCTGGAGAAGTGGCTGAAG	TGGACCCTTGACT CTGTTCC
E-cadherin	CGGGAATGCAGTTGAGGATC	AGGATGGTGTAAG CGATGGC
METTL14	GTTGGAACATGGATAGCCGC	CAATGCTGTCGGC ACTTTCA
β-actin	CTGGGACGACATGGAGAAAA	AAGGAAGGCTGG AAGAGTGC
E-cadherin-CHIP	AAAGCCCTTTCTGATCCCAGG	GTGGGTTATGGGA CCTGCAC
E-cadherin-meRIP	TTACTGCCCCCAGAGGATGA	TGCAACGTCGTTA CGAGTCA

**siRNA and shRNA**	sequences
si-NNMT	CAGCTACTACATGATTGGTGA
si-METTL14	AAGGATGAGTTAATAGCTAAA
si-IGF2BP1	UGGCCCAGGUUAAGCAGCAGCAUCA
sh-NNMT	GCAGCTACTACATGATTGGTGACTCGAGTTCACCAATCATGTAGTA GCTGTTTTT

Supplementary Table 12: Differentially expressed genes after NNMT knockdown

Supplementary Table 12: Differentially expressed genes after NNMT knockdown

AccID	log2FC	FDR	Style	Si-NMT-1	Si-NMT-2	Si-NMT-3	NC-1	NC-2	NC-3
A1BG	-5.4747338	9.09445E-08	down	1	2	1	60	46	19
A2M	-11.9880142	0	down	12	14	12	32120	34198	43069
A4GALT	-1.534641581	1.39544E-15	down	285	267	268	685	491	521
AADAT	-1.355769817	0.002626674	down	61	103	69	177	71	182
AAGAB	1.089219712	2.88565E-12	up	1274	1424	1239	468	437	404
AAMDC	-1.645970904	1.04608E-09	down	201	219	188	553	454	339
AARS	1.094203276	3.04118E-17	up	4060	4416	3738	1310	1272	1473
AARS2	1.185769167	7.43005E-06	up	495	623	516	163	197	143
AASDH	-0.984696683	2.58887E-05	down	156	213	143	251	236	227
AASS	-6.108123351	2.73754E-62	down	21	37	29	1106	1137	2021
AATK	2.624507018	1.32849E-20	up	674	839	772	122	76	66
ABAT	-8.191600916	2.53935E-30	down	5	0	2	419	336	727
ABCA1	-5.240942409	2.33934E-43	down	43	70	68	1040	1741	2016
ABCA10	-9.647027477	3.72697E-20	down	2	1	0	258	474	979
ABCA13	6.36888585	7.86611E-17	up	177	220	181	5	0	0
ABCA2	1.049094057	0.00244646	up	2995	3198	3216	764	1388	1011
ABCA3	1.669923192	4.68007E-31	up	1552	1709	1543	408	283	388
ABCA5	-0.714769011	0.006628331	down	304	297	313	294	392	371
ABCA6	-10.58688435	6.70099E-26	down	0	4	0	576	1433	2249
ABCA7	0.820404921	0.000135945	up	382	444	428	161	180	160
ABCA8	-13.44441658	1.52003E-24	down	0	0	1	1396	2910	4135
ABCA9	-12.09085617	2.42478E-24	down	0	0	2	959	1991	3239
ABCB4	-1.940751884	0.008789422	down	18	34	32	158	35	42
ABCB7	0.872912626	5.27221E-06	up	930	941	908	320	369	384
ABCB9	1.029670353	2.57306E-06	up	246	243	217	95	66	86
ABCC1	0.853004179	0.033110552	up	656	733	678	390	133	310
ABCC2	5.677940604	4.2139E-26	up	2462	2709	2624	12	32	64
ABCC3	1.827725046	0.000610059	up	1035	1098	1057	405	90	164
ABCC4	1.783591436	8.43994E-08	up	846	937	787	154	230	135
ABCC5	-0.804756085	2.95531E-10	down	602	619	619	775	695	817
ABCC8	-3.575850753	0.023601191	down	0	1	3	26	6	3
ABCC9	-5.135678532	1.00683E-43	down	60	62	39	970	1674	1286
ABCD1	1.269357034	3.54682E-11	up	1027	1095	1033	289	333	301
ABCD2	-7.043956545	4.65201E-10	down	4	6	3	49	934	127
ABCD3	1.249768354	1.30192E-12	up	2445	2519	2287	727	760	665
ABCD4	-1.8380798	1.03166E-17	down	267	293	196	624	631	652
ABCE1	1.688198436	7.15064E-21	up	4062	4309	3778	872	953	831
ABCF2	1.488013326	3.22995E-16	up	2364	2530	2273	549	640	612
ABCG1	-1.688083878	1.11523E-15	down	307	360	336	686	824	764
ABCG2	-1.960236128	7.06604E-09	down	59	77	44	171	117	212
ABHD1	-4.336542805	0.000634166	down	5	1	0	14	50	19
ABHD14A	-2.148780614	1.32899E-07	down	41	47	36	198	99	97
ABHD17A	-1.735664459	1.50633E-22	down	378	368	415	1068	829	862
ABHD17C	2.805541596	4.01178E-08	up	515	543	495	80	57	20
ABHD3	1.07669131	8.47019E-12	up	836	860	709	285	255	269
ABI2	-0.632042461	0.000255185	down	984	1132	942	1259	858	1273
ABI3	-5.490110562	5.31816E-18	down	3	2	5	132	111	76
ABI3BP	-11.14592507	1.65769E-51	down	0	2	3	2185	2174	3722
ABL1	0.585016154	0.019166999	up	2848	3216	3037	1244	1162	1925
ABL2	2.108017709	5.40506E-44	up	3080	3327	3212	495	530	554
ABLIM3	-2.593178137	1.65719E-08	down	336	384	360	977	2351	1136
ABO	-6.193523338	0.003783671	down	0	0	0	2	19	6
ABRA	-6.630483327	0.000167094	down	0	0	0	22	12	5
ABTB1	-1.835567825	3.10201E-20	down	380	363	414	1019	769	1176
ABTB2	1.504046957	8.09764E-13	up	652	674	664	142	158	198
ACAA1	-1.420761879	4.19315E-06	down	499	522	457	860	1168	731
ACACB	-4.552386207	7.93329E-08	down	348	396	375	857	12254	4360
ACAD10	-1.157991507	0.006933483	down	176	207	208	238	441	232
ACAD11	-1.949721931	0.001725738	down	16	27	14	28	45	82
ACAD9	0.593323762	0.012358281	up	1224	1452	1226	535	686	592
ACADL	-12.51570116	1.55126E-21	down	0	0	0	889	350	1118
ACADS	-2.242649483	0.001985478	down	162	207	171	244	1168	291
ACADSB	-3.600419089	3.04081E-30	down	102	103	85	690	1031	731
ACAN	-12.25931716	2.32609E-19	down	0	0	0	1213	394	358
ACAP1	-3.036734036	1.01454E-13	down	15	13	21	102	102	81
ACAT2	1.697715974	7.65089E-05	up	2271	2467	2134	436	723	301
ACBD3	1.693528915	1.18051E-48	up	3380	3509	3221	800	697	723
ACBD4	-1.726216396	4.98026E-08	down	159	136	121	423	205	368
ACBD5	0.998380948	0.002283967	up	1507	1679	1500	378	660	600
ACBD6	0.878154828	7.62878E-08	up	912	863	930	382	328	338
ACBD7	2.506371116	0.000555181	up	21	33	42	4	4	4
ACCS	-3.540577499	2.37846E-20	down	66	116	79	616	473	1080
ACD	0.865103954	0.00022525	up	397	409	421	204	117	163
ACE	-7.05033621	2.41611E-29	down	6	3	7	280	706	488
ACE2	-2.103784044	0.019652523	down	46	54	64	23	284	171
ACER2	-2.972699986	3.10981E-05	down	56	44	50	93	504	200
ACHE	-2.686067806	0.020823529	down	21	6	13	39	128	7
ACKR1	-13.93604911	7.69038E-30	down	0	0	0	2388	1961	1792
ACKR2	-4.664104198	0.000530794	down	0	2	1	6	18	29
ACKR3	-1.912639131	4.44707E-13	down	380	412	388	1361	673	1175
ACKR4	-10.10476426	3.51252E-15	down	1	0	1	1002	132	506
ACO2	-1.517276481	0.032248332	down	1182	1309	1169	958	4724	1325
ACOT11	-1.114518331	0.000469657	down	63	74	61	85	114	102
ACOT13	1.062356297	0.006291861	up	1000	1150	1076	369	479	226
ACOT2	-1.918405027	0.023813335	down	339	298	327	210	1795	417
ACOT7	2.135027976	1.10536E-51	up	980	1098	931	172	143	172
ACOT9	1.166381724	7.52682E-15	up	2812	2918	2643	998	690	983
ACOX2	-7.291771176	6.05559E-32	down	2	2	6	265	438	397
ACOX3	-1.846363898	3.10282E-14	down	95	137	90	271	231	319
ACP1	0.863192562	4.08226E-17	up	2454	2572	2300	1001	847	1018
ACP5	-5.402418061	7.55974E-16	down	8	11	12	128	506	258
ACP6	-4.87244639	1.62676E-20	down	17	8	5	278	135	224
ACPP	3.268710096	2.73378E-11	up	91	102	78	5	5	10
ACRBP	-2.5353293	0.000665655	down	6	10	7	51	12	34
ACRC	-1.906722978	1.89013E-07	down	24	23	22	77	50	58
ACSL1	-4.769785009	1.91584E-06	down	722	797	710	1624	33476	4496
ACSL3	0.956233979	6.6039E-16	up	4474	4937	4450	1799	1400	1899
ACSL4	1.109135548	6.2412E-07	up	4048	4549	3984	1215	1553	1322

ACSL5	-3.994059465	4.62569E-07	down	127	116	116	334	2753	720
ACSL6	-5.812764485	7.74763E-05	down	0	1	0	16	13	14
ACSM1	-7.8242795	5.4268E-07	down	0	0	0	23	13	55
ACSM3	2.210868906	4.07366E-07	up	184	204	243	19	29	49
ACSM5	-4.227513053	9.60868E-09	down	5	7	6	35	62	143
ACSS1	-3.336398953	2.0555E-46	down	81	86	77	503	601	635
ACSS2	-1.671573704	0.020289213	down	1461	1682	1570	1214	6791	2031
ACSS3	-11.3935459	1.84236E-18	down	1	0	0	455	925	623
ACTA2	-10.99644274	3.28165E-60	down	31	53	27	82807	10718	72651
ACTBL2	6.150666957	6.78171E-05	up	22	16	17	0	0	0
ACTC1	-6.216578879	4.33831E-09	down	114	166	155	3279	17409	437
ACTG2	-12.10460135	3.86143E-31	down	0	1	3	7881	1010	4196
ACTL6A	1.492290581	1.81019E-17	up	1823	2122	1854	600	375	500
ACTL8	6.902119546	1.15287E-06	up	36	32	25	0	0	0
ACTN1	-0.785951894	0.015683684	down	6271	6902	6447	9877	4482	10176
ACTR10	-1.843277865	7.94491E-33	down	505	559	529	1609	1089	1391
ACTR2	1.020865835	5.11335E-10	up	10742	11820	10305	3692	3194	4661
ACTR3	1.214997893	1.25903E-06	up	9405	10094	9141	3818	1907	3180
ACTR3B	1.48887017	6.04008E-09	up	368	332	352	88	99	78
ACTR3C	1.948992245	6.21583E-06	up	93	77	69	18	15	11
ACTR5	0.810953864	0.017186519	up	396	437	339	234	104	143
ACVR1	-2.386019184	3.74579E-23	down	174	181	187	843	466	733
ACVR1C	-10.54251753	6.62348E-06	down	1	0	0	10	882	153
ACVR2A	-2.437127303	3.42377E-32	down	73	82	66	288	241	323
ACVRL1	-5.667558211	6.6534E-52	down	20	21	29	742	1042	706
ACYP1	0.796670939	0.001416656	up	246	215	222	114	73	95
ACYP2	-2.162435664	1.1014E-05	down	60	119	80	188	391	213
ADAM10	0.940155704	7.96507E-06	up	2933	2997	2816	900	1050	1278
ADAM11	1.438805272	0.012580882	up	129	118	87	43	10	37
ADAM17	1.150313266	2.79084E-11	up	1717	1773	1673	512	479	666
ADAM19	-9.228705339	1.42345E-17	down	2	0	0	333	253	269
ADAM20	-3.324542631	0.025378985	down	2	0	1	4	11	6
ADAM23	-10.33562913	3.9081E-13	down	0	0	1	135	564	242
ADAM28	-4.313119239	1.71376E-12	down	7	10	5	133	46	138
ADAM32	-2.319425894	0.013129214	down	4	2	3	14	11	7
ADAM33	-10.34601615	4.80966E-27	down	2	3	0	2012	382	2366
ADAM8	-1.825506373	0.000803358	down	14	16	10	48	28	25
ADAM9	1.519654991	1.75044E-05	up	4989	5873	5059	904	1045	2008
ADAMDEC1	-7.458860736	0.000168024	down	0	0	0	19	2	51
ADAMTS1	-3.994750374	3.93815E-62	down	411	476	524	6179	5484	4254
ADAMTS10	-2.393227254	4.84186E-09	down	172	158	170	841	291	789
ADAMTS12	-9.062440331	6.39852E-10	down	0	1	1	60	548	93
ADAMTS16	4.578382687	9.27113E-08	up	44	78	47	1	1	3
ADAMTS17	-5.716969285	1.51207E-08	down	0	1	2	46	29	38
ADAMTS18	-4.930181713	0.030615532	down	0	2	0	1	35	2
ADAMTS19	-8.094186364	7.52321E-09	down	0	0	0	38	21	50
ADAMTS2	-10.64185295	1.03553E-22	down	0	1	2	483	1903	867
ADAMTS3	-5.889838409	2.60995E-14	down	1	4	1	71	102	74
ADAMTS4	-4.017486614	7.22375E-05	down	37	32	15	247	631	29
ADAMTS5	-1.871526852	0.025774674	down	257	312	246	111	542	1466
ADAMTS7	-1.824724653	3.61757E-08	down	65	92	85	165	239	195
ADAMTS9	-10.50603316	3.91502E-18	down	2	0	0	382	1076	539
ADAMTSL1	-8.641496602	1.58403E-23	down	1	4	2	771	183	1086
ADAMTSL3	-10.90211695	6.34743E-25	down	0	3	0	1302	534	2305
ADAMTSL5	1.356194171	1.17172E-09	up	374	380	333	90	89	123
ADAP1	1.431255673	4.77479E-08	up	240	240	237	58	50	82
ADAP2	-3.929439011	6.62229E-26	down	20	35	26	403	192	291
ADAR	0.691025481	7.48993E-05	up	5392	6052	5389	2224	2232	2946
ADCK3	-1.99095427	0.000331735	down	323	359	349	425	1539	832
ADCK5	-0.811055983	0.004251188	down	69	89	79	123	77	97
ADCY1	-10.71854874	5.90647E-12	down	0	0	0	77	443	99
ADCY2	-8.856716347	4.90871E-09	down	0	0	0	48	22	117
ADCY4	-12.43161231	1.10237E-22	down	0	0	0	522	660	982
ADCY5	-8.264427486	7.06164E-52	down	3	5	17	2200	1275	2103
ADCY6	-1.014083536	0.000559966	down	1252	1250	1184	1539	2175	1485
ADCY7	1.782618685	2.72027E-09	up	2203	2518	2157	683	294	467
ADCYAP1	-7.839867403	5.77589E-06	down	0	0	0	69	12	12
ADCYAP1R1	-5.419342757	3.5226E-05	down	10	4	0	23	65	348
ADD1	-1.951391261	1.53423E-15	down	1500	1504	1530	3534	3401	5600
ADD2	-4.132680545	0.000865685	down	0	4	9	16	99	39
ADD3	-8.318173267	1.1616E-169	down	12	14	21	3809	2810	4132
ADGRA2	-3.012729748	1.80802E-56	down	242	246	249	1393	1487	1320
ADGRB2	1.568948465	0.016334826	up	476	470	508	84	204	45
ADGRB3	-8.992232387	3.41287E-11	down	0	0	1	91	84	219
ADGRE2	3.637814089	8.9158E-12	up	635	600	602	69	15	24
ADGRF5	-11.41427539	1.74127E-24	down	0	3	0	639	2794	2098
ADGRG1	1.227782677	0.005983468	up	1614	1818	1707	301	745	465
ADGRG2	1.75891121	4.14854E-05	up	618	816	630	107	209	103
ADGRG4	-6.630572802	1.6435E-05	down	0	0	0	13	11	15
ADGRG5	2.149329255	1.99344E-05	up	67	56	50	13	6	9
ADGRG6	-2.886517164	1.15754E-09	down	96	60	103	293	355	734
ADGRL1	-1.780353758	1.6807E-08	down	140	176	160	502	385	270
ADGRL2	-5.353157165	6.0886E-145	down	55	49	55	1463	1500	1648
ADGRL3	-10.39756401	5.39451E-16	down	0	0	1	497	211	342
ADGRL4	-8.821127882	2.20675E-42	down	2	6	5	740	1935	1363
ADH1A	-5.799506497	2.2421E-05	down	1	1	0	27	10	44
ADH1B	-14.70068517	1.38121E-66	down	1	3	0	31177	12557	33323
ADH1C	-8.963331356	3.52371E-14	down	0	1	2	90	592	334
ADH4	-7.001957998	2.79653E-08	down	1	1	1	15	108	145
ADH5	-1.515239895	1.02529E-05	down	2590	2854	2394	7425	2836	6006
ADHFE1	-6.754306755	1.61066E-57	down	4	7	9	557	377	615
ADI1	-2.441270365	1.44135E-13	down	245	367	294	1022	793	1706
ADIPOQ	-16.77095088	5.27415E-08	down	0	0	0	145	37158	2391
ADIRF	-11.41194632	4.03069E-81	down	1	10	2	11031	7836	5945
ADK	2.284160839	2.01019E-53	up	1934	2284	1969	310	301	287
ADM	1.750311942	1.97347E-10	up	1583	1670	1621	349	406	261
ADM2	5.766223178	5.35636E-05	up	32	23	27	0	1	0
ADM5	-1.472509942	0.037281762	down	12	37	25	83	36	27
ADORA2A	4.232789342	0.021003959	up	9	4	15	1	0	0
ADORA2B	4.770800632	8.48406E-10	up	326	354	328	6	18	1
ADORA3	-9.77598554	4.80572E-14	down	0	0	0	103	84	160

ADPRH	-3.102412101	6.12269E-19	down	46	65	37	265	355	264
ADRA1A	-10.09004295	5.72854E-08	down	0	0	1	7	619	142
ADRA1B	-1.637848833	0.009264168	down	88	100	103	179	74	410
ADRA1D	-10.36383456	2.96822E-09	down	0	0	0	435	15	95
ADRA2A	-11.22304264	3.2197E-16	down	0	0	0	139	444	326
ADRA2B	-7.607343396	6.64332E-11	down	1	0	1	68	114	91
ADRA2C	-4.700164147	2.59132E-08	down	67	67	64	1915	109	1815
ADRB2	-1.90826645	0.000591746	down	61	44	36	78	187	100
ADRM1	0.857212065	7.85245E-07	up	2052	2399	2044	970	823	745
ADSS	1.033099952	2.97708E-13	up	2029	2284	1840	764	585	794
ADTRP	2.233427193	1.99683E-05	up	115	138	128	15	30	11
AEBP1	-13.32643196	3.13862E-58	down	0	1	6	28161	7402	17478
AEN	1.698992214	1.37276E-14	up	944	982	956	274	152	212
AES	-1.245171021	1.29208E-17	down	2810	2977	2893	4978	4927	4650
AFAP1L2	-3.856649258	1.73038E-30	down	32	44	32	294	314	502
AFF1	-1.416915299	0.000263185	down	1095	1230	1169	1424	1753	3481
AFF3	-5.305146401	7.07745E-18	down	14	4	5	290	114	263
AGA	-2.256624464	4.70534E-18	down	91	82	78	367	240	251
AGAP1	0.77965912	0.010451231	up	1046	1118	1077	317	497	512
AGAP11	-6.922173911	4.51775E-32	down	2	4	5	439	192	334
AGAP2	-4.434576617	4.81213E-09	down	6	4	6	31	109	101
AGAP3	0.982697142	1.97743E-16	up	1700	1801	1653	667	582	603
AGAP9	-2.064646902	0.013145483	down	38	24	43	119	19	190
AGBL4	-6.077100744	0.004412283	down	0	0	0	2	17	6
AGBL5	0.677665388	4.76404E-05	up	591	689	601	261	265	307
AGMAT	6.548128205	3.98879E-35	up	411	520	394	4	5	1
AGMO	-3.811510247	0.001092951	down	3	6	4	4	77	41
AGO2	0.853025792	0.002566868	up	2325	2704	2460	822	757	1384
AGO3	-0.670583305	0.004393041	down	500	521	511	500	496	742
AGO4	-1.571794812	1.52079E-08	down	363	404	316	678	568	1056
AGPAT3	-1.774198801	0.004676009	down	394	448	415	456	1803	641
AGPAT4	-1.168855372	2.25448E-05	down	125	152	138	238	151	281
AGPAT5	1.576521117	1.89019E-28	up	1243	1414	1180	316	255	345
AGPS	1.922583876	6.38215E-41	up	4392	4971	4255	860	859	818
AGRN	2.875791952	8.28106E-73	up	6321	7368	6928	626	682	675
AGT	-2.881709589	1.39176E-07	down	206	234	209	960	1811	494
AGTR1	-10.72544072	1.31625E-15	down	0	1	0	234	335	731
AGTR2	-6.133453895	0.003080786	down	0	0	0	5	18	3
AGTRAP	1.253971347	4.29601E-14	up	744	911	773	275	224	223
AHCTF1	1.431563517	2.79133E-07	up	4163	4315	3976	969	787	1558
AHCY	1.714721757	7.58948E-25	up	4234	4432	4066	978	942	822
AHCYL1	0.64375193	7.40056E-10	up	4645	5069	4656	2275	1866	2414
AHDC1	0.728035127	5.78443E-06	up	937	1078	961	438	344	500
AHNAK	-1.478461089	0.000221823	down	22089	24350	22482	37132	26431	75209
AHNAK2	3.96939235	2.2876E-26	up	8684	9060	8965	243	351	619
AHRR	5.47043708	4.0785E-122	up	1364	1542	1387	28	17	24
AHSA1	0.827713051	2.67658E-06	up	2645	3153	2498	1355	937	1036
AHSA2	-1.272921269	1.03523E-05	down	1080	1187	1046	2178	1146	2461
AHSG	4.088338348	0.017543068	up	7	12	7	0	1	0
AHSP	-6.391251484	0.039696671	down	0	0	0	0	27	3
AIG1	-2.086120824	5.38976E-09	down	249	320	251	1282	507	724
AIM1L	9.206822992	8.21287E-18	up	524	589	547	2	0	0
AIM2	-6.861290036	7.04974E-06	down	0	0	0	13	18	14
AIMP2	1.484414836	0.000832222	up	878	947	849	228	313	121
AJAP1	-2.580343954	0.00549305	down	2	2	5	9	15	14
AJUBA	2.716948003	3.53815E-53	up	1279	1391	1144	146	107	161
AK2	1.568515229	5.36866E-10	up	7050	7772	7110	1533	2076	1562
AK3	-1.59365085	4.77947E-15	down	842	930	858	1851	2058	1674
AK5	-7.385749674	1.33241E-07	down	1	0	0	25	49	52
AK7	2.110071958	0.000122099	up	88	82	59	12	18	7
AK9	-1.871232439	4.00752E-14	down	54	55	57	142	124	167
AKAP1	-1.414508366	1.84325E-18	down	996	1120	1089	2237	1568	2315
AKAP11	-1.071544712	4.73111E-09	down	819	902	804	1143	1101	1530
AKAP12	-2.796182177	1.01322E-21	down	627	658	670	2250	3288	4049
AKAP3	1.955804245	2.17739E-08	up	117	108	114	17	22	23
AKAP5	-1.681215656	0.016630516	down	43	33	42	107	24	149
AKAP6	-6.206566185	5.8725E-70	down	17	31	20	1428	760	1416
AKAP7	-1.886999754	9.11275E-10	down	56	63	71	168	187	140
AKIRIN1	1.151336857	1.20563E-21	up	2045	2258	1863	689	611	669
AKIRIN2	-1.03450148	3.06399E-06	down	389	422	379	721	533	479
AKNA	-1.94413302	4.26086E-11	down	152	198	165	594	291	543
AKNAD1	2.268736652	0.00204103	up	41	54	44	10	1	10
AKR1B1	1.325294859	8.38623E-09	up	4387	4633	4422	1009	1311	1465
AKR1C1	-2.862967757	2.82834E-10	down	534	521	571	1791	4262	2078
AKR1C4	7.313349598	1.9597E-07	up	34	59	32	0	0	0
AKR1E2	-2.186322856	3.04717E-06	down	16	15	32	73	58	75
AKT3	-2.309762295	0.048871386	down	4	54	9	91	14	131
AKTIP	-3.198649149	1.6744E-56	down	109	129	111	828	576	889
ALAD	-2.259595267	6.12741E-18	down	470	504	470	1373	1940	1524
ALAS2	-6.999840575	0.009664137	down	0	0	0	0	21	28
ALB	4.134602071	1.33474E-07	up	46	80	69	5	0	3
ALDH16A1	-1.290006826	0.000479198	down	193	175	104	225	311	272
ALDH18A1	2.417888666	9.0381E-126	up	4051	4459	3971	606	495	560
ALDH1A1	-5.590839015	4.12245E-45	down	81	85	89	4148	1378	3452
ALDH1A2	-3.506674856	8.33102E-08	down	35	43	49	172	612	191
ALDH1A3	1.314267838	0.029073261	up	640	634	607	66	277	175
ALDH1B1	-1.251485532	0.047771948	down	1532	1664	1456	5078	756	2344
ALDH1L1	-13.05768239	3.32159E-22	down	2	0	0	1058	7707	2732
ALDH1L2	-2.425496109	6.85717E-41	down	152	194	144	710	561	596
ALDH2	-3.07214721	1.7444E-104	down	760	817	717	4812	3697	5274
ALDH3A1	3.431448649	1.24835E-05	up	105	132	89	17	2	3
ALDH3B1	2.120977992	1.12728E-18	up	930	1080	1001	196	107	195
ALDH4A1	-2.208827389	4.84027E-07	down	123	133	136	235	563	460
ALDH6A1	-1.855486517	9.97931E-05	down	442	506	478	616	1760	1173
ALDH8A1	-2.57211924	0.000384763	down	4	3	5	19	15	17
ALDOB	4.047408411	0.026999949	up	13	8	4	0	1	0
ALG1	1.365917725	1.54547E-06	up	712	775	700	218	231	147
ALG10	1.605902214	2.55065E-06	up	207	266	222	45	39	79
ALG10B	1.563498903	3.79036E-10	up	878	1037	842	177	199	285
ALG12	-1.091492	3.68586E-09	down	175	186	182	260	262	299
ALG13	-0.852754353	5.15357E-08	down	458	519	408	599	513	666

ALG3	1.955964595	4.45149E-18	up	1003	1049	986	152	200	200
ALG5	-1.370059482	3.14357E-12	down	272	284	237	500	502	445
ALG8	1.824292972	3.35081E-11	up	1451	1585	1341	314	337	216
ALK	4.521297598	6.435E-11	up	262	275	264	8	15	1
ALKBH1	0.712527649	0.00069119	up	375	340	324	158	150	142
ALKBH2	1.371424748	2.00474E-06	up	399	476	407	145	121	84
ALKBH7	-1.021486125	2.61666E-05	down	383	384	373	717	483	452
ALLC	-4.63334982	0.000609058	down	0	1	1	11	12	12
ALMS1	0.706614459	0.00531214	up	930	1126	961	438	309	578
ALOX12B	4.707873565	0.037203129	up	13	3	4	0	0	0
ALOX5AP	-5.900365092	5.04694E-21	down	7	9	9	485	118	493
ALOXE3	5.143228344	3.85845E-05	up	30	36	35	0	2	0
ALPI	13.30576482	1.51729E-27	up	2341	2694	2835	0	0	0
ALPK1	-1.915293772	7.39659E-21	down	147	189	142	515	358	410
ALPK3	-4.094898047	4.95429E-39	down	93	80	99	880	1324	1058
ALPL	3.544861044	0.000140242	up	2814	3163	2875	25	396	77
ALPP	14.40618603	2.05105E-32	up	5216	5956	5719	0	0	0
ALPPL2	9.172186906	3.45511E-13	up	148	148	152	0	0	0
ALS2	-1.439785415	2.47518E-14	down	283	275	257	486	532	546
ALS2CR11	-4.359567602	0.000195658	down	0	5	1	36	36	12
ALS2CR12	-4.842443658	5.15497E-05	down	4	0	0	23	22	38
ALX1	4.377733527	9.3341E-08	up	52	45	49	2	2	1
ALYREF	1.587810767	5.4531E-09	up	1283	1312	1295	425	265	235
AMBP	5.808989684	0.000902304	up	13	8	22	0	0	0
AMBRA1	1.067309959	4.95701E-08	up	900	1043	975	300	284	407
AMD1	0.686857071	8.18063E-06	up	2266	2477	2120	1176	794	1079
AMER1	1.371156896	2.25373E-13	up	504	603	505	177	130	136
AMFR	-0.592799068	2.91667E-06	down	1049	1139	1065	1280	957	1267
AMH	5.662960235	2.645E-13	up	94	141	120	3	0	2
AMHR2	2.332709999	0.037073713	up	8	10	17	2	1	2
AMIGO1	-0.775573771	0.00245316	down	145	167	127	174	139	223
AMIGO2	-1.290655715	0.018980073	down	360	369	357	879	202	874
AMIGO3	1.303359133	0.043798243	up	117	119	100	55	9	36
AMMECR1	2.230610057	3.40494E-30	up	690	779	671	97	113	112
AMMECR1L	0.666988677	0.000203125	up	1233	1271	1039	516	538	522
AMOT	-11.36286596	2.94464E-20	down	0	0	0	388	289	361
AMOTL2	0.673272905	0.00013606	up	2644	2855	2494	1055	1161	1329
AMPD2	1.607247616	1.76068E-09	up	2010	2272	2095	668	316	527
AMPH	-10.29298473	1.73562E-14	down	0	0	0	209	75	221
AMT	-6.09829657	6.67447E-26	down	8	4	12	591	194	425
AMY2B	-2.727341799	1.25561E-19	down	79	67	65	357	223	428
AMZ1	1.806935933	0.013138238	up	32	51	51	3	9	15
ANAPC1	1.877079134	1.25696E-25	up	1763	2092	1736	321	334	423
ANAPC10	-0.657048114	0.003082964	down	202	251	220	309	192	258
ANAPC13	-0.674542769	0.000744881	down	937	1079	1027	1419	1042	995
ANAPC16	-0.853805717	3.00456E-11	down	2105	2261	2024	2919	2237	3092
ANAPC4	-1.378376771	1.6445E-05	down	373	318	305	869	388	620
ANG	-2.757258241	1.28664E-08	down	31	17	24	175	71	109
ANGEL2	-0.667369058	0.001916848	down	403	490	359	487	365	566
ANGPT1	-9.61220126	8.31544E-34	down	4	2	0	882	641	1871
ANGPT2	-11.86620659	6.90467E-22	down	0	0	0	462	493	502
ANGPT4	-9.218557753	9.56218E-07	down	0	0	1	74	4	398
ANGPTL1	-13.27399924	4.30584E-22	down	0	0	0	1514	407	2097
ANGPTL2	-5.475071536	1.90712E-28	down	45	31	37	861	1820	786
ANGPTL5	-3.797909133	1.63302E-12	down	4	10	8	56	81	77
ANGPTL7	-10.91519898	2.96065E-13	down	0	0	0	296	50	442
ANGPTL8	-10.27991117	3.87412E-09	down	0	0	0	32	371	46
ANK1	-2.324508199	1.8956E-13	down	64	56	56	253	135	249
ANK2	-7.215946336	5.66571E-68	down	10	13	7	845	1243	1021
ANKAR	-2.677334548	1.75094E-11	down	27	19	26	97	85	149
ANKDD1A	-4.667253271	5.69285E-16	down	21	26	13	542	129	441
ANKDD1B	-6.183314364	0.002011775	down	0	0	0	4	18	5
ANKIB1	0.63521591	9.79414E-05	up	2237	2515	2189	989	934	1253
ANKK1	-4.906837843	0.028085206	down	0	0	0	4	2	6
ANKLE2	1.604887231	1.07355E-28	up	3107	3245	3124	811	719	680
ANKMY1	-0.778930246	0.02720072	down	60	86	85	96	66	122
ANKMY2	0.694623041	6.55785E-06	up	912	997	1018	447	418	418
ANKRA2	-2.05128862	7.40497E-12	down	124	119	112	339	239	481
ANKRD10	-1.375640652	2.64524E-07	down	1106	1191	1156	3005	1506	1945
ANKRD11	0.922066143	0.000153297	up	2709	3084	2945	924	912	1451
ANKRD12	-0.889018631	6.90605E-08	down	994	957	1027	1406	1052	1495
ANKRD13A	-0.95211171	0.000125713	down	642	673	583	981	582	1081
ANKRD13B	1.70239686	1.15601E-14	up	315	375	350	79	79	68
ANKRD13D	-1.059959846	0.001207252	down	236	251	243	530	234	336
ANKRD18A	3.53565477	8.86322E-06	up	95	96	98	1	12	4
ANKRD18B	9.398304391	1.51354E-13	up	192	149	181	0	0	0
ANKRD22	-6.289985359	0.001654256	down	0	1	0	47	1	15
ANKRD23	1.044761155	0.025942392	up	70	76	68	17	19	38
ANKRD27	1.588165109	1.78473E-19	up	1686	1878	1702	520	335	397
ANKRD29	-2.016928638	1.31131E-11	down	145	160	179	358	421	614
ANKRD33B	1.532872234	0.000362235	up	874	965	910	191	120	377
ANKRD35	-10.86569828	6.4325E-17	down	0	0	0	365	143	239
ANKRD36B	-1.12056107	0.002613515	down	82	105	94	146	88	206
ANKRD39	0.699585189	0.010081723	up	208	219	179	102	90	72
ANKRD44	-2.830503375	2.43998E-13	down	108	104	111	671	273	731
ANKRD46	-1.718709955	5.63042E-16	down	171	205	167	532	347	396
ANKRD50	-0.804372995	6.16145E-05	down	497	662	599	686	748	729
ANKRD52	1.577702358	6.30138E-13	up	2786	2980	2915	559	698	798
ANKRD53	-5.95889792	3.8782E-05	down	0	0	2	34	42	11
ANKRD55	-3.547124815	0.002932171	down	3	0	2	13	7	23
ANKRD6	-12.33078729	9.55862E-21	down	0	0	0	1007	298	774
ANKRD60	5.278617028	0.026903951	up	21	8	1	0	0	0
ANKRD65	-6.754774236	6.05377E-09	down	0	2	1	130	71	28
ANKRD9	1.851368391	0.001198797	up	1440	1611	1694	223	524	148
ANKS1A	-0.66610315	0.020393533	down	868	850	828	941	649	1321
ANKS1B	-3.030382697	1.60679E-05	down	33	25	13	163	41	222
ANKS6	0.862528643	0.000166644	up	842	826	808	329	236	413
ANLN	7.305877098	5.95408E-62	up	13175	14473	13526	109	47	31
ANO1	-10.97162238	1.27448E-32	down	2	3	0	3697	689	2958
ANO2	1.81122879	0.000222872	up	400	478	325	38	106	93
ANO4	2.802680264	0.038842945	up	94	81	60	2	20	0

ANO5	-10.84191563	2.79859E-16	down	0	0	0	156	329	216
ANO7	2.856610132	0.008797254	up	124	100	107	0	20	11
ANO9	1.294103284	0.015093975	up	55	62	100	27	22	14
ANOS1	-1.002372602	0.002506906	down	292	320	288	311	362	610
ANP32A	0.907189581	3.91467E-11	up	4356	4799	4006	1942	1446	1608
ANP32E	1.870854993	1.51333E-11	up	5629	5900	4950	1435	662	1159
ANPEP	1.751802225	0.000262849	up	1240	1395	1267	143	210	474
ANTXR1	-1.371051357	0.001766823	down	4099	4654	4203	9426	3373	11659
ANTXR2	-3.423324668	1.46958E-25	down	188	209	165	959	1362	1937
ANTXRL	-4.700537934	0.000310003	down	1	1	1	14	6	37
ANXA10	5.63669786	0.002899129	up	21	5	12	0	0	0
ANXA11	-1.101721266	4.34696E-10	down	1951	2297	2206	3238	2656	3998
ANXA2R	1.379797798	3.48695E-05	up	165	183	186	70	38	39
ANXA3	2.899426588	2.16721E-13	up	1968	2161	1778	152	108	311
ANXA6	-1.273989919	5.47937E-09	down	3246	3355	3207	5018	6307	5338
ANXA8	9.264366889	4.18982E-34	up	1111	1252	1094	0	0	4
ANXA8L1	7.996035723	4.96726E-31	up	583	643	566	2	0	3
ANXA9	4.791953141	2.07111E-23	up	176	194	173	6	2	6
AOAH	-4.182787532	4.17513E-10	down	9	15	13	261	48	184
AOC3	-8.143153934	1.0754E-258	down	32	42	30	6493	6120	8255
AOX1	-3.62781588	1.18553E-09	down	221	262	211	1200	846	4186
AP1G2	-1.767964945	2.17384E-09	down	132	127	101	328	191	364
AP1M2	10.26900859	4.67171E-22	up	1132	1265	1074	0	0	2
AP1S1	2.553833994	7.44686E-32	up	2406	2552	2450	350	306	237
AP1S3	5.459495605	3.52495E-54	up	496	599	511	10	5	11
AP2A2	-0.829783058	0.004670937	down	269	224	185	347	200	321
AP2B1	1.085260422	6.67586E-17	up	7462	8183	7438	2448	2469	2790
AP3D1	0.631532143	0.044925456	up	2363	2752	2527	829	1363	1256
AP3M1	0.985392577	1.93316E-13	up	2500	2809	2475	904	916	961
AP3M2	-0.62573859	0.007168133	down	427	480	425	609	342	526
AP3S1	-1.102477266	1.2183E-05	down	829	918	823	1627	849	1504
AP4E1	1.0358553	3.48434E-06	up	753	860	734	226	286	294
AP4S1	-0.941796111	0.000854327	down	91	89	92	128	91	156
AP5B1	0.743323289	0.040794858	up	882	983	968	273	509	396
AP5M1	-1.05574405	2.89245E-12	down	521	528	476	756	743	746
APAF1	0.592399033	0.02668691	up	871	964	861	409	300	574
APBA1	-6.478046132	0.00027535	down	0	0	0	13	4	19
APBB1	-4.470122086	1.09345E-68	down	31	50	45	616	594	770
APBB1IP	-7.071917412	1.62583E-14	down	3	5	11	162	1220	329
APBB2	-2.64473602	5.89912E-20	down	504	524	460	1971	1541	3172
APBB3	-1.961989343	6.67374E-08	down	128	132	106	518	216	296
APC	0.721672452	0.00045271	up	1799	1808	1666	668	667	941
APCDD1	-12.49303776	1.04065E-22	down	0	0	0	802	447	1053
APCDD1L	-9.345751821	3.70438E-10	down	0	0	0	138	23	104
APEH	1.371820506	1.53527E-22	up	2623	2784	2585	784	721	683
APEX2	2.301148647	4.46512E-19	up	1323	1514	1432	210	238	160
APH1B	-0.904391973	4.09743E-06	down	272	334	260	354	351	443
APLF	-4.627535138	9.50288E-10	down	1	3	8	47	80	82
APLN	-1.262285483	0.027559106	down	28	38	39	33	84	57
APLNR	-9.187734934	3.52876E-10	down	2	0	1	199	939	29
APLP1	1.160601554	0.001490261	up	227	321	252	62	69	123
APOA2	6.451869205	4.13648E-05	up	13	23	32	0	0	0
APOB	-6.027134908	7.45746E-14	down	5	2	8	252	68	408
APOBEC3B	4.973473059	0.028295083	up	336	319	343	24	0	0
APOBEC3D	-5.084763893	1.98477E-10	down	0	2	4	53	36	57
APOBEC3F	-3.728798324	1.41104E-14	down	7	13	7	115	66	74
APOBEC3G	-7.944145804	5.58009E-12	down	0	1	1	169	96	87
APOBEC3H	-6.263887611	0.000435231	down	0	0	0	15	4	12
APOBR	-5.093151837	9.82904E-18	down	4	6	2	125	63	106
APOC1	-5.023318938	2.79826E-13	down	15	10	19	462	442	103
APOC3	5.106152667	0.005498303	up	7	12	8	0	0	0
APOD	-11.9764995	4.13202E-60	down	6	4	2	3997	14820	14707
APOE	-7.521909325	4.396E-22	down	21	42	43	1594	9325	1974
APOL1	-2.630772112	2.63551E-32	down	178	191	142	851	743	643
APOL2	-0.619700578	0.013183792	down	534	622	582	720	666	498
APOL3	-13.05840335	1.29015E-25	down	0	0	0	909	984	1456
APOL4	-7.677547287	5.43779E-08	down	0	1	1	29	185	59
APOL6	-6.72029056	2.20274E-23	down	27	23	11	627	2635	1076
APOO	2.367056186	2.55006E-05	up	852	961	833	71	207	70
APPBP2	-0.998746625	4.29687E-12	down	619	741	632	929	922	963
APPL1	-1.528854967	3.23969E-16	down	427	535	412	897	794	1126
APPL2	-1.092185492	7.9435E-13	down	483	549	507	753	683	898
APRT	1.081464896	0.000423661	up	1604	1705	1465	574	630	376
AQP1	-5.792257387	5.90875E-62	down	478	578	509	27862	10398	24767
AQP2	-7.42286906	0.000411993	down	0	0	0	7	52	3
AQP3	4.741187372	1.20851E-42	up	2303	2509	2211	61	83	39
AQP5	-2.164488679	0.034072251	down	3	4	8	8	28	10
AQP6	-3.579147153	0.045669894	down	0	1	2	1	17	6
AQP7	-5.097323513	1.70457E-06	down	57	65	62	88	3350	684
AQP9	-5.811071212	0.000167059	down	0	1	0	10	19	13
AQR	0.771615885	3.0026E-05	up	1235	1505	1270	553	444	678
AR	-2.06576385	5.77691E-19	down	369	434	332	1460	813	1134
ARAP3	-0.827700393	0.041553604	down	342	452	390	386	684	380
ARC	1.451886907	0.024392823	up	41	36	47	19	5	9
ARCN1	1.105075961	2.09527E-20	up	6779	7415	6725	2381	1932	2623
AREG	2.616215811	0.007200103	up	161	144	118	9	35	2
ARF3	1.207301254	5.43433E-13	up	7454	7866	7306	2360	1829	2823
ARFGAP1	1.10161098	8.49273E-12	up	2081	2189	2089	831	542	751
ARFGAP3	-0.643572611	0.003361305	down	739	839	771	1043	610	985
ARFGEF2	0.731649417	0.013723518	up	2501	2736	2373	769	1146	1308
ARFGEF3	1.120689737	0.012117635	up	232	304	351	56	118	111
ARFIP2	1.606274276	1.3304E-18	up	2051	2162	1969	605	372	478
ARFRP1	-0.653486521	0.000288856	down	384	397	410	424	446	457
ARGLU1	-0.599216025	0.02687745	down	3802	4128	3690	3813	3033	5777
ARHGAP1	-1.343536273	2.94043E-10	down	1233	1317	1082	2097	1692	2800
ARHGAP10	-1.830889385	0.000499224	down	520	515	503	925	611	2445
ARHGAP11A	3.882517066	1.85811E-07	up	86	89	90	2	1	10
ARHGAP11B	4.974437833	9.38773E-44	up	586	609	590	7	18	15
ARHGAP15	-8.933906117	1.78517E-16	down	0	1	1	242	230	220
ARHGAP18	1.150402914	5.03686E-07	up	1599	1773	1517	440	579	527
ARHGAP19	0.792063233	0.001122486	up	465	481	423	174	211	171

ARHGAP20	-2.08276133	6.56523E-05	down	74	98	60	166	354	150
ARHGAP24	-11.52124401	4.16696E-19	down	1	0	0	617	487	1172
ARHGAP25	-7.193980953	5.18299E-24	down	3	4	0	169	279	266
ARHGAP26	-2.532704284	1.98534E-12	down	134	182	143	516	424	962
ARHGAP30	-6.522038412	2.72939E-32	down	3	4	8	298	222	474
ARHGAP31	-1.782892565	5.63669E-06	down	460	461	429	712	1499	1005
ARHGAP33	-0.971061184	0.005470858	down	74	105	80	115	83	165
ARHGAP39	2.837214567	8.28899E-11	up	390	330	396	34	53	22
ARHGAP4	-3.894849118	5.94474E-29	down	31	41	47	598	298	379
ARHGAP42	1.541694387	0.003931602	up	766	901	856	91	309	194
ARHGAP44	-4.279536919	4.23163E-17	down	6	25	15	278	143	215
ARHGAP5	-0.790613967	2.78823E-07	down	1177	1399	1317	1524	1427	1834
ARHGAP6	-2.9319976	3.20322E-09	down	84	70	77	300	232	748
ARHGAP9	-6.500077333	9.9444E-20	down	2	0	4	148	93	151
ARHGDIB	-5.833424163	3.9754E-118	down	71	74	63	3695	2026	2806
ARHGEF10L	-2.234440403	2.90987E-05	down	396	469	480	2369	486	1804
ARHGEF15	-10.81338581	4.6366E-16	down	0	0	1	278	670	383
ARHGEF16	5.481051805	5.57699E-30	up	823	839	958	6	10	26
ARHGEF17	-1.84739599	2.51908E-07	down	1088	1076	1046	3689	1392	3337
ARHGEF18	3.019653955	3.40095E-46	up	3674	3829	3898	302	270	433
ARHGEF19	1.485473677	7.68999E-07	up	333	356	334	125	66	71
ARHGEF25	-8.352219938	8.13879E-38	down	2	7	9	2150	555	1598
ARHGEF26	-3.977606321	0.005718456	down	5	0	2	15	5	63
ARHGEF28	1.128209368	0.011359757	up	972	1127	978	230	211	571
ARHGEF3	-1.06390961	9.54526E-07	down	363	288	308	451	442	536
ARHGEF33	3.54899143	1.2254E-12	up	172	164	134	17	4	8
ARHGEF35	1.695270683	6.20583E-09	up	132	131	141	34	26	29
ARHGEF37	-2.848185982	2.0472E-20	down	75	75	85	301	434	458
ARHGEF39	4.053533742	7.24729E-15	up	423	487	454	36	6	18
ARHGEF4	1.995725537	1.55493E-05	up	792	1055	1026	196	223	82
ARHGEF40	-2.616795151	2.24168E-10	down	260	275	277	791	1689	956
ARHGEF5	1.89162461	4.17067E-10	up	478	506	480	134	79	69
ARHGEF6	-2.763890194	1.47349E-24	down	196	268	227	852	1094	1361
ARHGEF7	-0.715038983	1.11837E-06	down	1184	1386	1128	1583	1156	1592
ARHGEF9	-3.011207014	1.03786E-13	down	175	238	174	1489	519	1427
ARID1B	-1.234938318	1.86303E-11	down	744	763	700	1243	965	1510
ARID2	-0.672028315	0.002108881	down	481	547	424	619	398	640
ARID3A	1.355332837	6.70058E-08	up	314	369	377	89	109	94
ARID3B	1.467243828	1.4977E-05	up	280	264	247	93	39	75
ARID4A	-1.108371491	0.000122713	down	487	588	514	657	650	1135
ARID5A	-3.289915568	1.63347E-07	down	213	199	219	2803	383	1394
ARID5B	-1.455825185	2.80285E-05	down	1278	1474	1394	4089	1548	2615
ARIH2OS	-1.781210049	0.004096075	down	17	6	18	40	28	34
ARL10	-8.868626254	1.99665E-11	down	0	1	0	91	88	181
ARL11	-5.128514617	1.72291E-05	down	1	0	4	59	11	61
ARL13B	1.062323715	6.39413E-09	up	572	668	584	222	159	243
ARL14EP	-1.249259932	1.1181E-09	down	352	391	298	725	463	580
ARL15	-1.945238778	1.64152E-10	down	248	274	245	730	832	505
ARL2	-1.214466254	1.32492E-06	down	197	259	290	490	316	435
ARL2BP	-1.218871643	1.42785E-24	down	656	709	594	1119	985	1134
ARL4A	1.198208066	0.00120354	up	1074	1151	1084	231	451	319
ARL4C	-2.265857762	2.5298E-06	down	65	85	105	263	411	175
ARL4D	1.274669222	0.005761834	up	399	413	438	199	106	65
ARL5A	-0.941216046	4.74479E-07	down	605	656	622	1016	637	942
ARL5B	0.914208225	4.45609E-06	up	1088	1196	972	415	434	368
ARL6	-0.799395227	1.06812E-05	down	156	184	169	224	178	229
ARL6IP4	-1.363086933	2.38826E-07	down	1119	1207	1049	2891	1456	1901
ARL6IP5	-2.144624998	8.16095E-26	down	1747	1869	1664	6508	3871	6415
ARL9	-5.390881913	0.013327471	down	0	0	0	11	2	4
ARMC12	-3.690362052	0.00227119	down	1	0	6	28	11	28
ARMC2	-1.606315508	2.57862E-05	down	42	45	27	74	66	107
ARMC4	4.554259024	3.84223E-10	up	100	92	76	3	5	0
ARMC6	1.490190634	1.71919E-09	up	738	821	722	190	221	159
ARMC9	0.687675983	4.18401E-05	up	423	471	425	221	178	183
ARMCX1	-8.476599611	3.19821E-48	down	6	1	2	928	527	861
ARMCX2	-5.316910348	5.10221E-51	down	22	19	34	933	508	722
ARMCX4	-10.85470726	1.51676E-17	down	0	0	1	498	292	648
ARNT2	-0.850054886	0.00149072	down	85	80	80	99	90	126
ARNTL2	1.016174865	0.048796703	up	643	678	543	169	338	124
ARPC2	-0.720713506	0.000258843	down	3605	3859	3358	5359	3189	4246
ARPC5L	1.818263922	1.73764E-07	up	2115	2414	2146	575	489	269
ARPIN	-0.869658197	0.00017024	down	552	630	518	772	525	930
ARPP19	-0.657506612	0.001430402	down	2771	3018	2634	3057	2437	4009
ARPP21	-8.939153451	0.037419488	down	0	0	0	49	0	153
ARRB2	-1.256670892	1.24698E-05	down	457	516	527	1018	523	1051
ARRDC1	1.505275298	4.15887E-10	up	762	847	815	275	157	180
ARRDC2	-1.001589643	0.000563502	down	552	648	545	878	524	1114
ARRDC3	-1.094226038	0.000981073	down	627	725	622	715	1244	972
ARRDC4	-1.961752421	4.83108E-05	down	127	178	149	276	632	301
ARSA	-0.94262087	5.66409E-05	down	356	407	384	428	531	597
ARSB	-0.769897301	1.06007E-05	down	251	291	260	333	267	375
ARSD	-1.642922903	1.87369E-07	down	186	229	189	332	534	449
ARSE	4.346385806	8.51282E-26	up	577	699	633	27	8	33
ARSG	0.697775514	0.002710263	up	280	286	286	142	90	145
ARSI	-3.956086114	0.00127076	down	0	1	2	12	11	10
ARSJ	-1.695380031	0.000432851	down	87	109	99	291	93	313
ART1	4.121116171	2.60633E-08	up	77	58	80	4	0	5
ART4	-10.37326394	8.95731E-15	down	0	0	0	107	235	165
ARVCF	-2.797335312	3.48846E-20	down	98	111	89	620	300	575
ARX	4.366979339	0.040709777	up	5	5	6	0	0	0
ASAH1	-2.170923493	1.53128E-09	down	1645	1722	1521	3631	7001	4608
ASAH2B	-0.887901652	0.034622237	down	68	57	47	71	93	59
ASB1	-1.247337773	1.07121E-09	down	356	415	345	651	473	771
ASB15	-20.96880415	2.48786E-08	down	0	0	0	0	70	0
ASB16	-7.117580632	5.21382E-06	down	0	0	0	10	19	25
ASB2	-6.770599435	0.000164774	down	0	0	0	5	11	27
ASB6	1.156158484	5.13547E-12	up	878	1014	860	348	230	304
ASB7	0.838474273	2.99518E-07	up	583	668	597	233	221	280
ASB8	-1.148734767	3.03169E-12	down	384	389	360	575	596	607
ASB9	1.9835384	1.37439E-08	up	211	228	204	29	30	57
ASCC1	1.048255159	2.37296E-14	up	1296	1599	1314	481	445	516

ASCC3	0.924168294	4.88076E-06	up	1654	1779	1621	546	569	777
ASCL2	-5.990593683	0.00069034	down	0	0	0	6	7	12
ASF1B	5.833760369	5.73164E-29	up	1425	1593	1497	37	13	7
ASGR1	-3.372612394	0.002969951	down	3	1	2	13	24	6
ASGR2	-4.503652636	6.85772E-05	down	1	2	0	17	15	16
ASIC1	2.533868289	5.27914E-10	up	385	455	436	48	73	32
ASIC3	1.345220333	0.013791945	up	88	93	87	11	29	34
ASMTL	-1.527035263	0.021741229	down	50	71	80	279	63	82
ASNSD1	-1.532125921	7.62074E-20	down	420	486	387	949	702	1018
ASPA	-9.02624261	1.1181E-10	down	0	1	0	82	212	89
ASPHD1	6.389603293	1.82976E-35	up	778	954	794	8	12	1
ASPHD2	-4.398947145	0.001919171	down	1	0	1	12	11	7
ASPM	6.533439737	7.96811E-22	up	5369	5993	5282	74	46	7
ASPN	-12.02523354	1.16671E-33	down	1	1	1	4767	1224	3170
ASPRV1	-3.576631871	3.52177E-08	down	6	2	6	50	28	43
ASRGL1	0.90975113	0.004068257	up	264	252	246	70	105	111
ASS1	3.268839826	2.92118E-09	up	25348	28722	26742	982	3368	1331
ASTN1	-5.089194734	0.000238572	down	2	3	0	54	58	4
ASTN2	-0.599678311	0.031316104	down	90	88	79	106	69	104
ASUN	2.672959562	8.03557E-31	up	2364	2459	2246	261	300	218
ASXL1	1.064464645	3.11627E-07	up	3121	3387	2985	897	1028	1288
ASXL2	0.62099322	0.000504238	up	1314	1375	1324	553	601	695
ASXL3	-7.000312019	3.30935E-07	down	0	1	0	44	23	32
ASZ1	7.677283083	2.10097E-08	up	59	65	36	0	0	0
ATAD2	2.702426754	1.07782E-84	up	3770	4085	3553	458	330	464
ATAD3A	2.048418967	3.17593E-12	up	1140	1084	1100	178	235	150
ATAD3B	1.271554558	5.30669E-08	up	685	633	718	259	157	190
ATAD3C	-3.823088352	0.004763038	down	0	2	2	23	13	4
ATAD5	3.799660188	5.80412E-62	up	649	761	630	42	32	30
ATF1	2.216862034	1.34414E-53	up	1929	2081	1876	305	248	350
ATF2	-0.997867933	1.27741E-11	down	694	745	629	1091	788	1071
ATF4	-1.65315179	1.50945E-11	down	2457	2716	2558	7972	4179	5342
ATF5	-2.620659556	8.55124E-10	down	101	112	113	774	383	282
ATF7	-0.962649562	0.00011869	down	886	948	837	1175	898	1660
ATF7IP	-1.16166652	3.65409E-09	down	761	806	712	1037	1183	1386
ATF7IP2	-0.725339989	0.017523494	down	97	112	110	154	123	97
ATG10	-0.6859754	0.003745277	down	110	100	102	135	106	117
ATG101	1.583057105	5.10648E-09	up	1442	1595	1527	493	322	271
ATG14	-1.134908938	2.5347E-05	down	405	391	338	598	409	784
ATG16L2	-1.938587203	2.83936E-11	down	124	130	167	453	260	453
ATG2A	1.405583848	4.21751E-11	up	914	1033	1010	230	233	331
ATG2B	-0.945570986	3.20631E-06	down	605	694	656	810	758	1114
ATG4B	-0.699636709	0.001654256	down	537	562	566	739	453	754
ATG9A	0.939087672	0.001821467	up	1723	1783	1704	466	752	685
ATHL1	-0.897523858	0.000306465	down	298	366	354	410	358	586
ATIC	1.577281031	8.49057E-38	up	2183	2277	2050	522	454	577
ATL1	-2.453899328	6.33707E-09	down	50	56	40	264	97	219
ATL3	-1.006225316	0.005955189	down	2323	2275	1983	4186	1612	3822
ATOH7	4.980240851	0.001058335	up	16	19	13	0	1	0
ATOH8	-6.506331789	1.77718E-83	down	13	11	19	937	723	1147
ATP10A	-8.902804128	1.7456E-27	down	3	1	2	713	270	1126
ATP10D	-1.117037803	0.009251415	down	574	694	713	1016	504	1603
ATP11A	2.323449802	2.04549E-07	up	3606	3913	3501	265	724	530
ATP13A1	0.621486429	1.36947E-06	up	911	1014	940	440	404	479
ATP13A4	-5.560825809	0.004804854	down	0	0	0	4	9	5
ATP1A1	0.82870782	3.25771E-05	up	9617	10253	9457	3360	3578	4785
ATP1A2	-15.04685718	1.1816E-33	down	0	0	0	5925	2963	4597
ATP1A4	-5.577801904	0.015375929	down	0	0	0	1	10	7
ATP1B1	3.951686614	5.251E-100	up	13704	14303	13871	716	658	541
ATP1B2	-5.231852159	1.32089E-14	down	4	4	10	211	190	77
ATP1B3	2.123369808	5.39436E-20	up	9728	10493	9444	1545	1856	1372
ATP2B1	2.243390945	2.50733E-96	up	3971	4279	3995	614	568	655
ATP2B2	5.881519548	1.37073E-20	up	203	187	187	4	1	2
ATP2B4	-0.939947464	6.65482E-14	down	3534	3862	3660	4844	4799	5393
ATP2C1	1.444996703	3.67589E-48	up	4710	5016	4681	1289	1178	1290
ATP2C2	3.773767315	0.012775991	up	10	13	15	0	0	2
ATP5C1	-0.814618823	0.019713326	down	2401	2742	2263	3002	3929	2123
ATP5G1	1.007033419	0.015290738	up	1645	1685	1490	582	756	328
ATP5I	-1.192044389	0.000452691	down	816	895	755	1765	1373	843
ATP5S	-1.246208154	0.000442394	down	83	74	67	96	147	130
ATP6	-1.794027258	5.39515E-37	down	25734	30369	25503	62678	63964	73496
ATP6AP1L	-2.618458058	7.61153E-05	down	38	34	29	255	41	162
ATP6V0A4	7.797458262	1.02822E-09	up	101	130	107	0	0	1
ATP6V0D2	4.559630977	0.000112745	up	37	40	26	0	3	0
ATP6V0E2	-1.152003469	0.002351431	down	212	231	206	280	458	262
ATP6V1A	1.530848359	2.19471E-09	up	2959	3133	2823	596	854	710
ATP6V1C1	0.587317125	1.21414E-07	up	1899	2186	2009	1022	894	963
ATP6V1G2	-5.829238441	0.003779806	down	0	0	0	15	3	5
ATP7A	1.23925641	9.64385E-07	up	1263	1323	1170	419	247	480
ATP7B	-0.667184912	0.030469452	down	146	128	114	118	141	178
ATP8	-0.826516734	0.033660058	down	2866	3150	2851	2203	4576	4169
ATP8A1	-4.245859592	3.99395E-25	down	47	35	74	552	826	714
ATP8A2	-5.662229568	0.003034275	down	0	0	2	48	1	28
ATP8B1	-1.911202038	2.68001E-07	down	456	441	368	1458	569	1435
ATP8B2	-2.188860105	1.94458E-08	down	456	535	496	1860	771	2301
ATP8B3	2.834826426	9.55733E-18	up	208	207	216	28	20	15
ATP8B4	-3.690301335	1.79104E-16	down	37	35	36	240	470	254
ATP9A	-0.724559304	0.000176152	down	1617	1914	1670	1770	1910	2404
ATPAF1	-0.90317921	0.024826447	down	760	803	741	855	1418	707
ATR	1.572091164	4.34985E-18	up	1333	1482	1428	304	349	355
ATRAID	-0.920288705	4.19098E-07	down	1203	1295	1321	2018	1616	1504
ATRIP	0.837974267	0.002112282	up	250	215	235	80	86	113
ATRN	-0.975190103	2.9114E-05	down	743	746	700	827	988	1233
ATRNL1	-6.87139605	5.54367E-10	down	3	1	7	216	36	718
ATXN1	-1.569317182	2.40678E-22	down	488	464	468	1115	797	1108
ATXN2L	0.881218666	4.15219E-09	up	2114	2230	2075	816	694	976
ATXN3	-1.024183219	9.64203E-12	down	308	322	282	436	407	474
ATXN7L1	-0.625620073	0.013189754	down	160	174	128	151	163	190
ATXN7L3	0.681221712	5.58694E-06	up	1270	1424	1265	581	493	685
AUH	-1.240657256	1.98446E-06	down	322	441	383	517	689	694
AUNIP	7.534925178	6.70199E-27	up	435	477	399	1	4	0

AURKA	6.038139771	1.7441E-105	up	3567	3753	3545	49	43	25
AURKB	6.270923912	2.34599E-26	up	1575	1731	1643	33	4	10
AVEN	-1.244133039	7.4682E-09	down	159	213	178	287	315	316
AVIL	-4.343842176	2.936E-24	down	6	12	7	137	92	133
AVL9	1.554150149	6.15105E-15	up	2022	2294	2062	433	514	587
AVPI1	2.77680636	6.53774E-47	up	2303	2511	2380	247	271	222
AVPR1A	-9.264753845	6.59436E-12	down	0	1	1	63	468	303
AVPR2	-1.595648321	0.011745556	down	30	34	26	32	100	54
AXIN1	1.683473502	9.93547E-22	up	1320	1281	1274	264	258	337
AXIN2	-1.160864776	0.009125014	down	150	136	124	200	117	348
AXL	0.726584372	0.010231249	up	3174	3414	3014	1474	873	1838
AZGP1	-8.927741818	2.83423E-11	down	2	2	2	42	1704	144
AZI2	-0.847689075	3.9513E-07	down	712	934	824	1073	1041	1025
AZIN2	-1.888902456	2.54184E-05	down	22	19	19	47	64	45
B2M	-1.321016543	7.24417E-09	down	5406	5830	4974	12969	7384	8684
B3GALNT1	-10.60501368	1.39302E-16	down	0	1	0	420	478	274
B3GALT2	-9.672365086	6.91093E-08	down	0	0	0	13	235	48
B3GALT6	0.773345164	0.000404881	up	467	570	498	235	219	180
B3GAT1	-5.273958945	0.009998326	down	0	0	0	6	6	3
B3GNT3	8.215796238	4.32828E-37	up	971	991	1009	0	7	0
B3GNT4	4.050649296	3.19008E-05	up	47	35	55	5	1	0
B3GNT5	1.337973991	0.001903301	up	612	520	603	174	214	92
B3GNT6	8.088310504	5.15163E-10	up	64	78	70	0	0	0
B3GNT7	-3.658780979	1.94792E-11	down	7	6	4	63	37	54
B3GNT8	-8.477955289	1.29132E-10	down	0	0	0	56	36	49
B3GNT9	-0.935273517	0.001126052	down	298	311	290	575	288	375
B3GNTL1	2.442839671	3.88244E-06	up	128	148	179	15	31	12
B4GALNT1	2.204708944	1.33042E-34	up	657	673	723	117	90	111
B4GALNT2	7.951751318	3.9094E-09	up	45	85	64	0	0	0
B4GALNT3	-5.641338052	1.92801E-18	down	6	12	1	235	135	305
B4GALNT4	4.835174378	6.34534E-20	up	569	587	627	7	26	10
B4GALT1	1.876645835	3.74305E-27	up	6345	7098	6363	1346	1330	1129
B4GALT2	1.826190199	2.42496E-19	up	2107	2365	2131	404	487	418
B4GALT3	1.37348435	1.60956E-11	up	1063	1103	1044	270	321	282
B4GALT4	1.946700473	3.15065E-15	up	2229	2567	2111	529	274	487
B4GALT5	1.148434796	2.90443E-10	up	2596	2868	2458	809	902	805
B9D1	0.887230279	3.69706E-05	up	320	305	295	129	118	106
BAALC	-9.229036884	2.51165E-09	down	0	0	0	33	144	46
BACE2	2.307228774	1.10892E-32	up	10462	11989	10166	1413	1305	1974
BACH1	1.263671695	3.55392E-16	up	2155	2521	2308	815	543	722
BACH2	-5.403458183	8.91259E-10	down	6	0	4	83	54	175
BAG3	-1.002759574	3.25466E-05	down	738	676	637	1294	803	848
BAHCC1	1.193255011	2.9527E-12	up	1347	1362	1422	402	383	502
BAHD1	0.760535219	1.31456E-07	up	983	1132	1023	450	433	428
BAIAP2	1.305597793	2.13986E-07	up	749	751	734	266	140	246
BAIAP2L1	4.481614767	9.38773E-44	up	2064	2242	2076	56	91	52
BAIAP2L2	3.587237431	6.19199E-21	up	194	204	175	17	9	8
BAK1	2.0539467	4.87589E-27	up	896	916	825	135	156	158
BANF1	1.025181889	1.35751E-10	up	3092	3477	2968	1351	946	1043
BANK1	-5.696233051	1.4594E-05	down	5	2	0	22	187	31
BARD1	1.298511783	3.93132E-05	up	523	527	479	145	93	210
BARX1	3.042088565	0.038799252	up	23	30	25	7	0	0
BASP1	4.20658306	1.60323E-42	up	12351	13271	12736	724	292	490
BATF	-6.736699119	1.71563E-06	down	0	1	0	33	29	19
BATF2	-4.029934529	2.16523E-10	down	3	5	4	56	29	56
BATF3	1.040300566	0.003517718	up	67	82	88	32	25	25
BAZ1A	0.882708735	0.000897496	up	1706	1858	1607	919	553	532
BAZ1B	0.883125796	1.55007E-07	up	3808	4148	3562	1406	1251	1791
BAZ2A	1.150736159	0.000150848	up	4134	4400	3995	1014	1090	1921
BAZ2B	-2.274489269	2.01017E-27	down	255	326	280	991	757	1228
BBOF1	-1.348688047	3.01394E-07	down	83	83	84	132	158	160
BBOX1	-6.874376914	1.46742E-05	down	0	0	0	22	15	9
BBS1	-1.392261366	1.13844E-07	down	279	328	315	560	408	769
BBS2	-0.750179312	1.15456E-06	down	622	717	619	885	618	850
BBS5	-2.390447733	2.81645E-24	down	47	53	57	200	189	195
BBS7	-0.662267259	0.00966199	down	313	338	274	403	236	413
BBX	0.673637291	0.009327745	up	3319	3740	3362	1574	1056	2067
BCAM	-2.956176755	2.04065E-84	down	236	309	289	1671	1391	1534
BCAN	-3.713404156	0.025966422	down	0	1	1	12	3	4
BCAP29	-1.110476364	9.74105E-08	down	1321	1415	1149	2386	1422	2215
BCAR3	1.296904209	1.14415E-30	up	1423	1584	1451	470	373	449
BCAS1	-5.317461268	0.035650765	down	0	0	0	1	2	13
BCAS2	0.742129356	0.000342436	up	1253	1456	1215	712	465	496
BCAS3	-0.657743493	0.000120867	down	330	340	278	355	317	391
BCAT1	-9.961113468	8.61826E-24	down	0	0	3	614	413	1145
BCCIP	0.676310608	0.00022035	up	1660	1743	1543	917	591	706
BCHE	-8.518898007	2.28377E-11	down	0	0	1	96	95	88
BCKDHB	-2.092458723	5.79061E-08	down	232	310	249	528	1063	738
BCL11B	-5.091598571	0.000309816	down	2	0	0	11	20	17
BCL2	-3.394444512	1.62192E-27	down	90	87	96	551	539	966
BCL2L12	2.010177822	8.60341E-21	up	408	456	507	83	77	82
BCL2L13	0.650893502	0.049626638	up	1586	1805	1476	567	927	660
BCL2L15	3.066927609	1.24697E-11	up	158	131	139	17	5	15
BCL2L2	-2.381492524	7.10801E-29	down	467	482	438	1625	1335	2212
BCL6	-3.126629221	9.82183E-23	down	412	393	411	1708	2401	3425
BCL6B	-1.655007971	0.006623675	down	167	165	137	382	515	121
BCL7B	-0.610332443	8.02249E-07	down	707	780	658	817	674	839
BCL7C	-0.798162178	0.005585727	down	565	671	650	1101	547	713
BCL9	0.94232314	0.001268781	up	775	912	795	323	193	414
BCL9L	1.319391962	3.14508E-18	up	2707	3110	2979	789	751	965
BCO2	-4.494462069	9.73376E-08	down	6	10	5	53	210	52
BCOR	2.364792554	3.24338E-14	up	2508	2769	2491	543	264	277
BCR	0.87264582	0.00079115	up	1995	2023	1980	592	780	944
BDH2	-2.378909878	1.12167E-12	down	335	405	335	1989	794	1260
BDKRB2	-1.355572415	4.90763E-05	down	64	61	79	167	93	116
BEAN1	-1.576488654	0.002088361	down	18	15	23	51	40	28
BECN1	-1.124442948	1.61229E-15	down	857	871	805	1263	1185	1479
BEGAIN	5.10058101	1.84901E-84	up	848	943	901	24	13	19
BEND3	2.047490397	9.4971E-13	up	181	168	174	27	29	34
BEND5	-9.774918496	2.13936E-11	down	0	0	0	220	40	96
BEND7	-11.01341859	4.89397E-18	down	0	0	0	203	259	346

BEST1	-5.696929348	1.92286E-16	down	1	3	4	71	111	109
BEST3	6.69834129	5.07843E-07	up	48	51	58	0	1	0
BET1	0.795808249	1.73947E-06	up	993	1147	966	430	432	401
BEX1	-5.85843985	0.003130223	down	0	0	0	7	12	3
BEX2	-8.669393813	1.7352E-06	down	0	0	0	121	5	42
BEX4	-12.96945175	7.25381E-24	down	0	0	0	1543	558	1120
BEX5	-8.793142788	9.15254E-08	down	0	0	1	225	11	120
BFSP1	1.039835499	0.001448709	up	171	177	185	84	55	46
BGN	-12.98394429	5.8735E-106	down	7	4	1	32449	12598	25595
BHLHB9	-1.91645099	1.97761E-11	down	55	48	50	173	110	131
BHLHE22	-7.003140002	5.143E-06	down	0	0	0	11	17	22
BHLHE40	1.85578792	0.007435906	up	2896	3080	2880	678	875	131
BHLHE41	-2.231684003	2.73518E-09	down	104	75	93	316	369	214
BHMT2	-9.601162481	4.98155E-19	down	1	0	1	387	367	346
BICC1	0.773461613	0.001276022	up	2223	2206	2091	887	1026	768
BICDL1	3.151989952	8.11542E-17	up	125	168	134	10	11	13
BID	1.718776634	2.00462E-07	up	749	893	886	188	224	125
BIK	5.677162024	5.08309E-13	up	124	111	128	0	4	1
BIN1	1.876588272	7.10303E-09	up	3124	3349	3135	707	728	400
BIN2	-6.752392224	2.27931E-20	down	2	1	3	202	94	173
BIRC3	1.540064676	1.10958E-06	up	1459	1622	1376	391	434	247
BIRC5	5.366086049	2.46147E-12	up	2779	2980	2656	109	31	8
BIRC6	0.626491104	0.003611115	up	3081	3205	3138	1294	1210	1853
BIRC7	8.561957816	2.61032E-28	up	685	692	755	0	4	0
BIVM	-1.385874066	6.23788E-11	down	196	175	154	372	303	301
BLK	4.901312645	1.45187E-06	up	79	80	88	0	0	6
BLM	4.263587588	4.27158E-34	up	1308	1357	1178	51	61	28
BLNK	-8.71598751	9.35093E-10	down	1	0	0	196	41	95
BLOC1S3	0.81227177	6.19332E-05	up	357	315	349	144	124	147
BLVRA	-0.805423116	4.80176E-05	down	582	565	498	846	575	633
BMF	-1.993470642	7.33964E-18	down	83	87	103	259	216	302
BMP1	0.838260931	7.86679E-15	up	1854	2029	1866	822	649	821
BMP2	1.614558798	1.26039E-16	up	834	938	877	211	155	253
BMP2K	-0.806332204	0.00066955	down	534	511	457	727	433	731
BMP3	-5.950811678	0.00170475	down	0	0	0	13	3	9
BMP4	-4.561783209	1.95206E-21	down	29	20	17	333	507	243
BMP5	-3.556018721	4.77939E-05	down	13	6	9	52	146	26
BMP6	2.212632157	0.001002567	up	1345	1572	1451	111	420	101
BMP8B	3.343905588	3.27998E-36	up	379	347	358	27	25	24
BMPER	-3.552746901	6.72946E-08	down	6	22	16	60	123	177
BMPR1A	-1.257594941	0.004617304	down	815	775	692	1818	551	1624
BMPR1B	-2.853805003	0.003762562	down	49	61	63	581	18	336
BMPR2	-2.079941062	6.367E-39	down	965	1015	917	2717	2555	3443
BMS1	1.482017448	1.0922E-26	up	2501	2813	2510	685	662	634
BMT2	-1.936354801	1.07957E-17	down	120	117	98	296	250	369
BMX	-1.79731963	1.07741E-06	down	106	97	95	188	317	217
BNC1	-6.224507441	0.010570671	down	0	0	0	3	23	1
BNC2	-1.934297083	3.61925E-23	down	154	153	127	376	353	450
BNIP1	0.742464503	0.012661044	up	239	236	254	124	107	78
BNIP3	1.574561606	0.003223499	up	3070	3337	3275	348	1196	679
BOC	-6.657547411	2.5664E-81	down	8	15	6	764	563	757
BOP1	1.696208428	1.01438E-11	up	966	1142	1002	266	239	172
BORA	2.830658951	9.11514E-47	up	613	720	561	70	56	63
BORCS7	-3.57230892	1.21456E-58	down	61	50	57	535	386	511
BPHL	-1.581315532	0.001446015	down	119	132	109	170	388	180
BPI	-5.845843827	0.00024922	down	1	0	0	23	14	7
BPNT1	1.558362714	1.11045E-32	up	1353	1394	1235	334	302	324
BRAT1	0.711941021	3.78381E-07	up	798	942	828	399	309	409
BRCA1	3.552089704	1.89086E-40	up	1692	1890	1630	83	127	101
BRCA2	3.307925575	5.72601E-45	up	675	714	706	45	40	66
BRCC3	1.016789511	4.21751E-11	up	1109	1164	1104	375	393	414
BRD1	-0.777076743	3.11709E-06	down	445	487	458	640	440	625
BRD2	-7.198654558	2.04214E-07	down	1	1	1	160	140	7
BRD3	0.724323803	1.71011E-06	up	1156	1316	1274	605	430	586
BRD9	1.516202879	1.14227E-32	up	1424	1659	1528	430	329	389
BRI3BP	3.344642986	7.27646E-27	up	449	533	482	31	44	26
BRINP1	-7.971310947	8.11035E-06	down	0	0	0	64	30	5
BRINP2	-6.878774954	0.014313625	down	0	0	0	0	32	11
BRINP3	-7.793333286	0.004554789	down	0	0	0	8	0	83
BRIP1	4.858333742	7.81969E-60	up	743	700	647	15	21	15
BRIX1	2.65488093	7.4538E-42	up	2918	3417	2808	443	273	321
BRK1	-0.912735795	4.50165E-06	down	2171	2411	2218	3920	2367	2887
BRMS1	1.148870393	1.37931E-10	up	1053	1250	1042	432	319	321
BRMS1L	-1.483626078	1.33117E-15	down	159	161	151	342	249	351
BROX	0.833987609	2.90495E-08	up	2024	2469	2141	977	699	981
BRPF1	1.144207272	6.53737E-13	up	730	832	749	271	241	229
BRSK1	-2.777112537	3.93766E-06	down	15	29	21	148	39	137
BRSK2	1.83739234	0.045299047	up	55	70	67	3	26	7
BRWD3	1.17534542	6.88832E-05	up	1197	1352	1222	344	283	570
BSDC1	-0.878628965	4.50737E-11	down	1038	999	962	1311	1174	1442
BSG	0.83300784	8.17798E-06	up	11959	12896	12301	4666	5312	4712
BSN	1.422438727	0.029630129	up	34	52	75	8	19	15
BST1	-4.750084594	1.61743E-24	down	10	7	8	124	183	166
BST2	1.50138854	0.000154672	up	4051	4349	3937	1625	888	602
BTBD11	-4.576140889	0.000971221	down	1	2	0	33	12	6
BTBD19	-1.01911064	0.014400372	down	48	64	44	60	57	108
BTBD2	-0.709310428	1.06324E-05	down	624	687	715	804	774	770
BTBD3	2.015150164	4.09824E-23	up	3129	3408	3095	513	625	537
BTBD6	-0.680187895	0.004342182	down	657	805	757	781	944	770
BTC	-11.39075898	8.96865E-07	down	0	0	0	647	16	446
BTD	-0.955299108	0.010553857	down	377	413	417	373	712	543
BTG1	-1.365610229	0.000365273	down	1499	1562	1570	1904	3851	2510
BTG2	-3.024002855	2.13119E-10	down	397	446	405	1439	3748	1749
BTLA	-4.033787202	0.015647267	down	2	0	0	8	4	12
BTN2A1	-1.004619519	0.002279082	down	593	515	516	1004	447	917
BTN2A2	-1.279973166	0.0007248	down	101	161	121	284	124	262
BTN3A1	-1.35874281	7.59319E-13	down	292	278	244	499	408	584
BTN3A2	-0.760828703	3.23314E-05	down	614	664	603	804	775	673
BTN3A3	-3.876828476	4.25101E-47	down	35	49	52	509	358	564
BTNL9	-8.241846853	4.03947E-23	down	6	5	8	233	1702	2081
BTRC	0.644038147	0.000141429	up	939	1007	842	401	423	437

BUB1	6.615054907	4.65486E-31	up	3833	4105	3684	54	23	8
BUB1B	6.696621945	1.25792E-44	up	2550	2478	2356	31	14	6
BUB3	1.099803359	2.13321E-16	up	2813	3069	2802	1087	767	1043
BVES	-4.564943486	6.8937E-43	down	21	19	17	264	307	385
BYSL	2.233191038	1.10828E-07	up	693	863	689	108	157	65
BZW1	1.117731974	2.45528E-08	up	5349	5704	5037	1943	1825	1466
BZW2	2.331418737	5.99304E-05	up	3830	4251	3697	351	962	270
C10orf10	-7.156770493	9.11001E-37	down	36	30	49	1517	5495	4375
C10orf105	-6.040214953	0.000460194	down	0	0	0	12	7	7
C10orf107	-3.419825927	0.003181553	down	1	5	1	28	5	21
C10orf11	-2.916158178	1.85642E-06	down	44	37	42	411	76	199
C10orf12	1.657857173	6.48155E-12	up	199	211	192	46	37	53
C10orf128	-12.10624881	1.3752E-22	down	0	0	0	510	538	679
C10orf2	2.282501828	2.30693E-08	up	732	730	732	63	146	104
C10orf25	-2.3605606	7.11979E-06	down	16	15	8	41	57	42
C10orf54	-1.844954007	1.00116E-39	down	488	546	498	1515	1154	1249
C10orf55	-5.918868052	0.00128026	down	0	0	0	13	6	5
C10orf76	-0.763232251	0.016565537	down	400	447	434	393	628	497
C10orf82	-2.647348912	0.04175659	down	1	2	1	6	3	9
C11orf1	-1.734660248	2.03286E-08	down	85	85	65	235	176	144
C11orf21	-8.966932533	2.78507E-09	down	0	0	0	59	105	25
C11orf24	1.150007678	5.48228E-05	up	1217	1410	1168	582	311	332
C11orf49	1.534578297	1.89918E-17	up	790	885	776	244	178	180
C11orf53	6.269177933	0.000187452	up	27	9	23	0	0	0
C11orf68	-1.061331696	2.06044E-06	down	385	429	445	800	470	617
C11orf70	-7.691572698	5.04504E-08	down	0	1	0	89	39	32
C11orf71	-1.083472827	0.000866533	down	77	139	104	182	160	135
C11orf74	-2.138552533	6.21406E-14	down	66	96	86	326	236	213
C11orf84	1.318042354	2.34694E-11	up	548	661	550	208	136	159
C11orf86	11.40807735	1.57555E-20	up	663	720	729	0	0	0
C11orf95	-1.429285003	8.2922E-16	down	372	343	382	739	689	671
C11orf96	-10.10387285	2.35953E-39	down	6	5	3	6946	906	3546
C12orf49	0.869813748	0.000945402	up	866	1003	812	300	405	321
C12orf57	-2.391214056	5.63076E-08	down	431	617	526	3400	1070	1518
C12orf65	0.629626373	0.021263838	up	447	386	409	249	135	195
C12orf66	0.837034885	0.024187795	up	178	166	138	48	78	63
C12orf73	0.782789772	0.005777108	up	279	296	197	99	111	106
C14orf1	1.448255289	1.75857E-24	up	1265	1460	1251	399	306	331
C14orf132	-4.453970344	1.33222E-13	down	57	62	83	1203	296	1771
C14orf159	-2.698777147	6.7211E-07	down	116	120	114	349	886	313
C14orf169	1.216763442	7.07278E-06	up	288	268	266	68	90	92
C14orf180	-5.810230813	0.00471053	down	0	0	0	3	13	5
C14orf28	-3.69470193	2.92228E-19	down	40	23	24	350	165	304
C14orf37	-5.885959067	5.30526E-34	down	7	14	10	422	251	646
C14orf79	1.432960404	3.59827E-09	up	264	289	306	70	81	74
C14orf80	2.42842881	1.14002E-08	up	394	383	411	83	24	54
C14orf93	-0.60242234	0.025719278	down	133	185	166	212	127	187
C15orf39	1.223959826	5.01448E-09	up	810	776	799	279	241	205
C15orf41	-2.480437852	0.000195988	down	109	94	111	187	758	240
C15orf56	-2.872400063	0.023664812	down	1	1	2	11	6	4
C15orf59	-6.131337592	3.17393E-35	down	8	2	4	270	218	212
C15orf61	-1.180061687	0.000100079	down	69	58	61	108	75	123
C15orf65	1.755036236	3.19545E-12	up	186	200	165	42	35	39
C16orf52	-0.716413176	5.63541E-06	down	315	345	339	397	334	439
C16orf54	-1.453525897	0.026582457	down	12	8	15	15	25	28
C16orf58	-1.039620533	1.90321E-19	down	588	651	613	989	807	913
C16orf59	5.406110255	2.49999E-30	up	299	297	295	9	4	2
C16orf62	-2.476234724	7.60818E-43	down	175	188	172	646	678	785
C16orf74	4.007894951	2.53273E-14	up	222	241	320	11	18	5
C16orf86	-4.41107372	4.89927E-12	down	3	4	7	105	43	68
C16orf87	1.686008981	2.24343E-10	up	458	521	402	128	98	78
C16orf89	-6.523971965	2.32253E-19	down	7	3	0	151	197	306
C16orf91	0.991889177	2.01312E-05	up	373	334	346	140	127	109
C17orf100	0.907018284	0.040241495	up	47	59	54	19	14	28
C17orf107	-1.990657234	0.020663112	down	3	15	15	50	27	16
C17orf51	1.946318999	1.47118E-10	up	1038	1261	1112	150	250	218
C17orf53	3.661227739	1.05041E-24	up	249	221	207	14	14	10
C17orf80	-0.61117817	6.79426E-06	down	429	494	417	510	450	492
C17orf82	-5.282896708	0.00138426	down	0	0	1	16	8	6
C17orf96	5.460075283	0.001529301	up	12	9	13	0	0	0
C18orf32	-2.425255947	0.009446871	down	3	3	4	12	6	21
C18orf54	0.818756895	9.37839E-05	up	266	283	278	106	114	112
C18orf8	-0.633171611	0.000735117	down	264	266	287	345	245	317
C19orf12	-1.756573043	0.000296851	down	271	304	269	468	1045	438
C19orf18	-7.537107113	8.18443E-08	down	0	0	0	30	22	21
C19orf33	5.862301453	4.78593E-65	up	1393	1575	1276	28	14	10
C19orf35	-5.818268607	0.001793541	down	0	0	0	10	8	4
C19orf38	-2.187037347	0.011140583	down	8	3	4	24	8	18
C19orf43	-1.029690246	0.000370463	down	1211	1283	1234	2618	1264	1606
C19orf48	1.671390392	9.74028E-05	up	780	894	703	266	171	92
C19orf60	-1.158188614	3.40031E-05	down	334	281	271	635	389	394
C19orf66	-2.424628267	2.31534E-24	down	142	137	156	674	398	611
C19orf68	-0.6394081	0.031405309	down	297	336	326	345	242	487
C19orf70	-1.018382966	0.006103203	down	444	412	416	829	626	373
C19orf71	-2.601563965	0.014385163	down	1	3	2	12	6	8
C19orf73	-5.309837463	0.000128657	down	1	1	0	33	13	11
C1GALT1C1	-0.724813724	0.001245077	down	372	470	373	568	342	528
C1QA	-14.0132806	1.36123E-28	down	1	0	0	4131	2619	6087
C1QB	-13.83474992	2.82052E-28	down	0	0	0	1995	1233	2590
C1QBP	1.840969304	1.20539E-07	up	4681	5030	4332	925	1193	609
C1QC	-13.78489465	8.03028E-28	down	0	0	0	1807	1193	2618
C1QL1	4.739363933	5.47779E-18	up	594	623	614	22	22	4
C1QL4	8.402441063	8.05401E-11	up	77	90	96	0	0	0
C1QTNF1	-7.117099613	8.293E-126	down	25	27	15	2772	1867	1986
C1QTNF2	-8.294219342	1.73309E-15	down	3	0	1	529	87	320
C1QTNF3	-1.740598872	0.039932215	down	21	44	29	94	13	123
C1QTNF6	1.928612905	3.29787E-06	up	697	785	678	139	180	75
C1QTNF7	-12.63876546	4.02158E-20	down	0	0	0	1166	257	1168
C1QTNF9	-7.559821007	1.42564E-05	down	0	0	1	19	104	11
C1QTNF9B	-7.040517242	0.003049783	down	0	0	0	2	43	2
C1R	-6.647788436	2.0509E-126	down	181	200	228	11038	13485	18848

C1RL	-1.613503415	3.62325E-15	down	462	504	501	927	961	1303
C1S	-4.707288849	4.02102E-50	down	745	840	761	9560	13989	19759
C1orf106	5.483824252	1.32549E-07	up	689	828	764	35	1	2
C1orf109	0.623637729	0.000760965	up	517	581	473	224	237	260
C1orf112	2.558392423	4.22787E-10	up	595	656	590	42	64	116
C1orf115	-0.815731931	0.003956687	down	459	500	482	466	691	621
C1orf116	6.845302213	8.97316E-14	up	155	179	155	0	3	0
C1orf122	-1.253239383	1.82068E-13	down	345	397	300	579	520	664
C1orf123	-2.707438969	1.66394E-15	down	161	193	185	1265	518	764
C1orf131	0.676259183	0.002286419	up	369	379	295	168	153	141
C1orf159	2.163951782	9.09435E-29	up	471	536	492	85	80	72
C1orf162	-3.601665491	1.26691E-39	down	72	89	68	615	494	877
C1orf186	-5.369392043	0.000254188	down	2	0	0	33	8	20
C1orf204	-1.241125857	0.008487665	down	34	31	25	76	36	41
C1orf21	-2.298202324	1.60227E-11	down	279	335	298	705	1297	1121
C1orf226	2.23311426	2.42393E-39	up	677	779	775	125	98	115
C1orf233	2.223233088	0.003367253	up	173	201	182	15	55	10
C1orf50	-1.015711965	5.66094E-05	down	101	101	96	180	116	135
C1orf54	-9.93832502	3.95627E-22	down	2	1	0	1274	346	529
C1orf61	6.454538111	2.11605E-05	up	30	21	17	0	0	0
C1orf74	1.193661904	4.2634E-06	up	183	167	166	64	47	50
C20orf194	-2.939251027	3.04167E-27	down	134	187	126	669	722	1032
C20orf195	3.749415867	0.000265408	up	36	55	57	1	0	7
C20orf196	-1.221528532	0.004924693	down	35	38	24	60	59	40
C20orf197	-7.099603253	1.17699E-06	down	0	0	0	17	15	22
C20orf202	-3.553287274	0.000718912	down	2	1	2	21	13	8
C20orf24	2.249367335	1.15941E-10	up	692	758	689	73	139	101
C20orf96	-1.645061465	1.94349E-06	down	67	61	36	133	118	112
C21orf2	-2.48177921	7.233E-30	down	61	70	68	311	216	267
C21orf33	-1.266104353	0.002760815	down	138	140	163	168	340	229
C21orf58	2.680581186	4.50227E-34	up	462	506	540	58	45	65
C21orf59	0.931072567	3.30858E-05	up	1118	1214	1178	564	312	448
C21orf91	-0.721181366	0.003287591	down	228	316	240	299	328	280
C22orf29	1.42734297	1.21944E-12	up	807	888	725	185	210	241
C22orf34	1.931080703	2.34119E-12	up	204	221	230	34	39	49
C22orf39	-1.75451513	8.71229E-13	down	255	287	239	805	429	661
C22orf46	-0.757300616	0.00017478	down	412	456	400	443	482	593
C2CD2	-1.601336246	1.92477E-06	down	475	498	476	670	1114	1309
C2CD2L	0.669006622	0.001918433	up	332	411	337	197	124	164
C2CD3	0.896970386	3.66838E-07	up	649	681	647	237	220	299
C2CD4B	-7.68890466	1.22877E-05	down	0	0	0	36	38	5
C2CD4C	4.68991403	4.73782E-19	up	137	185	151	7	4	2
C2CD5	-0.821464471	0.000460623	down	770	919	794	1255	706	1194
C2orf40	-10.54527665	2.53466E-14	down	0	0	1	627	123	433
C2orf42	-1.199044553	0.000569888	down	106	106	98	248	130	133
C2orf48	4.748785489	1.4271E-09	up	67	71	88	4	2	0
C2orf54	8.503182061	3.24752E-11	up	90	96	96	0	0	0
C2orf74	-1.213124487	0.007144529	down	254	270	219	578	177	502
C2orf76	-1.441338409	6.56583E-06	down	99	118	72	247	150	162
C2orf81	-1.875283585	5.24363E-05	down	19	21	16	59	29	60
C2orf82	2.917530725	2.93661E-05	up	33	41	54	5	5	2
C2orf88	-7.728803074	1.40834E-17	down	3	0	1	238	242	118
C3	-2.514148365	1.98482E-05	down	3140	3809	3637	4365	16038	21867
C3AR1	-11.70062413	7.74877E-17	down	0	0	0	564	124	665
C3orf14	-9.793681078	1.48961E-14	down	0	0	0	134	83	135
C3orf18	-1.01553754	5.19984E-05	down	234	274	251	481	308	306
C3orf22	5.014259221	0.009129386	up	12	7	6	0	0	0
C3orf33	0.682188274	0.007209429	up	116	134	127	60	51	56
C3orf36	-2.865247708	0.03297343	down	3	1	1	3	15	7
C3orf38	-0.848713408	8.75201E-05	down	323	295	278	478	310	368
C3orf52	3.094628425	7.9119E-23	up	381	444	393	24	42	34
C3orf58	-1.948030415	1.32279E-07	down	241	301	251	1042	378	800
C3orf70	-9.225242257	1.3275E-18	down	1	0	2	683	155	489
C4BPA	6.043676176	1.50323E-10	up	95	92	91	0	1	2
C4BPB	7.700535526	1.10275E-36	up	4277	4632	4250	3	12	30
C4orf19	-4.525893007	0.000694119	down	3	3	3	9	118	8
C4orf3	-2.12654766	1.46648E-40	down	1009	1101	990	3835	2486	3376
C4orf32	-1.397493715	0.002567132	down	349	383	397	428	1041	577
C4orf46	1.684751166	1.90278E-11	up	557	646	523	170	89	126
C4orf50	6.00435533	1.87251E-05	up	27	31	39	0	0	1
C5	-2.02384123	1.70104E-05	down	100	108	72	326	118	384
C5AR1	-3.415457131	8.33199E-37	down	36	35	45	354	260	270
C5AR2	2.184554625	0.002725916	up	95	109	101	12	4	33
C5orf15	0.723670357	0.000181214	up	1790	2166	1954	972	835	725
C5orf22	1.954272163	1.77785E-29	up	1812	1963	1805	327	361	328
C5orf30	1.217662447	1.74382E-09	up	466	497	452	126	141	164
C5orf34	3.953751551	4.11729E-78	up	807	848	820	46	30	38
C5orf38	-7.319546678	6.16517E-07	down	0	0	0	23	24	15
C5orf42	1.778767622	5.67601E-11	up	2429	2841	2423	388	522	672
C5orf45	-0.988264311	0.005954211	down	161	184	163	305	131	294
C5orf46	-4.64590412	3.52958E-12	down	7	14	22	202	114	469
C5orf49	-5.302064947	0.023969379	down	0	0	0	6	8	1
C5orf56	-4.582988791	1.39364E-23	down	7	7	5	127	82	117
C6	-9.780064074	7.44248E-15	down	2	0	1	125	1245	407
C6orf1	-0.718554704	0.041089729	down	138	193	159	274	156	145
C6orf120	-0.965670023	2.42335E-06	down	482	523	499	603	720	750
C6orf141	-9.25864795	1.9013E-09	down	0	0	0	26	57	159
C6orf163	-1.236628534	0.012806348	down	22	35	17	32	43	47
C6orf203	-1.680297441	2.08769E-07	down	115	141	135	363	315	206
C6orf223	12.91453474	3.37206E-26	up	1837	2181	1994	0	0	0
C6orf226	-3.232360708	1.4785E-06	down	4	25	12	130	66	76
C6orf89	-0.778383919	1.12938E-05	down	1639	1688	1537	1879	1656	2417
C6orf99	6.210150848	0.000124697	up	23	25	10	0	0	0
C7	-14.93147315	3.25846E-29	down	1	1	0	5238	29894	6231
C7orf26	-1.256214523	1.12897E-08	down	147	197	139	309	255	252
C7orf31	-1.786162342	4.64846E-06	down	41	37	25	90	58	107
C7orf49	0.958167291	8.36297E-10	up	1330	1340	1326	482	414	572
C7orf55	-4.634857314	0.008475181	down	0	1	0	9	6	4
C7orf65	5.213224204	0.007317129	up	4	12	13	0	0	0
C8orf22	2.50410097	0.021251749	up	18	15	21	5	0	2
C8orf33	0.746745491	3.08247E-08	up	1697	1806	1706	769	722	709

C8orf34	-9.371188066	4.09651E-10	down	0	1	0	285	32	211
C8orf46	-3.747739677	4.59857E-08	down	9	13	13	139	33	173
C8orf48	-8.739529633	1.83856E-10	down	0	0	0	35	60	71
C8orf82	-2.056295882	8.48926E-06	down	150	138	105	280	566	279
C8orf88	-7.26990558	2.03915E-26	down	0	1	5	265	188	209
C9orf116	3.170238385	1.02265E-17	up	143	163	125	13	10	11
C9orf139	-2.243558848	0.002725492	down	5	5	6	24	9	22
C9orf142	0.951961429	0.034060531	up	785	886	768	519	189	204
C9orf152	5.08750588	0.019503262	up	2	16	9	0	0	0
C9orf153	5.323819231	0.002388419	up	10	9	12	0	0	0
C9orf163	4.887244491	2.78034E-09	up	57	85	69	0	3	2
C9orf3	-2.138791228	0.000114198	down	679	706	704	3652	671	2465
C9orf40	1.793960123	1.17493E-12	up	506	580	456	141	87	89
C9orf47	-5.101119442	1.91709E-05	down	1	2	0	22	12	40
C9orf64	1.357037804	9.05706E-19	up	787	898	771	252	215	213
C9orf66	-2.66714686	0.009640286	down	1	2	3	8	9	10
C9orf69	1.946994537	1.04925E-14	up	2195	2444	2192	348	492	400
C9orf72	-1.842336658	9.71004E-09	down	121	160	156	266	379	459
C9orf84	6.158754075	5.21371E-14	up	131	151	123	1	3	0
C9orf85	0.63690452	0.000728121	up	358	344	327	177	128	168
CA1	-9.004338259	0.035969821	down	0	0	0	0	130	59
CA11	-1.584634756	0.014625479	down	163	102	161	566	97	288
CA12	1.846344659	8.74405E-11	up	1523	1710	1623	259	250	456
CA13	2.00171606	0.003715968	up	109	97	81	12	30	7
CA2	-5.914412379	0.000114624	down	0	6	1	13	230	22
CA4	-8.899447021	4.60544E-07	down	0	0	0	6	84	92
CA9	8.847392737	1.40362E-43	up	1188	1285	1411	3	3	0
CAAP1	0.864227643	4.78804E-05	up	495	572	522	203	224	188
CAB39L	-4.328783774	7.434E-11	down	81	133	66	728	515	2814
CABLES1	-1.565911077	0.001739383	down	346	395	319	412	1140	592
CABLES2	1.221328306	0.000459696	up	217	210	222	55	84	56
CABP1	-8.66891809	1.70137E-09	down	0	0	0	95	32	36
CABP4	-4.886429864	1.2416E-07	down	1	4	0	40	24	41
CABP7	4.825718655	0.012145044	up	7	7	8	0	0	0
CABYR	-5.017721645	7.94416E-07	down	3	1	0	40	31	21
CACNA1A	-2.54247946	4.15826E-07	down	14	11	8	59	41	37
CACNA1B	7.253591959	2.49609E-08	up	74	91	67	0	1	0
CACNA1C	-5.677168226	1.86683E-47	down	44	33	29	1462	710	1771
CACNA1D	5.25558053	2.83617E-33	up	430	394	401	14	5	4
CACNA1F	-2.835164665	0.004507416	down	2	3	3	24	10	7
CACNA1G	3.108296583	8.00453E-10	up	83	100	93	8	3	12
CACNA2D2	-6.684874168	5.49293E-11	down	2	0	1	100	40	85
CACNA2D4	-8.902674656	1.6438E-09	down	0	0	0	81	20	93
CACNB2	-3.710040316	3.36048E-18	down	16	25	31	321	143	217
CACNB4	-3.004868585	6.95935E-07	down	14	13	26	134	43	136
CACNG4	6.142028627	1.794E-07	up	64	59	74	0	0	2
CACYBP	1.508764422	1.18136E-11	up	4330	4587	3946	1402	932	889
CAD	2.799306008	9.19305E-68	up	3264	3514	3452	324	309	413
CADM1	1.679616012	0.00228448	up	1039	1160	1037	267	341	90
CADM2	-6.968340876	2.79893E-07	down	0	1	0	30	34	31
CADM3	-11.55802521	2.37405E-19	down	0	0	0	304	301	586
CADPS	-7.456782575	0.017602642	down	0	0	0	1	61	0
CADPS2	-8.486610171	1.8833E-22	down	0	0	4	249	301	475
CALB1	5.385695359	0.000294331	up	18	18	27	0	0	1
CALB2	-6.020340396	0.045623103	down	0	0	0	0	16	8
CALCOCO1	-2.765168636	5.10281E-22	down	606	641	586	2864	1929	4186
CALCOCO2	-1.580001529	5.95061E-10	down	1335	1530	1398	2942	2102	4111
CALCRL	-11.63206185	8.8437E-22	down	0	1	1	570	2021	1786
CALD1	-2.463069252	1.81558E-06	down	8545	9344	8318	34725	12145	58910
CALHM2	-4.228308628	2.54638E-65	down	62	54	71	977	626	921
CALHM3	8.92620371	1.79434E-12	up	120	140	119	0	0	0
CALM1	-1.55741517	1.88213E-06	down	5240	5766	4801	15296	6111	12332
CALM3	-1.128219402	9.91147E-06	down	2838	3044	2798	5483	2866	5353
CALN1	-5.316486238	0.024099564	down	0	0	0	2	2	12
CALR	1.560465578	1.25886E-13	up	25024	27765	24523	6840	6576	5056
CALU	1.410539396	3.53865E-14	up	17463	19309	17602	4325	4218	6009
CAMK1	-1.711684806	3.62687E-09	down	262	299	213	589	701	481
CAMK1D	0.839083395	5.6213E-07	up	728	740	696	270	285	302
CAMK2B	-5.634802699	2.85195E-08	down	4	4	4	81	289	27
CAMK2D	-2.287943428	6.20055E-16	down	433	469	461	1375	1931	1343
CAMK2N2	3.151450808	0.032758494	up	24	23	20	0	5	0
CAMK4	-5.362878554	2.08286E-05	down	1	0	1	19	16	24
CAMKK1	1.267614924	3.5939E-07	up	517	577	545	216	137	133
CAMKK2	-0.944028306	1.52314E-14	down	727	789	729	1023	975	1065
CAMLG	-0.817395453	0.043858021	down	766	810	685	1557	584	744
CAMP	-5.331816425	0.014935121	down	0	0	0	10	4	2
CAMSAP1	1.69846135	3.05655E-38	up	2112	2251	2257	505	466	477
CAMSAP2	0.878623567	0.000159965	up	3558	3833	3456	1370	1018	1844
CAMSAP3	8.576761447	4.27667E-15	up	295	373	406	1	1	0
CAMTA1	-0.850858297	0.028585976	down	629	753	723	1363	478	919
CAMTA2	-0.96573108	1.53112E-05	down	463	427	489	599	532	797
CAND1	1.100306045	1.21491E-28	up	4151	4236	4009	1491	1202	1428
CAND2	-8.36745413	4.20011E-20	down	1	1	1	284	167	261
CANT1	1.228774516	3.88745E-11	up	1428	1559	1394	397	465	456
CANX	1.141966465	4.00445E-14	up	26259	28601	25551	8191	8811	8716
CAP2	-1.788939606	2.48263E-10	down	318	407	355	667	999	945
CAPG	1.775920913	4.16576E-05	up	2286	2489	2326	780	445	254
CAPN1	0.869789836	1.32087E-08	up	2579	2712	2500	984	856	1200
CAPN10	-0.672619975	0.004398984	down	191	196	173	264	187	185
CAPN11	-1.665812206	0.009640684	down	10	11	16	18	37	27
CAPN15	1.876632778	4.24893E-16	up	1139	1226	1228	207	264	217
CAPN3	-5.919367053	9.97575E-30	down	5	5	9	346	155	335
CAPN5	-1.779748011	2.23243E-16	down	154	185	161	470	399	346
CAPN6	-7.162190976	1.54254E-05	down	0	0	0	34	7	17
CAPNS1	-0.697642117	1.98155E-05	down	2988	3311	2951	3235	3553	3809
CAPRIN1	1.215508033	2.3557E-45	up	9944	10516	9591	3318	2768	3119
CAPRIN2	-1.890491953	2.53346E-29	down	215	216	224	558	519	652
CAPS	-3.66646361	1.06041E-20	down	23	14	19	181	116	217
CAPS2	-1.837859487	0.000321027	down	49	85	51	138	81	256
CAPZA1	1.49275638	1.04421E-49	up	6127	6559	6101	1746	1338	1679
CARD10	2.2538315	7.07243E-08	up	1621	1716	1638	142	344	235

CARD11	-4.456584507	2.4634E-08	down	2	2	6	80	25	56
CARD14	6.036260459	2.03209E-38	up	363	459	467	6	3	5
CARD16	-3.473542125	2.7015E-11	down	27	22	11	179	190	97
CARD19	-1.105568063	0.006584455	down	257	260	234	577	194	406
CARD6	-1.314949103	0.005461302	down	238	236	270	214	502	583
CARD8	-3.257886919	1.16269E-34	down	103	143	151	755	928	994
CARD9	-2.440809798	0.001135247	down	10	14	9	71	13	47
CARF	-4.844652771	1.63554E-37	down	17	21	11	354	226	429
CARHSP1	0.980005659	1.00712E-05	up	2339	2485	2623	748	934	984
CARM1	0.589971658	0.000263704	up	936	945	964	485	437	420
CARMIL3	-3.810506284	0.01121951	down	1	1	0	7	5	8
CARNMT1	1.563944617	4.12476E-06	up	708	761	646	180	208	112
CARNS1	-2.32566345	2.17416E-09	down	27	55	37	157	94	175
CARS2	1.344210187	1.1084E-28	up	2102	2303	2055	674	553	581
CARTPT	-11.19748459	0.008202239	down	0	0	0	35	0	928
CASC10	1.916337405	8.24619E-06	up	171	145	157	31	39	18
CASC3	-0.922355057	6.44595E-05	down	1316	1558	1267	2020	1290	2322
CASC4	-1.490270877	7.25397E-08	down	1323	1358	1146	2921	1584	3259
CASC5	5.95102841	3.7199E-25	up	1898	1918	1799	26	32	5
CASD1	-2.086945296	9.04259E-28	down	165	197	167	501	545	539
CASKIN2	-0.598505507	0.007791339	down	513	514	471	575	571	455
CASP1	-3.240088429	9.9775E-14	down	57	78	45	416	518	242
CASP10	-1.527145048	7.24901E-07	down	153	111	106	215	232	313
CASP12	-9.267254345	7.84363E-11	down	0	0	0	98	30	121
CASP14	7.71024626	1.18734E-08	up	64	58	41	0	0	0
CASP2	1.46759048	2.57013E-23	up	1071	1129	944	271	257	278
CASP3	-2.056575991	8.07904E-09	down	267	307	276	1327	578	644
CASP8	1.007098155	0.000287388	up	782	757	665	200	242	336
CASP9	0.800034956	4.36293E-05	up	464	482	535	219	162	228
CASQ1	-10.47456323	1.00086E-14	down	0	0	0	217	237	94
CASQ2	-14.47013106	2.56154E-11	down	0	0	0	580	6603	1037
CASS4	-3.542195518	0.000294961	down	1	3	4	26	8	34
CAST	0.640822327	0.000188564	up	9672	10032	9271	4390	3530	5369
CAT	-1.017161014	0.046735984	down	1389	1521	1382	1095	3174	1671
CATSPER1	2.923294875	0.000141828	up	68	71	60	14	3	2
CATSPER3	-2.199845031	0.007450655	down	2	5	6	15	16	11
CATSPERG	-3.946853123	2.56612E-05	down	2	4	1	14	22	40
CAV1	-2.169810341	7.50618E-22	down	2789	3028	2665	8611	6725	12010
CAV2	-1.379193879	4.30584E-22	down	1429	1488	1342	2596	2635	2603
CBARP	3.882601949	4.09378E-17	up	160	200	146	5	12	7
CBL	1.997137843	1.20526E-22	up	3671	3812	3575	577	561	836
CBLN1	-11.11247837	0.008731378	down	0	0	0	0	638	164
CBLN2	-7.537697799	0.000242796	down	0	0	0	60	13	2
CBLN3	-2.338234611	3.52168E-09	down	18	28	17	96	65	65
CBR1	0.937244442	0.002568278	up	2110	2204	1998	1164	534	680
CBR3	-1.789659034	2.05495E-05	down	96	96	115	368	127	280
CBR4	-2.085417858	3.3986E-18	down	139	172	147	382	473	514
CBWD1	-1.171920114	0.000179083	down	225	238	216	430	396	252
CBWD6	-0.961850715	0.030595381	down	33	40	30	69	32	43
CBX1	0.752796206	0.006289518	up	3213	3603	2967	1811	873	1504
CBX2	4.503491653	1.44571E-26	up	1888	1899	1751	52	85	32
CBX3	1.720363647	3.67267E-22	up	8905	10043	8117	2396	1705	1743
CBX4	2.068595215	6.44346E-15	up	940	898	900	191	158	114
CBX5	1.860773178	1.66367E-26	up	6204	6605	6075	1080	1227	1369
CBX6	-1.323893002	0.000244878	down	939	1001	914	2537	919	1738
CBX7	-4.404651153	2.08488E-30	down	146	170	122	2604	1077	3048
CBX8	2.736809996	1.20454E-11	up	300	336	313	29	48	22
CBY1	-2.238915205	1.71539E-21	down	129	141	161	610	379	471
CC2D2A	-1.606806805	3.6749E-06	down	257	348	249	758	340	779
CC2D2B	-6.005955991	1.73159E-07	down	0	3	0	67	23	48
CCAR1	0.81748497	0.000720032	up	2999	3400	2920	1677	881	1244
CCAR2	1.080785219	5.70769E-25	up	2503	2656	2365	928	738	867
CCBE1	-7.744573142	4.13552E-06	down	0	0	0	10	50	20
CCDC102A	-1.351994327	6.23533E-06	down	101	103	131	258	143	216
CCDC102B	-4.969644217	1.00879E-06	down	16	1	4	85	283	86
CCDC103	1.821444341	0.00982017	up	90	102	89	8	34	12
CCDC106	-0.995325346	3.59944E-05	down	114	122	89	153	152	153
CCDC107	-3.014565842	8.61866E-13	down	96	105	123	1001	324	586
CCDC109B	-0.90086099	0.000307952	down	212	200	163	233	271	253
CCDC110	-4.376294855	3.25276E-06	down	2	7	2	108	21	36
CCDC113	1.979131535	8.9932E-14	up	450	445	433	69	94	74
CCDC115	-1.696001363	2.73413E-06	down	299	321	292	1010	372	766
CCDC117	0.876763109	4.25053E-06	up	1323	1383	1190	625	386	509
CCDC120	0.927052552	0.002968856	up	211	199	173	71	51	98
CCDC121	-1.323356083	0.00029771	down	91	116	63	194	103	187
CCDC13	-2.405713841	0.022095431	down	4	3	2	5	9	20
CCDC130	-1.708344117	1.41285E-08	down	158	194	184	534	255	476
CCDC134	1.140285171	1.58493E-08	up	286	359	285	106	83	111
CCDC136	-3.562976522	7.4351E-10	down	11	20	29	185	78	254
CCDC137	1.682886854	1.01065E-22	up	892	939	943	233	196	185
CCDC138	3.786259727	2.08688E-45	up	753	766	696	49	38	27
CCDC141	-5.811958773	0.000141002	down	2	0	6	5	238	60
CCDC142	1.642289066	1.02772E-15	up	425	429	408	98	75	116
CCDC144NL	-3.069479078	0.032223131	down	0	1	2	8	5	5
CCDC146	-6.010102208	2.44768E-22	down	10	28	12	867	272	1186
CCDC149	-11.74223619	1.16635E-20	down	0	0	0	586	294	485
CCDC15	1.036563498	0.00112574	up	108	92	107	41	31	35
CCDC150	2.597962056	1.04573E-10	up	111	169	126	12	19	16
CCDC152	-5.351825294	1.34535E-14	down	5	3	3	107	53	167
CCDC154	-3.720407085	0.005114304	down	3	0	1	13	5	21
CCDC158	-5.865290655	3.27248E-05	down	3	0	0	63	9	59
CCDC159	-2.931404903	3.25225E-12	down	41	47	16	207	163	191
CCDC17	-1.939781901	0.004892153	down	11	7	17	30	18	50
CCDC170	-6.911586916	5.24967E-09	down	0	0	2	62	64	44
CCDC171	-2.208322239	6.52181E-07	down	35	32	21	123	55	116
CCDC174	-0.824670986	1.40407E-06	down	298	283	301	386	316	414
CCDC177	4.83011734	0.00231645	up	10	14	19	0	1	0
CCDC178	-7.154262168	5.35226E-06	down	1	0	0	11	55	39
CCDC18	2.468136413	8.50479E-16	up	592	721	661	70	107	72
CCDC181	-5.788234923	4.71536E-05	down	0	0	2	49	15	17
CCDC184	-7.374544475	4.3998E-07	down	0	0	0	16	20	29

CCDC187	7.919865168	2.29066E-09	up	50	74	65	0	0	0
CCDC188	-5.047427372	9.49187E-06	down	2	1	0	15	24	31
CCDC189	2.041776448	3.96122E-05	up	122	178	165	46	19	16
CCDC190	-3.778399212	0.000424304	down	7	4	13	141	7	101
CCDC191	-0.81824811	0.00277581	down	111	150	102	149	121	185
CCDC22	0.8267058	8.28837E-05	up	402	466	421	216	147	155
CCDC24	1.401220444	0.000101121	up	218	312	276	99	41	80
CCDC28A	-1.873313875	4.05718E-13	down	142	138	120	451	248	355
CCDC3	-11.16511761	1.64186E-43	down	5	2	0	6460	1602	3735
CCDC30	-3.033057377	0.000649524	down	9	5	2	39	11	46
CCDC34	3.29533686	8.97657E-18	up	1244	1423	1228	142	88	52
CCDC36	-8.081188719	1.53694E-08	down	0	0	0	23	46	35
CCDC39	-7.453528701	1.80392E-06	down	0	0	0	37	21	11
CCDC40	1.435075026	0.002045599	up	136	161	133	22	31	60
CCDC42	-4.886882015	0.042064196	down	0	0	0	5	1	6
CCDC43	1.26584955	2.58137E-08	up	949	1174	987	280	343	284
CCDC50	-1.609386831	1.62345E-16	down	1114	1279	1171	2326	2185	3233
CCDC51	1.700655647	2.07563E-16	up	748	860	793	182	188	151
CCDC53	-2.894270318	1.46608E-24	down	120	153	121	965	487	651
CCDC58	1.856991888	1.46108E-13	up	812	848	671	151	173	128
CCDC61	-1.82970504	6.92468E-07	down	62	76	48	173	96	206
CCDC62	1.762918549	0.026121772	up	40	23	18	8	6	3
CCDC65	-5.508516223	0.020404847	down	0	0	0	1	4	13
CCDC66	-1.276541319	3.5238E-07	down	221	217	230	395	276	493
CCDC68	1.730457992	1.84273E-05	up	701	767	760	91	147	238
CCDC69	-10.75146393	3.31616E-33	down	1	1	5	1199	4987	2045
CCDC7	-1.277419965	0.003305243	down	44	48	46	54	68	116
CCDC71L	-0.783025398	0.005101486	down	423	386	404	386	468	629
CCDC74A	-7.914366886	1.13842E-09	down	1	0	0	72	46	68
CCDC77	1.801124026	2.64652E-08	up	540	683	578	173	71	129
CCDC78	4.807769224	5.12676E-05	up	87	119	91	0	7	0
CCDC8	-8.710725225	3.56828E-23	down	2	0	3	912	274	353
CCDC80	-4.152440067	5.56286E-37	down	587	687	573	5612	10012	7150
CCDC81	-3.715241274	2.06592E-05	down	1	7	4	65	21	27
CCDC85A	-1.91642614	0.000643414	down	56	30	32	57	123	134
CCDC85C	3.560448461	7.32862E-09	up	1968	2208	1900	70	222	55
CCDC86	2.288537826	1.9022E-13	up	1258	1297	1323	159	241	154
CCDC88A	1.357374753	3.49416E-07	up	4161	4581	3948	1397	729	1449
CCDC88C	1.092475993	0.003597787	up	361	466	482	95	174	160
CCDC89	-6.815961089	1.07667E-06	down	0	0	1	29	34	22
CCDC90B	-0.656676251	8.70717E-07	down	635	689	617	813	662	700
CCDC92	-2.609917741	7.99658E-11	down	19	24	20	75	108	86
CCDC92B	2.447699122	0.000524807	up	27	33	47	3	4	7
CCDC93	0.872074222	8.39889E-05	up	1750	1889	1656	574	738	723
CCIN	-3.892379082	0.029888182	down	0	2	0	13	4	4
CCL1	6.993705222	4.98747E-07	up	35	33	31	0	0	0
CCL16	-4.455011812	0.04044343	down	1	0	0	1	3	13
CCL18	-9.044918692	1.16477E-07	down	0	0	0	24	146	23
CCL19	-9.999795718	6.53559E-11	down	0	0	0	102	240	40
CCL2	-4.622812078	2.97036E-07	down	56	60	79	2111	1209	97
CCL20	3.342614205	0.013546165	up	23	24	22	5	0	0
CCL21	-8.872519216	1.17339E-09	down	0	0	2	93	465	70
CCL23	-6.648116853	2.89964E-05	down	0	0	0	9	14	16
CCL28	1.145100207	0.004845086	up	142	150	143	46	26	70
CCL3L1	-5.67584072	0.015939838	down	0	0	0	16	1	4
CCL5	-5.010627181	0.034768924	down	0	0	0	9	2	2
CCL7	2.417124458	0.016985827	up	18	22	21	4	4	0
CCL8	-3.405247665	0.000212392	down	14	11	13	221	54	20
CCM2L	-5.811311363	2.82605E-13	down	10	6	0	140	279	203
CCNA2	4.584290352	1.00443E-22	up	2278	2620	2332	134	38	47
CCNB1	6.055988263	6.96061E-47	up	4867	5255	4897	81	55	24
CCNB1IP1	-0.818353265	0.011866301	down	638	717	599	1194	511	784
CCNB2	6.112905011	0.000152866	up	1719	1906	1726	50	7	0
CCNB3	-2.476410998	0.008382644	down	2	2	5	17	10	9
CCND2	-7.974916282	1.04731E-51	down	6	12	7	2308	738	1510
CCND3	1.54123789	1.24072E-24	up	2942	2987	2865	665	677	803
CCNDBP1	-1.52035188	2.22891E-17	down	505	586	469	1139	816	1243
CCNE1	5.239760594	2.7409E-40	up	811	916	701	17	21	7
CCNE2	3.975479728	3.72402E-34	up	613	570	583	25	35	19
CCNF	5.068877173	1.06383E-30	up	1652	1737	1618	56	35	15
CCNG2	-2.124724273	4.08389E-18	down	454	472	456	1289	1098	1930
CCNI	-1.782545894	2.21753E-11	down	2905	3044	2887	9405	4497	8091
CCNK	1.596580438	1.60982E-39	up	1759	1843	1644	460	355	421
CCNL2	-1.192194977	2.07013E-11	down	1674	1769	1674	2879	2126	3374
CCNO	3.78746183	1.25111E-05	up	151	145	116	18	1	3
CCNT1	1.002371095	1.93969E-09	up	1911	1987	1720	800	523	684
CCNT2	-1.155372727	4.80209E-16	down	529	570	529	1006	726	856
CCP110	1.100304926	1.55665E-06	up	1106	1266	1086	347	302	502
CCR1	-9.61464278	3.06746E-14	down	0	0	1	212	217	165
CCR2	-9.383425632	6.58345E-10	down	0	0	0	180	28	64
CCR5	-9.610079557	2.9162E-13	down	0	0	0	152	77	81
CCR6	-3.635182985	0.037135764	down	0	1	1	4	2	12
CCR7	-5.266777184	0.015010563	down	0	0	0	2	5	8
CCRL2	-5.86301774	6.91815E-11	down	0	2	2	45	63	55
CCSAP	1.858050207	1.27639E-30	up	1009	1001	924	225	168	184
CCSER2	-1.797958173	7.20622E-27	down	589	709	630	1460	1447	1848
CCT2	1.61741903	4.51724E-12	up	5219	5766	4882	1318	1346	971
CCT3	1.993123858	2.46444E-33	up	10620	11674	10276	2210	1893	1691
CCT4	1.434357534	9.50415E-19	up	7372	7842	6979	2318	1787	1733
CCT5	3.255840272	1.4103E-58	up	21708	23252	20276	1689	1751	1367
CCT6A	1.906980616	6.70701E-29	up	10668	11653	9956	2535	1737	1866
CCT6B	-4.104643798	0.000123851	down	4	1	3	13	60	21
CCT7	1.033392148	8.47722E-25	up	6354	7037	6177	2303	2135	2340
CCT8	1.664186127	4.04926E-12	up	9991	10752	9642	3060	1962	1820
CD109	1.921310603	7.69159E-23	up	4340	4524	3928	860	584	979
CD14	-7.094744979	2.24988E-64	down	11	15	10	1719	921	878
CD151	-1.66469713	3.72955E-06	down	4134	4638	4240	13420	4924	11677
CD163	-13.87226602	1.63197E-21	down	0	1	0	1411	1610	8706
CD163L1	-1.771918219	0.032845414	down	48	51	55	66	246	40
CD164	-0.852819311	3.69787E-05	down	4211	4259	4189	6211	3844	6406
CD180	-5.80204226	9.5952E-09	down	0	3	2	106	23	74

CD19	2.698159037	0.004796861	up	26	26	22	4	4	0
CD1C	-7.422573451	1.52706E-05	down	0	0	0	38	5	27
CD1D	-6.451912774	1.13774E-07	down	0	0	2	48	45	31
CD1E	-6.8159863	0.000215858	down	0	0	0	25	3	18
CD2	-6.532947121	2.60796E-11	down	2	1	0	82	44	74
CD200	-12.46272907	6.41165E-20	down	0	0	0	1377	315	593
CD200R1	-4.498398191	1.72603E-06	down	2	2	1	21	18	42
CD207	-6.577490417	0.001984963	down	0	0	0	2	26	7
CD209	-8.624142354	4.34236E-16	down	0	3	0	144	384	279
CD22	2.929882208	0.000662224	up	85	137	110	21	9	1
CD226	-3.28305838	1.74512E-05	down	6	2	3	19	27	30
CD24	6.801435171	4.07346E-53	up	1905	2030	1782	10	4	23
CD244	-6.707734409	3.5964E-05	down	0	0	0	10	19	11
CD247	-8.306071972	6.52013E-10	down	0	0	0	39	32	54
CD248	-12.29396344	1.61219E-21	down	0	0	0	510	880	532
CD27	-6.447632228	1.3258E-05	down	0	0	1	36	13	19
CD274	-2.36137931	8.23032E-12	down	35	44	46	205	134	119
CD276	1.594254418	1.38212E-07	up	2708	2935	2849	973	420	642
CD28	-10.23813226	4.53415E-13	down	0	0	0	127	66	293
CD2AP	0.58768495	0.003975766	up	1292	1426	1224	541	565	755
CD300A	-5.749203191	1.66496E-15	down	4	3	1	157	76	76
CD300C	-4.004749499	0.001084159	down	0	2	4	43	7	21
CD300E	-8.464673633	1.01323E-07	down	0	0	0	88	38	14
CD300LB	-6.93911312	0.000130913	down	0	0	0	33	11	5
CD300LF	-7.569192762	8.78351E-07	down	0	0	0	28	33	12
CD300LG	-11.13581225	9.67571E-15	down	0	0	0	94	442	315
CD302	-5.220105835	0.014153453	down	0	0	0	6	2	7
CD320	0.89695051	6.73519E-09	up	1058	1143	1054	408	350	488
CD33	-5.250345213	4.02746E-17	down	2	6	2	119	70	82
CD34	-10.46141492	3.36991E-81	down	4	4	5	3162	3449	6490
CD36	-10.52432346	8.40921E-21	down	6	15	5	509	20191	3876
CD37	-8.345032388	6.75487E-14	down	0	0	2	158	114	195
CD38	-5.666548636	5.17311E-08	down	2	4	9	32	356	123
CD3D	-8.001355461	5.71796E-09	down	0	0	0	39	35	26
CD3E	-9.163537233	4.41213E-12	down	0	0	0	108	59	60
CD3EAP	2.696783073	7.39918E-19	up	573	542	484	53	73	47
CD3G	-5.589722969	0.00256657	down	0	0	0	6	5	8
CD4	-9.832207113	5.03388E-18	down	0	1	1	387	270	656
CD40	-7.414170083	1.77287E-45	down	2	7	3	562	334	570
CD44	1.09749013	0.005585727	up	11123	11919	10893	4274	1813	5456
CD46	1.51047892	3.56395E-11	up	15738	17757	15894	3469	3475	5397
CD47	-0.891798029	0.000251281	down	1427	1539	1390	2395	1268	2165
CD48	-9.117586256	4.13278E-12	down	0	0	0	99	49	73
CD5	-6.233305005	0.000206751	down	0	0	0	13	6	11
CD52	-9.561059901	2.46434E-13	down	0	0	0	138	84	76
CD53	-6.159500335	6.64771E-28	down	3	15	14	625	294	729
CD55	4.539595154	1.0079E-129	up	28514	31668	28765	1039	646	1062
CD59	-0.852963313	1.55788E-20	down	7996	8570	8098	11195	9837	10610
CD6	-4.959063639	2.19928E-07	down	3	1	0	31	24	34
CD63	-0.951537989	1.56212E-06	down	7222	7812	7459	13166	8912	8973
CD68	-1.811005931	2.45773E-09	down	756	824	825	1905	1295	2886
CD69	-8.695874008	3.02791E-08	down	0	0	0	38	96	21
CD7	-5.371222474	2.72305E-07	down	2	0	1	29	32	27
CD72	-8.324596455	9.07161E-10	down	0	0	0	59	32	36
CD74	-12.04775849	2.8351E-131	down	4	5	3	15099	8452	12974
CD79A	-4.54113447	0.00926113	down	0	0	1	7	4	7
CD79B	-2.712286045	5.72937E-07	down	12	23	16	61	109	60
CD80	-6.452546237	5.51026E-05	down	0	0	0	11	13	10
CD81	-2.693330309	3.8392E-170	down	1842	2020	1877	9546	8113	8713
CD83	2.396896775	0.000599606	up	926	1033	899	286	67	43
CD84	-11.19406802	1.55024E-16	down	0	0	0	247	152	540
CD86	-10.52313691	1.55762E-14	down	0	0	0	316	80	199
CD8A	-5.061835279	7.68827E-15	down	4	5	1	109	57	73
CD8B	-6.990412893	1.19938E-05	down	0	0	0	28	9	14
CD93	-14.69984022	4.86511E-32	down	0	0	0	3365	4094	2830
CD96	-8.924952377	2.91545E-09	down	0	0	1	58	232	61
CD99L2	-1.892298814	7.97037E-24	down	619	692	571	1447	1592	1901
CDA	4.739814443	1.75938E-54	up	1539	1798	1656	28	45	59
CDADC1	-0.767862583	0.001241385	down	206	225	225	249	290	248
CDC123	1.225218274	2.52239E-37	up	2506	2800	2497	844	700	830
CDC20	5.549321786	5.34248E-14	up	2591	2870	2691	96	22	9
CDC23	0.716547128	6.78069E-08	up	1225	1287	1127	539	505	526
CDC25A	5.772645094	2.37863E-84	up	1473	1506	1288	24	9	23
CDC25B	1.276522589	1.69531E-17	up	2129	2234	2208	702	508	732
CDC25C	5.28708179	3.02638E-14	up	549	598	558	25	3	4
CDC27	0.662282932	6.56245E-10	up	2819	3037	2756	1290	1186	1389
CDC37L1	-1.061622854	2.92772E-14	down	387	388	389	641	496	597
CDC42BPA	-0.58603359	0.045716854	down	1523	1671	1563	1352	1371	2373
CDC42BPG	3.623636971	7.88541E-10	up	94	132	118	4	3	13
CDC42EP2	1.045291978	0.02997781	up	409	448	375	133	203	76
CDC42EP3	-3.336593275	1.65592E-14	down	301	278	244	3387	1439	1172
CDC45	5.530233176	1.82323E-47	up	945	1030	901	21	17	6
CDC6	5.352656386	1.06531E-26	up	2397	2471	2267	61	48	14
CDC7	2.911420198	2.54969E-08	up	760	819	735	50	120	40
CDC73	0.646312099	1.99987E-06	up	1955	2105	1856	1040	737	922
CDCA2	6.2147937	6.6185E-18	up	2068	2174	1984	26	30	2
CDCA3	4.064941724	1.60511E-10	up	728	838	726	68	21	10
CDCA4	3.463340637	7.33107E-23	up	1448	1416	1336	118	99	52
CDCA5	4.911848966	2.96624E-12	up	1563	1584	1490	76	28	7
CDCA7	5.093010639	4.22667E-13	up	1025	1153	1152	17	44	5
CDCA7L	3.300793508	9.06839E-20	up	1938	2037	1880	84	131	206
CDCA8	5.521616172	2.43912E-96	up	1143	1241	1164	25	16	14
CDCP1	4.488093446	4.77398E-22	up	702	727	785	42	9	21
CDH1	4.171488814	0.000692633	up	230	227	206	0	22	2
CDH11	-13.26678748	1.36465E-26	down	0	0	0	1148	1457	1214
CDH12	6.38793982	1.55388E-05	up	23	21	21	0	0	0
CDH13	-2.377358063	3.8472E-08	down	1441	1449	1411	5193	2446	8652
CDH16	8.189012898	8.5709E-11	up	146	153	143	0	1	0
CDH17	4.455683812	2.1043E-07	up	67	61	56	0	1	5
CDH19	-9.974039925	5.91525E-14	down	2	2	0	127	2175	304
CDH2	-0.993749677	0.027476372	down	803	896	803	827	1746	849

CDH20	-6.702030097	0.000875526	down	0	0	0	2	23	14
CDH23	-6.851332512	2.09607E-15	down	3	3	0	143	239	93
CDH26	-4.394077091	3.4556E-06	down	0	5	1	33	28	27
CDH3	4.179057508	0.002262726	up	73	94	83	0	9	0
CDH5	-7.717391807	4.483E-35	down	4	21	10	1064	2511	1406
CDH6	-10.79127078	7.86978E-18	down	0	0	0	252	242	197
CDH8	-7.767043286	4.74549E-06	down	1	0	2	291	1	207
CDHR1	-6.99982066	5.04714E-05	down	0	0	0	10	29	9
CDIP1	0.783144925	0.006805595	up	107	107	113	42	44	49
CDIPT	-1.040314732	2.77099E-12	down	952	1002	844	1581	1109	1421
CDK1	6.108544805	1.37555E-30	up	9105	9710	8618	186	60	42
CDK12	0.875524222	9.9648E-05	up	1686	1797	1605	550	589	831
CDK15	-9.399727628	1.68421E-13	down	0	0	0	87	74	105
CDK16	1.534595684	0.000504928	up	3647	3856	3621	582	1348	709
CDK18	0.888315163	0.048111399	up	758	905	965	292	480	209
CDK19	-1.420743376	3.2958E-12	down	290	314	250	531	436	664
CDK2	3.32903993	3.7217E-180	up	4071	4428	4110	316	281	294
CDK4	1.682540823	2.01567E-17	up	4391	4638	4134	1157	947	803
CDK5	1.743961244	2.54633E-05	up	456	443	444	92	130	57
CDK5R2	5.496166825	0.001241385	up	10	11	14	0	0	0
CDK5RAP1	0.98115931	6.31679E-07	up	704	792	706	275	279	233
CDK5RAP2	0.691490845	0.010356625	up	2434	2710	2406	1337	697	1340
CDK5RAP3	-0.901643116	9.94345E-07	down	958	1015	959	1579	974	1379
CDK6	3.163792221	5.2331E-116	up	5795	6673	6021	521	391	561
CDK7	1.991548677	5.18901E-05	up	77	76	111	18	19	10
CDK8	0.625311805	0.003732691	up	512	561	470	278	171	268
CDKAL1	0.841542996	3.45005E-05	up	402	364	380	146	136	174
CDKL1	1.245637626	0.011669754	up	157	140	118	22	54	46
CDKL2	-4.708394195	7.45551E-06	down	1	3	1	46	10	39
CDKN1B	-1.115045167	0.000224315	down	1045	1163	1104	1857	1025	2304
CDKN2A	6.998661845	7.26617E-78	up	3286	3569	3243	33	9	15
CDKN2AIP	-1.179387371	7.94982E-10	down	253	286	220	487	332	407
CDKN2B	2.480787044	6.68261E-06	up	945	1029	1024	44	151	180
CDKN2D	-0.605246276	0.02040665	down	84	105	96	124	86	99
CDKN3	2.948519658	0.000311637	up	480	513	461	44	78	6
CDNF	-1.612742856	6.98164E-05	down	17	18	20	42	34	44
CDO1	-12.90640512	2.98751E-22	down	0	0	0	533	1434	952
CDR2L	2.856764044	1.04717E-71	up	2181	2167	2196	237	208	197
CDS2	-2.342148895	1.29906E-14	down	642	644	632	2406	1399	3232
CDT1	6.903077901	1.57373E-37	up	1313	1288	1384	17	5	2
CDYL	1.464871756	1.8402E-24	up	1417	1562	1311	389	302	417
CDYL2	3.117483142	2.35681E-26	up	1715	1826	1690	101	170	151
CEACAM19	-0.64535159	0.039701361	down	49	51	54	58	56	57
CEACAM7	7.10138533	8.89871E-07	up	24	52	32	0	0	0
CEBPD	-2.948097119	1.65747E-12	down	309	258	280	859	1998	1699
CEBPZ	1.082388853	2.54851E-12	up	2348	2631	2241	925	762	731
CECR1	-9.982036286	3.14508E-18	down	0	1	1	283	546	585
CECR5	1.007418271	0.000200923	up	922	1139	941	296	416	331
CECR6	-4.151130028	8.4109E-05	down	2	0	5	50	16	26
CEL	-2.934299449	0.019862532	down	1	2	1	12	4	6
CELF1	1.405399487	8.3092E-42	up	5292	5622	5281	1526	1237	1597
CELF2	-2.78792802	3.44091E-31	down	302	362	340	1317	1550	2042
CELF5	5.687372845	0.000481486	up	13	13	14	0	0	0
CELF6	-5.560489115	3.34004E-15	down	2	1	4	105	59	74
CELSR1	3.505634648	2.58296E-11	up	1391	1641	1471	65	156	49
CELSR2	3.336243321	4.98179E-76	up	1887	2266	2081	181	114	146
CELSR3	4.773477912	6.43343E-25	up	1134	1162	1141	22	14	55
CENPA	6.233308921	1.51127E-73	up	963	1006	879	14	6	7
CENPC	-1.083334727	6.98553E-06	down	306	376	304	631	348	520
CENPE	4.033156821	7.17246E-96	up	1354	1419	1287	74	47	56
CENPF	7.408940426	6.39275E-53	up	10784	11550	10566	65	55	16
CENPH	3.690896833	2.71988E-37	up	846	878	768	52	53	31
CENPI	4.075840232	1.16035E-67	up	770	829	737	33	37	28
CENPJ	1.67492727	1.92713E-18	up	803	853	810	192	138	223
CENPK	4.592077906	7.41544E-35	up	934	996	855	32	35	14
CENPL	2.79593083	3.01054E-40	up	465	483	452	48	49	46
CENPM	3.528694364	6.34148E-05	up	291	290	248	44	5	4
CENPN	3.961424447	2.02953E-26	up	1760	1981	1842	81	116	52
CENPO	2.984599241	3.77771E-18	up	917	1021	954	69	117	68
CENPP	0.923512703	0.003842842	up	126	107	143	48	40	54
CENPQ	1.559372922	5.20037E-20	up	525	516	489	128	117	124
CENPT	-0.6410604	0.007301961	down	548	607	592	794	443	725
CENPU	1.60183459	0.00038908	up	168	182	164	68	27	27
CENPV	2.585199932	9.51856E-23	up	864	879	779	140	78	83
CENPW	3.479732925	3.19576E-30	up	551	632	523	50	20	40
CEP112	-2.907214705	1.22969E-24	down	86	80	75	337	460	477
CEP120	-0.929927773	0.000118202	down	466	477	380	682	416	713
CEP126	-1.169881977	0.01311577	down	266	275	221	466	173	611
CEP128	2.035086747	3.98379E-24	up	361	428	409	70	60	78
CEP131	0.970405095	4.76404E-05	up	371	422	369	168	139	113
CEP135	1.088005777	6.55181E-11	up	512	570	509	177	150	206
CEP152	2.425999409	2.91347E-18	up	832	777	836	86	91	148
CEP162	-0.712689297	0.003106912	down	209	265	217	332	194	285
CEP170	1.780261307	3.43306E-05	up	266	213	119	37	42	44
CEP170B	1.289296369	1.72007E-05	up	1634	1774	1587	338	474	632
CEP250	1.854289413	1.15512E-09	up	1587	1748	1656	234	288	459
CEP295	2.524744892	1.51127E-47	up	2584	2959	2484	337	257	402
CEP41	-1.435016223	1.15933E-05	down	81	78	77	160	174	115
CEP44	-0.59224104	0.000399433	down	335	340	335	401	292	396
CEP55	5.439933741	2.8436E-11	up	2756	3206	2848	120	20	9
CEP57	1.550841781	7.49046E-39	up	2508	2762	2401	704	547	612
CEP57L1	0.661047033	0.001757315	up	433	470	384	231	178	169
CEP63	-1.235211044	4.61198E-13	down	305	372	318	653	493	519
CEP68	-2.217549951	9.15798E-26	down	272	304	306	864	847	1210
CEP70	0.623633177	0.000648259	up	784	869	749	383	288	443
CEP72	4.555356699	1.63994E-75	up	1118	1211	1093	40	38	25
CEP76	1.093419337	2.24189E-07	up	236	280	238	83	84	83
CEP78	0.801705023	5.45201E-06	up	600	591	559	252	190	276
CEP85	2.607512538	1.76813E-06	up	1007	1097	953	75	200	66
CEP85L	-1.774090922	9.98098E-19	down	96	94	101	234	219	255
CEP97	0.919771722	3.51402E-09	up	522	576	535	203	199	210

CERK	-0.639467799	2.76972E-06	down	869	997	923	1136	968	979
CERS2	0.855667561	0.000346203	up	5105	5353	5021	2594	1362	2214
CERS4	-2.672276556	1.05738E-22	down	53	44	46	269	185	198
CERS5	-0.948916739	0.009931864	down	468	532	434	1034	449	511
CES1	-11.66876014	1.88878E-07	down	0	0	0	39	847	309
CES4A	-7.339331312	1.77728E-09	down	0	2	0	55	56	119
CFAP20	0.802620369	0.004078159	up	657	710	704	277	331	225
CFAP221	-3.031642207	6.59611E-05	down	10	2	12	52	56	32
CFAP36	-0.628788636	0.001695881	down	622	704	689	902	552	782
CFAP45	1.95846309	0.003846339	up	58	67	52	16	13	3
CFAP61	-6.464773245	0.000464017	down	0	1	0	4	47	12
CFAP69	-3.764864508	8.82572E-16	down	22	14	9	167	93	182
CFAP70	-1.143558903	0.001272082	down	28	45	40	67	54	56
CFAP97	-1.552667391	1.53083E-10	down	554	583	523	1117	838	1539
CFH	-9.795094453	5.76444E-92	down	11	13	16	6191	12097	6234
CFHR1	-2.584942682	0.048897864	down	0	3	3	4	10	11
CFI	-9.459167499	1.00174E-24	down	1	3	0	347	907	675
CFL1	1.018847337	4.6563E-20	up	14718	15919	14613	6074	4480	5382
CFL2	-1.536208098	2.17458E-29	down	1449	1684	1311	3025	2745	3371
CFLAR	-3.216329912	1.77288E-24	down	525	626	527	2451	3674	4862
CFP	-4.089714193	5.69855E-07	down	7	12	4	109	23	153
CGA	11.10385278	1.64353E-19	up	533	613	567	0	0	0
CGB8	5.620527486	0.000873566	up	16	10	12	0	0	0
CGGBP1	-1.829706602	2.74997E-54	down	938	1055	956	2652	2070	2756
CGN	7.984084676	6.18547E-10	up	96	152	137	0	1	0
CGNL1	-5.460182673	2.07256E-47	down	35	50	48	2145	1039	1019
CGREF1	-2.023992505	0.011276953	down	26	19	14	66	84	16
CGRRF1	-3.014986131	1.45305E-20	down	51	57	60	458	224	297
CH17-360D5.1	4.030914336	4.48117E-06	up	54	50	34	4	2	0
CH25H	-7.108604122	1.0461E-05	down	0	0	1	31	60	10
CHAC2	3.404952701	8.62779E-20	up	406	432	397	15	32	35
CHADL	-5.189880524	2.26037E-10	down	3	1	3	79	69	33
CHAF1A	3.318523328	8.26376E-22	up	1610	1625	1612	97	156	85
CHAF1B	4.24511794	4.90576E-69	up	742	740	730	33	27	23
CHAMP1	1.623472545	1.24827E-27	up	1173	1337	1176	277	245	329
CHCHD1	0.921971395	0.005537353	up	781	780	753	415	258	199
CHCHD2	0.907510544	0.001141932	up	7097	7815	6923	3971	1996	2405
CHCHD4	0.997871988	0.004749536	up	429	467	424	115	202	143
CHD1	1.100012443	1.19118E-19	up	2060	2188	2037	764	650	669
CHD4	1.58159838	5.88534E-41	up	10484	11115	10395	2619	2474	2481
CHD7	2.632497633	2.92294E-07	up	1624	1741	1548	83	284	176
CHDH	1.175813246	0.020122365	up	168	192	161	47	81	31
CHEK1	4.386529472	2.6047E-163	up	2187	2326	2152	88	65	74
CHEK2	2.985285214	1.0451E-75	up	1306	1440	1264	131	118	110
CHERP	1.050691977	5.69418E-19	up	1070	1187	1091	413	331	407
CHFR	-1.827774759	6.74948E-20	down	114	141	108	343	285	285
CHIC1	-0.860731981	0.005756489	down	311	361	312	526	251	514
CHIC2	-1.812498516	3.94926E-15	down	110	134	116	273	319	298
CHIT1	-6.322166728	0.007861341	down	0	0	0	4	24	1
CHKA	1.668956219	2.52934E-06	up	1289	1216	1147	175	336	291
CHL1	-5.545719556	1.72839E-10	down	14	14	15	83	935	323
CHML	2.48861671	8.98917E-12	up	2932	3060	2582	367	192	548
CHMP1A	0.912110753	4.54218E-09	up	1818	2011	1803	826	550	766
CHMP1B	-1.107811629	7.0845E-12	down	635	811	716	1058	990	1258
CHMP2A	-1.098138731	1.85797E-08	down	844	736	684	1340	1066	1046
CHMP4A	-0.962442765	2.24976E-10	down	601	674	684	1043	750	935
CHMP6	-2.01448429	3.20026E-16	down	76	86	86	272	236	202
CHN1	-3.127833297	5.54516E-19	down	51	75	35	336	242	423
CHN2	-3.96032221	1.44543E-13	down	23	22	16	142	147	393
CHODL	-5.799724749	9.82213E-06	down	1	0	3	125	25	16
CHORDC1	2.502759387	2.2828E-09	up	4531	4338	3639	900	293	412
CHPF	-1.683805687	5.8474E-17	down	581	673	626	1653	1025	1660
CHPF2	1.699074754	9.20945E-11	up	2205	2214	2200	395	398	663
CHPT1	-1.042523128	5.41169E-05	down	697	800	649	906	1217	967
CHRAC1	0.822058344	0.003061502	up	1152	1218	1125	559	291	578
CHRD	-3.520434111	7.50212E-20	down	23	51	44	358	344	247
CHRDL1	-14.10034033	1.02774E-26	down	0	0	0	1150	2978	2598
CHRDL2	-4.221390282	0.027691316	down	6	1	8	9	175	0
CHRM1	6.384796678	6.95E-05	up	12	35	19	0	0	0
CHRM4	5.592895475	8.83402E-05	up	25	24	24	0	0	1
CHRNA4	6.141930725	6.00695E-11	up	91	107	99	1	0	2
CHRNA5	5.402134596	1.69864E-51	up	451	529	450	9	8	7
CHRNA6	5.169090056	0.00393412	up	8	10	10	0	0	0
CHRNB1	-1.179789565	4.70371E-05	down	72	73	93	125	134	123
CHRNE	-3.700025042	0.00175581	down	0	3	1	10	14	12
CHST1	-10.62094141	1.29979E-17	down	0	0	0	214	202	200
CHST10	-2.201773698	6.07031E-14	down	40	34	44	154	107	128
CHST13	5.324507716	6.46538E-10	up	248	234	228	2	10	0
CHST2	1.292940027	0.028900082	up	276	292	333	49	146	56
CHST3	1.53556582	5.39375E-13	up	2775	2981	2786	610	590	900
CHST6	1.467205785	8.27118E-05	up	210	190	204	53	30	75
CHST8	-4.734530912	0.00045271	down	0	3	0	13	26	15
CHSY1	-0.615289047	0.001532601	down	761	860	756	791	913	860
CHSY3	-4.158987969	1.32551E-11	down	22	33	24	303	113	612
CHTF18	3.056046201	1.40054E-63	up	957	1006	998	102	72	80
CHTOP	0.864949822	1.14714E-07	up	1948	2057	2001	955	641	763
CHUK	0.635486603	6.83891E-06	up	1058	1145	1027	541	398	546
CHURC1	-1.031401894	4.06987E-09	down	1240	1340	1204	1824	1460	2244
CIAO1	0.691408775	5.26218E-05	up	2587	2822	2377	1074	1177	1149
CIAPIN1	1.421599858	6.01355E-10	up	1284	1469	1239	330	401	314
CIART	-5.233973165	1.25039E-07	down	1	4	0	27	56	46
CIB1	1.031812354	4.88779E-06	up	2968	3134	2841	1145	1108	831
CIDEA	-11.80951	3.52911E-05	down	0	0	0	7	1072	215
CIDEB	-0.879740878	0.013136654	down	102	119	98	101	125	190
CIDEC	-10.34647733	6.46644E-09	down	1	1	8	122	7659	818
CIITA	-5.524785297	3.25378E-52	down	9	11	11	333	268	419
CILP	-10.67010731	5.3268E-13	down	0	1	1	59	663	1577
CIPC	-0.726175676	0.000171171	down	467	451	475	500	502	638
CIR1	-1.788212125	1.50204E-09	down	351	384	313	1231	570	808
CIRBP	-4.152148772	7.8553E-115	down	365	507	399	6188	4166	5769
CISD1	0.884022397	0.044445238	up	1057	1188	1037	361	603	262

CIT	3.688413649	2.31927E-30	up	998	1115	1047	81	57	36
CITED4	2.110218847	2.39994E-12	up	664	671	677	157	68	112
CIZ1	0.740159554	3.70932E-05	up	2230	2487	2314	925	853	1220
CKAP2	2.48413055	1.9214E-20	up	1751	1791	1599	262	231	156
CKAP2L	5.447339874	2.3335E-09	up	1368	1424	1191	50	15	1
CKAP4	1.157401267	1.67223E-13	up	3251	3307	3352	1273	882	1023
CKAP5	2.353116938	6.02545E-40	up	7833	8410	7641	1005	957	1366
CKB	1.949418975	2.68898E-15	up	7631	8161	7806	1905	1337	1107
CKS1B	2.397672836	2.41515E-31	up	1739	1834	1711	305	209	201
CKS2	5.316280429	2.14116E-22	up	5174	5416	4838	176	68	35
CLASP2	-0.703871899	0.00370251	down	1067	1074	945	1155	881	1569
CLCA2	-6.613364098	0.00032145	down	0	0	0	4	20	13
CLCF1	1.10424684	0.008588993	up	128	119	130	52	47	25
CLCN2	1.422302442	5.83203E-05	up	158	209	160	38	35	67
CLCN5	0.997655845	4.14072E-11	up	596	617	552	209	194	225
CLCN7	1.03998817	4.3528E-05	up	1744	1839	1917	520	722	637
CLDN1	2.865933718	2.33995E-06	up	672	898	782	153	24	60
CLDN10	-6.241646888	0.000153256	down	0	0	0	11	7	12
CLDN11	-5.91387938	4.80822E-17	down	2	4	3	80	108	197
CLDN12	0.816127889	0.001091834	up	1238	1403	1143	681	357	506
CLDN15	-1.291501258	0.017310485	down	104	78	93	84	233	149
CLDN16	4.079011916	0.029669065	up	3	14	9	0	0	1
CLDN4	9.064783814	1.59743E-40	up	1224	1300	1236	2	1	2
CLDN5	-12.18094817	1.74291E-20	down	0	0	0	459	868	441
CLDN7	3.141592252	8.17093E-35	up	415	427	468	33	29	44
CLDN9	4.570422012	5.27722E-05	up	31	36	32	3	0	0
CLDND1	-1.412865172	1.63448E-11	down	905	907	980	2082	1265	2009
CLEC10A	-10.59641247	1.45321E-14	down	0	0	0	321	77	229
CLEC11A	-11.11935401	2.09863E-18	down	0	0	0	338	314	214
CLEC12A	-9.346737851	1.08278E-11	down	0	0	0	47	83	124
CLEC14A	-10.81818756	2.08089E-21	down	0	0	2	710	1155	644
CLEC16A	0.982133764	3.1535E-05	up	855	842	812	247	281	374
CLEC18A	-5.382583437	6.65373E-07	down	1	3	3	117	10	89
CLEC18B	-3.624531251	0.009833569	down	0	7	2	56	5	19
CLEC19A	7.140328059	4.46991E-08	up	74	78	62	1	0	0
CLEC1A	-10.1235754	2.6167E-15	down	0	0	0	114	141	181
CLEC2D	-3.813513158	1.49446E-31	down	19	19	12	174	138	189
CLEC3B	-12.90217337	8.247E-23	down	1	0	0	3305	965	1738
CLEC4A	-4.721131677	5.41972E-10	down	8	2	3	136	42	74
CLEC4E	-8.540195787	1.04611E-06	down	0	0	0	22	11	118
CLEC4F	-7.870451307	2.06484E-07	down	0	0	0	14	38	38
CLEC5A	-9.640682061	1.38905E-11	down	0	0	0	198	63	59
CLEC7A	-6.886078458	1.7986E-17	down	1	3	2	278	85	152
CLEC9A	-6.260356165	0.000718934	down	0	0	0	19	4	8
CLECL1	-6.524745061	0.001060907	down	0	0	0	27	7	3
CLIC2	-2.420028881	2.68596E-35	down	132	136	132	499	524	490
CLIC3	2.794993991	3.82614E-14	up	368	425	455	67	25	38
CLIC4	-1.733471032	1.47764E-08	down	6846	7666	6966	19665	9669	22385
CLIC6	-8.237633076	5.00115E-09	down	0	0	0	57	21	43
CLINT1	0.811757996	1.78482E-07	up	2605	2672	2546	1006	923	1246
CLIP2	0.750947938	0.024578363	up	1117	1239	1232	500	309	731
CLIP3	-5.562204109	6.88484E-35	down	19	16	14	676	275	735
CLIP4	-1.647068247	5.04815E-06	down	261	307	260	406	534	900
CLK1	-2.365833093	1.19815E-10	down	1295	1287	1116	5760	2224	5897
CLK3	0.597863735	0.000139383	up	1207	1273	1237	696	479	581
CLK4	-2.697428131	7.82048E-21	down	174	188	169	1018	497	975
CLMN	-2.445640892	9.60821E-06	down	138	158	148	222	815	627
CLN3	0.856174938	7.89793E-06	up	756	855	819	329	333	286
CLN5	-2.165327545	8.04048E-41	down	257	296	221	905	687	880
CLN6	0.902761163	1.83615E-05	up	484	533	499	193	207	172
CLNS1A	1.566189122	2.34681E-45	up	2551	2820	2473	696	566	621
CLOCK	-1.397378813	2.28321E-06	down	346	457	440	587	708	1025
CLP1	1.360092441	3.60271E-13	up	468	581	484	169	119	137
CLPB	2.183251756	2.63615E-08	up	790	789	831	87	176	105
CLPP	0.933259008	0.00134598	up	845	965	868	351	387	245
CLPTM1L	2.905287541	2.47221E-24	up	2828	3001	2413	351	246	184
CLSPN	5.337837882	1.09328E-37	up	1012	1083	932	27	19	7
CLSTN1	0.679698674	0.001955157	up	4504	4647	4624	2109	1481	2591
CLSTN2	-6.568298162	1.76149E-47	down	6	13	13	505	664	978
CLTC	0.924707326	7.42405E-21	up	13851	15095	13796	5526	4605	5910
CLTCL1	2.373507265	6.28588E-12	up	1189	1337	1209	104	187	215
CLUAP1	-0.676844981	0.003472136	down	376	432	343	455	319	543
CLUH	1.343984356	0.023025575	up	2787	2912	2929	426	1390	488
CLYBL	-0.82403138	0.002334083	down	133	151	122	199	172	137
CMA1	-6.657884537	0.000522773	down	0	0	0	26	11	3
CMBL	1.904794108	4.13341E-22	up	3200	3420	3164	687	643	516
CMC1	0.721670162	0.034264092	up	554	614	564	198	319	214
CMC2	1.999186292	8.15349E-12	up	1623	1844	1681	366	333	208
CMC4	-1.537712093	0.000463465	down	48	55	46	147	98	62
CMIP	1.061272517	0.000449476	up	1482	1655	1579	489	665	423
CMKLR1	-11.68074641	1.24006E-20	down	0	0	1	853	573	1114
CMPK2	-9.170293494	2.3067E-10	down	1	0	0	293	96	60
CMSS1	1.060200618	1.7037E-07	up	908	954	855	264	297	362
CMTM1	2.15535157	0.000567563	up	34	36	36	4	4	9
CMTM3	-6.992035489	1.18704E-41	down	2	9	8	754	551	404
CMTM4	1.496481597	1.61061E-06	up	1212	1249	1079	222	368	285
CMTM5	-8.110391389	6.30519E-07	down	0	0	0	78	15	19
CMTM7	-8.933366902	3.46271E-12	down	0	0	1	100	135	135
CMTM8	-8.727676528	9.10283E-08	down	0	0	0	42	101	15
CMTR1	-0.631741131	0.000105295	down	806	853	755	842	779	1040
CNDP2	-1.257670657	0.000772278	down	593	644	680	782	1459	939
CNEP1R1	-0.724582373	0.02050052	down	237	263	186	373	192	248
CNGA1	-4.383509	0.005582933	down	2	0	2	53	6	4
CNGB1	9.060081229	1.30415E-12	up	139	167	111	0	0	0
CNIH3	-2.504095048	0.000156974	down	8	8	6	48	19	23
CNKSR1	2.906023809	5.18111E-16	up	141	172	179	19	10	18
CNKSR2	-9.387255061	7.75141E-09	down	0	0	0	167	13	95
CNN1	-13.02482421	1.2808E-35	down	1	3	0	12779	1657	9943
CNN3	-1.165121962	1.23215E-12	down	2640	2738	2454	4061	3441	5035
CNNM1	-5.798683258	0.001464443	down	0	0	1	10	3	31
CNNM3	1.231298078	8.4965E-05	up	1088	1105	1122	244	289	473

CNNM4	2.033606538	4.88206E-44	up	923	994	924	186	136	173
CNOT1	1.212006657	7.96426E-10	up	5715	5951	5563	1519	1602	2163
CNOT10	0.961022837	4.21314E-12	up	776	839	781	297	282	294
CNOT11	1.156760536	1.92001E-09	up	1455	1598	1521	557	485	411
CNOT6L	-0.849764428	1.371E-05	down	665	644	564	715	765	913
CNOT7	1.195395235	1.21751E-15	up	3355	3919	3342	1319	919	1067
CNOT8	0.682882124	0.000269841	up	1987	2035	1974	1099	676	906
CNOT9	0.886505141	1.61628E-09	up	1263	1436	1284	590	466	475
CNPPD1	-1.006128403	9.63721E-05	down	592	528	510	675	884	743
CNPY4	-0.67124444	0.008772789	down	308	316	310	445	240	387
CNR1	-3.69790348	3.0026E-05	down	49	65	56	256	84	1283
CNRIP1	-11.40577643	8.47094E-20	down	0	0	1	870	461	773
CNTD2	4.132095001	9.46954E-08	up	52	77	70	0	3	5
CNTFR	-1.698400453	0.00817571	down	143	179	159	142	626	288
CNTN1	-9.275967101	6.07131E-12	down	1	4	0	1127	27	1104
CNTN2	-7.116146554	2.4679E-06	down	0	0	0	22	20	12
CNTN3	-11.2235145	1.37073E-11	down	0	0	0	396	22	565
CNTN4	-12.75528355	1.2399E-09	down	0	0	0	1247	73	1523
CNTN6	-7.046367582	0.014693687	down	0	0	0	50	0	5
CNTNAP3	-1.435244733	8.02668E-05	down	48	42	56	100	64	121
CNTNAP3B	-2.897273845	6.60872E-05	down	14	16	9	37	37	135
COA3	-0.619091938	0.025676238	down	495	620	473	746	538	444
COA4	0.97204894	4.55216E-05	up	1254	1331	1263	582	447	364
COA5	-1.285934561	2.45954E-05	down	334	430	363	940	440	597
COA6	3.154343365	3.16278E-29	up	2691	2844	2560	210	263	163
COA7	2.2525588	2.79137E-10	up	1142	1175	993	139	222	121
COASY	1.915309789	1.20939E-13	up	2395	2600	2408	398	555	420
COBLL1	-2.012854001	6.09156E-13	down	328	335	305	867	646	1295
COCH	-10.83987137	9.34376E-11	down	0	0	0	627	42	86
COG1	-0.962198282	3.95866E-11	down	317	327	314	442	406	479
COG2	0.670785615	0.000494408	up	804	868	762	374	380	325
COG3	-0.818062943	4.37408E-07	down	491	478	413	629	478	633
COG7	0.752812753	1.73808E-06	up	508	567	480	217	209	228
COIL	0.986140924	1.93987E-05	up	853	975	739	374	216	339
COL10A1	-5.661421364	0.002132333	down	0	0	0	6	5	9
COL13A1	-4.006955856	1.94226E-07	down	34	38	30	483	59	671
COL14A1	-12.05746944	2.5582E-64	down	8	6	3	23485	4559	25364
COL15A1	-1.587895983	0.004068158	down	1387	1751	1531	2274	5515	1734
COL16A1	-2.926526065	5.25916E-18	down	292	428	368	2394	1079	2506
COL17A1	3.256284838	0.000530109	up	93	68	93	3	14	1
COL18A1	-4.497696233	3.26493E-36	down	469	499	476	12248	4375	7089
COL19A1	-5.265889168	0.044901799	down	0	1	0	0	23	4
COL1A1	-4.775594969	5.90357E-09	down	893	904	925	7432	37703	4191
COL1A2	-17.93490426	1.27532E-45	down	0	0	0	31863	41717	22772
COL21A1	-10.77991496	1.36465E-26	down	0	0	4	1999	564	2651
COL23A1	-7.83804684	1.35374E-05	down	1	0	0	15	134	12
COL24A1	-3.459343519	4.83734E-08	down	6	13	20	58	128	113
COL27A1	-3.059488376	3.85424E-13	down	133	157	128	1411	501	610
COL28A1	-4.113755322	2.30479E-15	down	6	15	9	87	109	170
COL2A1	10.72579133	3.63271E-18	up	441	434	440	0	0	0
COL3A1	-11.19536573	1.46828E-86	down	16	16	19	20413	46638	14587
COL4A1	-2.073100386	6.0717E-05	down	4876	5150	4959	8615	24372	9963
COL4A2	-0.88561391	0.006115154	down	9472	10354	9557	9267	15970	12569
COL4A3	-1.686605253	1.1774E-05	down	260	269	274	989	367	521
COL4A3BP	-1.280834651	1.22968E-11	down	646	774	664	1067	1235	1265
COL4A4	-0.972254724	0.00994432	down	533	683	712	912	1134	590
COL5A1	1.504366537	0.004889863	up	12802	14458	13916	2068	5666	2138
COL5A2	-8.816865518	4.5357E-125	down	10	16	13	3195	4552	4577
COL5A3	-4.708423663	4.77837E-09	down	80	92	100	333	3341	1064
COL6A1	-3.085791563	1.651E-107	down	2020	2200	2095	12009	11464	14641
COL6A2	-7.083002171	6.0371E-189	down	205	197	241	25528	14433	23034
COL6A3	-14.9878558	5.52191E-25	down	0	1	0	2503	15773	5097
COL6A6	-9.803583123	1.74695E-08	down	0	0	0	58	251	17
COL7A1	4.806871379	1.88057E-16	up	12556	14794	14231	490	85	521
COL8A1	-6.696876339	2.19898E-22	down	36	45	39	1038	1462	6496
COL8A2	-5.541723629	2.38817E-12	down	7	32	15	587	139	1092
COL9A2	-1.664024617	0.046824724	down	115	118	114	455	33	331
COL9A3	2.171053481	2.04193E-05	up	404	472	417	91	83	27
COLCA1	-8.01601745	1.75452E-08	down	0	0	0	24	27	51
COLCA2	-3.133667209	1.56497E-05	down	4	6	12	37	27	76
COLEC12	-12.29198165	6.64302E-23	down	0	0	0	539	702	708
COLGALT1	1.567415852	1.34285E-31	up	5306	5943	5416	1257	1276	1450
COLGALT2	-8.058222668	4.36199E-12	down	1	1	0	181	71	134
COLQ	-4.864108028	3.59214E-17	down	4	10	4	88	149	126
COMMD1	-1.905779163	6.4216E-13	down	118	111	141	390	321	276
COMMD10	-1.279925926	7.69436E-09	down	225	265	239	528	372	360
COMMD3	-2.225355447	7.96445E-10	down	29	48	45	183	103	122
COMMD5	-0.648424077	0.002446963	down	285	341	315	440	289	324
COMMD6	-1.325637618	0.000628293	down	855	815	771	2383	965	1081
COMMD9	0.79875014	0.000756543	up	900	977	868	376	419	314
COMP	-7.30365663	2.49999E-30	down	9	14	18	977	2625	793
COMTD1	0.831395666	0.02654069	up	352	428	316	224	109	108
COPA	1.166035074	6.76842E-11	up	8577	9366	8501	2491	2497	3386
COPB1	1.03766783	3.38062E-27	up	5366	5904	5061	1979	1659	2020
COPB2	1.50167949	2.16935E-24	up	8500	9246	8332	2074	1901	2579
COPG1	1.673734382	7.6374E-31	up	5806	6518	5858	1263	1343	1422
COPS3	1.05435866	2.72194E-05	up	1998	2248	1930	785	768	539
COPS4	-0.815838069	2.95164E-07	down	673	660	635	976	693	807
COPS8	-0.883162949	6.03969E-11	down	848	985	814	1272	1080	1109
COPS9	-1.149821365	0.000218495	down	363	420	312	750	564	404
COPZ1	1.198573485	4.8641E-10	up	3705	4189	3569	1378	1164	992
COPZ2	-3.028750479	4.30681E-47	down	151	141	122	969	617	837
COQ10B	-0.982565196	8.88039E-08	down	445	484	437	785	497	651
COQ3	0.825529115	0.028624766	up	369	434	358	155	198	103
COQ4	0.653269347	0.008058237	up	662	778	628	356	324	247
CORO1A	-7.217099458	4.4575E-28	down	3	3	7	761	220	438
CORO2A	3.820781107	4.78581E-24	up	1323	1429	1307	55	40	112
CORO2B	-10.64199219	1.42617E-16	down	0	0	0	161	255	200
CORO6	-0.929222773	0.000252835	down	303	349	371	537	317	549
COTL1	2.321784765	2.19325E-08	up	6317	6518	6211	1527	677	540
COX1	-1.760435742	4.65201E-10	down	157706	181351	156106	363389	488151	317413

COX14	-0.83665311	0.018336415	down	470	553	480	778	709	399
COX18	-0.775945059	0.037096771	down	191	190	184	170	295	209
COX2	-1.182226018	1.48619E-12	down	78721	91228	77923	121447	136521	138380
COX20	-0.731150083	6.99588E-06	down	832	824	778	1106	890	878
COX3	-1.707802077	1.6701E-24	down	70908	80708	71087	158561	179334	174567
COX4I2	-8.626323851	7.25181E-09	down	0	0	0	22	76	52
COX6B2	6.466122157	2.38515E-06	up	40	60	35	0	1	0
COX7A1	-13.58405671	1.2981E-25	down	0	0	0	1669	2107	938
COX7A2	-0.63938605	0.022448987	down	1739	1880	1796	2314	2173	1457
CP	-4.999101683	2.37328E-34	down	64	81	69	925	2078	1738
CPA1	-5.417257604	0.0089098	down	0	0	0	10	4	3
CPA3	-3.637298801	2.63667E-09	down	25	28	18	381	76	190
CPA4	7.208085783	2.17934E-36	up	567	575	531	1	6	1
CPAMD8	-3.004443971	6.30117E-12	down	45	50	53	324	135	406
CPB1	-7.898658888	6.57067E-07	down	0	0	0	50	9	38
CPD	0.992697805	0.000339594	up	4579	4696	4539	1241	1886	1741
CPE	-12.03796214	3.74165E-37	down	1	11	1	20216	2809	16182
CPEB2	-1.446811652	0.000572568	down	282	365	310	999	319	577
CPEB4	-1.813260696	5.59946E-09	down	349	352	319	632	1038	832
CPED1	-3.47622984	4.76738E-13	down	293	306	280	2277	934	3935
CPLX1	-0.848884341	0.011329294	down	86	67	82	107	109	84
CPNE4	-8.884164212	1.39327E-10	down	0	0	0	98	50	39
CPNE5	-6.628167036	8.06632E-07	down	1	2	3	41	333	13
CPNE6	-7.176338978	0.000235173	down	0	0	0	43	12	3
CPNE7	7.202953901	8.66352E-48	up	928	936	996	10	0	4
CPO	-6.12074387	1.11861E-05	down	0	2	0	32	13	55
CPOX	0.606943132	0.000651556	up	701	672	613	293	285	349
CPPED1	-1.077118959	1.95667E-10	down	431	424	382	623	516	724
CPQ	-4.577357546	2.10197E-52	down	78	83	73	1666	802	1572
CPS1	6.367741386	4.32212E-62	up	15282	17390	15446	108	199	95
CPSF2	1.473086637	5.14845E-36	up	2829	2915	2722	717	662	789
CPSF3	1.221441451	2.69607E-13	up	1157	1315	1171	426	353	329
CPSF3L	-0.801456768	0.00337225	down	747	684	691	1113	568	995
CPSF4	0.950920614	3.57365E-11	up	772	887	723	322	263	290
CPSF6	0.657195828	0.00025727	up	2360	2474	2345	1071	860	1325
CPT1A	1.694226491	8.82441E-38	up	4349	5011	4530	1082	825	1157
CPVL	-1.331115347	0.003007915	down	240	295	259	264	402	752
CPXM1	-10.30205461	8.53613E-12	down	0	0	0	86	310	72
CPXM2	-14.43186118	1.68596E-29	down	0	0	0	3063	1568	4220
CR1	-9.008333825	1.04833E-10	down	0	1	0	56	140	193
CRABP1	-5.921512303	0.01749411	down	0	0	0	22	1	2
CRABP2	1.626210833	0.001189337	up	478	461	551	200	90	58
CRACR2A	-6.448010157	0.0232917	down	0	0	0	0	17	16
CRACR2B	-2.731732619	4.56998E-09	down	27	29	19	97	158	90
CRADD	-0.97954994	0.001256273	down	119	201	128	194	220	204
CRAT	-0.709635492	0.041814628	down	686	779	678	628	1071	734
CRB1	3.295867468	0.000200923	up	47	70	40	0	6	5
CRB2	4.439783967	1.33263E-07	up	44	53	54	3	0	2
CRB3	5.756892672	6.84635E-14	up	128	108	141	3	1	1
CRBN	-1.852353992	1.64582E-21	down	472	430	403	1366	871	1145
CRCP	0.825379805	4.62242E-06	up	898	946	863	330	325	431
CREB1	-1.192795549	9.52183E-11	down	593	566	567	1038	712	1083
CREB3	-0.798724598	0.000195934	down	525	531	562	734	488	802
CREB3L1	-1.95060983	5.51026E-05	down	53	56	43	112	200	93
CREB3L2	1.217868916	0.001334266	up	6394	6811	6632	2487	987	2739
CREB3L3	9.199658789	1.18659E-12	up	119	145	192	0	0	0
CREB5	-5.421948832	1.37482E-14	down	24	15	22	1179	145	629
CREBL2	-0.930696069	2.87225E-06	down	1167	1284	1105	1412	1425	1978
CREBRF	-2.746904572	1.25725E-19	down	236	282	263	1161	823	1787
CREBZF	0.706701995	0.021781522	up	3416	3889	3103	1513	958	2119
CREG1	-2.068444812	2.93934E-75	down	1141	1291	1114	3730	2974	3894
CREG2	8.803760719	3.6909E-23	up	620	736	543	0	3	0
CRELD1	-3.097087418	2.13693E-37	down	171	133	157	1090	688	1068
CREM	1.135267992	4.48828E-07	up	1412	1406	1327	547	434	359
CRHBP	-9.975851547	4.2593E-15	down	0	0	0	129	101	168
CRHR2	-6.403028005	0.006499863	down	0	0	0	1	4	29
CRIP2	-2.909629212	2.1445E-113	down	565	632	551	3019	2865	3435
CRIP3	-6.267430414	0.00057848	down	0	0	0	19	5	7
CRIPAK	-1.63669252	3.93334E-05	down	76	49	65	183	86	161
CRISPLD1	-4.187994929	2.8604E-08	down	191	218	179	4481	341	3139
CRISPLD2	-2.153503571	2.63679E-05	down	371	384	391	1224	454	2057
CRKL	0.897313303	2.56168E-08	up	2239	2313	2272	796	819	986
CRLF1	-1.878772051	1.01026E-43	down	368	432	414	1064	995	1108
CRLF3	0.832399641	9.21153E-05	up	558	641	510	219	182	283
CRLS1	-1.700209014	0.00296914	down	423	493	452	646	1787	580
CRMP1	1.9895511	0.011248592	up	200	202	234	10	72	26
CROCC	-1.086963097	4.84127E-09	down	269	310	273	481	327	487
CROT	-1.860265486	7.37483E-07	down	104	147	130	402	175	424
CRTAC1	-9.314500397	1.01359E-10	down	0	0	0	157	39	61
CRTAM	-6.996168266	7.18161E-05	down	0	0	0	27	4	21
CRTAP	-0.893150576	0.008202667	down	6185	6941	6321	10972	4625	10619
CRTC3	1.018280457	0.000266366	up	3432	3544	3406	1292	773	1636
CRY1	1.038304833	2.71824E-06	up	902	1045	860	342	232	405
CRY2	-2.349121099	2.69427E-39	down	197	223	179	852	648	669
CRYAB	-6.838049647	9.9938E-46	down	55	84	81	4921	8993	3320
CRYBB1	-7.68365099	9.53766E-08	down	0	0	0	40	17	25
CRYBG3	-1.324601702	1.92728E-05	down	615	622	663	781	1200	1374
CRYGN	-4.609268381	0.014460034	down	0	0	1	4	3	12
CRYGS	-3.31947583	1.33127E-06	down	2	6	8	53	25	37
CRYL1	-4.56185353	7.93292E-23	down	17	47	39	728	319	704
CRYM	-3.239504253	0.00945506	down	5	3	4	17	56	3
CRYZ	0.821375405	6.46705E-05	up	1615	1772	1636	818	479	746
CRYZL1	-1.377672538	3.47728E-05	down	283	272	311	700	307	629
CSAD	-2.871755938	5.96294E-39	down	178	173	177	788	848	1111
CSAG1	10.39533644	4.55915E-17	up	321	396	333	0	0	0
CSDC2	-4.72700635	3.55839E-41	down	45	46	29	1067	495	727
CSE1L	2.48647002	1.1719E-100	up	8016	8850	7710	1131	983	996
CSF1	-1.874883221	7.96308E-18	down	346	350	321	738	891	1007
CSF1R	-2.419646432	5.53822E-08	down	259	269	302	1017	495	1727
CSF2RA	3.060830762	0.006054716	up	29	19	56	6	3	0
CSF2RB	-7.841232285	2.37273E-25	down	3	0	2	327	280	207

CSK	0.886174466	0.000531801	up	1079	1057	1128	541	389	329
CSMD1	4.326742939	0.000107661	up	29	22	33	2	1	0
CSMD2	-8.45015221	1.27554E-10	down	0	0	0	51	37	50
CSMD3	6.844407014	1.75191E-06	up	24	39	27	0	0	0
CSNK1A1	0.62481226	6.23284E-06	up	4755	5192	4417	2106	2125	2362
CSNK1D	-0.625623596	1.15747E-08	down	1247	1349	1268	1494	1211	1545
CSNK1G1	1.251025455	3.97967E-12	up	775	949	877	237	254	282
CSNK1G3	0.786701599	5.9223E-10	up	1214	1217	1227	542	458	510
CSNK2A1	0.732622891	1.02637E-07	up	1848	2047	1824	775	775	889
CSNK2A3	1.054697414	0.006792019	up	212	240	209	46	79	99
CSPG5	3.024817984	1.88727E-05	up	243	265	216	7	40	13
CSRNP2	-1.020272971	4.19328E-11	down	454	459	402	672	612	607
CSRNP3	-7.203170607	5.93259E-21	down	1	0	4	154	196	170
CSRP2	-1.908521351	0.001146282	down	759	890	724	3902	646	2010
CST3	-4.057616935	1.37224E-52	down	948	1025	1052	14589	7390	14478
CST6	-8.860893068	1.10747E-06	down	0	0	0	139	5	48
CST7	-7.222959976	9.0866E-08	down	1	0	0	47	37	30
CSTA	-9.022398844	1.44593E-10	down	0	0	2	120	493	87
CSTF1	0.821217457	2.53221E-06	up	658	744	726	336	259	262
CSTF2	1.830849946	6.14517E-37	up	779	843	768	165	151	161
CSTF2T	1.228706968	5.36516E-19	up	2105	2146	2078	641	634	639
CSTF3	1.89522933	6.54344E-38	up	1504	1630	1520	321	291	275
CT45A1	9.335652182	5.27664E-14	up	322	332	324	1	0	0
CT45A10	7.39035524	2.70688E-11	up	141	184	147	2	0	0
CT55	8.951649564	1.62443E-12	up	134	133	118	0	0	0
CT83	9.779590575	7.78825E-15	up	218	208	255	0	0	0
CTAG2	11.04904703	2.82298E-19	up	562	587	500	0	0	0
CTAGE6	5.024540058	0.010097265	up	8	5	12	0	0	0
CTBS	-1.573491911	2.62109E-12	down	290	337	260	750	444	699
CTCFL	7.880405045	7.3348E-10	up	112	113	131	0	1	0
CTDP1	-0.821505963	0.002001291	down	77	109	85	134	99	107
CTDSP1	-1.744606164	1.38658E-22	down	831	846	831	2410	1508	2118
CTDSP2	-0.858228479	6.51176E-05	down	2881	3267	2787	3202	3568	4701
CTDSPL	-1.438331468	1.21903E-10	down	621	701	619	1353	870	1556
CTDSPL2	1.418041145	3.74579E-23	up	1486	1650	1578	466	334	461
CTF1	-10.00739764	2.91465E-15	down	0	0	0	167	99	142
CTGF	-4.620024153	3.96171E-36	down	991	940	947	27150	9550	14832
CTH	2.557735352	3.35717E-14	up	744	944	1028	82	138	102
CTHRC1	-11.71849401	2.20431E-21	down	0	0	0	498	346	488
CTLA4	-5.477696207	0.017398895	down	0	0	0	1	8	8
CTNNA3	-5.620238273	1.60981E-07	down	6	5	6	32	448	70
CTNNAL1	1.164800792	1.20456E-09	up	3017	3303	2756	1050	986	821
CTNNBIP1	-1.288045559	0.000270454	down	206	290	231	375	536	320
CTNNBL1	0.763041165	3.10569E-07	up	1185	1355	1216	620	429	531
CTNND2	-5.066584317	0.008452721	down	0	1	3	1	81	5
CTPS1	1.946197539	5.03925E-05	up	4209	4822	4025	886	308	1280
CTPS2	0.927509289	0.00010645	up	999	1120	879	469	257	406
CTR9	1.335532228	1.05295E-32	up	2567	2785	2600	745	677	818
CTSB	-1.664824754	3.1967E-15	down	4737	5342	5044	9781	12457	11455
CTSC	-2.027476577	8.71911E-12	down	457	451	417	923	1479	1383
CTSD	-1.029373144	2.98022E-11	down	4201	4577	4419	6179	6547	6300
CTSF	-2.841758864	2.08711E-16	down	402	407	362	2549	1036	2519
CTSG	-8.394507765	1.5052E-06	down	0	0	0	104	22	10
CTSH	-9.869671639	8.55357E-44	down	2	3	1	1521	750	1786
CTSK	-6.32112465	4.48354E-71	down	40	34	29	2760	1183	2028
CTSL	0.923713094	1.74847E-06	up	6198	6521	5892	2453	2458	2008
CTSO	-6.275263065	0.000399708	down	0	0	0	18	6	7
CTSS	-2.854893116	1.80708E-23	down	241	234	208	1161	779	1620
CTSV	5.063833247	3.3043E-26	up	256	317	273	11	5	2
CTSW	-8.287003618	1.28118E-09	down	0	0	0	56	36	31
CTTN	1.032694457	1.4739E-09	up	7482	8008	7318	2743	2040	3200
CTXN1	4.728883236	1.46259E-29	up	304	370	293	15	6	5
CUBN	-10.06478383	1.26346E-15	down	0	0	0	138	116	168
CUL2	0.967855494	5.46961E-08	up	1707	1720	1457	666	575	529
CUL4A	2.073686308	1.27663E-47	up	6606	7249	6457	1059	1104	1253
CUL9	-1.045311743	9.52205E-06	down	220	272	271	358	296	471
CWC27	0.668043525	2.14406E-05	up	921	1074	969	460	358	513
CX3CL1	-10.22043518	2.04992E-26	down	0	5	0	2594	815	835
CX3CR1	-10.09681099	8.14868E-10	down	0	0	0	354	29	67
CXADR	4.966615499	0.006746585	up	1739	2002	1727	17	97	0
CXCL1	-5.597643475	0.014534557	down	0	2	0	1	57	2
CXCL10	-6.680487819	8.25227E-06	down	0	1	3	250	46	7
CXCL11	-5.022734332	0.004619294	down	0	0	1	4	13	7
CXCL12	-15.48073253	1.08631E-36	down	0	0	0	6874	5768	5264
CXCL14	-11.34922844	5.94387E-19	down	0	0	2	1262	332	2270
CXCL16	-1.496664661	2.43412E-12	down	198	219	203	457	303	495
CXCL2	-2.652281955	0.023126281	down	41	36	35	52	394	14
CXCL9	-9.164978226	3.03612E-12	down	0	0	0	65	89	68
CXCR2	-6.291176074	0.000167058	down	0	0	0	15	8	8
CXCR4	1.386536771	2.40734E-18	up	1169	1182	1046	299	307	314
CXCR6	-6.708953631	2.37633E-06	down	0	0	1	18	30	31
CXXC4	-2.686246911	5.37869E-08	down	18	12	11	90	47	53
CXorf21	-7.73007552	1.54313E-07	down	0	0	0	45	16	24
CXorf23	-0.801345732	0.010211411	down	213	297	232	313	202	412
CXorf36	-10.89466138	7.90405E-20	down	0	2	0	449	1263	891
CXorf38	1.59113936	4.78422E-31	up	968	1119	1061	262	231	249
CXorf57	1.882810407	6.64869E-05	up	131	154	147	46	13	26
CYB561	0.676713457	0.000288438	up	922	910	831	477	354	356
CYB5A	-3.720261904	1.41852E-07	down	260	281	231	878	4694	1210
CYB5B	2.374060859	9.84003E-15	up	7243	8041	7139	961	1293	758
CYB5D2	-2.555027626	9.92546E-31	down	92	118	87	476	392	367
CYB5R1	-1.356440831	3.07183E-19	down	498	602	554	1024	991	980
CYB5R2	-4.489046823	6.19431E-18	down	5	10	7	125	137	83
CYB5R3	-2.21811636	6.95693E-17	down	2087	2253	2070	7993	4412	9137
CYB5R4	-0.746338762	0.000113528	down	275	234	260	326	282	312
CYBA	0.957912774	8.02926E-05	up	2329	2514	2472	1189	619	906
CYBB	-3.578263613	1.31693E-39	down	101	112	93	1043	559	1032
CYBRD1	-4.35119147	5.44874E-40	down	964	1122	950	16126	8045	20688
CYFIP2	-6.965587638	2.80598E-15	down	4	2	16	1244	186	640
CYGB	-4.448571815	1.84348E-14	down	57	41	32	358	1102	477
CYHR1	-1.25424883	6.06441E-09	down	347	360	403	788	538	568

CYLD	-1.009621376	9.83419E-07	down	680	826	852	1123	894	1378
CYP1A2	-4.884610158	0.032626996	down	0	1	1	0	34	4
CYP20A1	-3.26568723	9.91486E-47	down	65	54	55	400	334	462
CYP26B1	-2.432630772	2.97503E-14	down	79	83	109	272	324	445
CYP27A1	-5.913243711	1.79211E-56	down	7	18	23	747	658	644
CYP27C1	4.068563103	5.32673E-23	up	158	181	180	9	7	6
CYP2C18	2.698680824	0.032056077	up	13	25	20	0	5	1
CYP2C19	5.215844533	2.59031E-05	up	37	36	33	0	2	0
CYP2C8	2.666259028	0.000510393	up	56	75	55	5	13	2
CYP2C9	4.992664332	5.13834E-06	up	101	102	82	0	6	0
CYP2E1	-2.733788036	0.003417305	down	8	6	3	30	7	46
CYP2R1	-1.374910222	0.001165248	down	187	185	147	476	157	347
CYP2S1	3.853741457	6.92809E-09	up	308	374	362	38	5	10
CYP2U1	-3.131264212	4.96667E-26	down	111	100	78	695	393	738
CYP39A1	-6.656015373	1.0942E-11	down	2	0	1	68	84	61
CYP3A4	4.109234503	0.001747548	up	57	62	64	0	7	0
CYP3A5	1.99762692	6.59093E-15	up	196	245	214	37	40	39
CYP46A1	-4.708376763	6.83623E-06	down	3	0	3	25	60	24
CYP4B1	-10.37788931	1.8634E-11	down	1	1	0	249	51	1660
CYP4F11	-2.935468752	0.007938853	down	3	4	14	88	19	12
CYP4F12	-2.952853183	6.07587E-07	down	13	19	26	60	84	177
CYP4F3	3.432057313	0.022751765	up	18	19	8	0	0	3
CYP4V2	-12.47265216	2.72759E-23	down	0	0	0	973	488	804
CYP4X1	-10.40854198	2.96031E-16	down	0	2	0	708	179	1090
CYP4Z1	-6.893337603	0.010757381	down	0	0	0	19	0	30
CYP51A1	2.175155165	2.99737E-09	up	4087	4506	3787	538	891	466
CYP7B1	-11.36843106	1.84761E-18	down	0	0	0	222	416	381
CYR61	-2.539479801	0.001713403	down	2316	2334	1894	22415	2402	3397
CYS1	-5.078311501	5.96636E-09	down	15	6	13	445	38	382
CYSLTR1	-10.08230169	8.91129E-15	down	0	0	0	108	173	137
CYSLTR2	-7.146957889	1.7342E-05	down	0	1	0	11	70	21
CYSRT1	3.270595104	8.00987E-11	up	101	114	115	8	12	4
CYTB	-2.843487396	2.13875E-49	down	26101	29994	26420	118199	137463	162712
CYTH1	0.961818185	1.92895E-06	up	1880	2037	1903	603	734	771
CYTH2	-1.056800847	4.19329E-08	down	585	574	604	1085	701	844
CYTH3	-0.592489701	0.025221438	down	1502	1597	1435	1682	1091	2148
CYTH4	-4.744796366	4.53692E-26	down	15	10	22	415	190	313
CYTIP	-5.411337182	6.62955E-15	down	3	0	5	73	80	90
CYTL1	-12.59049498	1.25615E-08	down	0	0	0	1595	51	901
CYYR1	-12.93355619	2.27079E-24	down	0	0	0	703	1166	1157
D2HGDH	-1.452308446	6.6246E-12	down	220	218	191	512	333	386
DAAM1	-2.406375204	3.41992E-28	down	327	375	342	1716	978	1278
DAAM2	-13.68715435	1.14761E-25	down	0	0	0	1693	849	2752
DAB1	-7.463176839	0.000519214	down	0	0	0	1	45	19
DAB2	0.782223424	0.002231773	up	4859	5312	4903	1606	2269	2283
DACH1	-1.830823648	0.026408679	down	20	29	21	16	100	52
DACT1	-8.663738438	3.43258E-33	down	1	6	1	1105	379	852
DACT2	-7.1342707	4.38642E-05	down	0	0	0	8	10	38
DACT3	-7.013974144	6.626E-24	down	13	18	10	2357	300	1241
DAD1	-0.61353746	4.13648E-05	down	1479	1681	1443	1990	1416	1614
DAG1	2.064186406	2.75434E-38	up	10619	11677	10299	1670	1706	2152
DAGLA	1.790376876	1.70035E-05	up	201	237	224	40	63	30
DAGLB	1.351367678	3.49348E-12	up	654	739	681	243	154	184
DAP3	1.22041412	2.17581E-06	up	3488	3823	3450	1019	1295	917
DARS2	1.568562768	0.005274117	up	1310	1387	1234	188	529	183
DAZAP1	1.352856797	5.29863E-08	up	2888	3019	2661	715	931	703
DBF4	3.941266076	1.63045E-43	up	1787	1976	1713	124	67	64
DBF4B	2.694586023	1.1838E-40	up	755	829	830	97	65	105
DBN1	1.172759511	5.65356E-05	up	3783	4056	3912	1298	769	1698
DBNDD1	2.678583997	4.80987E-06	up	538	590	547	67	17	108
DBNDD2	-4.303762554	1.51158E-06	down	6	3	1	69	17	59
DBR1	0.74168794	0.001482751	up	640	715	532	330	195	283
DBT	-0.836010759	0.032372887	down	775	775	701	667	1291	825
DBX2	-5.496521502	0.023870302	down	0	0	0	1	3	14
DCAF11	-1.31229503	6.33179E-21	down	498	624	525	993	925	976
DCAF13	1.698893214	2.57094E-18	up	1596	1617	1543	403	344	292
DCAF15	0.702838163	1.39978E-05	up	432	519	449	213	173	226
DCAF17	-0.921379012	0.000325783	down	264	360	257	349	334	500
DCAF4L2	8.300436368	1.23141E-10	up	80	83	82	0	0	0
DCAF5	-1.703405388	1.46085E-17	down	623	615	521	1187	1305	1565
DCAF7	0.616722655	0.000239023	up	2050	2319	2038	902	907	1155
DCAF8	-1.31334465	9.92269E-21	down	1313	1414	1283	2276	2076	2739
DCAKD	-1.304869966	4.92107E-11	down	161	153	166	322	259	264
DCBLD1	1.491131536	2.49847E-09	up	909	943	921	193	268	232
DCDC2C	-3.640357559	0.000493541	down	5	3	0	21	13	38
DCHS1	-10.12264767	9.08799E-39	down	0	1	3	1103	812	1280
DCLK1	-9.583498309	3.03063E-30	down	3	4	1	810	652	2970
DCLRE1A	1.148626753	6.22625E-14	up	575	659	547	212	168	191
DCLRE1B	2.064809264	3.05063E-16	up	635	639	565	81	104	126
DCN	-8.298301706	1.2462E-193	down	200	178	183	31971	37440	56378
DCP1B	-1.450476441	7.30226E-08	down	101	127	93	233	146	249
DCPS	2.200185562	2.33513E-49	up	847	937	815	148	121	133
DCT	-3.069774738	0.000325874	down	5	4	2	23	10	34
DCTD	-1.44512197	9.83083E-21	down	509	532	494	980	1000	976
DCTN2	-1.099633531	1.45706E-09	down	975	1031	893	1836	1165	1446
DCTN3	-1.008217566	4.06556E-06	down	448	516	415	831	579	563
DCTN4	-0.784438141	5.4183E-12	down	1176	1227	1077	1502	1322	1432
DCTPP1	1.558705674	9.98819E-12	up	997	1076	916	310	216	196
DCUN1D3	-0.743834166	3.11238E-05	down	277	296	326	357	331	383
DCUN1D4	-1.043409997	7.65089E-05	down	806	773	679	1489	758	1109
DCUN1D5	1.436087817	5.40326E-29	up	1611	1922	1548	477	404	451
DCXR	-1.533217035	0.00014027	down	280	336	309	528	883	438
DDAH1	-0.931101631	3.22969E-05	down	548	614	491	702	590	957
DDB1	1.407368796	1.64275E-17	up	7845	8711	8051	1991	1989	2609
DDB2	0.830149922	0.003691224	up	1101	1151	1190	565	283	553
DDC	4.797812864	0.002130222	up	12	13	17	0	1	0
DDI2	1.036612165	0.006069588	up	156	140	172	38	62	61
DDIAS	4.249997808	1.7807E-74	up	815	915	744	30	33	29
DDIT3	-1.3886438	0.014872153	down	247	310	234	955	289	256
DDIT4	1.381545436	0.024143226	up	8651	9719	8258	834	3841	2280
DDIT4L	-9.607429229	7.76525E-14	down	0	0	0	135	84	89

DDN	5.204986201	7.04574E-11	up	108	123	88	1	5	0
DDO	-8.018796435	4.27071E-13	down	0	2	0	119	122	125
DDR2	-6.206393971	6.6966E-34	down	54	47	53	1772	1440	5051
DDRGK1	-1.432958297	2.52771E-13	down	233	278	233	459	498	458
DDX1	0.782523286	1.15146E-11	up	4031	4533	4075	1717	1661	1828
DDX10	1.313541829	1.38132E-13	up	822	957	861	257	261	232
DDX11	4.067747206	1.38909E-67	up	956	1123	1076	36	48	49
DDX17	-1.181336253	3.82612E-07	down	5987	6339	5843	10143	6793	12702
DDX18	1.370946216	4.1981E-14	up	4150	4598	3774	1309	848	1312
DDX20	0.955885629	2.05625E-10	up	754	843	717	328	238	284
DDX21	3.040618658	4.2131E-129	up	11452	12490	11055	962	939	1116
DDX23	0.956305	9.27149E-10	up	2871	3101	2717	1049	878	1267
DDX27	1.393544014	2.85007E-25	up	1959	2003	1943	584	434	589
DDX28	0.74431727	0.030702852	up	311	335	313	130	170	100
DDX31	2.542540347	1.60545E-36	up	978	991	1004	107	128	126
DDX39A	1.378675163	1.45836E-07	up	1332	1447	1329	502	339	284
DDX3X	1.275283957	5.47002E-10	up	18548	19450	18272	4833	6031	5486
DDX3Y	-13.55592971	0.001202101	down	0	0	0	2236	0	2739
DDX43	-8.392230949	1.54996E-06	down	0	0	0	74	5	59
DDX46	1.712303202	1.19462E-15	up	5089	5325	4645	943	931	1398
DDX47	1.104767421	9.46828E-11	up	2068	2211	1852	774	514	753
DDX49	0.662773116	0.006662998	up	705	669	630	335	317	243
DDX5	-0.778200409	9.44958E-19	down	9380	10218	8893	12649	10223	11895
DDX52	1.486780904	3.6197E-06	up	245	280	235	46	60	86
DDX54	1.268503123	2.1949E-24	up	2094	2261	2149	625	605	686
DDX55	1.044320796	1.59418E-12	up	1036	1074	1038	353	357	372
DDX56	1.135468734	5.47377E-13	up	1552	1716	1499	563	507	466
DDX58	-0.977232833	0.014717215	down	353	397	341	833	340	377
DDX60	-1.362455844	2.50859E-10	down	277	297	273	604	510	429
DDX60L	-2.176126353	3.53734E-16	down	133	162	124	580	408	356
DECR1	-2.501721926	0.001036201	down	452	472	428	751	3674	665
DEF6	-4.881081683	1.74073E-07	down	0	9	4	158	49	66
DEFB124	-6.294168	0.000104451	down	0	0	0	11	8	12
DEK	1.226776543	2.9858E-07	up	6347	7328	6219	2177	1356	2578
DENND1A	1.351321731	1.88817E-17	up	1080	1130	1132	319	254	363
DENND1C	-2.561936575	2.44941E-06	down	9	8	18	64	39	46
DENND2A	-5.96740517	2.87067E-31	down	6	4	3	164	195	216
DENND2D	-3.728407467	2.92068E-10	down	19	21	13	86	252	142
DENND4B	0.663250399	9.29124E-05	up	1223	1153	1235	622	495	509
DENND4C	-1.000335283	0.000282157	down	850	787	905	1045	954	1649
DENND5B	0.876321656	7.30849E-05	up	985	1028	916	345	420	357
DENND6A	-1.683327306	2.05899E-10	down	265	369	338	761	519	958
DENND6B	-1.202648723	0.000228387	down	39	43	49	80	71	63
DENR	0.961016343	1.22089E-10	up	2519	2669	2394	1007	906	846
DEPDC1	5.695712139	7.47643E-09	up	1499	1666	1310	32	28	0
DEPDC1B	5.317649282	3.77031E-24	up	599	719	546	20	2	12
DEPDC4	1.879666213	0.026588014	up	39	41	39	3	15	4
DEPDC7	4.891326448	1.40666E-64	up	1502	1777	1627	51	41	25
DEPTOR	-1.501066146	0.000820448	down	144	163	189	191	442	342
DERA	0.946353828	5.00747E-10	up	927	952	790	334	291	360
DES	-6.295401397	8.97567E-12	down	266	258	228	2501	31903	4229
DESI1	1.004093226	0.000764468	up	1451	1529	1467	462	652	434
DET1	-1.588646236	1.20117E-06	down	67	69	58	113	161	135
DEXI	-0.676067831	0.000101282	down	445	510	453	590	412	607
DFFA	1.228397634	3.77187E-12	up	978	962	860	264	284	298
DFNA5	1.526339085	2.89986E-15	up	1255	1267	1215	362	223	347
DFNB59	-2.554770474	1.86425E-09	down	16	21	28	124	65	86
DGAT1	-2.099412182	0.001225974	down	176	205	183	224	1012	393
DGAT2	-5.958172319	8.55337E-05	down	144	139	143	312	15743	1048
DGCR6L	-1.5773223	7.25786E-10	down	277	273	271	768	516	465
DGKD	1.35344135	3.43645E-15	up	1413	1593	1551	395	435	430
DGKH	-1.455516114	1.23485E-07	down	403	516	371	819	586	1126
DGKI	-3.025799617	6.89403E-07	down	20	15	15	48	79	164
DGKQ	-0.987997768	1.54913E-05	down	112	127	111	151	148	194
DHCR24	1.912484134	0.022953064	up	8288	8808	7988	243	2949	1270
DHCR7	2.144196823	0.005880777	up	3878	4274	3939	157	1269	397
DHDH	-3.250719375	0.032027826	down	2	4	1	39	8	1
DHFR	1.582212107	2.40059E-06	up	1170	1293	1086	199	357	268
DHFRL1	-0.892585785	0.010282787	down	147	183	154	236	125	287
DHH	-3.842638361	2.48273E-07	down	4	8	2	54	58	27
DHODH	0.931420472	0.021318369	up	230	311	220	69	125	82
DHPS	-0.713963798	7.52747E-05	down	479	536	484	691	445	625
DHRS1	-1.502332806	7.2913E-13	down	106	104	91	220	165	224
DHRS12	-4.538018196	3.26559E-31	down	13	11	10	247	149	170
DHRS13	2.062954021	1.19947E-07	up	299	369	355	71	68	33
DHRS2	4.192685983	0.000181154	up	24	22	30	1	0	2
DHRS3	-2.568618192	5.62288E-56	down	289	301	304	1201	1254	1300
DHRS4	-1.22246369	9.99821E-05	down	190	203	156	269	368	257
DHRS7	-1.445039433	2.33981E-07	down	659	552	551	1484	743	1247
DHRS7B	-1.3621819	0.00170459	down	170	161	167	239	432	215
DHRS9	-7.472927888	2.60693E-08	down	0	0	1	40	53	41
DHX15	1.10696828	3.71197E-19	up	3921	4215	3745	1278	1171	1472
DHX30	0.859314437	1.4614E-13	up	1913	2149	1947	832	746	771
DHX32	0.810633313	0.001360018	up	845	933	805	324	404	304
DHX33	1.747593242	4.77448E-37	up	1438	1554	1264	318	265	318
DHX34	1.217981417	8.61626E-09	up	483	489	522	191	123	146
DHX36	1.10059805	0.000264803	up	1247	1408	1298	307	424	572
DHX37	1.85374076	6.99977E-18	up	848	923	797	162	187	151
DHX57	1.027314831	1.03267E-07	up	883	875	765	324	221	342
DHX58	-5.036014125	4.17939E-34	down	11	10	5	253	159	198
DHX8	0.935832357	1.4867E-09	up	1644	1696	1533	562	564	682
DHX9	1.641350785	2.94633E-19	up	7430	8000	6890	1529	1474	2086
DIAPH1	2.277018308	1.65796E-26	up	6937	7707	7152	932	887	1387
DIAPH2	-4.60469643	1.22794E-49	down	27	23	27	415	349	579
DIAPH3	5.05446365	3.67345E-38	up	1941	2077	1924	34	23	72
DIDO1	0.645410995	0.009518037	up	2792	3018	2814	1443	872	1657
DIEXF	1.35941223	2.20246E-14	up	1096	1198	1092	277	300	358
DIO2	-3.715020915	1.45992E-15	down	47	41	32	620	196	332
DIO3	-1.331515415	0.026862812	down	9	18	22	22	30	35
DIP2B	1.995034907	2.47206E-22	up	3407	3705	3461	520	638	715
DIP2C	-2.070330744	1.37893E-15	down	372	414	359	1366	727	1375

DIRAS3	-7.368990498	3.26208E-10	down	1	0	3	108	295	48
DIRC2	1.239666128	7.57883E-10	up	651	704	666	181	178	250
DIS3	0.686296936	9.73315E-05	up	2059	2309	2089	1072	711	1093
DIXDC1	-1.29351236	4.17299E-07	down	1090	1227	1041	1982	1327	2606
DKC1	2.453217955	2.2186E-42	up	3741	4071	3663	571	491	422
DKK1	3.773289999	2.99528E-30	up	921	968	763	29	54	53
DKK2	-9.544289765	3.25432E-07	down	0	0	0	38	223	9
DKK3	-9.185683225	6.73836E-55	down	35	19	36	23163	4547	11271
DLC1	-9.355684511	7.52616E-61	down	0	3	8	1531	1901	1639
DLEC1	-5.852566056	0.000122834	down	0	0	1	19	8	18
DLEU7	-5.765224824	0.005747717	down	0	0	0	6	2	14
DLG4	-1.858054407	7.54984E-12	down	138	90	119	326	277	297
DLG5	2.78240562	8.18436E-05	up	39	125	74	4	9	11
DLGAP1	-5.549334912	8.05855E-05	down	1	2	0	22	7	73
DLGAP3	-4.980848157	0.003102569	down	0	0	1	5	8	11
DLGAP5	5.691530706	9.37179E-12	up	1594	1852	1488	52	15	2
DLK2	5.22581335	3.61159E-11	up	78	107	81	2	3	0
DLL1	-4.798005939	0.037986462	down	0	0	0	6	3	2
DLL3	6.216278844	3.5838E-11	up	103	118	92	2	0	1
DLL4	-3.96431418	3.14405E-09	down	17	13	25	168	334	89
DLX3	3.651402499	0.000513342	up	39	50	41	1	6	0
DLX4	7.834448674	2.39049E-12	up	212	177	247	1	0	1
DLX5	-7.41787868	7.15322E-07	down	0	0	0	19	14	35
DMBX1	9.239728397	2.01112E-13	up	148	174	149	0	0	0
DMC1	-2.915058877	0.010438272	down	15	9	1	99	14	26
DMD	0.833105174	0.030588548	up	6792	7544	6707	2349	1701	4469
DMGDH	-8.457479137	4.24857E-10	down	0	0	1	107	107	51
DMKN	1.389349495	0.016206842	up	527	580	472	240	44	159
DMPK	-2.189447639	1.82842E-17	down	649	808	745	2872	1524	2845
DMRT1	5.774856888	0.001378689	up	10	8	24	0	0	0
DMRT2	-7.759769362	0.006442059	down	0	0	0	0	72	5
DMRTA1	1.685029871	0.046621719	up	47	42	51	2	17	11
DMRTA2	8.2361299	1.2602E-10	up	117	175	166	0	1	0
DMXL1	-0.965676477	0.000420246	down	685	763	786	877	821	1422
DNA2	3.839573077	2.217E-106	up	1195	1243	1126	63	55	59
DNAAF3	3.905614855	4.01313E-07	up	84	108	66	6	6	0
DNAAF5	2.035542603	5.3457E-45	up	1534	1753	1595	332	245	271
DNAH1	-3.322171968	9.12106E-25	down	30	48	39	233	237	360
DNAH12	-3.746981883	0.014011875	down	1	1	0	8	6	5
DNAH14	4.563684985	6.96997E-19	up	663	790	608	19	33	8
DNAH5	4.522380074	5.79601E-12	up	135	159	133	6	7	0
DNAJB11	0.878123263	0.001105161	up	1666	1968	1570	856	649	499
DNAJB14	-1.050447742	7.29389E-05	down	562	609	584	798	628	1182
DNAJB2	-1.043353179	2.07985E-07	down	726	787	751	1422	902	1015
DNAJB4	-1.67546185	1.72214E-05	down	1143	1082	937	3846	1316	2188
DNAJB5	-3.338007636	2.07106E-09	down	109	153	124	1745	341	773
DNAJB9	-1.427719982	5.04656E-11	down	305	307	266	717	466	505
DNAJC1	-1.180148138	8.2627E-11	down	318	309	349	573	504	496
DNAJC11	1.206709279	7.44393E-05	up	1502	1757	1648	378	614	491
DNAJC12	3.684543666	9.56528E-64	up	909	1027	999	64	54	44
DNAJC14	1.2902777	4.03102E-19	up	1574	1777	1477	455	459	482
DNAJC15	-1.997353891	5.34364E-15	down	297	379	314	1085	981	717
DNAJC18	-2.624827018	8.61209E-13	down	129	121	88	581	275	654
DNAJC19	-1.05144351	2.48704E-06	down	398	459	323	710	510	517
DNAJC2	2.566603455	2.67557E-36	up	2741	3160	2960	430	341	290
DNAJC21	1.136215589	9.797E-12	up	3200	3378	3028	1128	789	1214
DNAJC22	5.886135214	9.82475E-29	up	886	945	843	18	12	2
DNAJC27	-1.794407274	4.36213E-17	down	96	83	90	248	189	230
DNAJC4	-2.304000387	2.40936E-19	down	81	91	89	336	322	252
DNAJC5	0.603415586	0.000857211	up	1839	2012	1844	822	932	889
DNAJC5G	-3.9677298	0.021998286	down	3	0	1	2	34	6
DNAJC6	2.324581942	4.32633E-13	up	440	472	413	45	78	62
DNAJC7	0.655412399	0.000322531	up	2521	2719	2262	1200	1156	1006
DNAJC9	2.004415618	4.94849E-25	up	1737	1840	1776	407	255	293
DNAL4	-0.889224555	0.000132852	down	114	133	149	195	160	167
DNALI1	-10.19564147	4.47269E-15	down	0	0	0	203	89	177
DNASE1L2	1.707141661	0.040578266	up	19	33	19	5	8	2
DNASE1L3	-7.39177439	3.99669E-15	down	0	4	0	129	208	123
DNER	3.516243569	0.000104262	up	1091	1308	1119	127	4	98
DNLZ	-1.002852847	0.028765889	down	98	82	77	193	99	78
DNMBP	0.718669806	0.012670109	up	773	869	790	257	345	442
DNMT1	2.359703855	9.37308E-58	up	3131	3433	3264	421	439	497
DNMT3B	3.850108775	4.62869E-79	up	1010	1114	1055	51	57	48
DNPEP	-1.990568005	3.13877E-11	down	200	267	211	489	749	640
DNPH1	1.131787381	0.001828879	up	667	722	712	249	279	143
DNTTIP2	1.375878155	9.41606E-25	up	2897	3125	2752	903	642	871
DOC2A	-1.880517514	0.0099746	down	10	9	7	11	27	29
DOCK1	-0.755350977	0.000246531	down	1081	1255	1163	1235	1222	1743
DOCK10	-1.233670086	0.040801555	down	463	507	465	803	244	1430
DOCK11	-2.928924548	1.15122E-21	down	291	263	237	1003	1452	1800
DOCK2	-1.796189946	3.92743E-06	down	90	120	99	230	154	388
DOCK4	-2.112445882	3.64045E-46	down	165	180	169	541	493	544
DOCK6	-1.371638907	0.025679361	down	542	558	585	508	1805	640
DOCK7	-0.667839686	0.00132738	down	694	673	648	756	590	948
DOCK8	-3.04150186	5.05768E-23	down	61	84	50	344	298	499
DOCK9	-1.061194269	0.001247211	down	1017	1101	959	1002	1682	1818
DOHH	0.761801377	0.003425227	up	294	283	253	136	117	94
DOK1	-1.750338571	5.6191E-09	down	84	125	131	353	216	249
DOK2	-10.52245891	5.81155E-16	down	0	0	0	284	117	187
DOK3	-1.235157015	0.001356393	down	39	53	70	115	68	91
DOK4	1.685674768	2.87928E-16	up	2218	2323	2270	657	389	473
DOK5	-10.51608159	1.46948E-14	down	0	0	0	316	167	95
DOK6	-10.04657541	1.72389E-15	down	0	0	0	123	138	152
DOLPP1	1.792079325	6.34545E-11	up	568	631	557	105	145	105
DONSON	2.080666651	4.07972E-35	up	1015	1082	987	182	135	204
DOPEY1	-2.801615515	8.61796E-42	down	140	158	137	803	530	841
DOT1L	1.700498465	2.4395E-15	up	873	1006	917	232	210	166
DPAGT1	1.672058706	1.29832E-32	up	1201	1239	1195	264	258	288
DPCD	1.5860466	3.16131E-10	up	568	633	614	141	165	119
DPEP1	4.085357673	0.00029703	up	62	121	75	0	9	1
DPEP2	-6.523790719	2.70687E-17	down	1	2	2	124	72	135

DPF1	3.248372645	5.95935E-07	up	78	85	73	6	1	11
DPF3	-3.107760987	9.49365E-08	down	16	14	17	101	133	48
DPH2	1.263326482	2.15837E-10	up	643	684	539	171	187	191
DPH3	-0.634673512	0.000493797	down	525	567	590	746	499	625
DPH5	0.775506679	0.004688879	up	826	830	691	396	330	244
DPM1	1.112604594	2.3424E-12	up	1899	2060	1822	770	538	599
DPM2	1.754485479	1.48545E-20	up	1146	1295	1226	319	229	226
DPM3	-0.961008721	0.016013468	down	153	191	165	352	205	149
DPP3	1.197843121	3.05951E-05	up	716	876	733	174	255	283
DPP4	-4.05010802	3.23992E-10	down	8	10	4	46	109	97
DPP8	-0.635000842	0.002089002	down	1020	1031	1057	1022	1003	1415
DPP9	1.230505473	1.36176E-09	up	1803	2081	1916	516	632	591
DPPA2	8.0564109	1.03625E-09	up	56	72	79	0	0	0
DPT	-11.74140579	2.38861E-45	down	0	1	3	4765	1979	3186
DPY19L1	0.600928152	1.05209E-05	up	1275	1482	1211	698	521	644
DPY19L2	-11.93529714	1.48783E-18	down	0	0	0	218	623	665
DPY19L3	-1.780672834	1.12309E-14	down	291	343	277	828	516	902
DPYD	-0.890033222	0.001126268	down	526	571	458	525	741	757
DPYS	-3.775037008	0.025085314	down	0	0	2	8	4	8
DPYSL2	-1.526168827	6.56105E-30	down	1388	1352	1480	3173	2505	3007
DPYSL3	-11.19501573	2.07264E-65	down	3	5	2	6985	2422	7629
DPYSL4	2.927588453	0.037070923	up	93	142	107	2	27	0
DR1	1.612586565	1.80331E-42	up	3178	3435	2995	845	658	733
DRAM1	0.862468758	0.00015015	up	1033	1227	1008	368	365	544
DRD1	-7.085367532	7.18707E-05	down	0	1	0	81	4	24
DRD2	-5.030731218	0.020477575	down	0	0	0	7	3	3
DRGX	6.957562874	8.36966E-07	up	25	38	34	0	0	0
DROSHA	1.899177687	3.42507E-70	up	3118	3393	3008	611	551	651
DSC3	1.069255729	0.011333535	up	71	74	62	31	23	16
DSCAML1	-6.021974375	0.00018847	down	0	0	1	14	26	8
DSCC1	2.115255621	2.39116E-06	up	448	460	459	132	44	53
DSG2	4.204887625	2.15293E-10	up	5943	6788	6238	65	421	209
DSN1	2.165133279	5.57699E-30	up	1404	1505	1343	269	214	190
DST	-1.107339714	0.001208288	down	5092	5653	5440	7091	5371	12656
DSTN	-2.678176438	7.833E-06	down	6652	7348	6312	45745	7482	42720
DTD2	-0.608703454	0.004403679	down	226	203	177	236	183	241
DTHD1	-5.643244925	0.008484116	down	0	0	0	3	3	14
DTL	5.522240435	3.46558E-44	up	1843	1881	1616	28	39	14
DTNA	-10.87339926	6.41659E-24	down	0	0	2	793	795	1081
DTNB	0.637214765	0.031227055	up	190	227	193	72	92	113
DTNBP1	-0.957369517	2.86242E-05	down	189	202	176	310	188	292
DTWD1	0.664609409	0.000211823	up	1346	1406	1247	731	464	612
DTWD2	0.597619881	0.018718605	up	231	291	220	117	124	104
DTX1	-10.15463342	2.77232E-09	down	0	0	0	21	313	82
DTX2	1.688554754	0.000559038	up	132	191	218	22	51	44
DTX3	-9.897509854	1.26864E-25	down	1	1	1	913	367	798
DTYMK	1.371784204	0.000301305	up	636	688	770	263	197	114
DUOX1	-6.526876544	0.000887162	down	1	0	0	36	32	1
DUOX2	-4.036765511	0.008474274	down	1	1	0	10	9	4
DUOXA1	-2.785987596	0.020095224	down	1	1	4	10	14	5
DUS2	1.086247256	0.019287509	up	313	377	334	89	168	75
DUS3L	1.034707586	5.72352E-05	up	424	469	462	132	178	155
DUS4L	1.436004752	5.54238E-06	up	352	412	372	71	117	106
DUSP1	-2.190732837	0.000127882	down	1805	1977	1750	10857	5097	2126
DUSP12	0.833778013	0.000256063	up	596	740	585	306	180	286
DUSP15	-9.301003842	4.48682E-09	down	0	0	0	181	21	56
DUSP16	1.844871615	0.005779278	up	30	36	21	6	7	4
DUSP18	-1.38428615	0.000231312	down	61	51	42	74	106	103
DUSP19	-2.172087633	2.3565E-08	down	63	72	66	316	119	222
DUSP2	2.117892789	0.004908778	up	189	200	154	61	22	7
DUSP22	-2.396966181	2.52963E-47	down	210	247	252	891	763	1007
DUSP23	-2.084796793	1.07482E-10	down	163	157	160	564	542	329
DUSP26	-8.640423593	1.47829E-26	down	0	5	2	862	261	897
DUSP27	-11.44408546	1.11926E-16	down	0	0	0	196	585	268
DUSP3	-0.784342351	4.89752E-17	down	2049	2124	2011	2725	2247	2613
DUSP5	2.815243622	5.22047E-06	up	1160	1269	1101	139	174	33
DUSP6	-5.326209906	1.93985E-23	down	47	65	38	958	2280	838
DUSP9	8.486376017	1.19603E-21	up	539	511	467	0	3	0
DUT	0.655084095	0.008632742	up	2126	2348	2145	1331	687	1009
DVL1	1.492650978	1.00196E-31	up	2183	2422	2147	586	477	647
DVL2	0.927619176	1.09499E-10	up	1033	1080	1070	388	357	446
DVL3	-0.745135817	1.05407E-09	down	1042	1182	999	1337	1091	1418
DYM	-0.951371021	1.0126E-15	down	567	627	590	870	705	883
DYNC1H1	1.206523133	1.52959E-05	up	15344	17030	15494	3527	5006	6088
DYNC1I1	-5.893403632	1.74291E-20	down	10	4	4	314	114	357
DYNC1I2	-1.164211527	7.6309E-09	down	1337	1531	1385	2523	1614	2707
DYNC1LI2	-0.920688009	1.15673E-06	down	2322	2634	2280	2873	2891	3962
DYNC2LI1	-1.334912549	1.17139E-11	down	189	214	163	393	269	357
DYNLL2	-1.022321334	2.41931E-10	down	950	924	844	1242	1298	1369
DYNLRB1	-0.772697709	0.000122606	down	1145	1131	1104	1716	1025	1410
DYNLT1	-0.821349384	0.0081771	down	762	824	820	1237	1076	689
DYNLT3	0.789977455	0.014034575	up	3062	3210	3055	1647	723	1544
DYRK3	2.150899088	2.68211E-11	up	1022	1070	1025	128	215	146
DYRK4	-7.435205155	2.73802E-22	down	0	3	4	458	156	265
DYSF	-3.137047602	7.77723E-09	down	85	85	124	376	988	410
DZANK1	-2.455102173	1.54513E-07	down	8	14	19	56	48	56
DZIP1	-1.224112528	2.72197E-05	down	323	324	282	584	321	662
DZIP3	-0.707004115	2.25906E-05	down	351	384	321	391	376	456
E2F1	4.376409538	4.46134E-10	up	1334	1592	1544	60	79	9
E2F3	2.016819095	2.15196E-52	up	1567	1737	1480	317	237	288
E2F5	2.541548976	3.59748E-08	up	280	328	254	23	53	26
E2F6	1.007985968	1.32317E-09	up	563	626	586	200	208	217
E2F7	5.622898339	1.29762E-25	up	706	811	626	9	18	3
E2F8	4.11333225	4.55989E-07	up	308	247	256	10	21	1
E4F1	1.00357625	2.25011E-05	up	631	689	695	315	176	231
EAF2	1.385781749	0.026514471	up	116	122	133	66	14	24
EAPP	-1.092364371	6.95585E-10	down	424	477	352	662	525	715
EBAG9	-1.390314085	5.76624E-13	down	240	276	207	452	453	432
EBF1	-11.18098149	3.47872E-35	down	1	1	1	2097	1090	1837
EBF2	-12.08847407	1.42345E-17	down	0	0	0	182	836	632
EBF3	-8.927033437	7.42405E-21	down	3	1	0	216	640	490

EBF4	-5.394567537	4.5282E-12	down	0	5	6	144	55	136
EBNA1BP2	2.329671635	2.34587E-19	up	3800	4330	3717	791	429	469
EBP	2.720875795	1.23774E-05	up	3854	4008	3821	218	766	210
ECD	0.636744433	1.55007E-07	up	855	975	886	448	363	432
ECE2	2.731993908	1.25615E-08	up	226	231	225	16	38	17
ECEL1	-3.288569647	8.92358E-09	down	7	9	7	34	56	68
ECH1	-1.779928392	0.000452256	down	18	27	15	66	28	54
ECHDC2	-4.011920275	7.21427E-67	down	159	164	121	1671	1314	2134
ECM1	0.743859116	0.006461007	up	1334	1536	1396	462	678	646
ECM2	-5.565376994	2.2264E-62	down	65	72	81	2877	1402	3212
ECSCR	-11.12174121	3.29093E-18	down	1	0	0	510	721	438
ECT2	4.950628615	1.29241E-60	up	4205	4482	4104	94	125	70
EDA	-2.691900833	1.58619E-11	down	25	28	26	98	92	175
EDA2R	-6.963501212	8.49451E-19	down	2	2	2	168	93	282
EDAR	3.96842922	0.029008185	up	4	12	8	0	1	0
EDC3	0.85017373	0.000482981	up	1069	1209	1083	356	483	471
EDC4	0.602308784	2.39687E-05	up	1021	1140	1067	571	408	540
EDEM1	1.3879765	6.73992E-13	up	1718	1970	1726	420	468	576
EDEM2	0.699300189	0.001757974	up	745	792	800	347	372	296
EDIL3	-3.731138209	2.06284E-10	down	334	357	382	4542	861	5126
EDN2	4.750691379	5.44811E-14	up	88	140	112	4	1	4
EDNRA	-2.120212441	2.34649E-15	down	248	280	254	1128	634	674
EDNRB	-11.27459808	2.73871E-14	down	0	0	1	208	1225	346
EDRF1	0.728336883	0.009254766	up	574	679	517	193	257	302
EEA1	0.598348317	0.007574262	up	1804	1850	1754	709	778	1050
EED	1.832148035	1.73483E-17	up	1428	1614	1344	380	214	289
EEF1A1	-1.080463449	2.91044E-08	down	47715	52041	45576	93637	55688	71040
EEF1AKMT1	-1.215342972	5.48228E-05	down	82	81	75	176	96	125
EEF1E1	1.427238525	3.34974E-05	up	217	225	192	83	42	44
EEF2KMT	1.711165271	8.2215E-17	up	459	502	470	92	108	109
EFCAB1	-6.262200289	0.00052234	down	0	0	0	17	4	10
EFCAB13	-1.022404446	0.0029145	down	60	98	57	91	99	117
EFCAB14	-1.285437617	2.36351E-10	down	1562	1799	1625	2713	2284	3684
EFCAB6	-4.547428404	0.000451694	down	1	0	2	24	19	7
EFCC1	-8.563545876	4.32129E-09	down	0	0	0	25	71	48
EFEMP1	-1.939632696	5.29805E-28	down	6950	7796	6935	24759	15216	19617
EFEMP2	-4.521533907	2.15047E-81	down	110	83	111	1943	1291	1796
EFHD1	-1.846228881	0.011611537	down	734	814	719	2252	330	3447
EFHD2	1.810596956	3.9274E-12	up	2413	2707	2492	724	411	419
EFNA1	2.360265159	2.35416E-10	up	2224	2596	2321	217	446	299
EFNA2	7.245792751	1.96222E-07	up	33	53	33	0	0	0
EFNA3	4.413924413	3.9491E-51	up	563	518	567	15	19	21
EFNA4	2.102611247	1.08206E-07	up	271	322	280	69	48	27
EFNB1	-2.166366677	7.48772E-07	down	177	157	157	356	757	409
EFNB2	-3.520958725	2.7209E-47	down	100	157	130	1002	1118	997
EFNB3	-6.588345581	0.000620264	down	1	0	0	17	50	2
EFR3A	-1.217976113	2.23141E-09	down	996	1142	988	1736	1295	2174
EFR3B	1.710136367	0.025079819	up	237	219	190	29	89	15
EFS	-11.4075004	2.9437E-19	down	0	0	0	486	225	373
EFTUD2	1.920249094	3.73449E-18	up	3701	4089	3598	602	793	717
EGFL7	-1.034355714	0.004035604	down	723	801	738	1210	1339	689
EGFLAM	-6.773607384	4.71789E-18	down	3	2	3	167	322	113
EGFR	2.361435744	5.99308E-08	up	4475	5022	4772	366	519	1096
EGLN1	0.645892151	0.00969882	up	2787	3171	2840	1057	1457	1434
EGR1	-5.352870695	3.28597E-17	down	67	101	108	3566	3559	711
EGR2	-5.931446572	1.20841E-06	down	5	5	1	181	265	8
EGR3	-7.314526829	9.33566E-22	down	2	7	2	692	368	174
EHBP1L1	0.75028348	0.016800192	up	6034	6703	6112	2754	1613	3726
EHD2	-1.153581949	3.59748E-08	down	1776	1895	1887	2448	2849	3460
EHD3	-12.44193607	6.63124E-23	down	0	0	0	895	443	884
EHD4	-0.909126638	1.85791E-06	down	772	718	825	1127	1017	946
EHF	3.300910103	0.009109521	up	16	11	15	1	2	0
EHHADH	-2.855243916	0.000434319	down	63	89	64	143	755	134
EHMT1	0.630323827	0.00087902	up	1161	1393	1218	539	488	707
EI24	0.78797556	0.000174805	up	3729	4110	3507	1560	1701	1358
EID1	-0.908512868	5.13187E-07	down	4063	4363	3822	6297	4013	6189
EIF1	-1.010020198	1.47199E-05	down	5285	5641	5046	9885	6932	6077
EIF1AD	1.055375233	7.20896E-14	up	813	950	816	318	244	322
EIF1AX	1.034010859	4.76169E-09	up	4216	4846	4051	1436	1597	1474
EIF1AY	-11.67954187	0.005687215	down	0	0	0	780	0	578
EIF1B	-1.205146258	9.19247E-08	down	656	638	575	1195	1028	829
EIF2AK1	1.692750019	4.71364E-18	up	4847	5074	4586	920	1125	1117
EIF2B5	-0.611710909	3.45313E-05	down	527	662	598	724	567	651
EIF2D	-0.799565701	1.92544E-06	down	659	745	737	1052	706	909
EIF2S1	1.143127144	1.78003E-15	up	3593	3715	3349	1190	1142	1083
EIF2S2	1.061364421	7.02755E-12	up	4089	4407	3798	1566	1349	1259
EIF2S3	2.44483688	1.20486E-33	up	20323	21446	18668	2831	2797	2195
EIF3B	1.980888103	4.90473E-32	up	8045	8960	7802	1445	1569	1413
EIF3G	-1.21480963	2.07109E-05	down	1254	1328	1208	2970	1372	2011
EIF3I	0.788358871	1.23741E-07	up	4436	4692	4158	2131	1664	1675
EIF3J	1.170210902	2.08284E-15	up	3773	3868	3375	1254	1134	1080
EIF3L	-0.928923373	2.12139E-06	down	3697	4053	3704	6618	3920	5108
EIF3M	0.679417253	0.018718605	up	4838	5240	4541	3116	1668	1764
EIF4A2	-1.395802964	2.25553E-09	down	5939	6356	5684	14340	7522	12245
EIF4A3	1.772509896	4.15301E-44	up	2638	2872	2615	651	500	542
EIF4B	0.613467984	6.46275E-06	up	17764	19024	17279	8111	7342	9699
EIF4E3	-3.208545774	4.91967E-44	down	132	123	131	735	896	889
EIF4EBP1	1.746228196	9.30223E-08	up	1102	1267	1165	224	322	187
EIF4EBP2	-0.96704526	0.005068489	down	2182	2363	2161	1991	3623	3550
EIF4ENIF1	-1.227300328	2.13623E-05	down	224	258	249	360	301	565
EIF4G1	1.638248267	1.00891E-06	up	12001	13013	12067	1912	3550	2826
EIF4H	1.106024355	7.19611E-19	up	11518	12592	11411	4061	3234	4483
EIF5A	2.088298495	2.05553E-15	up	11085	11882	10486	1757	2231	1511
EIF5A2	-0.896564432	0.004861716	down	152	219	161	304	150	257
EIF5AL1	3.153150257	2.66892E-12	up	407	402	341	32	43	15
EIF6	1.171121406	4.67773E-14	up	2646	3003	2626	1036	782	795
ELAC1	-2.486549126	3.68693E-10	down	30	18	32	122	107	91
ELAC2	0.666712964	0.043120571	up	1672	1763	1715	553	945	766
ELAVL1	0.972999672	7.36559E-19	up	1887	2070	1903	746	666	707
ELAVL2	-3.866561337	0.029583917	down	0	0	2	11	7	3
ELAVL4	-5.533600876	0.019084696	down	0	0	0	14	1	4

ELF2	-1.530333656	2.24442E-13	down	492	499	385	945	746	1148
ELF3	9.804782115	9.35244E-46	up	2002	2066	2189	5	0	0
ELF4	1.730892077	1.7772E-35	up	969	1101	1010	226	212	220
ELFN1	-0.979733791	0.03936951	down	29	42	31	56	54	31
ELFN2	6.986976623	1.43927E-57	up	830	896	778	2	6	6
ELK1	1.743292356	7.04531E-40	up	1831	2024	1836	395	394	415
ELK3	-1.142624626	0.000183534	down	738	856	721	1022	1511	1023
ELL	0.711805455	0.002655804	up	540	523	471	246	157	270
ELL2	0.778625521	0.002595351	up	4329	4390	4073	1387	1609	2300
ELMO1	-8.684987901	4.53337E-26	down	1	3	0	325	316	529
ELMO2	-0.926214933	2.29085E-07	down	465	510	514	748	516	764
ELMO3	1.18195648	0.046274819	up	141	146	125	83	23	26
ELMOD1	-5.866026872	0.001085596	down	0	0	0	6	6	11
ELMOD2	-1.329210097	1.68019E-15	down	323	420	309	650	587	632
ELMOD3	-2.837049952	1.97931E-06	down	144	152	116	342	1203	444
ELN	-11.8439326	5.2623E-117	down	4	4	9	11195	18719	13743
ELOVL2	-6.530623579	0.000153952	down	0	0	2	6	94	24
ELOVL4	-9.261436921	6.33405E-10	down	0	0	0	136	22	92
ELOVL6	-4.47277246	4.59803E-05	down	194	177	167	252	6863	638
EMB	-12.35013006	7.72629E-19	down	0	0	0	259	575	1215
EMC1	1.288600167	1.24371E-31	up	1895	2044	1867	570	522	596
EMC10	-0.738733856	0.000304038	down	1357	1315	1287	2009	1235	1497
EMC2	-1.396088172	4.07678E-14	down	493	546	426	1116	762	871
EMC3	-1.166916432	1.85942E-05	down	568	592	538	876	1067	727
EMC6	0.642012049	0.049787791	up	480	472	487	260	241	150
EMC8	1.030295322	1.25615E-08	up	826	856	792	290	300	266
EMCN	-12.97394979	2.27079E-24	down	0	0	0	741	1292	1064
EME1	4.894089905	5.7402E-38	up	488	581	468	21	7	9
EMG1	1.085016561	9.37295E-05	up	1074	1172	1159	537	308	305
EMID1	-7.683763724	0.000235849	down	0	0	0	48	31	1
EMILIN1	-7.510123456	5.564E-35	down	33	5	14	2912	1463	2597
EMILIN2	-6.884681455	1.39218E-60	down	4	9	4	488	398	543
EMILIN3	-7.267860742	9.19167E-07	down	0	0	0	14	21	25
EML2	0.889259381	7.36345E-05	up	1256	1457	1382	617	361	612
EML3	-1.002712999	1.01603E-19	down	620	715	626	987	837	968
EML5	-4.043918776	0.002166793	down	5	1	1	63	4	20
EML6	2.574664018	1.42794E-09	up	256	317	310	51	36	18
EMP1	-1.912122129	2.38339E-09	down	1623	1575	1613	3130	5408	4090
EMSY	0.664077005	2.81662E-07	up	876	939	855	405	353	441
EMX2	-2.558021729	0.000482464	down	5	7	4	28	11	29
EN2	5.769964216	5.07784E-05	up	29	33	21	0	0	1
ENAM	-7.200515958	8.57493E-06	down	0	0	0	26	23	8
ENC1	-6.344662844	9.96428E-13	down	4	8	3	182	544	82
ENDOD1	-2.794199367	2.00034E-13	down	404	407	386	1927	1044	3057
ENDOG	1.125986316	0.003729286	up	460	454	408	197	148	84
ENDOU	-3.554329617	0.000288799	down	2	5	1	12	32	20
ENDOV	-0.604778809	0.018753642	down	132	141	142	167	108	178
ENG	-1.829933855	3.4104E-07	down	1749	1914	1623	4283	5840	2920
ENGASE	-0.709630421	0.000350105	down	430	464	424	598	492	439
ENO1	2.561139342	5.5233E-44	up	55753	59444	54694	6137	7303	6827
ENO2	1.366038575	0.009478344	up	2621	2721	2780	1324	281	713
ENO4	-2.7474967	0.048950365	down	1	2	1	11	6	2
ENOX2	-1.179159112	7.31514E-08	down	228	251	256	373	322	494
ENPEP	-10.20388668	5.55547E-12	down	1	0	0	126	575	148
ENPP2	-12.05521668	2.31156E-22	down	0	0	1	1406	872	999
ENPP3	-8.914958991	7.96581E-10	down	0	0	0	53	97	33
ENPP4	-11.99447755	4.67225E-22	down	0	0	0	464	486	650
ENPP5	-9.15840095	9.71046E-11	down	0	0	0	54	42	132
ENPP6	-9.324737978	8.87194E-09	down	0	0	0	137	12	114
ENTPD1	-7.874306403	7.75673E-61	down	10	10	5	1918	769	1562
ENTPD2	1.977561406	4.07427E-05	up	105	146	163	41	14	21
ENTPD3	-8.632959623	2.66179E-10	down	0	0	0	60	60	34
ENTPD5	-0.948542362	0.024218039	down	154	222	176	156	318	264
ENTPD7	2.585367622	2.35965E-43	up	732	840	746	87	96	90
ENTPD8	6.523705654	1.42078E-06	up	41	44	54	0	1	0
EOGT	-2.758112128	4.32009E-08	down	319	393	327	2062	575	2507
EPAS1	-2.156974688	6.38231E-32	down	4270	4531	4390	14920	10358	16958
EPB41	1.446982102	1.06126E-07	up	2215	2457	2123	462	696	584
EPB41L2	-0.864325107	5.1967E-06	down	2816	3175	2827	3271	3743	4329
EPB41L3	-6.067826984	1.33937E-57	down	7	17	11	502	461	703
EPB41L4A	-2.374718065	2.06473E-11	down	75	79	79	210	358	275
EPB42	-5.506707229	0.01952176	down	0	3	0	0	66	20
EPC1	-1.696297311	5.7365E-13	down	275	333	324	939	532	697
EPC2	-1.378230289	6.91447E-09	down	317	365	286	524	497	769
EPCAM	4.815027548	8.92494E-15	up	110	110	97	3	3	2
EPHA1	4.673859804	6.0788E-16	up	187	208	172	4	1	11
EPHA2	2.960563505	2.68312E-37	up	3736	4014	3936	353	407	295
EPHA3	-2.056613902	8.30151E-08	down	190	186	205	362	730	593
EPHA4	-11.40191819	1.95443E-16	down	0	1	0	346	397	1357
EPHB1	2.533189017	6.92893E-07	up	226	165	192	16	37	17
EPHB2	1.519358383	3.19545E-12	up	562	635	580	172	147	120
EPHB3	-1.842712514	0.003382028	down	19	14	15	50	51	20
EPHB4	0.960960714	0.006336686	up	2186	2262	2258	754	1065	579
EPHB6	-9.946106382	1.38418E-11	down	0	0	0	199	33	170
EPHX1	-2.058183894	3.19314E-05	down	942	1021	1002	1380	4310	2792
EPHX2	-2.106254134	8.47058E-13	down	128	143	86	448	339	301
EPM2A	-2.956593594	1.94307E-23	down	42	79	52	350	243	362
EPM2AIP1	-0.615732564	0.000972842	down	858	986	854	1110	728	1124
EPPK1	3.653559084	1.76815E-07	up	967	1020	967	121	12	41
EPRS	2.052909507	4.65133E-42	up	8540	9624	8281	1441	1542	1513
EPS15	-0.927984384	5.09943E-05	down	1200	1323	1255	1526	1368	2241
EPS8	-0.977980021	0.003473398	down	2435	2591	2415	2632	2633	5225
EPS8L1	1.123129455	0.033925574	up	28	42	31	13	8	12
EPS8L2	1.649682379	0.000542078	up	1056	1202	1110	285	100	399
EPS8L3	8.622102167	5.43674E-29	up	665	799	764	0	4	0
EPSTI1	-2.928253196	4.09587E-14	down	28	29	24	197	138	103
EPT1	3.127193153	3.916E-14	up	3486	3754	3269	152	378	303
ERAL1	0.730470238	0.020022967	up	1064	1185	998	405	579	379
ERAP1	-2.339947913	5.59649E-21	down	193	253	210	650	860	818
ERAP2	-3.210649415	2.39467E-05	down	52	51	34	174	85	669
ERBB3	2.169650597	1.27999E-36	up	600	638	607	99	96	96

ERC2	-4.469942392	0.037359005	down	0	0	1	1	11	4
ERCC2	1.805673644	1.83883E-16	up	1118	1238	1065	291	216	188
ERCC3	0.861547571	2.60456E-08	up	1722	1901	1716	793	539	771
ERCC4	1.77563554	6.35115E-20	up	880	922	899	175	201	181
ERCC6	1.02064158	9.73376E-08	up	1034	1119	939	348	293	446
ERCC6L	7.812562819	1.82125E-45	up	816	873	839	4	3	1
ERF	0.880565012	9.65843E-06	up	1633	1791	1528	761	610	536
ERG	-4.831776938	4.67368E-46	down	78	81	99	2320	996	2028
ERGIC1	0.91873896	5.21183E-08	up	6670	7217	6811	3072	1956	2828
ERH	0.830322698	0.000222872	up	4139	4707	4130	2301	1285	1651
ERI1	2.638672033	3.00082E-58	up	1655	1768	1686	178	195	208
ERLEC1	-0.79439297	0.000374044	down	1152	1284	1105	1679	1004	1732
ERLIN1	1.067296252	3.7561E-06	up	1436	1560	1469	468	575	453
ERMAP	-1.404803694	7.24493E-14	down	150	162	133	283	237	319
ERMN	-6.03927793	0.000605177	down	0	0	0	13	7	6
ERMP1	1.407376625	5.74282E-12	up	1162	1417	1358	304	356	388
ERO1A	2.115434942	2.37085E-54	up	4558	4931	4629	810	625	893
ERO1B	-5.10163679	2.34052E-40	down	14	8	19	372	253	391
ERP27	-2.108178031	0.001722149	down	7	6	10	35	14	23
ERV3-1	-1.587815429	0.005436762	down	182	188	171	193	631	283
ERVFRD-1	-4.886882015	0.042064196	down	0	0	0	5	1	6
ERVV-2	9.46058026	6.43458E-14	up	195	167	184	0	0	0
ESCO2	6.176673552	6.12773E-65	up	890	1048	833	9	13	5
ESD	-1.701090187	3.02226E-08	down	1070	1270	1016	3615	1538	2733
ESM1	-7.785841212	0.000206125	down	0	0	0	33	49	1
ESPL1	6.435905923	1.57258E-12	up	1641	1740	1588	14	25	0
ESPN	8.343998653	1.85987E-27	up	605	641	589	0	4	0
ESR1	-1.982064125	1.98911E-11	down	80	87	88	264	157	305
ESR2	2.500482292	0.001750396	up	39	52	32	1	8	6
ESRP2	8.093816585	7.27984E-43	up	855	956	888	2	5	0
ESRRA	1.197999432	0.018796136	up	1373	1471	1421	321	695	256
ESYT3	2.589100228	5.51876E-28	up	304	344	307	46	32	35
ETAA1	0.715848097	0.00027345	up	975	1104	987	396	388	543
ETF1	0.950634568	8.10276E-12	up	5810	6360	5675	2048	2084	2409
ETFA	1.294203546	0.009839517	up	4271	4841	4503	942	2074	777
ETFBKMT	-2.642844051	0.000125577	down	20	13	6	79	63	30
ETFDH	-1.544618839	0.04054701	down	414	431	412	331	1711	405
ETNK2	-2.052630108	0.000800596	down	54	47	56	178	49	253
ETS1	-1.993066844	1.95046E-09	down	911	995	926	1775	3262	2822
ETS2	-1.152873842	6.6599E-19	down	1778	1780	1708	2933	2302	3134
ETV4	6.192646589	6.11822E-48	up	1491	1634	1502	13	24	7
ETV6	-2.000622551	1.32583E-09	down	219	208	224	868	362	663
ETV7	-6.068962336	2.69755E-07	down	0	1	2	78	16	54
EVA1B	-3.923818776	1.34566E-18	down	37	41	51	779	284	360
EVC	-2.757282292	6.79065E-27	down	277	319	291	1449	967	1896
EVC2	-9.23543004	5.40966E-12	down	0	1	0	161	76	234
EVI2A	-10.27858922	1.14758E-14	down	1	0	0	390	138	447
EVI2B	-2.8016739	5.61721E-06	down	66	73	77	373	103	632
EVL	-0.741759258	3.04972E-05	down	666	678	715	944	767	739
EVPL	7.930616212	9.05436E-51	up	971	1066	1038	3	0	6
EVPLL	7.835558082	8.96586E-10	up	116	108	121	0	1	0
EXD1	3.842795878	0.033577358	up	6	11	5	0	1	0
EXD2	-1.041729444	1.64086E-05	down	285	301	257	344	443	434
EXO1	7.104420158	4.33089E-35	up	1897	1927	1872	14	14	1
EXOC1	-1.373507641	1.97539E-13	down	483	611	480	967	776	1162
EXOC2	0.727575205	0.000982556	up	1076	1056	1097	389	458	535
EXOC3	0.618863464	0.00056289	up	1599	1787	1708	794	622	956
EXOC3L1	-6.069372601	2.30634E-08	down	0	3	1	40	102	39
EXOC3L2	-8.83603652	4.39419E-10	down	0	0	0	36	86	52
EXOC6	0.654038679	2.60547E-05	up	577	607	561	293	246	249
EXOC7	-0.604967228	1.19678E-07	down	1468	1630	1455	1696	1409	1827
EXOC8	0.727638801	5.53412E-05	up	726	869	779	408	264	353
EXOSC1	0.782834134	0.00256346	up	610	737	614	365	224	224
EXOSC2	2.631310047	2.1645E-102	up	2073	2287	2128	253	220	272
EXOSC3	1.0102721	7.18571E-12	up	604	621	564	233	181	219
EXOSC4	1.028856015	0.011614297	up	357	351	391	192	116	77
EXOSC5	1.251177514	3.65777E-05	up	420	429	342	168	94	96
EXOSC7	0.927148708	0.000158627	up	849	851	731	401	217	301
EXOSC9	1.011231115	8.79856E-06	up	939	1051	878	444	250	326
EXT1	-1.11324768	0.004127272	down	1006	1078	1068	1818	808	2336
EXTL1	-4.794420682	0.00012976	down	2	1	0	35	14	11
EYA1	-7.245068833	0.003704404	down	1	0	0	0	98	7
EYA2	-10.15190097	1.21866E-14	down	0	0	0	102	127	218
EYA4	3.198082212	0.003401763	up	821	969	782	2	132	52
EZH1	-2.015627676	1.32469E-15	down	482	496	473	1357	983	1878
EZH2	4.432011645	2.7657E-24	up	1762	1709	1642	70	71	25
EZR	2.248727512	3.69625E-10	up	6628	7133	6671	734	1385	857
F10	-3.229338131	1.08818E-09	down	50	47	48	462	114	424
F12	5.671259321	1.576E-13	up	112	137	109	4	1	0
F13A1	-14.98929684	3.24999E-31	down	0	0	0	2509	3937	6276
F2R	-0.923032423	0.009663878	down	1007	965	898	1588	1446	799
F2RL1	1.2292668	0.03530138	up	199	254	234	95	85	25
F2RL2	-1.721767824	0.003158717	down	36	34	32	49	124	58
F2RL3	-7.220872224	0.003770713	down	0	0	0	1	50	2
F8	-4.756140722	3.05632E-13	down	55	71	75	419	2224	1028
F8A1	-0.726857115	0.014028778	down	124	107	133	161	147	120
FA2H	7.841129948	7.54326E-52	up	979	1068	1169	6	3	1
FAAH2	2.323614869	1.17297E-05	up	71	72	85	18	5	10
FAAP24	1.789637211	1.68388E-08	up	170	168	159	42	35	25
FABP4	-15.42320078	2.93843E-13	down	0	2	0	1491	46785	6932
FABP5	-2.80256422	0.000460194	down	33	50	47	75	424	104
FABP6	3.815756329	0.00144619	up	13	26	20	2	0	1
FADD	1.253760788	8.87283E-18	up	832	833	815	280	213	249
FADS1	1.526071335	0.025955809	up	6679	7314	6682	556	3071	1202
FADS2	-2.124606003	2.86805E-09	down	454	522	476	910	1731	1785
FAF1	1.054789958	1.09135E-08	up	1717	1952	1619	560	636	596
FAF2	0.741480197	3.23206E-05	up	1541	1647	1531	593	655	750
FAH	-3.077636115	1.10776E-06	down	179	186	190	503	2013	643
FAHD1	0.844284575	0.033740788	up	752	828	792	263	431	221
FAHD2B	1.247758025	5.90289E-08	up	552	644	568	218	167	142
FAIM2	-8.77321349	7.8381E-12	down	0	2	0	77	196	338

FAM101B	1.131954293	0.047059764	up	1082	1212	1125	234	619	207
FAM102A	2.118164989	2.1384E-26	up	1851	1933	1727	322	319	255
FAM102B	1.730125625	5.27485E-28	up	2914	3163	2791	738	485	691
FAM103A1	-0.656571468	0.004576769	down	159	155	138	162	143	203
FAM107A	-12.88515731	5.83474E-22	down	0	0	0	531	746	1709
FAM107B	0.834048665	0.001891853	up	2671	2754	2578	1495	849	875
FAM109A	1.062168796	3.80547E-05	up	393	426	362	106	131	163
FAM110B	-10.6619987	1.63077E-17	down	0	0	0	265	178	196
FAM110C	1.855637636	0.002493525	up	89	119	146	11	17	42
FAM110D	-7.838239579	1.77271E-18	down	3	1	0	132	247	263
FAM111A	-3.95275586	1.03836E-44	down	77	60	74	1014	556	791
FAM111B	4.406132938	7.02318E-06	up	783	847	791	71	11	2
FAM114A2	-1.291866185	7.77891E-10	down	256	287	243	410	483	462
FAM117A	-7.246680846	2.91582E-29	down	4	0	5	437	207	352
FAM118A	-1.550180277	0.001354694	down	264	327	272	570	262	1005
FAM120A	0.906166588	4.17909E-07	up	4622	5077	4761	1713	1940	1784
FAM122A	-1.83308392	1.28883E-18	down	223	290	249	670	490	780
FAM122B	0.971873402	0.000211823	up	1293	1481	1249	594	309	575
FAM122C	1.283964065	1.39081E-07	up	253	325	242	97	61	82
FAM124A	-7.1066675	4.44836E-14	down	3	0	0	97	100	96
FAM124B	-9.664960861	7.64656E-11	down	0	0	0	43	180	81
FAM126B	-0.904274836	4.24615E-08	down	312	370	291	451	361	484
FAM127A	-1.193195172	8.11795E-24	down	1004	1125	1046	1899	1591	1667
FAM129A	-4.511950503	3.11017E-24	down	354	429	335	6631	2506	9438
FAM129B	2.032024294	1.4736E-28	up	8314	8780	8546	1287	1499	1646
FAM131A	-0.711575849	0.007949901	down	248	246	220	369	198	274
FAM131B	-3.46244538	2.36199E-06	down	3	7	10	75	22	64
FAM131C	4.77253932	9.09255E-21	up	172	193	136	5	4	4
FAM132B	2.117172446	3.80539E-06	up	109	127	107	31	10	16
FAM133A	-2.804230194	0.017242908	down	5	4	8	37	2	50
FAM134A	-0.675959106	0.011321318	down	922	1021	996	906	1282	1103
FAM134C	-0.869254488	6.68468E-09	down	486	538	495	774	553	653
FAM135A	1.021776492	2.5361E-07	up	733	685	678	220	229	285
FAM13B	-1.699260761	1.1985E-06	down	748	806	691	1771	964	2542
FAM13C	-4.255224923	5.17383E-31	down	40	75	37	650	486	926
FAM149A	-7.233972082	1.60175E-29	down	1	2	5	231	244	384
FAM149B1	-2.02964444	7.93011E-25	down	172	145	164	466	409	528
FAM150B	-8.556420698	1.95919E-07	down	0	0	0	107	12	35
FAM155A	-1.369226688	0.010668989	down	33	21	23	65	25	55
FAM155B	-6.70991704	1.04176E-05	down	0	0	0	13	13	15
FAM160A1	3.568586064	1.13016E-25	up	790	736	771	28	48	61
FAM160A2	-0.842555678	0.000355925	down	387	556	364	513	498	644
FAM160B1	-1.043358896	5.07648E-06	down	623	678	600	836	746	1215
FAM160B2	-0.59797046	0.007041841	down	627	639	683	658	567	886
FAM161A	1.621805177	1.16477E-07	up	391	385	342	96	52	114
FAM161B	-1.847862854	6.86084E-07	down	45	92	57	149	132	213
FAM162A	1.172124696	0.002807487	up	2674	2898	2825	1079	1043	492
FAM162B	-9.25790184	5.51772E-10	down	0	0	0	26	106	102
FAM166B	-10.00399847	4.92882E-14	down	0	0	0	94	105	206
FAM167A	6.1453531	4.06822E-17	up	162	159	176	1	2	2
FAM167B	0.964866529	0.012031252	up	157	143	127	58	61	35
FAM171A1	-3.525841623	2.90334E-16	down	53	43	37	239	484	341
FAM171A2	2.759476213	0.013952719	up	288	318	274	11	72	2
FAM171B	-8.304288838	4.82849E-10	down	0	0	1	58	101	78
FAM172A	-0.831821081	5.64851E-06	down	574	572	514	681	582	846
FAM174B	-5.304260276	3.42781E-10	down	1	11	1	108	82	169
FAM175A	-1.811235874	3.69726E-20	down	149	203	158	409	374	486
FAM177A1	-0.822488028	1.28068E-06	down	598	650	620	884	601	882
FAM178B	-3.135122691	0.007666552	down	1	4	1	18	4	16
FAM179A	1.242939565	0.005941432	up	114	118	111	19	38	45
FAM179B	-1.068748831	0.000793443	down	222	237	217	235	357	406
FAM180A	-10.20679036	3.80014E-25	down	0	1	2	973	387	1224
FAM180B	-4.709391604	0.000612191	down	1	3	0	37	4	35
FAM181B	-7.866791767	1.07677E-06	down	0	0	0	48	8	39
FAM184A	-5.376646484	1.40957E-11	down	3	0	3	69	39	73
FAM188A	-1.377032739	6.79345E-11	down	358	436	392	840	523	847
FAM189A1	-5.560105521	0.025944804	down	0	0	1	30	7	0
FAM193A	-0.99373222	2.76327E-11	down	275	306	290	420	358	457
FAM193B	-1.416236059	8.75917E-07	down	335	413	390	885	443	865
FAM198A	-9.00148362	1.96177E-09	down	0	0	1	199	26	183
FAM198B	-12.62909325	9.18817E-46	down	0	0	3	4421	3472	5733
FAM19A2	-4.269222504	3.18109E-05	down	14	11	8	293	10	173
FAM19A5	-10.5361001	8.30869E-12	down	0	0	0	231	32	344
FAM200B	-1.17709841	1.92007E-06	down	119	145	150	270	203	193
FAM204A	-1.046197978	2.17135E-09	down	496	458	436	768	540	749
FAM207A	1.697477384	4.44731E-14	up	717	682	653	156	163	128
FAM208B	1.039836024	3.6297E-11	up	2459	2642	2499	802	821	1000
FAM20A	-9.364068289	2.87889E-13	down	0	0	1	126	172	202
FAM20C	-0.790670146	0.005360973	down	423	487	452	690	569	407
FAM210A	1.132301389	0.006580081	up	585	678	639	145	291	163
FAM210B	-3.781583284	1.2428E-119	down	173	193	160	1628	1654	1836
FAM212A	-2.52336537	1.14181E-07	down	16	12	17	88	43	56
FAM212B	-1.37083128	3.32902E-05	down	140	131	131	246	298	185
FAM213A	-1.626503441	0.049538684	down	1311	1484	1334	611	5943	1929
FAM214A	-1.949351828	1.32344E-09	down	342	396	347	665	979	1312
FAM216A	0.972822604	0.002875062	up	350	369	274	172	93	97
FAM218A	-6.852925254	8.17123E-06	down	0	0	0	21	10	15
FAM219B	-2.17180961	5.30386E-51	down	434	497	428	1488	1205	1672
FAM221A	-1.88211507	0.001249983	down	15	37	19	51	39	96
FAM227A	2.462255529	1.29846E-05	up	180	185	159	8	31	27
FAM228A	-7.049038433	1.65134E-06	down	0	0	1	26	51	21
FAM228B	-4.978739594	1.09014E-25	down	6	10	15	329	156	221
FAM229A	-1.176591631	0.044112124	down	16	21	26	54	21	28
FAM229B	-1.929829266	1.72771E-05	down	112	109	140	520	279	189
FAM234A	-0.621876715	0.001221776	down	871	902	871	859	994	1021
FAM234B	0.989626044	4.19315E-06	up	323	345	336	150	101	110
FAM24B	1.512649061	0.010072224	up	39	64	33	13	6	15
FAM25A	5.362724793	0.003353597	up	15	11	6	0	0	0
FAM26E	-5.383442359	3.83258E-28	down	30	40	44	1207	474	1793
FAM26F	-2.528832327	1.94852E-05	down	9	13	14	74	24	53
FAM3C	-0.895753991	0.001105906	down	499	633	335	707	565	658

FAM43A	-3.945749753	1.49205E-13	down	17	28	21	145	357	193
FAM43B	-7.188343972	1.36222E-05	down	0	0	0	10	31	14
FAM46B	-1.579752972	0.010906491	down	151	188	165	664	109	336
FAM46C	-2.577457855	4.50129E-15	down	63	60	63	368	171	264
FAM47A	5.408814721	0.00153457	up	11	11	11	0	0	0
FAM47B	6.600449594	1.12195E-05	up	16	33	27	0	0	0
FAM47C	8.821745173	4.68337E-12	up	102	124	126	0	0	0
FAM47E	-3.503901525	0.003216967	down	5	1	0	15	20	13
FAM49A	-6.096700083	5.43199E-38	down	2	9	11	305	336	424
FAM49B	1.753886163	2.2158E-19	up	2464	2646	2353	673	441	466
FAM50B	-2.403866452	8.2196E-13	down	59	68	59	311	141	259
FAM53A	-0.975075379	0.006362173	down	28	44	37	55	45	52
FAM53B	-0.772884326	0.000704584	down	393	441	449	509	569	466
FAM57A	1.965199138	1.51006E-10	up	585	644	532	100	133	82
FAM57B	3.269204715	0.039808691	up	14	5	8	1	1	0
FAM60A	3.870528749	4.74835E-06	up	145	65	49	8	1	4
FAM63A	-1.416556009	3.75065E-07	down	183	189	180	272	322	452
FAM63B	-1.558212193	1.74533E-07	down	441	500	494	954	665	1424
FAM64A	6.02455341	1.77492E-19	up	567	700	571	13	7	0
FAM65B	-9.639108171	2.99314E-13	down	0	0	0	77	85	152
FAM65C	-5.749044325	1.68968E-09	down	6	4	4	25	209	293
FAM69A	-3.543021027	4.37965E-66	down	61	57	50	485	447	457
FAM69B	-6.707126932	3.66463E-13	down	7	0	0	162	190	170
FAM69C	-5.982729019	0.007266055	down	0	0	0	10	13	1
FAM72A	3.145477793	1.52623E-18	up	182	158	191	16	11	16
FAM72B	4.903576087	1.44597E-40	up	427	444	382	12	4	14
FAM72C	6.091431929	6.29765E-12	up	125	158	99	4	0	0
FAM72D	4.233055853	2.51224E-18	up	143	172	132	8	2	7
FAM76A	-1.470014396	1.49388E-07	down	125	141	126	348	179	254
FAM78B	-7.327286414	6.80775E-08	down	0	0	1	38	49	34
FAM81A	5.005939535	4.8478E-105	up	1132	1212	1141	28	27	22
FAM83A	12.35278035	5.0816E-24	up	1241	1478	1354	0	0	0
FAM83B	7.400705003	4.75735E-35	up	546	555	561	4	3	0
FAM83D	3.570425775	2.61364E-09	up	1715	1874	1707	157	24	148
FAM83G	3.45391612	9.12647E-39	up	1121	1203	1070	85	44	94
FAM83H	6.435697554	5.07946E-07	up	105	109	33	0	1	1
FAM84A	-8.626292819	2.95034E-06	down	0	0	0	134	7	22
FAM86B2	2.080906869	0.043289697	up	41	39	40	8	0	13
FAM86C1	1.29408113	0.049671548	up	168	209	162	21	88	40
FAM89A	-6.30480167	1.21303E-11	down	5	6	3	82	533	116
FAM89B	-1.780252636	7.5129E-10	down	285	340	320	1055	488	801
FAM8A1	-1.012126602	0.000245165	down	1087	1136	1000	1962	959	1777
FAM92A1	-0.66871933	0.008712219	down	274	283	216	281	318	265
FAM96A	2.055623034	5.00992E-30	up	2248	2319	2137	397	402	340
FAM98A	0.957715914	2.33758E-10	up	2387	2476	2364	963	867	808
FAM9B	8.71710344	1.14536E-11	up	123	110	94	0	0	0
FAM9C	-5.732032939	0.011466889	down	1	1	0	5	63	0
FANCA	5.190870924	1.4602E-101	up	1814	1932	1839	37	43	28
FANCB	4.642545399	1.41943E-52	up	565	685	566	22	9	21
FANCC	2.126288454	1.11751E-07	up	653	604	588	74	142	77
FANCD2	3.591688087	4.44223E-86	up	1142	1263	1183	86	61	65
FANCE	2.349521575	7.64025E-15	up	495	573	508	51	84	82
FANCF	0.719173524	0.000672552	up	299	359	332	143	114	172
FANCG	1.918539571	1.26268E-15	up	748	788	713	191	103	133
FANCI	4.302160498	8.9817E-50	up	2835	3128	2861	126	119	70
FANCL	0.685941407	0.01960162	up	554	643	556	365	176	243
FANCM	1.452950558	7.84678E-08	up	370	421	345	82	114	95
FANK1	1.364557784	4.39367E-05	up	95	101	125	32	26	31
FAP	-2.946162345	1.26773E-11	down	65	109	102	712	506	284
FAR2	-2.606639259	7.4637E-06	down	49	57	68	148	414	161
FARP1	1.279643161	0.011381419	up	1512	1673	1572	218	675	454
FARSA	1.886949767	1.12644E-14	up	2035	2291	2100	414	473	332
FARSB	0.878562921	4.7987E-05	up	1188	1371	1088	452	514	427
FAS	-0.924862641	2.33958E-06	down	299	309	272	443	299	455
FASLG	-4.144948338	0.011909857	down	0	0	3	25	9	5
FASTKD2	-0.599285177	0.019827164	down	617	602	596	673	731	532
FASTKD3	1.971285874	9.73313E-08	up	808	930	762	217	146	89
FAT3	-11.74688289	2.69437E-16	down	0	0	0	471	117	809
FAT4	-2.659032434	6.69162E-17	down	306	323	292	1180	944	2057
FAXDC2	-6.995571274	3.06846E-58	down	16	8	8	950	660	1340
FBLIM1	-4.130366724	1.4447E-11	down	383	438	442	7015	1206	8124
FBLN1	-1.77020225	1.95537E-05	down	5987	6648	6540	21700	6802	19313
FBLN2	-4.639327019	3.7508E-31	down	347	401	340	3380	7127	8465
FBLN5	-6.695847953	1.9911E-36	down	119	190	141	11508	3726	18756
FBN2	5.564305876	1.32572E-13	up	6748	7401	6992	48	218	31
FBN3	5.902833792	0.000268413	up	13	22	12	0	0	0
FBP1	-3.293735906	7.53289E-12	down	10	11	12	111	61	60
FBP2	-5.494769476	0.011854556	down	0	0	0	12	4	2
FBXL12	-1.503109517	9.42835E-09	down	172	162	182	450	246	359
FBXL13	-1.840891407	0.004080487	down	18	10	16	63	23	29
FBXL15	-1.08354253	0.000452358	down	192	183	208	417	221	252
FBXL16	7.877665313	1.24788E-51	up	1028	1167	1103	8	2	0
FBXL17	-1.532252812	8.56925E-23	down	274	276	289	568	533	625
FBXL18	2.102556971	4.29939E-17	up	877	948	916	119	169	161
FBXL19	2.166608735	6.10006E-29	up	958	993	1088	146	154	181
FBXL20	-0.914691631	2.3246E-05	down	356	349	348	435	392	591
FBXL22	-5.76951695	6.29119E-12	down	9	8	7	456	44	473
FBXL3	-1.658535177	1.32395E-09	down	666	736	669	1446	1081	2169
FBXL4	-1.40451512	2.28452E-10	down	241	275	261	591	347	540
FBXL5	-2.630102322	2.72056E-43	down	548	586	540	2390	2647	2260
FBXL7	-8.623903897	1.8095E-48	down	5	3	0	834	549	881
FBXO10	-1.009013827	0.000821258	down	93	121	94	136	168	130
FBXO16	2.017401463	0.001338423	up	63	48	36	14	7	5
FBXO17	-2.090649512	0.014926299	down	9	6	9	19	9	47
FBXO18	-1.048402326	1.72556E-17	down	831	973	887	1310	1205	1436
FBXO2	2.576857328	2.90746E-06	up	982	1068	1063	229	42	113
FBXO22	1.07710942	8.49738E-14	up	976	1187	989	348	333	377
FBXO24	-2.426230058	0.013752158	down	2	3	3	6	13	11
FBXO25	-1.186363891	2.94877E-10	down	310	356	398	562	530	630
FBXO27	-2.521074946	0.000437541	down	21	43	23	41	184	112
FBXO3	-0.953997662	6.46436E-14	down	580	671	572	937	713	865

FBXO30	0.652956186	0.006361456	up	1100	1164	1132	657	353	546
FBXO31	-1.078476596	0.001862947	down	545	574	562	657	610	1280
FBXO32	-5.179461486	1.90232E-27	down	111	112	116	2297	1401	5243
FBXO33	-0.877632848	0.000476031	down	349	374	294	464	314	562
FBXO38	-0.677219787	0.001188514	down	739	691	651	733	665	978
FBXO41	2.948078199	3.52837E-26	up	500	534	513	49	30	65
FBXO42	-1.585847039	0.000724889	down	442	472	439	997	408	1559
FBXO43	3.017992875	7.85825E-05	up	103	150	146	27	4	5
FBXO45	1.837926903	1.27329E-13	up	1150	1151	1013	212	254	185
FBXO46	1.569007428	6.72129E-14	up	472	572	518	135	129	108
FBXO48	-1.675916501	2.96933E-08	down	45	44	35	93	97	90
FBXO5	2.11651314	5.00134E-20	up	627	707	670	136	74	122
FBXO8	-2.311225432	3.32558E-21	down	130	125	141	460	511	416
FBXO9	-0.712100723	3.98209E-05	down	883	971	895	959	1041	1187
FBXW4	-3.034772536	8.57115E-59	down	131	174	145	883	719	1024
FBXW9	0.805107142	0.008184433	up	173	198	143	74	76	57
FCER1A	-8.241219231	2.07365E-06	down	0	0	0	93	9	22
FCER1G	-10.02586043	2.02308E-24	down	0	0	3	970	364	954
FCGBP	-5.097406213	0.000140699	down	0	8	1	82	10	128
FCGR1A	-10.29304306	8.25138E-12	down	0	0	0	361	56	94
FCGR1B	-7.895108054	1.41607E-08	down	0	0	0	38	32	23
FCGR2A	-5.745398943	3.20539E-38	down	11	18	32	1060	532	776
FCGR2B	-10.69260076	9.32852E-17	down	1	0	0	320	323	630
FCGR2C	-10.47254669	1.19297E-15	down	0	0	0	148	131	284
FCGR3A	-12.64820136	4.8658E-23	down	0	0	0	1240	489	842
FCGR3B	-6.232375811	0.002332405	down	0	0	0	3	5	22
FCGRT	-5.683578563	3.725E-86	down	50	71	66	1759	2230	2792
FCHO1	2.384256374	0.021676698	up	428	476	455	10	144	16
FCHO2	-0.949679435	5.01102E-06	down	564	630	584	889	588	988
FCHSD2	-1.743170473	1.70163E-07	down	544	647	546	1292	847	2050
FCN1	-9.119906055	7.49182E-11	down	0	0	0	103	78	36
FCN2	-5.561718936	2.20074E-05	down	1	3	0	15	86	23
FCRL3	-4.535370314	0.037096771	down	1	0	0	1	3	14
FCRL6	-5.496192962	0.005236015	down	1	0	0	14	1	21
FCRLB	-4.370902119	0.003631343	down	1	3	2	3	70	7
FDFT1	2.329414207	1.63454E-18	up	7085	7730	6941	767	1165	1101
FDPS	1.58129243	0.000963825	up	5796	6253	5879	954	2213	932
FECH	-0.811623131	0.000749843	down	393	463	425	504	600	474
FEM1B	-0.828776971	0.000163853	down	1316	1395	1195	2048	1157	1784
FEN1	4.254384169	3.69993E-30	up	3411	3780	3312	176	142	70
FERMT1	2.938047813	0.000959861	up	462	459	398	18	88	8
FERMT2	-1.308084578	2.12996E-05	down	2767	3071	2851	6423	2903	6269
FERMT3	-5.382356028	6.72713E-39	down	11	23	9	554	365	349
FES	-1.813765114	1.51247E-20	down	108	130	122	344	241	319
FEZ1	-9.646019307	1.53512E-40	down	3	2	1	1492	605	1396
FGA	5.656218225	0.000771882	up	11	11	17	0	0	0
FGB	5.54455156	0.001341756	up	11	9	16	0	0	0
FGD1	2.572044287	7.06459E-65	up	1124	1131	1101	136	119	147
FGD2	-6.578931487	4.05535E-16	down	0	7	2	165	121	324
FGD4	-1.65476342	0.000857436	down	395	412	346	442	654	1496
FGD5	-8.32048011	6.20558E-34	down	5	4	0	424	824	752
FGD6	1.612414075	1.29079E-17	up	1265	1400	1187	260	284	348
FGF1	-7.201283	1.80697E-58	down	8	6	2	506	534	627
FGF10	-11.55254556	8.67392E-13	down	0	0	0	174	71	977
FGF13	-9.266466956	1.9423E-15	down	0	0	2	391	142	367
FGF14	-6.678217979	1.88126E-06	down	0	0	2	64	64	16
FGF16	-5.035952696	0.017240814	down	0	0	0	4	3	6
FGF18	-3.5900187	8.09013E-07	down	7	5	6	47	77	26
FGF19	3.795351045	0.039715487	up	4	7	10	0	1	0
FGF7	-8.763950591	1.8902E-34	down	1	4	5	498	1407	1092
FGF9	-8.366380773	7.06059E-08	down	0	0	0	14	52	61
FGFBP1	10.56179799	1.09383E-17	up	379	415	382	0	0	0
FGFBP2	-9.892937903	6.17784E-09	down	0	0	0	17	249	83
FGFR1	-1.57244018	3.7276E-12	down	1473	1654	1482	3075	2441	4299
FGFR1OP2	-1.393975739	3.00279E-10	down	607	619	619	1321	799	1373
FGFR3	3.127904896	4.24855E-15	up	688	848	699	55	85	37
FGFR4	5.939104168	5.68341E-11	up	649	574	608	0	16	4
FGGY	-1.442218452	0.001905452	down	48	77	52	116	146	71
FGL2	-15.41400535	1.4201E-16	down	1	0	0	22390	945	11750
FGR	-2.245772252	1.47885E-08	down	75	87	92	232	385	219
FH	1.32702455	0.019442362	up	2907	3463	3000	510	1493	527
FHDC1	3.321483904	4.93912E-10	up	279	315	306	9	34	19
FHIT	-1.987061636	0.000632823	down	28	19	10	42	66	50
FHL1	-5.247446591	9.55334E-70	down	777	971	832	21281	15306	33824
FHL5	-13.68245979	1.48065E-19	down	0	0	0	792	460	4073
FHOD1	0.872487834	2.88736E-08	up	809	899	893	395	285	334
FHOD3	-9.689730554	8.14662E-18	down	0	0	3	279	1001	415
FIBCD1	5.543232691	0.027535142	up	791	898	868	0	35	0
FIBIN	-15.04842262	1.21091E-15	down	0	0	0	7068	602	6241
FIG4	-1.855389095	1.70785E-16	down	135	163	160	348	355	474
FIGF	-4.481555566	0.006248657	down	0	3	2	1	22	55
FIGN	-1.355998405	0.000439406	down	292	254	202	400	288	693
FIGNL1	2.16395107	1.98969E-31	up	1113	1294	1161	208	192	163
FIGNL2	-7.706538463	1.44632E-07	down	0	0	0	33	32	16
FILIP1	-6.971439256	1.27395E-47	down	13	10	8	677	665	1450
FILIP1L	-16.21088909	6.19128E-29	down	0	0	0	17716	1798	11583
FIS1	-1.470286125	1.75119E-14	down	891	970	870	2250	1577	1565
FIZ1	-1.084970218	6.63512E-06	down	116	142	133	216	145	233
FJX1	1.534331342	0.017236635	up	218	250	238	63	87	18
FKBP14	1.415649195	1.4347E-19	up	1593	1859	1581	417	443	473
FKBP1B	-6.88369766	4.58413E-12	down	0	3	0	112	57	84
FKBP2	-0.693324048	0.012888858	down	593	641	709	1033	560	668
FKBP4	1.628300838	9.39598E-11	up	4072	4329	3732	1270	792	740
FKBP5	-2.666204895	0.000182808	down	961	1153	924	1364	2472	10052
FKBP7	-2.275146514	9.07009E-13	down	214	223	194	921	413	879
FKRP	-1.530974771	8.7672E-11	down	128	129	108	260	190	305
FLAD1	1.257064503	0.002356367	up	851	1026	969	231	399	192
FLCN	-0.655035405	0.017209819	down	592	596	658	616	532	937
FLI1	-12.61656737	6.37919E-25	down	0	0	0	810	775	875
FLII	0.62466781	0.001458957	up	3335	3721	3429	1380	1609	1815
FLJ44635	-3.092867746	0.034751633	down	1	0	3	15	6	4

FLNB	0.989853084	7.41438E-07	up	8017	8650	8306	2721	2463	3792
FLNC	-9.891197885	1.2492E-97	down	6	5	8	3071	4884	4716
FLRT1	-6.268813826	0.000785435	down	0	0	0	5	17	7
FLRT2	-9.921233504	7.017E-30	down	1	2	0	715	598	757
FLT1	-3.55473376	5.42534E-14	down	184	166	199	826	2266	1357
FLT3LG	-3.705034697	2.09459E-12	down	17	17	8	204	80	111
FLT4	-1.41559451	0.016163537	down	160	182	117	131	453	249
FLVCR1	2.820925782	1.26165E-64	up	1326	1457	1180	131	131	135
FLYWCH2	1.870584208	1.22291E-08	up	1132	1227	1129	300	232	143
FMNL1	1.647671747	2.22963E-11	up	1272	1328	1178	368	188	313
FMNL3	-2.533965641	3.73762E-19	down	189	205	189	699	968	692
FMO1	-8.970225138	1.08913E-07	down	0	0	0	30	135	19
FMO2	-15.69401682	1.62081E-34	down	0	0	0	6781	3829	10599
FMO3	-10.25979253	2.50371E-15	down	0	0	0	166	192	116
FMO4	-7.495728974	1.9985E-18	down	1	1	5	491	110	334
FMO5	-5.415510424	2.32201E-17	down	4	3	8	197	74	198
FMOD	-13.94149693	7.57704E-33	down	0	1	1	5239	4196	13273
FN1	-6.516121102	3.5796E-219	down	1397	1423	1507	122379	78293	83027
FNBP1L	1.031502969	0.002485273	up	2129	2310	2140	491	882	879
FNBP4	0.616700296	0.025043455	up	3041	3524	3123	1319	1119	2083
FNDC1	-10.64481612	6.99506E-16	down	0	0	0	312	191	127
FNDC3A	1.183807908	1.49298E-06	up	5383	6046	5328	2006	1138	2171
FNDC5	-10.66141452	2.19431E-12	down	0	0	0	480	66	115
FNIP2	-2.166536284	2.49025E-29	down	332	314	346	972	952	1250
FOCAD	0.764095404	0.04520864	up	847	853	754	236	452	315
FOLH1	-7.55498774	1.83912E-06	down	0	0	0	10	26	37
FOLR1	15.38883553	9.53646E-49	up	39133	41892	39581	1	1	0
FOLR2	-8.066256487	2.06925E-36	down	3	3	2	441	354	744
FOS	-2.8501904	0.000274991	down	337	291	301	2055	2345	237
FOSB	-1.977261315	0.042464774	down	91	83	95	636	109	37
FOSL1	3.376972341	1.38407E-05	up	721	724	723	95	47	7
FOXA1	8.732200236	7.80321E-12	up	114	106	110	0	0	0
FOXC1	-2.000255369	0.001780797	down	689	846	850	4214	567	2278
FOXD3	-3.692119914	0.017229418	down	3	0	0	7	8	13
FOXE1	9.372671583	4.55025E-14	up	315	384	308	1	0	0
FOXF1	3.492407509	3.17461E-23	up	357	482	428	39	14	28
FOXF2	5.600609716	3.93392E-16	up	722	774	799	3	23	6
FOXK1	1.039118494	1.99683E-05	up	1697	1601	1729	595	413	754
FOXK2	1.175976445	7.487E-20	up	1743	1841	1557	554	471	594
FOXL1	1.05608133	0.044025036	up	749	875	767	444	99	300
FOXL2	7.041555679	5.23739E-19	up	236	250	257	1	3	0
FOXL2NB	8.67681619	5.69216E-12	up	200	193	225	0	1	0
FOXM1	5.97025843	6.85148E-20	up	2933	3189	2865	56	39	6
FOXN1	9.973394077	1.22235E-15	up	268	250	262	0	0	0
FOXN3	-2.313525975	1.50666E-11	down	1050	1097	932	3498	2111	5455
FOXO1	-1.355998417	2.23714E-08	down	543	596	594	850	1144	1127
FOXO3	-1.980155951	2.98312E-06	down	659	754	646	1335	1229	3276
FOXO4	-0.975605311	0.000414841	down	412	430	376	505	426	784
FOXP1	-1.691582827	9.93477E-05	down	673	692	691	2166	666	2030
FOXP2	-4.599508695	2.70458E-14	down	13	14	10	297	73	287
FOXQ1	11.27344844	4.03491E-20	up	619	669	637	0	0	0
FOXRED1	1.050228181	0.000272677	up	1017	1009	952	282	414	310
FOXRED2	1.973302668	1.9399E-36	up	1201	1201	1070	244	176	211
FOXS1	-6.687884016	1.42495E-16	down	2	4	1	257	171	80
FPGS	1.240723608	2.62816E-30	up	1767	1951	1752	596	500	549
FPR1	-9.779308408	3.85316E-13	down	0	0	0	186	86	77
FPR2	-5.351578201	0.005727137	down	0	0	0	5	5	6
FPR3	-11.06779293	2.46483E-18	down	0	0	0	383	193	278
FRA10AC1	-0.790533588	5.77589E-06	down	311	384	366	458	358	493
FRAS1	-5.530729972	3.01E-22	down	2	3	5	107	112	108
FRAT2	1.314114577	0.001946431	up	408	419	376	84	74	190
FREM1	-12.10825715	1.10917E-20	down	0	0	0	778	287	710
FRMD3	-2.660236007	8.16628E-07	down	57	80	78	136	422	382
FRMD4A	-6.86844757	1.3754E-107	down	16	11	13	1050	1125	1133
FRMD4B	-2.690488321	2.29368E-16	down	107	160	122	448	503	828
FRMD5	-6.258488447	8.84097E-05	down	0	0	3	16	121	17
FRMD6	-1.943744842	6.92438E-30	down	327	323	301	1042	751	818
FRMD8	1.128624844	0.000105038	up	1049	1055	1031	248	385	374
FRMPD3	-5.587445256	0.002554579	down	0	0	0	8	5	6
FRRS1	2.301320153	1.40041E-12	up	495	510	552	51	88	83
FRS3	-1.208538427	0.00221135	down	43	53	70	124	75	76
FRY	-3.239078964	4.70305E-71	down	237	293	258	2063	1340	1923
FRYL	-1.517665407	4.26903E-06	down	857	797	799	1464	1103	2500
FRZB	-10.13938992	2.21731E-23	down	8	13	0	5084	1070	10976
FSCN1	3.431499479	1.3878E-26	up	8111	8612	8289	534	701	381
FSD1	7.394488107	2.06752E-16	up	231	237	239	2	0	1
FSD1L	1.010618436	0.038395168	up	247	246	196	47	116	73
FSIP1	2.766759897	0.000556623	up	70	47	38	1	7	8
FST	1.840173898	1.33429E-07	up	1003	998	869	142	251	164
FSTL1	-1.529350322	8.83307E-12	down	7727	8188	7346	17561	10875	19896
FSTL3	0.602347275	0.014405251	up	3083	3799	3469	2070	1100	1736
FSTL4	8.289544246	3.55814E-50	up	1212	1380	1355	0	1	8
FTH1	0.848439	2.72197E-05	up	110772	115651	109708	57465	33972	42582
FTL	-2.519711228	7.12065E-85	down	14676	16090	15020	65504	61439	58903
FTO	-0.59166844	5.15696E-05	down	761	796	740	800	724	940
FTSJ1	2.266484347	2.45317E-40	up	2550	2917	2546	488	308	395
FTSJ3	1.423094212	2.03134E-27	up	2235	2442	2047	644	490	654
FUBP1	0.850775577	6.57756E-10	up	3676	4030	3588	1609	1184	1683
FUBP3	1.22115566	9.93625E-11	up	2997	3246	3071	942	733	1185
FUCA1	-2.045766728	7.07825E-44	down	305	358	286	945	800	1041
FUCA2	0.83466787	1.17722E-05	up	2071	2250	2111	947	873	730
FUNDC1	0.749156151	0.000351569	up	499	583	533	279	208	196
FUNDC2	-0.957065313	0.000922336	down	1040	1026	1060	1500	1683	1079
FURIN	1.468149321	1.1544E-05	up	4254	4383	4184	770	1400	1055
FUS	1.018460786	3.39207E-24	up	8534	9318	8626	3244	2650	3423
FUT1	-2.004993258	0.000119716	down	19	23	21	70	73	34
FUT11	1.025699747	1.84959E-16	up	1299	1383	1358	519	399	498
FUZ	-6.138428872	1.1325E-32	down	9	2	3	223	207	275
FXR1	0.798121455	2.76649E-14	up	4873	4879	4538	2096	1778	1973
FXR2	0.852589558	6.64133E-06	up	1533	1696	1446	668	629	531
FXYD1	-8.850305549	1.05606E-50	down	1	3	8	1934	934	1134

FXYD3	2.042715292	1.31197E-07	up	112	110	127	23	23	14
FXYD4	5.756736108	0.000344669	up	14	14	14	0	0	0
FXYD6	-11.33742104	8.86835E-19	down	0	0	0	300	425	271
FXYD7	-4.943947571	0.027764494	down	0	0	0	2	4	6
FYB	-5.440933977	2.41291E-49	down	12	9	13	426	253	385
FYCO1	-2.685928858	1.75271E-18	down	517	515	530	2897	1368	3032
FYN	-0.85305945	0.006648846	down	601	608	582	534	849	890
FZD1	-3.125212792	1.95896E-26	down	199	184	174	1533	711	1277
FZD10	4.735457473	1.47962E-13	up	371	441	457	24	2	9
FZD4	-4.132450104	6.28333E-16	down	439	477	437	2331	8222	5737
FZD7	-1.724378781	6.62714E-15	down	153	186	146	433	278	436
FZD8	-1.786775104	5.27848E-05	down	110	148	135	415	368	168
FZD9	3.731348655	0.001897272	up	93	118	152	0	16	2
FZR1	0.637291493	0.000558141	up	648	647	728	358	257	316
G2E3	0.8422753	2.47862E-05	up	634	659	595	221	228	299
G3BP1	0.995589181	1.61115E-12	up	5126	5809	4977	1779	1834	2031
G6PC3	0.758208213	0.033123138	up	914	982	923	298	517	343
G6PD	0.796236476	0.042665794	up	2264	2332	2239	632	1268	826
GAA	-2.065231931	7.74442E-21	down	315	360	322	1138	687	1174
GAB1	-3.196872888	1.01043E-31	down	188	190	171	1367	757	1511
GAB2	-1.069214139	0.000511997	down	797	850	741	898	982	1690
GAB3	-9.839406092	3.26559E-15	down	1	0	0	219	239	238
GABARAP	-1.556842189	1.40358E-09	down	2370	2587	2233	7007	4024	4112
GABARAPL2	-1.160466152	6.08482E-11	down	1318	1368	1247	2609	1705	1975
GABBR2	5.275436341	0.002902767	up	9	9	12	0	0	0
GABPB1	1.375490588	6.15665E-09	up	769	797	775	288	160	200
GABPB2	-1.345934915	3.95202E-08	down	163	198	165	403	226	330
GABRA2	-7.722335022	2.21285E-07	down	0	0	0	21	17	46
GABRA3	9.958420395	1.35957E-15	up	267	248	257	0	0	0
GABRA4	-9.357486583	8.61253E-06	down	0	0	0	3	212	19
GABRB1	-5.659261061	0.022165748	down	1	1	0	0	63	1
GABRB2	-4.700332988	0.006134587	down	0	0	2	5	7	26
GABRP	-4.918009805	0.03181834	down	0	0	0	7	3	2
GABRR1	-6.528612198	0.020415298	down	0	0	0	12	0	26
GAD1	3.025884411	0.002077681	up	339	365	353	62	1	34
GADD45A	-2.293271602	1.22556E-10	down	352	423	391	1592	703	1853
GADD45B	-3.079363728	1.76852E-13	down	217	252	227	1181	1998	874
GADD45G	-10.09554605	1.15013E-14	down	0	0	0	111	110	211
GAGE12H	6.096019303	7.54292E-05	up	19	16	18	0	0	0
GAGE2A	8.161861085	1.12637E-09	up	60	105	60	0	0	0
GAL	9.699068314	8.37932E-29	up	1154	1258	1104	0	3	0
GAL3ST1	3.081232617	8.93013E-10	up	90	107	97	9	3	13
GAL3ST4	-4.380189082	1.47401E-18	down	7	7	14	196	96	130
GALC	-12.02820424	2.15024E-22	down	0	0	0	503	487	650
GALE	3.158590222	1.58767E-19	up	1472	1686	1517	97	169	97
GALK1	1.031520508	0.002749226	up	448	520	495	259	114	143
GALK2	0.904552393	0.000485585	up	569	587	548	186	250	203
GALNS	0.997143197	1.73947E-06	up	725	832	785	239	288	301
GALNT1	2.071781454	3.38716E-20	up	6133	6789	5845	911	1197	1021
GALNT11	-0.654143726	8.67371E-05	down	675	702	633	698	744	796
GALNT13	1.583611729	0.002438148	up	48	52	63	10	9	20
GALNT14	-5.693196621	0.000690997	down	0	0	1	9	22	7
GALNT15	-12.90619931	1.81379E-20	down	0	0	2	1005	1454	8817
GALNT16	-4.080947977	1.30288E-41	down	20	27	33	295	288	380
GALNT18	-3.259406116	1.88865E-32	down	72	87	62	638	459	404
GALNT2	1.066105722	0.001044825	up	6542	7451	6746	2208	1465	3468
GALNT3	2.954663987	2.6638E-10	up	351	396	353	17	27	57
GALNT5	-9.3232976	2.14291E-12	down	1	0	0	130	105	261
GALNT6	-5.822113439	1.21243E-10	down	0	1	3	67	51	43
GALNTL6	3.998675621	1.6935E-07	up	39	66	52	4	1	2
GALT	-1.571069623	3.08899E-11	down	146	182	157	429	248	356
GAN	1.273383563	7.72921E-06	up	134	136	133	38	32	49
GANAB	1.34873577	3.12946E-20	up	11371	12879	11563	3105	3018	3860
GANC	-1.383653326	3.88512E-06	down	191	261	170	404	259	498
GAP43	-10.35152393	1.47156E-12	down	0	0	0	332	128	60
GAPDH	1.626946135	2.55287E-09	up	84746	92144	84613	19665	24025	15511
GAPT	-7.4407545	1.8898E-06	down	0	0	1	87	12	39
GAPVD1	1.249468429	1.49296E-18	up	2580	2773	2289	734	702	844
GAR1	1.549518634	9.05323E-25	up	1010	1078	922	278	221	232
GAREM2	-1.594719629	4.76845E-05	down	37	37	63	111	97	87
GARNL3	-1.989043874	1.03814E-05	down	108	102	95	469	141	274
GARS	1.879716133	2.69485E-16	up	4226	4797	4221	856	964	705
GART	2.364859324	9.48947E-13	up	3324	3777	3358	326	606	480
GAS1	-3.269471815	1.58991E-12	down	221	206	204	884	945	2555
GAS2	-3.481562912	0.000134835	down	4	2	2	14	31	17
GAS2L3	2.623664882	2.02005E-21	up	1025	1050	899	131	127	82
GAS6	-4.409950573	9.50415E-19	down	445	409	454	8316	2251	9912
GAS7	-4.947251443	2.68215E-24	down	67	86	80	780	1581	2718
GAS8	0.70276029	0.000203323	up	611	579	593	312	208	265
GATA6	-2.045113687	2.75015E-29	down	225	237	218	696	679	609
GATAD2A	1.465488265	1.24249E-39	up	2395	2587	2471	645	582	692
GATAD2B	1.401743096	0.001623084	up	53	77	49	15	13	20
GATM	-6.901319349	6.473E-26	down	0	5	4	284	156	333
GATSL2	1.583927832	9.46176E-09	up	892	849	848	166	169	282
GATSL3	-3.028701684	9.02068E-19	down	26	22	26	136	160	130
GBA2	-1.405325334	5.84072E-15	down	381	370	397	698	733	723
GBF1	0.992385977	1.15909E-06	up	2144	2461	2414	711	814	972
GBGT1	-9.794985751	1.74648E-14	down	0	0	0	122	84	146
GBP1	-9.861437093	1.33075E-22	down	0	0	3	1128	379	532
GBP2	-5.486952997	6.05893E-42	down	70	93	85	3645	1223	3231
GBP3	-2.612226207	1.2773E-27	down	126	117	141	674	523	477
GBP4	-8.410139552	3.87601E-27	down	2	2	2	247	579	598
GBP5	-7.30013866	9.28893E-11	down	0	2	0	83	63	78
GBX2	3.262133947	0.001812254	up	20	41	23	2	4	0
GCA	-5.511367519	9.05682E-47	down	7	14	10	417	245	350
GCDH	-0.953523418	0.004037374	down	329	319	313	388	558	353
GCGR	5.906133943	1.1819E-06	up	38	58	73	1	1	0
GCK	-8.180300826	1.66957E-08	down	0	0	0	34	20	62
GCKR	-2.994609416	0.014095322	down	8	7	3	7	76	11
GCLC	1.163966693	0.001653092	up	1484	1701	1598	372	684	422
GCLM	1.883392096	1.22092E-12	up	1448	1473	1444	234	335	260

GCN1	1.801886162	9.24021E-31	up	5392	6000	5436	1128	937	1379
GCNT1	-2.237024357	1.18983E-14	down	41	61	50	147	170	188
GCNT2	1.189616293	0.000510914	up	1039	1027	930	457	179	317
GCNT3	9.30526665	2.82938E-26	up	826	927	910	3	0	0
GCNT4	-4.957141802	1.16057E-11	down	1	3	3	46	41	68
GCSAM	-5.586224087	0.003027585	down	0	0	0	9	5	5
GDA	11.7611303	7.58658E-90	up	11239	12113	11033	0	7	0
GDAP2	0.649429459	0.000194165	up	563	578	512	292	225	233
GDE1	0.942364667	0.000345806	up	3118	3437	3144	1024	1420	1085
GDF10	-9.746755391	1.82322E-11	down	0	0	0	152	33	164
GDF11	1.647913162	2.8253E-13	up	781	885	812	181	211	164
GDF15	3.622566957	1.83041E-07	up	442	532	521	57	24	6
GDF5	-3.495914313	0.043592979	down	1	0	2	4	17	2
GDF6	-4.811814242	2.49628E-05	down	0	5	5	50	119	19
GDF7	-7.561385446	8.44315E-08	down	0	0	0	30	17	28
GDPD1	1.650813473	1.80954E-08	up	180	199	141	42	30	46
GDPD3	-2.144936485	4.65063E-09	down	30	36	28	124	63	112
GEM	-3.590203897	5.31432E-15	down	369	351	375	5206	1308	3174
GEMIN2	0.664782817	0.03062621	up	349	390	317	205	153	114
GEMIN4	1.652407878	2.81583E-32	up	1189	1201	1163	268	238	299
GEMIN5	1.553813665	2.27327E-17	up	1220	1332	1161	272	314	306
GEMIN6	0.774486787	0.008333316	up	305	271	227	133	111	89
GEMIN7	1.480582502	1.1672E-14	up	418	410	406	111	104	99
GEMIN8	-0.762784755	0.000770649	down	277	370	326	455	286	441
GEN1	2.118541109	1.04463E-09	up	714	771	699	121	152	78
GET4	0.885159664	1.96975E-05	up	967	1052	990	494	289	386
GFAP	-7.074871189	1.16047E-09	down	1	0	4	343	112	41
GFM1	0.942279198	0.037101597	up	2121	2409	2053	514	1191	647
GFPT1	0.715013398	0.002890674	up	3903	4291	3765	1428	1930	1752
GFPT2	1.120755514	0.048902445	up	1278	1490	1354	175	396	772
GFRA1	-12.74503258	2.30168E-20	down	0	0	0	1254	271	1265
GFRA2	-3.404892067	0.000872129	down	6	6	6	82	5	55
GFRA3	-2.443479226	7.98114E-05	down	6	5	11	25	30	30
GGACT	-1.535455722	0.000257809	down	25	31	30	71	63	42
GGCT	1.24683227	0.002229605	up	1594	1843	1547	453	688	310
GGH	3.11415041	1.62857E-25	up	1161	1294	1076	106	114	66
GGN	-5.09680562	1.8191E-06	down	1	2	1	60	18	21
GGPS1	0.657898959	0.000145677	up	1345	1537	1282	754	553	570
GGT1	4.521817947	1.82431E-26	up	1433	1660	1484	56	18	71
GGT5	-8.440503386	2.12433E-57	down	4	10	4	1208	1918	1184
GGT7	-8.012361096	1.5553E-32	down	1	0	7	547	345	606
GHR	-10.40032936	1.3635E-19	down	2	0	0	501	881	493
GIMAP1	-11.23960856	2.64532E-18	down	0	0	0	216	295	435
GIMAP2	-10.48862989	2.46689E-16	down	0	0	0	165	142	261
GIMAP4	-10.35465493	6.32249E-29	down	0	3	0	685	902	1176
GIMAP6	-12.86984066	4.07739E-23	down	0	0	0	553	1128	1210
GIMAP7	-12.31952133	7.99205E-20	down	0	0	0	286	798	884
GIMAP8	-12.9994101	8.58611E-24	down	0	0	0	677	1386	1076
GIN1	-1.076509013	0.001126988	down	136	147	115	277	127	201
GINM1	-1.89967039	1.78334E-18	down	446	495	389	1141	909	1495
GINS1	4.105287459	2.32028E-16	up	504	529	505	31	25	7
GINS2	5.330421939	1.11274E-08	up	852	934	780	28	17	0
GINS4	5.4565014	1.47654E-48	up	1009	1051	1015	14	25	10
GIPC1	1.582648284	2.08262E-40	up	2705	2920	2763	754	592	648
GIPC2	-8.088207238	5.20279E-13	down	2	0	0	113	112	163
GIPC3	2.884821453	5.97969E-42	up	1085	1295	1083	96	121	113
GIPR	2.959413129	0.000486546	up	242	239	275	5	48	12
GIT1	1.105768692	3.55878E-05	up	1347	1514	1447	391	560	450
GIT2	-0.956022338	9.69356E-07	down	595	754	698	853	846	1119
GJA1	-11.29982977	1.79305E-51	down	1	8	1	6117	2646	9273
GJA4	-10.77342895	2.63887E-21	down	2	0	0	643	1113	676
GJA5	-11.52010424	2.39772E-20	down	0	1	0	807	804	615
GJB3	9.652026594	1.70268E-19	up	723	760	774	1	0	1
GJB5	9.114683422	6.17625E-13	up	152	136	142	0	0	0
GJC1	1.180797431	1.28291E-09	up	1386	1476	1479	500	333	542
GJC2	1.627113209	5.03739E-05	up	170	185	153	53	19	47
GJD3	-5.162190663	6.08774E-06	down	4	1	1	44	90	13
GK	1.70326435	3.94164E-15	up	473	486	452	92	111	103
GKAP1	-1.21518549	4.91572E-08	down	92	108	90	154	159	163
GKN1	4.728137019	0.002637878	up	16	12	12	0	1	0
GLA	2.335704223	9.7816E-29	up	1499	1753	1495	285	197	187
GLB1	1.460878765	3.48898E-28	up	1754	1846	1658	438	422	496
GLB1L	-0.735379041	0.037602318	down	71	80	86	86	68	129
GLB1L2	4.263630301	5.47551E-33	up	644	699	556	18	33	18
GLCCI1	2.378655033	8.39644E-38	up	1007	1080	966	145	146	124
GLDN	-7.709190221	2.10469E-22	down	1	4	4	635	140	605
GLE1	1.113318753	1.08413E-09	up	1554	1692	1372	622	398	506
GLI1	-2.21052413	5.98104E-05	down	9	11	15	55	25	37
GLI2	0.924119159	0.039366125	up	389	284	327	200	101	79
GLI3	-0.9006957	0.004834942	down	131	165	180	172	180	281
GLI4	-0.991265234	0.004260454	down	81	115	117	144	172	120
GLIPR1	-7.198986671	1.45836E-25	down	29	17	27	4031	528	3459
GLIPR1L2	-5.131772528	3.35743E-06	down	4	1	1	101	37	15
GLIPR2	-4.377152927	5.19295E-34	down	55	49	48	1144	559	573
GLIS3	1.183372864	3.69828E-06	up	767	748	706	185	212	298
GLMN	1.386576598	2.31212E-07	up	427	467	335	98	124	108
GLMP	1.113427956	2.28825E-17	up	1474	1524	1419	472	452	526
GLO1	0.969680048	1.95144E-17	up	4830	5224	4304	1900	1513	1804
GLP2R	7.52649323	1.14509E-28	up	2672	2727	2503	2	21	6
GLRB	-10.09535309	5.78928E-14	down	0	0	0	94	108	230
GLRX3	1.206141386	5.26494E-08	up	2346	2507	2209	709	813	628
GLS2	-6.495511971	5.96493E-05	down	0	0	0	15	7	14
GLT1D1	-5.785550739	0.000823679	down	1	1	0	64	2	16
GLT8D1	-0.914107825	6.82951E-06	down	528	636	564	972	587	773
GLT8D2	-2.447119611	1.71121E-06	down	304	315	316	2133	463	1153
GLTPD2	5.002133078	0.001605889	up	21	10	17	0	1	0
GLTSCR1L	-1.465977487	5.17771E-08	down	266	317	290	607	379	749
GLTSCR2	-2.092607218	1.08523E-13	down	1440	1611	1532	6680	3094	4337
GLUL	-3.902239434	1.9494E-16	down	1375	1545	1349	6525	19800	17838
GLYAT	-9.486601964	0.026660839	down	0	0	0	0	212	47
GLYATL2	-6.273218135	0.000557092	down	0	0	0	14	12	4

GLYATL3	4.534157718	0.027763093	up	7	6	5	0	0	0
GLYCTK	-3.64432161	3.69788E-05	down	64	76	64	148	1310	218
GLYR1	0.66282999	0.000989242	up	2625	2625	2413	984	1110	1335
GMEB2	1.032814597	6.4654E-06	up	569	656	631	200	170	278
GMFG	-8.706001077	8.28515E-27	down	1	1	2	491	395	298
GMIP	1.370194339	5.22935E-14	up	459	501	490	147	104	150
GMNC	-5.458949278	0.032117393	down	0	0	0	2	13	1
GMNN	2.834634445	8.95818E-45	up	1607	1642	1563	143	135	203
GMPPB	0.796200779	5.39205E-06	up	333	378	362	145	135	159
GMPR	-7.68392478	1.67126E-24	down	3	1	2	183	285	407
GMPR2	-1.705834226	3.11439E-12	down	337	410	317	1022	557	914
GMPS	2.019644556	2.97423E-57	up	4303	4448	3941	804	610	821
GNA11	-0.632005923	2.89499E-07	down	1075	1176	974	1204	1097	1244
GNA14	-7.474933315	6.11492E-12	down	1	0	2	94	200	74
GNA15	-3.889885926	3.07669E-09	down	14	8	3	131	51	87
GNAI1	1.168838253	2.385E-10	up	1679	1885	1629	488	569	571
GNAI2	-0.852435762	4.04418E-12	down	3478	3740	3494	5010	3732	5083
GNAI3	1.22979804	2.31829E-20	up	3926	4387	3788	1428	1075	1169
GNAO1	1.15713038	0.026787344	up	341	403	409	99	189	67
GNAQ	-1.251446777	7.89866E-06	down	1465	1538	1414	3107	1514	2988
GNAZ	-0.981593126	0.001496254	down	78	75	72	103	119	91
GNB2	0.706440026	1.11753E-06	up	4218	4624	4386	2298	1636	1852
GNB3	-3.862135869	6.1719E-08	down	8	7	4	108	26	68
GNE	0.935882507	1.89934E-09	up	1443	1705	1536	552	512	676
GNG11	-0.934542315	0.0024505	down	1299	1567	1309	1938	2268	1363
GNG2	-7.324002838	6.0968E-37	down	4	7	2	335	587	524
GNG4	3.517683626	0.003982518	up	20	15	14	0	2	1
GNG7	-2.866759401	4.84487E-16	down	19	28	31	139	145	117
GNG8	-8.775572483	2.63927E-08	down	0	0	0	110	14	55
GNGT1	5.655810411	0.000644826	up	15	11	13	0	0	0
GNGT2	-3.422324528	6.79806E-05	down	8	1	2	30	25	30
GNL2	1.188331633	1.53688E-06	up	1877	1980	1659	786	448	500
GNL3	1.318909725	7.7837E-13	up	2537	2758	2551	779	785	655
GNL3L	2.515414089	2.70728E-45	up	2350	2478	2146	261	300	300
GNLY	-7.129165376	4.88281E-07	down	0	0	1	37	46	22
GNPDA1	1.238915295	3.62405E-18	up	2295	2283	2181	789	607	645
GNPDA2	-0.709910258	0.002318712	down	375	347	347	489	408	345
GNPNAT1	1.493420137	1.90504E-10	up	1890	2131	1786	408	420	638
GNPTG	-1.634893047	6.25992E-17	down	357	371	338	982	602	790
GNRH1	-2.591818572	0.00014527	down	23	23	17	117	25	137
GOLGA2	1.445155049	2.39566E-24	up	4164	4731	4200	1132	955	1340
GOLGA4	0.930005447	4.51382E-06	up	5616	5934	5620	1818	2239	2297
GOLGA7	-0.782830151	1.57764E-09	down	818	864	801	1165	876	1005
GOLGA8B	-0.917421702	0.018091157	down	597	608	602	1207	420	860
GOLGA8H	-4.32952645	0.003303525	down	3	0	3	74	4	14
GOLGA8K	-2.345041626	0.000222398	down	7	7	5	21	17	31
GOLGA8M	-5.134550002	0.038927258	down	0	0	0	1	9	3
GOLGA8N	-2.079981658	0.00021162	down	25	39	37	172	46	93
GOLGA8S	-1.602713624	0.011329294	down	11	11	6	24	13	24
GOLM1	2.781051696	5.53973E-22	up	4483	4969	4356	341	560	503
GOLPH3	0.862296544	6.45609E-14	up	3902	4240	3767	1629	1305	1737
GOLT1B	1.005722132	2.13119E-10	up	1943	2087	1905	854	584	673
GOT2	1.588412312	0.000205823	up	3980	4156	3892	639	1391	722
GPALPP1	-1.170739476	2.28633E-09	down	293	352	276	516	381	583
GPAM	-5.407737727	5.15053E-07	down	445	481	466	789	32471	5264
GPAT2	-6.181391113	3.12161E-09	down	5	0	0	61	115	79
GPATCH4	1.731520625	5.51568E-12	up	1555	1528	1353	272	368	297
GPBAR1	-6.82571792	5.25747E-08	down	1	2	0	38	151	37
GPC1	1.098395588	5.56079E-12	up	2194	2395	2210	780	770	694
GPC2	3.558530862	0.000235362	up	135	131	111	17	6	0
GPC3	-8.611620339	5.25343E-15	down	0	3	1	91	552	413
GPC4	-6.651951974	1.51883E-37	down	15	21	10	1222	477	1652
GPC5	1.616670498	0.000192761	up	56	77	90	19	13	20
GPC6	-12.74896729	1.6944E-24	down	0	0	0	1196	631	910
GPCPD1	-2.933234757	5.5766E-26	down	123	155	154	1086	598	683
GPD1	-8.134341439	9.14744E-07	down	39	60	51	174	25084	1744
GPD1L	-1.670714913	7.09903E-17	down	645	762	717	1369	1572	1839
GPD2	1.541249347	3.60832E-15	up	1720	1900	1606	377	453	434
GPI	1.468156733	0.001830709	up	7891	8378	8014	1378	3208	1426
GPIHBP1	-10.88741408	5.78033E-14	down	0	0	0	72	325	327
GPKOW	1.494689901	5.69661E-12	up	1372	1601	1377	477	267	363
GPLD1	-1.938287399	0.000937227	down	94	97	80	401	77	286
GPM6A	-9.591876574	6.27818E-11	down	0	0	0	113	30	170
GPM6B	-8.49566377	9.58612E-42	down	1	7	1	894	483	946
GPN1	1.025236233	3.2382E-08	up	1136	1298	1155	482	408	359
GPN2	0.627240768	0.004890098	up	327	363	356	140	163	176
GPNMB	-2.146216407	8.15384E-18	down	1511	1643	1609	4975	3454	6742
GPR107	0.804809048	2.10226E-05	up	3723	3851	3558	1464	1204	1889
GPR132	-4.045455338	9.27539E-05	down	2	0	8	54	24	44
GPR137	0.928021009	5.81043E-05	up	640	614	661	240	261	210
GPR137B	-10.65546053	1.02516E-17	down	0	0	1	385	434	404
GPR137C	-2.52951305	0.000263282	down	15	13	3	33	48	44
GPR141	-4.579700353	0.000513986	down	4	0	1	57	7	26
GPR142	-5.808990888	0.004277448	down	0	0	0	4	13	4
GPR143	5.101580449	0.006212578	up	7	13	7	0	0	0
GPR146	-3.856807421	6.73528E-14	down	31	23	29	144	377	317
GPR153	0.952477595	0.000230685	up	370	450	390	147	168	124
GPR155	-2.235751501	1.06717E-15	down	90	87	128	346	280	405
GPR156	6.04409162	1.74006E-15	up	292	336	291	0	1	9
GPR157	2.784313647	2.66332E-12	up	208	223	248	16	32	21
GPR158	3.807487596	6.35783E-05	up	126	87	74	4	10	0
GPR160	1.21416197	0.014780765	up	152	171	168	32	75	39
GPR161	-0.600521972	0.03031171	down	328	386	326	414	245	475
GPR162	-5.012052957	1.1111E-18	down	4	3	5	90	103	80
GPR17	5.432851039	0.000264991	up	15	30	21	1	0	0
GPR171	-6.808042993	6.99354E-06	down	0	0	0	13	13	18
GPR173	-1.889099365	0.000182968	down	16	28	39	70	51	100
GPR18	-5.208977532	0.04003911	down	0	0	0	12	2	1
GPR180	0.778011882	0.021663505	up	719	810	709	221	384	304
GPR183	-4.963230573	6.43416E-11	down	8	3	2	143	41	114
GPR19	3.64167009	0.000295073	up	27	42	21	2	3	0

GPR21	-4.953697691	1.04387E-08	down	4	6	3	177	23	96
GPR27	-5.71693263	0.001968029	down	0	0	0	9	4	8
GPR3	2.790566151	0.01372464	up	71	87	84	1	20	2
GPR32	7.633702121	1.19856E-08	up	57	48	49	0	0	0
GPR34	-4.59209519	7.76101E-33	down	34	29	28	477	332	776
GPR37	3.246879723	1.18582E-10	up	503	519	543	15	50	50
GPR37L1	3.296013478	3.53299E-07	up	50	70	59	8	3	2
GPR39	4.323816797	1.27065E-08	up	90	84	107	0	3	7
GPR4	-9.995006493	3.82942E-13	down	0	0	0	113	197	78
GPR63	-2.001789202	0.000515012	down	12	20	13	36	26	67
GPR65	-9.442670494	4.39782E-11	down	0	0	0	156	34	92
GPR68	-2.01010693	0.022111359	down	5	12	16	12	49	30
GPR75	1.892477993	0.011367958	up	39	47	33	3	13	6
GPR82	-7.635302502	0.0002079	down	0	0	0	45	1	36
GPR83	-3.672108002	0.047285314	down	0	1	1	1	5	12
GPR84	-5.33924798	0.006528965	down	0	0	0	5	4	7
GPR85	-6.570356199	2.07365E-06	down	1	0	0	29	20	24
GPR87	5.558944034	0.001256816	up	8	17	12	0	0	0
GPR88	-7.089558497	0.000251823	down	0	0	0	8	37	5
GPR89A	0.885274218	0.037961603	up	157	302	195	77	104	64
GPRASP1	-7.112053609	5.76028E-37	down	11	12	5	1272	388	1159
GPRC5A	4.289921298	2.6247E-07	up	7532	7837	7417	408	23	437
GPRC5B	-2.910298236	2.30368E-16	down	169	167	131	531	861	1079
GPRC5C	-0.738556821	0.011685617	down	460	513	493	766	362	640
GPRIN1	5.620139971	2.14117E-20	up	461	484	478	6	13	1
GPRIN2	7.173287618	1.02771E-35	up	487	590	540	6	0	2
GPRIN3	-3.834852362	1.21432E-14	down	17	34	33	183	387	256
GPS2	-0.695592834	9.63711E-07	down	523	593	583	667	567	726
GPSM1	2.220040759	3.05875E-32	up	1439	1673	1528	214	251	237
GPSM2	4.059259277	6.69485E-26	up	3123	3349	3041	133	67	214
GPX3	-12.9678616	1.20221E-55	down	3	0	6	8475	11858	32210
GPX4	-1.541204425	8.92978E-06	down	1853	2135	1862	3643	5307	2857
GPX7	-10.16081665	5.60001E-14	down	0	0	0	171	190	80
GRAMD1B	4.802334754	4.09357E-27	up	2067	2272	2299	30	85	49
GRAP	-2.263306743	0.000803644	down	19	9	15	44	72	28
GRAP2	-5.020658762	9.88329E-05	down	0	3	0	37	14	18
GRASP	-4.643143353	5.44683E-22	down	19	30	30	737	411	259
GRB7	5.834432682	1.29024E-44	up	547	480	486	7	3	9
GREB1	1.982698672	0.000431559	up	208	237	184	17	59	33
GREB1L	1.752150584	0.004097313	up	696	758	662	115	51	293
GRHL1	3.618665271	8.88218E-11	up	191	215	197	11	19	4
GRHPR	-1.076151499	1.97017E-08	down	706	738	638	1163	1045	897
GRIA1	-7.996613039	1.64664E-08	down	2	2	2	360	3	781
GRIA2	-11.19838452	6.02997E-07	down	1	0	0	588	11	1285
GRIA3	-10.00629651	3.14382E-12	down	0	0	0	234	44	140
GRID2	-6.071522733	0.001560944	down	0	0	0	3	10	13
GRIK2	1.699246737	0.009759723	up	165	185	181	23	69	19
GRIK3	-7.921086608	3.07067E-07	down	0	0	0	14	47	31
GRIK5	-5.931019672	4.39616E-12	down	4	6	4	307	36	287
GRIN2C	-3.783858718	0.000123721	down	1	2	2	13	21	14
GRIN2D	4.636541707	1.35085E-45	up	369	385	366	10	11	11
GRIN3A	-6.503182868	0.001342864	down	0	0	0	2	20	12
GRIN3B	2.99477914	0.000860398	up	34	34	31	7	1	1
GRIP2	-1.20313637	0.005232802	down	73	73	96	96	173	120
GRK3	-5.012451029	9.1754E-09	down	34	63	38	205	2156	488
GRK4	-2.762600899	1.89492E-09	down	12	8	15	54	57	58
GRK5	-2.892442265	3.36274E-13	down	355	426	367	1748	1117	3295
GRK7	2.92081163	0.011704971	up	24	16	22	1	0	5
GRM5	-2.826019375	0.025778913	down	2	0	3	9	5	12
GRM7	-6.9183061	0.001594078	down	0	0	0	39	1	10
GRN	0.93978284	1.66751E-08	up	6198	6704	6426	2284	2002	2903
GRPEL1	0.844013981	0.017332171	up	1251	1367	1269	443	676	385
GRTP1	2.731437341	1.98368E-16	up	288	362	297	46	33	22
GRWD1	0.740062707	0.022806891	up	679	682	656	360	302	192
GS1-259H13.2	-3.34083433	0.001827012	down	1	4	2	32	9	10
GSAP	-9.826531024	2.71081E-15	down	0	1	0	270	195	232
GSC	-6.236051893	0.000270392	down	0	0	0	9	6	15
GSDMA	-6.506809524	0.000718912	down	0	0	0	23	10	3
GSG1L	-8.254713485	7.22457E-08	down	3	0	0	198	13	481
GSG2	6.073685672	7.47971E-34	up	400	435	401	7	6	0
GSK3B	1.125812519	1.19143E-05	up	2655	2989	2638	694	999	969
GSN	-5.469083202	1.82717E-48	down	1623	1647	1687	55487	26002	78409
GSPT1	2.030114156	5.53783E-46	up	8090	8648	7826	1357	1434	1460
GSPT2	-8.800466377	4.43071E-16	down	0	0	2	215	201	217
GSR	2.069519918	2.06971E-12	up	2380	2493	2220	333	502	343
GSS	1.777922395	6.83163E-47	up	1681	1801	1532	365	305	370
GSTA4	-2.982065426	1.92404E-24	down	116	117	75	725	403	619
GSTCD	1.397368076	4.84772E-10	up	397	428	355	98	88	133
GSTK1	-1.140608494	7.63454E-08	down	1011	1074	892	1537	1698	1379
GSTM1	12.36093561	4.30225E-24	up	1282	1496	1319	0	0	0
GSTM2	-5.211610215	7.85166E-35	down	20	10	31	585	423	629
GSTM3	2.244016517	2.28688E-58	up	7849	8099	7355	1315	921	1285
GSTM4	1.30933076	1.79239E-05	up	1095	1098	1058	234	380	303
GSTM5	-8.91921088	4.2013E-08	down	0	1	0	216	12	160
GSTP1	0.766109906	0.030728861	up	9068	9720	8622	6009	2568	3060
GSTT2	-3.64876882	4.18091E-06	down	3	6	2	32	17	50
GTDC1	-2.129265336	4.57435E-32	down	150	175	135	462	461	500
GTF2E1	1.029494903	8.76868E-09	up	524	515	501	198	173	165
GTF2E2	1.111625304	5.28378E-06	up	1155	1321	1163	428	441	315
GTF2F2	0.882591948	1.12139E-05	up	922	1015	909	430	357	307
GTF2H2C	1.611337536	0.042464282	up	34	22	16	5	3	9
GTF2H5	-1.792781273	4.57666E-13	down	242	283	211	801	488	531
GTF2IRD1	2.894462728	3.68202E-22	up	1168	1334	1247	81	120	155
GTF2IRD2	-2.842025746	4.94036E-12	down	17	16	11	95	56	75
GTF2IRD2B	-1.437277156	1.61796E-07	down	116	123	167	249	266	265
GTF3C1	1.238024972	1.32238E-06	up	2820	2844	2627	643	917	911
GTF3C2	1.505374887	1.49738E-17	up	2096	2190	2027	471	540	560
GTF3C4	2.019739028	4.80412E-51	up	2572	2686	2369	430	416	489
GTF3C5	1.38419367	2.2356E-30	up	1835	2177	2007	598	469	575
GTF3C6	-0.734034524	0.016244092	down	395	510	380	514	591	387
GTPBP2	0.659094219	4.7335E-08	up	1286	1372	1292	595	558	623

GTPBP3	1.709587331	1.32431E-10	up	694	654	593	174	141	106
GTPBP4	1.856642295	2.5492E-21	up	2437	2793	2339	471	539	459
GTSE1	5.332450972	5.18617E-12	up	1121	1252	1180	42	19	2
GUCA1B	-1.626206864	0.013358944	down	15	9	16	28	16	46
GUCY1A2	-3.996728673	1.29288E-13	down	45	48	46	250	814	454
GUCY1A3	-14.38131584	3.48061E-31	down	0	0	0	3698	2329	2398
GUCY1B3	-12.43125463	6.80983E-24	down	0	0	0	893	603	683
GUF1	-1.44404809	2.04312E-06	down	212	249	198	316	490	447
GUK1	-1.178792796	1.04917E-10	down	1880	2228	1978	4117	2654	3073
GULP1	-2.026047286	0.001463983	down	246	233	190	854	156	1001
GVQW2	-2.494969832	0.020078184	down	3	1	2	7	9	8
GXYLT2	-2.552466681	3.37006E-12	down	43	51	56	156	177	294
GYG1	-1.241416679	0.000353567	down	905	983	776	2263	891	1404
GYG2	-3.203821595	0.008751412	down	112	184	142	34	2272	296
GYLTL1B	3.789697696	0.034532593	up	5	8	8	0	0	1
GYPC	-12.78021451	9.65431E-26	down	0	0	1	1851	1604	1927
GYPE	-8.675451473	8.26402E-11	down	0	0	0	51	38	73
GYS2	-3.248486005	0.043109828	down	8	5	2	0	42	57
GZF1	-0.646916473	4.66702E-05	down	275	279	285	341	279	316
GZMA	-8.951356593	4.83803E-11	down	0	0	0	77	75	40
GZMB	-6.123514976	0.000106533	down	0	1	0	19	25	8
GZMH	-8.214692456	1.37346E-09	down	0	0	0	51	27	40
GZMK	-8.679125665	6.5156E-11	down	0	0	0	69	36	58
H2AFJ	-1.839841427	1.25886E-13	down	586	536	592	1713	977	1746
H2AFX	2.311565376	3.76069E-11	up	1063	1044	928	228	106	106
H2AFY	0.885951323	8.86004E-16	up	3870	4410	3630	1567	1368	1641
H2AFY2	-8.795035078	4.69294E-11	down	0	1	0	134	134	67
H2AFZ	1.807462287	1.1225E-12	up	6916	7362	6537	1977	1009	1293
H3F3B	-0.704882654	0.012920284	down	5442	6105	5178	9262	4593	5753
H6PD	-1.905182934	1.52552E-10	down	521	619	554	1111	1302	2094
HAAO	-9.309710504	1.53955E-14	down	1	1	0	474	113	342
HABP4	-1.561514451	2.64031E-05	down	409	515	468	1344	495	1146
HACD1	-1.235074293	1.21339E-09	down	387	366	371	783	486	631
HACD3	1.065725668	2.84487E-10	up	2812	3051	2635	1018	984	865
HACE1	-1.81367863	2.99979E-19	down	122	119	116	296	249	351
HADH	-1.275498729	0.012376098	down	1027	1127	986	1168	2864	1153
HAGH	-0.956232561	4.83252E-05	down	497	501	473	606	761	639
HAGHL	3.242157901	3.88303E-24	up	489	574	566	54	42	26
HAL	2.734633151	8.15435E-06	up	38	40	43	5	3	5
HAND1	-7.041157064	0.003731768	down	0	0	0	1	43	3
HAND2	-12.26835448	2.46717E-20	down	0	0	0	405	445	1105
HAP1	7.126975031	7.01764E-08	up	62	65	84	0	1	0
HAPLN1	-9.446607526	1.73251E-15	down	0	0	2	559	170	292
HAPLN3	-6.343955678	7.79778E-40	down	10	10	9	861	314	536
HARBI1	1.433103586	0.00325987	up	101	124	93	30	37	15
HAS1	-4.233996628	0.026388369	down	0	1	0	3	7	4
HAS3	1.601888264	0.000506678	up	74	54	85	16	18	16
HAT1	0.649178786	8.20169E-07	up	1569	1753	1405	748	621	774
HAUS2	0.943375033	0.002281107	up	919	929	871	284	419	284
HAUS5	0.982664729	0.000306478	up	424	445	455	213	107	163
HAUS6	2.477334991	1.1685E-102	up	2722	3054	2933	386	337	389
HAUS8	1.938484579	3.11698E-15	up	296	341	352	77	53	54
HAVCR2	-3.907349802	1.91915E-26	down	14	28	32	233	261	290
HBA1	-3.078671257	0.017603562	down	2	7	10	2	43	67
HBA2	-10.09833007	8.54388E-11	down	0	0	0	28	156	240
HBB	-10.19241453	6.45386E-09	down	5	0	1	88	2617	2202
HBD	-6.330040184	0.000540148	down	0	0	2	3	82	23
HBE1	9.776780438	4.13457E-38	up	1573	1771	1584	4	0	0
HBEGF	-1.215224462	0.010302688	down	147	200	139	308	338	137
HBM	-6.453291974	0.027239964	down	0	0	0	0	24	8
HBP1	-0.845856494	0.000586887	down	1475	1512	1440	1570	1610	2491
HCAR1	-7.917763727	0.000789909	down	1	0	0	0	146	23
HCCS	2.42583589	2.67087E-10	up	2031	2148	1835	185	371	217
HCFC1	1.323069256	3.45726E-15	up	2298	2517	2397	614	680	745
HCFC1R1	-2.121066223	0.000166723	down	376	392	356	2032	365	1207
HCFC2	-2.039043985	9.39611E-27	down	201	236	200	653	480	738
HCK	-10.83863971	5.59309E-17	down	0	0	0	341	139	253
HCLS1	-10.68020783	2.54424E-22	down	0	0	3	784	524	2302
HCN2	6.373120536	6.48406E-47	up	631	682	685	5	10	2
HCST	-4.696858866	9.11466E-11	down	6	4	4	155	40	71
HDAC1	1.985230261	4.86314E-29	up	4764	4957	4709	1064	775	758
HDAC10	-0.698804709	0.004220736	down	192	152	187	231	187	198
HDAC11	-1.711474383	2.32278E-19	down	134	151	130	354	254	363
HDAC2	0.91665128	2.05495E-05	up	3020	3191	2733	1471	853	1074
HDAC7	-0.661475706	2.7971E-07	down	1317	1384	1256	1468	1311	1675
HDC	-2.815186883	0.001135817	down	2	2	5	17	16	12
HDDC2	-0.711010504	0.001390805	down	682	722	663	1041	685	690
HDGF	2.354113317	3.34889E-38	up	15166	15917	14482	1819	2083	2401
HDGFRP2	0.824192098	8.99023E-08	up	1370	1497	1384	685	470	560
HDGFRP3	-0.898343263	1.86684E-05	down	702	782	672	1040	685	1158
HDHD2	-2.134234252	5.42378E-26	down	183	236	160	704	515	586
HDX	-9.960593528	7.02922E-13	down	0	0	0	187	50	166
HEATR1	2.240192386	2.69586E-27	up	3313	3454	3275	424	538	536
HEATR3	0.608546755	0.037955088	up	424	521	414	163	238	223
HEATR5A	0.81147532	0.000548946	up	1316	1477	1355	450	587	628
HEATR5B	-0.937910862	0.000721996	down	469	540	421	505	588	847
HEBP1	-0.691467425	0.007193365	down	617	701	615	927	486	836
HEBP2	-3.508348725	1.07136E-15	down	177	222	202	1122	2408	1160
HECA	-2.413598723	4.23319E-22	down	323	392	284	1350	869	1596
HECTD1	-0.60313614	0.011701208	down	1941	2151	1911	1721	2236	2467
HECW1	8.606147382	1.5298E-15	up	359	402	335	0	0	2
HECW2	-1.547626365	0.014123669	down	124	161	125	108	458	247
HEG1	-0.890004879	3.00764E-06	down	1718	1950	1746	2143	2527	2391
HELB	2.144204876	3.33008E-07	up	80	92	97	16	17	10
HELLS	4.421415617	8.06939E-39	up	1611	1688	1575	65	64	31
HELQ	-1.421310703	1.73734E-16	down	143	175	167	332	280	311
HELZ2	0.792572255	0.033449983	up	648	703	693	318	336	173
HEMGN	-6.391251484	0.039696671	down	0	0	0	0	27	3
HEMK1	-0.684105989	0.000624135	down	276	342	289	418	270	353
HENMT1	2.506527434	4.41238E-27	up	826	860	721	110	108	81
HEPACAM	-10.4389747	0.000206895	down	0	0	0	5	436	56

HEPH	-13.86184304	1.66918E-24	down	0	0	0	2708	635	2700
HERC2	-0.735468247	0.021126335	down	135	107	84	141	100	148
HERC4	1.65870211	2.11084E-35	up	3558	3872	3570	901	799	770
HERC6	-2.207135071	4.76915E-09	down	59	103	53	317	198	187
HERPUD1	-0.951391705	5.24879E-12	down	1077	1144	899	1537	1257	1496
HES2	9.591854927	3.36413E-19	up	639	792	742	1	1	0
HES7	5.922770182	1.72922E-25	up	247	271	327	6	1	3
HESX1	-1.337867179	0.035914894	down	18	17	8	27	31	18
HEXDC	-2.201075636	2.4818E-16	down	71	80	65	291	165	257
HEXIM2	-1.064109929	0.014069005	down	61	98	71	170	94	78
HEY2	-5.721535046	1.00798E-20	down	21	13	16	1095	207	653
HEYL	-8.916611864	1.73886E-54	down	5	3	1	871	893	1331
HFE	1.004462686	0.001027674	up	430	543	478	155	120	243
HFM1	-7.802131837	1.05308E-08	down	0	0	0	32	24	32
HGF	-8.545261968	4.66162E-30	down	2	3	1	443	723	381
HGS	0.75199502	1.38162E-11	up	1717	1877	1758	842	657	765
HGSNAT	-1.013355116	1.52666E-07	down	861	1040	874	1172	1368	1404
HHIP	-8.138746785	5.05564E-08	down	0	0	0	20	55	32
HHIPL1	-5.753330503	4.89531E-21	down	0	5	4	125	110	108
HHLA2	4.357710457	0.008547524	up	13	10	8	0	1	0
HIBCH	-1.617262399	9.22491E-08	down	369	339	309	610	905	666
HIC1	-2.420189157	4.52613E-12	down	88	49	73	231	259	313
HID1	-5.494539254	1.20486E-33	down	6	4	12	213	217	278
HIF1A	0.875339219	0.009728748	up	4627	4907	4543	1711	2330	1301
HIF3A	-7.901093726	1.66685E-39	down	5	3	7	792	448	1376
HIGD1A	-0.825720975	1.88314E-10	down	2223	2569	2161	2819	2746	3162
HIGD1B	-7.0604977	0.00026067	down	0	0	0	5	36	8
HINT2	-1.78504308	8.4883E-06	down	124	156	167	389	460	226
HIPK1	-0.606836867	0.025566835	down	1134	1193	1150	1046	1019	1717
HIPK2	-2.144581411	0.001549319	down	524	576	481	517	2941	1247
HIPK3	-2.705936568	8.74239E-19	down	609	755	691	2271	2803	4439
HIRA	0.850954955	1.7141E-05	up	973	989	937	333	391	412
HIST1H1D	4.666169705	0.024186145	up	6	10	4	0	0	0
HIST1H2AE	3.18544939	0.001085935	up	52	68	83	1	12	2
HIST1H2AG	3.265509494	1.8808E-07	up	72	91	66	3	3	11
HIST1H2AI	4.384486591	4.32483E-05	up	30	30	28	2	1	0
HIST1H2AM	5.295914405	0.000399433	up	15	27	18	0	0	1
HIST1H2BF	3.951518401	0.006704012	up	13	18	14	0	2	0
HIST1H2BG	2.882431357	0.005779584	up	58	43	74	1	13	2
HIST1H2BH	7.047093453	8.44315E-08	up	68	78	55	0	1	0
HIST1H2BJ	2.396401439	0.003188305	up	75	90	84	7	22	3
HIST1H2BK	0.936799729	1.50834E-07	up	1117	1169	1161	383	419	474
HIST1H2BL	3.372746919	0.017200143	up	15	15	13	0	0	3
HIST1H2BN	2.878106125	6.7125E-06	up	70	77	57	13	3	4
HIST1H2BO	4.391459853	0.002006189	up	9	29	21	1	0	1
HIST1H3B	7.217514661	2.31914E-07	up	44	31	40	0	0	0
HIST1H3D	5.696473128	0.000687281	up	13	10	17	0	0	0
HIST1H3E	-2.200777023	0.021143052	down	3	3	5	6	11	19
HIST1H3G	6.379696002	3.17646E-06	up	50	42	34	1	0	0
HIST1H3H	2.161882627	0.009232874	up	28	16	31	2	4	6
HIST1H4I	4.695319654	0.000673191	up	26	17	31	0	2	0
HIST1H4K	5.420684264	0.003964025	up	6	20	8	0	0	0
HIST2H2BE	-1.670272718	0.048457605	down	224	223	207	167	1032	180
HIST2H2BF	2.353721453	0.033962891	up	12	17	15	0	3	3
HIVEP1	0.673469416	0.003392772	up	845	926	965	358	349	516
HIVEP2	-1.43700962	8.2928E-13	down	435	483	419	910	635	1049
HJURP	5.175079327	2.09325E-07	up	1007	1189	1054	56	9	1
HKDC1	8.658134444	3.95655E-65	up	3613	3934	3672	15	3	2
HLA-B	-2.451202108	1.09835E-15	down	413	480	407	1145	1736	2120
HLA-C	-0.663601565	0.00063825	down	1454	1599	1459	1995	1639	1433
HLA-DMA	-8.231678512	0.000998517	down	0	0	0	59	56	0
HLA-DRA	-13.9825128	2.89169E-28	down	0	0	1	6117	2453	4051
HLA-DRB1	-10.47197813	9.50427E-05	down	0	0	2	150	4	2007
HLA-DRB3	-6.51031297	0.037986462	down	0	0	0	0	2	35
HLA-DRB4	-6.015358795	0.009203294	down	0	0	0	20	5	1
HLF	-9.352021214	5.03054E-31	down	1	1	2	754	397	737
HLTF	2.209431581	5.66449E-77	up	5884	6696	6115	1072	884	915
HM13	0.860342013	9.1166E-10	up	2439	2641	2445	1133	902	912
HMBS	2.167569817	4.20142E-12	up	681	781	716	97	147	94
HMCES	1.352151893	9.6524E-07	up	1790	1842	1653	411	593	443
HMCN1	-7.394180842	1.59079E-46	down	28	32	26	5142	1201	4266
HMCN2	-2.352575719	0.000205823	down	52	65	51	65	268	259
HMG20B	2.06170708	8.62207E-09	up	5096	5754	5492	1444	516	884
HMGA1	5.079082644	1.2915E-122	up	9200	9606	9144	195	222	165
HMGA2	5.37702921	8.56906E-30	up	338	305	299	4	9	3
HMGB3	3.14889667	1.25162E-09	up	1845	1986	1620	148	216	59
HMGCL	-1.222331402	0.001593083	down	287	394	373	443	782	476
HMGCLL1	-7.972480622	7.02435E-07	down	0	0	0	15	57	22
HMGCR	2.574693486	2.13743E-34	up	3054	3054	2647	329	386	319
HMGCS1	2.434871488	6.17319E-05	up	8501	9467	7635	627	2006	555
HMGN1	0.859852309	0.000293189	up	4043	4523	3805	2017	1552	1262
HMGN3	-0.65976457	0.001761155	down	1314	1315	1336	1903	1268	1309
HMGXB3	1.193682338	3.37201E-14	up	1809	1722	1631	528	461	621
HMHA1	0.825214527	0.026884041	up	624	606	615	344	130	284
HMMR	5.70485914	4.85531E-15	up	1714	1888	1664	53	16	4
HMSD	-5.309036478	0.011907892	down	0	0	0	8	2	6
HMX3	7.917312081	2.68342E-09	up	69	53	65	0	0	0
HN1	3.96749532	2.63587E-24	up	4817	5058	4391	260	264	114
HN1L	2.356376263	2.35894E-89	up	4589	4983	4514	657	634	658
HNF1B	8.25416203	1.82369E-10	up	81	77	79	0	0	0
HNF4A	9.196854869	1.44362E-12	up	104	179	175	0	0	0
HNF4G	4.597208404	4.02951E-12	up	122	150	98	4	0	7
HNMT	-1.799073773	1.40091E-47	down	532	586	507	1519	1157	1352
HNRNPA1	0.693604448	2.04537E-11	up	8821	9692	8325	4274	3623	3883
HNRNPA2B1	1.612865156	3.67552E-38	up	39235	41264	36863	8719	8447	10065
HNRNPAB	1.788068054	4.73471E-39	up	5821	6399	5543	1304	1197	1139
HNRNPC	0.78380967	0.000348355	up	12436	14278	12367	7117	4057	5103
HNRNPD	1.075175479	2.23963E-26	up	7066	7679	6828	2622	2048	2631
HNRNPF	1.804408905	1.50188E-41	up	8933	9807	8875	2075	1799	1731
HNRNPK	0.818307332	4.1893E-10	up	17090	18062	16107	6907	5850	7966
HNRNPL	1.113583263	3.95786E-11	up	453	520	466	154	155	162

HNRNPM	1.356334676	3.03445E-41	up	6170	6549	5908	1735	1577	1857
HNRNPR	1.571800542	2.04452E-06	up	91	115	105	26	26	22
HNRNPU	1.305015447	2.294E-19	up	18862	20566	17924	6288	4289	6036
HNRNPUL1	0.749167976	9.24533E-05	up	5979	6376	5792	2432	2062	3219
HNRNPUL2	-1.279007196	1.26781E-06	down	547	616	507	1265	628	1027
HOGA1	-1.070457922	3.10013E-05	down	75	74	79	107	102	132
HOMER1	1.753939801	0.004794934	up	442	480	443	37	161	77
HOMER2	-1.819467996	0.004199196	down	33	42	50	64	173	64
HOMER3	0.75304845	0.012527363	up	977	1035	925	601	315	335
HOOK2	-1.5164602	0.000112715	down	243	215	265	525	618	307
HOOK3	-0.884523006	4.99297E-07	down	848	880	867	1183	887	1362
HOPX	-8.56858595	2.37056E-07	down	0	0	0	13	91	37
HORMAD1	6.425543273	4.41779E-29	up	311	328	328	1	3	4
HOXA1	2.222444718	0.000501735	up	55	87	52	16	3	11
HOXA10	7.420468497	5.43165E-36	up	551	613	521	3	1	3
HOXA11	11.20676411	7.66292E-20	up	568	686	588	0	0	0
HOXA13	10.56727955	1.47091E-17	up	398	375	404	0	0	0
HOXA2	-7.184931688	1.94089E-05	down	0	3	0	144	3	168
HOXA3	-3.755642579	0.000645135	down	21	16	17	313	6	229
HOXA4	-2.492890771	0.005892423	down	17	22	21	169	12	70
HOXA6	5.800471199	0.000613243	up	11	11	21	0	0	0
HOXA7	5.32830275	1.01889E-11	up	105	92	87	0	3	2
HOXB13	9.863351143	2.48621E-15	up	249	259	217	0	0	0
HOXB2	-5.840980068	1.76465E-12	down	12	2	3	410	86	230
HOXB3	-2.481876537	0.000123001	down	101	97	106	668	97	494
HOXB4	-5.235877367	2.90663E-09	down	4	3	7	247	29	118
HOXB6	2.851255914	1.14034E-05	up	326	315	316	49	6	43
HOXB7	2.156896771	0.022719801	up	160	142	157	55	2	20
HOXB8	4.467340951	1.90106E-07	up	57	44	52	3	0	2
HOXB9	6.345101124	2.67658E-06	up	41	41	41	0	1	0
HOXC10	9.50424892	1.19929E-27	up	1016	1073	970	2	0	1
HOXC11	10.63858985	1.16364E-17	up	428	375	432	0	0	0
HOXC13	11.15259176	1.31547E-19	up	600	646	527	0	0	0
HOXC6	3.1720262	2.36237E-05	up	162	181	161	11	2	28
HOXC8	4.815607086	2.82057E-07	up	56	43	57	1	0	3
HOXC9	5.715144382	7.86611E-17	up	135	165	143	1	1	4
HOXD1	-6.662344335	3.32902E-05	down	0	0	0	10	10	20
HOXD3	-7.18118222	1.98909E-05	down	0	0	1	27	7	81
HOXD4	-6.85104627	1.03891E-12	down	3	0	2	127	55	244
HOXD8	-3.87515233	3.5029E-11	down	10	5	5	80	41	92
HP	-4.214428576	1.67943E-07	down	47	30	32	248	968	140
HPCAL1	1.790664513	2.04847E-23	up	1760	1845	1777	349	392	357
HPDL	6.615252813	2.7727E-21	up	663	810	691	2	12	1
HPF1	-1.641690814	1.18026E-09	down	173	151	143	463	261	324
HPGD	1.025017907	0.025076628	up	120	121	118	46	20	62
HPGDS	-8.91730629	2.70924E-10	down	0	0	1	211	44	127
HPR	-9.55955539	7.7916E-10	down	0	0	0	70	180	31
HPRT1	2.392086763	1.97143E-12	up	1981	2326	1890	436	208	203
HPS1	-1.611331658	2.60262E-15	down	350	305	265	759	530	725
HPS3	2.082258579	7.72251E-35	up	2552	2756	2455	429	349	531
HPS5	0.974553501	2.9346E-06	up	1643	1905	1661	620	476	800
HPS6	0.715798439	1.28097E-05	up	385	442	378	181	149	192
HPSE2	-7.555383883	7.03412E-14	down	3	2	9	410	98	1465
HR	4.302703691	3.50659E-34	up	1792	1942	1905	108	53	44
HRASLS	-3.899692525	0.04115482	down	1	1	1	1	27	1
HRASLS2	4.571422942	5.37966E-09	up	68	87	47	3	2	1
HRASLS5	-9.159349763	3.60475E-06	down	2	0	2	25	1384	101
HRCT1	-1.475400024	0.000862316	down	95	137	99	294	102	271
HRH2	-9.839043458	4.83066E-13	down	0	0	0	96	172	81
HRK	2.003139414	0.021807148	up	24	23	16	1	5	5
HS2ST1	1.233719608	5.86384E-08	up	1803	1946	1805	474	625	560
HS3ST1	-8.85675079	1.37235E-10	down	0	0	0	68	75	36
HS3ST2	-10.7676834	2.46043E-14	down	0	0	0	129	382	143
HS6ST2	9.610294397	5.23613E-45	up	1852	1965	1675	4	1	0
HSBP1L1	0.907191248	0.003189609	up	234	268	223	103	102	67
HSD11B1	-6.994374579	1.27765E-12	down	3	2	0	66	110	279
HSD11B2	2.998693799	4.94635E-07	up	95	111	125	12	14	3
HSD17B1	-2.276299089	0.003462067	down	13	9	11	32	62	16
HSD17B10	1.478820224	8.50707E-07	up	3388	3548	3298	825	1080	658
HSD17B11	-2.922293254	1.2372E-36	down	225	259	175	1263	881	1426
HSD17B12	-1.970277665	7.65609E-08	down	895	1014	919	1646	3369	2676
HSD17B14	-2.895765278	3.44459E-13	down	39	33	30	240	102	209
HSD17B3	-2.383645231	0.011077554	down	3	2	11	30	14	17
HSD17B7	2.349337381	1.6554E-25	up	689	778	625	121	68	105
HSD3B7	-0.969679964	0.020995749	down	189	153	160	187	318	179
HSDL1	0.694654707	0.001360018	up	737	831	732	294	363	343
HSF2BP	4.808068438	6.2666E-14	up	103	123	92	1	4	3
HSF4	-0.970340504	0.004065428	down	280	248	265	482	214	433
HSH2D	1.766132072	3.49964E-05	up	68	63	55	16	11	12
HSP90AA1	1.633846711	1.95513E-12	up	59281	61373	52488	17330	11777	10639
HSP90AB1	1.320010239	2.32657E-32	up	40850	44129	39990	12217	11516	11718
HSP90B1	1.073438759	2.82186E-13	up	23576	27186	23440	9143	8184	7612
HSPA12B	-7.667890527	5.10771E-24	down	4	0	2	195	343	321
HSPA13	1.075349182	3.66446E-10	up	1721	1958	1632	715	458	630
HSPA14	1.020102729	3.5171E-11	up	1083	1217	1060	435	379	360
HSPA2	-1.481066879	3.76011E-19	down	266	311	245	524	515	585
HSPA4	1.900693682	1.4329E-69	up	6865	7426	6450	1331	1247	1360
HSPA4L	1.808323845	9.46552E-05	up	1205	1251	1207	268	102	394
HSPA5	1.985245101	5.20284E-12	up	28923	30370	27001	5801	5896	3604
HSPA6	-3.016037138	2.27477E-06	down	31	20	18	256	68	85
HSPA8	1.002249195	2.16327E-06	up	32826	33225	27669	13779	10187	9300
HSPA9	1.505835577	5.96114E-05	up	13433	14731	13435	2673	4807	2641
HSPB1	-1.948924477	3.44565E-05	down	4598	4878	4729	21410	5202	13632
HSPB11	0.646382232	0.006968978	up	796	853	734	482	277	332
HSPB2	-8.368065352	2.67571E-28	down	2	1	2	572	270	351
HSPB6	-10.88530715	5.7969E-48	down	5	1	0	1871	2273	3963
HSPB7	-2.442789524	7.03276E-11	down	1893	2202	2034	4983	10527	7550
HSPB8	-1.942181626	1.23138E-22	down	838	890	805	2959	1959	2033
HSPD1	2.275731056	1.73675E-09	up	16689	18390	15501	1865	3462	1877
HSPG2	-2.243735521	4.85733E-22	down	3463	4216	3899	10272	12830	15606
HSPH1	1.804731445	4.30227E-05	up	5499	5459	5178	1918	761	660

HTATIP2	2.350235113	7.1013E-65	up	2581	2918	2730	400	378	365
HTR1B	-9.219181993	1.24042E-09	down	0	0	0	122	19	102
HTR1D	7.720082546	1.78011E-12	up	199	219	178	0	2	0
HTR1F	-5.891584881	0.00880301	down	0	0	0	3	2	19
HTR2A	-9.958224899	5.80734E-15	down	0	0	0	148	90	157
HTR2B	-9.792841894	1.13656E-11	down	0	0	0	88	190	56
HTR2C	4.739051209	0.024392823	up	8	10	3	0	0	0
HTR4	-5.585549838	1.95015E-07	down	3	0	0	32	31	40
HTR7	-7.636269448	3.54065E-08	down	1	0	0	78	43	32
HTRA1	-4.100744039	9.88307E-34	down	414	486	392	7862	2962	5237
HTRA3	-3.179925547	1.11078E-25	down	156	192	190	1098	770	1640
HTRA4	-6.420688989	0.000795118	down	0	0	0	4	20	8
HUNK	1.492889641	0.027452274	up	62	45	39	6	19	11
HUWE1	1.720181931	2.10524E-11	up	11776	13248	12561	2112	2388	3607
HVCN1	-8.862218941	3.06478E-15	down	1	0	1	244	138	291
HYAL1	3.696906114	5.05588E-11	up	2659	2836	2710	73	259	97
HYAL3	2.69478185	4.35666E-17	up	341	415	400	35	54	36
HYI	-0.840244161	3.88426E-05	down	456	560	481	687	654	549
HYKK	-2.25472775	1.38269E-08	down	26	41	29	127	66	136
HYLS1	-2.017525537	0.000136931	down	18	40	25	123	47	71
HYOU1	3.019207972	2.9933E-13	up	146	174	166	12	20	10
IAH1	-0.926119293	8.1991E-08	down	496	552	484	701	709	645
IARS	1.617939713	4.46512E-19	up	5391	5865	5156	1126	1324	1318
IARS2	1.305646434	0.000147581	up	6828	7413	6416	1437	2591	1767
ICA1	-10.7903311	6.73442E-16	down	0	0	0	169	334	171
ICA1L	-5.277908609	3.96258E-18	down	16	9	7	284	131	493
ICAM2	-8.32113498	1.24414E-26	down	1	3	1	302	484	322
ICAM3	-2.067712735	0.015404494	down	108	113	102	157	661	81
ICAM5	3.102161098	5.6517E-29	up	287	302	293	20	23	30
ICE1	1.868369998	1.31343E-24	up	4545	4939	4264	833	740	1111
ICK	-2.35889982	1.91719E-18	down	170	232	182	789	470	891
ICOS	-6.035716997	0.003053966	down	0	0	0	2	12	11
ID1	-2.100023087	1.81133E-07	down	548	626	622	2975	1444	1110
ID2	-5.200749402	2.88097E-33	down	50	52	78	2548	1207	1028
ID3	-2.64270423	5.10938E-05	down	12	12	8	83	32	29
ID4	-14.77612827	4.83353E-30	down	0	0	0	6091	2000	3183
IDE	0.586782948	0.000132395	up	1448	1508	1357	638	656	741
IDH3B	-0.917568744	0.000403829	down	796	804	805	1041	1251	897
IDI1	1.268085914	0.000105442	up	2772	3048	2628	772	1061	607
IDNK	-1.773266817	4.19959E-08	down	47	45	31	122	82	96
IDS	-2.689081249	2.67556E-12	down	882	975	898	4908	2002	6026
IDUA	-3.578564777	2.10793E-21	down	23	18	33	205	161	271
IER5	1.10145216	0.014606531	up	1136	1286	1105	681	216	297
IFFO1	-8.127483732	1.24607E-33	down	1	8	1	881	377	740
IFI16	-2.622848101	1.14412E-24	down	489	557	461	1672	2338	2511
IFI27	-3.227880166	0.023900676	down	0	1	3	4	13	9
IFI30	1.693379006	1.77353E-13	up	3387	3818	3453	997	730	614
IFI44	-2.76433961	4.0159E-27	down	201	222	140	1050	884	767
IFI44L	-11.22516937	1.38461E-18	down	1	0	0	838	578	414
IFI6	-2.271224266	1.17925E-08	down	212	251	181	1200	433	615
IFIH1	-3.395183277	3.55156E-29	down	46	56	44	499	263	340
IFIT1	-1.124713489	0.040437409	down	529	544	453	1525	426	473
IFIT3	-1.441277014	0.000402211	down	294	346	251	931	421	384
IFIT5	-0.623870329	0.022197475	down	404	537	409	624	330	539
IFITM1	-6.590123044	7.0043E-144	down	31	37	38	2159	2570	2464
IFITM10	-2.945554091	5.22681E-07	down	18	10	15	93	36	114
IFITM2	-1.538161169	5.08818E-11	down	1093	1157	1230	3222	1912	2119
IFITM3	-2.312476305	1.39273E-30	down	3023	3312	3274	13307	7988	13060
IFNAR2	0.680595272	7.37268E-05	up	918	1009	926	410	437	410
IFNE	5.588823785	0.002387563	up	8	8	21	0	0	0
IFNGR1	-2.710314304	2.94038E-84	down	494	581	482	2347	2165	2720
IFNGR2	-1.103494827	0.00015979	down	83	120	120	144	151	197
IFNLR1	3.304294526	5.89726E-08	up	167	128	129	20	7	4
IFRD1	0.896169187	0.014457741	up	3205	3453	3117	1941	721	1149
IFRD2	1.788826583	5.24453E-10	up	1574	1737	1744	324	425	274
IFT140	0.772394136	0.001753806	up	577	651	614	234	198	339
IFT172	-1.564854276	3.75908E-21	down	212	230	185	459	372	490
IFT27	-0.907148098	0.008493681	down	128	108	119	226	124	129
IFT46	-1.538729394	8.01379E-12	down	178	151	167	387	261	388
IFT52	-0.958587175	2.00219E-06	down	333	364	304	537	341	516
IFT57	-1.298284043	3.31831E-21	down	367	362	376	683	575	679
IFT74	-5.292915061	4.09537E-44	down	8	10	15	389	255	282
IFT81	1.148861452	2.02134E-05	up	851	1054	829	311	192	382
IFT88	-0.771295281	0.000154629	down	189	216	181	273	182	260
IGDCC4	-4.138830014	1.74214E-07	down	5	9	3	28	101	75
IGF1	-5.585739893	1.46018E-14	down	54	61	58	561	3791	1197
IGF1R	0.903438873	0.013465291	up	3109	3375	3396	1318	681	1834
IGF2	-3.432802879	0.019445933	down	1	1	1	3	14	5
IGF2BP1	8.530933557	5.99413E-58	up	1521	1557	1615	3	6	0
IGF2BP2	2.412974297	1.41215E-06	up	715	736	696	39	105	139
IGF2BP3	7.197107299	2.3024E-154	up	2630	3037	2795	13	12	16
IGF2R	1.282127523	2.72848E-05	up	7221	7927	7174	1477	2216	2776
IGFBP1	10.0486969	8.47008E-16	up	265	326	236	0	0	0
IGFBP2	-13.09360524	1.92187E-27	down	1	1	0	4216	1423	7143
IGFBP4	-2.006635324	2.37621E-05	down	3155	3594	3293	6801	14890	5897
IGFBP5	-18.03055453	2.69451E-40	down	0	0	0	32282	12479	63464
IGFBP6	-4.652307345	4.0579E-09	down	453	519	495	10536	972	15886
IGFBP7	-4.514984676	6.51773E-32	down	2506	2909	2486	69312	22190	39897
IGFBPL1	-4.091994918	2.44973E-05	down	14	7	7	187	12	159
IGFLR1	-2.382968789	3.99454E-05	down	24	30	18	159	53	59
IGFN1	3.275350908	7.22172E-12	up	130	188	172	7	9	20
IGIP	-3.808003499	8.20744E-27	down	40	67	46	695	313	532
IGK	-4.312933806	0.002882607	down	0	1	1	12	10	6
IGL	-0.989455869	0.000129506	down	228	248	258	398	234	418
IGSF1	-3.872618053	2.37966E-05	down	0	8	7	46	68	37
IGSF10	-8.464700339	1.02159E-39	down	3	5	3	511	827	1408
IGSF11	-2.168082088	0.0282252	down	1	5	8	9	18	17
IGSF21	-8.755470775	2.09755E-09	down	0	0	0	92	21	62
IGSF22	-5.923768407	0.000658942	down	0	0	0	8	6	10
IGSF3	3.932789475	1.68595E-15	up	3768	3864	3733	143	279	88
IGSF6	-9.746929341	5.53607E-14	down	0	0	0	158	89	93

IGSF9B	-2.534090524	1.88541E-05	down	39	37	30	147	53	249
IKBIP	2.038493671	1.51595E-16	up	2067	2347	2208	345	449	335
IKBKAP	0.658205447	4.77608E-08	up	1682	1868	1643	770	729	835
IKBKB	-0.669692442	0.02730415	down	795	832	827	1003	573	1246
IKBKE	1.793644104	2.13155E-13	up	267	320	333	76	56	57
IKBKG	1.012959443	0.00013079	up	198	174	217	79	59	71
IKZF1	-8.372255227	7.312E-12	down	0	1	1	128	77	275
IKZF2	-5.555604351	4.75057E-15	down	5	1	5	82	98	193
IKZF3	-7.31692484	4.80296E-10	down	2	0	0	89	46	96
IKZF4	-0.911823378	0.000381829	down	108	154	108	171	131	193
IKZF5	-1.072368749	1.68486E-08	down	271	310	275	464	325	499
IL10	-7.853325574	3.72414E-08	down	0	0	0	27	39	23
IL10RA	-11.7479353	2.01497E-20	down	0	0	0	475	286	609
IL11	9.514280405	2.97635E-14	up	191	189	188	0	0	0
IL11RA	-5.597654646	4.53483E-34	down	25	34	37	1689	504	1202
IL12A	2.775089656	0.003923323	up	28	30	18	6	1	1
IL12RB1	-7.63317253	7.06352E-08	down	0	0	0	34	17	28
IL12RB2	-6.131830584	0.001361296	down	1	0	0	2	12	40
IL13RA1	-0.76778251	0.000267149	down	1654	1952	1647	2373	1504	2540
IL13RA2	-11.43042768	1.49867E-05	down	0	0	0	740	6	397
IL15	-1.035384155	0.027126687	down	100	137	120	293	104	132
IL16	-9.9552194	6.58363E-19	down	0	0	2	488	293	655
IL17B	-6.191660619	8.57775E-06	down	2	0	0	59	13	36
IL17D	-6.125987445	8.83307E-12	down	4	3	0	156	42	159
IL17RA	-0.622903325	0.003710812	down	524	542	546	630	433	720
IL17RB	2.5391072	4.36094E-05	up	128	135	144	34	8	9
IL17RD	-3.897970431	3.48672E-60	down	26	34	28	338	279	315
IL17RE	-1.275670658	0.003120905	down	70	79	71	167	62	158
IL17REL	4.840068406	0.000175562	up	25	28	29	0	2	0
IL18	3.276168709	4.7532E-09	up	3118	3475	2774	321	62	328
IL18R1	-8.143853089	1.9788E-13	down	2	0	0	166	109	130
IL18RAP	-7.316365488	7.91501E-07	down	0	0	0	14	22	26
IL1B	-7.19382769	1.27586E-05	down	0	1	0	74	31	8
IL1R1	-6.758778303	1.10579E-83	down	37	44	47	2310	3971	3396
IL1R2	-3.519720674	0.028000752	down	0	0	5	9	10	23
IL1RAP	1.677074026	2.0935E-11	up	524	678	603	118	152	126
IL1RL1	-6.186600085	4.02996E-05	down	1	1	0	11	67	19
IL1RL2	-6.141565502	0.001431114	down	0	0	1	4	40	6
IL1RN	-3.915533044	0.002216399	down	6	4	1	100	16	6
IL2	4.374771622	0.047850849	up	4	4	8	0	0	0
IL20	4.684296804	0.018034969	up	7	7	6	0	0	0
IL20RA	-6.201558632	1.32529E-05	down	1	0	0	19	19	18
IL20RB	2.115906071	0.008595983	up	306	339	360	39	10	122
IL22RA1	3.879509516	1.78589E-12	up	92	103	74	4	3	6
IL24	6.144614087	7.37901E-05	up	14	19	22	0	0	0
IL27RA	0.746739195	0.000365415	up	295	339	304	121	136	138
IL2RA	-8.348743361	6.03223E-08	down	0	0	0	30	18	83
IL31RA	-2.763643307	0.023809825	down	20	20	23	119	2	199
IL32	2.130242405	1.49329E-05	up	1957	2196	2169	394	463	146
IL33	-9.091194448	1.20291E-74	down	6	3	17	3992	3168	3027
IL34	-5.957154545	6.8548E-16	down	2	5	8	410	98	175
IL6ST	-1.877013932	8.4539E-11	down	3959	4253	3853	7951	8759	14864
IL7	-6.84263445	0.001470145	down	0	0	0	1	25	17
IL7R	1.912960641	0.002620444	up	630	689	577	37	102	218
ILDR2	-6.080875245	9.2442E-05	down	0	1	1	18	64	8
ILF2	1.801597685	1.16778E-38	up	6710	7250	6624	1614	1295	1287
ILF3	0.982238256	8.53057E-15	up	6599	7096	6350	2377	2088	2759
ILK	-1.385343128	0.00091277	down	2146	2091	2035	5396	1725	4866
ILKAP	-0.885323187	0.001614247	down	301	312	299	558	285	370
IMMP1L	0.885423909	0.001331639	up	200	269	191	84	91	76
IMPA2	2.727075844	7.43013E-20	up	1358	1531	1391	105	176	172
IMPACT	-1.223146523	1.61552E-12	down	418	442	329	663	593	714
IMPDH1	2.012253665	2.67079E-07	up	2851	3225	2803	299	669	559
IMPDH2	1.002346092	7.62878E-08	up	4409	5008	4355	1756	1688	1405
IMPG2	-3.154818298	0.000348113	down	5	3	1	26	18	13
INA	7.574248462	2.27516E-35	up	637	617	615	0	1	6
INAFM1	-3.263239798	8.16382E-12	down	28	58	28	365	135	286
INAFM2	-2.008557346	5.02259E-08	down	271	369	334	1335	491	1017
INCA1	-2.223187304	0.011679469	down	7	3	2	11	12	17
INCENP	3.519990659	3.36211E-71	up	2865	3063	2783	203	126	216
INF2	1.557937175	3.72326E-30	up	3925	4411	4457	1176	919	1000
ING1	1.211284713	7.80389E-15	up	619	757	615	226	177	208
ING3	-0.74266848	0.00010097	down	283	293	262	402	265	336
ING4	-1.40301237	1.83068E-10	down	287	355	316	710	426	682
ING5	-1.428556466	7.98094E-17	down	307	306	280	644	448	629
INHBA	-5.092689735	2.65874E-30	down	34	21	19	944	394	495
INHBE	5.944128725	7.71144E-12	up	107	123	122	0	4	0
INIP	0.624613735	0.000854327	up	330	386	316	159	135	182
INMT	-10.02041513	1.97488E-44	down	3	3	3	2449	3051	979
INO80C	1.723377622	2.32367E-17	up	571	670	546	147	126	110
INPP1	-0.597781201	0.007124363	down	371	420	357	530	335	376
INPP4B	-3.500897328	1.86303E-11	down	157	127	111	682	575	1986
INPP5A	-2.596986052	3.04159E-22	down	284	294	273	1450	768	1502
INPP5B	-1.500084879	5.14719E-06	down	274	316	301	693	339	793
INPP5D	-6.428631071	0.001872565	down	0	0	0	2	8	24
INPP5F	-1.871111271	2.46532E-16	down	217	225	202	600	399	693
INPP5J	-1.468439173	0.000576332	down	17	35	33	64	48	55
INPP5K	-1.526240079	1.75753E-24	down	302	332	302	685	617	609
INPPL1	1.923703285	1.0071E-61	up	6024	6557	6252	1157	1121	1243
INSC	-6.531486052	0.000603466	down	0	0	0	3	12	21
INSL4	7.337157456	7.91785E-08	up	34	47	45	0	0	0
INSR	-0.596169972	0.017169395	down	1203	1285	1174	1226	980	1761
INSRR	6.017523247	2.50692E-21	up	209	202	228	1	4	2
INTS1	1.150330688	1.18307E-24	up	2514	2714	2572	837	746	917
INTS10	0.727537526	6.47246E-07	up	1804	1889	1842	798	794	775
INTS12	-0.770803982	4.97834E-05	down	367	378	324	465	335	505
INTS3	1.852389752	0.02351203	up	144	62	62	35	13	6
INTS4	0.898190824	5.11409E-05	up	652	714	627	233	204	326
INTS5	0.677503267	9.90676E-06	up	466	512	471	238	197	209
INTS6L	-8.786182246	2.12759E-25	down	0	4	0	540	261	465
INTS7	2.117414579	4.94413E-17	up	1105	1103	1126	170	214	156

INTS8	1.067041743	3.04587E-16	up	1448	1760	1470	547	501	533
INTU	-1.04116027	0.002921066	down	88	94	123	176	96	182
IP6K3	-5.537814564	0.000961116	down	4	0	0	19	98	8
IPCEF1	-3.705295485	2.21645E-05	down	7	2	6	92	21	32
IPMK	2.108294595	1.00591E-13	up	1277	1348	1199	163	174	295
IPO11	1.614346483	1.91943E-07	up	532	639	580	94	153	154
IPO4	1.635131872	1.90718E-06	up	791	973	884	173	265	154
IPO5	1.185102239	9.35909E-35	up	7113	7734	6914	2306	2026	2473
IPO7	1.647155674	2.37907E-26	up	15135	16243	14819	3457	2860	4215
IPO9	1.112614911	1.34711E-06	up	3379	3676	3501	1233	800	1479
IPPK	1.808443154	3.46635E-11	up	625	612	631	136	144	96
IQCA1	-7.899404108	1.46053E-07	down	0	0	0	55	16	25
IQCB1	1.065404555	1.70197E-08	up	784	802	701	307	195	281
IQCC	1.323039705	0.005286448	up	110	169	103	58	25	26
IQCD	1.772304781	1.98356E-05	up	74	93	84	12	17	23
IQCE	1.259470826	1.94688E-17	up	904	934	916	281	229	311
IQCH	2.197718686	1.84877E-06	up	61	82	90	9	12	15
IQGAP1	1.12160573	0.003892974	up	21507	23332	21725	7566	3691	10968
IQGAP2	-5.451531813	1.15799E-24	down	21	16	6	311	359	670
IQGAP3	5.067216088	1.02918E-11	up	2055	2223	2149	106	24	10
IQSEC1	-1.927533808	5.69759E-25	down	582	654	585	1426	1590	1884
IQSEC3	-8.249644548	6.78761E-06	down	0	0	0	16	86	9
IRAK1	2.637425663	6.56272E-12	up	6886	7403	6937	558	1137	660
IRAK1BP1	-1.545503581	3.9063E-05	down	41	58	32	119	71	82
IRAK3	-9.713287565	1.51681E-39	down	1	0	4	790	920	1278
IRAK4	-1.029764829	3.54381E-06	down	360	391	360	646	377	608
IREB2	0.892950569	8.96034E-05	up	2265	2461	2177	716	830	1087
IRF1	-1.278361249	7.89793E-06	down	228	213	198	478	347	278
IRF2	-2.268186043	6.61272E-27	down	123	152	140	479	373	576
IRF4	1.583533953	0.000453322	up	72	85	76	28	10	18
IRF5	-1.969382005	4.1498E-06	down	73	53	55	237	90	190
IRF6	-6.70336062	1.67465E-19	down	3	0	3	127	115	208
IRF8	-10.95158815	4.71387E-15	down	0	1	0	308	869	266
IRF9	-2.982886603	2.41806E-24	down	94	142	103	868	459	590
IRS2	0.764659191	0.019741942	up	3304	3325	3315	1045	1102	2039
IRX1	-8.013743035	0.000125276	down	0	0	0	77	1	29
IRX2	-6.848986798	2.16062E-05	down	0	0	0	21	16	8
IRX6	-7.884042855	0.005030543	down	0	0	0	0	78	6
ISCA1	-0.990658032	5.97356E-09	down	710	740	678	952	1034	1000
ISCA2	1.075003303	0.002314297	up	487	522	499	137	223	138
ISCU	-2.138748952	1.89189E-49	down	916	1015	907	3469	2353	3118
ISG15	1.63860379	0.006422771	up	1992	1989	1726	900	217	223
ISG20L2	1.389484611	6.58986E-17	up	1150	1212	1105	383	254	309
ISL1	4.381217105	0.000211663	up	66	80	55	0	0	7
ISL2	6.524705183	1.63181E-08	up	83	106	70	2	0	0
ISLR	-11.51602322	5.28337E-24	down	0	0	2	1866	688	1723
ISLR2	-6.679888901	0.000581216	down	0	0	0	8	26	4
ISM1	-7.372201428	4.12532E-08	down	1	0	6	147	30	701
ISM2	2.829297014	0.031964013	up	5	22	22	3	0	2
ISY1	0.874795759	0.001464443	up	366	363	293	176	110	112
ISYNA1	-4.432133218	5.98686E-33	down	39	65	58	850	496	1172
ITFG1	-1.127638761	1.89262E-12	down	953	986	905	1553	1163	1731
ITFG2	-2.222613881	1.14768E-22	down	111	89	80	343	277	312
ITGA10	-9.982276745	3.32458E-18	down	1	7	0	3151	215	2471
ITGA11	-13.69267168	1.56075E-21	down	1	0	0	3144	736	6621
ITGA2	3.881166064	1.04038E-31	up	3641	3925	3761	167	240	128
ITGA2B	1.953437017	0.001410973	up	76	100	92	18	24	6
ITGA4	-10.7197655	4.17613E-16	down	0	0	0	356	158	157
ITGA5	1.577361536	2.65068E-20	up	8039	8796	8614	2308	1512	2301
ITGA7	-8.023805687	4.2886E-267	down	26	27	26	4809	4233	5602
ITGA8	-17.27333356	1.68111E-15	down	0	0	0	26956	1315	36902
ITGA9	-12.40959672	4.81476E-22	down	0	0	0	712	409	1053
ITGAD	4.097820379	5.78333E-12	up	117	93	100	8	3	2
ITGAL	-6.327598164	1.36584E-18	down	0	3	3	147	85	113
ITGAM	-7.187845778	3.54179E-24	down	1	5	3	374	142	434
ITGAX	-3.967697845	6.71447E-16	down	15	22	12	290	128	131
ITGB1BP2	-5.853608682	2.88092E-20	down	8	2	3	193	215	122
ITGB2	-1.621355381	0.000372479	down	348	340	393	1063	328	1049
ITGB3	-2.420656809	2.96808E-08	down	204	204	200	974	329	1078
ITGB3BP	1.310860424	0.00452623	up	612	734	567	322	107	129
ITGB4	1.316915574	1.57282E-06	up	2075	2189	2192	455	590	791
ITGB6	5.944556156	0.000829937	up	8	30	11	0	0	0
ITGB7	-6.674048911	1.55354E-07	down	2	0	0	34	68	40
ITGB8	1.248907738	1.31312E-07	up	551	567	537	192	170	131
ITGBL1	-8.6501128	7.95124E-55	down	13	14	14	5715	1337	5057
ITIH3	-5.241331466	2.23053E-10	down	2	7	7	281	101	53
ITIH4	-6.728369459	2.91365E-23	down	5	8	6	883	171	422
ITIH5	-13.01765535	4.13804E-25	down	0	0	0	918	1308	973
ITK	1.729317244	0.001416656	up	193	197	176	20	30	72
ITLN1	-7.797460914	2.4241E-07	down	0	0	2	197	13	125
ITLN2	-6.000022077	0.003256974	down	0	0	0	14	9	2
ITM2A	-13.18998518	6.17884E-26	down	0	0	0	931	1174	1547
ITM2B	-3.108309596	3.8248E-206	down	2116	2374	2121	14788	11349	14524
ITM2C	-2.20600217	0.000316391	down	884	802	818	5038	740	2785
ITPKA	4.089128581	6.79891E-08	up	88	88	93	4	7	0
ITPKB	-1.589070679	1.37016E-11	down	299	292	305	553	552	818
ITPKC	-1.08204234	0.006277177	down	238	257	253	234	477	391
ITPR2	-1.087060785	0.025488789	down	733	831	709	700	1748	855
ITPR3	3.199156849	7.24166E-19	up	6830	7491	7160	321	625	693
ITPRIP	1.922196559	5.9407E-70	up	2449	2829	2488	520	444	491
ITPRIPL1	-8.414007215	5.96709E-10	down	0	0	0	34	52	46
IVD	-0.938705099	0.01344496	down	839	898	854	925	1606	908
IVNS1ABP	0.955039958	0.008326174	up	4142	4753	4102	1224	2131	1284
IWS1	1.824321232	4.43664E-73	up	3055	3248	3056	665	575	639
IZUMO4	-3.530447934	3.3703E-15	down	12	12	8	88	62	115
JADE1	-0.836363138	0.000409227	down	851	939	834	911	1004	1410
JADE3	2.979776724	4.03157E-59	up	1531	1529	1520	126	144	141
JAG1	-3.333088672	1.21516E-49	down	540	530	527	4782	2624	4206
JAG2	1.224889975	0.001470877	up	1362	1525	1440	381	601	300
JAGN1	0.596735235	0.009279054	up	796	852	738	411	395	307
JAK1	-0.641522235	0.001855837	down	3252	3358	3330	3137	3445	4425

JAK2	-2.851215134	1.6593E-09	down	269	312	228	1430	569	2250
JAKMIP2	-7.106607668	4.71817E-05	down	0	0	0	5	22	26
JAKMIP3	1.509317587	0.004830153	up	81	71	71	9	23	23
JAM2	-11.90695266	2.80209E-22	down	0	0	1	1147	828	973
JAM3	-10.82411641	2.3463E-40	down	2	0	3	2354	1037	3251
JAML	-10.7483878	1.78258E-15	down	0	0	0	385	111	197
JAZF1	-0.90909713	0.008964686	down	455	580	504	787	380	923
JCHAIN	-9.352027565	2.67417E-10	down	0	0	1	110	302	62
JDP2	-1.110914402	6.88061E-06	down	255	308	258	518	286	470
JMJD4	1.567728265	2.30664E-17	up	582	624	595	149	149	132
JMJD6	1.127901684	5.95395E-08	up	1097	1115	1035	457	282	325
JMJD7	-0.959898289	0.005910155	down	35	36	32	56	39	48
JMY	-0.815651721	5.37946E-05	down	570	604	538	755	532	873
JOSD1	0.68631511	4.39709E-05	up	1815	1949	1867	750	805	924
JOSD2	-1.882983201	7.24574E-17	down	107	110	95	335	239	246
JPH1	3.306265215	5.64086E-07	up	593	758	709	23	93	24
JPH3	7.416057957	4.23199E-47	up	784	791	833	1	6	3
JPH4	-5.280155136	0.014126636	down	0	0	0	2	6	7
JRKL	1.150631025	4.68104E-07	up	967	1029	954	296	241	414
JTB	0.602789256	0.014924493	up	2783	3134	2746	1849	1040	1200
JUN	-2.631897849	0.000103862	down	940	991	1026	9804	1531	2206
JUND	-1.306401968	0.004714205	down	977	1064	1072	3080	823	1733
JUP	2.456242595	6.90786E-28	up	6573	6933	6899	932	972	715
KALRN	-6.073821021	1.05172E-32	down	52	37	41	1966	872	3584
KANK1	-2.259269351	4.17812E-32	down	1086	1136	1065	3953	2757	4586
KANK2	-3.237439895	2.85595E-32	down	945	1040	962	8033	3977	8095
KANK3	-5.782717474	1.58773E-20	down	9	3	9	169	386	252
KANK4	-3.000295178	0.009176298	down	21	42	24	52	381	16
KANSL1L	-3.708868639	1.72692E-21	down	61	84	50	635	337	863
KANSL2	0.614456041	0.000103787	up	922	921	859	475	390	390
KARS	0.878137385	2.96895E-11	up	4068	4469	4037	1817	1527	1511
KAT14	-2.006443644	6.02626E-17	down	55	73	70	215	173	177
KAT2B	-2.113957701	2.08346E-41	down	386	479	379	1247	1120	1455
KATNAL1	-0.612458361	0.030782878	down	471	589	450	483	641	487
KATNB1	1.28883171	3.60745E-10	up	457	477	495	127	144	143
KATNBL1	-0.761571614	3.33894E-06	down	354	426	367	540	384	462
KAZN	-1.270810595	7.69036E-06	down	122	170	147	200	260	285
KBTBD12	-9.647132682	2.19431E-12	down	0	0	0	55	132	121
KBTBD13	-7.397313987	8.24101E-06	down	0	0	0	22	34	8
KBTBD3	-1.967489227	6.26338E-09	down	87	70	74	271	132	252
KBTBD4	0.73171873	5.82877E-05	up	648	622	651	328	227	273
KBTBD8	-2.869121509	1.62364E-06	down	5	7	7	39	35	24
KCNA1	-6.992534016	0.017169727	down	0	0	0	49	0	4
KCNA2	-8.424314724	2.12279E-09	down	0	1	0	98	34	138
KCNA3	-8.124051655	6.64091E-08	down	0	0	0	16	47	44
KCNA4	-6.348727734	0.04674224	down	0	0	0	0	27	2
KCNA5	-11.70477238	4.2593E-15	down	0	0	0	463	83	816
KCNA6	-9.188947641	1.40536E-09	down	0	0	0	135	20	83
KCNAB1	-12.02956908	2.66213E-21	down	1	0	0	1232	617	1407
KCNAB2	2.748239031	4.94201E-57	up	2349	2329	2242	220	227	285
KCNB1	-8.936706622	1.33881E-11	down	0	2	0	89	392	174
KCNB2	2.659711948	0.010461442	up	120	124	129	21	0	23
KCNC3	-4.141059151	3.89089E-07	down	7	2	3	38	76	33
KCND2	-6.617680813	0.000155715	down	0	0	0	7	8	24
KCND3	-4.565923098	7.71147E-18	down	22	27	25	200	596	409
KCNE1	-5.291708751	0.000234078	down	0	2	1	6	51	21
KCNE1B	-7.291574838	0.007705606	down	0	0	0	0	44	13
KCNE2	-3.46403018	0.011154375	down	1	6	0	33	6	16
KCNE3	-7.190633599	1.52068E-17	down	4	1	0	118	218	174
KCNE4	-8.592275262	4.10375E-22	down	0	1	2	290	263	268
KCNG3	7.145217751	3.2499E-07	up	44	37	29	0	0	0
KCNH1	4.228492081	0.018527344	up	8	5	15	0	1	0
KCNH2	-4.427579527	0.000745784	down	5	11	7	23	286	8
KCNH3	2.341816339	2.62946E-08	up	80	91	85	17	10	9
KCNH4	2.911703545	1.70104E-05	up	38	40	38	6	3	2
KCNH5	6.838312042	1.2172E-06	up	31	31	27	0	0	0
KCNIP1	-5.564626149	0.004318104	down	0	0	0	9	3	7
KCNIP2	-6.519797934	7.10024E-08	down	14	19	24	54	3047	285
KCNIP3	-1.739977396	2.63092E-05	down	57	66	54	142	78	207
KCNJ12	3.508340092	3.21298E-46	up	836	819	854	55	59	42
KCNJ14	2.529318526	9.6194E-10	up	85	125	91	16	10	11
KCNJ16	4.446142921	0.000659347	up	19	26	17	0	1	1
KCNJ18	10.78350562	2.48268E-18	up	452	515	407	0	0	0
KCNJ5	-1.524039475	0.012849647	down	70	52	47	67	186	80
KCNJ6	8.197316976	2.49101E-10	up	76	76	76	0	0	0
KCNJ8	-10.18166835	9.28491E-20	down	2	0	0	464	688	470
KCNJ9	2.764022844	0.001772891	up	30	30	33	4	0	6
KCNK1	4.029993742	3.82239E-28	up	1799	1986	1882	47	103	92
KCNK13	-6.631240407	0.000155338	down	0	0	0	15	18	5
KCNK17	-11.37434245	1.15216E-05	down	0	0	0	907	11	184
KCNK2	-6.45227451	0.024614474	down	0	0	0	9	0	27
KCNK3	-3.883339687	6.64081E-05	down	122	133	137	479	3120	192
KCNK5	2.501865091	1.85756E-20	up	233	249	243	26	28	37
KCNK6	-4.778388573	2.14253E-17	down	16	15	20	334	134	558
KCNK7	-3.328850589	0.039748214	down	0	3	0	8	5	8
KCNMA1	-12.77627794	1.0361E-10	down	2	0	0	4040	174	6420
KCNMB1	-13.11775789	8.12365E-19	down	0	0	0	1891	190	1563
KCNMB3	-1.240743275	0.041652724	down	10	36	22	35	32	46
KCNMB4	-1.059108222	0.000965741	down	85	123	115	172	175	126
KCNN1	2.417146405	0.003076594	up	21	29	32	2	2	7
KCNN2	-5.155608497	0.007798643	down	0	1	1	2	41	3
KCNN3	-6.733558406	2.51764E-14	down	0	0	4	94	112	95
KCNN4	4.911501106	2.28061E-23	up	1210	1284	1244	51	9	31
KCNQ1	2.128757689	2.76676E-08	up	407	426	420	39	79	83
KCNQ2	5.699570331	5.80551E-05	up	23	31	25	0	1	0
KCNQ3	-7.43281238	8.48025E-10	down	0	1	1	93	102	46
KCNQ5	-5.679223813	3.40731E-07	down	5	0	0	60	35	93
KCNS2	-6.803943958	5.96654E-05	down	0	0	0	6	18	19
KCNS3	-3.070804512	2.23693E-09	down	29	59	37	401	118	240
KCNT1	-6.049006743	2.9886E-05	down	0	0	1	21	13	17
KCNT2	-7.741956262	1.04277E-16	down	1	0	4	149	393	199

KCNU1	5.259489033	0.003108205	up	8	13	9	0	0	0
KCTD10	-1.263956592	0.000985492	down	1470	1645	1527	3319	1276	3522
KCTD12	-2.657817956	4.0074E-140	down	926	1049	922	4365	3914	4706
KCTD15	-0.931087359	1.28628E-09	down	449	519	471	715	520	725
KCTD16	-3.56817971	0.001207671	down	4	1	1	31	10	11
KCTD18	-2.612166834	2.11664E-35	down	103	86	83	414	330	445
KCTD2	-1.123269373	0.000134927	down	440	450	428	701	803	509
KCTD5	1.914825795	9.54409E-32	up	1344	1467	1416	295	261	239
KCTD6	-0.658049676	0.006657232	down	163	184	167	247	153	180
KCTD8	-5.863292683	0.000794847	down	0	0	0	9	6	8
KDELC2	-0.756839164	0.039067506	down	1357	1591	1299	1861	914	2420
KDELR2	1.193386294	2.5382E-16	up	8411	9364	8038	3209	2188	2664
KDELR3	1.114348887	1.98956E-08	up	1123	1290	1182	425	411	336
KDM1A	2.949632767	1.12E-168	up	5885	6332	5933	566	494	612
KDM1B	2.14836736	1.02843E-28	up	1463	1583	1467	262	175	293
KDM2B	0.983386267	5.81581E-09	up	868	912	877	383	281	294
KDM5B	2.086926229	1.22639E-22	up	3739	3934	3545	514	657	690
KDM5C	1.889106958	2.02096E-20	up	5077	5612	5270	860	1080	1095
KDM5D	-12.9552852	8.2744E-05	down	0	0	0	1394	1	1884
KDM6A	0.693628989	0.00061405	up	811	924	866	337	400	397
KDM7A	-0.840353374	0.008795133	down	599	636	679	665	610	1183
KDM8	-1.325648567	0.010900755	down	34	16	26	61	34	43
KDR	-2.54027202	7.53433E-06	down	300	279	268	446	1789	1141
KDSR	-1.147604319	4.17177E-05	down	767	815	773	1451	762	1559
KEAP1	-1.741092275	3.80996E-23	down	401	347	328	943	792	826
KHDRBS1	0.917041724	0.000106599	up	5465	5903	5161	2763	1457	2079
KHDRBS3	-1.920823362	8.0988E-18	down	78	87	88	212	206	263
KHK	-1.879748576	0.000242999	down	31	33	22	121	42	66
KHNYN	-0.832817374	0.003013262	down	912	902	909	891	1018	1544
KHSRP	1.751295362	7.26727E-32	up	4279	4641	4265	864	838	1081
KIAA0100	0.872494616	0.008001533	up	5670	6035	5374	1526	2686	2293
KIAA0101	6.024342256	1.22106E-10	up	2134	2460	1963	57	15	1
KIAA0141	-0.691522073	0.000326054	down	734	910	756	814	911	1009
KIAA0226L	-9.050975439	1.41716E-10	down	0	0	0	122	38	53
KIAA0232	-1.080535723	1.09154E-11	down	868	913	818	1241	1133	1539
KIAA0319	4.746153773	5.07648E-06	up	41	36	37	0	2	1
KIAA0408	-6.050649265	0.002081352	down	0	0	0	3	14	8
KIAA0513	-0.641480924	0.011569149	down	232	239	238	298	181	317
KIAA0753	0.633172732	0.02028127	up	520	626	537	203	233	333
KIAA0754	0.803968658	0.038128162	up	522	582	565	140	213	324
KIAA0825	2.411813182	3.95911E-13	up	275	318	279	26	37	53
KIAA0895	3.595905396	6.50723E-20	up	992	1049	965	80	25	76
KIAA0907	0.850329855	0.001786974	up	1739	1883	1841	814	455	919
KIAA0922	-0.918009844	0.001311292	down	314	399	309	351	427	586
KIAA1024	2.774943319	3.4195E-10	up	218	201	192	35	17	12
KIAA1107	-2.323650206	1.6467E-06	down	61	32	39	107	135	232
KIAA1109	-1.963815129	8.71989E-14	down	856	845	861	2195	1709	3282
KIAA1161	-1.232804003	1.80979E-05	down	176	241	186	455	234	327
KIAA1211	2.673689156	2.92022E-23	up	433	461	391	45	34	65
KIAA1217	-1.411972888	9.98383E-05	down	193	205	218	275	497	370
KIAA1257	-1.786934212	0.012682832	down	5	7	8	22	16	11
KIAA1324	5.448593224	8.73905E-14	up	576	499	419	20	3	2
KIAA1328	-1.819316543	2.90242E-06	down	66	109	74	289	121	222
KIAA1456	-1.972388092	2.54028E-06	down	87	70	89	152	193	346
KIAA1462	-9.690769652	2.34534E-69	down	2	4	4	2022	1343	2575
KIAA1468	-1.758901066	1.34208E-17	down	256	279	240	745	453	684
KIAA1522	3.686141497	8.9058E-108	up	2547	2821	2610	136	128	177
KIAA1524	3.419162542	1.23955E-87	up	1393	1642	1335	112	77	102
KIAA1549	4.916073448	1.03615E-98	up	1917	1912	1753	39	34	59
KIAA1551	-1.592302642	9.81762E-15	down	424	475	439	820	995	1029
KIAA1614	-1.752063886	2.44499E-05	down	34	27	47	72	94	92
KIAA1644	3.065903467	1.3261E-05	up	532	563	575	23	92	19
KIAA1715	-0.587004921	0.016905869	down	566	642	634	532	684	735
KIAA1755	-6.618900302	8.17367E-30	down	3	7	7	646	228	336
KIAA1804	6.962262652	7.02573E-55	up	1073	1124	1087	11	0	8
KIAA1841	0.963129084	4.02017E-07	up	440	477	418	148	150	188
KIAA1958	2.201739166	2.64013E-17	up	841	963	848	102	137	168
KIAA2022	-8.729999058	3.60171E-08	down	0	0	0	67	12	94
KIAA2026	-1.453913002	3.50909E-12	down	334	363	318	635	519	833
KIDINS220	-1.035425239	2.68463E-08	down	1490	1562	1430	1984	1873	2687
KIF11	4.654974302	9.03022E-16	up	3053	3281	3091	186	46	41
KIF13A	-1.451519085	3.81341E-08	down	1177	1206	1113	1888	1815	3129
KIF13B	-1.047511935	9.67371E-07	down	443	420	439	587	538	798
KIF14	6.562285137	5.94635E-27	up	2186	2440	2073	35	12	4
KIF15	6.390388579	3.42714E-05	up	19	32	15	0	0	0
KIF18A	6.297439197	4.11732E-55	up	1133	1241	1036	19	7	5
KIF18B	5.878183655	1.35175E-11	up	960	1057	933	32	3	2
KIF19	-6.418641612	5.7577E-11	down	0	3	0	64	49	69
KIF20A	6.302039957	1.54963E-18	up	2419	2746	2449	46	20	3
KIF20B	3.796941585	5.4415E-102	up	2240	2309	2140	117	88	139
KIF21A	2.051435025	7.30435E-05	up	1789	1901	1895	160	505	256
KIF21B	2.481609811	5.99798E-20	up	633	703	621	77	58	116
KIF22	2.012821784	1.21855E-10	up	1421	1551	1473	376	230	180
KIF23	4.745045838	2.59132E-53	up	2755	2941	2681	108	41	77
KIF24	2.707929007	1.08029E-21	up	192	231	193	23	19	25
KIF26A	4.354866609	3.2727E-24	up	4136	4467	4339	93	225	117
KIF27	-0.906659784	0.007811978	down	83	97	98	115	87	172
KIF2A	2.075682916	4.88131E-26	up	1915	2257	1919	291	327	404
KIF2C	6.358377252	8.1731E-58	up	2831	2957	2755	44	16	15
KIF3B	1.226869328	7.53468E-08	up	3000	3457	2999	842	1085	904
KIF3C	-0.761255597	4.87975E-05	down	256	268	264	305	273	374
KIF4A	5.79648123	5.03931E-43	up	1659	1683	1481	27	26	8
KIF4B	5.662417195	0.000980485	up	12	9	18	0	0	0
KIF5A	-7.750035831	4.66702E-05	down	0	0	0	22	3	63
KIF5C	-9.315780837	6.956E-12	down	0	0	0	111	41	104
KIF7	-3.474039967	6.03936E-17	down	33	22	25	316	158	168
KIFAP3	-0.857908776	3.70932E-05	down	596	716	595	967	792	687
KIFC3	2.420522569	1.45661E-13	up	3131	3236	3046	331	548	343
KIT	-1.245490932	0.012527903	down	176	193	201	498	128	365
KL	-9.6822894	5.69216E-12	down	0	0	0	113	151	50
KLC2	0.813908919	5.27252E-06	up	746	755	729	274	282	345

KLC3	6.979718913	1.18095E-07	up	71	64	56	1	0	0
KLF12	-1.035102608	1.01451E-06	down	550	629	546	918	600	1018
KLF15	-8.170588996	4.50181E-33	down	8	6	2	465	1107	1669
KLF16	1.257558343	2.48407E-07	up	566	593	576	196	182	135
KLF17	3.617589567	5.44053E-06	up	79	72	60	0	6	6
KLF2	-4.707573051	3.55769E-23	down	69	73	57	2024	1088	597
KLF3	-0.628937266	2.21286E-05	down	1031	1103	1011	1177	950	1345
KLF5	4.200098949	1.83235E-47	up	4033	4313	4148	238	103	150
KLF6	-2.519621255	2.44646E-18	down	881	862	851	4049	2117	4611
KLF7	-1.144982892	0.000260573	down	617	659	598	962	619	1408
KLF8	-6.971161054	7.03496E-59	down	9	7	6	534	576	854
KLF9	-3.29592721	2.41496E-48	down	392	401	400	2506	2224	3652
KLHDC1	-7.306283547	1.37499E-22	down	1	5	0	224	150	303
KLHDC2	-1.753892784	6.71045E-32	down	491	563	464	1209	1034	1400
KLHDC8A	-5.132199799	0.032397428	down	0	0	0	2	9	2
KLHDC8B	-7.687159162	4.58553E-26	down	1	8	8	642	1295	444
KLHDC9	-1.192253354	0.022920659	down	30	30	25	68	23	50
KLHL10	-6.373272733	4.14065E-05	down	1	0	0	9	27	26
KLHL13	2.964788272	3.63292E-25	up	1753	2017	1753	214	97	200
KLHL14	-6.189860659	0.000353778	down	0	0	0	8	13	7
KLHL15	1.547727449	1.00224E-18	up	798	1033	871	258	189	210
KLHL17	1.179670823	0.000235192	up	238	306	263	122	63	70
KLHL2	-0.853534032	0.00164793	down	314	320	291	392	278	531
KLHL20	-0.996205883	2.01469E-06	down	360	391	351	445	516	593
KLHL21	-0.639237992	0.008193782	down	1054	1033	935	1047	855	1470
KLHL23	4.506827989	0.048795909	up	6	10	2	0	0	0
KLHL24	-1.297432812	0.005164241	down	644	609	622	527	1159	1560
KLHL25	-1.089792267	0.008234573	down	87	99	79	103	178	110
KLHL26	0.80499461	0.001563802	up	306	358	284	169	97	122
KLHL28	-1.435869092	2.22702E-06	down	148	156	135	238	217	394
KLHL3	-4.335648252	2.88457E-31	down	28	39	42	507	643	386
KLHL32	-2.633741598	0.002529115	down	3	4	2	19	9	12
KLHL33	-6.058661669	1.14085E-05	down	2	0	0	15	30	50
KLHL35	3.040897659	1.29758E-16	up	375	416	388	55	23	25
KLHL36	-0.914120108	5.85387E-06	down	638	657	656	806	729	1090
KLHL38	-1.767338752	0.000802868	down	26	25	31	43	92	59
KLHL4	-3.842052831	0.000142795	down	1	3	4	28	41	10
KLHL41	-3.006275728	0.003341837	down	48	57	47	89	662	46
KLHL42	-3.525428243	5.63633E-08	down	291	307	278	2862	505	4085
KLHL6	-7.541137969	4.60025E-11	down	0	2	0	91	97	73
KLHL9	-0.807003643	5.70891E-06	down	746	728	743	1029	707	1048
KLK1	5.280889044	0.00041892	up	14	22	23	0	0	1
KLK10	4.938709056	0.016927024	up	11	10	3	0	0	0
KLK6	4.820276615	0.012060222	up	7	8	7	0	0	0
KLKB1	-7.173468877	8.15297E-07	down	0	1	0	64	19	30
KLRB1	-8.888458558	1.54344E-10	down	0	0	0	79	71	34
KLRC1	-6.512777939	0.000757718	down	0	0	0	9	3	25
KLRC4	-5.470757518	0.02980209	down	0	0	0	2	1	15
KLRD1	-2.784482062	0.001238356	down	3	7	8	11	31	45
KLRF1	-7.151151479	1.008E-06	down	0	0	0	24	16	16
KLRG2	8.186283949	3.50896E-10	up	84	72	70	0	0	0
KMT2B	0.678614443	0.001198565	up	1079	1189	1183	457	441	638
KMT2D	1.249700064	2.08235E-06	up	3513	3949	3868	897	971	1524
KNOP1	1.956800475	2.4162E-18	up	855	1112	871	219	153	147
KNSTRN	3.867400012	1.39142E-80	up	1811	1895	1762	107	87	72
KNTC1	2.688924259	8.10204E-73	up	1581	1855	1714	182	174	211
KPNA1	0.936826146	2.35356E-05	up	3131	3319	3006	967	1251	1259
KPNA2	4.104433355	8.87993E-55	up	6496	7367	6474	359	279	199
KPNA5	-2.014555776	6.09566E-14	down	132	129	109	470	254	351
KPNA7	8.008819868	1.07646E-09	up	60	66	74	0	0	0
KPNB1	1.637305545	1.36269E-22	up	10114	10496	9636	2099	2372	2393
KRAS	0.887086105	2.22689E-08	up	1407	1553	1426	682	463	549
KRBA1	-1.292227433	0.000372163	down	78	54	76	138	82	149
KRBA2	-1.860933472	0.000379815	down	41	29	28	48	106	95
KRBOX1	-7.908611532	4.7567E-06	down	0	0	0	55	33	6
KRCC1	-2.331142071	5.60117E-34	down	178	161	174	659	605	569
KREMEN1	-2.07438948	1.90761E-08	down	127	169	137	282	530	455
KREMEN2	10.05606122	7.49325E-16	up	237	302	290	0	0	0
KRI1	0.658521413	0.002098393	up	707	776	721	397	235	371
KRIT1	-0.743267204	2.63593E-07	down	611	699	667	787	677	893
KRR1	0.703044624	9.02341E-05	up	1490	1618	1410	758	491	740
KRT10	0.954873005	0.000315671	up	1240	1296	1098	620	337	388
KRT14	-6.921569269	2.28333E-09	down	2	5	0	265	282	28
KRT15	9.670297333	1.25016E-14	up	190	213	230	0	0	0
KRT18	6.368484856	2.11076E-16	up	13186	14314	13253	51	245	31
KRT19	-3.415788337	0.006127867	down	9	2	14	160	6	38
KRT222	-8.990623575	4.44881E-11	down	0	0	0	83	34	87
KRT7	4.304233041	1.39986E-17	up	4100	4513	4197	130	243	71
KRT8	5.099603041	3.43797E-75	up	18179	20045	18213	516	230	445
KRT80	7.941306665	6.7304E-153	up	3446	3911	3356	13	8	10
KRT86	2.384690075	0.011636722	up	40	52	36	10	0	8
KRTAP13-2	4.891932193	0.013338155	up	5	7	11	0	0	0
KRTCAP3	-4.519314665	0.001721896	down	0	1	1	10	6	17
KSR1	-0.744241507	0.001655034	down	315	360	387	402	458	394
KY	-6.412498149	3.14005E-06	down	0	0	2	45	57	17
KYAT1	1.882317256	0.000247018	up	467	500	460	101	33	149
KYAT3	-0.925916482	0.002110057	down	408	451	354	624	603	392
KYNU	2.188543326	9.93327E-13	up	336	368	364	66	32	71
L1CAM	5.790101271	1.66E-56	up	8280	9237	8711	78	158	92
L1TD1	-5.047914075	0.009933633	down	1	0	0	6	16	2
L3HYPDH	-1.26761774	3.85507E-08	down	272	310	237	408	412	579
L3MBTL4	-7.588819675	4.42941E-07	down	0	0	0	14	32	28
LACC1	-1.366332578	7.65824E-09	down	157	229	214	339	368	384
LACTB	1.712980889	2.73899E-07	up	1569	1602	1405	231	417	324
LAG3	-1.938128207	0.00200088	down	8	13	10	32	34	17
LAGE3	1.257484916	0.004604192	up	571	540	492	195	194	81
LAIR1	-8.277764596	4.5462E-08	down	0	0	0	39	61	18
LAMA1	6.003618204	1.32498E-08	up	1269	1469	1244	8	33	0
LAMA2	-8.761493309	1.99286E-54	down	22	19	10	2294	5085	8216
LAMA3	-1.508171382	4.37975E-08	down	303	348	347	516	626	871
LAMA4	-7.098856545	5.81158E-50	down	27	30	22	1864	3845	1708

LAMB2	-4.530585705	5.79645E-82	down	339	347	412	6208	4392	7672
LAMB3	3.199640321	0.000655321	up	1896	2008	2081	72	338	20
LAMC1	-0.853987919	0.00125546	down	6777	7488	6484	6996	7743	11910
LAMC2	-1.250395918	0.004594079	down	70	44	51	72	115	89
LAMC3	3.523743214	1.58961E-21	up	1216	1335	1150	126	50	56
LAMTOR1	-0.803829333	2.5878E-06	down	924	992	849	1247	1140	1027
LAMTOR3	-1.685398061	4.3131E-12	down	345	443	323	1100	613	845
LANCL1	-0.626396792	7.11979E-06	down	1379	1571	1346	1519	1403	1788
LANCL2	-1.023887105	0.001195009	down	148	145	125	169	241	185
LAP3	-2.028467189	1.70514E-11	down	506	510	467	1251	1795	1173
LAPTM4A	-0.804865592	2.12271E-05	down	4923	5174	4804	6522	4604	7551
LAPTM4B	2.3692738	1.88722E-10	up	9105	9621	8476	1090	1713	840
LAPTM5	-7.116082196	3.70221E-56	down	30	20	26	3907	1251	2574
LARGE	-7.031175172	1.88744E-57	down	8	4	4	475	509	496
LARP1	1.230158756	1.08829E-06	up	6374	6921	6344	1564	2247	2058
LARP4	1.811623086	3.48361E-10	up	3538	3843	3313	536	862	729
LARP6	-1.840127044	2.6167E-15	down	248	275	276	751	474	833
LARP7	-1.365807141	1.38436E-20	down	635	786	599	1341	1034	1328
LARS2	2.023576441	1.29557E-10	up	1010	1237	1034	137	235	190
LAS1L	1.082973305	2.32862E-08	up	1492	1711	1536	614	382	609
LASP1	1.465868389	9.27739E-45	up	9518	9950	9413	2715	2292	2426
LAT	-4.019320847	2.03729E-08	down	9	26	12	313	55	185
LAT2	-2.266748552	0.001464821	down	54	47	43	352	55	105
LATS1	-0.788716425	1.45882E-05	down	566	619	444	713	564	725
LAX1	-4.947743457	0.000223352	down	0	3	0	11	27	25
LAYN	-7.945948171	1.69718E-21	down	1	3	3	560	126	580
LBH	-2.414743503	5.41467E-14	down	628	530	538	1917	1444	3167
LBR	1.596385558	2.01427E-24	up	3690	3887	3158	976	762	785
LBX2	1.174698172	0.044089343	up	35	61	66	15	23	12
LCA5	-2.312692929	3.57596E-13	down	83	105	66	262	231	405
LCAT	-2.212373337	6.37293E-06	down	60	46	60	257	80	228
LCN10	-2.448554489	0.004142801	down	76	64	63	93	80	640
LCN15	4.594241789	0.006471196	up	6	18	13	0	0	1
LCN2	4.249562286	9.90866E-06	up	47	58	80	0	1	6
LCN6	-10.34368391	7.09811E-11	down	0	0	0	49	74	398
LCOR	0.738833345	0.002953832	up	732	817	838	296	280	445
LCORL	0.658032479	0.00177436	up	341	364	363	145	164	170
LCP1	-8.137012061	8.55085E-58	down	8	6	4	1591	670	1417
LCP2	-11.02341136	3.08054E-18	down	0	0	0	330	177	322
LDB1	-0.597853838	0.010622473	down	1141	1275	1155	1617	882	1391
LDB2	-13.34352812	1.13455E-26	down	0	0	0	1233	1585	1202
LDB3	-6.257564364	7.50567E-27	down	38	45	56	1461	4342	1417
LDHA	2.205793173	1.64465E-12	up	55233	59386	54197	6533	11049	7862
LDHB	1.393193885	0.00068445	up	23752	25628	21647	5147	9164	4315
LDHC	3.472811889	0.015206956	up	26	22	35	0	5	0
LDHD	-2.882768649	0.003507318	down	107	106	123	99	1339	188
LDLR	2.378126879	5.87112E-06	up	2196	2196	2092	246	470	138
LDLRAD2	-3.320050598	5.92019E-05	down	2	2	8	38	27	21
LDLRAD3	1.701515205	0.003321157	up	945	961	909	100	347	141
LDLRAD4	-4.807769265	4.28807E-22	down	60	59	47	1015	471	1902
LDOC1	-2.745569768	0.00135282	down	331	340	338	2612	143	2295
LEF1	1.485303993	0.011406373	up	127	113	123	58	20	16
LEFTY1	-2.424490248	0.014571252	down	2	4	3	19	5	11
LEFTY2	-7.689287657	4.73182E-09	down	2	2	0	511	36	64
LEO1	1.259644927	1.7821E-09	up	966	1083	850	255	304	293
LEP	-11.50634416	6.79896E-05	down	0	0	0	7	916	118
LEPR	-2.790706851	7.71793E-18	down	192	212	209	816	727	1500
LEPROT	-2.212347094	5.21471E-35	down	906	1000	815	3284	2247	3498
LEXM	5.092158455	0.009094913	up	7	15	5	0	0	0
LFNG	-0.883515194	2.62602E-06	down	168	191	171	251	186	261
LGALS1	-0.887247512	0.001244315	down	5958	6593	6145	11486	6873	6366
LGALS12	-11.7829582	2.93011E-06	down	2	0	0	41	4344	241
LGALS2	-6.239635485	0.004374391	down	0	0	0	25	2	4
LGALS3	-0.660657736	0.017026488	down	2184	2462	2227	2815	2899	1915
LGALS3BP	-3.618676814	1.22409E-27	down	313	353	299	2168	3681	2367
LGALS8	-5.365927385	6.71243E-96	down	41	38	44	1041	1237	1309
LGALS9	-6.001136042	1.44107E-51	down	12	4	12	443	427	407
LGALS9B	6.841678577	4.03593E-06	up	18	41	31	0	0	0
LGALS9C	6.331476938	4.83252E-05	up	26	14	22	0	0	0
LGALSL	-0.92951902	0.000324499	down	403	406	381	696	360	579
LGI1	-8.954019733	1.08499E-05	down	0	0	0	116	1	88
LGI2	-11.39695265	9.46731E-20	down	0	0	0	289	360	402
LGI3	2.008439578	0.02674453	up	79	99	83	26	1	21
LGI4	-8.393854675	2.79058E-31	down	5	8	4	706	651	2762
LGMN	-1.489232907	4.76411E-18	down	1255	1370	1143	2318	2579	2560
LGR4	1.759736439	1.48724E-14	up	5065	5548	5127	905	973	1426
LGR5	-4.306552334	0.000222289	down	4	1	1	26	7	55
LGR6	-4.638662855	5.23698E-07	down	57	82	85	1764	82	2307
LGSN	2.884225519	2.43586E-09	up	70	87	94	6	10	8
LHCGR	-7.493503754	0.006322735	down	0	0	0	0	54	11
LHFP	-4.785768079	3.09682E-61	down	198	211	198	5703	3284	2999
LHFPL1	-7.407731428	8.02689E-06	down	0	0	0	41	7	21
LHFPL2	-0.601934985	0.002702052	down	781	952	848	816	950	995
LHPP	-0.897905761	0.007536643	down	156	125	132	168	132	254
LHX2	5.348702066	1.4366E-05	up	218	212	192	0	10	0
LHX4	5.515075305	4.37147E-25	up	211	223	210	2	4	4
LHX6	-1.351652996	0.013890099	down	96	107	118	149	304	109
LIAS	-1.685932465	6.95571E-08	down	101	135	85	236	279	206
LIF	5.31825544	7.63401E-58	up	638	617	646	16	10	8
LIG1	1.337753543	4.18286E-11	up	416	407	440	127	118	110
LIG3	1.460808865	4.16303E-11	up	839	945	857	186	234	257
LILRB2	-5.236909814	0.01188158	down	0	0	0	4	3	8
LIMD2	-5.293389173	8.97326E-19	down	4	7	6	260	107	113
LIME1	-0.8457753	0.046298918	down	75	65	67	128	79	59
LIMK1	2.383470227	4.52576E-70	up	1398	1508	1330	212	176	189
LIMK2	-1.134578094	0.00013922	down	191	238	232	286	407	322
LIMS1	0.673366633	6.71039E-08	up	4193	4488	4068	1863	1846	1952
LIMS2	-2.578620192	2.99559E-10	down	449	475	435	2735	856	2340
LIMS4	-1.802700963	0.000331622	down	20	16	26	56	31	70
LIN54	1.078991177	4.99655E-14	up	601	630	568	211	179	216
LIN7A	-10.79230636	1.03308E-14	down	0	0	0	95	325	255

LIN7B	-1.884007399	3.69006E-05	down	34	34	32	139	55	73
LIN7C	1.456311079	2.35419E-15	up	1768	1847	1754	587	371	443
LIN9	2.235448054	2.71989E-08	up	782	879	790	107	174	79
LINC00176	-1.859986578	0.041553604	down	5	5	8	29	6	13
LINC01272	-1.517572612	0.019361811	down	28	26	24	31	85	37
LINGO1	-8.060642776	4.02922E-09	down	0	1	0	79	83	39
LINGO2	6.097790598	8.10117E-11	up	97	99	94	0	2	1
LIPC	-5.545020606	0.01522012	down	0	0	0	9	8	1
LIPE	-5.430349823	0.00055779	down	102	102	79	83	7143	643
LIPG	-6.374607735	2.75927E-05	down	2	0	1	45	119	5
LIPH	3.67244279	4.44655E-12	up	352	339	357	19	6	35
LIPJ	-6.108361743	3.03044E-05	down	1	0	0	15	21	16
LIPT1	-0.950956622	5.01009E-06	down	103	123	112	171	143	150
LLGL1	0.891344537	3.51684E-10	up	836	833	826	337	272	351
LLGL2	4.030110443	1.4938E-144	up	1821	1975	1918	92	74	83
LMAN1	1.909881065	2.50004E-39	up	9454	10472	9531	1725	1843	1974
LMAN2L	0.629894597	0.000937166	up	688	736	683	380	306	281
LMBR1	0.629068203	0.000241521	up	1363	1537	1395	593	628	746
LMBRD1	-1.991627605	3.05601E-21	down	422	455	429	1439	866	1431
LMBRD2	0.848657362	8.77982E-05	up	1289	1330	1195	443	427	639
LMCD1	-4.408263881	3.04304E-13	down	196	213	193	4319	712	4410
LMF1	-1.599552546	4.3395E-10	down	146	187	197	470	274	410
LMF2	-1.25515153	6.16186E-15	down	395	410	408	816	584	668
LMNA	0.792558719	0.008808515	up	12013	13121	12270	7433	3311	4864
LMNB1	4.219536808	7.94174E-14	up	2544	2863	2436	197	68	39
LMNB2	3.034088398	2.49227E-81	up	3005	3262	3076	283	277	247
LMNTD2	3.4836118	0.030706284	up	21	6	18	3	0	0
LMO1	3.981158589	8.46615E-18	up	162	190	159	4	6	13
LMO2	-8.951316	8.73411E-31	down	5	1	1	1436	461	638
LMO3	-12.41380061	1.17844E-09	down	0	0	0	651	81	1502
LMO4	-1.980471966	6.1012E-10	down	675	717	584	2778	1426	1389
LMO7	1.969860496	1.81363E-40	up	6706	7210	6739	1228	1044	1490
LMOD1	-13.74749401	8.4573E-38	down	2	3	0	19287	2278	28884
LMTK2	1.433196652	4.53053E-11	up	1315	1454	1288	293	337	434
LMX1A	-5.313116176	0.013013854	down	0	0	0	5	2	9
LNP1	-1.640739864	5.04736E-07	down	89	99	93	300	144	187
LNX1	-5.920058516	2.11355E-21	down	6	2	3	117	137	222
LOC100128108	-8.718633232	9.37023E-10	down	0	0	0	96	31	42
LOC100128905	-6.471780185	0.001171938	down	0	0	0	8	22	3
LOC100129924	4.038918645	0.018187751	up	7	10	8	0	1	0
LOC100129940	-6.400332717	0.024938573	down	0	0	0	0	16	16
LOC100130345	-4.877931998	0.007387579	down	0	0	1	6	3	14
LOC100132874	3.868763755	0.028508469	up	8	6	8	1	0	0
LOC100287896	-1.191864866	0.003316218	down	85	72	47	135	70	131
LOC100506127	1.356960664	2.17058E-07	up	225	293	212	84	52	67
LOC100506504	-2.335997641	0.00083806	down	8	4	4	20	15	23
LOC100506514	-6.236051893	0.000270392	down	0	0	0	9	6	15
LOC100509620	-5.522756215	0.000953927	down	2	3	4	0	216	56
LOC100653049	5.224902615	0.003614203	up	12	9	8	0	0	0
LOC100996643	-7.797130657	0.006138708	down	0	0	0	0	74	5
LOC100996693	-7.495743967	5.1535E-05	down	0	0	0	56	5	13
LOC101060389	4.124076573	0.023343259	up	10	4	12	0	1	0
LOC101927245	1.575690446	0.042997238	up	31	25	39	14	6	3
LOC101927322	2.228265743	0.049864878	up	21	16	24	3	6	0
LOC101927375	4.745027105	0.019285367	up	8	9	4	0	0	0
LOC101927401	-5.707230789	0.005833085	down	0	0	2	5	62	2
LOC101927572	3.206441964	0.00060658	up	35	32	49	6	3	0
LOC101927789	2.885842348	8.20086E-07	up	525	519	537	37	87	22
LOC101928120	-1.626196415	0.014308719	down	12	10	6	30	15	17
LOC101928212	4.540807044	0.027810998	up	7	5	6	0	0	0
LOC101928422	5.30729441	0.000363432	up	24	21	15	0	0	1
LOC101928548	3.705535973	0.016552249	up	9	12	15	2	0	0
LOC101928635	3.0847212	0.038919917	up	14	20	17	0	4	0
LOC101928722	5.01458156	0.007088014	up	9	7	9	0	0	0
LOC101928764	-3.677196378	0.000589249	down	4	3	0	28	9	28
LOC101928841	1.02545507	0.027700972	up	738	740	724	128	331	293
LOC101929726	-8.374180773	6.08589E-05	down	0	0	0	3	104	12
LOC101929796	-1.301227396	0.001738631	down	26	35	23	43	40	64
LOC101929839	2.542152261	0.000518975	up	24	56	28	4	4	5
LOC101929895	4.675109489	4.80555E-21	up	234	257	201	7	10	2
LOC102723415	-4.833179519	4.68099E-05	down	2	3	0	57	8	39
LOC102724023	-2.290800105	8.02926E-05	down	71	96	55	303	346	100
LOC102724135	5.756909534	0.000384329	up	12	14	16	0	0	0
LOC102724428	2.974475307	1.81619E-05	up	875	1021	926	70	151	22
LOC102724474	4.109929112	3.9197E-06	up	112	107	86	0	9	3
LOC102724488	-5.071966597	1.20874E-06	down	1	5	0	23	49	67
LOC102724957	-6.983807803	0.020523332	down	0	0	0	0	42	3
LOC102724965	-1.231672548	0.023538479	down	15	16	13	23	17	34
LOC102724985	1.391261027	3.64029E-09	up	334	327	404	100	94	95
LOC102724993	-1.520819045	0.006196791	down	26	39	37	91	29	93
LOC105369201	6.547821149	1.42825E-11	up	619	661	556	0	12	1
LOC105369205	-7.107672975	1.8506E-12	down	5	2	0	485	82	146
LOC105369212	-4.439545298	1.84025E-10	down	5	2	12	131	56	116
LOC105369972	6.494578004	1.3652E-05	up	18	23	29	0	0	0
LOC105370092	4.727759576	0.024485131	up	5	12	4	0	0	0
LOC105370295	6.078179071	0.000127054	up	12	24	17	0	0	0
LOC105370579	3.203064154	0.015341078	up	18	11	9	2	0	1
LOC105370708	4.682504951	9.35637E-06	up	33	34	42	1	2	0
LOC105371047	-4.851960962	0.00427385	down	0	1	0	5	7	10
LOC105371303	2.299139507	1.3068E-07	up	74	73	68	12	11	8
LOC105371409	5.670561216	7.3985E-05	up	22	25	30	0	1	0
LOC105371921	6.202721587	8.11888E-06	up	41	44	27	0	0	1
LOC105371932	-1.543192674	0.00496618	down	11	25	22	30	46	42
LOC105372109	6.869748429	3.33647E-06	up	36	20	34	0	0	0
LOC105372836	1.622370573	0.018564141	up	18	24	27	5	4	7
LOC105372883	-5.485099073	1.05539E-06	down	5	1	0	22	70	98
LOC105373132	3.724335305	4.02579E-12	up	300	378	311	35	10	9
LOC105373244	-7.132101707	6.577E-06	down	0	0	0	15	10	31
LOC105373759	-2.53496932	0.01765711	down	2	5	1	6	13	13
LOC105374013	-1.941342614	0.001070281	down	54	65	31	65	119	222
LOC105374811	-2.249466362	0.000678178	down	19	18	23	28	61	114

LOC105375254	4.620230526	0.029937208	up	10	5	4	0	0	0
LOC105375355	8.502129648	7.39895E-11	up	73	99	110	0	0	0
LOC105376204	-3.802912274	0.00078843	down	5	3	0	10	30	38
LOC105376339	-7.05705481	3.41799E-05	down	0	0	0	22	5	27
LOC105376353	-4.469352103	0.00733491	down	1	0	1	10	2	21
LOC105376678	3.247896543	0.038790514	up	9	5	12	1	0	1
LOC105376714	5.328056132	0.0034152	up	8	8	15	0	0	0
LOC105377622	4.490805136	0.000172984	up	62	67	73	0	6	0
LOC105378220	13.4345006	2.43476E-28	up	2710	3162	2752	0	0	0
LOC105378230	-4.191389063	0.004827017	down	0	0	3	7	15	17
LOC105378862	-7.780282219	1.65134E-06	down	0	0	0	59	12	18
LOC105379282	-1.637759052	0.028274352	down	16	21	16	14	58	41
LOC105379575	4.16597114	0.014558445	up	9	7	11	0	0	1
LOC107983953	-3.736786747	0.000867836	down	2	0	2	13	14	11
LOC107983962	-4.12859362	0.003346428	down	0	0	3	11	10	17
LOC107983990	-7.194583805	3.33298E-05	down	0	0	0	15	33	7
LOC107983997	1.71674649	0.003292949	up	138	147	129	19	15	57
LOC107983998	-1.906333935	0.000541283	down	37	35	32	56	63	161
LOC107984124	-4.733751199	0.011096713	down	0	1	0	11	2	8
LOC107984590	6.335797507	3.00944E-05	up	24	24	15	0	0	0
LOC107984638	-1.922564088	0.008163117	down	7	5	11	26	23	13
LOC107984751	4.1577225	4.48517E-05	up	26	43	34	0	3	1
LOC107984814	2.703467484	0.000215776	up	30	30	40	6	3	2
LOC107984817	5.509167882	0.000170435	up	28	24	17	0	1	0
LOC107984851	-8.602187598	1.11804E-07	down	0	0	0	100	11	48
LOC107984859	-5.359092786	0.001367966	down	1	0	0	9	5	18
LOC107984974	-5.413050815	1.55734E-10	down	5	0	2	62	48	107
LOC107985184	-2.463133083	0.007088893	down	4	2	5	11	9	24
LOC107985246	3.636903693	2.44815E-21	up	393	466	374	15	33	21
LOC107985425	9.571381563	1.82779E-14	up	186	210	196	0	0	0
LOC107985433	-6.271879312	0.000262151	down	0	0	0	14	5	12
LOC107985532	3.405627035	0.001178237	up	27	22	24	1	0	4
LOC107985687	2.847279633	0.001126268	up	28	23	19	4	1	2
LOC107985729	3.640260662	0.049365535	up	7	8	4	0	0	1
LOC107985770	-6.64180879	2.63252E-05	down	1	0	0	46	8	25
LOC107985876	-3.046800995	0.000137899	down	8	8	5	14	48	59
LOC107986163	-3.928752221	6.90783E-06	down	1	3	5	29	46	20
LOC107986192	-7.050245181	1.14007E-06	down	1	1	0	116	10	70
LOC107986554	6.975195269	5.51498E-07	up	29	36	33	0	0	0
LOC107986827	-6.089027521	4.9204E-05	down	0	0	1	26	10	17
LOC107986838	-1.841902873	0.007831201	down	7	8	11	15	30	20
LOC107987020	-3.392932965	0.002456975	down	1	3	1	10	18	8
LOC107987142	1.228401982	0.015211364	up	30	53	43	13	12	13
LOC107987423	-6.920174917	0.000135878	down	0	0	0	4	23	19
LOC283278	1.723088855	0.021660226	up	32	45	29	4	13	5
LOC388282	6.363244929	1.70085E-05	up	20	22	22	0	0	0
LOC389602	6.041925215	0.000162895	up	13	15	23	0	0	0
LOC401052	-2.391070984	5.029E-05	down	6	12	6	28	33	27
LOC441239	-5.809105419	0.001382156	down	0	0	0	10	7	5
LOC643355	-4.533878786	0.008402196	down	1	2	0	2	36	7
LOC644215	-4.747481234	0.007453204	down	0	1	0	8	3	10
LOC646588	-6.688161752	1.0299E-09	down	1	1	1	137	41	47
LOC653513	-9.452361788	3.3508E-20	down	4	0	2	438	1954	461
LOC653602	-7.432458812	0.00011334	down	0	0	0	56	4	11
LOC728392	-1.975967651	9.47481E-14	down	73	79	53	228	169	176
LOC730268	1.021862533	0.036528993	up	61	72	74	14	25	33
LOC81691	2.120294612	1.9891E-10	up	301	386	250	56	58	37
LONP1	1.237106232	0.000358901	up	2774	3065	2848	733	1158	666
LONP2	-0.642482846	0.017792733	down	927	978	896	784	1100	1194
LONRF2	-4.759847419	9.16343E-48	down	19	22	17	434	256	439
LOXHD1	-7.876640397	0.004168976	down	0	0	0	0	75	9
LOXL1	-4.364998391	1.90818E-39	down	52	45	65	1104	539	777
LOXL2	2.310883029	0.000434113	up	3915	4089	4173	293	1087	270
LOXL3	-4.34689884	8.09052E-21	down	22	14	24	403	154	337
LOXL4	-3.718917968	7.38872E-18	down	24	20	25	242	118	301
LPAR1	-10.9638491	1.06252E-27	down	0	4	0	2148	630	2979
LPAR4	-4.500075511	1.27933E-08	down	4	9	0	89	54	64
LPAR5	1.505420065	0.000382556	up	227	237	219	81	26	68
LPAR6	-13.56399118	1.00511E-28	down	0	0	0	1762	1464	1526
LPCAT1	3.053339789	8.42652E-29	up	311	341	322	31	18	35
LPCAT2	-1.296168634	1.41228E-10	down	322	353	293	500	580	596
LPIN1	-2.517454823	0.002499187	down	541	621	600	619	5033	1014
LPIN3	-0.986249269	0.009273405	down	366	411	367	348	464	798
LPL	-4.214759629	0.006841635	down	1070	1048	958	422	33135	3399
LPP	-3.167600308	1.05621E-08	down	1826	2179	2072	13900	3930	22260
LPXN	-3.522793618	7.06709E-14	down	33	54	23	379	153	377
LRAT	-4.844665068	0.0484143	down	0	0	0	1	5	5
LRCH1	-1.918202645	2.78919E-07	down	329	330	334	1052	450	1231
LRCH2	-7.66078113	7.47841E-18	down	0	3	2	360	96	280
LRCOL1	-5.056497063	0.036726758	down	0	0	0	1	4	8
LRFN1	-7.167165704	8.40684E-06	down	0	0	0	32	8	18
LRFN4	2.96371801	4.98786E-16	up	1215	1284	1364	112	157	76
LRFN5	-8.427037898	1.3844E-10	down	0	0	0	49	42	44
LRIF1	2.439290683	2.05629E-12	up	1519	1583	1392	237	227	118
LRIG1	-2.494446693	5.7968E-17	down	91	95	96	282	368	475
LRIG3	-1.749169274	3.57018E-11	down	231	226	216	600	354	678
LRIT3	-4.773499171	0.005289189	down	1	0	0	10	6	5
LRMP	-10.43131261	1.42981E-16	down	0	0	0	156	161	225
LRP1	-1.337163582	1.98794E-06	down	7403	8458	8823	11236	13160	19946
LRP10	-1.591850965	9.04224E-08	down	1427	1441	1523	3152	1993	4423
LRP11	1.264629251	1.52243E-10	up	1649	1824	1747	540	381	637
LRP1B	-1.919430152	0.025615562	down	10	20	19	21	19	94
LRP2BP	-2.295986986	0.000956661	down	21	26	22	111	21	117
LRP3	-0.920864203	0.000123691	down	394	441	402	480	622	542
LRP4	1.955432452	2.19198E-06	up	560	639	595	62	145	114
LRP5L	1.114681027	0.011389239	up	188	224	204	40	58	104
LRP8	3.66063452	3.30493E-14	up	2619	3030	2718	184	55	245
LRPAP1	-1.025781888	9.04794E-14	down	1315	1413	1320	2308	1671	1902
LRPPRC	1.447426269	1.4663E-06	up	7569	8287	7382	1606	2532	1793
LRR1	1.522857465	0.00029715	up	367	393	300	71	123	61
LRRC17	-7.89501535	5.0095E-35	down	3	6	5	1401	528	473

LRRC2	-6.000964006	2.01702E-05	down	13	3	1	12	617	88
LRRC25	-6.867272617	1.19879E-16	down	1	0	3	132	83	121
LRRC26	7.552571124	4.9276E-09	up	79	107	99	0	1	0
LRRC27	-1.040802669	0.001816058	down	60	72	74	82	113	103
LRRC28	-0.925111526	1.87397E-06	down	209	215	199	326	261	254
LRRC29	-4.114383374	3.27583E-06	down	1	4	2	44	24	18
LRRC3	0.786687275	0.018156218	up	135	146	101	65	49	43
LRRC31	6.662796929	3.43256E-06	up	23	30	26	0	0	0
LRRC32	-7.001204771	1.7927E-80	down	12	18	21	1652	1753	1183
LRRC34	-8.828277195	1.67871E-11	down	0	0	0	63	64	50
LRRC37A3	-0.901630624	0.002497335	down	62	72	49	82	68	93
LRRC37B	-1.15397828	2.11864E-06	down	95	95	94	166	113	174
LRRC38	6.420627668	5.14667E-08	up	67	73	99	0	0	2
LRRC39	-2.590172115	0.017652004	down	22	24	34	16	259	42
LRRC3B	-6.310167259	0.000526818	down	0	0	0	16	11	4
LRRC3C	-3.105148858	6.7405E-05	down	3	7	3	22	36	19
LRRC4	-7.27423247	4.33629E-10	down	0	0	2	58	83	77
LRRC40	0.743724689	5.98203E-05	up	716	859	647	294	299	345
LRRC42	1.72433816	1.11894E-23	up	1085	1027	1020	259	209	207
LRRC45	1.426966286	4.40729E-09	up	368	419	352	133	76	94
LRRC49	-2.376578784	3.90804E-20	down	52	78	51	239	215	209
LRRC4B	-9.060363149	1.50105E-11	down	1	0	0	224	77	116
LRRC4C	-9.582081267	3.59285E-12	down	0	0	0	174	60	73
LRRC56	4.129225366	0.002493305	up	14	23	13	0	1	1
LRRC58	1.553702874	1.04478E-20	up	3619	4250	3693	848	844	1102
LRRC59	2.560751065	4.04298E-29	up	7403	8214	7272	873	1056	790
LRRC6	-6.200653397	1.07247E-08	down	0	3	0	38	64	50
LRRC61	3.711530109	7.99997E-31	up	405	495	420	28	28	15
LRRC63	-7.0584386	3.93379E-06	down	0	0	0	16	11	26
LRRC66	-5.303775187	8.93793E-09	down	0	2	2	39	27	47
LRRC70	-5.895187338	3.33832E-18	down	3	2	2	94	112	87
LRRC73	1.676922292	0.034806787	up	21	36	26	7	9	2
LRRC8C	-3.389588456	1.22832E-16	down	73	70	88	376	769	539
LRRC8D	2.03273945	1.59755E-26	up	1097	1137	991	164	176	219
LRRC8E	5.007669755	0.000172658	up	200	209	185	0	12	0
LRRCC1	-1.868689035	1.36033E-05	down	151	139	135	438	170	525
LRRIQ1	3.478818217	0.013910217	up	49	40	31	8	0	0
LRRK2	-4.39424736	2.41089E-14	down	47	55	67	829	280	1502
LRRN1	-9.797275895	3.8046E-08	down	0	0	0	144	6	216
LRRN2	3.407198325	1.58507E-18	up	233	327	322	28	19	12
LRRN3	-8.359538301	1.15583E-10	down	0	0	1	99	85	66
LRRN4	-4.768150454	0.031738477	down	1	0	3	0	67	5
LRRN4CL	-7.11694411	7.96811E-22	down	3	2	3	131	334	309
LRRTM1	-6.565797222	7.08091E-05	down	0	0	0	17	13	7
LRRTM2	-6.446653513	0.000303612	down	0	0	0	9	5	21
LRRTM4	-6.092004074	0.009155866	down	0	0	0	5	19	1
LRSAM1	0.840823918	5.00104E-05	up	543	695	680	234	253	270
LRTOMT	-0.765686931	0.04464312	down	130	75	92	151	104	108
LRWD1	1.885815719	1.94069E-18	up	656	653	703	141	134	111
LSAMP	-10.87544553	1.08366E-17	down	0	0	0	342	191	211
LSG1	0.587438318	1.40413E-06	up	958	1032	999	507	430	478
LSM11	1.500169398	4.94493E-09	up	596	590	558	196	100	148
LSM12	1.459307747	2.94513E-11	up	321	360	276	80	83	83
LSM14A	-0.91030046	0.001323405	down	78	119	80	145	106	118
LSM14B	0.757497073	2.92186E-07	up	1353	1348	1270	573	551	541
LSM4	1.769404099	4.26193E-12	up	2989	3097	2761	705	666	458
LSM5	0.930664411	0.000614572	up	1557	1708	1392	768	532	436
LSM6	-0.790931333	0.000669283	down	255	239	246	385	231	304
LSMEM1	-0.992830647	0.021678671	down	59	58	72	98	50	125
LSP1	-7.935467869	2.86336E-41	down	1	5	4	816	483	446
LSR	-3.128173697	3.47831E-17	down	14	32	21	148	109	159
LTBP1	-3.526899323	4.85322E-09	down	1816	1982	1749	20908	3557	22734
LTBP2	-8.153632146	4.51672E-52	down	88	82	74	23308	4915	22998
LTBP3	-3.375067889	3.06357E-14	down	579	631	572	5736	1727	6068
LTBP4	-3.26561618	1.56722E-13	down	1073	1148	1159	10205	3054	10563
LTC4S	-6.182389397	3.75596E-12	down	0	2	5	211	84	73
LUC7L	-0.9375759	0.000318861	down	591	660	583	1036	537	960
LUC7L3	-0.817169236	0.045933508	down	4410	5017	4396	8730	2784	6233
LUM	-8.755394885	4.45974E-47	down	23	28	17	3603	12066	4104
LURAP1	-8.669270801	4.35E-10	down	0	0	1	179	47	94
LURAP1L	-2.630749558	4.08196E-16	down	65	60	83	322	209	400
LUZP1	0.859496127	7.39424E-05	up	2118	2397	2066	738	936	874
LUZP2	2.415728068	0.021162467	up	139	149	166	5	47	4
LVRN	-7.042363976	1.01916E-09	down	5	0	3	33	318	396
LXN	-11.27216566	2.88252E-18	down	0	0	0	208	335	419
LY6D	6.11500767	6.26685E-05	up	16	20	18	0	0	0
LY6E	2.566495935	0.008304974	up	24	225	37	12	10	11
LY6K	5.756132326	2.72099E-41	up	2445	2626	2277	23	17	58
LY75	-9.111251621	8.85805E-12	down	0	0	0	101	43	77
LY86	-7.872608914	1.9551E-15	down	0	3	0	235	148	114
LY9	-3.211333978	0.00487302	down	2	2	3	25	3	20
LY96	-6.922840692	1.82902E-36	down	5	2	2	295	232	251
LYAR	1.516189961	4.39842E-11	up	867	938	833	262	219	172
LYG1	1.251417231	0.030554674	up	53	61	47	8	22	17
LYL1	-10.23647473	7.77063E-16	down	0	0	0	210	126	141
LYN	0.693401888	9.61016E-06	up	789	926	833	437	330	354
LYNX1	-7.520339101	2.91951E-48	down	5	4	12	746	1142	817
LYPD3	5.460750004	5.77333E-40	up	965	902	883	27	9	9
LYPD6	2.612034914	1.67146E-08	up	224	218	224	15	19	44
LYPLA1	1.476408234	1.3815E-06	up	2325	2472	2229	464	750	545
LYPLA2	1.554927423	4.10735E-20	up	1366	1530	1300	379	332	300
LYPLAL1	-1.448201337	8.90578E-10	down	245	309	271	562	576	445
LYRM1	-0.895000951	9.13945E-05	down	355	490	398	470	532	628
LYRM2	-0.713427888	0.002001697	down	399	514	378	503	535	448
LYRM5	-2.177727761	4.63264E-23	down	160	170	135	504	528	450
LYRM9	-2.169762768	8.78379E-11	down	61	51	52	240	119	174
LYST	-1.67194694	4.81234E-06	down	549	557	518	980	808	1938
LYVE1	-12.98622259	3.26679E-20	down	0	0	0	509	549	2185
LYZ	-9.679368075	1.64034E-27	down	3	1	1	1798	381	843
LZTR1	-1.05899293	8.54576E-11	down	504	550	526	916	613	828
LZTS1	-3.899636739	0.001409282	down	10	0	3	20	79	36

LZTS3	-0.985057306	0.017238812	down	170	139	141	190	128	327
M1AP	1.815942523	1.40523E-06	up	115	108	114	28	13	28
M6PR	1.445083505	2.04164E-09	up	3427	3901	3252	715	939	1083
MAATS1	-8.417902105	1.37091E-09	down	1	0	0	131	36	102
MAB21L1	-9.065893191	1.61226E-07	down	0	0	0	15	29	171
MAB21L2	-10.00987448	0.000482045	down	0	0	0	21	5	396
MAB21L3	5.85497358	5.13169E-12	up	113	97	114	0	1	3
MACC1	-6.61517352	1.9241E-05	down	1	0	0	46	18	12
MACF1	-1.041924965	1.1177E-05	down	5305	5741	5569	7671	6015	10854
MACROD1	0.678340836	0.026891712	up	457	536	440	276	207	151
MACROD2	1.49539593	4.26462E-05	up	161	199	145	31	36	60
MAD1L1	1.223371275	8.76838E-10	up	512	566	582	160	143	204
MAD2L1	4.145314219	3.29527E-20	up	1488	1590	1476	97	59	27
MAD2L2	2.190223607	3.1387E-18	up	1114	1171	1026	199	184	129
MADD	0.961623434	3.42834E-12	up	1618	1650	1556	565	559	634
MAEA	-0.603367179	5.31082E-05	down	675	634	600	751	635	678
MAEL	-5.894254904	0.010680741	down	0	0	0	1	15	6
MAF	-9.479108783	1.42667E-52	down	1	3	3	1350	820	1421
MAF1	-1.092351856	3.87151E-10	down	1106	1238	1054	1999	1289	1903
MAFA	5.248186265	9.72081E-19	up	150	199	182	7	1	2
MAFB	-3.038158804	1.39666E-61	down	140	162	152	926	700	1040
MAFF	-1.313451128	0.000252362	down	314	415	344	771	336	823
MAFG	1.128443807	2.25849E-13	up	1854	1913	1840	733	508	592
MAFK	1.348935813	0.000411758	up	2967	3295	3420	960	464	1339
MAGEA1	12.8717528	4.3202E-26	up	1891	2099	1845	0	0	0
MAGEA10	5.977116161	0.000268952	up	22	17	10	0	0	0
MAGEA11	7.753159866	4.56998E-09	up	57	61	50	0	0	0
MAGEA12	10.995933	4.5787E-19	up	554	549	485	0	0	0
MAGEA3	12.79362281	8.94395E-26	up	1780	2007	1742	0	0	0
MAGEA6	12.50490954	1.6944E-24	up	1476	1720	1337	0	0	0
MAGEB6	5.662417195	0.000980485	up	12	9	18	0	0	0
MAGEC1	7.205262265	1.74471E-07	up	31	43	41	0	0	0
MAGEC3	-3.332788535	0.047109985	down	4	1	3	18	0	43
MAGED1	-9.402859617	6.58451E-64	down	1	4	3	1301	1279	1246
MAGEE1	-10.02276856	1.16206E-13	down	0	0	0	189	62	168
MAGEF1	-0.744096902	0.025241139	down	416	518	400	775	423	398
MAGEH1	-8.890595258	1.32743E-30	down	2	0	3	825	350	551
MAGEL2	-4.424573734	0.022024707	down	1	0	0	6	8	2
MAGI1	-1.114839816	1.18273E-07	down	458	430	441	652	735	645
MAGI2	-6.968813571	9.78349E-29	down	6	3	3	529	180	389
MAGOHB	1.100961259	0.000709658	up	633	760	567	315	176	161
MAGT1	0.735360065	4.7174E-06	up	3247	3480	3116	1563	1074	1598
MAK16	0.858718863	0.000828782	up	916	1048	944	462	385	287
MAL	-4.02955595	3.96984E-07	down	3	1	4	39	19	37
MAL2	5.092898623	2.95874E-09	up	2473	2646	2284	29	108	7
MALRD1	-4.576602244	0.000137437	down	2	1	0	11	21	18
MALT1	0.840742196	5.66757E-05	up	1239	1208	1131	405	453	559
MAMDC2	-3.280498517	1.27033E-06	down	75	70	39	319	135	852
MAML2	-1.435683324	1.35279E-05	down	388	446	394	795	470	1133
MAML3	-0.608064719	0.017089419	down	174	204	219	188	202	256
MAMSTR	-1.416410264	0.016143549	down	13	22	14	47	25	21
MAN1A1	-3.038483086	3.94352E-09	down	425	420	453	1150	1960	4520
MAN1B1	1.046111563	1.1163E-12	up	2016	2300	2221	877	668	709
MAN1C1	-5.943540397	6.22886E-85	down	12	11	14	594	450	583
MAN2A1	0.784943975	0.037507865	up	2388	2655	2571	1025	583	1593
MAN2A2	-0.858746641	0.00149288	down	824	994	896	956	963	1586
MAN2B1	-1.305757803	3.7015E-18	down	562	647	613	1095	883	1236
MAN2B2	-2.449303372	4.62887E-13	down	383	473	377	1916	857	2101
MAN2C1	-1.649994916	2.65083E-11	down	450	530	553	1180	805	1474
MANBAL	-0.674988592	0.03064188	down	836	937	937	1435	634	1063
MANEAL	3.493884403	6.32982E-55	up	644	714	613	50	29	46
MANF	1.150300106	0.002828298	up	1851	2028	1865	1006	413	450
MANSC1	-2.501573391	0.008575313	down	7	7	2	40	8	18
MAOA	-8.579795209	2.00849E-43	down	7	19	7	1692	1854	5410
MAP10	-8.522345298	8.02942E-09	down	0	0	0	34	76	29
MAP1A	-1.325250514	0.000283892	down	448	548	491	632	1168	780
MAP1B	-4.796315251	3.41319E-17	down	436	440	391	14076	2277	9651
MAP1LC3A	-3.641773404	5.40725E-46	down	37	48	35	357	367	331
MAP1LC3C	-7.32394952	2.99579E-06	down	0	1	0	12	37	73
MAP1S	0.978347172	1.26829E-10	up	658	643	666	256	210	246
MAP2	-1.607740231	0.000563967	down	363	363	318	958	302	1068
MAP2K1	0.860967433	1.49999E-11	up	1748	1871	1851	730	688	719
MAP2K2	1.381625392	1.56148E-10	up	2700	2956	2905	865	821	634
MAP2K3	1.635842237	2.3991E-12	up	1719	1863	1858	483	431	324
MAP3K1	1.22533865	1.04789E-12	up	1178	1265	1155	330	368	390
MAP3K10	0.623930758	0.00103332	up	378	391	354	188	135	198
MAP3K12	-3.65651453	4.94201E-57	down	46	65	50	519	379	550
MAP3K14	1.321305781	5.32734E-19	up	629	663	616	181	164	198
MAP3K3	-2.339990777	4.65524E-27	down	372	322	301	1121	998	1482
MAP3K5	-1.629175878	8.22825E-08	down	563	567	606	1455	759	1679
MAP3K6	-0.92417887	0.000759905	down	574	630	592	635	704	1085
MAP3K7CL	-2.488734383	1.75321E-10	down	119	103	94	649	345	279
MAP3K8	-1.553027904	3.36091E-24	down	386	393	349	830	643	891
MAP3K9	5.552321768	4.19117E-30	up	1070	1217	1121	8	29	13
MAP4K2	1.388459545	5.57623E-13	up	561	681	614	196	126	185
MAP4K3	-0.701854077	8.05295E-08	down	744	856	751	907	796	1016
MAP4K4	-0.819052104	0.002919974	down	1802	1911	1734	1807	2677	2238
MAP4K5	-0.728701477	8.46638E-05	down	903	1041	898	1231	833	1309
MAP6	-10.70541454	1.04144E-17	down	0	0	0	230	169	261
MAP6D1	2.236655765	5.24196E-05	up	109	89	74	21	5	16
MAP7	4.387345576	4.40509E-63	up	2857	3163	2927	88	74	144
MAP9	-11.86822608	4.38953E-21	down	0	0	0	589	313	588
MAPK10	-3.53878937	3.79232E-26	down	77	85	82	626	846	505
MAPK11	-1.481977492	0.000858257	down	233	260	272	331	733	409
MAPK6	1.869722546	1.19444E-23	up	2386	2445	2367	559	443	398
MAPK8IP1	-1.815836347	1.73764E-07	down	42	44	52	94	131	117
MAPK9	0.762967301	0.000104791	up	1802	2041	1827	678	763	923
MAPRE1	0.964387765	1.30769E-07	up	5069	5477	4844	2075	1377	2206
MAPRE2	-1.46545804	2.7841E-21	down	679	659	666	1268	1165	1511
MAPRE3	-0.87166646	0.002091614	down	246	250	274	313	390	287
MARC2	-0.763234183	0.002307118	down	525	563	534	617	749	566

MARCH1	-5.299782992	2.11472E-18	down	8	7	8	201	97	363
MARCH2	-3.557541974	5.22029E-41	down	71	71	89	553	684	685
MARCH3	-2.672870483	0.000765262	down	28	42	32	32	243	166
MARCH6	1.055244387	9.58153E-09	up	6456	6802	6334	2616	1630	2528
MARCO	-6.892928203	4.67225E-22	down	7	4	2	191	270	646
MARK1	-0.885052191	4.83134E-07	down	240	308	269	348	326	396
MARK2	0.749457447	0.000543409	up	751	827	770	285	354	345
MARS2	1.228661612	0.024143226	up	269	242	260	46	124	56
MARVELD1	-1.062058458	0.014069005	down	953	1108	885	1997	638	1848
MASP1	-5.614202033	2.49897E-10	down	5	6	17	107	635	184
MAST1	5.879083915	1.008E-06	up	42	64	62	0	2	0
MAST3	-0.989616964	0.02904405	down	262	306	268	601	176	434
MAST4	-4.586925069	5.91087E-79	down	48	82	59	1011	1069	1115
MASTL	2.446806101	1.63105E-33	up	1548	1655	1563	197	162	266
MATK	3.428136875	1.46788E-26	up	575	579	591	58	23	36
MATN2	-3.517829535	2.32615E-21	down	230	271	215	2295	2308	1128
MATN3	1.0260732	0.015235713	up	107	105	104	36	21	55
MAU2	-0.789180252	0.000424205	down	597	535	590	763	523	856
MAVS	-0.826034461	0.007555162	down	1278	1472	1233	1174	1604	2209
MAX	-0.606468766	0.002407479	down	1019	905	838	1157	769	1093
MAZ	1.232422762	6.78172E-13	up	4159	4412	4322	1412	1321	1150
MB21D1	2.226451908	3.85523E-19	up	400	376	307	51	55	58
MBD1	-0.961547835	1.71677E-12	down	642	729	680	1034	774	1044
MBD3	-1.316323961	4.71207E-18	down	481	532	554	972	893	904
MBD4	1.209027901	5.39397E-27	up	1671	1787	1699	540	476	571
MBD5	-1.395738606	2.2222E-08	down	174	151	154	301	227	377
MBD6	-1.051418743	0.00022223	down	331	260	340	557	319	521
MBIP	-1.024817176	0.000372482	down	308	397	323	609	310	587
MBLAC2	-0.624174028	0.007213828	down	121	130	142	152	141	137
MBNL1	-1.480719305	0.000131671	down	3641	3823	3275	8767	3380	9678
MBNL2	-0.754813132	0.002082262	down	1573	1794	1559	1864	1453	2636
MBNL3	3.027825642	1.12578E-27	up	1812	1849	1606	109	155	192
MBOAT7	3.30878649	0.016143549	up	19	17	18	4	0	0
MBP	-1.883133971	1.61552E-12	down	213	210	236	592	402	758
MBTPS1	-0.8393302	1.03405E-11	down	1541	1701	1487	2062	1698	2269
MBTPS2	1.614519093	4.61071E-17	up	1676	1890	1533	344	369	466
MC1R	1.196849237	0.000424766	up	188	255	203	92	60	48
MCAM	-1.772382393	5.13536E-07	down	4479	5238	4781	12429	6301	17167
MCC	-1.822282194	0.000121802	down	238	193	213	289	759	530
MCCC1	-1.34296352	0.024826447	down	487	565	518	441	1602	654
MCEE	-0.733235478	0.000802736	down	144	164	161	220	166	169
MCF2	-5.42031087	1.89705E-08	down	4	1	2	74	21	127
MCF2L	1.258735141	0.002421677	up	1780	1837	1774	320	732	507
MCF2L2	1.771401521	0.002959339	up	53	48	34	13	10	5
MCHR1	-4.241615262	0.025201267	down	2	0	0	2	16	8
MCIDAS	8.154741237	6.19565E-10	up	88	69	64	0	0	0
MCM10	5.294557883	1.1519E-79	up	1018	1215	1040	24	22	13
MCM2	5.213612533	7.27025E-40	up	5771	6347	5769	158	125	57
MCM3	2.321393648	3.12352E-67	up	4682	5213	4482	789	550	715
MCM4	4.061432949	1.56082E-71	up	5816	6035	5626	281	266	192
MCM5	2.330727451	6.83132E-74	up	1698	1794	1607	252	206	264
MCM6	2.347456428	1.70209E-57	up	2689	3077	2695	453	367	360
MCM7	2.677884357	1.604E-144	up	6901	7467	6823	855	659	848
MCM8	1.887434754	2.26382E-36	up	947	1016	934	189	184	182
MCM9	-0.739508325	0.012008254	down	100	147	132	127	144	176
MCMDC2	-1.78779039	0.023551071	down	4	8	8	16	22	10
MCOLN2	-5.214494728	0.000258672	down	2	0	1	18	6	59
MCOLN3	-7.572979986	1.31193E-05	down	0	0	2	158	3	128
MCRIP1	-4.301811774	2.63123E-37	down	69	47	66	1212	628	759
MCRS1	0.628506139	0.00012182	up	1290	1519	1218	663	606	572
MCTP2	4.982243148	2.40154E-41	up	1123	1220	1156	16	20	43
MCTS1	0.64652042	1.85438E-06	up	1441	1552	1325	696	627	632
MCU	2.265450782	1.66018E-17	up	3580	3749	3224	542	337	702
MCUR1	0.69390828	0.002037618	up	847	1059	849	350	432	414
MDFI	-3.108825192	0.000262849	down	21	8	29	84	216	44
MDFIC	-2.571635307	9.91711E-10	down	258	259	210	666	1459	852
MDGA1	-0.904027703	0.000145265	down	98	100	92	138	101	149
MDH1B	-4.831142647	7.69211E-05	down	0	0	3	23	21	17
MDH2	1.172882098	0.007470733	up	5181	5654	5144	1191	2481	1198
MDK	-3.276243186	3.32966E-12	down	19	16	25	141	174	93
MDP1	-2.329586291	0.021349399	down	6	3	4	28	14	5
ME1	-1.913191171	0.030506998	down	1179	1312	1240	752	7170	1335
MECOM	-0.709566892	5.8777E-08	down	983	1084	1039	1210	1046	1362
MECP2	-0.748653152	0.00568112	down	1028	1130	959	1135	906	1711
MED1	0.599798601	0.000650899	up	1738	1948	1696	752	829	929
MED10	1.294462978	2.09253E-13	up	1218	1400	1303	457	343	337
MED13L	-1.048073873	0.000828632	down	1371	1404	1331	1670	1455	2969
MED14	2.433059523	9.88912E-43	up	4003	4367	3898	602	395	632
MED14OS	-2.365674645	0.003287632	down	6	2	8	24	21	14
MED16	-1.833790606	3.93504E-18	down	120	106	93	268	253	287
MED17	1.835343174	2.72986E-46	up	1647	1814	1594	372	311	323
MED20	1.202519322	1.66288E-11	up	723	827	690	277	205	210
MED26	0.821283027	8.48951E-05	up	252	280	229	109	82	116
MED27	0.749335906	0.00161582	up	617	723	553	306	186	315
MED30	-0.906251442	0.020199844	down	149	172	151	315	118	208
MED31	1.133513218	0.000591384	up	335	357	297	145	102	73
MED4	-0.726291219	9.70417E-08	down	788	771	738	1023	773	917
MED8	0.871697796	3.03662E-06	up	1154	1187	1129	522	436	388
MEDAG	-11.72466067	2.83282E-21	down	0	2	0	2411	472	2054
MEF2C	-9.086513315	6.52254E-61	down	2	8	12	3955	1566	3161
MEGF10	-7.040088746	6.1836E-05	down	0	0	0	5	25	20
MEGF6	-1.530152727	3.02715E-05	down	1178	1235	1148	2928	1226	3329
MEGF8	-2.185463517	4.22787E-10	down	264	331	285	622	1167	994
MEI1	-4.843530331	0.007423848	down	1	1	0	29	11	1
MEI4	-3.936355347	0.017092328	down	2	0	0	10	7	5
MEIKIN	-5.86325968	0.018742429	down	0	0	0	21	1	2
MEIOB	7.757692175	7.12824E-09	up	47	55	66	0	0	0
MEIS1	-2.19157956	2.26284E-05	down	198	167	171	690	210	900
MEIS2	-1.97265125	8.15885E-29	down	222	239	189	707	532	576
MEIS3	0.600548577	0.011045053	up	267	300	287	153	134	112
MELK	5.692468338	1.53615E-07	up	1557	1558	1357	51	12	0

MELTF	1.294211158	0.02288916	up	402	399	341	62	63	211
MEMO1	0.630644567	0.01831541	up	548	583	542	238	299	221
MEN1	1.660429116	2.98256E-25	up	984	974	961	215	218	222
MEOX1	-2.067686733	1.44437E-07	down	101	125	109	372	408	200
MEOX2	-11.69306365	1.13733E-19	down	0	0	0	291	529	455
MERTK	-1.863740926	0.000182972	down	186	187	236	525	224	874
MESDC1	-0.949555692	0.001686703	down	217	215	193	303	329	216
MESP2	2.046757765	0.007572946	up	25	28	18	3	6	3
MET	3.558797823	1.99646E-23	up	8315	9155	8307	308	504	733
METAP1	0.645975928	0.014075135	up	2075	2126	1976	1158	601	1092
METAP2	0.619701449	0.000196176	up	3650	3960	3469	2033	1312	1820
METRNL	0.834553683	0.031730712	up	196	245	253	127	50	105
METTL1	1.118318622	5.52749E-05	up	237	241	236	81	88	63
METTL10	-0.871167033	0.007986224	down	230	243	195	420	228	227
METTL13	0.725056162	0.000894374	up	935	962	962	418	446	356
METTL14	-0.901267526	0.000411423	down	386	376	336	574	326	578
METTL15	0.722971988	0.000167467	up	526	619	517	226	248	236
METTL16	0.59892687	0.002069887	up	1084	1344	1129	619	414	647
METTL21B	-0.814057259	0.003711565	down	159	153	187	269	154	209
METTL24	-8.872793533	4.87767E-10	down	0	0	0	73	25	91
METTL25	-1.848748591	2.46841E-05	down	71	52	34	171	83	155
METTL2A	1.392187376	2.36342E-09	up	573	561	486	129	160	146
METTL2B	1.722258552	2.81045E-18	up	768	847	843	166	189	171
METTL3	-0.671314689	0.000106863	down	510	574	477	710	483	581
METTL6	0.918882685	0.003217721	up	559	618	496	148	229	244
METTL7A	-6.115490891	5.76331E-50	down	65	84	120	2729	4535	5923
METTL9	1.010099032	2.18729E-06	up	3427	3992	3490	1239	1424	1139
MEX3A	3.976027928	8.82983E-31	up	339	287	327	13	17	13
MEX3D	1.43597492	9.10238E-15	up	772	729	767	220	155	226
MFAP1	0.763882573	1.9468E-05	up	1507	1666	1466	789	493	675
MFAP2	-1.90511872	2.63662E-05	down	120	119	118	545	174	255
MFAP3L	-1.972720801	0.000377833	down	132	129	104	363	119	567
MFAP4	-15.30756248	3.309E-28	down	0	0	1	15885	2500	13849
MFAP5	-5.295657972	8.94524E-15	down	160	155	156	1556	2094	9786
MFGE8	-5.735922766	3.86787E-23	down	382	495	464	21907	4319	26398
MFHAS1	1.309352215	5.564E-06	up	1596	1633	1521	361	365	643
MFNG	-2.667544578	3.17355E-13	down	66	52	58	185	303	299
MFSD1	-0.959540506	2.08298E-11	down	999	1041	885	1352	1159	1540
MFSD12	4.703700629	2.6638E-261	up	8510	9621	8916	267	239	230
MFSD2A	1.99516704	0.036213954	up	66	75	100	33	9	2
MFSD4A	-2.59625854	4.10117E-07	down	11	18	13	85	55	40
MFSD4B	0.642369918	0.048821131	up	189	177	170	81	55	111
MFSD6	-1.458729502	1.24318E-11	down	198	226	171	345	385	424
MFSD7	-4.358551846	4.14822E-28	down	12	11	8	140	162	146
MFSD8	-1.29161111	3.88132E-10	down	186	182	165	326	241	367
MGA	1.17960735	1.19459E-09	up	2426	2758	2350	787	604	987
MGARP	-5.701405626	1.33456E-08	down	0	3	3	145	34	48
MGAT1	-1.390961464	5.21045E-10	down	1255	1356	1302	1989	2593	2644
MGAT2	0.751086879	0.000726427	up	965	1062	943	394	466	380
MGAT3	-6.632664467	1.29564E-12	down	3	1	8	120	530	163
MGAT4A	-4.337577274	5.80508E-67	down	40	51	31	623	577	540
MGAT4B	1.281990706	0.000114063	up	2298	2437	2301	590	893	529
MGAT4C	-6.763310244	1.18616E-06	down	0	1	0	23	32	27
MGAT5B	4.348773734	5.21843E-21	up	466	514	444	6	20	23
MGME1	0.809759524	0.001292835	up	633	630	639	214	287	263
MGMT	-2.280062095	3.46316E-11	down	97	141	118	608	307	323
MGP	-15.40281017	2.88477E-75	down	0	1	5	102816	26013	62695
MGRN1	0.600892065	0.003661719	up	1163	1242	1136	517	449	701
MGST1	-2.314183893	0.012153136	down	1307	1481	1311	687	10272	2470
MGST2	-1.028446644	5.32075E-08	down	464	389	384	628	578	588
MGST3	-1.217556868	2.89713E-09	down	1591	1753	1594	3086	2789	2238
MIA2	4.456501885	0.033191629	up	6	5	6	0	0	0
MIB2	-1.195931296	4.05304E-06	down	261	217	286	473	307	483
MICAL1	-1.940696709	1.94137E-08	down	286	329	322	1156	459	998
MICAL2	-2.513134214	5.99177E-10	down	1035	1163	1020	4019	2223	7080
MICU1	1.315536553	1.85008E-10	up	2861	2982	2696	724	892	797
MICU2	-0.874000323	0.000442763	down	615	664	535	745	896	689
MICU3	-11.39791472	9.64816E-19	down	0	0	0	305	235	532
MID1	0.915967209	1.24117E-07	up	1896	2138	1958	933	598	741
MID1IP1	1.004364455	5.53462E-19	up	1750	1898	1743	646	602	659
MID2	-0.95989598	0.000118391	down	342	373	346	392	522	539
MIF4GD	-0.934341898	0.028513803	down	197	267	204	431	146	349
MIIP	0.909621312	2.17341E-05	up	438	481	470	219	155	153
MILR1	-4.459684529	5.91516E-19	down	5	12	9	183	125	97
MINK1	0.695191248	0.000411027	up	1996	2033	2074	786	822	1072
MINPP1	1.091099736	2.66176E-11	up	599	601	551	222	177	186
MIR1-1HG	-5.621191002	0.021907818	down	0	0	0	3	14	1
MIS18A	1.680895537	9.51638E-09	up	429	485	419	124	101	69
MIS18BP1	0.959569131	0.001184638	up	992	1139	1041	276	380	496
MISP	12.6430046	3.43768E-25	up	1605	1798	1577	0	0	0
MITD1	0.921542214	0.00135099	up	788	935	779	443	213	294
MITF	1.144935738	8.89361E-13	up	1557	1786	1541	485	524	551
MIXL1	-5.588606729	0.00241894	down	0	0	0	7	5	7
MKI67	5.901957061	3.82181E-17	up	7326	8320	7488	169	90	16
MKKS	-0.848637049	1.69875E-08	down	544	654	567	837	709	707
MKL1	-0.678420198	0.005536072	down	415	432	399	463	349	618
MKL2	-1.541242755	3.75944E-06	down	944	963	922	1399	1581	2899
MKLN1	0.684485091	0.018107748	up	4004	4436	4000	1563	1369	2610
MKNK1	-0.900817835	0.000399433	down	598	642	644	1088	562	888
MKRN3	4.43720919	9.95266E-05	up	152	187	160	16	1	0
MKX	-7.789519447	9.15443E-10	down	1	0	1	115	36	175
MLF1	1.888451021	0.001554924	up	1181	1442	1155	164	416	110
MLF2	1.134864666	1.44858E-06	up	4293	4532	3976	1270	1587	1229
MLIP	-7.514860779	2.34184E-06	down	4	1	1	4	662	49
MLKL	0.986803015	0.002388033	up	753	806	711	406	208	208
MLLT1	0.595458431	6.37387E-05	up	1794	1811	1839	820	826	911
MLLT10	-0.673361413	0.000130814	down	691	759	641	905	603	879
MLLT11	-1.46954238	0.002596048	down	107	128	138	204	360	148
MLLT3	1.550961109	4.27943E-19	up	643	729	718	160	154	193
MLLT4	-0.831503707	1.77375E-05	down	998	1039	969	1111	1308	1357
MLPH	-10.61653558	3.58156E-16	down	0	0	0	172	140	311

MLYCD	-2.588198239	0.000310013	down	105	140	70	217	837	206
MMAA	-2.501753643	1.25701E-09	down	61	48	43	174	268	158
MMACHC	2.191673667	2.73536E-05	up	339	334	303	21	70	57
MMD	-2.955601813	0.000137421	down	113	121	109	218	1275	275
MME	-11.82149129	2.74564E-12	down	2	1	1	170	8327	938
MMP10	3.996929036	0.02211265	up	9	6	9	0	0	1
MMP13	3.885274454	0.000138643	up	42	21	40	3	2	0
MMP14	-4.784648735	6.53629E-91	down	163	142	196	3224	2690	4000
MMP16	-10.55040938	1.6683E-15	down	0	0	0	261	95	247
MMP17	3.23621978	2.77665E-06	up	680	904	745	17	107	42
MMP19	-5.206758758	9.65015E-51	down	8	19	22	447	411	423
MMP2	-6.208153043	8.4728E-197	down	162	135	116	6783	6811	8095
MMP23B	-6.240587013	8.64146E-13	down	1	2	1	100	51	66
MMP24	3.865930796	3.17296E-37	up	372	503	468	22	25	18
MMP27	-7.015346423	0.027559106	down	0	0	0	1	0	52
MMP8	-5.097284631	0.022849638	down	0	0	0	4	7	2
MMRN1	-10.80117812	1.80348E-20	down	0	2	0	496	708	1319
MMRN2	-2.962956652	1.58402E-24	down	335	333	376	1593	2348	1758
MMS19	0.671967086	1.2816E-06	up	1269	1431	1159	629	531	558
MMS22L	2.182960204	6.41233E-23	up	635	578	562	82	96	99
MND1	4.014282241	2.5756E-07	up	303	272	257	30	3	5
MNDA	-10.51001203	1.42129E-12	down	0	0	0	359	46	191
MNT	1.263260786	8.1972E-07	up	900	1064	947	266	211	391
MNX1	8.769805076	1.5071E-11	up	132	124	84	0	0	0
MOB2	-2.777230759	6.13695E-21	down	152	152	117	885	432	766
MOB3A	-1.157058886	3.99434E-05	down	258	307	238	321	439	499
MOB3C	-1.242248113	1.08549E-05	down	329	301	322	750	398	475
MOCOS	2.625432315	6.60541E-11	up	352	356	376	54	48	22
MOCS1	-12.68911548	1.16533E-20	down	0	0	0	397	1305	796
MOCS2	-0.69365809	3.2198E-05	down	879	1086	926	1328	953	1047
MOCS3	1.568504225	1.17271E-10	up	374	364	341	73	93	91
MOGAT3	7.258069822	1.19617E-07	up	34	41	44	0	0	0
MOGS	1.138899548	1.18175E-07	up	1471	1519	1308	388	437	558
MON2	-0.791965876	0.000246332	down	951	1154	974	1363	906	1552
MORC3	-0.610716773	0.038015089	down	776	820	780	1126	530	969
MORF4L1	-0.589240456	0.033003225	down	5197	5629	4978	7145	3523	6510
MORN2	0.856528702	0.013138135	up	332	449	317	151	170	102
MORN3	-2.527030084	0.02997781	down	6	0	3	14	9	15
MOSPD1	2.094548136	2.85357E-18	up	1134	1213	1151	175	226	174
MOV10	1.014831445	1.64026E-11	up	1402	1544	1330	489	431	586
MOV10L1	-1.352147957	0.03059844	down	36	30	23	92	44	27
MPC1	-3.025168853	9.09003E-11	down	130	177	182	837	1375	528
MPC2	-1.151208484	0.023735515	down	1081	1126	1098	1259	2744	1019
MPDZ	-11.43488885	7.41286E-36	down	1	0	2	1491	1793	2623
MPEG1	-12.65253061	6.85604E-23	down	0	0	0	805	497	1268
MPHOSPH10	0.946456224	3.87609E-07	up	1423	1446	1315	642	400	514
MPHOSPH6	0.849497805	0.002414184	up	459	424	368	182	179	129
MPHOSPH8	-1.266086937	1.55122E-07	down	770	875	742	1431	935	1758
MPHOSPH9	1.277581336	1.10636E-12	up	785	875	709	250	231	210
MPI	0.772858956	5.48026E-05	up	765	773	780	315	339	305
MPL	-5.184623554	0.000167687	down	0	0	2	23	17	12
MPLKIP	0.836778826	0.000566246	up	488	550	501	218	223	166
MPO	-7.0053075	5.8021E-05	down	0	0	0	26	19	5
MPP1	-1.044900004	4.6446E-05	down	380	345	405	591	600	458
MPP3	0.720158879	0.00407059	up	299	387	345	193	117	137
MPP4	-5.225195275	0.032817541	down	0	0	0	11	3	1
MPP5	2.242448739	5.10496E-52	up	3032	3144	2758	521	356	473
MPP6	1.577007736	0.000789003	up	1138	1246	1020	364	104	366
MPPE1	-1.1365739	2.72079E-07	down	271	294	280	480	317	537
MPPED2	-10.67878369	2.06282E-13	down	0	1	0	834	101	371
MPST	-0.694245641	0.000324582	down	589	629	603	664	742	672
MPV17L	1.32905486	0.030547953	up	48	52	37	6	17	15
MPV17L2	1.203219516	8.36973E-07	up	410	477	479	154	150	115
MPZ	-4.379379974	7.30445E-14	down	8	17	9	112	109	283
MPZL2	-1.755474494	2.94691E-05	down	199	253	215	653	636	285
MPZL3	1.628473422	1.91618E-07	up	190	188	152	44	45	32
MR1	-1.338225472	8.59269E-05	down	411	423	427	759	447	1102
MRAP	-11.11384695	3.74E-06	down	0	1	0	18	1400	120
MRAP2	-7.200865648	1.51232E-12	down	16	32	20	3729	84	3587
MRAS	-2.293734304	0.000336116	down	475	478	509	744	3088	1003
MRC1	-9.891669525	1.9982E-27	down	0	1	5	633	993	2480
MRC2	-2.828216347	4.45564E-32	down	520	538	538	3663	2182	2280
MRE11A	1.357097706	1.47925E-06	up	1424	1700	1499	338	351	596
MRFAP1	-0.67330951	0.000761791	down	4628	5081	4498	6818	3997	5446
MRFAP1L1	-1.515384922	3.70323E-10	down	658	770	684	1925	1011	1405
MRGBP	2.40297318	3.49386E-21	up	1354	1485	1263	215	196	136
MRGPRF	-4.380148137	9.01825E-11	down	73	64	56	1505	188	1289
MRI1	1.556510456	3.98198E-13	up	1079	1199	1096	244	223	351
MRM2	1.215014285	1.80563E-16	up	976	1063	887	334	275	286
MRO	-2.082505293	5.61636E-08	down	21	19	25	82	56	59
MROH6	3.908023589	0.000569719	up	22	51	13	1	1	2
MROH8	-4.985438873	5.31878E-05	down	0	6	0	47	55	26
MRPL1	-1.807034477	6.27905E-08	down	162	152	135	482	380	251
MRPL11	0.967468298	5.95892E-06	up	1445	1566	1465	706	459	470
MRPL12	1.351917364	0.005526702	up	1872	2084	1843	446	819	293
MRPL13	0.743718745	0.000720032	up	1281	1306	1243	657	525	442
MRPL14	0.996018248	0.0007908	up	1119	1207	1127	514	426	284
MRPL17	1.458595248	1.01807E-08	up	1692	1776	1843	586	440	348
MRPL2	0.594718635	0.01092043	up	672	628	626	266	328	306
MRPL22	0.76548187	0.000673864	up	821	919	758	349	382	302
MRPL23	-2.102369301	1.25354E-08	down	38	44	26	146	92	92
MRPL28	1.260487878	1.9993E-10	up	1832	1921	1649	652	497	453
MRPL3	1.700502251	3.34911E-23	up	4741	5074	4278	1115	1039	910
MRPL30	0.934811435	1.7105E-05	up	1939	2131	1789	813	756	593
MRPL36	1.566139387	5.08569E-12	up	948	962	938	290	210	185
MRPL37	1.307200762	0.000234344	up	2530	2793	2399	674	976	515
MRPL39	0.637820997	0.026611045	up	772	919	773	365	444	298
MRPL42	0.713225846	0.000495725	up	1225	1413	1158	640	537	461
MRPL45	0.601603179	0.00316095	up	438	438	441	194	215	205
MRPL48	0.774286129	0.002001909	up	519	583	498	299	176	194
MRPL51	0.682520139	0.00618249	up	2997	3131	2946	1759	1215	1054

MRPL53	-0.722609471	0.025740137	down	358	433	352	528	491	306
MRPL54	-0.725411198	0.01550223	down	416	415	388	609	478	342
MRPL57	-1.064910907	0.004142801	down	293	349	330	689	455	301
MRPL9	0.707204618	8.00065E-05	up	1259	1437	1246	711	468	545
MRPS16	0.856470633	0.005632416	up	2041	2070	2053	724	1009	644
MRPS17	1.266416668	1.34106E-07	up	562	592	517	190	171	130
MRPS2	1.389691783	5.28972E-10	up	1346	1512	1344	382	424	321
MRPS22	0.673066281	0.0125183	up	1191	1240	1075	491	612	437
MRPS25	0.677028705	0.002331126	up	1841	2140	1773	751	939	835
MRPS27	0.803180508	1.3202E-06	up	2195	2306	2255	932	947	858
MRPS30	2.319867729	4.84317E-26	up	1874	1951	1708	269	291	220
MRPS31	-0.73745804	0.014692745	down	238	323	233	413	284	241
MRPS34	1.72651686	1.91339E-13	up	2460	2742	2403	539	621	452
MRPS35	0.713746863	0.000473141	up	1640	1775	1589	705	788	654
MRPS5	1.530221525	5.30138E-15	up	2409	2661	2335	601	660	543
MRPS9	0.869694402	0.000589249	up	1045	1242	1047	478	470	335
MRRF	1.227097706	0.000321711	up	1074	1277	1112	246	444	338
MRTO4	1.976953134	2.8838E-17	up	1636	1676	1493	313	317	230
MRVI1	-12.12402749	2.07145E-36	down	3	1	1	7286	1134	8161
MS4A1	-7.031375734	3.43684E-06	down	0	0	0	17	11	24
MS4A14	-9.815964473	6.64707E-11	down	0	0	0	228	31	109
MS4A2	-8.487191551	3.57869E-07	down	0	0	0	101	10	36
MS4A4A	-12.18654712	2.56988E-19	down	0	0	0	338	405	1106
MS4A4E	-10.53783955	6.67734E-10	down	0	0	0	120	19	469
MS4A6A	-11.88769149	5.02056E-25	down	1	1	0	1662	929	2899
MS4A7	-10.01369182	2.06372E-28	down	1	0	2	754	522	956
MSANTD3	1.223864642	2.4363E-13	up	443	549	500	161	134	159
MSANTD4	0.662921726	0.001745117	up	857	932	805	491	287	397
MSC	-11.48369669	2.35926E-20	down	0	0	0	350	318	459
MSH2	2.725987117	9.49034E-14	up	2497	2741	2447	186	374	244
MSH6	2.149475916	4.0101E-105	up	3636	3985	3592	635	523	641
MSI2	0.660417187	0.00011109	up	2318	2478	2295	984	932	1277
MSLN	7.918131044	3.02236E-76	up	1463	1667	1629	4	4	6
MSMO1	2.161032638	2.35939E-17	up	2640	2925	2527	366	509	390
MSN	0.753034373	2.634E-07	up	16723	18563	17066	7862	5818	8540
MSR1	-12.66996846	2.4762E-21	down	0	0	0	1398	355	883
MSRA	-3.222829318	1.28865E-07	down	37	36	23	142	350	117
MSRB2	-3.857107209	5.55734E-34	down	51	60	64	819	541	448
MSRB3	-3.660410835	2.8498E-18	down	512	581	468	5030	2096	7234
MSS51	-2.889646986	9.82642E-07	down	18	14	10	54	107	54
MST1	-3.660310001	2.88601E-20	down	7	14	12	111	82	104
MST1L	-3.061583761	0.015068608	down	0	9	2	34	9	22
MST1R	6.325484807	2.4261E-121	up	1635	1757	1672	20	12	13
MSTN	-4.232445551	0.010368809	down	3	0	0	12	4	26
MT1A	-8.855919369	3.87383E-07	down	0	0	0	21	130	18
MT1F	-6.216514447	1.09761E-09	down	1	3	0	72	96	36
MT1G	-4.167017683	0.011302929	down	2	0	3	4	51	5
MT1M	-9.829150522	1.22615E-13	down	0	0	0	100	157	92
MT1X	-1.092826641	0.002485273	down	677	667	713	757	1368	927
MT3	-5.260688237	0.008287895	down	0	0	0	6	5	4
MTA1	0.802915056	7.01577E-10	up	1607	1695	1611	656	645	696
MTA2	1.062786581	2.30477E-16	up	2524	2848	2373	974	816	840
MTAP	1.510734418	1.85048E-19	up	1356	1508	1390	421	270	378
MTBP	2.044267993	3.49853E-14	up	375	388	380	91	47	61
MTCH2	1.729591437	4.065E-05	up	4275	4490	4112	667	1348	657
MTCL1	3.695863905	2.73888E-21	up	899	969	912	46	72	31
MTERF2	-1.783363593	5.38837E-16	down	181	220	170	585	397	417
MTERF4	-1.319830487	3.94608E-18	down	305	318	256	557	464	539
MTF2	1.523826376	3.22678E-36	up	1433	1534	1441	406	310	377
MTFP1	1.462165472	0.027016678	up	262	295	234	42	121	31
MTFR1	1.682305874	3.157E-28	up	1412	1493	1347	329	315	294
MTFR1L	-1.744652645	2.32399E-26	down	433	524	456	1163	1131	1049
MTFR2	4.646363711	3.56039E-25	up	230	280	258	13	3	6
MTHFD1L	1.96592756	2.83675E-13	up	1678	1801	1709	372	339	226
MTHFD2	1.137121197	0.002837992	up	4134	4695	4036	1721	675	1857
MTHFD2L	-0.853303857	0.000740506	down	81	83	76	101	104	102
MTIF3	-2.172673181	2.19548E-29	down	253	267	290	891	892	801
MTM1	-0.763107075	0.004456669	down	238	230	267	295	219	383
MTMR1	1.136651826	1.5579E-05	up	1078	1108	1086	342	247	481
MTMR10	-1.425032378	0.003717621	down	49	43	72	87	141	80
MTMR11	-2.073571827	0.000269553	down	219	205	233	998	196	850
MTMR12	0.607574044	0.000107916	up	1517	1726	1509	723	624	874
MTMR14	-0.631950934	3.73865E-05	down	417	423	373	473	424	437
MTMR2	0.708391849	7.74765E-05	up	2551	2859	2365	1209	858	1337
MTMR3	-1.272979388	2.19823E-14	down	506	543	526	841	810	1056
MTMR6	-0.712815894	6.58281E-06	down	689	724	644	940	714	746
MTMR8	-7.008617734	2.32713E-05	down	0	0	0	20	6	26
MTOR	1.154787621	2.76857E-06	up	2609	2864	2577	664	829	1064
MTPAP	1.078488087	2.16973E-09	up	697	767	681	254	247	217
MTRR	1.446967996	1.55031E-13	up	1926	2123	1872	592	367	600
MTSS1	-8.563389096	5.29065E-38	down	0	5	3	505	648	981
MTSS1L	0.646815791	0.000399433	up	1707	1681	1670	940	597	781
MTTP	-2.78058754	0.000437537	down	4	3	4	13	15	26
MTURN	-3.436906122	1.6151E-103	down	304	309	314	2230	2267	2628
MTUS1	-1.540202927	0.000168641	down	1028	1031	1062	1200	2038	3195
MTUS2	-4.010407055	5.46816E-15	down	58	61	44	502	289	1115
MTX1	1.778825753	1.53217E-15	up	701	688	734	133	161	143
MUC1	4.892810477	5.99E-65	up	2244	2595	2679	82	57	41
MUC12	4.365214651	7.82358E-05	up	42	58	42	5	0	0
MUC13	14.31198894	3.07211E-42	up	18214	20061	18880	0	0	2
MUC16	8.477477368	1.44662E-46	up	1444	2063	1998	0	2	9
MUC17	4.64745655	0.04370194	up	2	13	5	0	0	0
MUC20	-3.648946201	0.031545328	down	0	0	2	7	6	5
MUC5B	5.073046484	0.008329148	up	6	7	13	0	0	0
MUC8	3.868438946	0.039089398	up	12	6	4	0	0	1
MUM1	-1.104157687	5.07903E-06	down	490	490	509	755	553	993
MUM1L1	-6.126569137	0.00062844	down	2	0	0	10	76	7
MUSK	-8.184474084	7.0011E-09	down	0	0	0	38	49	25
MUSTN1	-5.470915278	0.000322556	down	0	2	0	32	6	26
MUT	-1.120446689	0.000323689	down	551	701	586	725	1158	897
MVB12B	-1.738241149	4.40397E-08	down	100	113	76	189	190	306

MVK	1.517563683	7.48652E-05	up	685	802	703	131	249	149
MX1	-6.063482624	1.21206E-34	down	14	12	18	1171	505	455
MX2	-7.459769365	6.67113E-24	down	2	3	1	287	290	162
MXD4	-1.941537423	2.48966E-33	down	530	580	524	1438	1258	1777
MXRA5	-11.87683517	1.90283E-07	down	0	0	0	137	1130	90
MXRA7	-3.368566494	4.1793E-59	down	1092	1112	1002	6847	6743	9997
MXRA8	-10.95300472	2.0138E-46	down	1	0	4	3335	1415	2466
MYADM	-1.794092451	7.84571E-06	down	960	1065	1002	3197	1132	3329
MYB	4.712682618	4.02928E-21	up	164	200	151	5	2	7
MYBBP1A	1.918321886	7.61478E-66	up	1723	1899	1721	361	294	351
MYBL1	1.835563175	0.001686614	up	1050	1136	1086	199	70	403
MYBL2	6.925189968	2.59583E-24	up	4697	5079	4847	42	38	4
MYBPC1	-5.643425057	0.004188566	down	0	0	0	5	10	4
MYC	2.12305899	0.000935047	up	4519	4936	4395	392	1385	360
MYCBPAP	-6.727686997	4.86248E-06	down	0	1	0	43	13	27
MYCT1	-8.929138658	1.02037E-27	down	2	1	2	313	724	663
MYD88	-1.113849318	1.14155E-13	down	284	307	275	489	414	428
MYDGF	1.774951139	1.04512E-29	up	3152	3265	3099	718	657	595
MYEF2	-8.71378947	2.65873E-22	down	6	4	1	1443	196	1777
MYEOV	7.085278162	1.97309E-23	up	331	305	319	1	3	1
MYH10	-7.623499064	7.90183E-26	down	143	131	125	14097	3566	40497
MYH11	-13.78106705	2.85431E-25	down	0	0	0	3137	819	1732
MYH15	3.922764997	8.38157E-06	up	147	151	131	3	15	1
MYH4	4.352325075	0.048732804	up	3	7	6	0	0	0
MYH8	6.614262741	5.70573E-06	up	30	24	22	0	0	0
MYH9	-1.353745735	0.000814529	down	13449	15299	14492	26451	12993	40863
MYL12A	-1.851502194	3.36001E-09	down	3400	3658	3253	8976	10853	6140
MYL12B	-0.795190896	0.000329308	down	4481	4967	4240	7471	4291	5267
MYL5	-2.367178579	2.29099E-07	down	28	29	29	100	139	70
MYL6	-1.425044135	3.99178E-05	down	11788	13241	11773	34938	12945	23868
MYL7	-7.751647319	0.001692058	down	0	1	0	0	140	9
MYL9	-4.62367242	6.43571E-19	down	1841	1914	1783	39757	10098	50537
MYLIP	-5.579665377	6.55641E-29	down	60	67	43	1849	798	3264
MYLK	-4.923597022	3.18323E-12	down	625	650	593	16709	2121	23194
MYLK4	-4.038732793	2.8656E-08	down	4	4	2	35	25	58
MYO10	4.199599044	9.27155E-22	up	12937	14105	13320	248	674	602
MYO15A	-4.475176942	2.89127E-06	down	1	1	3	37	14	30
MYO16	2.136627607	0.033056379	up	68	70	65	1	22	8
MYO18B	-7.349052614	6.64771E-28	down	2	8	2	322	642	370
MYO19	3.162839092	0.000435316	up	42	44	15	4	2	2
MYO1A	2.689399057	1.70282E-06	up	65	51	90	10	6	7
MYO1D	-8.650541699	1.04726E-34	down	26	13	16	4206	1414	10764
MYO1E	2.774688618	7.07404E-27	up	8870	9773	9416	959	657	1335
MYO1F	-6.855382707	6.95114E-29	down	2	3	6	389	167	370
MYO1G	0.700234188	0.049536307	up	96	168	108	59	53	49
MYO1H	-5.499364231	0.004525924	down	0	0	0	9	4	5
MYO6	-0.849275481	7.65544E-07	down	456	576	501	630	571	761
MYO7A	-4.910805975	9.03496E-20	down	10	5	10	122	153	264
MYO7B	3.707656066	7.76584E-09	up	65	76	60	7	2	2
MYO9A	-1.299319731	7.54765E-06	down	552	638	661	893	848	1517
MYOC	-12.98987227	1.35647E-20	down	0	1	0	1911	604	3911
MYOCD	-9.370498294	1.12438E-40	down	7	5	6	3649	733	4437
MYOF	1.408417548	9.26896E-05	up	9631	10775	9451	1764	2192	4052
MYOM1	-5.675004158	1.84126E-30	down	37	43	47	917	2373	1155
MYOT	-10.03723603	8.97601E-12	down	0	0	0	41	160	204
MYOZ1	-5.847021292	2.96677E-27	down	4	4	5	204	114	222
MYOZ2	-8.89317266	6.02246E-21	down	5	8	9	586	5484	791
MYOZ3	-9.197779423	3.13627E-11	down	0	0	0	123	65	44
MYRF	4.951598237	2.83944E-06	up	1248	1451	1393	3	76	7
MYRFL	-5.016943198	0.002781262	down	1	0	2	61	2	11
MYRIP	-10.3399655	3.96218E-16	down	0	0	0	138	181	185
MYSM1	-2.045772119	3.61376E-15	down	254	271	250	610	663	1003
MYT1L	4.08487319	0.000820428	up	30	54	34	0	0	5
MZT1	2.251316864	1.59101E-17	up	1435	1562	1188	278	186	161
MZT2B	-1.228651155	0.001671679	down	734	923	695	2130	859	981
N4BP2	-0.603324236	0.029819981	down	172	204	158	155	192	225
N4BP2L1	-6.850113095	3.30017E-59	down	8	9	8	804	434	843
N4BP2L2	-1.563389187	1.55859E-12	down	896	1024	900	2402	1351	2243
N4BP3	3.081251136	2.4264E-05	up	631	666	634	30	106	17
N6AMT1	-1.622295327	8.90198E-08	down	109	118	87	241	154	299
NAA11	5.790967743	4.87244E-05	up	21	31	32	0	1	0
NAA15	1.814891881	8.16953E-56	up	2495	2805	2394	549	486	516
NAA25	1.78555551	1.32481E-22	up	1707	1817	1674	312	360	394
NAA30	-0.634395541	0.011183676	down	390	471	374	380	494	471
NAA35	1.293883387	7.57883E-10	up	1518	1624	1587	381	465	517
NAA38	-0.957252618	0.006289518	down	492	608	522	1148	550	563
NAA40	1.069621872	7.30752E-08	up	879	890	905	316	318	267
NAA50	1.395990519	9.54728E-31	up	5833	6221	5459	1600	1537	1575
NAAA	-1.028047775	0.006259684	down	284	324	279	620	228	465
NAALAD2	-5.587009596	1.51926E-12	down	0	5	2	78	95	60
NAALADL1	-7.830745745	9.78662E-16	down	0	3	0	233	114	141
NAALADL2	-4.832246189	4.65639E-17	down	9	7	8	136	90	270
NAB1	-0.747391792	8.37077E-09	down	1153	1214	1207	1592	1177	1520
NABP1	0.754562575	3.00091E-08	up	881	912	859	405	350	362
NABP2	0.970871689	2.36708E-09	up	935	1146	927	424	322	344
NACAD	-2.307551631	5.76216E-05	down	39	32	31	162	43	166
NACC1	2.523372503	7.65709E-26	up	2429	2612	2462	273	362	280
NADK2	0.762119715	2.21902E-06	up	1503	1648	1448	597	579	750
NADSYN1	-0.919446646	4.51131E-05	down	380	476	390	696	402	591
NAE1	1.28332674	3.69001E-25	up	1945	2343	1916	624	561	621
NAGK	-1.608076255	4.17919E-17	down	339	334	327	901	602	678
NAGLU	-1.115819878	1.42617E-16	down	448	524	459	835	627	747
NAGPA	0.648423679	0.012864623	up	379	348	344	192	163	130
NAIF1	0.81959917	0.001587497	up	231	271	243	120	99	80
NAIP	-2.238500887	4.5862E-06	down	205	202	215	1144	277	736
NANOS1	-1.29171144	1.69451E-05	down	48	79	53	111	98	102
NANP	1.701369768	4.09279E-06	up	445	506	439	61	118	120
NANS	1.470107223	2.18028E-23	up	1548	1606	1457	414	392	373
NAP1L1	0.787315519	5.76238E-11	up	14184	15823	13864	6985	5194	5932
NAP1L2	-10.91214398	1.36072E-15	down	0	0	0	403	99	278
NAP1L3	-8.518724952	4.82731E-22	down	3	2	0	641	166	535

NAP1L4	1.320393036	2.06129E-07	up	304	339	291	82	101	80
NAP1L5	-0.670084114	0.016852969	down	315	294	294	468	248	319
NAPA	-1.1532645	3.02639E-10	down	729	762	681	1041	1189	1178
NAPB	-1.038309935	6.70939E-07	down	166	199	175	254	218	318
NAPEPLD	-2.149727161	0.029608236	down	1	5	4	12	12	7
NAPG	-0.706844584	0.000538727	down	536	525	515	763	467	617
NAPSA	-6.020337062	0.001423561	down	0	0	0	16	5	5
NARS2	1.198009473	2.02031E-16	up	615	631	580	198	173	195
NASP	1.177478303	1.96536E-12	up	4339	4581	4040	1296	1140	1649
NAT10	1.688858091	1.04725E-33	up	2201	2286	1917	521	390	506
NAT16	6.045957903	0.000223786	up	25	13	13	0	0	0
NATD1	-2.213129296	3.56522E-06	down	135	129	123	213	581	448
NAV3	-3.386485687	3.51138E-26	down	31	44	32	215	289	280
NAXE	0.941211653	0.000177815	up	1581	1749	1541	796	539	471
NBEA	-1.038377986	0.00018489	down	258	228	227	274	337	428
NBEAL1	-1.418488796	8.99732E-05	down	686	735	711	1070	907	2123
NBPF12	-1.050452647	1.29444E-05	down	180	243	206	263	304	352
NBPF14	-0.968157489	0.007536417	down	150	205	149	265	134	311
NBPF4	3.96504116	1.98874E-05	up	31	49	32	2	3	0
NBPF6	5.351390422	2.83793E-08	up	87	83	66	0	4	0
NBPF8	2.048623884	8.94028E-22	up	871	1006	827	139	129	197
NBPF9	0.944889284	1.31027E-06	up	413	413	380	176	135	135
NCALD	-5.989916846	2.40377E-76	down	16	15	15	762	500	839
NCAM1	-6.460186478	5.95873E-20	down	21	13	7	801	1374	297
NCAM2	-9.052815224	2.0857E-08	down	0	1	0	16	165	216
NCAPD2	3.410389866	1.5087E-225	up	7894	8405	7652	599	463	543
NCAPD3	4.051100667	2.42485E-57	up	4709	5180	4510	164	243	201
NCAPG	4.937924823	8.74412E-11	up	2040	2207	1849	105	31	8
NCAPG2	4.172987761	1.0847E-222	up	3957	4328	4004	159	150	175
NCAPH	5.701602794	1.86774E-26	up	2069	1975	1946	53	20	10
NCBP1	1.683515526	1.49928E-37	up	2228	2378	2040	510	475	481
NCBP2-AS2	-0.677700866	0.002514234	down	196	213	211	294	201	213
NCCRP1	5.134931634	3.25208E-05	up	28	32	38	1	0	1
NCDN	0.64133503	0.000447781	up	792	924	917	369	394	432
NCEH1	2.511703018	4.22906E-46	up	1581	1704	1499	222	199	173
NCF1	-8.564076847	4.04637E-10	down	0	0	0	52	29	70
NCF2	-1.598266889	1.39207E-06	down	140	160	122	303	191	425
NCF4	-9.690116076	1.72706E-13	down	0	0	0	161	73	95
NCK2	-2.506357858	4.48449E-22	down	135	156	129	445	584	651
NCKAP1	-0.60713064	0.01843621	down	3731	4040	3707	4313	2802	5470
NCKAP1L	-11.49582962	9.89923E-19	down	0	0	0	500	192	468
NCKAP5L	0.97612838	1.98522E-05	up	747	709	752	223	264	309
NCL	0.853627165	6.14191E-13	up	15431	16720	14412	5960	5621	6703
NCLN	1.695104497	2.6569E-10	up	1973	2207	2131	411	557	395
NCMAP	-5.796743006	0.031103307	down	0	0	1	0	2	42
NCOA1	-1.538603647	2.93744E-10	down	534	579	476	863	1122	1267
NCOA2	-1.515273791	1.52032E-08	down	392	419	384	625	739	1060
NCOA3	0.737192968	0.015214819	up	2638	2773	2641	982	826	1659
NCOA6	0.767510784	1.8634E-11	up	1487	1558	1409	632	564	664
NCOR2	1.208724965	1.46094E-20	up	4037	4354	4347	1273	1192	1454
NCR3LG1	3.916348617	5.55079E-12	up	1298	1511	1276	24	47	122
NCS1	1.588995456	0.003291022	up	3781	3853	3674	1353	279	1137
ND1	-2.332337426	2.43737E-33	down	21447	23411	22245	67430	79147	92369
ND2	-2.085670671	8.70716E-19	down	23611	27523	23590	58355	75593	89595
ND3	-3.263831887	3.1928E-122	down	8172	8985	8459	67810	46006	62371
ND4	-1.440584758	2.12184E-13	down	88565	103727	86777	153677	186782	192438
ND4L	-0.7755931	0.016841919	down	13933	15879	14117	12200	21068	19220
ND5	-2.458296994	6.62705E-45	down	25177	28596	25982	94126	109238	105289
NDC1	2.379769597	1.84001E-81	up	2058	2350	1948	310	254	304
NDC80	3.239111701	8.2435E-33	up	729	798	673	70	56	39
NDFIP1	-1.080839159	2.90829E-05	down	1902	2176	1880	4072	2015	2991
NDFIP2	2.492983289	2.51777E-18	up	2714	2954	2739	283	438	321
NDN	-11.63790904	4.33303E-20	down	0	1	0	1161	689	600
NDNF	-2.972034793	2.04295E-05	down	9	7	13	25	52	85
NDOR1	1.167745779	3.04585E-12	up	671	769	791	233	224	248
NDP	-6.719923201	0.000315476	down	0	0	0	28	10	4
NDRG1	2.34859305	1.08262E-14	up	27769	28987	29635	3121	3115	5909
NDRG2	-8.942097223	3.6997E-109	down	6	9	5	2135	1972	2889
NDST2	-0.904478123	3.57269E-09	down	265	321	288	416	329	420
NDUFA10	-1.132478395	0.007912784	down	833	1065	969	1121	2141	1061
NDUFA13	-0.889964906	0.00022221	down	1748	1942	1892	3066	2366	1910
NDUFA2	-0.889275302	0.001587661	down	573	562	611	842	858	580
NDUFA4	-1.189783083	8.64086E-11	down	2999	3329	3082	5374	5324	4444
NDUFA4L2	-5.928135617	1.36868E-27	down	31	26	27	866	1963	670
NDUFA5	-0.621247704	0.014069005	down	1647	1952	1580	1991	2094	1503
NDUFA6	-1.739537202	0.000105871	down	42	63	35	169	78	87
NDUFA7	-1.098496799	0.001726349	down	277	382	295	547	561	319
NDUFAF5	-0.679283809	0.024985736	down	198	220	221	253	279	187
NDUFB11	0.995705494	1.49163E-06	up	2592	2876	2595	1020	1024	805
NDUFB2	-0.921222881	0.006042718	down	797	976	901	1309	1429	803
NDUFB7	-1.165879888	8.66288E-06	down	846	824	805	1773	1165	1028
NDUFC1	-0.830142922	0.000731435	down	551	645	535	828	767	573
NDUFC2	-1.016273842	0.006055294	down	97	97	79	194	94	109
NDUFS4	-0.921840208	0.000529672	down	708	780	681	1187	995	722
NDUFS6	1.531964303	7.55448E-07	up	3003	3094	3024	894	832	496
NDUFS7	-0.932996073	0.000185668	down	475	571	459	655	773	584
NECAB1	-5.040534775	0.001351953	down	1	0	1	11	29	5
NECAB2	3.154881706	0.000136799	up	41	44	31	1	6	2
NECTIN1	2.463505503	3.01851E-17	up	643	774	718	89	112	70
NECTIN4	4.222439343	1.43099E-08	up	72	93	69	7	1	1
NEDD1	0.975557616	9.04224E-08	up	1027	1052	949	327	340	426
NEDD4L	2.491921973	9.15435E-07	up	1408	1604	1372	115	299	117
NEDD9	-2.100352975	8.66447E-05	down	704	874	830	3972	794	2827
NEFH	-1.615466423	0.014473765	down	10	6	7	17	18	15
NEFM	-4.839670598	0.030374895	down	0	0	0	3	5	3
NEGR1	-2.260437544	2.88932E-17	down	350	435	384	1306	909	1806
NEIL1	-3.934032316	1.39689E-35	down	17	27	17	267	178	219
NEIL3	4.275792182	4.10534E-25	up	230	211	239	11	9	5
NEK1	-1.458680066	1.57009E-15	down	223	229	231	493	347	505
NEK10	3.513248711	8.16975E-15	up	313	344	293	21	28	9
NEK2	7.952357169	8.186E-39	up	2494	2501	2451	17	1	4

NEK3	-2.01659535	1.02839E-08	down	145	153	116	519	220	474
NEK6	2.084295392	9.46176E-09	up	1913	2199	1910	247	459	276
NEK7	-1.069981074	2.92423E-09	down	1636	1871	1540	2233	2532	2706
NELFB	1.25333095	2.80612E-28	up	2141	2316	2197	745	570	675
NELFCD	1.50110968	3.46111E-46	up	2504	2654	2560	702	581	658
NELL2	-2.297912857	0.043582066	down	2	3	5	20	12	3
NEMF	-0.779036804	4.00688E-08	down	788	871	749	987	831	1125
NEMP1	2.044963048	1.44066E-25	up	2113	2233	2172	378	283	471
NENF	-0.947104553	0.001532811	down	939	1078	967	1952	1135	1025
NEO1	-1.065350562	0.011333535	down	831	912	836	1390	604	1934
NES	-2.571332898	7.38573E-18	down	623	694	613	2248	3418	2316
NET1	-1.094015911	0.007378985	down	2123	2191	1930	4228	1449	4048
NETO1	-5.054632493	0.023164123	down	0	0	0	2	4	7
NETO2	3.53201462	1.67751E-15	up	1023	989	956	46	93	38
NEU3	1.594195984	1.03851E-11	up	350	388	365	72	83	104
NEURL1	5.778090745	3.75E-10	up	305	311	355	12	1	0
NEURL1B	-2.505295662	2.95153E-07	down	380	379	406	910	2450	1172
NEURL2	-1.23512177	0.010004462	down	40	27	37	46	74	52
NEXN	-3.762918282	1.46169E-28	down	569	717	596	4940	4032	9357
NF1	0.63222733	0.00253018	up	2893	3328	3026	1283	1168	1802
NF2	1.077622088	3.42174E-07	up	1014	920	866	322	333	283
NFAM1	-3.946944613	1.39992E-13	down	7	8	12	84	67	149
NFASC	-11.5103678	2.96725E-19	down	1	0	0	873	860	469
NFATC1	-2.191601892	6.83345E-21	down	85	82	101	313	231	334
NFATC2	-0.838549666	0.00877661	down	173	216	203	337	156	271
NFATC4	-5.598546323	4.39853E-54	down	34	27	22	1153	575	1185
NFE2	2.488683408	7.94655E-06	up	60	82	86	16	8	5
NFE2L1	-1.136877818	0.000118088	down	3966	4117	4116	5085	4975	9139
NFE2L2	-1.166632534	1.30148E-08	down	1979	2128	1978	4190	2413	3209
NFE2L3	1.217916828	7.44599E-08	up	220	279	217	82	59	78
NFIA	-3.293318755	1.11479E-17	down	425	487	404	4963	1660	2747
NFIB	-3.760339088	1.81305E-63	down	377	397	363	4891	2915	3233
NFIX	-1.965549395	4.30786E-17	down	929	900	968	2599	1879	3384
NFKB2	2.129442938	4.69257E-19	up	2413	2640	2695	503	428	323
NFKBIA	-2.071023244	5.05262E-28	down	500	568	561	1451	1672	1708
NFKBIZ	-0.810169495	0.014534557	down	574	633	584	584	562	1101
NFRKB	1.377465034	1.05055E-11	up	1422	1532	1334	454	277	454
NFS1	1.222828378	0.001463983	up	1381	1447	1286	318	578	323
NFU1	-1.522852758	2.46657E-09	down	362	463	373	774	936	697
NFXL1	0.908332725	0.000506394	up	440	480	476	175	125	234
NFYB	-1.654594831	1.15481E-07	down	794	909	772	1781	1159	2673
NGEF	5.093193172	1.36488E-45	up	588	595	585	20	10	7
NGF	-10.19986132	4.36785E-16	down	0	0	0	172	119	174
NGFR	-6.401608301	4.0958E-13	down	13	7	10	156	1258	280
NGLY1	0.651005575	0.000909309	up	892	1036	935	452	453	381
NHEJ1	-0.884784582	5.40456E-05	down	107	118	99	152	137	135
NHLRC1	-8.368980899	3.34593E-10	down	0	0	0	47	45	37
NHLRC3	-1.831908388	7.49231E-14	down	219	259	273	535	681	667
NHLRC4	-2.635298658	0.000207786	down	4	5	3	19	15	19
NHP2	0.691045268	0.001033761	up	1271	1385	1246	654	582	473
NHSL2	-12.35339886	1.48888E-23	down	0	0	0	714	529	826
NICN1	-1.453006234	5.16682E-06	down	166	156	108	245	222	373
NID1	-13.66795877	1.03654E-24	down	0	1	0	1435	3690	4679
NID2	1.279295723	0.033368541	up	2349	2505	2314	378	1218	402
NIFK	1.578936378	7.35304E-12	up	2031	2199	1909	605	471	381
NINJ1	-0.785093333	0.001790663	down	436	563	530	738	426	730
NINJ2	-0.709221515	0.041439401	down	49	52	60	57	68	61
NINL	-1.346949398	3.28795E-06	down	170	165	164	328	196	392
NIP7	1.289395495	1.75534E-07	up	989	1013	981	321	315	225
NIPA1	2.596707655	5.2412E-22	up	2215	2421	2044	199	231	354
NIPA2	1.822002529	7.77244E-16	up	2682	3057	2692	534	644	494
NIPAL1	1.899830781	0.011018737	up	45	39	35	15	5	3
NIPAL2	-4.030447914	1.72785E-72	down	63	54	70	760	596	834
NIPBL	1.036375163	2.57437E-07	up	3474	3918	3502	1143	1042	1601
NIPSNAP1	1.619757935	5.25849E-09	up	1713	1756	1623	405	462	295
NIPSNAP3A	-1.187976047	8.23101E-06	down	342	301	306	550	566	410
NIPSNAP3B	-4.928142686	3.39379E-41	down	12	17	16	389	215	382
NISCH	-1.502406537	2.1242E-14	down	776	812	777	1636	1194	1976
NIT1	-0.721038308	3.87909E-07	down	374	413	396	517	413	458
NKAIN1	2.749446206	0.003754649	up	26	32	37	6	4	0
NKAIN2	-7.108563714	3.35951E-06	down	0	0	0	15	24	14
NKAIN4	5.685121481	0.002209281	up	5	14	21	0	0	0
NKAPL	-9.620278376	1.37875E-11	down	0	0	0	193	65	57
NKD1	-5.005388446	2.68203E-12	down	33	45	60	1473	191	1627
NKG7	-8.785916416	6.20437E-11	down	0	0	0	70	62	40
NKIRAS1	-1.124645727	1.01451E-06	down	233	306	232	490	294	418
NKRF	2.134129663	3.02398E-13	up	1094	955	973	231	107	160
NKTR	-0.935364346	0.001431576	down	1856	2181	1915	2439	1872	3857
NKX2-1	6.916398719	1.34828E-07	up	59	64	60	0	0	1
NKX2-8	5.446239008	0.001445829	up	9	13	12	0	0	0
NKX3-1	1.169622121	0.000228815	up	107	106	95	35	34	28
NKX3-2	3.696750867	6.76581E-05	up	66	59	92	0	3	9
NLE1	1.879412929	2.4787E-27	up	815	840	740	146	137	180
NLGN1	-10.94266865	2.65397E-15	down	0	0	0	265	96	434
NLGN2	0.931685622	6.51321E-05	up	1605	1787	1729	800	433	701
NLGN3	-1.674012746	0.008934655	down	61	56	50	208	37	148
NLGN4X	-10.49409265	1.20378E-09	down	0	0	0	73	421	29
NLGN4Y	-9.915851928	3.88433E-07	down	0	0	0	148	2	248
NLK	0.79612608	4.05001E-05	up	583	629	602	220	250	269
NLN	2.588887626	5.43995E-18	up	1853	1932	1738	154	260	228
NLRC3	-4.555503484	2.05261E-13	down	4	3	3	66	54	47
NLRC4	-7.755204179	5.57723E-08	down	0	0	0	42	19	25
NLRC5	0.893801143	0.003765616	up	913	915	906	288	434	306
NLRP1	-0.946958189	0.001618209	down	565	763	686	701	809	1243
NLRP12	-6.715533944	2.41713E-05	down	0	0	0	20	8	14
NLRP3	-8.857351287	3.77323E-10	down	0	0	0	95	27	65
NMBR	-5.571360034	0.007049796	down	0	0	0	12	4	3
NMD3	1.21928234	4.88921E-13	up	3115	3544	3015	1190	883	881
NME1	2.972418657	3.38253E-12	up	868	941	826	113	86	38
NME5	-7.288649652	7.31013E-08	down	1	0	0	33	46	39
NME7	1.34920316	2.93091E-05	up	808	869	814	345	145	217

NME8	-6.581820038	0.025899671	down	0	0	0	34	5	0
NMI	-1.272175816	1.77527E-06	down	175	181	132	362	231	248
NMNAT1	-1.296044261	0.000735847	down	97	137	126	153	266	195
NMNAT2	-3.296081741	2.92839E-13	down	16	9	14	75	101	95
NMNAT3	-6.121901058	2.75618E-18	down	2	0	4	112	87	100
NMRK1	-2.937159443	1.07853E-33	down	100	124	86	677	409	613
NMT2	-1.005559934	0.000679134	down	246	366	260	399	475	347
NMU	4.880971599	0.010650459	up	8	9	6	0	0	0
NMUR1	-7.249699268	7.18284E-07	down	0	0	0	27	17	16
NNAT	-6.124999134	0.000183464	down	2	0	0	10	68	16
NNMT	-3.747682099	1.09098E-36	down	359	391	323	2837	4261	2932
NNT	1.252615291	0.021995981	up	3963	4176	3952	621	1963	871
NOB1	1.264431814	9.50217E-20	up	1558	1544	1425	446	432	459
NOC2L	2.234168407	6.18571E-51	up	4230	4708	4194	779	603	602
NOC3L	1.644772187	5.28853E-13	up	1208	1362	1133	244	233	366
NOC4L	0.729409859	0.000224766	up	283	329	313	148	126	122
NOCT	1.936201211	0.000888512	up	148	148	150	19	46	15
NOD1	-1.114078389	1.24201E-07	down	228	267	234	325	369	421
NOD2	-2.387859073	0.000103477	down	9	19	14	67	23	69
NOG	4.168290282	2.30078E-17	up	206	269	252	18	6	5
NOL10	1.245355137	1.08001E-07	up	1090	1114	1022	300	368	288
NOL11	1.410022651	8.70766E-31	up	1844	1992	1783	560	458	485
NOL6	1.156888265	3.79874E-13	up	900	1002	978	333	299	284
NOLC1	2.507617023	3.0918E-123	up	6684	7359	6406	957	734	870
NOM1	1.293143953	6.81329E-18	up	956	1037	980	289	242	334
NOMO1	1.111618217	3.89799E-07	up	349	342	322	109	89	137
NOMO2	1.769404884	7.94523E-10	up	947	1023	1042	162	176	292
NONO	1.200562648	1.06335E-17	up	10539	10848	10084	3760	2577	3469
NOP14	1.203497417	0.000198246	up	1052	1215	990	245	416	323
NOP16	2.068256357	2.44278E-08	up	1176	1187	1105	186	262	127
NOP2	2.87191086	4.53402E-78	up	2222	2367	2107	233	216	199
NOP56	0.909972002	2.19788E-06	up	2008	2222	1824	894	736	652
NOP58	1.149046889	7.23753E-07	up	2122	2471	1973	940	544	634
NOS1	5.291554022	1.01923E-33	up	292	306	284	7	6	3
NOS1AP	1.947309203	1.15035E-10	up	134	153	141	24	22	33
NOS2	3.775093919	6.65296E-20	up	215	171	212	9	13	9
NOSTRIN	-1.743754288	4.72412E-09	down	137	139	126	236	321	394
NOTCH3	-0.824123568	0.000137142	down	3636	4013	4061	4277	4269	6221
NOTUM	7.43795028	1.90278E-11	up	150	160	179	0	2	0
NOV	-3.71626979	7.47428E-06	down	525	538	559	7102	471	8322
NOVA1	-10.65474836	3.2476E-15	down	0	0	0	254	89	307
NOVA2	-3.45931492	9.57851E-08	down	16	12	28	63	177	194
NOX4	-8.05524094	8.51088E-16	down	5	0	6	478	184	1534
NOXO1	4.508825575	0.008911635	up	11	6	17	1	0	0
NPAS2	1.820565008	2.1119E-13	up	1662	1942	1778	288	320	474
NPAS3	-5.884240192	1.11318E-06	down	0	1	1	27	24	33
NPAS4	-6.719362277	3.22438E-06	down	3	1	0	261	10	46
NPAT	1.379854835	1.72008E-14	up	1186	1234	1212	312	348	326
NPC1	1.937365472	3.67666E-43	up	3167	3356	3224	571	551	687
NPC1L1	-3.422869624	2.75405E-06	down	5	8	5	26	69	37
NPC2	-1.025509524	9.94048E-18	down	1673	1641	1536	2470	2009	2566
NPDC1	-1.123410236	4.18693E-10	down	712	691	682	1077	869	1305
NPFF	-3.746906979	2.45104E-07	down	4	5	10	104	29	54
NPHP1	-0.590918291	0.010152785	down	124	140	141	149	146	137
NPHP3	-2.82571908	3.62286E-13	down	38	30	28	233	108	151
NPHP4	0.874598934	0.022113025	up	376	428	403	99	193	167
NPIPA3	-3.592658481	0.04915906	down	0	1	1	10	6	1
NPIPA5	-4.598353169	0.017935594	down	0	0	1	13	3	3
NPIPB11	-1.547169605	0.026434469	down	12	15	13	49	20	15
NPIPB15	-2.591166885	4.57505E-06	down	30	20	20	176	62	69
NPIPB5	-0.699160599	0.004074634	down	539	577	567	824	445	700
NPL	-3.747065663	2.41159E-24	down	46	56	43	339	586	426
NPLOC4	0.690277999	0.001758836	up	3007	3153	3003	1110	1399	1497
NPM1	1.726071465	6.54549E-14	up	30961	33593	29731	9218	5165	6064
NPM3	2.130824207	4.28994E-13	up	682	650	685	113	130	81
NPR1	-1.336383271	0.01199863	down	1400	1682	1355	2210	1092	4849
NPR2	-1.228183469	1.90895E-10	down	612	748	658	1257	825	1305
NPR3	2.066626358	1.62771E-21	up	3535	3541	3372	482	574	711
NPRL3	1.138066455	1.41229E-05	up	730	837	733	188	258	290
NPTN	-0.892805077	0.011920437	down	2031	2285	2024	3683	1460	3408
NPTX1	7.437987114	4.29459E-27	up	2910	3135	2915	30	3	5
NPTX2	-12.33585408	4.25165E-15	down	0	0	0	1393	77	660
NPTXR	-1.595269457	0.007845219	down	315	333	335	902	193	1095
NPY1R	-13.46144283	8.60866E-22	down	0	0	0	2506	387	1709
NPY2R	-5.881297213	0.000743793	down	0	0	0	8	8	7
NPY4R	8.403652438	6.49161E-11	up	88	87	88	0	0	0
NPY5R	-9.594443874	1.06153E-11	down	0	0	0	159	38	116
NQO1	1.707052103	9.50717E-14	up	4709	5372	4617	933	1217	1003
NR1D1	-0.887093078	0.040437409	down	280	299	314	214	427	520
NR1D2	-1.001113739	2.85093E-10	down	829	992	856	1244	1071	1499
NR1H2	-1.053014591	5.1447E-07	down	612	667	675	1045	699	1169
NR1H3	-1.40186073	3.57068E-08	down	375	352	337	564	756	656
NR1H4	5.599037177	5.39845E-10	up	113	134	106	0	5	0
NR2C2AP	0.832936294	0.00033046	up	410	463	343	202	136	148
NR2F1	3.470627657	1.15045E-11	up	1124	1180	1104	29	105	78
NR2F2	-1.472846399	8.07231E-07	down	1081	1062	1056	1541	2497	2178
NR2F6	1.499643015	2.69622E-10	up	1330	1383	1343	367	374	270
NR3C2	-5.743482879	9.95458E-32	down	8	12	3	389	196	297
NR6A1	2.395688888	1.33625E-08	up	88	100	81	12	15	9
NRAS	2.033443159	6.03931E-23	up	3728	4188	3571	649	735	587
NRBP2	-1.931786996	1.80693E-08	down	358	438	371	1084	601	1531
NRCAM	-4.42154393	1.66254E-08	down	4	6	0	69	39	44
NRDC	0.751328006	0.002006189	up	4474	5010	4769	1732	2300	1903
NREP	-1.32934012	0.00025948	down	312	361	337	812	617	366
NRG2	-2.663594715	3.26681E-06	down	10	8	11	28	46	56
NRG3	-6.080558468	0.00146207	down	0	0	0	17	6	4
NRG4	4.842771259	1.64699E-29	up	241	325	247	8	8	4
NRIP1	-2.374082539	5.26465E-08	down	229	227	241	545	1246	701
NRIP2	-10.95286295	1.72319E-16	down	0	0	0	289	331	143
NRIP3	4.511446505	1.7387E-63	up	652	683	619	27	18	16
NRK	-10.78183879	6.93444E-18	down	0	0	0	249	172	276

NRL	-4.058333318	5.64132E-07	down	2	2	2	23	22	26
NRN1	-11.68189625	4.78887E-21	down	0	0	0	388	446	446
NRP1	-3.437080832	5.36905E-23	down	455	523	450	2969	4928	2804
NRP2	-3.003188416	4.10343E-26	down	117	156	137	588	834	885
NRROS	-9.952167885	3.21778E-15	down	0	0	0	124	109	157
NRTN	2.056087386	0.005585727	up	93	89	98	12	29	5
NRXN1	-6.592339969	6.1611E-10	down	1	0	2	65	95	42
NRXN2	-6.351726661	5.56402E-18	down	3	8	1	395	130	177
NSG1	-7.002148215	2.74774E-05	down	0	0	0	18	24	7
NSMCE1	-0.957661359	0.000817709	down	399	493	413	847	421	553
NSMCE4A	-1.620784865	5.29694E-26	down	304	336	315	817	580	700
NSRP1	-0.896516189	0.000619151	down	691	753	744	1100	628	1211
NSUN2	2.672438041	2.69541E-77	up	6521	7139	6169	853	681	675
NSUN5	1.943263071	1.75697E-12	up	762	887	881	217	104	153
NSUN6	-0.929650533	2.65277E-06	down	228	207	216	279	284	318
NSUN7	-10.10862166	3.30338E-09	down	0	0	0	193	10	251
NT5C1A	-6.451229544	0.000415838	down	0	0	0	9	19	5
NT5C3A	1.507044469	9.32214E-08	up	1004	1151	1012	214	320	244
NT5DC1	-1.174627456	1.05E-14	down	491	474	464	769	750	769
NT5DC2	0.960981751	0.000939369	up	1663	1712	1699	584	751	489
NT5DC3	-1.944504944	0.001688174	down	183	171	157	668	120	665
NT5DC4	4.702414174	0.022887312	up	7	4	9	0	0	0
NT5E	0.742527372	0.024972299	up	2429	2532	2192	1291	565	1244
NTAN1	-0.935730004	0.008108003	down	58	43	49	86	55	65
NTF3	-8.834639559	5.17673E-08	down	0	0	1	272	18	76
NTF4	4.922593258	0.00364313	up	9	29	9	0	1	0
NTHL1	1.306660543	3.4408E-05	up	435	541	433	193	85	132
NTM	-5.749817767	1.19174E-08	down	7	0	3	57	216	100
NTMT1	2.116674188	1.38678E-17	up	1464	1723	1582	298	280	197
NTN1	-2.283530244	0.008690205	down	454	499	485	3546	173	1512
NTN3	6.030036048	3.47428E-07	up	49	68	69	0	2	0
NTN4	-1.943403622	5.24527E-08	down	586	688	607	2664	985	1596
NTN5	-4.043798452	0.001613057	down	4	1	0	30	6	25
NTNG1	-6.044861016	0.004570103	down	0	0	0	20	2	5
NTNG2	-5.169957056	2.21726E-09	down	1	4	0	42	43	41
NTRK1	2.451937274	2.21083E-05	up	145	172	176	9	34	19
NTRK2	-3.220912072	1.26557E-26	down	192	236	197	1053	1678	1326
NTRK3	-4.03771983	3.0441E-08	down	163	184	169	3030	295	2965
NTSR1	5.649176103	4.12073E-16	up	136	140	148	3	3	0
NUAK1	-0.875356196	0.009093613	down	921	979	898	825	1457	1302
NUAK2	3.806428807	3.09162E-27	up	433	374	405	15	16	31
NUBP2	0.909828072	1.3362E-06	up	918	957	887	434	300	315
NUBPL	-1.170474324	0.002756401	down	199	234	166	253	426	253
NUCB1	-1.404616477	7.99609E-29	down	1976	2282	2019	3873	3845	4035
NUCKS1	0.681698489	6.09886E-05	up	13606	14676	12576	7382	4718	6137
NUDC	0.917126432	1.0645E-05	up	3191	3425	3128	1588	913	1204
NUDCD1	1.610019615	7.33964E-18	up	1070	1277	1033	278	270	233
NUDCD2	0.634810146	0.001535851	up	697	733	685	405	278	289
NUDT1	1.803690952	2.85083E-10	up	505	550	562	153	94	84
NUDT10	-8.853122449	1.34106E-07	down	0	0	0	123	9	58
NUDT11	-8.974113196	2.17949E-09	down	0	0	0	129	25	50
NUDT15	0.957780342	0.000801887	up	686	722	682	272	294	190
NUDT16	-1.049812982	0.003024503	down	1225	1332	1080	1240	1454	2667
NUDT19	1.125380005	0.02504322	up	546	589	437	88	247	160
NUDT2	-1.041462657	0.006312456	down	241	250	209	540	227	273
NUDT21	0.858666975	1.67944E-14	up	3961	4325	3840	1629	1360	1774
NUDT3	-0.9660456	0.004891855	down	85	59	67	106	72	119
NUDT4	-1.205214513	4.54185E-05	down	1214	1268	1231	1447	2299	2259
NUDT5	1.348967775	9.77079E-11	up	2031	2112	1963	479	600	611
NUDT7	-3.966104162	1.06551E-13	down	19	38	23	260	426	168
NUDT9	-0.856043196	2.59475E-09	down	385	409	404	579	434	535
NUF2	5.444353719	4.37471E-19	up	1282	1489	1236	48	10	9
NUFIP1	0.720331196	0.000952902	up	273	287	284	146	92	129
NUFIP2	1.388127162	1.4999E-15	up	5087	5587	5025	1248	1366	1632
NUMA1	-0.908590213	0.000388141	down	2656	2821	2663	3329	2668	4964
NUP107	1.615420052	6.46103E-27	up	2149	2417	1979	600	426	496
NUP133	0.889961222	2.50489E-15	up	2055	2143	1902	798	722	818
NUP153	1.413794037	1.63465E-11	up	2515	2994	2421	591	667	846
NUP155	3.845185952	1.85257E-86	up	3891	4419	4036	178	221	206
NUP160	3.165674663	5.93106E-71	up	7624	8491	7667	543	611	721
NUP188	2.753761909	3.88334E-26	up	4521	4863	4569	360	518	579
NUP205	2.497270012	1.21926E-20	up	4702	4840	4383	437	536	777
NUP210	5.61403904	4.72727E-51	up	5264	5851	5401	135	48	61
NUP214	1.048173847	1.43138E-13	up	1923	2149	1942	795	548	736
NUP37	1.817596499	3.25936E-16	up	983	1130	953	262	188	168
NUP50	0.601720384	6.10067E-06	up	1508	1650	1617	822	697	717
NUP58	1.762299061	2.07459E-24	up	2219	2341	2012	569	376	439
NUP62	1.175462406	2.04167E-17	up	1114	1225	1071	414	311	350
NUP62CL	5.355478346	3.64479E-23	up	321	371	280	3	2	12
NUP85	1.354994814	3.03412E-12	up	1556	1575	1470	535	375	373
NUP88	1.13088593	1.47276E-11	up	1381	1489	1357	430	473	460
NUP93	2.38889034	6.68739E-72	up	2266	2507	2248	337	310	303
NUP98	1.622264183	2.37842E-18	up	4566	4862	4780	1009	920	1364
NUPR1	-2.777053699	3.21105E-37	down	532	534	487	3038	2475	2036
NUPR2	-7.250611284	0.005684497	down	0	0	0	37	0	26
NUS1	0.673421142	5.3906E-06	up	1582	1827	1518	859	603	741
NUSAP1	5.541940345	1.93306E-33	up	3968	4526	3929	118	38	37
NUTM2D	-3.953072408	0.001375182	down	0	2	2	25	6	14
NUTM2E	-4.991045157	0.041432071	down	0	0	0	4	7	1
NVL	1.081090855	2.43275E-08	up	641	737	641	274	207	197
NWD2	-8.881402972	0.038748571	down	0	0	0	0	141	29
NXF1	-1.224131603	4.88594E-05	down	897	934	848	1334	1017	2149
NXF3	-4.729337937	0.028028638	down	0	0	1	1	14	4
NXN	1.095180007	0.002965754	up	1080	1167	1003	549	199	356
NXNL2	2.366794088	4.33523E-07	up	104	76	98	15	7	17
NXPH3	-6.708082463	2.47444E-19	down	17	23	25	2435	248	2361
NXT2	0.657965925	0.001071899	up	373	394	297	174	143	162
NYAP2	7.435712356	1.31953E-83	up	1613	1787	1698	4	4	13
NYNRIN	-9.327937044	1.94437E-24	down	2	0	1	360	455	550
OARD1	-0.687334592	4.76123E-05	down	363	447	388	537	394	442
OAS1	0.727906266	0.004165089	up	843	948	909	438	413	299

OAS2	-6.299333761	1.58294E-36	down	6	12	8	682	465	300
OAS3	1.865595464	3.13287E-06	up	2167	2313	2159	622	438	231
OASL	2.651583964	0.000201887	up	366	395	286	70	40	8
OAT	-1.002225632	1.63477E-06	down	1347	1428	1279	2068	1383	2381
OAZ2	-0.73482028	2.96624E-12	down	1277	1420	1339	1729	1451	1595
OBFC1	-1.66020463	6.72248E-16	down	211	248	228	466	449	629
OBSL1	-3.110208038	1.06809E-72	down	196	170	200	1282	1004	1210
OCA2	-3.299482068	0.002484151	down	5	11	5	83	4	65
OCIAD1	-1.119125793	3.46727E-19	down	1201	1334	1091	2103	1607	1897
OCIAD2	1.146415611	0.000463595	up	618	616	579	283	118	192
ODC1	1.583708432	5.82915E-08	up	3972	4371	3685	1122	1046	654
ODF2	1.824781542	9.33697E-26	up	2161	2388	2122	411	385	544
ODF2L	0.726385114	0.031685403	up	1302	1386	1186	376	516	766
ODF3B	-3.188054774	3.14612E-12	down	38	27	24	274	186	118
ODF3L1	3.149700391	0.000451107	up	52	57	58	5	8	0
OFD1	0.966056066	0.002058692	up	1128	1406	1054	442	272	606
OGDH	1.016111035	0.029833465	up	5195	5845	5293	1156	2866	1515
OGFOD1	0.803652481	3.16949E-11	up	1348	1492	1281	612	473	597
OGFOD3	-1.194856555	2.55685E-08	down	174	184	186	269	307	304
OGFR	0.81723823	2.37339E-09	up	1093	1175	1128	509	373	495
OGFRL1	-1.258215445	3.25502E-11	down	804	898	695	1365	1078	1648
OGN	-16.49185992	2.88829E-29	down	0	0	0	19359	1882	16554
OIP5	4.804286124	7.57744E-11	up	439	468	416	25	1	9
OLA1	0.939125825	2.59414E-07	up	2411	2704	2180	888	946	845
OLAH	2.061300859	0.025440057	up	22	30	12	4	1	6
OLFM2	-2.015556928	0.024052022	down	162	179	150	176	1011	121
OLFM4	-7.507840363	3.97798E-05	down	0	0	0	57	9	8
OLFML1	-6.052053341	5.69495E-58	down	13	15	14	881	423	708
OLFML2A	2.500549068	6.72246E-14	up	3068	3253	3292	336	533	312
OLFML2B	-1.212313808	0.00029281	down	488	592	514	1334	641	678
OLFML3	-11.44920174	1.41317E-24	down	1	0	1	1199	846	1994
OLR1	5.379677521	2.7164E-12	up	2940	3360	3039	132	14	20
OMD	-14.79525436	4.69125E-25	down	0	0	0	3714	859	7001
ONECUT2	9.484321523	6.34641E-44	up	1629	1688	1718	2	3	0
ONECUT3	5.886426402	0.000206895	up	13	16	17	0	0	0
OPCML	1.173760912	0.044941992	up	72	51	55	26	21	9
OPHN1	0.73247758	0.035311395	up	641	618	554	210	186	387
OPN3	3.235239757	1.42083E-81	up	1897	2044	1975	135	150	160
OPRK1	-5.485187432	0.004654822	down	0	1	0	26	7	2
OPRL1	-2.545763903	0.01327483	down	2	3	2	6	8	15
OPTN	-1.076358666	6.59997E-15	down	1449	1579	1492	2143	2084	2541
OR1F1	5.822997388	0.000254021	up	14	15	15	0	0	0
OR2B6	5.267546497	0.002867068	up	8	11	11	0	0	0
OR2G3	7.444044526	4.42802E-08	up	41	42	52	0	0	0
OR2L2	7.623402177	1.11988E-08	up	50	49	54	0	0	0
OR2L5	5.69321896	0.000558468	up	15	11	14	0	0	0
OR51B2	8.20711919	2.338E-10	up	78	82	70	0	0	0
OR51B4	11.43353886	1.24821E-20	up	663	808	685	0	0	0
OR51B5	9.074732596	1.03684E-39	up	1234	1421	1136	1	0	4
OR51E1	-7.042446488	1.89694E-05	down	1	0	0	15	65	15
OR6A2	7.935764962	1.50121E-09	up	63	61	66	0	0	0
OR8D1	5.50638149	0.001631645	up	12	8	15	0	0	0
ORAI1	1.155144869	0.000316349	up	478	482	460	156	183	108
ORAI2	1.934234027	2.18284E-12	up	1294	1207	1231	192	278	215
ORAI3	-0.782379083	3.83387E-06	down	242	238	210	309	250	286
ORC1	6.176376022	4.32973E-25	up	900	949	959	20	6	2
ORC2	0.737005179	7.65529E-08	up	947	989	861	399	358	436
ORC5	1.10336643	3.01436E-07	up	575	658	541	218	201	164
ORC6	5.06420969	2.31483E-12	up	918	806	723	32	18	2
ORM2	-2.498239296	0.02333611	down	3	3	1	5	8	15
ORMDL1	-1.621976457	1.20982E-27	down	562	595	499	1267	993	1377
ORMDL2	1.158613037	4.18131E-08	up	757	792	745	272	173	293
ORMDL3	-2.004280909	1.11854E-05	down	455	454	402	836	1883	890
OS9	-1.016234289	3.09868E-12	down	2831	2934	2710	4081	3370	4789
OSBP2	5.66187394	2.41761E-21	up	1611	1682	1781	24	39	6
OSBPL10	2.948539628	1.05913E-22	up	2593	2832	2703	170	241	334
OSBPL3	2.352953465	1.78733E-35	up	1364	1586	1423	224	208	173
OSBPL8	0.632389559	0.000276741	up	3120	3144	2975	1339	1200	1713
OSCP1	-1.546368663	0.001235227	down	41	65	55	102	151	72
OSGEP	-0.981495118	6.79627E-05	down	232	309	234	468	276	349
OSM	-7.027770599	0.010359737	down	0	0	0	39	13	0
OSMR	1.417004755	0.000107186	up	6034	7003	6785	1139	2280	1733
OSR1	-12.62222814	5.47197E-17	down	0	0	0	1638	134	818
OSR2	-1.651625092	1.01771E-05	down	101	82	101	226	126	296
OST4	-0.822106373	0.000134891	down	1738	1879	1759	2741	2172	1831
OSTC	0.7420115	0.007685253	up	2101	2196	1981	992	993	655
OSTM1	-1.365615191	8.22822E-29	down	452	524	501	946	820	939
OTC	-5.874700346	0.007393135	down	0	0	0	11	1	12
OTOA	-3.276920757	0.009978525	down	2	0	6	35	8	15
OTOG	6.158364648	7.392E-05	up	14	25	17	0	0	0
OTOGL	-4.072453049	0.000288336	down	0	2	2	18	19	10
OTUD1	-1.205160455	2.56791E-06	down	297	341	267	645	341	513
OTUD6B	0.930492161	1.13271E-05	up	645	669	553	251	240	201
OTULIN	1.777200827	4.70577E-17	up	2500	2544	2151	561	351	596
OTX1	7.50419728	1.44504E-11	up	182	153	176	0	2	0
OXCT1	0.908920366	0.043471422	up	5253	5550	4760	1255	2884	1555
OXCT2	2.38469423	0.044911254	up	18	11	16	2	4	0
OXER1	-7.647264655	2.95639E-08	down	0	0	0	30	22	27
OXGR1	-5.50197067	0.003728574	down	0	0	0	7	4	7
OXLD1	0.610425299	0.001274062	up	449	515	519	261	218	211
OXR1	-1.047056271	1.74142E-06	down	883	857	838	1268	943	1614
OXSM	0.882461519	0.010617187	up	344	402	384	141	182	105
OXSR1	1.706364091	7.6104E-16	up	3386	3767	3491	636	805	858
OXTR	-2.623221858	0.007831201	down	18	38	21	273	51	20
P2RX1	1.631127867	2.50567E-05	up	117	145	161	43	32	22
P2RX4	-1.189810924	4.18862E-05	down	130	199	145	330	182	261
P2RX5	4.533427728	4.04159E-15	up	219	250	202	4	13	3
P2RX6	-1.778719824	0.037702221	down	13	38	28	23	114	44
P2RX7	-6.575092051	5.10072E-23	down	3	0	4	204	129	147
P2RY1	-9.48565715	7.35424E-14	down	0	0	1	149	182	214
P2RY10	-6.631234914	9.42779E-05	down	0	0	0	18	5	17

P2RY11	2.663568677	3.85902E-05	up	51	60	71	3	11	6
P2RY12	-8.854287193	1.57772E-10	down	0	0	0	58	33	94
P2RY13	-9.131189959	2.25628E-11	down	0	0	0	70	41	113
P2RY14	-10.29934624	4.47311E-14	down	0	0	0	192	69	247
P2RY2	2.086312351	8.83082E-05	up	421	432	377	47	112	39
P2RY6	2.512643765	4.42645E-23	up	389	415	391	64	46	39
P3H1	1.325964145	1.22227E-09	up	1738	1862	1841	516	570	442
P3H2	3.393460599	9.69357E-18	up	3428	3808	3361	192	137	399
P3H3	-1.922844025	2.00429E-05	down	169	224	208	929	289	438
P3H4	1.97182974	2.80612E-28	up	1024	1300	1164	254	172	208
P4HA1	2.293702199	3.01532E-23	up	4708	5261	4663	566	774	756
P4HA2	1.365246986	1.94875E-28	up	1958	2176	2093	580	502	639
P4HA3	2.915854163	1.7775E-32	up	536	627	620	61	61	45
P4HB	0.800301697	5.94266E-09	up	13729	14642	13915	5792	5778	5606
PA2G4	1.445593791	4.43183E-24	up	5434	5884	5464	1748	1186	1472
PABPC1	0.905641431	7.82187E-28	up	20532	22628	21247	8724	7113	8653
PABPC1L	-1.276325984	0.002639417	down	99	111	120	235	93	254
PABPC4	0.778595148	6.29806E-12	up	3636	4116	3777	1676	1522	1566
PABPC5	-9.738864972	2.44342E-14	down	0	0	0	128	79	132
PACRG	-7.693976012	0.000512072	down	0	0	0	4	67	3
PACS1	1.259037826	5.36343E-08	up	2882	3246	3011	717	901	1079
PACS2	-1.112193879	5.01685E-15	down	496	537	529	785	721	896
PACSIN1	-4.53236571	0.0046418	down	2	0	1	3	34	10
PACSIN2	-1.138744177	0.002249795	down	1333	1507	1284	2045	1210	3310
PACSIN3	1.832442511	1.39719E-50	up	1966	2205	1976	460	370	397
PAEP	7.083453042	4.9492E-07	up	31	31	43	0	0	0
PAFAH1B2	0.793069524	3.65092E-12	up	2759	2905	2622	1211	1071	1112
PAFAH1B3	2.971042564	6.33642E-13	up	464	456	441	41	57	23
PAG1	-2.646724734	2.83272E-10	down	86	62	68	208	413	324
PAGE1	9.584364519	1.97508E-14	up	210	207	180	0	0	0
PAICS	3.232836802	5.34353E-51	up	7296	7790	7055	553	627	475
PAIP1	1.328318127	6.57231E-34	up	3591	4026	3559	1089	1008	1057
PAIP2	-0.664774679	1.7283E-10	down	1898	2055	1798	2224	2022	2222
PAIP2B	-2.62758635	2.08619E-08	down	63	45	42	142	301	201
PAK1IP1	1.501241751	1.06438E-07	up	1050	1029	870	283	271	180
PAK2	0.717043968	0.000273095	up	3293	3543	3116	1282	1241	1785
PAK3	-3.484739448	7.94937E-05	down	66	66	78	782	47	927
PAK4	2.433840313	7.10758E-54	up	1244	1279	1295	180	163	159
PAK7	-7.670593216	0.000190325	down	0	0	0	44	1	39
PALB2	1.729233728	8.35344E-12	up	650	701	611	109	134	176
PALD1	-6.717011594	3.43419E-34	down	6	2	4	345	321	228
PALLD	-2.317383587	1.51928E-07	down	3292	3704	3412	16090	4923	16936
PALM	-1.21073898	0.000123995	down	399	442	406	529	851	631
PALMD	-4.000431709	5.47937E-09	down	98	150	104	449	2454	853
PAM	-1.291862218	0.000200274	down	6300	6696	6539	12388	6156	16177
PAMR1	-10.67236521	1.35276E-09	down	0	0	0	12	382	212
PAN2	-1.99421904	7.64151E-20	down	266	246	247	654	605	901
PAN3	-1.163086452	6.07745E-06	down	469	459	454	637	588	989
PANK2	-0.782963685	0.000532309	down	515	584	482	684	463	803
PANX1	0.917783823	8.44886E-16	up	1497	1556	1446	582	526	584
PANX2	2.274669563	0.004627828	up	188	168	200	65	9	11
PAOX	-2.837357968	8.17386E-06	down	14	24	4	80	52	80
PAPD5	0.762443605	0.001365997	up	883	864	795	410	243	426
PAPD7	1.671168121	2.62377E-18	up	2630	2789	2772	769	459	619
PAPLN	-1.568679035	0.001980676	down	146	133	142	145	261	483
PAPOLA	0.812008838	1.2602E-10	up	6129	6689	5879	2422	2382	2744
PAPPA	-1.517843445	0.000570904	down	104	129	119	151	330	215
PAPPA2	-4.394683241	4.51498E-07	down	9	20	1	132	210	83
PAPSS2	1.256461211	0.005567962	up	1736	1776	1664	253	647	607
PAQR4	3.776063497	3.71923E-43	up	1506	1647	1569	103	85	56
PAQR5	8.069241669	1.63953E-64	up	2270	2422	2390	14	2	3
PAQR6	2.031089665	1.81226E-15	up	249	254	270	56	39	40
PAQR8	-1.995731107	1.53325E-07	down	37	89	51	165	163	166
PARD3	1.134827847	4.42802E-08	up	2086	2208	2019	597	587	863
PARD3B	-2.985573614	2.62931E-15	down	132	123	122	588	449	1119
PARD6A	-1.84482184	0.000146561	down	17	18	18	43	29	65
PARD6B	2.973879664	6.55697E-08	up	172	174	159	5	19	21
PARD6G	-2.64640299	1.19609E-09	down	13	28	15	83	89	73
PARG	1.126463126	9.1427E-18	up	953	998	956	317	288	342
PARK2	-6.917463121	3.26403E-15	down	2	2	1	78	204	134
PARM1	-14.29893064	2.47754E-28	down	0	0	0	2965	1272	3864
PARP1	2.256442448	5.72296E-17	up	8179	8877	7809	1088	1501	1043
PARP10	-3.446610111	2.11357E-41	down	57	69	42	424	443	422
PARP11	-10.32411968	1.18976E-16	down	0	0	0	175	161	166
PARP14	-0.706836737	0.004601858	down	983	1133	987	1640	907	1085
PARP15	-6.767638173	1.75698E-11	down	3	0	0	69	59	107
PARP2	1.090055171	1.81558E-06	up	957	969	909	405	285	259
PARP3	-0.747562607	0.014458346	down	302	285	239	263	382	327
PARP6	-1.185926601	1.78897E-13	down	433	490	454	693	687	843
PARP9	-1.246580922	9.23509E-10	down	416	484	411	836	740	622
PARPBP	3.856275808	2.38489E-69	up	1009	1038	982	51	55	42
PARVA	-2.208444604	1.40018E-24	down	1072	1270	1105	3385	3064	4908
PARVG	-3.672246148	2.44342E-14	down	27	35	67	464	225	510
PASD1	12.10195245	4.88751E-23	up	1167	1227	1028	0	0	0
PASK	1.392873745	1.31591E-14	up	336	387	350	97	91	102
PATL1	1.478855188	1.93141E-10	up	2010	2033	2003	427	572	527
PATL2	-5.212625443	0.000832293	down	2	0	0	9	30	12
PATZ1	-0.94249896	0.000408443	down	245	281	295	433	251	453
PAWR	0.751011803	0.017823014	up	2608	2931	2414	1505	651	1263
PAX6	6.263436161	4.01577E-11	up	101	121	101	3	0	0
PAX9	4.483891231	0.000254875	up	84	109	80	9	0	0
PAXIP1	1.872613399	5.80907E-14	up	810	834	919	133	159	205
PBDC1	-1.755626261	1.0898E-08	down	187	155	194	599	302	409
PBK	8.395881967	1.5319E-108	up	3066	3067	2883	7	9	3
PBLD	-2.665673349	2.70777E-11	down	29	42	45	127	210	177
PBRM1	-0.748673624	1.49642E-08	down	917	1022	869	1127	967	1263
PBX1	-1.126312536	3.16457E-09	down	968	1112	959	1429	1347	1943
PBX3	-0.991808286	0.000519787	down	332	340	303	353	522	485
PBX4	3.046327982	4.61363E-11	up	106	106	111	15	7	6
PBXIP1	-2.498093756	1.50219E-07	down	970	1005	1019	5606	1467	5352
PCBD2	-1.170784584	0.020404847	down	90	105	82	110	220	98

PCBP3	-6.848528411	2.59945E-09	down	0	0	3	45	121	74
PCBP4	-1.133002165	1.03633E-05	down	359	527	412	826	467	746
PCCA	-1.328030956	0.003166405	down	323	317	319	360	830	470
PCDH1	-1.759773207	1.2399E-05	down	281	267	240	422	869	556
PCDH10	-12.36829116	3.79063E-16	down	0	0	0	703	107	1351
PCDH11X	-4.99400069	0.001208003	down	9	10	1	78	1	389
PCDH11Y	-7.74001469	0.003191707	down	2	0	1	178	0	313
PCDH12	-4.659393838	8.3586E-13	down	12	8	12	100	311	144
PCDH17	-10.57709378	8.09668E-15	down	0	0	0	129	301	149
PCDH18	-13.20878736	3.96976E-25	down	0	0	0	820	1462	1371
PCDH19	-9.127395011	1.25754E-10	down	0	0	0	34	78	105
PCDH20	-6.805021826	6.71633E-12	down	1	5	4	499	38	288
PCDH7	-4.144276587	7.72009E-08	down	304	330	290	5131	470	6529
PCDH9	-2.553055641	2.07487E-13	down	52	40	29	145	159	200
PCED1A	-0.729640527	0.000635365	down	253	312	323	325	346	371
PCGF3	-2.027731713	1.98392E-29	down	292	348	303	1120	759	864
PCGF5	-1.469823867	0.000215157	down	1203	1289	1220	2399	1253	3815
PCGF6	1.150638235	1.01678E-10	up	400	456	440	134	134	146
PCID2	2.192694805	2.60817E-47	up	4348	4830	4521	862	605	671
PCK1	-5.230748784	0.005232064	down	73	72	74	14	5044	224
PCK2	-1.059836537	0.000223483	down	230	255	224	343	412	280
PCM1	-0.728744793	7.70812E-06	down	2089	2305	2059	2833	1935	2892
PCMTD1	-2.112448048	6.53434E-40	down	622	634	625	2231	1500	2102
PCMTD2	-2.050293715	5.28212E-37	down	154	171	150	458	433	505
PCNA	1.683319102	2.41188E-10	up	2497	2724	2323	792	416	478
PCNT	0.594208128	0.00429886	up	1199	1234	1199	486	572	644
PCNX1	-1.386679744	6.94236E-08	down	660	798	739	1060	1490	1482
PCNX2	0.857621513	0.010833069	up	283	305	287	114	71	163
PCNX3	1.814932515	5.88226E-46	up	1729	1882	1765	354	338	393
PCOLCE	-4.040989618	3.8813E-88	down	152	171	168	2150	1928	1641
PCOLCE2	-2.023039195	3.91453E-22	down	689	871	671	1838	2006	2563
PCP2	2.520111889	4.98447E-06	up	61	53	85	12	6	7
PCP4	-7.473834606	0.000101087	down	0	0	0	34	2	37
PCP4L1	-6.158924815	4.03479E-05	down	0	0	1	28	15	12
PCSK1	3.058131562	7.13435E-05	up	357	400	325	18	61	8
PCSK1N	-7.359294252	0.000736521	down	0	0	0	55	1	12
PCSK2	-7.673908623	2.87204E-06	down	0	0	0	23	44	10
PCSK4	-1.331885895	0.036368965	down	13	14	22	47	22	20
PCSK5	-11.268019	2.21717E-19	down	0	0	1	577	485	836
PCSK6	1.744131725	0.029357633	up	1224	1298	1226	42	422	298
PCSK9	11.84310707	9.6838E-69	up	8245	9028	8636	0	5	0
PCTP	-0.713086852	0.000343413	down	242	293	235	277	298	316
PCYOX1	-1.093280701	3.41422E-22	down	2198	2430	2234	3414	3173	3811
PCYOX1L	-2.797479633	3.0659E-17	down	31	39	27	213	122	147
PDAP1	0.600162947	2.95616E-08	up	3428	3724	3261	1758	1375	1764
PDCD10	0.823723757	0.005592852	up	1585	1765	1555	950	440	608
PDCD11	2.040268313	2.6085E-36	up	2544	2664	2306	470	432	391
PDCD1LG2	-7.406610349	2.0715E-10	down	1	1	0	106	48	91
PDCD2L	1.969373788	7.6503E-19	up	309	314	268	62	47	53
PDCD4	0.687821011	2.51236E-09	up	4245	4654	4250	2112	1595	2121
PDCD6	1.607614391	1.38919E-08	up	2614	2964	2706	924	526	495
PDCL	0.669441348	0.000483352	up	973	1070	965	457	345	551
PDE10A	0.742825681	0.013357411	up	503	560	539	167	256	249
PDE11A	-4.29018425	0.023164123	down	1	0	3	1	46	5
PDE1A	-0.666765533	0.027871308	down	667	780	666	764	961	616
PDE1B	-12.11475374	1.94398E-21	down	0	0	0	525	728	454
PDE1C	-2.438510681	0.022792545	down	143	129	130	34	1104	302
PDE3B	-3.111829927	4.86729E-07	down	187	171	214	635	2104	612
PDE4D	1.808507341	1.04435E-10	up	2052	2471	2210	352	543	447
PDE4DIP	-2.383182563	3.52102E-05	down	560	635	518	822	3394	1858
PDE5A	-5.642748402	2.77745E-23	down	116	137	97	6491	1139	5225
PDE6B	-7.704914182	4.48797E-08	down	0	0	0	36	25	21
PDE6D	-0.817207174	2.35509E-07	down	242	262	269	345	288	337
PDE7B	-1.89125515	0.005138451	down	88	84	101	89	415	184
PDE9A	1.296536663	1.96209E-11	up	338	395	372	116	104	99
PDGFA	-1.455326939	0.004082606	down	428	368	407	1316	311	802
PDGFC	-5.84770809	2.21999E-41	down	20	14	18	724	404	1050
PDGFD	-7.258003486	6.9323E-50	down	42	29	33	3915	1623	6139
PDGFRA	-4.667058099	3.5233E-37	down	251	235	247	2606	5108	5344
PDGFRB	-3.626872653	4.5552E-106	down	607	631	686	5693	5710	5428
PDGFRL	-5.221276376	7.2236E-15	down	9	12	28	536	150	666
PDHA1	1.472884121	0.022953636	up	7759	8272	7346	902	3629	1134
PDIA2	-5.210245485	0.004237927	down	1	0	0	6	17	4
PDIA3	0.99177987	2.52964E-11	up	12342	13576	11947	5335	3543	4749
PDIA4	2.532144636	8.49624E-82	up	7333	8252	7551	1022	925	886
PDIA5	1.281697246	3.08316E-11	up	1086	1139	1062	381	307	272
PDIA6	1.714328951	2.75025E-17	up	11482	12334	11313	2553	2781	2197
PDIK1L	-0.731600038	0.000465141	down	135	138	140	163	140	186
PDK2	-1.865675601	1.32365E-09	down	271	312	255	597	900	628
PDK3	-1.804128573	0.005873368	down	960	1052	1046	2755	587	4539
PDK4	-2.397268426	0.017584114	down	2287	2457	2304	700	18684	5101
PDLIM1	-0.996779797	6.75423E-15	down	1739	1889	1707	2618	2095	2884
PDLIM2	-0.733804489	0.019760734	down	663	801	745	1257	561	834
PDLIM3	-8.17752539	1.6041E-101	down	18	29	22	6096	2633	5705
PDLIM4	-6.138454984	9.72812E-16	down	10	1	5	355	108	375
PDLIM5	-1.272191624	1.6308E-21	down	3216	3547	2989	5629	5521	5497
PDLIM7	-1.779698677	0.002303283	down	1208	1289	1185	4775	822	3733
PDPN	-11.07896307	5.19079E-18	down	0	0	0	309	172	382
PDPR	-0.796160374	2.9344E-11	down	872	1010	979	1278	1037	1222
PDRG1	0.804958524	0.000797241	up	590	655	531	279	246	193
PDSS1	1.727098184	7.10218E-09	up	261	251	292	73	56	44
PDXDC1	1.03060237	0.017437168	up	311	290	266	87	57	163
PDXK	1.630405808	0.000119983	up	5921	6240	6047	1066	2042	942
PDXP	1.55985932	3.83387E-06	up	356	377	440	102	112	66
PDZD2	-1.028190365	0.022221921	down	428	521	518	540	1035	484
PDZD3	3.077465294	0.012501196	up	14	23	10	3	0	1
PDZD7	1.970682189	4.63427E-05	up	149	220	174	22	21	56
PDZK1	2.01126765	0.000164137	up	51	59	39	6	10	10
PDZK1IP1	3.843718523	4.05699E-15	up	198	229	226	18	3	12
PDZRN3	-12.38255424	2.46086E-20	down	0	0	1	1566	508	2131
PDZRN4	-10.17443834	8.8002E-09	down	0	0	0	214	7	255

PEA15	-1.580649992	7.12778E-28	down	3700	3604	3423	8991	6246	7729
PEAK1	-1.289600032	1.52734E-06	down	724	935	824	1122	1617	1509
PEAR1	-1.709090615	7.08258E-07	down	342	418	344	546	872	1120
PEBP1	-0.826112343	0.00220965	down	4140	4418	4061	4649	6352	4636
PEBP4	-10.03268986	1.37512E-13	down	0	0	0	216	121	76
PECAM1	-10.93235018	5.149E-80	down	5	2	8	4491	8938	7102
PECR	-3.657384018	2.8869E-06	down	42	44	40	127	755	176
PEF1	-1.217567256	4.50463E-07	down	615	749	615	1435	764	1106
PEG3	-10.53391535	1.5767E-17	down	0	1	0	434	345	355
PELI1	-2.255307891	7.83921E-43	down	167	155	153	593	490	532
PELI2	-4.540736399	9.41365E-32	down	29	34	48	779	354	745
PELO	1.060395016	1.55861E-14	up	1105	1206	1093	385	335	442
PELP1	1.088313529	2.87892E-14	up	1542	1657	1576	627	436	542
PENK	-7.320828237	0.000293715	down	0	0	0	5	47	6
PER1	-1.706775205	7.7161E-11	down	576	589	650	1141	1217	1868
PER2	-1.823970922	0.000112306	down	117	165	120	530	148	357
PER3	-1.376291431	1.70887E-06	down	354	352	386	936	570	525
PERP	2.810518786	3.89445E-73	up	7727	8179	7283	954	624	787
PES1	1.04907857	4.89018E-11	up	1805	1853	1797	662	630	575
PEX11A	-1.748246788	0.025043455	down	188	208	196	190	943	191
PEX11B	-1.024363849	4.93811E-09	down	251	261	208	353	330	355
PEX11G	-2.016124339	8.10502E-06	down	16	23	18	47	66	48
PEX19	-1.316299516	0.000415119	down	911	1106	998	1280	2406	1504
PEX26	0.612691382	0.00093917	up	875	889	932	381	407	463
PEX5	-0.696520293	0.004523507	down	603	670	587	635	814	664
PEX7	-0.852990064	0.001729535	down	91	130	96	159	127	119
PFAS	2.639579697	1.17431E-49	up	1620	1678	1638	175	198	187
PFDN2	1.216815334	1.84074E-05	up	1417	1662	1513	615	452	334
PFDN5	-1.174961714	0.002103567	down	3169	3511	3122	8611	3346	4054
PFKFB1	-7.029662499	0.000112253	down	1	1	2	0	288	55
PFKFB2	2.714276669	1.68242E-17	up	2527	2691	2585	192	346	292
PFKFB3	-2.009211836	7.36661E-06	down	1279	1364	1177	2371	5488	2682
PFKFB4	3.727507386	1.29007E-14	up	910	1060	1058	100	37	28
PFKM	0.816614556	0.007238004	up	4045	4164	3906	1513	2019	1270
PFKP	1.772526261	1.67322E-24	up	5488	6170	5760	1066	1211	1325
PGA3	-5.4460931	0.004252876	down	0	0	0	6	6	5
PGAM1	1.583876679	2.76148E-61	up	3471	3942	3552	917	793	888
PGAM5	2.466066525	1.54154E-11	up	1660	1714	1595	161	294	168
PGAP1	-0.769286374	0.001881248	down	297	299	255	283	344	398
PGAP2	0.900611994	0.002794201	up	335	408	388	116	171	137
PGAP3	-2.466561119	3.85667E-30	down	91	107	82	442	291	372
PGBD4	1.42780186	3.84396E-05	up	204	172	137	36	46	53
PGBD5	-3.438659004	0.013136654	down	1	3	6	13	56	3
PGF	-2.154980099	0.000206872	down	61	47	59	246	210	69
PGGT1B	-0.973041488	2.14554E-12	down	364	417	398	563	483	601
PGK1	2.805070995	6.52254E-61	up	32684	35366	33166	3336	3644	3227
PGM2	0.636819276	0.00116824	up	951	1090	1015	516	343	550
PGM2L1	0.872734284	0.007409423	up	992	1075	1006	417	236	560
PGM5	-4.909382864	1.3664E-36	down	140	148	124	4035	1381	3607
PGP	1.637445776	2.53759E-07	up	1083	1192	1056	248	317	182
PGR	-4.168754887	1.78483E-13	down	74	80	58	1138	268	1392
PGRMC1	-0.733088398	7.8631E-15	down	2460	2607	2418	3180	2545	3144
PHACTR1	-10.51668558	6.84469E-16	down	0	0	1	555	197	394
PHACTR2	-0.686279822	0.021921589	down	784	950	783	729	1128	969
PHB	0.957885196	0.000641418	up	3494	3617	3304	1211	1529	1010
PHC1	-2.613284737	3.39081E-10	down	63	97	55	365	164	418
PHC2	-1.066888381	1.46984E-09	down	1035	1096	1128	1783	1236	1880
PHC3	-0.924742499	0.000303313	down	952	1049	967	1336	925	1789
PHF1	-1.372267322	0.000187474	down	81	85	66	181	82	171
PHF10	-0.650219435	0.046021568	down	530	591	572	665	381	874
PHF13	0.85912065	0.004501827	up	956	1066	995	577	275	346
PHF2	-1.603172073	1.62224E-13	down	387	463	404	986	642	1105
PHF20	0.800297777	2.60937E-05	up	1942	1965	1804	750	627	967
PHF20L1	-0.855494709	7.31702E-14	down	770	805	722	1083	864	1013
PHF23	0.833236544	8.40414E-05	up	1001	1037	946	485	375	330
PHF24	-4.804543832	6.60951E-05	down	2	1	0	26	9	26
PHF3	-0.613495508	0.009806822	down	1455	1467	1279	1429	1172	2000
PHF6	1.786465875	6.82276E-14	up	1615	1817	1557	268	360	390
PHF7	-1.751060433	1.05125E-06	down	40	51	26	100	73	107
PHF8	1.858556076	1.08555E-17	up	1965	2230	1898	371	309	520
PHKA2	0.694901138	1.46507E-05	up	1301	1341	1190	525	551	600
PHKG1	-3.079858071	2.79317E-10	down	19	14	15	150	70	73
PHLDB1	-1.275253848	8.14622E-27	down	1144	1316	1095	2151	1730	2245
PHLPP2	1.090278939	1.11279E-09	up	695	706	620	209	224	239
PHOSPHO1	4.404675708	1.7031E-10	up	173	155	195	2	12	3
PHOSPHO2	-1.221963701	0.003227788	down	27	21	21	45	33	37
PHTF1	1.8291884	1.6447E-10	up	790	902	782	130	134	232
PHTF2	0.712959914	0.030336032	up	1937	2030	1784	920	480	1139
PHYH	-2.399320037	9.67171E-14	down	245	270	217	972	1096	630
PHYHD1	-8.721884322	1.05587E-20	down	1	0	2	288	207	418
PHYHIP	-1.437438801	2.97059E-05	down	40	53	53	126	73	84
PHYHIPL	-6.235518039	5.52021E-07	down	2	1	0	93	13	61
PHYKPL	-1.802792167	6.52666E-20	down	234	275	273	612	675	638
PI15	-8.168803649	9.12879E-11	down	0	1	2	490	76	71
PI16	-7.527417358	9.40922E-89	down	15	9	8	1355	1074	1807
PI3	8.633696367	2.6045E-11	up	90	97	121	0	0	0
PI4K2A	0.732268493	0.003520984	up	1058	1162	1000	359	460	549
PI4K2B	0.736195144	2.74019E-07	up	626	715	588	293	250	279
PIANP	-9.664399646	1.6341E-07	down	1	0	0	339	3	311
PIAS1	-1.190601018	0.001537483	down	647	849	765	1318	647	1772
PIAS4	0.983282637	1.15399E-05	up	546	581	519	185	219	182
PIBF1	0.756064123	0.000541295	up	691	829	659	392	250	278
PICK1	-0.990931815	0.001202125	down	238	244	286	373	241	488
PID1	-6.198646916	8.39812E-18	down	62	54	50	3765	452	4841
PIDD1	-0.720559543	0.018174441	down	117	165	134	219	122	149
PIEZO1	1.457022938	0.003253617	up	4829	5590	5257	660	2001	1256
PIEZO2	2.113063639	1.25547E-05	up	513	703	605	70	154	64
PIF1	2.821870354	1.24562E-10	up	204	293	211	29	29	12
PIFO	-6.320558614	3.92244E-06	down	0	1	1	32	61	16
PIGA	2.106816858	4.15376E-19	up	1075	1130	1136	160	150	244
PIGBOS1	-0.801337075	0.005103876	down	125	105	113	166	141	118

PIGF	-1.040894329	1.73947E-06	down	134	171	116	222	182	210
PIGG	-0.631838895	0.004890794	down	527	636	596	545	617	767
PIGH	-0.981729583	0.003453218	down	216	225	167	400	180	286
PIGL	-1.731276603	9.72297E-13	down	97	122	104	266	189	312
PIGP	-0.708289589	0.023248696	down	290	361	260	408	381	256
PIGQ	1.093414712	9.73284E-16	up	873	964	947	327	290	310
PIGS	1.038585479	6.61332E-05	up	1674	1854	1566	481	682	572
PIGU	0.682964145	0.002132333	up	537	561	534	297	225	201
PIGZ	-2.097280579	4.48265E-16	down	55	74	66	176	198	215
PIK3AP1	-2.115019772	9.52886E-08	down	102	119	92	427	160	397
PIK3C2B	1.588437545	1.53094E-10	up	2521	2863	2530	731	657	468
PIK3CA	-1.370196018	2.18159E-06	down	658	767	701	1031	1052	1836
PIK3CB	1.030778453	1.1482E-15	up	1427	1511	1473	516	493	523
PIK3IP1	-4.3744673	1.84135E-25	down	68	71	66	684	697	1672
PIK3R1	-4.920483135	1.87312E-41	down	132	152	107	2563	1663	4302
PIK3R2	1.732329398	3.23772E-24	up	1563	1696	1554	315	355	353
PIK3R3	-1.950224643	0.000112982	down	384	466	391	948	1752	579
PIK3R4	0.903406246	1.05736E-10	up	1315	1374	1224	479	455	552
PIK3R5	-6.136880255	2.99419E-24	down	3	7	3	247	117	295
PIK3R6	-6.354077527	2.98312E-06	down	0	2	0	32	58	21
PILRA	-2.986716408	1.88288E-18	down	19	21	20	112	88	140
PIM1	1.0947859	0.000286791	up	1609	1612	1643	635	598	373
PIM2	2.740620488	1.45751E-52	up	863	873	781	86	79	103
PIN1	-1.152354059	7.33098E-06	down	399	482	488	977	542	666
PIN4	-0.694772323	0.041690191	down	308	369	332	580	260	337
PINK1	-1.5601314	1.33242E-16	down	946	990	995	1788	2056	2270
PINLYP	-4.860148657	2.97947E-12	down	3	9	2	151	61	78
PINX1	1.046405771	0.00082722	up	329	382	318	171	81	106
PIP4K2A	-1.851864489	1.01255E-06	down	546	562	481	1397	714	2050
PIP5K1A	1.940201807	7.49311E-27	up	3504	3807	3584	851	566	611
PIP5K1B	-5.616336889	9.89032E-29	down	10	5	10	367	314	190
PIP5K1C	-0.641777411	0.000904501	down	834	861	861	1072	700	1093
PIP5KL1	2.027670159	0.000264201	up	97	117	95	20	6	29
PIPOX	-3.361162072	0.008308406	down	4	3	1	36	2	23
PIR	0.971246579	0.023182253	up	175	201	177	109	46	48
PITPNC1	-0.82803267	0.009633337	down	237	258	237	228	364	317
PITPNM1	1.534862351	6.33929E-23	up	1060	1088	1166	279	260	274
PITPNM3	2.420737081	8.70444E-11	up	526	587	540	59	46	117
PITRM1	-0.654993541	1.60642E-06	down	995	1029	919	1182	1052	1053
PITX1	5.849521443	1.34971E-36	up	624	701	669	16	2	7
PITX2	3.334183701	0.00296552	up	32	32	40	1	6	0
PITX3	4.76820914	0.017065918	up	7	5	9	0	0	0
PIWIL1	5.495696385	0.001417524	up	15	11	9	0	0	0
PIWIL2	2.873686489	1.20825E-06	up	171	140	177	26	4	19
PIWIL4	-3.517625113	2.9878E-09	down	13	29	14	190	61	212
PJA1	-1.813157505	2.50271E-05	down	225	227	246	397	837	460
PJA2	-1.013451253	0.000299919	down	3017	3293	3022	5086	2748	5778
PKD1	-1.750442772	2.31783E-05	down	1396	1538	1475	3973	1604	5238
PKD1L1	-0.946500627	2.3215E-05	down	112	115	117	155	133	185
PKD2	-4.493257731	9.85203E-17	down	259	275	265	4752	1319	7156
PKDCC	-4.92632581	2.74749E-64	down	58	38	58	1364	1020	970
PKDREJ	-4.850932439	0.003609917	down	0	0	1	6	7	9
PKHD1L1	-11.35544273	1.25811E-12	down	0	0	0	73	710	175
PKIG	-4.555204309	8.0587E-82	down	82	110	76	1788	1403	1265
PKM	1.857632534	7.25744E-14	up	41479	43985	41557	6499	9344	8749
PKMYT1	6.705345282	5.4397E-19	up	1166	1264	1126	21	2	2
PKN2	0.689456244	9.56887E-06	up	2330	2578	2295	991	944	1239
PKN3	2.306495864	6.51862E-30	up	1391	1495	1378	190	226	191
PKNOX2	-9.770054196	1.6014E-14	down	0	0	0	116	122	102
PKP1	-3.100765313	0.031545328	down	3	3	3	1	40	10
PKP3	6.475767696	5.67283E-13	up	772	816	812	0	15	3
PLA2G12A	-0.995347955	5.87855E-09	down	530	569	512	716	779	773
PLA2G15	0.984537167	6.11414E-07	up	712	734	712	303	189	291
PLA2G2A	-15.3125753	6.02086E-12	down	0	0	0	549	11140	3157
PLA2G4A	1.771191315	0.010108402	up	1793	1935	1722	222	708	142
PLA2G4C	-2.605733645	1.31528E-09	down	43	49	50	334	134	157
PLA2G4D	5.510736754	0.001929777	up	9	19	8	0	0	0
PLA2G5	-9.296458796	1.71794E-12	down	0	3	0	133	893	196
PLA2G6	-2.175413757	5.22939E-11	down	117	69	70	263	274	286
PLA2G7	-7.759930976	3.05562E-05	down	0	1	0	14	130	8
PLA2R1	-5.494805884	2.48416E-55	down	21	21	18	646	431	870
PLAC1	5.566651022	2.75584E-08	up	65	65	68	3	0	0
PLAC8	6.384247204	2.9623E-116	up	1763	1811	1702	12	12	21
PLAC9	-12.88888199	8.82233E-24	down	0	0	0	1401	949	626
PLAG1	2.432182189	6.81062E-11	up	322	432	403	31	60	59
PLAGL2	1.798784407	1.06105E-19	up	734	760	751	140	131	189
PLAT	-2.509782951	0.00014796	down	406	478	503	1517	497	3796
PLAU	-2.123786577	2.38603E-05	down	122	136	127	670	343	187
PLAUR	2.365187483	8.22299E-15	up	1253	1411	1343	277	129	154
PLB1	-2.30244116	6.88748E-06	down	37	29	39	165	54	161
PLBD1	-2.446245811	2.18394E-21	down	98	93	111	397	424	342
PLBD2	-0.688818109	0.007576906	down	1017	1109	1068	950	1169	1527
PLCB1	-2.497557113	4.68464E-08	down	43	73	47	153	298	181
PLCB2	-3.140555315	1.92506E-19	down	41	53	65	413	209	392
PLCB3	1.499516998	1.20104E-30	up	1646	1735	1702	482	355	447
PLCB4	-7.445792452	1.64284E-32	down	1	9	3	480	344	788
PLCD1	-5.270394378	4.57323E-37	down	16	16	13	474	236	547
PLCD3	1.147088433	0.004992842	up	2378	2572	2426	1131	364	936
PLCD4	-2.224955403	8.52071E-05	down	12	17	25	53	80	43
PLCE1	-1.460557084	4.81651E-11	down	235	227	205	428	340	546
PLCG2	-2.843753119	1.25913E-20	down	70	104	79	369	503	394
PLCL1	-9.078856113	2.1774E-47	down	2	3	1	785	680	842
PLCXD1	2.782908843	3.12308E-06	up	97	87	70	3	10	13
PLCXD2	3.787134564	6.46656E-06	up	57	45	56	0	5	3
PLCXD3	-10.56011894	1.23137E-16	down	0	1	1	302	1280	447
PLD1	-1.279614045	3.86402E-11	down	388	359	365	765	492	682
PLD2	-1.018538705	3.58503E-08	down	279	333	313	467	351	521
PLD3	-0.774832546	0.000826137	down	1826	1883	1823	2460	1548	2809
PLD4	-6.628641959	0.002021907	down	1	0	0	73	1	6
PLD5	-7.374829894	0.000712282	down	8	0	2	503	2	770
PLD6	1.502012458	7.15419E-06	up	224	214	184	75	35	48

PLEC	0.597270922	0.000216805	up	8802	9711	9739	4440	3639	5265
PLEK	-10.66653349	3.75619E-17	down	0	0	1	509	258	496
PLEK2	4.644438118	9.03488E-08	up	429	581	519	38	4	3
PLEKHA1	-1.148901668	2.65475E-12	down	457	478	501	724	680	863
PLEKHA2	0.973217412	2.02122E-21	up	1946	2166	1912	755	667	757
PLEKHA3	-1.508957598	2.55876E-12	down	249	221	188	529	356	456
PLEKHA4	-6.311394637	4.14521E-77	down	19	21	23	1601	1053	916
PLEKHA5	-0.848430457	4.98147E-06	down	564	714	516	741	668	881
PLEKHA6	1.323903273	6.23913E-06	up	1351	1402	1302	358	472	302
PLEKHA8	1.750901052	8.40225E-15	up	1110	1313	1113	201	244	297
PLEKHB1	-4.675112299	2.37325E-16	down	8	13	5	186	77	216
PLEKHF1	-1.884702349	6.1558E-11	down	59	90	80	221	206	169
PLEKHG1	-1.930549986	7.5377E-08	down	129	169	135	340	507	297
PLEKHG4	2.57610155	6.30317E-17	up	848	888	918	158	79	83
PLEKHG5	-2.26305956	1.71753E-15	down	86	108	105	266	335	413
PLEKHH2	-2.482867578	6.67693E-11	down	131	120	88	311	382	655
PLEKHM3	-1.682518313	8.12567E-08	down	159	221	155	300	389	520
PLEKHN1	3.522502073	1.61095E-11	up	97	90	105	7	8	3
PLEKHO1	-5.386824161	4.32829E-26	down	53	42	35	2206	532	1257
PLEKHO2	-2.297918492	1.87112E-10	down	226	227	178	1091	640	483
PLGLB1	-2.937647937	0.024020281	down	3	3	0	15	13	4
PLGRKT	-1.513715896	3.68748E-06	down	69	103	94	217	183	136
PLIN1	-11.17106811	5.84574E-11	down	1	3	5	120	11904	1440
PLIN3	2.149786774	8.89376E-50	up	3976	4308	4177	795	543	670
PLIN4	-7.329336241	1.47067E-06	down	47	67	72	315	17709	1240
PLIN5	-6.540365532	1.98213E-07	down	5	3	6	14	729	114
PLK1	5.979660575	3.66267E-57	up	2407	2670	2465	44	27	14
PLK2	-0.733207282	2.24364E-07	down	925	1108	992	1175	1043	1357
PLK4	3.912909522	3.8931E-13	up	770	925	747	43	56	13
PLLP	2.082351657	3.61923E-21	up	400	439	369	84	59	60
PLN	-17.49672787	1.90325E-42	down	0	0	0	37405	12725	24212
PLOD1	1.561909328	2.54616E-05	up	5757	6455	6196	1297	2009	1013
PLOD2	0.880435228	2.50859E-10	up	2893	3131	3085	1141	1037	1345
PLOD3	1.778657621	7.65908E-11	up	2647	2874	2521	446	668	523
PLP1	-9.913912683	7.18966E-20	down	0	2	0	486	475	398
PLP2	1.133916156	0.013538774	up	4807	5296	4825	2569	682	1727
PLPP1	-3.129013996	5.92574E-41	down	381	383	422	1998	2463	2886
PLPP2	4.883684513	2.76115E-22	up	988	1050	975	13	41	16
PLPP3	-4.24838596	5.64408E-96	down	236	261	179	2826	2746	3531
PLPP7	-4.860313909	8.33904E-14	down	8	12	8	135	312	105
PLPPR4	-7.329026303	2.10452E-11	down	5	0	0	107	310	139
PLS1	4.49142372	2.63206E-21	up	565	687	611	18	7	35
PLSCR1	-1.104982014	0.000142287	down	558	586	453	802	959	641
PLSCR4	-4.692419294	7.7242E-119	down	131	130	111	2137	1936	2782
PLTP	-5.148939959	1.59222E-30	down	75	67	68	870	2125	2213
PLVAP	-11.99815188	4.57435E-32	down	1	0	2	1294	3906	3337
PLXDC1	-5.225901406	1.25175E-22	down	6	5	10	132	229	190
PLXDC2	-6.985568132	4.00441E-41	down	59	68	44	5172	1894	8728
PLXNA3	0.684937663	5.69653E-07	up	824	882	857	429	325	383
PLXNA4	-10.34179725	3.71588E-27	down	1	2	0	636	1207	848
PLXNB3	-1.700713722	0.004367585	down	152	176	170	529	108	555
PLXNC1	-7.949933597	1.45381E-29	down	2	3	0	300	248	332
PLXND1	0.667100074	0.002969155	up	5906	6539	6170	2386	3074	2775
PM20D1	-7.946403446	0.00033135	down	0	0	0	1	74	14
PMAIP1	4.92885627	2.00407E-20	up	520	597	518	12	3	24
PML	-1.77846969	2.08116E-17	down	261	287	318	688	741	670
PMM1	-1.251997903	2.62852E-06	down	299	314	316	506	610	437
PMM2	1.957209556	7.39297E-43	up	1491	1545	1508	282	274	274
PMP2	-7.225287118	6.58281E-06	down	0	0	0	18	29	10
PMP22	-1.924618847	8.24223E-17	down	1321	1462	1310	2996	3306	4729
PMPCA	1.9150922	4.39668E-07	up	2874	2965	2799	418	758	408
PMS1	-0.959487753	3.93935E-07	down	230	312	243	375	302	408
PMS2	1.144461124	3.40661E-07	up	605	704	514	188	157	242
PNISR	-1.245086051	2.14076E-05	down	2136	2455	2184	3571	2536	5441
PNLIPRP3	-7.355013334	0.001532147	down	0	1	0	23	0	108
PNMA2	-0.844721015	0.03297343	down	323	355	333	367	262	681
PNMAL1	-11.95497113	3.87597E-18	down	0	0	0	688	167	756
PNMAL2	-8.95104796	6.5156E-11	down	1	0	0	222	83	80
PNMT	-7.005927884	1.5065E-06	down	0	2	0	41	18	126
PNO1	1.193175571	2.56791E-06	up	788	830	763	240	286	205
PNP	1.425196922	2.7201E-06	up	1480	1504	1482	487	422	266
PNPLA2	-4.31617859	8.2397E-07	down	394	378	393	846	11631	2880
PNPLA7	-2.977070408	4.04419E-17	down	51	63	76	455	213	416
PNPO	0.990124835	0.000798607	up	838	877	793	298	359	227
PNPT1	1.582991798	2.7947E-23	up	1814	2021	1753	408	443	467
PNRC1	-2.28592242	3.30471E-46	down	544	622	595	2014	1693	2420
PNRC2	-0.825235823	0.002587877	down	2071	2295	1952	3122	1656	3300
POC1A	3.330763319	1.06549E-11	up	401	424	396	37	37	11
PODN	-14.73807011	4.74452E-27	down	0	0	0	2371	1619	6982
PODNL1	-3.047947641	2.01821E-09	down	10	13	13	108	51	55
PODXL	2.952930729	2.21032E-21	up	18361	19623	17978	1168	2063	1813
PODXL2	3.937892524	1.29745E-18	up	2607	2997	2591	113	188	67
POFUT1	1.31034928	1.24396E-11	up	2926	3172	2761	725	801	1004
POGK	0.761685967	0.000187621	up	1853	2128	1831	813	616	1023
POLA1	3.385392446	1.84087E-70	up	3634	4084	3692	230	222	325
POLA2	3.029622624	3.12987E-64	up	1492	1607	1443	121	139	133
POLD1	2.459609041	7.60924E-37	up	884	976	849	147	99	105
POLD2	1.437213645	3.74584E-09	up	3383	3764	3447	827	1083	829
POLD3	1.512956141	1.62174E-18	up	1030	1169	1005	313	202	288
POLD4	-1.504722789	3.24353E-09	down	412	500	437	1225	770	735
POLE	1.739815377	1.61915E-27	up	1951	2096	2022	451	339	510
POLE2	2.481211912	9.71391E-07	up	353	290	289	63	39	17
POLE3	1.131715676	7.16882E-18	up	1668	1787	1603	549	471	624
POLG	1.268183905	5.2592E-15	up	2154	2393	2300	644	694	670
POLG2	0.793345946	2.14221E-05	up	365	374	318	165	120	150
POLH	1.083953028	2.29728E-08	up	833	745	706	238	248	279
POLI	-1.977317452	2.95256E-21	down	185	210	195	493	484	675
POLK	-0.769070506	1.48347E-05	down	622	699	601	820	601	921
POLN	-1.126701344	0.041818507	down	23	32	45	32	56	66
POLQ	4.927790662	3.86442E-20	up	1216	1278	1111	53	22	10
POLR1A	1.315987133	8.06011E-05	up	983	1131	1026	195	296	399

POLR1B	2.182594605	1.54365E-48	up	1632	1665	1509	280	238	234
POLR1C	1.707465733	5.40352E-23	up	863	919	731	203	170	173
POLR2A	0.852577116	1.2635E-06	up	3652	3827	3533	1343	1236	1767
POLR2C	0.755633618	5.41639E-05	up	2143	2296	2218	1117	689	1026
POLR2D	1.917841444	3.746E-38	up	883	887	854	166	152	176
POLR2E	-0.631900829	0.011844553	down	1184	1299	1202	1218	1566	1217
POLR2H	1.305364301	1.34062E-08	up	1572	1716	1548	592	430	369
POLR2I	-0.963090628	5.27185E-05	down	336	353	367	640	410	423
POLR2K	-0.841638989	0.002929974	down	860	993	784	1557	752	1092
POLR3A	1.4547648	3.72404E-20	up	1091	1199	965	284	277	279
POLR3B	1.034226772	0.002557484	up	459	467	389	144	192	112
POLR3D	1.673710161	6.56072E-14	up	785	892	826	208	196	150
POLR3F	-0.847222206	0.009925815	down	169	252	205	377	208	219
POLR3G	3.601980388	5.72417E-50	up	596	661	545	39	24	43
POLR3GL	-1.755061575	5.63533E-24	down	275	309	238	682	540	755
POLR3K	2.005772654	6.21952E-09	up	880	839	774	198	151	91
POLRMT	1.309537323	4.58869E-21	up	1279	1374	1402	427	358	379
POM121	1.103912683	1.00671E-06	up	1350	1349	1300	359	444	513
POM121C	0.863548234	8.92668E-07	up	1114	1254	1088	403	442	498
POMC	-8.146815066	2.53198E-09	down	0	0	0	51	30	31
POMK	2.103013265	0.030914708	up	45	33	34	2	2	15
POMT2	1.577736345	2.05752E-07	up	800	968	821	147	241	218
PON2	1.754881592	4.53518E-55	up	4325	4822	4530	1040	902	935
PON3	-5.63466869	0.000753643	down	0	1	1	3	46	17
POP1	3.294014681	1.33141E-18	up	1091	1001	936	118	56	48
POP7	1.143759511	0.000254794	up	1026	943	850	432	257	224
POPDC2	-11.80016836	4.99834E-08	down	0	0	0	199	1017	81
PORCN	2.51956733	5.68399E-36	up	976	1079	1146	147	133	116
POSTN	-13.11630942	5.78378E-38	down	3	0	0	3749	9531	5089
POTEF	5.628062478	0.002281107	up	8	8	22	0	0	0
POU2F1	0.951219417	1.08434E-05	up	1191	1176	1125	515	301	484
POU2F2	-4.68557468	6.94877E-08	down	8	11	3	288	39	87
POU2F3	3.102281235	0.040783605	up	14	24	14	0	4	0
POU3F2	3.763006571	0.001567277	up	15	19	24	1	2	0
POU3F3	-5.628372908	0.00036938	down	0	2	1	91	12	5
POU5F1B	2.344725354	0.001019246	up	24	25	36	5	3	4
POU6F1	-2.047582549	6.07344E-08	down	115	146	145	554	210	455
PPA1	1.386194649	0.001901016	up	6320	7132	5995	1205	2634	1261
PPAN	1.704189978	0.004457739	up	25	42	30	8	7	6
PPARD	-0.694315451	0.004767864	down	245	383	362	426	318	396
PPAT	2.926174119	1.24002E-39	up	1132	1235	958	131	92	88
PPBP	-5.946675179	0.048729441	down	0	0	0	0	14	9
PPCS	-1.431287032	1.15337E-16	down	459	550	460	1131	822	865
PPEF1	7.617415579	7.99434E-52	up	852	964	937	5	1	4
PPFIA1	0.614465392	0.000254987	up	1752	1882	1884	782	814	961
PPFIA2	-9.14957689	2.39364E-09	down	0	0	1	214	23	216
PPFIA3	1.015285106	0.001874882	up	202	237	214	93	82	53
PPFIA4	1.946801018	1.35506E-23	up	645	795	775	141	109	160
PPFIBP1	-0.687810218	1.49059E-05	down	2040	2179	1946	2159	2311	2555
PPFIBP2	-2.921903711	6.68813E-53	down	85	81	80	497	363	473
PPHLN1	1.003313388	3.68133E-18	up	1844	1911	1734	722	559	671
PPIA	1.182819547	3.06376E-05	up	15138	16497	14282	6519	4416	3448
PPIB	0.675407839	0.011783127	up	9175	9919	8827	5286	4049	3058
PPID	0.773070064	1.41852E-07	up	1316	1366	1197	624	478	514
PPIG	-0.724243958	0.000610843	down	1399	1472	1309	1972	1157	1837
PPIH	1.136381122	9.95269E-08	up	1026	1077	937	423	278	286
PPIL1	0.764008647	0.040018254	up	853	1039	894	402	490	250
PPL	1.068324645	0.026159742	up	2021	2324	2399	1164	302	892
PPM1A	-1.861674154	1.06315E-18	down	586	602	556	1428	1175	1933
PPM1B	-1.371823999	8.97378E-12	down	712	747	665	1274	1013	1646
PPM1G	1.836937181	3.64242E-16	up	4786	5186	4685	885	1112	880
PPM1H	1.30081442	5.35867E-13	up	578	602	557	203	134	167
PPM1J	-1.860279248	0.000157981	down	17	26	22	84	34	52
PPM1K	-4.134755034	1.88035E-29	down	113	133	136	974	1888	1825
PPM1L	-5.41147648	1.5863E-50	down	17	20	14	389	531	607
PPM1M	-2.37469692	7.32389E-23	down	154	157	162	715	403	650
PPM1N	1.689486555	0.003467869	up	86	87	88	35	8	16
PPME1	1.640339921	2.17683E-37	up	1954	1975	1843	500	376	445
PPOX	-0.682749752	0.001669891	down	165	203	184	255	181	195
PPP1CA	0.724520819	0.000333398	up	2114	2224	2165	934	1009	831
PPP1CB	-1.076779458	0.025707806	down	5873	6344	5526	9510	3578	14243
PPP1CC	0.809676075	8.34881E-10	up	5744	6337	5479	2594	2277	2227
PPP1R12A	-1.301015652	0.003255315	down	1992	2089	1876	5002	1457	4280
PPP1R12B	-4.883151753	6.69001E-21	down	604	691	658	15214	4433	22647
PPP1R12C	-2.517983989	1.22604E-37	down	413	365	375	1701	1195	1850
PPP1R13L	1.414350003	6.96198E-10	up	1219	1318	1301	436	235	366
PPP1R14A	-15.14439682	1.16768E-13	down	0	0	0	7125	428	7343
PPP1R14B	1.19892432	1.23631E-09	up	1949	2239	2036	717	659	539
PPP1R15A	-1.54120767	4.54839E-09	down	541	597	602	1665	978	985
PPP1R15B	1.882164712	3.14515E-42	up	4390	4772	4649	916	885	852
PPP1R16B	-11.0874262	5.44584E-17	down	0	1	0	287	703	636
PPP1R1A	-11.10567301	2.53714E-17	down	0	1	1	412	2005	520
PPP1R1B	-7.644921407	9.86098E-08	down	0	3	1	27	434	50
PPP1R1C	-5.945085753	0.005768224	down	0	3	1	0	145	9
PPP1R21	-1.379493867	3.91184E-13	down	298	285	284	627	410	582
PPP1R26	1.792183397	2.13256E-25	up	1110	1391	1172	231	241	278
PPP1R32	-2.672813925	0.006760809	down	1	19	8	60	19	48
PPP1R35	1.40103001	4.82571E-07	up	750	770	768	289	139	197
PPP1R3B	-1.571727688	0.001471364	down	879	875	825	2791	668	2174
PPP1R3C	-3.263366422	1.12119E-10	down	235	322	255	2342	631	2726
PPP1R3E	-1.160051782	0.000145998	down	152	191	185	292	183	374
PPP1R3G	1.22984068	0.002655804	up	101	105	98	20	37	34
PPP1R7	-0.632077883	8.10607E-05	down	761	838	758	949	849	789
PPP2CA	0.767226292	1.23119E-08	up	3822	4309	3743	1938	1442	1587
PPP2CB	-0.816970735	2.53986E-05	down	1431	1587	1335	1871	1378	2239
PPP2R2A	1.284934495	1.19441E-30	up	2746	3057	2895	849	791	894
PPP2R2C	4.593321988	6.59812E-16	up	347	372	330	9	18	3
PPP2R3A	-0.889797542	0.003760977	down	324	280	237	318	433	342
PPP2R3C	-0.9683939	2.14076E-05	down	311	398	280	539	433	396
PPP2R4	1.311077711	1.6036E-10	up	4015	4379	4025	1189	1301	1031
PPP2R5A	-1.161104111	4.99306E-06	down	1301	1484	1297	2029	2533	1823

PPP2R5B	-0.876579876	0.000448323	down	348	350	355	587	316	491
PPP3CB	-1.750211394	2.82411E-29	down	624	675	590	1810	1228	1503
PPP3CC	-0.652414065	0.023809825	down	338	394	345	353	478	352
PPP4C	0.781736038	5.7365E-07	up	1708	1790	1734	854	648	666
PPP6R1	0.61720852	0.000156163	up	1396	1410	1452	620	604	751
PPP6R2	-0.908804557	2.20913E-07	down	464	567	475	614	637	749
PPRC1	1.862828637	3.2789E-16	up	1332	1487	1338	358	234	223
PPT1	0.675781107	0.015615306	up	4759	4990	4455	2860	1351	2213
PPWD1	-0.629439829	0.025252092	down	730	780	679	998	503	944
PQBP1	0.964193625	0.00076911	up	1589	1708	1465	835	430	488
PQLC1	-1.82924666	4.19044E-12	down	280	246	257	690	739	533
PQLC2	1.008068927	9.87796E-10	up	429	436	407	165	137	148
PQLC2L	-6.386551275	3.48425E-06	down	4	0	1	13	205	65
PQLC3	-2.218542593	4.18539E-21	down	275	291	294	1207	657	1021
PRAM1	-8.356944219	6.74411E-08	down	0	0	0	84	16	33
PRAME	6.339856783	1.88697E-05	up	20	22	21	0	0	0
PRC1	5.248584733	2.57043E-56	up	6541	6877	6416	197	86	93
PRCC	0.94387479	9.69776E-14	up	1215	1433	1312	511	460	489
PRCD	-4.116910095	4.84453E-12	down	11	4	6	66	109	81
PRCP	-0.829132664	6.68125E-05	down	1982	2002	1858	2164	2657	2487
PRDM1	2.225647582	3.67217E-05	up	490	489	610	45	131	57
PRDM10	1.087836408	6.81062E-11	up	526	528	490	176	146	196
PRDM11	-0.854418841	0.007591864	down	209	255	192	267	190	393
PRDM12	6.62302431	4.58769E-06	up	23	31	23	0	0	0
PRDM15	1.977344747	3.22328E-23	up	583	615	625	98	103	128
PRDM16	-3.464874138	9.84314E-08	down	109	78	72	998	158	966
PRDM2	-1.55571247	1.0165E-09	down	423	483	397	803	704	1228
PRDM5	-1.625622214	1.89328E-12	down	87	93	77	207	145	215
PRDM6	-5.98943628	1.02726E-08	down	31	38	46	3621	55	1811
PRDM8	-3.229485106	0.005246479	down	5	2	4	5	47	18
PRDX1	1.767064655	2.51784E-24	up	18159	19007	17356	4783	3081	3600
PRDX4	1.580739126	9.67391E-13	up	4098	4136	3715	1099	972	754
PRDX5	0.798224434	0.007158777	up	5314	5675	5332	2777	2320	1536
PRELID1	0.94560951	1.26316E-05	up	2497	2685	2555	1089	981	770
PRELID2	-0.757040676	0.020451449	down	64	111	82	121	94	92
PRELID3A	1.355865162	0.005560026	up	132	103	103	33	15	48
PRELID3B	2.691833932	7.41582E-23	up	3320	3838	2987	339	450	306
PRELP	-15.93575399	3.17635E-28	down	1	0	0	9942	4159	35414
PREP	0.671381006	0.000611239	up	1033	1077	1014	505	480	402
PREX1	-1.020059364	0.029735215	down	788	848	879	1333	512	1883
PREX2	-2.049366437	5.95044E-09	down	293	285	283	547	1027	916
PRF1	-6.760064536	1.43741E-11	down	0	2	1	69	96	61
PRG4	-4.518285353	1.38957E-11	down	5	9	5	60	72	178
PRICKLE1	-4.843336272	2.71663E-15	down	9	19	7	273	305	113
PRICKLE2	-5.366857084	1.53581E-44	down	29	15	32	604	740	894
PRICKLE3	1.807825374	1.92525E-09	up	524	532	551	135	115	75
PRICKLE4	-1.489786105	5.03657E-06	down	83	95	73	124	180	191
PRIM1	2.283982474	1.22309E-33	up	598	663	609	112	74	88
PRIM2	2.851658311	5.54157E-38	up	968	1032	878	112	64	110
PRIMPOL	-2.206507007	1.56722E-13	down	64	119	81	342	218	307
PRKAB1	-0.715710919	0.000726865	down	286	339	315	356	293	453
PRKACB	-1.898210923	2.77635E-13	down	507	576	526	1889	927	1511
PRKAG2	-2.172808494	2.96424E-54	down	243	302	269	963	759	889
PRKAG3	-5.935978138	0.005270505	down	0	0	0	18	2	5
PRKAR1A	-0.929026352	4.79405E-07	down	3976	4472	3641	4870	4914	6541
PRKAR2A	0.697035399	0.002711955	up	2139	2177	2055	783	1027	954
PRKAR2B	-10.25900123	1.7571E-11	down	7	9	2	268	12327	1536
PRKCA	2.678820327	4.48828E-07	up	3203	3445	3422	159	578	339
PRKCDBP	-2.007633229	5.45889E-09	down	463	545	503	2139	811	1458
PRKCG	5.273177046	1.12471E-16	up	140	139	151	5	0	3
PRKCH	-1.538107196	8.84126E-06	down	267	324	281	400	725	639
PRKCI	2.271165466	1.89647E-30	up	3285	3672	3354	569	358	607
PRKCQ	2.712084932	2.29166E-09	up	217	224	244	25	35	13
PRKCZ	3.715043273	1.16496E-12	up	646	744	731	51	49	13
PRKD1	-4.63134302	1.07947E-33	down	21	25	19	470	221	473
PRKD2	-0.972223964	9.74628E-06	down	280	309	293	503	382	345
PRKDC	2.851538934	2.86903E-31	up	16484	18125	16684	1274	1729	2007
PRKG1	-4.292258995	3.61925E-11	down	221	251	241	4129	679	5518
PRKRA	-1.609513498	4.19421E-13	down	354	359	342	997	573	743
PRKX	1.711510184	1.26181E-15	up	848	921	899	162	182	234
PRLR	-5.870476583	5.88413E-06	down	3	0	0	18	62	42
PRMT2	-2.071906029	6.80813E-23	down	798	761	740	2261	1750	2924
PRMT3	1.715546887	2.63646E-15	up	1034	1166	988	207	256	219
PRMT5	1.500143243	8.44269E-20	up	2368	2404	2049	538	573	594
PRMT6	1.467377937	1.573E-09	up	549	611	537	136	167	128
PRMT7	0.923739594	1.15249E-10	up	822	875	862	359	292	309
PRNP	-2.346980388	3.7565E-30	down	1049	1092	954	4396	2624	4305
PROCR	1.342329469	1.34097E-23	up	1943	2019	1935	567	462	631
PROK1	-6.466319224	0.029394133	down	0	0	0	0	26	6
PROM1	-4.920982918	7.20509E-11	down	12	16	9	358	52	414
PROM2	3.422465558	2.24978E-22	up	315	376	337	26	11	32
PROS1	-2.583017754	8.48591E-08	down	1074	1179	1007	2347	3469	8133
PROSC	-0.886107599	7.60309E-10	down	761	768	738	1128	804	1066
PROSER1	0.640315718	0.000115263	up	694	717	754	337	278	377
PROSER2	3.335477176	1.43769E-14	up	723	833	758	68	21	78
PROSER3	1.400604044	3.74349E-06	up	185	186	195	39	51	62
PROX1	-8.786359691	2.87116E-06	down	0	1	0	8	271	29
PRPF18	-1.207905702	2.99559E-10	down	390	374	379	700	474	722
PRPF19	1.901095195	1.85134E-52	up	4386	4669	4127	953	757	800
PRPF3	0.6017905	0.000465211	up	1798	1813	1645	909	630	943
PRPF38A	0.909141162	1.08261E-08	up	1654	1873	1675	775	510	697
PRPF39	-0.903854148	8.7117E-08	down	506	545	482	820	545	686
PRPF4	1.617656352	1.54691E-33	up	1343	1426	1373	367	280	315
PRPF40B	-1.003540161	0.002079787	down	211	260	205	423	190	365
PRPH2	-8.373829708	1.00931E-09	down	0	0	0	33	53	42
PRPS1	1.738556695	2.09114E-37	up	2305	2514	2144	570	405	514
PRPS2	3.187799881	1.16704E-22	up	4577	5081	4194	506	196	400
PRPSAP2	0.949302566	2.02134E-05	up	693	847	742	315	289	230
PRR11	5.122451416	8.14433E-33	up	3038	3237	3109	108	53	32
PRR13	1.417220537	3.61389E-18	up	3046	3242	2693	956	700	738
PRR14	-0.686433816	0.042710283	down	412	394	391	659	278	460

PRR14L	0.791822665	0.000109724	up	1049	1199	1024	390	403	548
PRR16	-9.729574852	4.6455E-14	down	1	0	0	200	144	313
PRR19	4.000278589	1.63829E-05	up	35	50	30	0	3	2
PRR26	-8.156114491	2.20421E-07	down	0	0	0	54	10	52
PRR29	-7.477122547	4.22184E-06	down	0	0	0	13	39	15
PRR5L	2.026364756	2.62613E-07	up	505	626	555	120	116	54
PRR7	2.536860315	2.60321E-19	up	270	276	290	46	28	29
PRRC1	1.116932477	2.48936E-29	up	2418	2530	2323	844	709	833
PRRC2B	0.907033473	7.89387E-06	up	7006	7927	7078	2504	2319	3541
PRRC2C	0.744591297	0.02173291	up	7534	8498	7835	4881	2016	3421
PRRG1	0.979895141	0.002541194	up	971	1024	1029	248	419	408
PRRG4	1.236905332	0.027641572	up	261	261	266	68	32	144
PRRT2	-4.602468433	5.47258E-11	down	11	15	24	372	74	453
PRRT3	-0.885038124	0.023279678	down	26	36	30	49	33	39
PRRT4	-6.55643025	3.72132E-07	down	3	2	0	30	242	37
PRRX1	-13.77018236	3.04657E-27	down	0	0	1	5544	2259	3072
PRRX2	-2.593959583	0.000124144	down	69	84	68	678	110	193
PRSS12	6.779876915	2.42995E-21	up	265	272	233	2	0	3
PRSS21	13.17466195	2.67507E-27	up	2383	2528	2282	0	0	0
PRSS35	-10.95032781	5.58287E-17	down	0	0	0	400	148	245
PRSS42	-6.142380072	0.000526106	down	0	0	0	6	13	8
PRSS43	-5.535195158	0.002013927	down	2	1	0	4	82	4
PRSS56	6.31982479	2.23763E-05	up	22	20	20	0	0	0
PRSS8	7.364796557	1.13134E-08	up	80	91	79	0	0	1
PRUNE2	-8.147520522	2.25759E-31	down	36	28	44	8068	1411	13332
PRX	-1.123394261	0.000963653	down	110	125	129	157	141	270
PSAP	-0.683364788	4.66709E-06	down	16575	18069	17147	18689	17351	23154
PSAPL1	4.660262645	0.026935988	up	4	11	5	0	0	0
PSAT1	4.03480334	1.13041E-06	up	3009	3265	2814	63	277	27
PSCA	6.062703352	1.39896E-43	up	485	519	509	4	9	3
PSD2	-3.319349947	0.029842158	down	0	1	5	31	9	4
PSD4	0.644532929	0.009859481	up	191	212	209	94	71	115
PSG4	9.354051639	9.7707E-14	up	168	190	152	0	0	0
PSG5	5.768199977	0.000523217	up	15	10	17	0	0	0
PSG6	5.547862544	0.001500605	up	12	8	16	0	0	0
PSG9	6.456702738	1.40901E-05	up	21	20	27	0	0	0
PSIP1	-0.932017852	0.022559691	down	1628	1746	1580	2892	1036	2964
PSKH1	-1.536009686	6.16174E-07	down	214	301	270	414	629	546
PSMA1	0.588473667	9.49719E-06	up	3802	4093	3482	2033	1635	1708
PSMA2	0.735799803	0.002790015	up	3697	3954	3305	1979	1474	1216
PSMA4	0.979270108	4.12883E-07	up	4211	4681	4124	1826	1539	1309
PSMA5	1.888375148	5.75069E-27	up	4292	4664	4157	997	794	723
PSMA7	0.747470991	0.002846551	up	4485	4968	4444	2037	2216	1557
PSMB10	-1.608413906	1.2999E-06	down	158	161	200	472	388	263
PSMB2	1.456338865	1.23868E-34	up	3677	3931	3677	1119	858	953
PSMB7	1.429380155	1.2396E-08	up	4784	5058	4655	1594	1268	952
PSMC3	1.552200605	3.21735E-20	up	6276	6717	6067	1923	1267	1456
PSMC3IP	2.382502059	1.04097E-38	up	531	576	546	79	74	72
PSMD10	0.765414086	3.75882E-05	up	1226	1322	1193	632	478	455
PSMD11	1.413173703	5.01213E-34	up	2951	2963	2710	812	717	772
PSMD12	1.218266807	1.17779E-11	up	2222	2490	1995	738	691	606
PSMD14	1.151344857	3.60362E-05	up	2094	2179	1939	614	801	540
PSMD2	1.508033802	4.17067E-10	up	6598	6979	6370	1307	1821	1800
PSMD3	0.86494051	3.777E-05	up	2060	2216	2092	769	916	774
PSMD5	1.217896775	3.52628E-29	up	2233	2337	2158	705	607	747
PSMD7	0.984626028	1.4891E-06	up	3281	3574	3190	1086	1319	1163
PSME1	-1.363881284	1.75483E-07	down	551	681	595	1495	981	871
PSME3	1.221849158	4.17625E-09	up	3175	3359	3073	811	999	1097
PSME4	0.829470582	0.000311096	up	2321	2492	2288	770	885	1178
PSMF1	-1.461387811	4.23726E-28	down	747	781	696	1671	1209	1493
PSMG1	1.985372895	3.58068E-48	up	1651	1789	1651	328	295	289
PSMG2	-0.9491049	3.21672E-05	down	687	655	623	1183	737	798
PSMG3	2.120733895	7.51499E-33	up	1037	1191	1073	206	175	157
PSPH	1.373850805	0.000287754	up	334	429	347	90	135	72
PSRC1	2.840101034	8.87179E-14	up	621	610	595	92	55	35
PSTK	-0.812943057	0.026434469	down	43	41	39	66	38	51
PSTPIP1	-9.061349824	4.29014E-10	down	1	0	0	247	39	138
PTAFR	-4.851607691	8.32852E-41	down	8	11	17	235	235	268
PTBP1	1.651431322	1.3583E-49	up	6460	6998	6776	1582	1308	1703
PTBP3	2.026765494	2.28336E-31	up	5950	6116	5752	944	1084	1058
PTCH2	-2.37094463	1.19978E-05	down	15	25	14	103	37	61
PTCHD1	-7.703728273	7.17161E-07	down	0	0	0	17	16	50
PTCHD4	-7.277880685	1.11265E-06	down	0	2	0	15	62	139
PTDSS1	1.367460616	2.77622E-18	up	2694	2819	2468	749	747	690
PTDSS2	1.427604783	3.06384E-06	up	305	327	311	62	98	86
PTEN	-0.643746015	0.002572801	down	160	173	193	204	164	219
PTGDR	-8.100111536	3.24152E-09	down	0	0	0	39	24	46
PTGDS	-4.89079432	3.14237E-23	down	53	55	59	941	1807	654
PTGER1	-3.771119271	0.011047027	down	1	0	2	13	13	3
PTGER2	-3.066175292	7.62672E-06	down	9	2	6	30	33	39
PTGER3	-7.98386677	2.44626E-84	down	7	7	3	969	991	1084
PTGES	4.32440758	1.63555E-11	up	2572	2728	2566	21	122	129
PTGES2	1.717566659	1.44561E-07	up	1691	1686	1523	302	458	277
PTGES3	1.081468346	1.68619E-06	up	9978	10646	9042	4291	2321	3474
PTGFR	-7.833856712	2.05055E-21	down	2	1	2	214	383	193
PTGFRN	1.802038327	3.75061E-09	up	5121	5293	4943	880	1329	858
PTGIR	-8.799161793	6.41374E-24	down	2	3	0	923	248	446
PTGIS	-14.25548463	8.83927E-28	down	0	0	1	6735	2125	6517
PTGR1	1.947811719	1.37064E-10	up	2657	2838	2639	692	291	545
PTGS1	1.231314828	4.85595E-06	up	974	955	1030	258	353	274
PTGS2	2.111060436	0.018999151	up	1368	1486	1295	499	169	25
PTH1R	-5.263686642	2.37641E-33	down	8	10	5	172	207	244
PTHLH	-3.578841715	0.001832868	down	15	18	18	333	5	119
PTK2B	-0.676887421	0.006674759	down	355	363	326	354	319	519
PTK6	7.992370733	3.29043E-51	up	1021	1100	1113	1	6	2
PTN	-5.183192067	1.61966E-11	down	53	75	44	568	2992	559
PTOV1	-1.032677107	6.24178E-06	down	602	621	709	1208	722	912
PTP4A1	2.423353186	4.40032E-45	up	7007	7903	6930	1117	936	831
PTP4A2	0.692434952	1.03523E-05	up	6405	7075	6450	2749	2982	2975
PTP4A3	-7.661763095	1.53132E-08	down	0	0	1	46	65	41
PTPMT1	1.404116091	7.38359E-17	up	1440	1675	1389	481	363	365

PTPN1	0.883901341	2.39942E-05	up	1648	1825	1768	572	704	727
PTPN13	-0.861481952	0.000401894	down	541	590	573	615	637	952
PTPN14	1.005917893	0.000745237	up	2813	3004	2895	730	982	1360
PTPN18	-2.063690674	4.39042E-12	down	347	354	311	1048	625	1381
PTPN20	-8.837473365	2.75102E-12	down	0	1	0	134	92	126
PTPN21	-2.149240736	2.38644E-10	down	262	377	372	1171	623	1445
PTPN5	-6.954799459	1.31513E-06	down	0	1	0	39	15	43
PTPN6	-2.335888814	1.26616E-07	down	68	97	70	460	154	244
PTPN7	-8.814677382	1.4452E-09	down	0	0	0	105	25	52
PTPRA	-2.264271463	4.57271E-68	down	427	458	406	1468	1299	1645
PTPRB	-2.640704699	7.91718E-11	down	346	399	427	1056	2366	1636
PTPRC	-9.597925029	7.53123E-17	down	2	0	0	438	190	501
PTPRCAP	-4.070201578	1.60572E-08	down	4	5	10	136	50	46
PTPRD	-1.426554363	0.012748468	down	498	532	523	1378	290	1408
PTPRE	-1.144451059	1.93523E-11	down	175	205	190	307	288	298
PTPRF	1.81414697	0.009178655	up	7845	8852	8231	672	3203	867
PTPRG	-2.587766304	4.35137E-12	down	587	666	569	1771	1933	4125
PTPRH	4.202762241	5.59353E-08	up	152	222	173	2	15	3
PTPRJ	1.474856048	4.57635E-19	up	1420	1497	1295	350	301	429
PTPRM	-1.165792352	1.22612E-06	down	1321	1430	1376	1805	2463	2232
PTPRO	-7.234767952	2.26568E-12	down	2	4	1	303	35	435
PTPRQ	-3.957212433	5.70746E-05	down	2	5	5	13	30	90
PTPRR	4.512876359	2.16392E-35	up	306	313	284	8	12	8
PTPRT	-6.671498539	0.000418255	down	0	1	1	10	119	2
PTPRZ1	-3.882291938	0.000181901	down	5	8	2	15	101	30
PTRF	-1.250726315	2.3649E-05	down	7441	7825	7633	15138	7617	16723
PTRH1	1.465859682	3.45189E-05	up	436	424	404	119	132	70
PTRH2	1.381954936	1.00879E-06	up	667	771	732	246	202	141
PTS	1.128622744	2.22775E-05	up	941	923	848	411	223	257
PTTG1	5.024495134	1.80081E-11	up	2459	2687	2447	132	25	14
PTTG2	-4.956506741	0.0217242	down	0	0	0	4	5	3
PUDP	2.250323678	3.36156E-09	up	877	905	862	98	184	103
PUF60	0.708238745	0.009860548	up	138	141	154	72	47	71
PUM3	0.910667602	1.99907E-08	up	999	1044	908	412	360	341
PURA	-1.039612972	2.07535E-05	down	374	436	343	651	382	669
PURB	0.795337179	2.91977E-14	up	2607	2830	2599	1199	930	1173
PURG	-4.890806845	5.65553E-07	down	2	2	2	64	12	55
PUS1	2.209367152	1.42292E-18	up	790	935	865	173	123	102
PUS3	1.539579192	5.80926E-21	up	624	733	634	172	159	154
PUS7	2.670670285	2.73939E-74	up	1693	1867	1683	194	196	193
PUS7L	1.493889452	1.42869E-09	up	650	729	540	132	165	183
PUSL1	2.834087075	3.01139E-16	up	530	552	480	50	68	35
PVR	1.331055497	1.54538E-09	up	1432	1499	1334	459	416	323
PVRIG	-2.517979127	0.02489087	down	3	1	2	13	5	7
PWP2	3.429002856	0.000806023	up	263	285	254	8	1	46
PWWP2B	1.196871607	0.000140699	up	422	401	416	120	158	101
PXDC1	-2.545184923	1.46117E-07	down	511	594	511	2732	814	3356
PXDN	1.051446798	0.000524096	up	7103	7834	7412	1885	3097	2543
PXDNL	-4.697851794	1.37332E-06	down	4	6	4	23	170	48
PXK	-2.277424929	3.3451E-18	down	136	164	166	450	462	696
PXMP2	-1.295865781	7.44858E-05	down	222	300	227	370	541	366
PXMP4	-0.697951941	0.004146439	down	213	200	180	227	245	207
PXN	0.867045269	7.34249E-05	up	5008	5383	4987	1749	1675	2587
PYCARD	-10.64988871	3.47251E-17	down	0	0	0	279	157	201
PYCR1	4.582573833	4.89772E-18	up	3543	3806	3499	120	155	38
PYGB	-1.159418936	5.15593E-08	down	1647	1607	1693	2631	1977	3330
PYGM	-6.934196469	2.06089E-39	down	3	8	7	473	665	389
PYGO2	0.948572738	6.23441E-11	up	1019	1235	1164	473	356	433
PYHIN1	-6.785354558	3.39352E-06	down	1	0	0	19	20	46
PZP	-5.323925306	6.23458E-05	down	1	0	1	15	26	15
QDPR	-1.827732992	0.001964649	down	270	318	297	478	1276	374
QKI	-1.034035016	0.001960564	down	2237	2298	2262	2354	4166	3175
QPCT	-5.774490498	7.81604E-06	down	4	0	0	32	89	30
QPCTL	2.878185958	1.22374E-50	up	587	594	573	58	49	63
QSER1	2.149214504	3.7496E-52	up	3554	3801	3499	557	582	592
QSOX1	0.649595787	0.000367515	up	3690	3775	3833	1999	1630	1490
QSOX2	2.906543014	1.93108E-89	up	2039	2312	2076	194	200	213
QTRT1	-1.225912019	2.19198E-06	down	242	226	287	541	347	380
QTRT2	1.376929271	2.90069E-20	up	847	978	868	242	242	250
R3HCC1	-0.689861799	0.030058878	down	410	458	336	655	310	432
R3HDM1	1.19278993	7.77979E-08	up	986	1189	1101	280	339	393
R3HDM2	-1.961518472	1.87727E-49	down	293	348	288	882	762	926
R3HDM4	-0.797743342	9.37998E-05	down	241	271	280	350	329	296
RAB11B	-1.658982729	1.54077E-19	down	502	535	500	1223	872	1384
RAB11FIP1	1.316466959	0.000560394	up	1455	1637	1565	264	420	639
RAB11FIP4	1.697422167	3.09375E-06	up	123	129	142	34	32	20
RAB12	-1.155746301	1.37527E-19	down	727	719	643	1141	977	1196
RAB15	-2.988566583	2.58311E-05	down	23	39	31	53	279	168
RAB17	1.706299263	0.011976704	up	121	123	125	56	10	17
RAB18	-0.71311134	4.13278E-12	down	1325	1503	1354	1674	1442	1759
RAB21	-1.192404945	1.83664E-07	down	952	983	929	2012	1087	1616
RAB23	-1.575480106	0.000972358	down	686	770	652	2073	562	1964
RAB24	-0.967393948	0.001044825	down	356	316	391	676	413	404
RAB25	7.637734675	1.60363E-08	up	55	43	56	0	0	0
RAB26	4.868048245	5.07542E-26	up	489	617	625	22	15	5
RAB27A	-2.116316535	1.56021E-09	down	225	239	236	947	381	879
RAB27B	5.948053483	5.1478E-67	up	940	985	947	15	13	5
RAB2B	-1.834632563	1.42735E-12	down	233	212	198	684	368	603
RAB31	-1.590892837	1.81275E-06	down	1432	1526	1231	3727	1622	3800
RAB33B	-1.755438308	4.39998E-08	down	142	159	162	523	231	377
RAB35	0.677951855	4.21364E-05	up	1569	1603	1624	666	640	828
RAB36	-2.014180007	2.63636E-06	down	20	17	20	43	57	63
RAB37	-3.928712584	1.05575E-06	down	4	8	1	67	23	52
RAB38	-5.046869093	0.000488246	down	7	0	0	28	98	34
RAB39A	-5.708383811	0.000614427	down	1	0	0	12	5	24
RAB39B	-7.336170094	3.13859E-05	down	0	0	0	33	4	29
RAB3B	3.011287863	0.001085935	up	24	23	31	5	1	1
RAB3C	-7.307290501	2.50822E-13	down	0	3	0	92	85	160
RAB3GAP1	-1.306303717	3.16154E-08	down	871	955	916	1527	1196	2139
RAB3GAP2	0.640433857	0.001721929	up	1873	1949	1844	855	661	1088
RAB3IP	1.145590164	2.78007E-11	up	428	423	414	144	115	149

RAB40A	-5.956390579	4.00699E-08	down	2	0	3	95	19	118
RAB40C	0.70600975	0.00020616	up	470	539	521	228	177	265
RAB42	2.970344512	1.88239E-10	up	524	605	412	34	71	30
RAB5B	-0.780928306	0.000190363	down	2119	2376	1985	2807	1912	3264
RAB7B	-4.42833238	3.6158E-12	down	9	13	12	210	231	67
RAB8A	1.043687276	1.85293E-16	up	1589	1748	1576	578	480	640
RAB8B	-1.024969082	0.000107524	down	735	748	736	1036	753	1459
RAB9A	2.015848677	2.74339E-62	up	2295	2694	2350	446	409	431
RAB9B	-11.17180571	4.01547E-19	down	0	0	0	341	217	357
RABAC1	-1.931400499	1.87394E-06	down	601	529	588	2476	795	1522
RABEP2	-0.594914444	0.02934823	down	103	121	120	128	92	152
RABEPK	0.987791586	3.09559E-08	up	875	993	857	402	272	306
RABGAP1L	-1.090722746	4.42775E-07	down	549	550	512	960	574	935
RABGEF1	0.705918952	2.82946E-05	up	1392	1452	1333	686	463	686
RABGGTA	-0.627114989	0.002099709	down	231	200	196	232	216	241
RABGGTB	1.193270876	4.79848E-14	up	2571	2769	2317	893	761	719
RABL2A	-1.626453479	5.30101E-06	down	103	88	92	221	125	287
RABL2B	-1.8521719	1.11421E-22	down	113	108	102	313	238	281
RABL3	-0.78229886	1.39527E-06	down	371	465	408	491	465	561
RABL6	0.948480081	8.34597E-05	up	2080	2320	2049	690	910	741
RAC2	-2.083261627	1.82882E-07	down	43	53	56	233	101	131
RACGAP1	4.358521302	1.9478E-159	up	4038	4591	4051	173	136	130
RAD1	1.742064814	3.59308E-16	up	1872	2032	1702	475	275	454
RAD18	2.402202862	2.35022E-28	up	910	933	871	119	136	107
RAD21	1.281745893	9.01755E-19	up	7731	8514	7455	2199	2026	2707
RAD23B	0.890589086	0.000123851	up	6141	6730	6074	2027	2707	2436
RAD51	4.916738057	2.56753E-58	up	648	761	640	20	18	10
RAD51AP1	4.597946385	3.69498E-24	up	1036	1045	915	53	21	15
RAD51B	1.166747033	4.21733E-06	up	178	189	174	72	47	53
RAD51D	0.946858774	0.000503203	up	190	277	235	98	63	99
RAD54B	2.581902227	8.49725E-20	up	487	508	496	46	71	58
RAD54L	6.138105939	1.21848E-73	up	810	883	880	8	8	10
RADIL	-9.296131793	5.11972E-12	down	0	0	0	59	64	125
RAE1	1.526984155	4.09095E-30	up	1174	1310	1184	332	247	329
RAET1E	4.465161054	2.70761E-40	up	319	346	298	10	9	12
RAET1G	1.599308956	0.020670893	up	26	20	22	4	5	7
RAET1L	6.87668719	1.30386E-06	up	29	27	35	0	0	0
RAG1	-1.328353661	0.004743729	down	14	28	25	48	30	42
RAI1	1.458553155	1.44582E-09	up	863	1061	977	209	279	253
RAI14	0.730022321	0.003900752	up	2674	2927	2640	1301	777	1501
RAI2	-12.41919489	6.02246E-21	down	0	0	0	683	345	1171
RALA	2.324317051	8.2705E-18	up	4129	4303	4093	518	723	507
RALB	0.855412221	0.000859454	up	2770	3007	2822	897	1279	1157
RALGAPA1	-1.416546096	1.99066E-09	down	354	497	400	745	624	1009
RALGAPA2	-1.129202256	0.000983111	down	531	554	546	545	928	1034
RALGPS2	3.113613405	4.16403E-14	up	1620	1979	1754	77	189	163
RAMP1	-12.8946888	6.27061E-21	down	1	0	0	3079	562	2398
RAMP2	-12.06989238	1.82533E-21	down	0	0	0	402	551	725
RAMP3	-12.4829782	4.88574E-24	down	0	0	0	795	771	667
RAN	1.947955382	7.67458E-29	up	13328	14475	12753	2952	2363	2143
RANBP1	2.007637262	1.0081E-15	up	3679	4019	3433	917	494	575
RANBP10	-0.59384568	0.000490338	down	355	413	339	386	347	454
RANBP17	1.235767313	2.5807E-06	up	162	217	176	64	42	62
RANBP3L	-7.631552857	1.79399E-10	down	3	6	0	247	50	996
RANGAP1	1.820914478	1.22943E-30	up	2797	3036	2962	556	602	602
RAP1A	-0.688805998	0.011637864	down	1843	2132	1872	2858	1409	2535
RAP1GAP	5.796383169	8.4322E-44	up	827	936	944	8	19	7
RAP1GAP2	2.763016382	7.94968E-19	up	1699	1758	1651	123	217	186
RAP2C	1.689211559	3.79232E-26	up	1919	1997	1725	383	401	455
RAPGEF1	0.874620583	0.009163104	up	3158	3118	3107	796	1366	1431
RAPGEF3	-2.843038955	1.04323E-18	down	215	246	257	985	902	1807
RAPGEF4	-1.768309549	0.000161081	down	54	48	74	95	188	136
RAPGEF5	-10.52473156	2.23339E-21	down	0	2	0	575	832	645
RAPGEF6	0.671379754	0.010833069	up	823	852	839	494	250	395
RAPGEFL1	2.642108263	7.29969E-37	up	446	511	444	64	42	54
RARB	-4.529830738	5.96194E-13	down	19	12	10	99	325	230
RARG	0.629380631	0.007831916	up	936	1096	945	605	337	438
RARRES1	-11.31866504	2.79982E-17	down	1	0	0	299	678	955
RARRES2	-4.202386083	7.3095E-99	down	77	89	72	1086	1046	962
RARRES3	-3.123964554	2.4703E-08	down	48	25	43	384	226	118
RARS	1.449342248	1.08246E-24	up	2985	3171	2745	835	752	722
RARS2	-0.966334134	1.34282E-06	down	431	486	465	662	675	568
RASA1	-0.78214816	3.14912E-05	down	579	640	559	760	554	874
RASA4	-2.911180706	3.35362E-13	down	16	23	34	122	139	127
RASA4B	-2.009003608	8.48818E-07	down	24	31	22	56	83	78
RASAL1	4.531277609	3.2985E-08	up	125	116	111	9	2	0
RASAL3	-8.284078639	8.8097E-11	down	0	0	1	77	74	87
RASD1	-5.655772732	1.96715E-07	down	8	17	11	61	1005	103
RASD2	-2.694604723	0.000552636	down	38	28	60	257	38	315
RASEF	3.684126075	1.16741E-09	up	190	226	191	23	7	4
RASGEF1A	5.177171641	5.81137E-65	up	610	618	601	14	11	11
RASGEF1B	-0.943914568	0.000121952	down	110	133	134	169	181	162
RASGRF1	2.454029797	0.00406001	up	30	29	28	3	7	1
RASGRF2	-7.910452931	4.68905E-29	down	3	2	2	255	504	414
RASGRP1	-6.306295562	1.07345E-07	down	2	0	0	38	33	42
RASGRP2	-6.989969246	1.83966E-35	down	4	8	6	720	256	685
RASGRP3	-1.192654687	0.044238611	down	142	176	168	136	434	185
RASGRP4	-5.874990598	8.84224E-12	down	2	0	3	82	40	92
RASIP1	-6.656481388	3.79232E-26	down	2	10	2	311	410	250
RASL10A	-3.004665276	0.001084192	down	3	2	5	31	8	20
RASL10B	-4.434085485	2.05261E-13	down	12	9	5	71	155	167
RASL11A	-4.541534004	2.82489E-15	down	33	29	45	612	209	1016
RASL11B	-5.999847909	1.7013E-29	down	4	6	2	170	197	172
RASL12	-8.812387553	1.3189E-29	down	6	6	3	2738	344	1898
RASSF10	-3.648432711	0.043538465	down	0	2	0	6	8	3
RASSF2	-1.194865638	0.000572597	down	452	473	431	833	404	1014
RASSF3	-2.472866824	5.69489E-06	down	880	904	827	5671	1059	3955
RASSF4	-6.332413063	9.266E-74	down	23	19	21	892	1257	1436
RASSF5	0.861749051	0.004007189	up	394	399	401	191	95	189
RASSF7	4.094427262	1.52161E-06	up	170	191	192	17	0	7
RASSF8	0.789728528	0.000249808	up	2104	2243	1934	981	603	1027

RAVER1	0.946042419	3.23897E-07	up	799	743	828	338	265	275
RAVER2	-0.91827303	1.0748E-06	down	445	425	376	517	515	641
RB1	0.755359279	0.000180256	up	2147	2212	2100	829	984	882
RB1CC1	0.795770389	1.7759E-05	up	3434	3607	3314	1245	1299	1691
RBAK	1.170689617	8.78268E-07	up	1182	1206	1224	325	321	499
RBBP5	1.335247176	7.36619E-14	up	957	1089	915	254	249	330
RBBP6	0.729796799	2.73649E-06	up	2556	2737	2554	1347	892	1148
RBBP7	1.617472167	1.60427E-13	up	7260	8272	7426	2316	1255	1809
RBBP8	2.221966506	3.06126E-16	up	1182	1254	1180	147	150	256
RBBP9	-0.696988981	0.024109099	down	442	549	455	600	344	744
RBFA	-1.185691523	4.63741E-08	down	171	238	155	321	269	318
RBFOX2	-1.397344723	1.40874E-06	down	93	125	121	230	146	265
RBKS	1.19703472	0.003246361	up	70	98	70	28	27	18
RBL1	1.894734541	3.31324E-30	up	647	709	681	139	105	147
RBL2	-0.966426195	0.004964401	down	1661	1773	1579	1863	1618	3551
RBM10	0.73328618	8.6578E-06	up	1365	1358	1287	686	500	535
RBM12	0.95017854	7.14525E-06	up	2016	2116	2010	988	612	676
RBM12B	1.257509572	2.12569E-19	up	1635	1930	1581	573	417	544
RBM14	0.885254108	0.006384852	up	1195	1188	1072	661	289	402
RBM15	1.709997375	4.98131E-20	up	861	897	851	235	163	171
RBM19	1.078741652	5.58475E-05	up	881	952	945	247	273	416
RBM20	-2.03382721	1.29419E-05	down	185	179	158	255	674	552
RBM27	1.403918033	7.68698E-11	up	2657	2772	2536	854	490	823
RBM28	1.496568266	7.62878E-08	up	1114	1237	1075	213	326	312
RBM34	0.676917734	0.000469339	up	1311	1497	1249	725	557	519
RBM43	-2.742183643	1.21606E-24	down	71	92	73	354	286	487
RBM44	-1.221543839	0.001363178	down	42	44	28	66	46	78
RBM47	1.105097367	7.86394E-09	up	568	695	546	182	187	226
RBM5	-1.861548808	1.45839E-42	down	1022	1118	922	2749	2194	2989
RBM6	-1.809338716	9.13375E-10	down	1017	1144	1035	3148	1553	3404
RBM8A	0.600491478	4.15642E-05	up	1957	2081	1887	959	926	880
RBMS1	-1.138076621	1.49922E-09	down	1189	1432	1264	1762	1974	2305
RBMS3	-4.20338143	1.83867E-47	down	135	150	142	1997	1165	2517
RBMX	0.681320518	1.16319E-07	up	5166	5962	5230	2708	1954	2622
RBMXL1	-0.627577069	0.003662266	down	439	557	522	614	411	655
RBP1	-11.57989257	8.25177E-16	down	0	0	0	854	183	202
RBP4	-5.063116475	4.48828E-07	down	181	196	183	386	10008	1854
RBP5	-5.746353722	2.37666E-08	down	1	2	0	30	40	43
RBP7	-6.349515648	4.99075E-21	down	9	13	10	235	1034	494
RBPJ	-1.036079727	0.000345954	down	1479	1485	1335	2078	1366	2911
RBPMS	-4.268299738	5.06027E-24	down	243	214	261	4164	1441	4545
RBX1	-1.009059118	0.000482045	down	603	663	523	1190	719	660
RC3H1	-0.946114298	1.15329E-07	down	487	504	552	734	705	669
RC3H2	0.837939725	0.019966555	up	851	1031	862	228	382	468
RCAN1	-1.615037265	8.86772E-08	down	525	643	591	1759	1171	901
RCAN2	-15.28483329	2.46394E-31	down	0	0	0	5770	2254	8063
RCAN3	1.736858617	3.55592E-13	up	194	246	218	48	44	48
RCBTB1	-1.758219778	2.54868E-18	down	270	309	212	632	539	730
RCBTB2	-4.442117159	1.8254E-84	down	52	51	59	1013	731	771
RCC1	3.689768364	6.96299E-25	up	2912	3069	2659	134	218	112
RCC2	2.815752624	1.53972E-08	up	115	92	83	6	13	10
RCCD1	2.258592344	1.21114E-24	up	850	1102	1036	172	146	124
RCE1	1.395985756	7.55102E-06	up	275	302	332	114	70	63
RCHY1	-0.851334148	0.000151218	down	318	329	235	441	341	348
RCOR1	1.914061906	1.64081E-29	up	1822	1960	1831	325	366	361
RCOR3	-0.62656989	0.00106751	down	671	723	706	790	595	937
RCSD1	-11.1543542	1.83766E-19	down	1	0	0	536	588	608
RDH11	0.849511222	1.5579E-05	up	2098	2285	2096	1061	625	896
RDH5	-4.245556714	4.05109E-08	down	15	13	10	59	313	113
RDX	1.05381735	0.000155113	up	5227	6206	5230	1400	2120	2100
REC8	-1.973794316	0.000284263	down	25	12	30	88	49	54
RECK	-2.158431081	6.59476E-09	down	273	303	296	816	548	1449
RECQL4	3.295288138	1.63425E-18	up	546	618	587	64	39	24
REEP1	-1.088158056	0.034836748	down	376	455	323	807	212	773
REEP2	-7.088521767	5.95029E-12	down	0	1	3	183	43	175
REEP4	2.332061937	5.41891E-26	up	829	906	884	128	88	157
REEP5	-1.664456839	2.11166E-13	down	1491	1626	1402	4099	2285	3931
REL	1.216331408	9.24298E-05	up	428	548	448	110	123	202
RELA	0.624237055	4.01385E-05	up	1603	1776	1688	840	618	893
RELL1	-2.138388789	5.0701E-09	down	101	131	101	346	436	237
RELL2	0.86713646	0.031281576	up	75	77	115	42	29	34
RELN	-8.897476904	7.126E-11	down	1	0	0	86	179	88
RELT	3.110604931	2.41988E-36	up	559	647	530	63	41	39
REM1	-9.877480937	2.66276E-14	down	0	0	0	141	76	158
RENBP	-3.40012548	5.10771E-24	down	14	19	26	154	130	160
REPS2	2.965934986	8.14639E-13	up	1241	1460	1375	148	53	179
RERE	-2.007168379	1.56729E-16	down	811	875	814	2319	1697	3202
RERG	-10.9716702	2.30191E-18	down	0	2	0	1057	279	1585
RERGL	-8.064740624	8.51245E-26	down	1	5	0	573	250	325
RETN	-7.983381848	0.001735189	down	0	0	0	77	0	28
RETSAT	-2.260615878	0.020534583	down	1234	1347	1106	675	9484	1380
REV3L	-1.233891345	8.20674E-06	down	556	677	559	755	925	1301
REXO2	0.638938687	0.001050111	up	1901	2043	1764	888	922	773
RFC1	0.863465408	0.000155958	up	2286	2479	2295	842	719	1214
RFC2	2.250212918	2.33218E-21	up	1413	1528	1439	292	189	176
RFC3	2.950162913	1.25315E-59	up	1594	1838	1626	159	164	139
RFC4	3.148715827	3.19268E-57	up	1485	1732	1666	166	100	128
RFC5	2.796119933	1.12271E-52	up	1518	1600	1484	137	158	174
RFK	1.592332344	1.12584E-08	up	1447	1745	1471	416	407	263
RFNG	-1.22546711	2.2417E-10	down	234	255	302	459	392	468
RFTN1	-1.816069439	1.88072E-37	down	461	553	461	1255	1043	1398
RFTN2	-10.7155433	1.29447E-17	down	0	1	0	431	310	557
RFWD3	3.478563162	1.38403E-83	up	3624	3887	3512	225	188	293
RFX3	-1.789550288	1.85438E-06	down	106	76	89	159	206	303
RFX5	0.645498263	0.001035216	up	1466	1687	1676	798	546	870
RFX6	4.779659138	0.024097221	up	9	3	9	0	0	0
RFX8	-5.03843891	0.000427958	down	1	1	0	9	24	12
RFXAP	-0.711480471	0.011350615	down	104	98	71	110	93	115
RGAG4	-10.11313867	1.17602E-15	down	1	0	0	250	225	376
RGCC	-6.453223084	1.25619E-29	down	6	15	6	460	789	365
RGL1	-7.06059547	4.43859E-81	down	8	11	10	769	933	1028

RGL3	-1.654153567	3.95642E-06	down	100	132	150	263	193	409
RGMA	-7.506940953	6.38001E-39	down	2	3	3	361	274	405
RGMB	-0.695215757	0.023900676	down	318	319	316	270	413	401
RGN	-8.547187739	3.40065E-23	down	1	2	1	534	198	352
RGPD4	-3.745845621	2.58311E-05	down	0	5	3	26	19	31
RGS1	-10.42873031	1.5751E-12	down	0	0	0	270	216	48
RGS11	-1.904845433	1.46637E-08	down	143	135	109	430	201	418
RGS12	-2.513414495	1.57345E-29	down	73	97	86	328	353	350
RGS13	-5.833995281	0.002155757	down	0	0	0	11	3	9
RGS14	-2.052298124	1.28999E-06	down	24	30	53	118	98	101
RGS16	-4.396923012	1.65898E-09	down	3	6	9	87	131	44
RGS18	-3.827793755	8.78026E-07	down	6	2	7	56	22	79
RGS19	1.170504656	0.015861803	up	460	467	407	246	102	79
RGS20	3.587707156	2.33615E-05	up	81	112	82	0	6	10
RGS22	-2.497273067	7.16939E-05	down	17	7	6	40	45	35
RGS3	-2.029819721	4.67225E-22	down	639	647	638	2440	1470	1727
RGS4	-6.242929673	4.73932E-17	down	11	20	8	1509	361	262
RGS5	-9.895008032	3.90801E-45	down	106	92	62	80466	9706	93256
RGS6	-9.676740861	3.76143E-12	down	0	0	0	50	130	135
RGS7BP	-12.53474368	8.6536E-20	down	0	0	0	1388	270	753
RGS9BP	2.836412718	0.002243673	up	35	29	27	5	4	0
RHBDD1	-2.193682483	1.27581E-19	down	142	147	121	378	404	549
RHBDF1	-0.865938401	0.023452174	down	409	507	479	553	356	926
RHBDL1	2.87747814	1.53016E-16	up	180	176	196	24	11	19
RHBDL2	3.592573767	0.000972384	up	77	89	70	0	11	2
RHEB	1.021319909	1.84074E-05	up	3096	3399	2862	1486	844	973
RHEBL1	3.774992805	3.56073E-12	up	126	138	127	2	10	8
RHNO1	1.366089583	7.65187E-14	up	644	729	615	212	173	162
RHOB	-1.014655534	0.015125939	down	5839	6295	5584	14253	6119	5422
RHOBTB1	0.997439145	1.32344E-09	up	1578	1785	1541	641	446	670
RHOD	2.208961193	3.76373E-29	up	1444	1700	1427	217	192	295
RHOF	5.608034156	1.98035E-98	up	2667	2817	2761	39	50	30
RHOH	-3.880641405	0.009860548	down	0	0	5	29	14	11
RHOJ	-12.72161517	1.10068E-24	down	0	0	0	888	963	774
RHOQ	-2.901681529	1.29777E-20	down	810	984	792	3303	5647	4479
RHOT2	0.632640098	9.04536E-06	up	1486	1546	1621	804	600	741
RHOU	-1.499893632	4.35808E-05	down	580	755	697	1187	838	2114
RHOV	7.115870638	2.93661E-24	up	659	740	697	10	0	1
RHPN2	4.206754523	5.37563E-84	up	886	974	923	31	35	41
RIBC2	5.192340971	3.92399E-21	up	177	183	154	5	3	2
RIC3	-6.926193846	1.89293E-13	down	0	8	0	287	144	250
RICTOR	1.532591864	1.16929E-17	up	3525	3786	3667	839	756	1112
RILPL1	-1.259433161	1.03102E-17	down	264	282	235	468	390	472
RILPL2	-2.034232228	3.71562E-09	down	278	304	277	1214	465	875
RIMBP2	-7.957439667	1.39915E-05	down	0	0	0	81	7	14
RIMBP3	2.928070752	0.000163922	up	43	38	46	8	3	1
RIMBP3B	4.629611762	0.006835838	up	10	7	20	0	1	0
RIMS3	-3.408668087	5.46741E-07	down	56	64	61	656	92	674
RIN1	3.295778864	1.30664E-36	up	774	787	759	73	54	41
RIN2	-2.020376662	3.59928E-17	down	303	367	337	879	758	1281
RIN3	-2.47462064	1.15003E-20	down	102	106	84	456	262	449
RINT1	1.241683936	4.41754E-10	up	953	1048	908	345	282	245
RIOK1	0.865327285	1.18395E-08	up	842	840	769	365	264	332
RIPK1	-1.036158665	4.98425E-09	down	421	440	390	559	555	708
RIPK3	-9.179682928	2.7615E-12	down	0	0	0	91	48	92
RITA1	1.924508636	1.11979E-09	up	792	861	807	189	168	100
RLIM	1.327017006	1.17927E-25	up	3088	3401	3221	1054	754	957
RMDN1	-1.159780204	3.78925E-06	down	694	743	625	936	1256	1034
RMDN2	-2.855173418	4.15658E-15	down	30	36	19	181	103	156
RMI1	2.272575224	1.0092E-15	up	690	629	628	101	111	72
RMI2	4.029980435	9.54388E-14	up	481	600	543	36	27	7
RNASE1	-13.56652426	1.37437E-29	down	1	1	0	3851	2763	10962
RNASE2	-8.521607492	1.01673E-07	down	0	0	0	71	10	69
RNASE4	-4.330266816	5.2709E-35	down	114	118	109	2333	880	1774
RNASE6	-11.79466924	9.12523E-19	down	0	0	0	492	206	732
RNASEH2A	3.45747879	1.03265E-16	up	1144	1296	1096	125	64	41
RNASEH2B	-0.775868384	0.000289043	down	296	293	274	443	282	333
RNASEL	-3.330774474	2.48063E-33	down	54	34	41	332	290	304
RNASET2	-2.075433779	8.92026E-16	down	134	160	167	575	318	506
RND2	-5.491955965	8.71989E-14	down	0	6	4	135	60	128
RNF103	-1.376110764	4.39672E-14	down	523	535	486	960	751	1156
RNF103-CHMP	3.734120055	0.041432071	up	6	5	9	1	0	0
RNF11	-1.153719631	5.13409E-16	down	1597	1845	1606	2876	2119	3028
RNF112	-2.947208211	7.42169E-09	down	22	19	10	137	57	90
RNF113A	0.970102229	1.59151E-05	up	406	426	398	192	113	145
RNF114	0.717150231	4.22199E-07	up	2087	2273	2090	957	763	1081
RNF115	-1.479059133	0.000293715	down	1126	1078	1077	2961	988	2740
RNF125	-1.581704607	0.015218584	down	198	175	220	122	522	596
RNF13	-1.060954923	1.85077E-12	down	753	947	860	1312	1057	1436
RNF130	-2.377650186	2.33731E-62	down	472	525	512	1979	1517	2108
RNF135	-1.501508505	3.05408E-25	down	219	256	221	514	415	472
RNF139	-0.713711003	0.00241151	down	602	597	616	903	502	740
RNF14	-0.707597276	6.60652E-07	down	925	1067	864	1094	1070	1134
RNF141	-1.30252669	1.42108E-07	down	533	617	569	1132	667	1254
RNF144A	-10.56649531	4.386E-16	down	0	1	0	429	474	237
RNF144B	-1.713424342	9.07095E-10	down	175	181	139	304	361	484
RNF146	-2.936208799	3.13981E-29	down	234	287	244	1415	940	1850
RNF149	0.894239334	4.98625E-11	up	1741	2034	1743	679	642	785
RNF150	-2.088789796	1.88153E-22	down	269	318	284	1038	860	725
RNF152	-11.775129	8.33748E-21	down	0	0	0	462	526	370
RNF157	-2.566642315	0.006720382	down	69	83	85	46	692	191
RNF165	3.176922515	2.56521E-12	up	223	224	254	13	28	13
RNF166	-1.036308087	1.16305E-07	down	224	244	243	409	266	370
RNF168	0.613145437	0.003086133	up	991	962	954	459	345	559
RNF169	1.335416301	4.99736E-30	up	1479	1511	1462	448	378	430
RNF170	-0.738038263	3.96148E-06	down	316	381	301	420	334	430
RNF175	-5.703416929	0.002723067	down	0	0	0	4	9	7
RNF180	-11.6230455	1.1954E-19	down	0	0	0	586	244	432
RNF182	7.868978161	6.98E-118	up	2505	2838	2551	5	7	12
RNF185	-1.678424137	5.08537E-12	down	493	474	419	1129	728	1336
RNF187	0.777519601	1.09955E-07	up	2173	2307	2395	1065	887	901

RNF19A	-0.761075737	0.008847933	down	1309	1550	1400	1997	1043	2172
RNF19B	0.929741476	0.000218952	up	776	797	900	412	239	282
RNF2	1.008441527	0.001436316	up	717	664	596	257	269	165
RNF212	2.973400515	3.39126E-21	up	363	400	387	52	25	28
RNF213	0.66367672	0.000488638	up	4734	5014	4859	1922	1995	2635
RNF215	-0.978388737	0.000499177	down	119	97	91	179	120	134
RNF216	0.765371231	1.04805E-06	up	1644	1687	1556	659	586	804
RNF217	-1.375305766	4.13973E-06	down	433	433	418	759	523	1119
RNF219	0.615634298	0.000167872	up	610	640	536	275	243	311
RNF223	6.712270858	1.5726E-16	up	172	195	219	3	0	1
RNF224	8.232758161	1.86954E-10	up	77	82	75	0	0	0
RNF26	1.680900326	9.65449E-33	up	1742	1964	1776	398	349	470
RNF31	-0.90012708	2.29649E-07	down	314	307	261	446	340	387
RNF38	-2.670258601	6.82268E-26	down	277	258	243	1276	794	1501
RNF4	0.726229873	1.52687E-06	up	1575	1553	1434	637	645	672
RNF40	1.058918469	1.11076E-07	up	2550	2611	2522	742	854	1016
RNF43	3.796920536	1.12551E-05	up	25	55	57	4	2	1
RNF44	0.987236781	2.64736E-06	up	968	1027	960	303	375	373
RNF8	0.973874858	4.96036E-12	up	691	788	658	265	229	279
RNH1	1.490472851	0.026730636	up	58	38	24	8	13	9
RNLS	-5.697437447	5.96878E-15	down	4	5	2	85	204	101
RNPEP	1.503784655	1.58345E-32	up	2155	2317	2246	549	529	604
RNPEPL1	-1.225093957	2.72814E-06	down	489	475	462	734	924	681
RNPS1	1.337773686	3.59882E-21	up	3624	3607	3434	1174	818	1027
ROBO2	-2.674071482	0.000399433	down	10	14	4	48	17	64
ROBO3	-4.210336425	8.71989E-14	down	16	11	28	198	145	400
ROBO4	-9.85685333	1.56371E-22	down	2	0	1	351	924	654
ROCK1	-1.16247043	0.00254212	down	1822	1954	1902	2664	1736	4797
ROGDI	-1.195538519	6.03754E-06	down	193	153	191	309	306	258
ROM1	-3.645965012	2.24264E-12	down	22	28	32	417	116	217
ROPN1	-5.538605835	0.003931188	down	0	0	0	4	7	7
ROR1	-4.599819326	6.75689E-24	down	28	19	21	526	193	487
ROR2	2.567268973	7.90119E-07	up	503	543	514	40	102	38
RORA	-4.674384146	1.55507E-48	down	31	50	39	593	615	965
ROS1	6.347831317	1.85569E-25	up	252	279	270	3	2	2
RP2	0.901883675	8.99023E-08	up	692	767	691	261	235	323
RPA1	1.599446082	9.60243E-54	up	4453	4572	4300	1155	908	1072
RPA3	1.577291319	7.0694E-07	up	936	1060	828	312	207	153
RPAP1	1.201426945	5.67529E-07	up	832	812	823	234	290	231
RPAP3	2.024267888	2.01413E-52	up	2101	2348	1920	370	338	403
RPGRIP1	-4.354483321	0.005725655	down	2	0	0	14	5	11
RPGRIP1L	1.301247017	2.79673E-05	up	333	319	350	74	84	132
RPIA	0.802113505	0.024849521	up	616	677	599	263	322	172
RPL10	-0.923028017	0.000102872	down	11640	12480	11138	21647	12010	14335
RPL13	-0.727348729	0.027284721	down	12875	14478	12585	23833	11494	12209
RPL13A	-1.175736992	3.17045E-06	down	13492	14331	13696	30938	17798	18509
RPL15	-1.17141052	0.000473024	down	8368	9177	8287	21111	8504	12510
RPL18	-0.78023985	0.015787269	down	4881	5130	4539	8846	4671	4407
RPL23	-0.845597783	0.014823224	down	8838	9501	8497	17496	9074	8015
RPL23A	0.716268519	0.026121772	up	412	444	428	273	136	153
RPL26L1	1.047747364	0.001282451	up	678	780	576	291	245	157
RPL28	-1.415088	6.54425E-08	down	2681	2823	2787	7242	4465	4106
RPL3	-0.656033382	0.000842781	down	17444	18697	16833	25491	15843	18429
RPL32	-0.725606994	0.047903783	down	11018	11648	10519	20605	9505	9403
RPL34	-1.334687996	0.002332405	down	3127	3418	2880	9865	3588	3749
RPL36A	1.087279846	0.006783349	up	97	109	98	22	35	44
RPL37A	-1.213781037	5.19106E-05	down	9092	9766	8956	22386	12350	11491
RPL39	0.976585939	0.009365262	up	123	124	96	49	45	29
RPL39L	1.282785277	0.000202706	up	293	309	287	129	53	82
RPL7A	0.930146667	0.001050975	up	5179	5993	4923	2762	1764	1487
RPL7L1	0.632313225	2.24322E-06	up	2704	2917	2625	1213	1225	1329
RPLP1	-0.78137299	0.039703255	down	13244	14310	12915	26759	10859	12663
RPLP2	-0.90585329	0.005193589	down	5760	6368	5393	10778	7355	5175
RPN1	1.365121087	3.93472E-33	up	8045	8691	7688	2356	2165	2197
RPN2	1.529298437	5.09605E-33	up	9690	10791	9791	2522	2469	2426
RPP25	1.434735006	3.22239E-09	up	697	660	644	231	152	146
RPP40	1.712868454	0.017713797	up	303	309	273	41	119	22
RPRD1A	0.738762345	3.45134E-06	up	1960	2145	1939	800	867	892
RPS10	3.657107945	8.28498E-07	up	42	47	36	2	3	2
RPS11	-1.7508212	1.22278E-07	down	7272	8544	7631	29013	12159	15818
RPS15	-0.634974558	0.025863409	down	3492	3689	3410	5484	3269	3011
RPS19BP1	-0.866060721	5.22115E-05	down	592	687	608	1000	764	677
RPS2	0.988560226	0.000862153	up	29995	31925	29516	15584	9349	7999
RPS20	-0.847242414	0.006270662	down	12420	12882	12626	23738	13125	12075
RPS26	2.325489043	2.13431E-11	up	499	565	506	46	75	100
RPS28	-4.386547253	5.02184E-21	down	68	55	34	1280	438	668
RPS29	-0.744653706	0.032248332	down	2664	2505	2427	4374	2710	2008
RPS3	0.706435242	2.87116E-06	up	33470	35963	33506	17809	13391	13722
RPS3A	-1.008591759	0.004233408	down	8686	9999	8152	19940	9629	9180
RPS4Y1	-13.22369867	0.000370692	down	0	1	0	4956	0	2767
RPS6	-0.947531004	0.00011289	down	13327	14572	12490	24535	16338	14610
RPS6KA1	3.159430677	0.000162046	up	27	60	56	2	7	2
RPS6KA2	-11.03629696	4.23726E-28	down	1	2	0	704	1663	2023
RPS6KA3	1.542376137	3.40514E-20	up	5458	5743	5429	1204	1301	1541
RPS6KA4	1.292373501	6.72013E-09	up	1203	1335	1249	487	298	321
RPS6KA5	1.943979899	3.17106E-19	up	379	404	360	65	75	70
RPS6KA6	-9.737409362	1.21216E-10	down	1	0	0	422	39	221
RPS6KL1	-6.421375171	9.28312E-18	down	2	1	2	97	85	124
RPTOR	1.087384301	3.64776E-12	up	929	1000	963	385	269	318
RPUSD1	2.702056128	2.7468E-34	up	1037	1117	1162	126	132	102
RPUSD2	0.974216751	6.24622E-05	up	246	292	232	85	100	91
RPUSD3	1.451763585	5.66381E-15	up	680	683	670	176	184	166
RPUSD4	0.605571952	0.001447742	up	766	845	773	382	389	335
RRAD	-8.585631402	3.68782E-41	down	4	2	0	619	452	575
RRAGB	-0.944244187	5.20008E-06	down	326	286	292	459	318	472
RRAGD	-7.14560272	1.32529E-30	down	2	11	6	488	896	451
RRAS	-2.752216318	1.17027E-11	down	415	514	517	3228	1050	2832
RREB1	0.819429702	0.000238783	up	955	1071	1001	346	441	420
RRM1	2.593579728	7.7723E-125	up	5958	6576	5922	820	613	747
RRM2	5.477959296	3.41806E-13	up	3992	4371	3888	126	61	9
RRM2B	-0.77214875	2.93632E-06	down	679	707	591	752	762	878

RRP1	1.842418787	6.69654E-13	up	904	959	886	177	213	148
RRP12	2.481229387	9.55114E-12	up	1064	1175	965	135	178	86
RRP15	1.342962143	3.30465E-16	up	924	1034	889	253	270	270
RRP1B	2.539741401	3.23723E-59	up	2642	3057	2724	323	350	350
RRP36	0.796358018	0.000772158	up	836	937	790	357	388	292
RRP9	1.164315116	8.58309E-05	up	518	593	489	163	204	132
RRS1	1.002736547	0.000111692	up	802	748	679	252	303	227
RSAD1	-0.669069657	0.000131528	down	485	462	430	605	468	487
RSAD2	-5.01869455	4.58447E-10	down	8	11	13	568	97	99
RSBN1L	0.669216177	0.03530138	up	629	714	619	382	171	339
RSC1A1	0.737188322	0.019709627	up	162	198	151	58	82	75
RSL1D1	1.563296679	3.93864E-32	up	7466	8305	7186	1963	1802	1732
RSPH4A	-1.71644076	0.040151464	down	4	4	13	18	12	20
RSPO4	-5.856658765	0.008998902	down	0	0	0	1	11	10
RSRC1	0.771321593	0.000205741	up	429	534	551	217	173	243
RSRP1	-2.083269553	1.35132E-12	down	852	795	736	2976	1424	2921
RSU1	-2.201943468	1.0319E-83	down	879	986	941	3057	2825	3286
RTKN2	4.97708523	2.21445E-23	up	182	230	208	8	4	2
RTN1	-12.49648143	1.01323E-07	down	0	0	0	923	31	1425
RTN4	-0.670346514	2.76707E-05	down	5798	6082	5658	6279	6797	6633
RTN4IP1	2.097931385	9.04637E-15	up	318	345	298	52	62	44
RTN4R	5.992251407	6.81878E-30	up	291	337	269	2	3	5
RTN4RL1	-6.850233115	4.81213E-09	down	1	0	2	45	142	50
RTN4RL2	5.004337975	6.30363E-31	up	816	838	842	10	29	15
RTP4	-8.486620682	8.56512E-10	down	0	0	1	154	72	51
RUFY3	-2.475454618	3.1731E-57	down	242	269	247	1116	797	1101
RUFY4	-6.310806547	0.037647964	down	0	0	0	29	0	4
RUNDC1	1.093292004	4.48582E-10	up	669	671	573	210	211	214
RUNDC3B	-2.194770504	4.23259E-06	down	22	17	26	48	74	89
RUNX1T1	-11.67675379	2.61022E-20	down	0	0	0	340	366	581
RUNX2	-2.310651929	0.000101626	down	13	22	8	43	65	39
RUNX3	3.367480375	8.70907E-52	up	660	703	656	52	32	56
RUSC2	-1.089961829	0.003687338	down	699	778	728	1503	558	1355
RUVBL1	2.844624727	1.86862E-26	up	2601	2947	2472	197	301	284
RUVBL2	1.359794455	2.90967E-14	up	1800	2123	1781	653	449	481
RWDD1	-1.777998976	6.99337E-11	down	632	632	551	2097	1103	1274
RWDD2A	-0.99649019	0.006394781	down	153	170	150	296	122	266
RWDD2B	-0.86798425	0.000385981	down	334	354	284	553	320	398
RWDD4	-1.138962972	7.32754E-05	down	189	185	192	363	189	349
RXFP4	4.645859493	0.006001255	up	18	14	6	1	0	0
RXRG	-8.239082999	1.59247E-06	down	1	0	0	39	164	12
RYBP	0.821085832	0.005928566	up	195	256	181	83	62	110
RYR1	1.049092046	0.018732299	up	196	209	217	91	32	96
RYR3	-6.4205717	7.4329E-16	down	4	8	4	447	70	487
S100A11	0.854144999	5.63473E-07	up	6502	7168	6446	3258	2281	2395
S100A12	-6.685063604	0.000336385	down	0	0	0	25	3	14
S100A13	-0.724375482	0.01843727	down	1499	1537	1323	1850	1984	1215
S100A14	4.487168029	2.71112E-11	up	131	137	153	1	9	3
S100A16	1.987785037	0.000102867	up	5144	5516	5340	707	1535	513
S100A2	4.427531162	4.50584E-19	up	451	427	391	25	4	14
S100A3	1.321565642	0.000350516	up	112	125	149	48	35	27
S100A8	-9.860195711	2.15135E-14	down	0	0	0	154	119	91
S100A9	-7.832716049	3.44341E-30	down	2	1	5	331	571	371
S100P	10.98461397	3.27259E-19	up	949	1090	1032	0	0	1
S1PR1	-3.53191693	7.84609E-18	down	152	134	144	724	1615	1111
S1PR3	-6.304849365	2.27088E-25	down	30	10	26	969	632	2242
S1PR5	-6.489814569	0.000128657	down	0	0	0	16	13	6
SAA1	-6.013116443	2.70465E-09	down	11	27	22	145	2089	276
SAA2	-3.879885373	0.035470228	down	3	3	4	0	90	5
SAAL1	0.988790143	2.70125E-08	up	463	483	446	174	167	156
SAC3D1	2.278556319	1.28458E-10	up	538	539	533	117	70	50
SAE1	0.898904187	1.02095E-15	up	2567	2853	2361	1052	903	1007
SAFB	1.080785402	1.12725E-14	up	2781	2812	2642	959	759	1064
SALL2	-9.628489155	1.14389E-12	down	0	1	0	248	83	291
SALL4	5.950877389	2.74334E-32	up	336	406	383	9	2	2
SAMD1	1.207211973	4.84019E-16	up	551	597	570	185	153	192
SAMD10	2.205785728	1.59864E-08	up	148	165	168	18	33	22
SAMD11	1.851859615	0.001564894	up	537	634	670	221	100	45
SAMD13	-2.870687996	2.72268E-05	down	6	2	8	29	26	29
SAMD14	-6.30982235	1.64408E-12	down	1	0	3	100	70	57
SAMD15	3.988335428	4.68099E-05	up	26	34	30	2	2	0
SAMD3	-7.757098305	5.91858E-07	down	0	0	0	46	27	12
SAMD4A	-1.868197513	6.81457E-06	down	656	675	672	1614	884	2817
SAMD4B	1.001117572	6.66912E-10	up	2638	2954	2837	984	823	1199
SAMD9	1.511169274	3.50379E-22	up	1591	1703	1738	401	373	483
SAMD9L	-3.444144016	8.90144E-65	down	59	87	78	610	495	629
SAMHD1	-3.135129587	4.77578E-07	down	564	646	546	1713	1721	7742
SAMSN1	-8.800092924	6.3942E-12	down	0	1	0	118	82	144
SAP130	1.012542476	9.72812E-16	up	873	966	921	353	286	335
SAP18	-1.362564383	2.49552E-14	down	1411	1476	1381	3131	1940	2810
SAP25	-4.525293368	1.25686E-07	down	4	1	6	100	24	64
SAP30L	-1.937356975	3.57129E-19	down	362	324	315	916	700	1135
SAPCD2	5.97650411	1.6176E-47	up	1390	1442	1467	29	12	8
SARAF	-0.986715456	4.29769E-13	down	2857	3021	2545	4026	3345	4524
SARDH	-2.565048899	0.000947572	down	7	10	12	21	68	28
SARM1	-1.056232016	8.35106E-08	down	164	213	187	293	225	318
SART1	0.668382832	0.00107551	up	1881	2022	1821	1087	636	862
SART3	0.770058444	1.03475E-05	up	1471	1550	1429	701	465	705
SASH1	-3.084233621	1.78098E-49	down	339	417	368	2004	1933	2841
SASH3	-10.6685038	6.33599E-17	down	0	0	0	275	135	239
SASS6	2.959244666	2.93758E-37	up	889	1006	818	95	55	100
SAT1	-1.126218627	0.000105197	down	3319	3419	3294	4517	6417	4352
SAT2	-0.756344489	0.001064011	down	493	565	501	775	588	506
SATB1	-2.898945813	5.72724E-22	down	127	117	152	545	651	908
SATB2	-1.58061054	0.003682445	down	82	60	89	203	65	241
SAXO2	-2.137117253	0.001470145	down	7	6	5	23	12	22
SAYSD1	-0.611720866	0.012332235	down	219	225	231	289	173	280
SBF2	0.693983893	0.015586156	up	1831	2048	1925	672	706	1179
SBK2	5.845545918	0.000299307	up	15	19	11	0	0	0
SBK3	3.702725035	0.000161655	up	96	76	78	0	10	3
SBNO1	1.565334811	6.35886E-35	up	2681	2965	2649	663	653	668

SBNO2	1.90030867	3.96026E-06	up	123	97	164	25	20	29
SBSPON	-8.451889108	2.64138E-23	down	1	3	2	794	166	578
SCAF8	-0.677710278	2.95905E-07	down	717	767	701	877	690	927
SCAI	1.061577616	3.63938E-07	up	532	542	533	162	163	223
SCAMP3	2.627329222	0.018375057	up	11	50	22	1	6	2
SCAMP4	-0.601282507	0.005710837	down	367	396	391	385	448	401
SCAMP5	-3.863721942	8.13282E-13	down	20	21	17	163	300	117
SCAND1	-0.752355831	0.014342093	down	394	458	426	719	436	382
SCAPER	-0.880707075	1.08343E-05	down	321	365	320	388	408	517
SCARA5	-2.209747348	0.000533437	down	1132	1088	1145	2473	1231	7716
SCARB1	3.114421025	1.94832E-19	up	4004	4435	4089	208	403	400
SCARB2	-0.769812272	1.34023E-08	down	3618	4120	3618	4337	4344	5032
SCARF1	-4.645854017	0.008019763	down	1	0	0	8	7	4
SCARF2	-4.781646968	1.07218E-49	down	30	21	28	630	357	584
SCCPDH	2.146190646	0.000517006	up	5410	5889	5142	441	1585	475
SCD	-3.342831657	0.039494371	down	20757	22385	20606	2762	363787	53359
SCD5	-6.881098926	3.42234E-41	down	3	2	6	346	293	283
SCFD1	-0.805410825	5.58496E-06	down	651	721	602	1002	683	776
SCG2	-4.816127777	1.76791E-09	down	7	4	6	44	194	89
SCGB3A2	-5.14981414	0.010433349	down	0	1	0	4	2	22
SCGN	-7.155978124	1.12146E-05	down	0	0	0	30	18	8
SCIMP	-8.630643948	1.94951E-09	down	0	0	0	74	21	65
SCIN	2.986295295	1.61421E-24	up	403	504	455	39	27	57
SCLT1	1.033840646	0.000481064	up	408	434	376	202	104	121
SCMH1	-1.483159727	9.74438E-11	down	385	361	415	809	570	957
SCML1	2.053433637	9.55975E-11	up	2655	2873	2478	299	478	580
SCML2	3.708860948	2.50143E-16	up	927	947	860	25	40	84
SCML4	-7.853660777	4.17845E-07	down	0	0	0	12	37	40
SCN11A	-7.314132061	6.68159E-06	down	0	0	0	31	23	8
SCN1B	-1.086146168	1.25972E-07	down	142	177	148	253	232	216
SCN2A	-5.567504721	0.008823365	down	1	0	0	1	5	31
SCN2B	-6.437035919	4.10648E-06	down	1	2	0	24	128	18
SCN3A	-9.421711437	8.81239E-12	down	0	1	0	76	224	212
SCN3B	-4.226245911	0.007351734	down	0	0	7	16	55	20
SCN4A	-3.482817233	2.84627E-05	down	2	7	11	23	64	69
SCN4B	-7.02521811	4.9501E-23	down	5	3	4	221	595	255
SCN7A	-10.78418234	9.30687E-14	down	1	0	0	191	831	252
SCN9A	-4.742211074	5.27722E-05	down	0	5	8	26	43	182
SCNN1A	3.276653524	1.09606E-14	up	118	107	128	9	8	9
SCNN1B	-7.535154479	2.21066E-07	down	0	0	0	18	20	35
SCOC	-1.304404513	4.39499E-14	down	685	868	720	1352	1356	1248
SCP2	-1.15760658	4.4198E-11	down	1610	1733	1489	2397	2677	2521
SCPEP1	-1.538055129	6.87449E-17	down	871	949	782	1840	1385	2178
SCRG1	-12.96214163	5.19624E-11	down	0	0	0	1653	115	1512
SCRN1	0.855906441	0.017640072	up	4048	4309	4026	1996	840	2138
SCUBE2	-4.460010781	1.22883E-09	down	8	9	5	42	170	119
SCUBE3	-10.70162063	5.09694E-22	down	1	4	0	3856	300	1920
SCYL1	0.96538273	1.16182E-12	up	2292	2539	2434	866	772	1011
SCYL3	-1.009901163	0.000104704	down	177	137	143	201	222	230
SDC1	7.843854533	9.24E-148	up	4346	4576	4221	21	6	14
SDC2	-3.717964859	9.1947E-34	down	350	465	422	5143	2247	4382
SDC3	-1.003409786	4.99475E-06	down	955	1089	1018	1920	1110	1373
SDC4	1.817762698	7.56327E-15	up	4683	5173	4552	1296	674	971
SDCCAG3	2.656101542	2.61697E-64	up	2673	2791	2652	310	314	287
SDCCAG8	-2.969019403	0.002100635	down	11	6	11	9	52	95
SDE2	0.898349064	3.98181E-08	up	734	764	656	311	254	256
SDF2L1	2.169876043	4.27974E-17	up	766	864	694	169	97	103
SDHA	1.737183423	0.00022217	up	8590	9255	8683	1171	2909	1367
SDHAF4	-1.090366924	0.001820141	down	71	69	65	91	125	90
SDHB	0.900069561	0.010585152	up	2564	2890	2593	844	1353	793
SDK2	-5.852683004	2.89952E-14	down	2	1	3	85	51	115
SDPR	-5.420907343	5.1018E-18	down	43	44	25	418	1843	990
SDR16C5	8.894483867	3.21482E-12	up	106	128	136	0	0	0
SDR39U1	-1.476674992	9.58379E-26	down	294	303	263	622	492	591
SDR42E1	-8.222426554	7.86317E-07	down	0	0	0	53	7	62
SDSL	-3.311968159	3.86415E-07	down	16	20	16	184	137	42
SEC13	1.162698487	9.95036E-15	up	2655	2980	2653	1052	764	821
SEC14L4	7.015602311	5.73195E-22	up	300	320	299	0	5	0
SEC14L5	-2.085733277	0.029134162	down	4	2	10	19	8	23
SEC14L6	-6.960831118	0.016447281	down	0	0	0	46	5	0
SEC16A	1.566906626	7.35841E-36	up	4232	4602	4492	1049	1031	1109
SEC16B	-6.153426321	7.15983E-35	down	6	3	5	249	172	296
SEC22B	0.608100291	5.83207E-07	up	2696	2934	2726	1393	1073	1448
SEC22C	-0.757985499	4.59298E-06	down	817	888	787	1035	793	1184
SEC23B	1.081005604	6.98263E-16	up	1361	1412	1318	530	389	461
SEC24A	1.731332246	3.97127E-25	up	2106	2246	2126	433	398	559
SEC24B	-0.811572123	2.96935E-05	down	602	722	595	832	609	966
SEC31B	-1.947286139	1.43935E-12	down	82	96	106	276	186	325
SEC61A1	1.296456397	9.10111E-18	up	8762	9107	8462	2706	2552	2328
SEC61A2	0.831320144	7.18838E-05	up	209	253	233	106	80	92
SEC61G	1.366122552	2.92627E-06	up	2478	3065	2353	1040	583	564
SEC62	-1.390629245	7.54765E-06	down	2395	2670	2222	6631	2814	4364
SECISBP2	-0.609537806	6.79627E-05	down	614	662	611	671	587	793
SECISBP2L	-1.272498097	1.19285E-09	down	892	991	885	1446	1290	2035
SECTM1	-1.586800786	8.00246E-08	down	55	70	40	126	104	121
SEH1L	0.697724198	0.000729059	up	890	957	909	442	418	341
SEL1L	-0.722496363	0.004953396	down	1844	2152	1987	1923	1980	3124
SEL1L2	-6.593674291	0.007705606	down	0	0	0	37	1	2
SEL1L3	2.116840867	2.15811E-12	up	1197	1335	1180	188	254	155
SELE	-9.633522513	7.04831E-06	down	0	2	0	803	288	13
SELENBP1	-1.745365148	9.33981E-50	down	752	845	785	2100	1582	2017
SELK	-1.090020528	0.00113513	down	502	556	490	1194	551	628
SELL	-9.049089556	3.6158E-12	down	0	0	0	70	76	60
SELM	-3.058531631	4.28539E-09	down	477	543	463	4879	976	3225
SELP	-12.20518244	2.20062E-21	down	0	0	0	442	795	575
SELPLG	-2.987780301	7.12757E-19	down	37	28	33	170	144	244
SEMA3A	1.887063542	5.23316E-08	up	219	229	219	28	48	51
SEMA3B	1.150009173	0.020731508	up	2409	2804	2749	1365	325	942
SEMA3D	-6.546254283	1.72028E-13	down	3	14	8	952	80	679
SEMA3E	-4.811345346	0.001787052	down	1	2	0	10	4	47
SEMA3F	1.335308072	7.64416E-21	up	1365	1524	1520	405	380	457

SEMA3G	-5.312440956	2.05117E-30	down	31	65	26	767	1440	1116
SEMA4A	-2.76215794	7.59396E-05	down	11	11	8	77	17	55
SEMA4B	2.466797213	2.48941E-13	up	1940	1999	1889	165	313	257
SEMA4C	-1.462147607	1.76545E-24	down	313	342	326	661	545	721
SEMA4G	-5.289956982	4.33816E-07	down	2	1	0	25	28	30
SEMA5B	-2.628953298	0.000361787	down	12	20	22	42	138	46
SEMA6A	-0.769497306	0.047309131	down	197	230	198	156	283	307
SEMA6B	-2.561173825	1.37791E-08	down	82	96	82	280	531	244
SEMA6C	-3.282411043	4.02457E-10	down	44	38	36	168	421	199
SEMA6D	-2.64128371	1.13633E-22	down	104	109	80	460	306	545
SEMA7A	2.40888966	5.28914E-07	up	489	535	539	88	88	30
SENP1	1.19368311	6.75588E-14	up	890	914	809	277	224	314
SENP6	-1.774842576	3.36436E-10	down	724	759	749	2327	1098	2107
SENP7	-2.121065766	2.61465E-18	down	234	297	240	873	546	987
SENP8	-1.8324437	1.4338E-05	down	29	22	24	54	75	59
SEPP1	-2.617692443	5.80141E-18	down	1795	1880	1861	6054	6507	11711
SEPSECS	-1.527563137	1.4903E-10	down	127	190	151	288	299	366
SEPT1	-3.472694329	1.25533E-08	down	8	13	9	132	61	45
SEPT10	0.761134866	0.000975792	up	4410	4809	4362	1861	1390	2496
SEPT12	4.891561255	0.010356625	up	7	7	9	0	0	0
SEPT2	-0.777424023	5.2545E-06	down	6939	7759	6639	9970	6505	9733
SEPT3	5.982846359	1.64729E-34	up	375	344	348	3	5	4
SEPT4	-6.369444305	1.89435E-55	down	7	19	13	638	870	738
SEPT6	1.005522406	0.000107666	up	938	1209	1078	297	403	429
SEPT8	-1.168873048	5.69392E-09	down	987	1135	947	1844	1175	1930
SEPT9	1.151536434	6.20868E-24	up	4992	5424	5419	1722	1501	1853
SEPW1	-2.052151864	2.25386E-15	down	1337	1463	1367	5799	3116	3496
SERAC1	-1.654081343	0.000285066	down	114	77	89	230	106	308
SERBP1	1.048297178	4.13332E-18	up	11449	12642	11060	4058	3449	4597
SERF2	-1.104222009	7.61039E-10	down	1099	1266	1197	2263	1518	1688
SERGEF	-1.018157154	0.018688139	down	245	280	294	549	181	481
SERHL2	-7.953344297	2.91748E-08	down	0	0	0	51	20	28
SERINC1	-2.650446489	6.44509E-30	down	1757	1855	1747	8658	5380	10204
SERINC2	3.803056944	0.000108026	up	1198	1344	1312	23	150	8
SERINC3	-1.225189384	2.17256E-09	down	2629	2763	2597	4660	3215	5541
SERINC5	1.881253487	2.83791E-07	up	1774	1888	1771	220	451	354
SERP2	-8.705719225	2.60783E-11	down	0	0	0	57	56	50
SERPINA1	-2.519049374	0.001994127	down	17	4	9	73	22	32
SERPINA3	-10.28367444	2.08641E-82	down	0	7	2	2692	2616	2601
SERPINA5	-7.472913513	3.42781E-10	down	1	0	1	66	58	132
SERPINB5	7.870892909	8.56755E-10	up	97	129	129	0	1	0
SERPINB6	-0.757972941	8.4613E-06	down	1816	2027	1841	2621	2193	2000
SERPINB7	6.395815611	3.06376E-05	up	23	29	14	0	0	0
SERPINB8	-2.024972484	4.80782E-08	down	112	92	70	306	158	343
SERPINB9	-10.82908412	1.27989E-17	down	0	0	0	327	181	213
SERPIND1	-4.673198175	0.001738631	down	2	1	0	3	24	26
SERPINE1	0.932339365	0.00277581	up	8918	10030	9097	4581	1995	4073
SERPINF1	-13.16866186	2.2558E-38	down	0	0	3	3387	9268	6494
SERPING1	-10.40647314	2.3203E-141	down	11	15	11	10237	8142	17624
SERPINI1	-0.894224617	0.002698287	down	214	302	257	472	250	307
SERTAD1	-1.220852571	0.041951713	down	194	172	170	559	219	125
SESN1	-3.680962408	2.49887E-12	down	152	170	136	908	688	2657
SESN3	-3.831596927	2.0471E-119	down	186	205	169	1777	1809	2057
SESTD1	-2.504800435	1.59312E-10	down	501	510	536	1988	1099	3294
SET	2.418566682	2.9559E-149	up	23674	25592	22539	3533	2784	3246
SETBP1	-1.255201779	1.68184E-06	down	298	352	299	448	447	717
SETD1A	1.129048168	1.23047E-06	up	895	923	912	239	291	354
SETD7	-1.363822996	2.51995E-05	down	1830	2088	1791	3477	2083	5045
SETDB2	-1.397907452	1.46752E-05	down	55	65	55	88	99	140
SETMAR	-2.364007461	3.43342E-09	down	156	159	144	785	267	671
SETSIP	4.159263258	1.8074E-08	up	72	63	67	4	4	0
SEZ6L	-4.20722035	0.024404789	down	1	0	0	3	5	6
SEZ6L2	4.023491597	1.13893E-10	up	600	695	641	22	50	9
SF3A3	0.99868096	7.72393E-15	up	2748	3005	2616	1151	829	1008
SF3B1	-0.767434314	0.005917945	down	6712	7502	6344	8594	5379	11224
SF3B3	1.954053685	1.9918E-15	up	5849	6244	5622	850	1213	1145
SF3B4	1.637580979	1.67622E-29	up	2019	2321	2068	513	392	565
SF3B5	-1.163697464	2.34988E-05	down	734	694	747	1589	992	909
SFMBT1	1.629788234	3.09813E-18	up	789	842	723	177	190	170
SFMBT2	2.655019465	9.98745E-42	up	1945	2127	2146	206	248	243
SFN	6.916267469	4.2591E-44	up	1884	1838	1753	22	3	8
SFPQ	0.652563938	1.06044E-05	up	8657	9362	8398	4165	3183	4665
SFR1	1.201762108	4.61538E-07	up	458	521	454	126	164	148
SFRP1	-15.19833854	2.24028E-25	down	0	0	0	8951	877	5591
SFRP2	-14.79418588	6.66154E-08	down	0	0	0	2978	43	8667
SFRP4	-13.1067464	6.84596E-27	down	0	0	0	1271	966	1240
SFRP5	-9.03198871	1.92639E-08	down	0	0	0	21	128	45
SFTPD	-5.305742714	0.015016516	down	0	0	0	10	2	4
SFXN1	2.633264097	7.52636E-24	up	2740	2913	2596	319	370	243
SFXN3	0.589157049	0.021065809	up	2669	2731	2652	1395	845	1620
SGCA	-8.837311499	7.61032E-34	down	0	4	5	1206	430	1359
SGCB	-4.194321449	6.21627E-41	down	184	192	150	2372	1390	3186
SGCD	-13.28799328	6.084E-25	down	0	0	0	788	1311	1797
SGCE	-1.568094755	5.31129E-10	down	679	636	662	1841	948	1447
SGCG	-10.61020159	5.07789E-16	down	0	0	0	235	242	128
SGF29	-1.011546113	0.004384297	down	140	137	144	273	116	227
SGIP1	-10.63809409	2.67335E-17	down	2	0	0	1332	225	807
SGMS2	1.120595506	7.42219E-05	up	1054	1154	978	287	272	487
SGO1	5.891084999	7.86215E-26	up	664	741	687	15	9	1
SGO2	3.213198909	1.44251E-67	up	933	993	888	75	74	66
SGPP1	0.649573916	0.005188287	up	820	926	799	477	364	310
SGPP2	-5.913121058	0.01317519	down	0	0	0	1	17	4
SGSM2	-0.6387591	0.006754328	down	618	665	692	699	560	945
SH2B1	-1.00911545	1.53566E-09	down	590	639	575	939	665	995
SH2B2	1.401113803	9.27375E-08	up	157	200	186	57	45	44
SH2B3	-1.506795553	1.785E-10	down	372	452	440	874	936	716
SH2D1A	-7.639411006	1.14886E-06	down	0	0	0	46	21	12
SH2D1B	1.844087493	0.006668225	up	35	33	18	6	5	6
SH2D2A	4.109479356	1.42799E-17	up	351	317	350	9	23	9
SH2D3A	5.531375519	9.63432E-36	up	323	352	299	5	3	7
SH2D3C	-4.281553173	1.08245E-15	down	13	31	33	301	497	233

SH2D4A	3.050650318	9.83923E-18	up	1057	1198	1136	75	133	76
SH2D5	6.976513995	2.55541E-18	up	239	268	202	3	1	0
SH2D6	3.491013292	2.29711E-05	up	45	49	46	7	1	1
SH3BGR	-5.379021832	7.38446E-05	down	0	2	0	24	21	13
SH3BGRL	-5.370531183	6.96616E-35	down	161	198	186	7561	2178	6748
SH3BGRL2	-3.845055465	3.2689E-16	down	76	112	133	622	886	1781
SH3BGRL3	-0.697684915	0.002846551	down	1297	1369	1253	2022	1139	1403
SH3BP1	-1.741507678	0.000538911	down	41	38	43	164	55	78
SH3BP2	0.657327155	0.032702742	up	1356	1471	1564	471	715	774
SH3BP4	0.86737505	0.006957662	up	1067	1263	1085	306	420	597
SH3D19	-1.83625389	2.29079E-09	down	1185	1158	1000	2088	2397	4017
SH3GL1	1.468967807	3.59864E-40	up	2950	3151	3058	797	737	816
SH3GL2	3.569627094	3.0901E-07	up	182	181	204	12	19	2
SH3GL3	-7.632089558	0.000172364	down	0	0	0	65	2	15
SH3GLB1	-1.405222672	1.06998E-09	down	1447	1655	1460	2440	3228	2800
SH3GLB2	0.739116024	0.037955088	up	1906	1994	1949	1183	452	913
SH3PXD2A	-1.031990877	1.03386E-07	down	1745	1852	1704	2325	2183	3217
SH3PXD2B	-0.861051004	1.16733E-05	down	534	601	585	650	700	865
SH3RF1	-1.156648311	2.79493E-11	down	201	220	192	362	260	354
SH3TC2	1.448269113	0.017186102	up	209	229	199	50	88	22
SH3YL1	-2.008317256	3.93438E-12	down	129	132	130	526	286	318
SHANK3	-2.345177495	0.007240124	down	7	16	12	11	63	47
SHARPIN	-0.969877082	5.33258E-07	down	347	363	352	615	398	476
SHC2	-3.296404737	5.79283E-13	down	36	16	36	275	138	218
SHC4	-3.926313375	2.2597E-10	down	20	30	20	246	84	445
SHCBP1	5.294483812	9.19549E-26	up	2671	2862	2606	97	25	29
SHD	3.925811717	0.000527412	up	20	24	21	1	2	0
SHE	-11.39082036	4.95175E-19	down	0	1	0	492	850	670
SHF	-4.53939589	7.44312E-21	down	10	10	8	225	93	153
SHFM1	0.63416079	0.028600832	up	2761	3022	2757	1832	1109	990
SHH	8.231475141	2.62176E-10	up	85	72	76	0	0	0
SHISA3	-6.161681852	3.81131E-15	down	7	14	5	349	115	886
SHISA4	-3.37973078	1.38198E-38	down	68	70	90	682	416	611
SHISA6	-7.45312851	3.7166E-05	down	0	0	0	4	30	33
SHKBP1	0.925375241	6.55704E-06	up	1032	1094	974	442	276	454
SHMT2	1.81010955	2.74797E-11	up	3303	3828	3254	777	791	510
SHPRH	-0.638610626	0.003911813	down	489	593	548	531	523	750
SHROOM2	-4.387855712	7.62237E-07	down	7	1	1	34	55	44
SHROOM4	-12.25139663	1.52524E-21	down	0	0	0	404	680	809
SHTN1	1.803063289	2.7907E-07	up	2354	2607	2373	483	283	754
SIAE	-0.876306456	0.048870143	down	472	514	503	478	943	462
SIAH2	0.673105282	1.7762E-05	up	957	978	899	497	369	401
SIDT1	-8.303515671	5.53992E-10	down	0	0	0	36	37	51
SIDT2	-1.615477631	7.67655E-11	down	383	467	435	736	972	1064
SIGIRR	-1.021000472	0.010717244	down	125	202	150	339	192	157
SIGLEC1	-8.602132331	2.727E-57	down	5	3	2	838	753	1184
SIGLEC10	-9.098499107	2.57646E-10	down	0	0	0	110	26	86
SIGLEC11	-6.726520681	2.61401E-05	down	1	0	0	47	7	30
SIGLEC12	-6.368436272	0.000137662	down	0	0	0	12	6	15
SIGLEC14	-8.123798321	0.001540026	down	0	0	0	0	26	84
SIGLEC16	-6.205950631	0.002128233	down	0	0	0	9	2	19
SIGLEC5	-6.13359141	1.89823E-05	down	1	0	0	19	14	21
SIGLEC6	-6.954029053	2.10466E-05	down	0	0	0	27	7	16
SIGLEC7	-6.690667864	2.10979E-06	down	0	1	0	20	23	36
SIGLEC8	-6.612127791	3.34854E-05	down	0	0	1	18	8	13
SIGLEC9	-6.645799204	2.4593E-11	down	2	0	1	89	43	86
SIGMAR1	1.321330008	8.33817E-25	up	2417	2636	2538	811	663	686
SIK1	5.505140753	1.09402E-06	up	476	556	566	0	21	2
SIM2	9.944200729	1.10913E-15	up	480	470	538	0	1	0
SIMC1	-1.758264617	3.3721E-07	down	61	46	38	120	88	144
SIPA1	0.602817848	0.000206748	up	855	933	1001	489	368	455
SIPA1L1	-3.703117184	5.07518E-24	down	71	67	64	528	411	957
SIPA1L2	-1.615068632	0.004142617	down	291	265	304	383	1041	374
SIPA1L3	1.9879905	5.93652E-35	up	827	854	807	142	136	168
SIRPA	-5.165837548	5.8909E-117	down	31	43	34	905	799	1051
SIRPB2	-5.003507663	7.52911E-05	down	0	5	6	40	16	202
SIRT1	0.606426675	0.001906649	up	1400	1342	1211	684	463	716
SIRT3	-1.171285432	2.99142E-05	down	201	228	219	297	407	318
SIRT5	-1.24629399	4.22391E-05	down	182	197	162	233	353	312
SIRT6	0.972186928	1.23328E-06	up	333	371	379	153	119	121
SIRT7	1.116153231	1.70164E-08	up	601	580	651	217	158	231
SIT1	-7.211566235	1.29934E-06	down	0	0	0	20	12	27
SIVA1	0.879268446	0.008443943	up	1371	1650	1420	837	340	569
SIX2	0.764865249	0.018334839	up	182	226	224	114	55	99
SIX4	1.804421165	0.000197871	up	342	392	346	75	30	120
SKA1	6.432808798	0.000210791	up	39	9	18	0	0	0
SKA2	0.888575917	1.10864E-06	up	1631	1785	1529	765	475	674
SKA3	5.494514097	7.59609E-13	up	867	890	808	34	3	5
SKAP1	-7.795753901	9.5338E-08	down	0	0	0	46	23	19
SKAP2	1.06858254	6.11312E-10	up	949	1117	990	383	263	396
SKI	-0.770179965	0.003929277	down	1254	1238	1273	1733	986	1922
SKIDA1	2.597043844	7.37483E-07	up	254	223	307	15	46	29
SKIV2L2	0.783804227	2.66575E-06	up	2437	2605	2210	1014	801	1193
SKP2	3.148014806	2.0677E-56	up	3164	3390	2797	226	210	315
SLA	-8.169209578	2.34539E-23	down	2	2	1	294	191	555
SLA2	-6.30522998	2.71884E-05	down	1	0	0	18	28	13
SLAIN1	-9.626264064	2.87985E-10	down	0	0	0	172	22	129
SLAIN2	-1.142096326	1.96819E-10	down	778	859	726	1280	958	1491
SLAMF1	-6.139765888	0.000879027	down	0	0	0	10	13	4
SLAMF6	-8.26155051	4.28187E-08	down	0	0	0	30	19	74
SLAMF7	-7.864513837	7.64462E-09	down	0	0	0	30	31	30
SLAMF8	-9.139206386	6.26607E-12	down	0	0	1	97	113	223
SLBP	1.104739851	9.30089E-06	up	1789	1910	1683	819	442	536
SLC10A4	-5.238883603	0.032466385	down	0	2	0	9	0	45
SLC10A6	-4.04941439	0.001099642	down	2	0	5	9	24	51
SLC11A1	-3.199525364	2.37307E-16	down	26	17	23	166	95	178
SLC11A2	0.839190575	7.45681E-05	up	1834	2040	1677	729	557	933
SLC12A4	-0.687572837	0.001235227	down	1051	1152	1126	1250	973	1618
SLC12A6	-1.034224672	0.013799451	down	697	759	698	964	548	1687
SLC12A7	1.0553078	0.046921651	up	779	1026	786	157	460	232
SLC12A8	3.159067733	6.85596E-15	up	117	114	107	10	7	10

SLC13A3	-6.933096669	3.35559E-07	down	1	0	0	39	25	30
SLC13A4	-6.116648712	0.000307643	down	0	0	0	10	10	7
SLC14A1	-9.230703298	4.54994E-06	down	0	0	5	1694	88	522
SLC15A2	-2.719859136	4.88398E-06	down	18	32	23	155	41	154
SLC15A3	-8.641943182	1.88137E-29	down	2	1	1	411	326	402
SLC16A1	2.611083999	0.009540498	up	269	59	31	8	22	11
SLC16A10	-6.139638827	1.28533E-06	down	1	0	1	50	17	36
SLC16A11	-6.132237133	0.000570473	down	0	0	0	8	5	15
SLC16A12	-4.258841949	2.89422E-09	down	5	7	6	33	84	125
SLC16A13	2.813273362	1.20837E-08	up	173	161	157	7	21	21
SLC16A14	-4.560290753	1.83008E-22	down	10	9	12	187	206	121
SLC16A2	-8.307923209	2.38864E-28	down	1	2	4	337	754	440
SLC16A3	3.695126342	3.28656E-21	up	5163	5653	5399	377	347	155
SLC16A4	-6.310754339	3.26794E-24	down	8	12	3	595	180	547
SLC16A5	1.254021661	0.000416433	up	407	451	395	130	155	82
SLC16A6	7.628214901	5.4329E-126	up	2866	3153	2909	14	13	5
SLC16A8	-7.616450893	9.83557E-06	down	0	0	0	56	14	9
SLC17A7	-1.546456439	0.037313288	down	24	23	26	28	24	103
SLC17A9	1.714138447	0.000321982	up	480	542	488	177	99	52
SLC18A2	-6.02118704	1.65671E-05	down	0	2	0	56	17	20
SLC19A1	2.122388838	0.000293154	up	597	660	575	101	153	35
SLC19A2	1.009055227	2.28001E-05	up	1032	1056	886	303	391	344
SLC19A3	-4.038709153	0.00031934	down	39	46	47	21	1102	306
SLC1A1	0.815024758	0.037073713	up	237	185	229	64	109	87
SLC1A2	-2.868760824	0.001846059	down	2	7	4	9	24	33
SLC1A3	-9.41839616	4.52848E-22	down	2	2	0	257	943	672
SLC1A5	1.699019941	0.000105295	up	3088	3268	3138	470	1032	508
SLC1A7	-2.267402519	0.001672159	down	16	25	24	108	19	103
SLC20A1	2.279814588	2.4762E-09	up	3687	4000	3544	681	641	300
SLC20A2	-2.625247012	1.35415E-22	down	195	201	188	1133	558	911
SLC22A1	2.086596092	0.032568005	up	13	14	20	2	1	5
SLC22A13	2.438525776	0.040307724	up	15	9	13	2	0	3
SLC22A16	-6.266373359	0.004482579	down	0	0	0	11	17	1
SLC22A17	-9.930333655	6.50394E-21	down	0	3	0	1068	257	796
SLC22A18	2.204673837	3.63456E-10	up	149	163	191	22	31	24
SLC22A18AS	2.48837839	1.80949E-05	up	51	39	43	6	4	7
SLC22A23	-3.681577257	1.04644E-09	down	29	31	15	173	358	124
SLC22A3	-2.762732426	9.96982E-06	down	537	666	565	5179	699	2981
SLC22A31	1.861690623	0.027409718	up	18	21	26	5	1	7
SLC22A4	2.570389683	0.00080422	up	119	135	125	6	30	7
SLC23A3	-2.757943153	0.00226054	down	6	7	3	32	7	40
SLC24A1	-3.087946031	1.04932E-22	down	21	33	35	164	170	202
SLC24A3	-12.00508112	2.66213E-21	down	0	0	0	686	326	630
SLC24A4	-8.80630691	5.47514E-08	down	0	0	1	67	20	268
SLC25A12	-1.475588933	2.73641E-13	down	423	499	456	805	801	1117
SLC25A13	1.820749753	1.48344E-06	up	1472	1635	1419	196	407	284
SLC25A15	2.514273342	2.29954E-07	up	520	618	498	41	106	49
SLC25A17	0.78423653	6.5175E-07	up	562	595	500	228	211	244
SLC25A18	-7.384812124	6.7766E-05	down	0	1	0	5	95	18
SLC25A19	2.286612835	1.38572E-11	up	528	622	550	80	106	57
SLC25A22	1.795368913	0.023317704	up	1001	1080	945	79	421	79
SLC25A24	2.121434058	8.15789E-25	up	3292	3684	3086	458	515	664
SLC25A25	1.061484896	0.003147667	up	875	934	959	431	316	194
SLC25A27	-4.554738095	9.50167E-42	down	27	32	23	485	300	600
SLC25A28	-1.309471175	1.22307E-23	down	344	412	366	679	595	701
SLC25A30	-0.663667869	0.002774058	down	473	511	458	453	534	628
SLC25A34	-3.656296124	4.09571E-08	down	13	17	18	108	239	65
SLC25A37	0.891449974	0.003249205	up	2282	2367	2306	840	587	1275
SLC25A38	-0.852885227	6.0779E-09	down	407	464	397	624	458	550
SLC25A39	1.667578139	2.56623E-12	up	3429	3749	3600	949	834	615
SLC25A4	-3.160482604	2.0196E-12	down	459	489	444	2348	4443	1753
SLC25A40	1.21197939	1.0756E-06	up	776	800	750	193	212	309
SLC25A42	-1.365430015	4.23823E-05	down	115	132	143	219	292	192
SLC25A45	0.828680813	0.031706475	up	362	392	348	95	186	151
SLC25A5	1.60593338	2.61583E-06	up	8460	9246	8131	1824	2639	1429
SLC25A53	-1.818515939	7.10777E-05	down	20	15	16	35	40	53
SLC26A1	2.859145021	0.007193743	up	11	19	22	1	3	1
SLC26A10	-8.035986191	3.8094E-10	down	2	0	0	197	42	149
SLC26A2	1.083108329	7.16939E-05	up	1482	1638	1392	377	482	647
SLC26A4	-5.340232869	0.000643639	down	6	0	0	125	9	51
SLC26A5	-4.802226519	0.018548788	down	0	0	1	1	8	12
SLC26A6	1.600372185	1.99005E-06	up	1010	1122	1075	298	135	334
SLC26A7	-6.774943561	1.07767E-06	down	1	1	0	18	88	42
SLC27A1	-1.538813745	5.49176E-06	down	219	245	196	366	581	385
SLC27A2	6.303882693	1.09677E-51	up	574	660	546	7	5	4
SLC27A4	2.470806225	1.64255E-18	up	1514	1650	1420	149	235	194
SLC28A1	-4.268217936	0.029674089	down	2	0	0	12	1	16
SLC29A2	3.176328532	6.49383E-12	up	873	952	874	32	95	80
SLC2A1	2.726962102	1.0838E-118	up	3636	3946	3748	450	372	395
SLC2A10	-11.50857126	2.13683E-20	down	0	0	0	479	296	378
SLC2A12	-10.24056303	4.39168E-13	down	1	0	0	491	84	385
SLC2A13	1.405588259	1.42609E-19	up	935	1036	925	242	246	287
SLC2A3	-1.777387353	0.001478879	down	218	235	192	333	868	301
SLC2A4	-4.153390453	7.58912E-07	down	38	65	52	171	1358	285
SLC2A9	-3.021572112	6.67796E-12	down	16	20	9	105	83	70
SLC30A1	1.586705235	3.40031E-05	up	676	772	700	99	204	196
SLC30A4	-2.699951788	1.89521E-16	down	38	62	46	177	230	259
SLC30A9	-0.7719501	1.23737E-05	down	871	1089	924	1175	1199	1096
SLC31A2	-0.840854036	0.044415747	down	146	137	145	116	160	270
SLC34A2	5.110656932	0.005117984	up	7	11	9	0	0	0
SLC34A3	5.486286915	0.000174119	up	17	25	26	1	0	0
SLC35A1	-1.695873855	1.32344E-09	down	250	293	268	877	434	580
SLC35A2	2.832597045	4.48456E-80	up	1512	1537	1426	156	145	145
SLC35A3	0.990299452	1.19697E-05	up	1451	1465	1315	562	350	620
SLC35A5	-1.442873631	1.93051E-15	down	354	388	319	771	522	771
SLC35B2	1.427880391	4.14426E-24	up	1751	1665	1662	485	420	438
SLC35B3	-0.960824571	2.98312E-06	down	312	262	246	404	314	424
SLC35C1	0.606945606	0.032817541	up	474	537	471	230	269	183
SLC35D1	-0.801755065	0.031879615	down	375	389	363	489	257	678
SLC35D2	-0.873477385	1.46649E-06	down	212	290	245	348	297	325
SLC35E1	0.708359471	9.37733E-08	up	1409	1575	1558	661	589	728

SLC35E4	1.441344523	1.85323E-07	up	205	255	201	75	41	58
SLC35F1	-9.490140399	4.39168E-13	down	0	0	0	118	57	112
SLC35F2	2.893012218	4.5397E-19	up	1394	1631	1338	182	74	169
SLC35F3	7.288389598	3.17651E-24	up	364	397	334	5	0	0
SLC35F5	0.650857826	1.44601E-07	up	2472	2570	2346	1120	975	1256
SLC35G1	1.744046861	0.011314695	up	219	190	149	16	70	27
SLC35G2	1.073342217	0.004041532	up	622	696	585	185	287	155
SLC36A4	2.219385568	5.88365E-52	up	1413	1482	1395	234	176	248
SLC37A1	0.851575643	0.003656817	up	292	325	283	88	120	144
SLC37A2	-2.703653703	3.23136E-16	down	36	51	46	234	133	256
SLC37A4	1.603293728	2.94306E-05	up	72	109	99	19	23	23
SLC38A1	3.635671814	1.9951E-128	up	12249	13550	11655	837	659	644
SLC38A11	-7.289890083	2.76351E-06	down	0	0	0	11	27	22
SLC38A5	1.961755694	2.68803E-08	up	214	230	234	39	53	30
SLC38A6	-1.009363677	4.23476E-05	down	112	168	147	233	188	188
SLC38A7	1.3219065	3.94161E-05	up	714	861	785	179	284	194
SLC39A1	1.325012381	1.84704E-20	up	5418	5914	5436	1876	1262	1651
SLC39A10	2.197182627	1.16911E-61	up	1864	1981	1825	290	258	332
SLC39A12	4.00716539	0.029525854	up	8	4	12	0	0	1
SLC39A14	2.555959572	1.85971E-35	up	8121	8909	8502	867	1102	1088
SLC39A4	2.307731031	2.23155E-20	up	360	436	325	61	54	45
SLC39A8	2.384540642	7.74595E-59	up	1224	1373	1373	184	162	195
SLC3A1	4.421374282	0.001274062	up	20	24	16	0	0	2
SLC3A2	2.40270406	1.08474E-30	up	7687	8794	7765	1221	1134	879
SLC40A1	-15.1977541	1.357E-33	down	0	0	0	6691	2926	5417
SLC41A2	-1.84543145	0.001722714	down	254	246	258	982	181	850
SLC41A3	1.041480947	1.52186E-07	up	2158	2326	2164	962	561	797
SLC43A1	-0.654359038	0.012278582	down	182	260	205	233	189	303
SLC43A3	2.761270115	5.1393E-116	up	7318	7999	7207	845	759	751
SLC44A2	-1.185881996	3.15737E-11	down	1455	1612	1589	2753	1890	2956
SLC44A5	-6.862929677	1.13787E-05	down	1	0	0	50	29	10
SLC45A1	-3.636763997	7.69215E-08	down	8	3	11	75	34	92
SLC45A3	0.74553212	0.014274905	up	267	250	214	119	67	128
SLC46A3	-1.401325447	5.24278E-21	down	229	237	207	435	387	441
SLC4A1	-8.817999829	0.0402359	down	0	0	0	0	127	37
SLC4A10	-5.986735868	0.014128892	down	0	0	0	3	19	1
SLC4A11	2.886596661	2.60258E-05	up	66	87	98	2	8	14
SLC4A2	1.906724424	1.45317E-62	up	5097	5154	4903	957	873	1045
SLC4A4	-8.214491004	4.41977E-11	down	1	1	2	54	623	107
SLC4A5	-3.747928443	5.13317E-07	down	8	3	2	36	29	61
SLC4A8	-5.594743991	2.20892E-05	down	0	2	0	31	14	24
SLC50A1	1.42450786	3.55705E-07	up	1183	1320	1144	372	354	230
SLC51B	5.807955177	4.63815E-11	up	88	119	106	4	0	0
SLC52A2	1.419898316	6.36868E-07	up	870	949	838	216	287	191
SLC5A10	4.943809755	0.001466034	up	15	21	11	1	0	0
SLC5A3	0.894421209	0.002024878	up	3295	3352	3335	1115	923	1819
SLC5A4	-3.063982009	3.77985E-07	down	6	5	5	26	30	39
SLC5A5	7.837085541	3.93504E-18	up	276	369	320	0	0	3
SLC6A1	-10.04279025	4.59424E-11	down	0	0	0	99	250	44
SLC6A12	2.373921115	0.000659718	up	57	63	90	17	9	3
SLC6A15	8.291534106	3.71509E-57	up	1295	1471	1206	3	3	3
SLC6A16	-1.06372723	0.0034152	down	72	80	75	147	66	130
SLC6A17	3.95654409	3.68611E-20	up	191	171	165	7	11	6
SLC6A2	-5.641493946	0.005040949	down	0	0	0	5	3	12
SLC6A4	-9.618476067	1.66769E-08	down	0	0	0	204	95	11
SLC6A6	2.718004963	2.05692E-06	up	7483	7922	7664	371	1416	590
SLC6A8	2.667195825	2.46318E-05	up	5851	6265	6559	416	1271	296
SLC6A9	1.123094331	0.002724179	up	358	394	384	194	92	89
SLC7A1	2.754767838	1.94068E-52	up	2597	2907	2506	291	297	249
SLC7A10	-8.958677937	1.74981E-05	down	0	0	0	3	158	17
SLC7A11	5.520712829	9.31388E-90	up	3178	3748	3434	45	68	45
SLC7A4	-7.206963934	0.008386113	down	0	0	0	0	40	14
SLC7A5	8.348096761	3.4979E-142	up	22284	25711	23016	70	55	29
SLC7A6	1.040292741	0.001745683	up	2080	2399	2084	530	940	750
SLC7A6OS	0.633007749	0.000521306	up	396	454	388	214	148	208
SLC7A7	-3.273047394	1.47853E-10	down	49	45	45	378	123	484
SLC7A8	-1.833203281	9.9648E-05	down	201	235	222	312	426	936
SLC7A9	-4.805979903	0.037865874	down	0	0	0	2	3	6
SLC8A1	-7.009005133	8.19758E-16	down	12	7	10	388	1862	244
SLC8A3	-5.001456728	0.035514696	down	0	0	0	5	1	7
SLC8B1	1.293680243	8.40815E-14	up	1342	1520	1420	374	379	487
SLC9A3	2.841319922	5.12593E-14	up	182	185	150	11	18	22
SLC9A3R1	3.021721628	8.75491E-25	up	2346	2612	2418	202	269	163
SLC9A3R2	-0.917416563	0.000207786	down	856	845	882	927	1236	1278
SLC9A5	1.967371947	1.12655E-10	up	166	166	197	29	34	33
SLC9A7	-2.825343875	6.64606E-11	down	19	14	16	92	53	106
SLC9A9	-8.663070869	9.10423E-28	down	0	1	3	369	309	481
SLC9B1	-3.275556482	0.002421684	down	3	1	2	24	11	7
SLC9C1	-4.337833396	0.034767218	down	0	1	0	6	1	9
SLCO1B1	5.823250585	0.000345332	up	11	15	18	0	0	0
SLCO1B3	9.35788804	1.31177E-26	up	850	981	934	0	0	3
SLCO1B7	7.299355188	1.23616E-07	up	31	49	43	0	0	0
SLCO2A1	-2.661438365	8.73898E-17	down	279	277	284	855	1218	1692
SLCO2B1	-7.315906784	1.80313E-40	down	4	10	11	537	898	1375
SLCO3A1	1.583590772	4.93386E-14	up	2400	2610	2356	518	646	563
SLCO4A1	3.02083478	1.44753E-09	up	2258	2372	2440	120	122	387
SLCO4C1	-6.904206732	3.07808E-05	down	0	0	0	7	20	19
SLF1	3.876690366	1.04084E-81	up	1999	2198	1925	114	101	80
SLF2	-0.759277257	8.29757E-05	down	517	586	472	585	655	638
SLFN11	-10.16166622	1.89381E-31	down	0	2	1	890	709	847
SLFN12	-1.682784541	1.68248E-11	down	147	131	101	269	260	336
SLFN12L	-6.849990554	0.000198025	down	0	0	0	19	3	25
SLFN13	-11.11610477	1.11547E-16	down	0	0	0	399	122	375
SLFN5	-2.706454922	2.61438E-11	down	313	404	366	899	1919	2117
SLIT2	-4.737066312	4.79166E-28	down	69	61	76	1206	676	2122
SLIT3	-11.09349289	4.03856E-57	down	1	6	0	3286	2409	5195
SLITRK1	-4.92552612	0.024702045	down	0	0	0	3	3	6
SLITRK2	-6.058705531	0.000332845	down	0	0	0	8	9	9
SLITRK3	-6.405851074	0.022731063	down	0	0	0	17	0	18
SLITRK4	-10.86536062	3.80527E-16	down	0	0	0	251	118	380
SLITRK5	3.919206347	8.88147E-05	up	284	349	281	32	0	13

SLMAP	-2.378489501	6.39322E-06	down	1290	1382	1317	8429	1658	5186
SLN	-6.07637505	0.000158266	down	0	3	0	8	96	26
SLPI	1.935151875	0.01225052	up	2116	2252	2114	526	55	677
SLU7	-0.797945852	8.35432E-05	down	991	1036	940	1504	888	1315
SLX4	1.466066429	9.39824E-10	up	463	526	465	155	84	139
SLX4IP	-1.57599975	9.64847E-05	down	100	80	101	304	120	186
SMAD1	-1.234361501	9.11839E-09	down	238	274	193	400	399	370
SMAD3	-0.810154867	0.000173971	down	939	969	1005	1133	974	1548
SMAD4	-0.798159466	9.21542E-07	down	925	1019	952	1241	947	1413
SMAD7	-0.605513144	8.47118E-06	down	467	475	453	520	443	549
SMAD9	-8.00066826	1.42663E-41	down	7	5	4	1345	424	1229
SMAGP	1.193972273	0.001209858	up	222	220	250	89	79	46
SMAP2	-0.978678973	0.010289952	down	552	646	580	631	569	1314
SMARCA1	-2.820429289	1.10238E-43	down	228	232	249	1319	881	1400
SMARCA2	-2.624843382	3.56541E-41	down	529	567	561	2857	1751	2742
SMARCA4	1.404878837	6.73819E-22	up	2172	2374	2217	595	613	598
SMARCC1	2.263741385	2.51283E-20	up	5124	5524	5003	589	762	952
SMARCD1	1.528004248	5.85E-11	up	1885	2268	1904	395	515	568
SMARCD3	-2.078830216	1.70424E-10	down	363	338	322	1262	575	1302
SMARCE1	-1.031165533	2.63034E-06	down	1501	1611	1413	2644	1510	2501
SMC1A	2.076079248	5.8264E-30	up	6834	7434	6423	1004	1132	1334
SMC2	2.248124235	7.74693E-39	up	2464	2648	2341	462	317	340
SMC3	1.969524482	3.13198E-38	up	5816	6535	5678	1162	852	1277
SMC4	2.345931616	1.62721E-48	up	7307	7864	7020	1271	819	1034
SMC5	0.722462643	0.000624112	up	1686	1833	1659	623	712	887
SMC6	0.675251272	0.006842751	up	1292	1420	1335	520	691	566
SMCHD1	-0.709237853	0.012775991	down	1101	1231	1066	1109	1014	1847
SMCO3	-8.25865446	1.70043E-09	down	0	0	0	56	26	40
SMCO4	0.827002443	0.01467489	up	208	258	243	135	60	93
SMCR8	1.430647266	3.93324E-09	up	1676	1727	1707	357	417	572
SMG1	0.611956534	0.015016516	up	4967	5325	5071	1908	2144	3090
SMG6	-1.302794817	5.05496E-11	down	403	471	429	759	593	946
SMG7	1.191272611	1.61821E-18	up	2332	2649	2475	754	761	796
SMG8	0.77887667	4.62933E-05	up	567	631	466	240	222	224
SMG9	0.74292615	4.74821E-08	up	617	674	585	280	237	280
SMIM1	-2.86474811	6.68098E-06	down	6	6	3	30	20	28
SMIM10L1	-2.157991064	0.020731802	down	2	5	3	15	6	11
SMIM10L2A	-5.658263486	2.31254E-07	down	2	0	1	26	48	32
SMIM10L2B	-2.800435246	2.01667E-09	down	15	10	11	71	40	70
SMIM11B	-6.346858412	0.045168909	down	0	0	0	0	2	31
SMIM13	0.683661755	0.000223784	up	641	619	527	304	237	251
SMIM14	-1.908120396	6.95098E-09	down	549	658	475	1665	854	2036
SMIM15	0.849149663	5.99308E-08	up	1677	1900	1663	833	583	658
SMIM17	-3.683759725	0.00571379	down	3	0	1	6	18	12
SMIM19	-3.18136759	5.41973E-23	down	107	93	97	933	504	501
SMIM20	-1.329160685	8.37301E-07	down	219	245	236	557	369	328
SMIM24	4.935334247	0.00242013	up	10	12	24	0	1	0
SMIM3	-7.599338557	6.82557E-23	down	7	2	6	270	1149	581
SMIM6	6.051617396	1.41236E-05	up	33	29	38	1	0	0
SMIM7	-0.803225206	5.23354E-11	down	886	929	812	1218	949	1098
SMIM8	-0.920492683	0.023936861	down	126	94	133	226	95	166
SMKR1	5.394882193	1.619E-05	up	46	33	38	0	0	2
SMN1	1.269328704	0.002549096	up	1962	2177	1871	841	262	716
SMNDC1	1.098923165	8.68101E-06	up	1390	1531	1358	642	332	463
SMO	-1.691327203	4.39168E-13	down	134	135	103	331	216	313
SMOC1	2.767814931	8.54151E-07	up	5413	5703	5453	1093	439	221
SMOC2	-16.10764922	4.25146E-32	down	0	0	0	13893	2723	12114
SMOX	1.823655339	3.91274E-22	up	447	523	454	106	74	107
SMPD3	-4.965563641	0.005051647	down	1	0	0	4	6	14
SMPDL3A	-2.951910152	1.03573E-07	down	41	26	30	99	134	307
SMPDL3B	3.565258193	0.001230538	up	20	21	27	0	3	1
SMPX	-12.20381417	1.08023E-05	down	0	0	0	33	1549	96
SMS	3.140566421	8.5067E-30	up	13962	14753	13458	1540	691	1230
SMTN	-3.271221292	1.00445E-07	down	468	549	575	4170	896	6272
SMTNL2	-6.108466316	2.98689E-05	down	1	2	0	7	96	33
SMUG1	0.998546795	0.007870739	up	644	682	679	342	227	145
SMYD3	1.610231525	6.40625E-25	up	552	577	538	140	112	137
SMYD5	0.815521749	1.83276E-05	up	709	782	704	269	309	302
SNAI2	-1.579423561	0.003391014	down	261	273	273	310	920	416
SNAI3	-3.115003267	0.00261654	down	4	3	3	43	6	15
SNAP23	-1.092051471	6.15751E-07	down	1014	1119	979	1631	1131	1998
SNAP47	0.904825472	2.68878E-05	up	780	824	784	339	313	250
SNAP91	-9.682180445	1.81642E-10	down	0	0	0	105	174	31
SNAPC1	0.894625391	3.26014E-09	up	465	508	454	187	170	188
SNAPC2	-0.857410551	0.008819985	down	157	149	140	276	144	161
SNAPC3	-0.714486003	0.00095889	down	770	790	752	1051	634	1045
SNAPC4	2.101320854	9.19156E-29	up	849	864	782	161	132	120
SNCA	1.094373383	2.01281E-12	up	476	567	495	177	159	175
SNCAIP	-7.627524089	4.25595E-15	down	4	0	0	195	96	288
SNCG	-3.213951823	3.30379E-26	down	101	109	92	760	737	473
SND1	1.766249747	5.03872E-77	up	9078	9462	9179	1996	1780	2018
SNED1	-3.137100763	5.57623E-27	down	291	327	350	1819	1426	2878
SNF8	0.900757628	3.43205E-07	up	1669	1948	1704	804	626	593
SNN	-1.460722039	0.000672438	down	392	395	419	452	1067	782
SNPH	-10.36660452	2.27653E-16	down	0	0	0	203	122	199
SNRK	-1.509085437	6.22882E-05	down	524	629	571	907	1592	892
SNRNP200	1.790770978	6.83385E-49	up	11496	12636	11553	2473	2071	2808
SNRNP25	1.587427758	2.86051E-10	up	1137	1219	1093	290	306	211
SNRNP35	-1.925080335	4.36214E-15	down	177	163	169	586	328	479
SNRNP40	1.509734141	3.36553E-10	up	1287	1443	1157	430	228	322
SNRNP48	0.672854085	0.000263243	up	837	927	842	347	357	456
SNRPA	0.805352818	0.000858136	up	1361	1414	1364	707	377	624
SNRPA1	2.473270754	6.76736E-36	up	3115	3708	3166	552	335	399
SNRPB	1.849679472	4.75685E-60	up	3839	4369	3916	906	679	812
SNRPC	1.063636358	1.43687E-19	up	1668	1976	1715	667	548	606
SNRPD1	1.624305879	7.75301E-11	up	1687	1922	1432	525	307	336
SNRPD3	0.945263795	1.76686E-12	up	3300	3773	3173	1402	1016	1380
SNRPE	1.611020356	3.7667E-07	up	1963	2226	1956	724	348	374
SNRPF	1.77554543	1.30045E-12	up	2044	2318	1842	547	410	327
SNRPG	1.124760732	1.66905E-05	up	2115	2351	2005	963	601	553
SNRPN	-8.09341001	2.1119E-09	down	0	0	0	37	27	44

SNTA1	-1.143779338	2.84192E-05	down	635	659	694	1464	747	956
SNTB1	-2.058173021	4.05323E-07	down	143	142	143	396	573	269
SNTG2	-6.563678222	2.58164E-05	down	0	0	0	11	12	14
SNW1	0.717750201	3.19146E-06	up	2644	2672	2502	1337	900	1167
SNX1	-0.604700225	0.000301648	down	2309	2521	2220	2338	2270	3009
SNX10	2.396478731	1.78216E-47	up	1150	1233	1053	155	128	182
SNX16	-1.085159156	2.78456E-05	down	156	162	104	215	204	214
SNX17	-0.674928109	4.86018E-10	down	1236	1409	1213	1555	1352	1463
SNX18	-4.123513628	8.07742E-24	down	163	160	154	2594	876	2608
SNX20	-9.133126226	2.0715E-10	down	0	0	0	130	31	66
SNX21	-1.708102963	3.64479E-23	down	374	437	376	1049	703	1023
SNX24	0.728516918	0.005856497	up	567	582	486	235	165	308
SNX29	-1.374138749	9.58465E-08	down	314	338	300	512	461	790
SNX3	-1.385336412	7.10556E-29	down	2004	2155	1950	3789	3716	3790
SNX31	-4.572423334	8.29789E-05	down	1	1	1	19	21	10
SNX32	-6.051915628	0.002300434	down	0	0	0	13	2	12
SNX8	1.319946817	4.04256E-10	up	1054	1091	1042	276	334	289
SNX9	-1.329287939	1.90454E-07	down	1330	1448	1312	2670	1588	3150
SOAT1	1.837233617	1.06514E-09	up	3615	4170	3623	544	912	770
SOAT2	-6.1977029	0.004796861	down	0	0	0	9	1	20
SOBP	-4.800456449	1.75771E-63	down	31	19	19	480	427	462
SOCS1	-2.774077039	0.000363364	down	3	16	6	63	27	31
SOCS2	-1.135068641	0.003066518	down	301	406	279	343	649	506
SOCS4	0.594150505	0.000181738	up	1372	1370	1226	623	526	726
SOCS6	-1.680478981	5.44018E-19	down	229	237	253	637	425	591
SOCS7	1.409611974	0.000149719	up	119	116	119	22	36	36
SOD3	-15.14964778	7.69038E-30	down	0	0	0	6983	1717	6034
SOGA1	0.825619853	5.85782E-07	up	1852	1956	1714	681	742	779
SOGA3	-6.083899482	0.000184744	down	1	0	0	23	22	6
SORBS1	-6.142550096	3.49243E-44	down	318	302	335	15046	7341	26886
SORBS2	-6.363995668	1.12439E-52	down	91	126	92	3772	4476	9875
SORBS3	-1.846853414	1.84236E-18	down	677	840	725	2019	1386	2373
SORCS1	-1.915360585	0.00016976	down	18	29	24	63	82	41
SORCS2	3.553782622	6.79146E-12	up	367	423	398	15	12	46
SORD	2.356890492	1.67948E-22	up	1467	1634	1460	270	201	161
SORT1	-2.640399119	3.90897E-16	down	1071	1122	1096	4188	3205	7382
SOS1	-1.141473277	9.68025E-06	down	838	1131	916	1267	1715	1464
SOS2	-1.600616275	4.53763E-08	down	266	281	292	441	593	767
SOST	-9.385456266	2.19747E-08	down	2	0	0	841	15	162
SOWAHA	-6.663924789	0.000105116	down	0	0	0	22	5	14
SOWAHC	2.713077743	2.69381E-25	up	2646	2915	2663	344	184	377
SOX10	-4.530303076	2.91002E-10	down	3	4	3	45	75	40
SOX12	0.971140258	0.001295829	up	479	533	500	159	225	155
SOX15	-6.471975425	7.49701E-05	down	0	0	2	95	6	33
SOX17	-1.86000951	2.48273E-07	down	57	63	77	197	187	121
SOX18	-4.299877794	4.1056E-19	down	56	48	53	1228	621	373
SOX4	0.879499467	0.004503475	up	1138	1086	1096	502	475	296
SOX5	-7.86087475	9.41239E-17	down	0	4	0	165	146	348
SOX6	-10.02871728	1.01685E-11	down	0	0	0	61	239	90
SOX7	-2.789983584	3.34642E-07	down	45	33	34	254	212	81
SOX8	-7.303532081	4.21986E-14	down	1	1	1	161	93	85
SP100	-1.980293992	2.35341E-56	down	507	501	493	1443	1239	1539
SP110	-2.565904811	2.13593E-41	down	89	125	115	485	422	475
SP140	-3.815310359	0.000175901	down	2	2	1	17	22	10
SP140L	-0.91339797	0.004903654	down	171	179	226	197	278	291
SP2	-0.820789192	1.87198E-05	down	237	277	293	339	292	385
SP3	-0.761795657	0.000109807	down	1274	1370	1216	1493	1233	1951
SPACA6	-1.867995908	3.24142E-05	down	60	57	66	214	75	199
SPACA9	-1.432914493	3.40431E-05	down	59	68	60	142	73	149
SPAG1	0.77270085	0.013233529	up	229	220	239	86	71	132
SPAG16	-1.049117585	0.000513994	down	261	307	229	496	242	450
SPAG4	2.685720947	1.41986E-06	up	148	169	136	28	16	6
SPAG5	5.151348181	2.80366E-46	up	3393	3483	3059	105	55	40
SPAG8	-5.434071322	2.22285E-06	down	0	5	0	69	24	60
SPANXB1	5.544421061	0.00123682	up	12	9	15	0	0	0
SPARC	-15.78436796	1.7287E-105	down	0	2	3	93160	49494	60082
SPARCL1	-17.38577356	2.87265E-47	down	1	1	0	78175	27694	144500
SPAST	0.666076879	0.004160967	up	651	659	513	236	269	307
SPATA12	3.81771425	0.00432224	up	24	21	17	0	3	0
SPATA13	-0.655775443	0.016536628	down	381	378	381	329	432	509
SPATA17	5.413379384	1.08681E-15	up	113	186	125	2	3	2
SPATA18	-6.854157789	1.39747E-15	down	0	4	3	231	72	285
SPATA2	0.687949581	0.003534059	up	363	336	339	171	111	181
SPATA20	-0.916786836	2.3268E-05	down	520	575	564	934	669	628
SPATA2L	1.276425193	0.030356071	up	273	234	264	146	32	56
SPATA33	1.994285917	1.23962E-09	up	273	293	230	58	51	32
SPATA5	1.487590324	6.03337E-19	up	464	512	463	127	118	119
SPATA5L1	1.354290922	5.9767E-06	up	553	642	560	136	201	142
SPATA6	-5.376211379	5.88806E-30	down	5	9	14	269	191	377
SPATA6L	-3.401224658	1.7109E-05	down	2	3	10	37	25	53
SPATA7	-1.75903292	9.01396E-08	down	85	80	84	287	154	165
SPATA9	-4.024483162	5.44647E-05	down	3	1	4	11	43	37
SPATS2	2.103299128	2.79546E-33	up	1546	1671	1391	311	218	235
SPATS2L	-1.907728685	7.73998E-53	down	382	434	399	1106	948	1190
SPC24	4.495107704	8.26602E-17	up	667	693	636	42	13	9
SPC25	4.563335766	0.000830289	up	16	27	23	2	0	0
SPCS3	-1.066669872	2.41823E-16	down	1187	1290	1086	1764	1553	1990
SPDEF	3.751824913	0.000460351	up	24	33	21	0	3	1
SPDL1	4.332643551	5.6953E-124	up	3628	3925	3358	111	129	143
SPDYE18	-3.110836497	0.005779834	down	1	3	1	13	5	13
SPECC1L	-1.196196339	1.48332E-08	down	290	295	264	543	335	519
SPEF2	-2.82175879	5.23625E-17	down	28	37	20	175	112	141
SPEG	-6.207512078	3.55793E-20	down	88	73	90	7783	848	5203
SPESP1	-8.387385178	7.49152E-09	down	0	0	0	56	18	61
SPG11	-0.777943506	0.000652507	down	641	708	667	676	860	900
SPG20	-0.689638385	0.000355432	down	1283	1319	1134	1643	1054	1627
SPG21	0.622175877	0.000260176	up	2659	2729	2550	1157	1267	1218
SPHK1	2.457951222	6.9301E-10	up	1219	1240	1208	213	175	83
SPHK2	-0.91338216	0.000130795	down	172	211	153	263	241	208
SPI1	-7.481118842	1.76488E-37	down	5	4	2	579	267	577
SPICE1	1.69144597	9.00257E-19	up	594	567	604	122	121	146

SPIN3	1.048271068	2.64419E-05	up	768	768	598	319	181	240
SPIN4	2.023995443	1.09651E-10	up	489	525	466	117	49	97
SPINK1	4.983633088	6.01551E-30	up	317	270	305	4	9	7
SPINK13	8.950420228	3.72205E-24	up	608	759	722	0	1	2
SPINK4	6.743403666	7.49117E-06	up	31	37	16	0	0	0
SPINK5	6.159763472	0.000315782	up	2462	3026	2410	11	61	0
SPINK6	9.364530421	2.06184E-42	up	1577	1603	1473	0	5	0
SPINK7	5.571790443	0.003894552	up	7	25	6	0	0	0
SPINT2	-5.930258203	1.09348E-11	down	59	43	51	2745	178	4061
SPIRE2	3.476616993	5.12745E-13	up	723	676	719	28	72	31
SPN	-5.32741088	1.98598E-07	down	0	4	0	40	29	44
SPNS1	0.670854416	0.000132176	up	885	922	835	482	323	381
SPNS2	-2.115849295	2.46107E-09	down	171	146	169	461	641	371
SPOCK1	-4.795550602	3.31254E-29	down	115	117	88	1090	2485	2620
SPOCK2	1.503528221	2.17818E-07	up	416	441	401	123	115	75
SPON1	-14.07521741	1.24909E-28	down	0	0	0	3435	1642	1785
SPON2	1.573376624	0.027728503	up	1089	1165	1192	119	527	130
SPOP	-1.26367571	0.001937209	down	851	991	838	2382	752	1550
SPOPL	-0.800787244	0.007543966	down	556	605	611	683	503	1035
SPP1	-9.975981038	3.57747E-28	down	11	2	1	6461	2706	1092
SPPL3	-0.661286856	1.27059E-05	down	538	583	480	686	503	616
SPRED1	-1.720084731	1.47836E-32	down	396	492	372	1031	865	1050
SPRTN	1.610131696	7.3992E-15	up	807	797	706	214	130	199
SPRY1	-4.676982452	1.322E-13	down	78	85	80	534	2587	1075
SPRY2	-3.171724246	4.47646E-21	down	132	131	117	638	1044	700
SPRY4	-2.926221743	2.81583E-32	down	118	151	137	911	688	590
SPRYD4	-1.130104152	0.006184263	down	143	146	145	205	304	151
SPSB2	1.417791702	6.58638E-08	up	350	375	320	104	100	72
SPSB3	-1.029552133	0.000743643	down	420	433	419	774	365	742
SPSB4	-6.331537276	0.001574964	down	0	0	0	15	14	2
SPTAN1	0.758494564	0.000121595	up	10550	11243	10901	4006	4094	5645
SPTB	-3.696428406	2.93925E-05	down	28	32	25	43	509	181
SPTBN1	-1.37371634	6.30226E-10	down	7128	7438	7308	11508	11442	17468
SPTBN2	5.804814317	3.01749E-89	up	1110	1105	1013	11	15	15
SPTLC3	-3.238219269	2.5649E-14	down	17	45	20	206	139	202
SPTSSB	-6.825956206	7.58033E-06	down	0	0	0	13	17	14
SPTY2D1	0.736138095	0.001167021	up	1208	1129	1243	582	364	603
SQLE	3.792268447	9.05701E-36	up	3107	3454	3031	126	210	146
SQRDL	-1.533023305	3.75109E-08	down	404	352	333	986	493	800
SRD5A1	4.184536759	1.2325E-186	up	2541	2837	2516	115	88	106
SREBF2	1.141351413	1.22288E-05	up	3156	3286	3192	779	1098	1200
SREK1IP1	-0.699535253	0.020671032	down	717	717	675	1160	536	779
SRF	-1.129271486	0.000857187	down	877	1030	899	1972	794	1685
SRFBP1	0.895337185	5.25089E-05	up	324	398	335	118	137	146
SRGAP1	2.062061887	2.37702E-15	up	822	834	800	105	148	162
SRGAP3	-1.345143867	0.000511847	down	29	40	25	61	59	48
SRGN	-9.594073836	1.20143E-92	down	4	3	5	2652	1895	2074
SRM	1.246649425	2.47869E-08	up	1479	1599	1234	395	473	408
SRMS	8.837370554	3.21295E-12	up	117	127	112	0	0	0
SRP14	-1.069617479	5.25188E-06	down	2903	3127	3022	6096	3302	4269
SRP19	0.676451497	2.09704E-05	up	867	1057	915	471	397	391
SRP54	0.668464242	3.19547E-06	up	1721	1821	1455	813	635	791
SRP72	0.587773785	2.55278E-07	up	2882	3400	2996	1463	1339	1578
SRPK1	2.456243426	4.06808E-95	up	3514	3955	3532	466	437	520
SRPK2	0.97584184	1.80782E-16	up	1707	1888	1710	650	614	648
SRPK3	-2.138082146	1.60085E-06	down	21	31	31	104	96	57
SRPRB	1.321160785	0.000108495	up	1921	2053	1818	594	671	355
SRPX	-10.49538744	4.14317E-30	down	0	0	5	1266	2590	1153
SRPX2	-8.452786809	3.34104E-20	down	1	4	0	176	462	572
SRR	-0.785656285	0.035919916	down	146	198	129	203	229	136
SRRM3	1.316435441	1.99879E-07	up	269	341	362	114	74	91
SRRM4	-2.442963565	0.003604668	down	5	3	3	12	10	21
SRRT	1.178428909	8.81239E-12	up	2412	2631	2370	874	579	895
SRSF1	1.005027774	1.76688E-13	up	8190	8799	7475	3185	2317	3208
SRSF10	0.82625289	5.63702E-06	up	4913	5455	4516	2081	1519	2405
SRSF12	-6.712726268	1.6833E-05	down	0	0	0	17	14	10
SRSF2	0.771542143	0.000136576	up	3929	4218	3770	1984	1177	1857
SRSF3	0.671800493	0.02247852	up	6723	7104	6097	4272	2341	2351
SRSF5	-1.420677657	7.06887E-10	down	3114	3616	3168	6292	4474	8243
SRSF8	-1.572057471	4.24857E-10	down	485	515	458	1266	668	1193
SRSF9	1.697083432	6.71981E-34	up	3905	3955	3496	962	740	792
SRXN1	1.472194839	1.20615E-08	up	2157	2283	1991	576	627	424
SS18	-0.861477416	9.8004E-07	down	1017	1188	1065	1412	1121	1710
SS18L2	0.655445374	0.039409899	up	318	405	408	224	161	125
SSBP1	1.133516292	0.000170459	up	869	983	794	414	225	224
SSBP2	-3.717233724	9.89215E-26	down	118	104	80	1346	554	980
SSC5D	-4.878102985	1.19379E-30	down	12	20	15	474	201	321
SSFA2	0.993414944	0.000541294	up	6552	7370	6572	1881	2039	3409
SSPN	-8.253584105	4.44879E-57	down	7	11	8	2627	852	2293
SSPO	-1.478381395	0.01076867	down	8	11	12	21	22	18
SSR1	1.310975411	1.17876E-47	up	7372	7927	7086	2294	1850	2283
SSR2	0.980639594	0.000241359	up	5532	5715	5377	2771	1689	1565
SSR3	1.432719661	1.21887E-41	up	8070	8783	7701	2184	2054	2208
SSRP1	2.13060248	1.18928E-90	up	6883	7300	6523	1128	1007	1227
SSSCA1	1.253347931	5.33938E-05	up	552	600	526	221	164	114
SSTR1	3.131503967	2.35855E-13	up	677	619	584	88	27	41
SSTR2	-7.769376621	1.84714E-08	down	0	0	0	25	30	30
SSX2IP	1.451941937	7.65943E-09	up	885	1053	1007	334	171	270
ST14	3.545398786	2.05862E-15	up	579	585	629	47	45	16
ST3GAL3	-1.63570533	4.66691E-08	down	129	168	96	354	207	311
ST3GAL4	1.531571654	7.71429E-07	up	2062	2222	2134	628	586	349
ST3GAL6	-8.431394565	1.13753E-30	down	2	2	2	375	651	411
ST5	-1.994559166	1.10657E-24	down	302	275	334	952	694	960
ST6GAL1	-4.354560581	1.8902E-34	down	109	116	103	1103	1273	2411
ST6GAL2	-12.852369	3.4921E-11	down	1	0	0	3078	112	2748
ST6GALNAC1	-6.095890362	8.05845E-05	down	1	1	0	5	50	38
ST6GALNAC2	-2.283833552	0.000183464	down	10	30	17	66	84	41
ST6GALNAC3	-10.44046638	2.6564E-16	down	0	0	0	141	176	226
ST6GALNAC4	1.776428173	2.57295E-13	up	1410	1465	1373	314	328	231
ST6GALNAC5	-5.866316946	2.04887E-05	down	2	1	0	74	6	50
ST6GALNAC6	-2.166680805	5.08959E-17	down	210	208	218	618	518	912

ST8SIA1	-8.55465273	7.65743E-11	down	0	1	0	96	116	71
ST8SIA4	-3.332164067	2.14116E-22	down	31	18	27	181	188	175
ST8SIA6	-4.102516237	0.000199476	down	6	15	5	44	228	17
STAB1	-8.152582315	1.0155E-146	down	15	25	20	3348	4291	4344
STAC2	-3.284181234	0.040801555	down	15	19	11	49	0	275
STAC3	-3.312234096	5.45353E-09	down	4	5	10	53	39	43
STAG2	0.992141636	3.89669E-11	up	5618	5943	5459	2199	1585	2342
STAM2	-1.906160098	5.70572E-28	down	274	279	229	715	574	802
STAMBP	-0.889933805	1.77974E-06	down	476	551	484	718	502	784
STAMBPL1	1.300466796	0.001697292	up	332	310	295	66	126	72
STAP1	-5.406755422	0.007320058	down	0	0	0	9	3	5
STAP2	-2.801688324	2.77239E-06	down	8	9	5	33	45	29
STARD10	-0.963586324	0.001004402	down	214	231	199	412	250	235
STARD13	-2.467767364	1.35959E-45	down	261	220	231	934	875	995
STARD3	-0.626473766	0.000378829	down	470	520	543	576	463	652
STARD4	1.5346915	1.96288E-30	up	1051	1186	978	276	237	275
STARD5	-3.00737151	3.63003E-07	down	10	11	17	71	99	43
STARD9	-2.885798725	2.06253E-17	down	203	240	225	1075	724	1761
STAT2	-1.969926719	7.71815E-36	down	540	515	506	1397	1436	1501
STAT3	-0.617527314	0.000116174	down	2780	3046	3065	3027	3209	3419
STAT6	-1.68439874	2.45032E-10	down	1545	1653	1476	4139	2220	4491
STAU1	0.699807772	1.13183E-09	up	3979	4323	4002	1778	1607	2002
STC2	3.77001142	7.03243E-06	up	1657	1809	1709	49	187	16
STEAP1	-8.499686919	2.35253E-17	down	2	0	1	245	354	157
STEAP2	-11.99052728	9.98675E-22	down	0	0	0	721	404	488
STEAP3	3.155847782	3.4672E-84	up	2231	2573	2295	203	145	221
STEAP4	-7.058483569	6.88872E-33	down	44	49	81	1905	7032	7252
STIL	5.356476889	5.39892E-47	up	1294	1436	1338	31	28	11
STIM1	-1.032685646	1.81553E-06	down	705	925	758	1035	1243	1150
STIM2	-1.444367668	3.51843E-12	down	215	205	206	368	417	420
STIP1	2.519259091	2.9227E-101	up	7106	7669	6822	1018	801	861
STK10	0.832585309	0.006602709	up	1100	1080	1091	337	530	418
STK11	-3.535200149	1.03515E-33	down	76	53	77	578	610	506
STK16	-1.033487533	0.000130217	down	196	218	197	329	322	230
STK17A	0.647498661	0.046735984	up	1512	1565	1417	954	401	724
STK17B	-1.637438454	2.89571E-05	down	288	351	309	402	815	841
STK26	3.975878003	2.40529E-49	up	3042	3351	2933	152	86	189
STK32A	-4.63503985	1.36094E-13	down	13	7	13	252	72	282
STK32B	-9.093847946	1.43561E-12	down	0	0	0	79	69	66
STK33	-7.167563532	3.75585E-06	down	0	0	1	35	58	13
STK35	0.686383574	0.010889507	up	587	583	511	212	286	235
STK36	-1.184466361	1.65231E-10	down	184	202	225	349	284	358
STK38L	-1.964618717	3.81742E-05	down	1693	1837	1570	5554	1785	7222
STK4	0.967173547	1.97503E-06	up	1616	1610	1614	670	422	689
STK40	-1.297891319	1.43862E-15	down	497	465	522	874	829	892
STMN1	1.454017078	3.29076E-05	up	3520	3981	3191	1436	556	831
STMN3	-5.347875644	4.6799E-24	down	14	17	25	907	252	513
STOM	-0.697896768	3.03772E-07	down	4735	5063	4860	5352	5442	6056
STOML1	-1.727943392	1.4791E-08	down	94	103	89	263	240	165
STON1	-7.486103795	1.07344E-36	down	10	3	5	498	1037	717
STON1-GTF2A	-5.01286902	0.027362535	down	0	0	0	8	2	3
STON2	1.533841691	6.01623E-21	up	575	615	579	154	118	164
STPG1	0.953003868	0.000304038	up	202	223	215	72	88	73
STRA13	1.44636416	7.41425E-07	up	1136	1163	1076	407	264	212
STRADA	-1.005810478	2.35029E-10	down	216	253	222	337	286	364
STRADB	-1.514694113	0.001431517	down	202	187	165	215	542	334
STRAP	1.464018837	1.07821E-11	up	5410	5605	4862	1367	1508	1171
STRBP	3.182422028	1.63033E-40	up	2176	2385	2117	165	204	147
STRN3	-0.775981861	5.81286E-06	down	475	469	438	556	472	661
STT3A	1.524047381	1.39004E-55	up	5255	5809	5156	1482	1154	1381
STT3B	0.74057774	8.89677E-05	up	6224	6985	6235	3313	2011	3030
STUM	-10.04286755	3.00298E-15	down	0	0	0	140	152	118
STX10	0.690284348	7.59396E-05	up	998	1022	965	527	393	398
STX11	-2.601147263	0.000935554	down	57	51	52	96	458	95
STX12	-2.158021648	1.71228E-30	down	514	493	488	1813	1178	1805
STX17	-1.15429895	2.45489E-14	down	462	543	436	784	637	862
STX19	-6.561891163	0.001013099	down	0	0	0	28	7	3
STX1A	1.209390042	5.94704E-05	up	167	163	188	70	39	52
STX1B	2.187115828	0.00022126	up	76	105	125	8	12	28
STX2	-0.673655142	0.004214021	down	711	685	601	675	629	969
STX6	0.792586757	7.19779E-12	up	1546	1620	1521	679	603	640
STX7	-2.080807647	3.16975E-31	down	574	611	570	1961	1313	2053
STX8	-1.56919767	7.18105E-07	down	214	289	228	745	345	469
STXBP1	0.830237332	0.019409441	up	1344	1465	1357	355	658	624
STXBP2	2.380545496	3.79651E-22	up	1043	1144	1056	147	102	198
STXBP5L	4.26005379	0.02332415	up	3	10	16	0	1	0
STYK1	-4.126881444	0.009565807	down	2	0	0	6	8	11
SUB1	0.788115616	0.001978416	up	7046	8214	6778	3067	3450	2420
SUCNR1	-6.063209044	5.59848E-11	down	4	2	2	56	231	75
SUGCT	-3.98521492	1.89607E-09	down	36	15	22	380	84	396
SULF1	-7.105326661	5.93809E-49	down	48	50	50	7488	1820	5661
SULF2	-1.370005743	1.59127E-12	down	988	902	953	1756	1381	2134
SULT1B1	-6.703987681	0.000732098	down	0	0	0	2	17	21
SULT1C2	-7.800904848	0.000493781	down	1	0	0	0	85	79
SULT1C4	-10.89182119	3.91101E-17	down	0	0	0	282	290	164
SULT4A1	3.057771772	0.045068635	up	148	154	138	0	33	1
SUMF1	-0.851650949	0.019425654	down	326	488	360	759	339	422
SUMO1	0.587448875	2.90829E-05	up	3406	3791	3219	1901	1480	1546
SUN2	-2.354696982	3.31488E-05	down	1621	1918	1697	6794	1892	10967
SUN3	3.513685819	1.24868E-05	up	45	54	48	4	5	0
SUOX	-1.435540277	0.000698366	down	254	319	314	420	808	426
SUPT16H	0.971319712	1.81083E-06	up	4625	4769	4144	1767	1187	2003
SUPT3H	-1.218053866	0.010309231	down	55	46	55	116	42	107
SUPT5H	0.658533706	7.94116E-06	up	2869	2978	2734	1261	1103	1511
SUPV3L1	1.243829381	8.3289E-08	up	1140	1183	1137	329	394	304
SURF4	1.929323095	0.000940198	up	73	111	186	18	31	19
SUSD2	-2.046168338	2.95044E-09	down	105	128	144	291	458	338
SUSD3	-8.356498049	8.55581E-08	down	0	0	0	86	16	31
SUSD4	-7.435749796	1.13473E-07	down	0	1	0	48	56	26
SUSD5	-13.11580882	3.00649E-10	down	0	0	3	6217	266	13814
SUSD6	-1.295118123	8.64522E-24	down	366	371	360	675	563	680

SUV39H1	3.531079271	1.43641E-44	up	923	953	851	67	59	41
SUV39H2	2.114343059	1.80922E-07	up	471	518	439	55	111	62
SUZ12	1.77269746	3.97292E-41	up	2301	2509	2144	477	469	495
SV2A	-7.43203817	2.62448E-13	down	0	4	4	537	58	422
SV2C	-5.258217477	0.042219072	down	0	1	0	0	22	5
SVBP	-2.09538404	5.70079E-17	down	74	70	94	274	221	231
SVEP1	-3.019239002	2.44779E-09	down	359	367	304	873	2884	1976
SVIL	-3.131890581	5.81186E-18	down	1321	1413	1289	10930	4080	10676
SWI5	1.542452616	1.24396E-11	up	985	1169	1032	305	263	205
SWSAP1	-1.613746718	0.00323766	down	19	26	12	34	30	60
SWT1	-1.088188258	0.001709907	down	114	100	101	137	117	227
SYAP1	1.717487041	8.82383E-12	up	2763	3076	2793	503	720	615
SYCE1L	-2.206397889	0.0156235	down	14	10	9	78	22	11
SYCP2	1.524192017	2.49783E-05	up	313	320	322	50	78	107
SYCP2L	-3.814890491	0.000344168	down	1	2	3	29	6	27
SYDE1	0.778977798	9.05033E-06	up	1110	1240	1159	541	366	558
SYF2	-1.052518103	0.000141322	down	982	1013	934	1932	915	1545
SYK	-2.069221712	5.19371E-06	down	139	105	113	344	179	570
SYMPK	0.649757781	4.04598E-05	up	1260	1458	1382	642	502	722
SYN1	-5.312346058	0.001484394	down	0	0	2	9	37	8
SYN2	-6.855753073	1.88794E-05	down	0	2	5	6	473	48
SYNCRIP	0.747451258	1.487E-08	up	6474	7161	6315	2897	2330	3250
SYNDIG1	-10.53057836	1.02772E-15	down	0	0	0	220	232	120
SYNE1	-2.066963975	7.89985E-06	down	1492	1510	1423	4199	1918	7410
SYNE2	-1.222147182	0.006049709	down	1968	2299	2111	2110	2575	5959
SYNE3	-1.155055187	0.026121772	down	155	155	150	108	293	314
SYNGR1	-1.010631663	1.11369E-07	down	218	258	244	411	274	352
SYNGR3	6.961788376	1.12656E-22	up	284	312	280	0	1	4
SYNJ2BP	-0.701174536	0.0007248	down	872	1039	885	1135	794	1326
SYNM	-1.319578086	1.38951E-12	down	2182	2299	2170	4005	3009	4883
SYNPO	-3.100687667	4.76477E-60	down	1273	1531	1380	8706	6435	10548
SYNPO2	-7.220222061	6.63633E-71	down	100	60	65	9851	4168	10527
SYP	-3.557316154	7.91937E-11	down	10	22	23	195	72	203
SYPL1	-0.708159944	2.4953E-09	down	1823	1975	1670	2331	1779	2259
SYPL2	-1.7335287	2.59134E-08	down	81	66	60	147	183	155
SYT1	-1.640502846	0.0010535	down	23	19	18	67	30	38
SYT11	-7.331824015	4.34638E-31	down	16	10	17	2162	468	2514
SYT12	3.212956013	8.55237E-20	up	632	692	692	62	61	30
SYT15	-4.264249835	1.33629E-07	down	6	10	1	94	32	109
SYT16	5.015445542	0.001091365	up	20	17	12	0	1	0
SYT17	1.811066287	3.98303E-08	up	257	266	243	49	65	39
SYT3	-5.706623886	0.005964709	down	0	0	0	2	9	9
SYT6	-5.455881101	0.00975637	down	0	0	0	8	7	2
SYT7	2.023772043	0.00102697	up	295	298	310	16	58	82
SYT8	-5.947553514	0.000687281	down	0	0	0	9	9	6
SYTL1	-1.479838366	0.022617755	down	10	6	12	23	17	16
SYTL2	-3.291822057	2.91358E-50	down	81	111	101	834	541	675
SYTL3	-1.683876111	0.001376563	down	32	28	28	53	96	47
SYTL4	-9.513082319	7.90533E-13	down	0	0	1	129	135	299
TAB1	-0.97899998	4.5932E-11	down	282	307	280	447	338	437
TAB2	-0.724488273	0.000594194	down	1357	1501	1417	1784	1211	2077
TAB3	1.792458977	2.54351E-20	up	2350	2531	2202	415	455	579
TAC1	-5.411791787	0.001141535	down	0	1	0	5	13	14
TAC3	3.947094332	0.002521349	up	24	25	15	3	0	0
TACC1	-1.879612649	3.95402E-07	down	2000	2115	2143	4314	3520	8769
TACC2	-1.356410752	3.18273E-24	down	597	681	622	1126	1107	1213
TACC3	3.219469059	4.19028E-38	up	2020	2024	2046	205	143	118
TACR1	-1.771301396	0.001279338	down	44	36	25	134	75	47
TACR2	-5.578823025	0.000372479	down	0	1	0	13	15	8
TACSTD2	1.336326494	9.27997E-05	up	203	183	180	64	31	67
TADA1	0.588550546	0.014084452	up	353	380	380	216	124	192
TADA2B	-0.648601903	0.000136971	down	385	372	349	395	391	445
TAF13	1.006964263	0.000279257	up	770	906	727	243	337	255
TAF1A	1.758285638	2.2393E-16	up	338	382	328	67	76	76
TAF1B	1.110487974	0.000165297	up	300	374	337	83	107	141
TAF1C	-0.646636943	3.79069E-05	down	516	585	615	705	573	635
TAF1D	1.797837413	4.74509E-18	up	2259	2492	2063	547	325	535
TAF2	1.209112178	0.000116027	up	1217	1353	1289	272	380	529
TAF3	-1.393326824	1.76901E-05	down	187	187	161	454	200	363
TAF4	1.797495041	1.15806E-06	up	132	125	169	27	22	39
TAF4B	1.489505377	2.19451E-14	up	293	319	319	85	71	80
TAF5	1.332087228	2.64968E-07	up	390	311	309	103	73	112
TAF6	0.888786236	1.14571E-05	up	1160	1229	1132	406	383	566
TAF9B	-1.390101989	1.33881E-15	down	344	386	277	692	547	636
TAGAP	-8.968826395	6.98947E-12	down	0	0	0	78	45	76
TAGLN	-10.3702944	5.18451E-69	down	70	62	48	72274	14563	88901
TAGLN2	0.902658744	9.3106E-12	up	8054	9355	8237	3630	2601	3569
TAL1	-8.392376796	3.26797E-18	down	2	0	1	159	234	322
TANC2	1.841608556	4.12436E-09	up	1889	2019	1948	302	494	342
TANGO6	1.563536001	6.90138E-14	up	725	817	670	159	152	221
TANK	-1.112454571	1.76897E-06	down	430	436	459	564	643	827
TAP2	-4.28993029	0.045824631	down	1	0	1	0	16	11
TAPBPL	-9.150450103	5.08438E-17	down	2	0	0	248	233	329
TAPT1	-1.833839964	1.21354E-10	down	311	373	240	893	514	956
TARBP1	2.151386753	4.53563E-12	up	2958	3295	3001	327	548	586
TARBP2	0.81430823	2.0757E-06	up	402	396	410	169	151	169
TARDBP	0.689084821	3.17844E-06	up	5532	5880	5455	2476	2057	2935
TARP	-6.48336771	0.000135851	down	0	0	0	17	12	6
TARS	2.869729672	3.94984E-62	up	10984	11726	10504	1213	1075	928
TARS2	1.057393458	0.000499177	up	905	1171	995	337	424	268
TARSL2	-0.857710869	7.73258E-06	down	294	322	281	351	350	453
TAS1R3	4.77470068	3.6279E-07	up	56	43	54	0	1	3
TASP1	-1.404614148	0.002378854	down	120	159	108	249	116	376
TAT	4.733946055	0.021730034	up	4	11	6	0	0	0
TATDN2	0.810494121	1.8637E-05	up	1321	1550	1331	507	589	592
TBC1D1	-1.890421932	3.50033E-29	down	583	735	604	1840	1314	1934
TBC1D10A	-1.271643469	1.52798E-05	down	133	195	199	387	218	308
TBC1D10C	-4.59780989	4.10568E-06	down	1	6	1	83	19	37
TBC1D13	1.104935345	2.14366E-16	up	1338	1421	1417	454	435	490
TBC1D17	-2.198099016	1.26947E-17	down	223	165	206	617	534	806
TBC1D19	-0.614216662	0.015824776	down	181	156	139	163	176	177

TBC1D2	3.107923298	1.65667E-46	up	1321	1543	1537	101	115	146
TBC1D20	-0.887073358	4.18365E-13	down	675	742	679	920	824	1012
TBC1D22A	-0.892847778	1.06386E-05	down	221	275	248	296	318	363
TBC1D2B	-1.718603358	1.88505E-08	down	712	744	595	1107	1668	1973
TBC1D30	1.337487069	0.000114198	up	198	212	164	77	45	40
TBC1D31	1.845624424	1.83851E-19	up	351	407	349	68	73	77
TBC1D5	-1.085521317	1.22937E-06	down	740	847	685	982	994	1448
TBC1D8	1.144116962	7.03782E-07	up	1480	1619	1543	407	540	531
TBC1D9	-1.598992354	1.65375E-14	down	288	284	287	539	633	668
TBCB	-1.244184143	2.22251E-07	down	518	589	520	1230	666	872
TBCE	2.376406817	0.019355953	up	27	63	231	27	12	6
TBCEL	-1.891718116	2.94174E-32	down	215	216	209	579	551	554
TBCK	-3.185428024	1.40501E-39	down	59	100	92	549	496	572
TBL1X	1.188824255	0.000516289	up	3146	3456	3162	1129	554	1422
TBL1XR1	-0.627324863	0.011143885	down	2111	2276	1932	3080	1618	2325
TBL2	1.419095223	2.64956E-22	up	1358	1458	1364	440	309	367
TBL3	1.48004933	3.25502E-11	up	1059	1118	1035	364	214	247
TBRG1	-1.186055982	4.2634E-06	down	684	757	694	1211	769	1517
TBRG4	1.698500055	4.16719E-08	up	1935	1986	1916	394	538	323
TBX1	3.754430917	1.40664E-10	up	452	538	550	52	21	9
TBX15	-5.661095318	3.05788E-10	down	4	1	4	62	190	60
TBX18	-12.58711525	2.45225E-22	down	0	0	0	1201	418	854
TBX19	-1.548404425	0.005859691	down	39	40	37	99	31	118
TBX2	-3.854370248	9.7791E-53	down	55	71	74	749	708	586
TBX21	-6.494863016	5.1492E-06	down	0	1	0	18	27	23
TBX3	2.117137851	5.55401E-06	up	1112	1100	1112	97	267	164
TBXAS1	-7.551473567	6.05633E-23	down	3	1	1	195	166	312
TC2N	-4.464763658	0.001923238	down	1	1	1	18	2	29
TCAF2	0.789682402	0.008407459	up	90	106	99	37	39	45
TCAIM	1.008386574	0.000535911	up	1343	1320	1252	352	546	465
TCEA2	-0.644405086	0.006311347	down	371	430	386	574	325	432
TCEAL1	-2.085920595	8.34154E-05	down	439	497	448	1754	450	2109
TCEAL2	-12.2999633	4.16924E-06	down	0	0	0	1311	9	768
TCEAL3	-10.90478292	1.3225E-28	down	1	1	2	2239	479	2947
TCEAL4	-1.92375345	0.000593571	down	2421	2727	2468	8383	2011	10850
TCEAL5	-8.125910778	7.68012E-08	down	0	0	1	129	18	75
TCEAL6	-8.140049287	0.001230538	down	0	0	0	83	0	34
TCEAL7	-10.49061436	8.17243E-14	down	0	0	0	348	75	160
TCEAL8	-0.823173592	0.003585041	down	1017	1154	957	1840	882	1264
TCEANC	-1.547289244	2.96123E-05	down	43	41	32	110	61	72
TCEB3	0.630027077	0.016305738	up	1671	1801	1609	680	913	705
TCERG1	1.232056355	3.76481E-11	up	2660	2939	2627	922	606	984
TCF12	-0.882566179	2.10652E-05	down	1101	1227	1104	1534	1112	1889
TCF15	-7.688131602	1.57653E-05	down	0	0	0	12	54	10
TCF24	5.517840848	0.003838296	up	4	17	15	0	0	0
TCF25	-0.849737741	5.918E-09	down	1032	1128	1061	1578	1091	1485
TCF3	1.780076969	2.06372E-28	up	1568	1665	1668	333	346	331
TCF4	-6.894571815	1.24815E-78	down	59	34	26	2817	3196	4060
TCF7L1	-5.442570114	4.71303E-66	down	19	13	18	558	393	612
TCF7L2	-1.320998703	3.70656E-06	down	313	340	326	629	368	768
TCFL5	1.23162906	1.29125E-08	up	1276	1235	1148	472	325	315
TCN2	-3.240046867	1.33251E-11	down	83	99	111	430	406	1161
TCOF1	2.426746224	1.34403E-33	up	3824	4228	4021	605	374	635
TCP1	0.98648367	3.46888E-07	up	5729	6052	5317	2144	2166	1773
TCP10L	1.724443388	0.028200991	up	53	66	46	25	7	4
TCP11L1	1.184164742	0.003473398	up	1078	1167	1130	330	187	560
TCTA	-1.355351687	8.31369E-16	down	362	390	301	657	620	629
TCTEX1D1	-7.793444527	3.87609E-07	down	0	0	0	17	45	22
TCTN1	-0.609452666	0.000890754	down	321	383	330	353	376	385
TCTN2	0.858834372	0.001237199	up	309	326	292	96	117	149
TDG	1.297899959	1.61345E-18	up	1152	1212	1072	343	273	381
TDP1	1.879557358	1.78038E-25	up	616	673	569	133	116	109
TDRD1	-6.854593485	0.000587749	down	0	0	0	2	15	28
TDRD10	-8.885251901	4.53563E-12	down	0	1	0	112	138	107
TDRD3	-0.873437048	4.37799E-05	down	290	339	288	504	307	391
TDRD5	-5.107561087	0.030505452	down	0	0	0	5	1	8
TDRD6	-6.730339185	7.61444E-09	down	1	0	1	58	35	60
TDRD7	-0.592274441	0.00655018	down	381	364	332	464	286	416
TDRKH	1.655194793	2.40476E-08	up	461	424	444	73	101	125
TDRP	-8.182040376	6.86844E-12	down	0	0	2	91	186	127
TEAD1	2.098980308	4.24857E-10	up	8386	9770	8640	922	1608	1857
TEAD2	-0.883974876	7.60541E-05	down	414	462	443	521	635	556
TEAD4	2.21279288	3.07436E-07	up	662	630	627	72	145	69
TEC	1.586127251	4.36083E-10	up	187	180	174	41	42	45
TECPR1	-0.858992417	3.58622E-08	down	275	282	271	361	341	364
TECPR2	-1.487946087	5.57194E-09	down	232	234	245	385	433	600
TECTA	-4.577880292	5.26503E-06	down	1	3	0	21	22	24
TEF	-3.215365173	4.72657E-25	down	97	89	87	537	736	501
TEK	-1.968164547	2.70358E-09	down	286	290	309	543	859	1041
TEKT3	-2.549310827	0.032757571	down	3	3	0	10	6	9
TELO2	0.959303782	7.81907E-10	up	963	971	878	394	313	322
TENM1	-5.436736779	7.92872E-13	down	2	2	2	86	43	58
TENM3	-3.385693093	1.49972E-08	down	13	12	16	56	150	90
TENM4	-9.898892885	1.75243E-11	down	0	0	0	93	213	52
TEPP	2.937270208	0.018511558	up	15	11	18	1	3	0
TERF2IP	-1.391071405	2.93804E-14	down	1072	1000	978	2316	1474	1954
TERT	7.02322138	1.46711E-14	up	174	178	194	1	0	2
TESK1	-2.612448436	1.01114E-35	down	139	134	134	553	621	584
TESMIN	3.894384232	1.34254E-18	up	143	155	138	9	3	9
TESPA1	-7.411838097	4.03467E-07	down	0	0	0	29	13	26
TET1	-2.684211317	2.88146E-05	down	9	24	24	151	54	59
TET3	1.75787285	7.7371E-37	up	916	1014	908	202	174	221
TEX10	2.232172403	7.29969E-37	up	1089	1103	905	154	153	160
TEX19	6.676153166	5.00559E-07	up	53	55	47	0	1	0
TEX2	-0.6745229	0.008794618	down	860	854	756	748	1022	1002
TEX261	-0.600354901	0.00857491	down	1012	1106	1015	919	1145	1288
TEX264	-0.670956684	0.020548439	down	385	434	350	375	523	402
TEX30	2.256934705	5.77838E-14	up	699	814	693	123	126	75
TEX36	5.141430007	0.01002372	up	14	4	9	0	0	0
TF	-9.514910879	1.08163E-08	down	12	11	11	105	14893	1046
TFAM	1.866300532	1.53887E-33	up	2461	2601	2365	444	448	554

TFAP2A	4.143243378	5.67622E-11	up	962	1191	1093	46	9	80
TFAP2C	6.177075632	5.59918E-74	up	889	873	876	9	7	10
TFAP2E	-1.782790828	0.013838781	down	6	15	7	33	12	24
TFAP4	1.860736848	1.53128E-09	up	323	333	324	45	64	82
TFB2M	1.975516527	1.01706E-05	up	832	922	771	111	228	102
TFCP2	0.890843679	1.92992E-12	up	1201	1287	1226	530	432	463
TFCP2L1	-4.157463959	5.70708E-05	down	1	6	4	30	14	98
TFDP1	1.976910939	1.49363E-29	up	3706	4063	3675	714	723	614
TFDP2	-0.658083181	0.008860698	down	768	872	824	759	805	1201
TFEC	-3.751142027	4.87767E-10	down	18	13	13	114	66	252
TFF2	6.173114626	4.80716E-05	up	20	18	18	0	0	0
TFF3	-7.474820047	0.000316531	down	0	0	0	13	50	2
TFG	1.296544564	2.8036E-29	up	3407	3870	3555	1150	971	1010
TFPI	-5.136292378	4.95281E-32	down	42	87	60	911	1585	2148
TFPI2	-1.303187885	0.022083258	down	65	86	92	261	67	110
TFR2	5.775201404	8.97357E-29	up	319	353	262	3	7	2
TFRC	3.693183793	5.69484E-90	up	17984	18776	17198	1252	854	871
TGFB1	0.616082908	0.003759134	up	1811	1850	1897	982	614	1014
TGFB1I1	-3.317131247	4.51368E-18	down	184	203	222	1950	702	1777
TGFB3	-3.420427702	1.91044E-20	down	83	87	104	980	668	442
TGFBI	-1.535404863	3.36162E-07	down	1513	1545	1469	3296	3700	2198
TGFBR1	1.433574921	0.003086133	up	8051	9051	8093	3440	832	2567
TGFBR2	-3.333845298	5.70488E-24	down	1297	1362	1284	6041	9073	13011
TGFBR3	-1.262622536	0.007138848	down	1415	1436	1314	1184	2097	3813
TGFBR3L	4.07326066	1.65195E-06	up	96	108	96	11	1	1
TGIF2	1.479721483	3.95904E-10	up	744	774	777	254	174	159
TGM1	2.708903873	1.24067E-07	up	73	83	72	14	5	6
TGM2	-1.842942998	0.019838021	down	5411	6143	5388	5056	29572	5631
TGM5	-3.463604906	0.047081758	down	1	2	0	6	1	17
TGS1	1.053081423	2.37554E-06	up	816	851	747	231	292	297
THAP3	-0.874806497	0.001072385	down	211	188	156	307	206	214
THAP6	-1.376543362	4.99872E-08	down	239	232	198	350	360	526
THAP9	-1.17973233	3.73269E-05	down	66	73	52	113	101	92
THBD	-4.194013075	2.27367E-51	down	88	87	66	1381	918	844
THBS2	-15.6861332	2.07396E-33	down	0	0	0	11708	5004	4272
THBS3	-1.838365453	1.43198E-41	down	297	322	294	779	684	860
THEG	7.404496963	9.70198E-08	up	38	61	34	0	0	0
THEM4	0.774416742	0.001635489	up	663	632	636	253	303	239
THEMIS2	-10.03268653	4.98755E-15	down	0	0	1	391	191	229
THNSL2	-11.41512227	8.20103E-18	down	0	0	0	427	441	186
THOC2	0.892526694	0.00094071	up	3392	3540	3048	1550	799	1533
THOC3	1.197816447	0.0003029	up	515	538	514	225	153	108
THOC6	0.913071352	0.031259484	up	595	571	552	363	133	166
THOP1	1.849078655	3.4676E-20	up	1354	1310	1304	261	282	235
THRA	-0.710804412	0.027489491	down	1007	1018	1022	1140	755	1706
THRB	-4.594261557	2.65716E-17	down	83	126	94	2236	537	2578
THRSP	-11.67481859	1.01938E-12	down	1	4	3	263	14973	1495
THSD1	-2.506321627	2.82608E-13	down	52	48	36	148	209	185
THSD4	-1.284097751	0.006170788	down	813	907	770	2017	564	1855
THSD7A	-10.85428551	2.62353E-16	down	0	0	0	135	280	300
THSD7B	-5.732483129	8.38168E-09	down	5	1	1	180	53	40
THTPA	-0.920682427	7.95882E-05	down	167	241	198	250	274	284
THUMPD1	-0.760150426	0.003438944	down	913	936	847	1155	725	1409
THUMPD3	1.007450417	1.46281E-08	up	1354	1486	1288	485	511	452
THY1	-12.27770128	2.74869E-17	down	0	0	0	335	1211	295
TIA1	-0.798224097	2.5427E-07	down	1482	1752	1447	2105	1519	2189
TIAM1	-7.820430589	6.27397E-12	down	2	0	0	92	89	142
TIAM2	-2.258766542	2.12481E-07	down	23	19	20	61	52	100
TICAM1	1.671808649	9.92031E-09	up	638	722	640	204	91	158
TICRR	7.325132283	7.55605E-65	up	1255	1350	1223	11	5	1
TIE1	-10.90214329	1.246E-20	down	2	0	0	482	1183	998
TIGAR	1.34060615	9.91203E-09	up	584	550	513	178	157	126
TIGD1	1.130023221	0.005437584	up	153	188	155	85	35	43
TIGD2	1.706226747	0.0058392	up	257	260	265	31	99	33
TIGD3	3.31324812	0.001396269	up	17	32	22	0	2	3
TIGD6	-0.737441618	0.016143549	down	82	105	84	95	120	102
TIGD7	-2.497341312	7.56212E-21	down	59	58	51	236	167	277
TIGIT	-5.701531063	0.000165528	down	1	0	0	18	11	11
TIMELESS	2.926868166	2.93196E-30	up	1724	1821	1752	163	195	131
TIMM10	0.805197525	0.037169645	up	796	940	793	392	415	204
TIMM17A	1.881688592	1.59864E-08	up	3463	3751	3274	562	888	525
TIMM17B	1.770264352	4.70438E-12	up	1163	1323	1179	342	227	196
TIMM21	-1.347379798	0.004699034	down	241	240	194	285	610	284
TIMM23	1.347341224	8.81794E-05	up	2150	2259	2016	674	716	378
TIMM50	1.32528827	3.1775E-09	up	1345	1332	1338	378	423	328
TIMM8A	2.280143784	5.30749E-12	up	403	484	449	59	84	49
TIMP1	-1.717492618	2.31748E-17	down	3361	3506	3302	8204	8585	6769
TIMP2	-1.258110008	0.000857906	down	9410	10192	9264	18178	8243	23761
TIMP3	-5.042072062	2.9367E-31	down	1008	1051	1163	21496	12565	43197
TIMP4	-3.791504666	2.26909E-18	down	183	244	180	1306	2913	1533
TINAGL1	-2.05240202	1.05802E-13	down	1225	1275	1181	4232	2206	4606
TINF2	-1.45357817	3.89285E-18	down	365	333	350	784	566	705
TIPARP	1.941504047	1.33212E-11	up	2484	2540	2326	321	446	586
TIPIN	2.087861481	5.61175E-22	up	457	452	371	81	66	67
TJP3	7.276154008	4.17527E-11	up	140	153	142	1	0	1
TK1	5.19130735	2.4451E-20	up	2815	2968	2966	116	34	24
TK2	-2.436387394	5.86034E-37	down	245	314	256	943	1049	1117
TKFC	0.922533813	2.28997E-11	up	615	672	588	251	213	239
TKT	1.435238249	0.003370654	up	12124	13131	12561	2053	5199	2307
TLCD1	4.064567068	1.32408E-06	up	212	269	222	3	22	3
TLDC1	1.767071589	2.37954E-18	up	1063	1038	1111	283	177	217
TLE2	-2.398933023	8.10731E-17	down	139	154	146	451	655	513
TLE3	1.172100509	2.25366E-09	up	834	876	909	345	232	254
TLE4	0.751454683	0.002030263	up	665	794	675	242	315	335
TLN1	-2.303434829	3.68394E-07	down	2718	2967	2711	7751	4997	17311
TLN2	-3.304796903	1.77502E-39	down	161	159	200	1035	1130	1494
TLR1	-5.444001783	1.18519E-35	down	5	15	8	283	216	368
TLR10	-4.010677807	0.014213521	down	3	0	1	38	2	9
TLR2	-11.74701244	2.57241E-18	down	0	0	0	432	198	753
TLR3	-4.797019543	1.05934E-26	down	3	13	12	191	179	179
TLR4	-3.15016258	6.11803E-33	down	190	245	195	1281	939	1784

TLR5	-6.042874635	1.16603E-17	down	4	1	4	164	73	197
TLR6	1.149808794	1.03523E-05	up	320	336	289	118	67	121
TLR7	-9.122068757	1.12214E-11	down	0	0	0	90	40	93
TLR8	-8.884642677	7.72848E-10	down	0	0	0	56	27	107
TLR9	2.497220901	0.001362267	up	135	132	145	22	2	30
TLX3	8.33857001	1.34991E-10	up	77	103	73	0	0	0
TM2D1	-1.149214234	0.000111643	down	438	495	396	875	421	832
TM4SF1	1.759867178	3.49216E-19	up	18865	21368	18451	5252	3550	3547
TM4SF18	-8.599236414	3.6907E-19	down	1	2	1	173	572	311
TM4SF19	6.453031586	1.07244E-05	up	23	22	23	0	0	0
TM4SF4	2.949000451	3.29892E-06	up	66	95	107	5	14	5
TM6SF1	-4.843286324	1.59473E-21	down	20	7	8	216	183	326
TM6SF2	-6.28584648	5.09163E-05	down	0	1	0	19	29	10
TM7SF2	-3.373208009	6.55783E-05	down	61	40	51	116	782	153
TM9SF2	-0.630341578	1.60101E-08	down	2531	2880	2515	3176	2428	3144
TMA16	1.020495356	2.00207E-06	up	603	624	574	268	156	213
TMBIM1	-2.397671085	8.91493E-40	down	1143	1253	1189	3889	4364	5116
TMBIM4	-2.757376677	2.42339E-55	down	280	328	241	1579	1095	1420
TMC2	-7.281416918	2.52881E-05	down	0	0	0	10	37	11
TMC5	6.375753401	3.02528E-45	up	539	639	589	3	9	3
TMC7	2.002006236	7.31643E-05	up	316	367	350	45	96	36
TMC8	-1.98282457	9.41217E-13	down	49	44	50	143	136	122
TMCC3	-3.720818357	1.44618E-33	down	33	41	23	262	297	342
TMED3	1.324928741	4.82949E-07	up	2479	2648	2489	1001	605	568
TMED8	0.780652239	6.04877E-06	up	972	1124	1015	395	386	505
TMED9	1.200520662	1.94607E-20	up	4467	4674	4411	1400	1194	1611
TMEM100	-10.52811242	1.03524E-13	down	1	0	0	127	576	383
TMEM102	2.341399211	2.00512E-26	up	314	336	330	52	37	49
TMEM105	7.083128554	3.75065E-07	up	39	31	35	0	0	0
TMEM106A	-1.087702993	0.000203619	down	59	62	73	98	79	118
TMEM106B	-0.598029952	0.000343804	down	962	1103	923	1071	878	1275
TMEM106C	1.438716293	0.00701405	up	4127	4374	4107	1605	353	1464
TMEM107	0.889355278	5.57225E-06	up	359	402	363	167	109	158
TMEM108	-8.255963655	4.48959E-09	down	0	0	0	51	44	24
TMEM110	-2.301169304	2.58503E-07	down	121	136	127	674	195	514
TMEM116	-1.003037537	7.59827E-05	down	88	88	82	129	126	111
TMEM117	-0.949455101	2.05049E-06	down	111	130	119	172	141	182
TMEM119	-6.900327235	6.49926E-18	down	1	7	1	160	364	203
TMEM120A	-1.228842689	5.92508E-10	down	401	414	450	817	674	617
TMEM120B	0.864110691	0.013500449	up	526	623	557	159	281	212
TMEM123	1.301652269	4.40387E-15	up	8066	9144	7917	2242	2091	2916
TMEM126B	0.763960934	3.07441E-08	up	1838	1952	1661	825	725	726
TMEM127	-0.602160149	3.62594E-06	down	1172	1330	1206	1301	1279	1407
TMEM128	-2.745890581	2.33314E-28	down	95	116	100	627	349	522
TMEM129	-1.391232038	5.78959E-13	down	307	371	289	564	619	603
TMEM130	-9.24220013	1.53204E-11	down	0	0	2	469	51	386
TMEM132A	2.926662814	3.83873E-17	up	1376	1505	1252	105	174	98
TMEM132B	-6.190315135	2.36016E-09	down	2	11	0	420	83	172
TMEM132C	-9.668153932	6.57418E-09	down	1	0	0	16	350	226
TMEM132E	-4.239327424	0.017363348	down	0	0	2	4	16	6
TMEM134	-1.426595045	2.06952E-10	down	131	126	106	258	225	209
TMEM135	-0.700182502	0.025618605	down	807	873	848	690	1117	1071
TMEM138	1.065401523	4.24253E-14	up	980	1023	895	366	272	350
TMEM139	5.099181203	1.28776E-42	up	502	606	493	15	13	5
TMEM140	-5.902069478	2.08049E-36	down	8	23	14	428	734	703
TMEM141	0.925379934	0.005818864	up	1103	1173	988	573	378	271
TMEM143	-2.043505571	1.28428E-05	down	44	37	49	93	176	104
TMEM144	-1.018981106	2.6519E-07	down	188	214	159	279	225	305
TMEM150A	-1.80895709	2.53203E-09	down	70	68	103	238	162	207
TMEM150B	-8.053089083	1.97017E-08	down	0	0	0	22	45	35
TMEM150C	-10.24531054	4.655E-11	down	0	0	0	35	274	144
TMEM151A	5.061567138	6.42772E-06	up	133	146	128	9	0	0
TMEM156	3.178457745	5.94745E-10	up	229	245	222	12	30	11
TMEM158	3.574614142	4.50717E-12	up	198	219	253	9	22	8
TMEM159	-2.939266919	7.62349E-35	down	145	134	128	876	518	855
TMEM161A	0.984348817	2.8981E-06	up	584	596	534	188	221	202
TMEM161B	0.712416854	4.78205E-06	up	591	609	537	256	217	282
TMEM167B	-0.948252802	1.66508E-12	down	699	876	741	1080	930	1164
TMEM168	-0.9466594	6.21066E-09	down	487	483	528	779	619	661
TMEM169	-6.546530849	3.06962E-05	down	0	0	0	13	9	15
TMEM173	-2.401404345	1.76378E-40	down	258	291	211	989	786	1084
TMEM175	-1.961604944	9.29147E-24	down	92	107	93	263	241	304
TMEM176A	-8.573760284	1.52695E-16	down	0	0	5	184	631	518
TMEM176B	-9.654348527	1.6504E-22	down	2	0	2	267	1046	933
TMEM177	1.818085397	0.00016978	up	267	277	270	53	79	28
TMEM178A	-5.746405597	6.75223E-12	down	3	1	4	190	52	74
TMEM178B	2.435719723	0.003064667	up	418	430	461	21	120	20
TMEM179B	-1.464529562	1.70442E-13	down	183	207	197	467	298	395
TMEM183B	1.182243296	0.010822225	up	82	114	86	17	33	37
TMEM184A	7.98676725	1.92552E-36	up	687	732	717	1	1	4
TMEM185A	-0.758179178	5.54747E-05	down	195	236	175	264	211	253
TMEM185B	1.305549925	5.14363E-09	up	1538	1725	1500	411	515	426
TMEM186	1.039802895	7.14533E-08	up	445	440	415	156	153	139
TMEM189	1.088315699	0.014682803	up	583	522	543	286	169	99
TMEM190	4.672558532	0.020535021	up	5	9	6	0	0	0
TMEM192	-0.814398178	0.016134573	down	394	418	367	336	461	671
TMEM198	-1.717564429	0.046501582	down	10	4	19	36	28	14
TMEM2	2.373496127	1.87914E-06	up	4101	4782	4202	428	955	339
TMEM200A	-9.824084186	2.37525E-12	down	0	0	0	89	184	70
TMEM200B	-1.1947759	0.002280894	down	259	269	249	556	208	531
TMEM200C	-5.317554379	0.016714471	down	0	0	0	4	9	2
TMEM201	1.777628953	7.48993E-05	up	537	663	537	107	173	69
TMEM204	-9.678888845	9.88461E-35	down	4	0	0	716	666	958
TMEM205	-0.921561744	3.8782E-05	down	382	431	456	689	538	482
TMEM206	1.981290981	6.49497E-09	up	467	486	388	123	57	64
TMEM209	1.189579661	1.97846E-09	up	912	1042	891	259	309	311
TMEM217	-1.5906344	0.044818841	down	7	7	3	10	13	13
TMEM219	-1.123732763	2.37332E-07	down	795	847	824	1679	987	1190
TMEM220	-10.6543428	1.15167E-17	down	0	0	0	249	186	199
TMEM222	-0.8861278	9.96638E-07	down	333	344	319	531	358	427
TMEM229B	-6.112477153	1.13876E-06	down	0	2	0	42	20	37

TMEM230	-2.092796338	2.73939E-74	down	734	774	699	2427	2073	2187
TMEM232	-3.337128764	0.001515326	down	9	3	0	23	30	33
TMEM233	-7.482192351	1.32252E-07	down	0	0	0	28	23	19
TMEM236	-3.376561273	0.000504237	down	7	7	3	10	34	81
TMEM237	-1.909920926	6.04869E-07	down	353	331	295	1024	437	1217
TMEM240	-3.35937391	6.75841E-07	down	3	5	10	62	27	45
TMEM241	0.841687117	0.000219057	up	184	177	187	78	65	75
TMEM242	-1.906851184	4.4758E-29	down	194	218	164	543	434	560
TMEM25	-5.464046038	2.7453E-20	down	7	12	9	390	111	400
TMEM255A	-5.200939524	3.23101E-09	down	1	1	6	75	34	107
TMEM26	-4.688047457	0.000629934	down	0	0	3	23	22	10
TMEM260	-1.699986508	3.57056E-12	down	117	182	138	375	256	381
TMEM261	-1.371743487	3.19314E-05	down	419	451	362	981	776	495
TMEM263	0.997526788	0.000165528	up	3085	3460	2981	1586	797	1049
TMEM265	0.82974153	0.005138956	up	211	238	197	116	58	87
TMEM266	3.214598937	0.001146855	up	28	24	28	0	4	2
TMEM267	1.607562259	6.94429E-18	up	762	972	731	219	152	205
TMEM27	2.919892678	9.30574E-11	up	124	124	163	20	10	9
TMEM30A	-1.201372638	0.000206191	down	2322	2571	2200	3891	2311	5575
TMEM30B	-10.76144093	4.89002E-14	down	0	0	2	1402	87	1123
TMEM33	1.218653453	1.56729E-16	up	1849	2011	1837	687	515	541
TMEM35A	-9.788436236	3.2679E-09	down	0	0	0	225	13	126
TMEM35B	-1.997885554	1.49594E-19	down	162	154	169	579	367	444
TMEM37	-1.397826922	2.26428E-06	down	126	133	117	194	275	225
TMEM38A	1.628834155	7.84783E-07	up	431	416	399	143	62	86
TMEM38B	1.440561861	2.23239E-08	up	947	977	799	188	261	256
TMEM39A	1.044409873	5.32733E-08	up	1544	1619	1445	673	416	511
TMEM40	4.66882014	6.05992E-08	up	74	55	51	2	3	0
TMEM41B	1.376183153	1.06187E-14	up	1110	1168	1147	379	239	328
TMEM43	-1.113840398	0.003495875	down	1911	2023	2003	3658	1504	4197
TMEM45A	0.749580494	5.75636E-06	up	899	1038	915	433	314	465
TMEM45B	4.725459186	0.002409394	up	13	13	14	0	1	0
TMEM47	-3.193668412	1.44084E-08	down	745	849	858	6886	1475	8172
TMEM50A	-0.616708248	2.47155E-07	down	1740	1812	1657	1956	1618	2121
TMEM50B	-2.092652987	1.76163E-07	down	16	20	13	49	46	53
TMEM52B	4.983695059	7.45398E-17	up	151	144	144	6	0	4
TMEM53	-1.693559613	0.001936004	down	37	45	46	80	147	58
TMEM54	1.139967025	0.00017631	up	388	421	446	188	117	102
TMEM55A	-2.152062009	3.46753E-20	down	102	137	147	477	325	425
TMEM57	-0.768994164	4.25586E-07	down	415	454	403	497	464	579
TMEM59	-1.55988906	2.19489E-23	down	2225	2317	2341	5690	3774	5090
TMEM59L	-1.978818955	8.70911E-07	down	22	32	40	99	92	71
TMEM63A	-0.604029417	0.002151602	down	676	839	776	800	674	1009
TMEM65	0.874736228	0.004314842	up	1088	1161	1102	426	528	324
TMEM69	1.366229051	2.09E-05	up	960	1086	1070	228	365	249
TMEM71	-7.295432732	5.35959E-13	down	2	0	1	77	89	172
TMEM74	-4.240682024	0.000201836	down	8	0	1	58	23	45
TMEM74B	-1.952884427	0.001391293	down	10	13	13	20	43	34
TMEM80	-3.498733758	3.02182E-22	down	31	43	33	353	169	350
TMEM86A	-3.678491656	5.24113E-14	down	19	18	10	115	99	217
TMEM87A	-0.885102075	1.52111E-06	down	966	1006	950	1436	950	1488
TMEM88	-7.338356726	1.12961E-17	down	3	0	2	109	239	220
TMEM8A	0.688121085	5.81523E-09	up	1175	1301	1172	599	461	553
TMEM8B	-3.554879376	6.0545E-37	down	42	52	47	458	269	464
TMEM9	0.935730372	0.000249011	up	1606	1707	1597	827	417	606
TMEM91	-3.555425701	3.28374E-20	down	17	21	20	209	101	182
TMEM92	2.81763542	0.000249324	up	89	63	102	8	15	2
TMEM97	3.152681669	7.95406E-21	up	1543	1479	1320	84	152	104
TMEM98	-12.93029226	2.00871E-24	down	0	0	0	1392	602	1129
TMEM99	0.823134625	0.00545839	up	391	422	355	222	120	131
TMIGD3	-9.598644639	4.82312E-11	down	0	0	0	35	124	139
TMLHE	-0.6360379	0.019692711	down	173	197	176	161	210	227
TMOD1	-2.658987237	5.30551E-61	down	233	279	285	1172	1126	1273
TMOD2	-4.104770496	3.24168E-30	down	62	70	55	510	691	1077
TMOD3	1.778813689	1.15281E-12	up	4222	4523	4025	716	746	1188
TMPO	2.298789357	4.23736E-31	up	4930	5493	4761	937	539	738
TMPRSS13	-3.919089688	0.017306295	down	0	2	0	9	7	5
TMPRSS3	11.75487683	7.97113E-22	up	872	986	834	0	0	0
TMPRSS4	5.171769011	0.000635452	up	18	24	13	0	1	0
TMPRSS9	4.163336618	0.001023045	up	67	57	71	0	0	8
TMSB15A	-3.130267551	0.000740211	down	2	5	7	54	10	26
TMSB4X	-0.918662084	0.013759494	down	18321	19440	17337	39343	14584	21633
TMSB4Y	-8.167400915	0.00101625	down	0	0	0	71	0	48
TMTC4	0.670784757	0.044184812	up	345	288	310	106	163	148
TMUB1	1.193262627	8.50566E-13	up	994	1089	1066	396	270	318
TMX1	0.749233911	3.55144E-07	up	1362	1738	1420	638	562	708
TMX3	-1.537131677	1.74196E-47	down	613	683	602	1390	1142	1388
TMX4	-2.4229267	6.17164E-56	down	427	486	397	1603	1469	1918
TNC	-6.26691514	1.04601E-20	down	38	45	58	920	4712	1687
TNFAIP1	0.800090909	0.001407672	up	2001	2182	1934	832	953	679
TNFAIP2	0.693563239	0.006619197	up	3293	3752	3389	1489	1770	1260
TNFAIP8	0.640255022	0.03297343	up	887	879	775	339	283	547
TNFAIP8L2	-9.687644109	1.66508E-12	down	0	0	0	185	60	86
TNFAIP8L3	-4.548114687	3.37968E-19	down	15	16	25	496	156	307
TNFRSF10A	2.771776775	1.00406E-29	up	421	403	386	50	41	35
TNFRSF10B	1.65657915	5.8557E-08	up	2923	3365	2985	519	571	1005
TNFRSF10D	1.11721234	1.13257E-09	up	552	582	538	208	177	162
TNFRSF11B	-4.740925097	1.59989E-09	down	84	108	73	3401	226	1627
TNFRSF14	-6.179561249	0.000185453	down	0	1	0	13	32	8
TNFRSF1A	-0.69766595	0.004161921	down	1641	1593	1570	1759	2107	1610
TNFRSF1B	-2.511263467	8.57543E-36	down	190	218	218	759	868	893
TNFRSF25	-3.320942747	5.79332E-18	down	13	23	19	159	89	146
TNFRSF4	-6.696114141	5.83729E-05	down	1	0	0	15	50	10
TNFRSF8	-3.796157075	0.00029195	down	5	9	0	20	54	59
TNFSF10	-10.43353563	5.8264E-30	down	0	1	2	826	774	1365
TNFSF12	-12.07292632	1.30519E-19	down	0	0	0	869	238	634
TNFSF13	-1.585895018	0.004342182	down	47	49	42	102	40	160
TNFSF13B	-6.163165002	5.64775E-13	down	4	3	2	121	257	61
TNFSF15	2.290394139	0.030094449	up	35	37	53	10	8	0
TNFSF4	-1.221137199	0.00304647	down	24	37	26	62	41	41
TNFSF8	-9.498563097	2.64248E-13	down	0	0	0	123	62	103

TNFSF9	6.268385043	9.98215E-81	up	1242	1345	1206	18	11	6
TNIK	-10.32223987	7.97282E-15	down	0	0	0	97	185	214
TNK1	2.406763237	3.2699E-10	up	272	343	262	62	23	34
TNMD	-5.747029241	5.62659E-06	down	0	0	2	22	28	26
TNN	-5.897236035	0.00144162	down	1	0	0	3	29	11
TNNC2	-3.667589663	0.000869749	down	6	3	1	65	9	20
TNNI2	-6.500011596	0.001282224	down	0	0	0	28	3	6
TNNT3	-8.293326679	1.56949E-12	down	1	1	0	108	201	125
TNPO1	1.766906766	1.99881E-22	up	10790	11275	10710	1977	2201	2645
TNPO3	1.437254313	2.12338E-53	up	3011	3309	2979	870	721	852
TNRC18	0.698166999	0.033318105	up	3086	3564	3433	1479	897	2112
TNRC6B	-0.919878571	0.000289664	down	538	551	594	673	601	1006
TNRC6C	-4.224123868	3.77075E-28	down	39	56	40	894	348	579
TNS1	-4.525832815	2.96352E-18	down	1745	1977	1719	29412	10227	52095
TNS2	-3.613549953	7.61652E-35	down	405	452	443	3434	2558	5457
TNS4	9.925094659	3.72786E-30	up	1371	1401	1329	1	2	0
TOB1	1.275803735	2.14144E-05	up	1939	2124	1880	778	564	399
TOB2	-0.728957683	0.001776786	down	939	1048	862	936	1213	1167
TOE1	0.973572032	0.000169797	up	377	416	393	194	116	122
TOLLIP	-1.482921331	3.60215E-25	down	382	408	386	773	761	795
TOM1	-0.701011322	0.014504589	down	374	431	387	374	541	440
TOM1L1	1.709476287	1.17326E-05	up	1180	1282	1093	390	127	274
TOM1L2	-1.596200639	1.85869E-13	down	481	562	572	981	1110	1368
TOMM20	0.824016996	3.72185E-05	up	7834	8815	7252	4006	2358	3318
TOMM34	2.724797836	1.12596E-82	up	2075	2283	1985	227	194	262
TOMM40	2.329363256	1.27042E-12	up	2357	2366	2213	343	405	217
TONSL	3.133095995	6.74819E-30	up	457	630	499	43	49	35
TOP1	1.830087197	1.55695E-20	up	4747	4706	4518	927	1012	831
TOP1MT	0.709739431	0.014385163	up	406	402	437	213	191	135
TOP2A	6.396738696	1.28957E-19	up	13390	14304	12620	243	79	24
TOP2B	0.59722468	0.000673688	up	5071	5299	4705	2364	1882	2878
TOP3A	1.07245403	6.64129E-09	up	857	927	865	264	299	328
TOP3B	1.151274264	4.12998E-05	up	500	544	468	127	139	218
TOPBP1	2.156283341	2.53957E-80	up	3200	3308	2988	512	464	538
TOR1AIP1	-0.861234794	0.002358773	down	1680	1933	1703	2692	1401	2880
TOR1AIP2	1.397083018	3.21734E-16	up	2593	2740	2502	872	551	708
TOR1B	1.060743399	1.69013E-09	up	1176	1249	1110	449	302	463
TOR2A	1.540631067	4.93128E-12	up	406	353	413	95	92	100
TOR3A	1.398885297	7.3552E-10	up	1376	1471	1275	409	396	297
TOR4A	3.635294912	1.2705E-81	up	2763	2810	2821	189	156	135
TOX	0.916916226	0.017848478	up	71	100	74	25	35	31
TOX3	7.5187134	6.81924E-09	up	75	112	92	0	0	1
TOX4	-0.660752987	3.66075E-06	down	1049	1065	878	1215	957	1199
TP53	0.633572047	0.001277943	up	902	969	898	513	392	364
TP53BP1	1.144967494	9.77936E-05	up	2455	2617	2398	629	981	747
TP53I11	-1.998813794	2.79432E-10	down	256	250	260	542	891	707
TP53I13	-1.043320667	4.29426E-06	down	336	380	369	631	521	433
TP53INP1	-10.36224867	1.76804E-28	down	0	0	3	679	860	1270
TP53INP2	-2.538593633	3.82544E-15	down	118	181	119	589	667	435
TP63	2.293536898	0.003850395	up	95	63	86	6	23	5
TP73	7.613213934	1.40133E-08	up	48	62	43	0	0	0
TPBGL	3.017614094	0.01645581	up	21	16	10	0	3	1
TPCN1	-0.880524413	0.000131641	down	1119	1191	1112	1767	1012	1760
TPD52	4.332164283	6.83E-57	up	4615	4997	4431	241	126	133
TPD52L1	-1.042482129	2.41931E-10	down	436	478	446	793	583	620
TPGS1	-2.22268608	8.7809E-25	down	61	60	56	216	169	204
TPH1	-10.03183783	6.74411E-08	down	0	0	0	160	4	267
TPI1	1.934135023	3.64806E-12	up	17557	18842	17098	3148	4036	2593
TPM1	-2.704121002	2.43404E-16	down	3695	3913	3622	13552	22890	14335
TPM2	-3.459118688	5.1903E-07	down	4064	4262	3967	40310	5574	54266
TPM3	1.933915878	3.18037E-38	up	11824	13101	11751	2248	2338	2188
TPMT	1.413813598	2.8523E-11	up	1325	1587	1301	355	413	342
TPO	-5.897737918	2.82774E-05	down	0	0	2	15	21	50
TPP1	-0.716313534	0.000760948	down	1857	2053	1907	2069	1840	2914
TPPP	-0.976592172	0.00493042	down	130	141	157	235	118	257
TPPP3	-7.439483964	1.78251E-24	down	2	2	1	241	200	174
TPR	0.626173332	0.011310721	up	5117	5611	5196	2195	1890	3296
TPRA1	1.440105572	1.34997E-10	up	1209	1450	1281	456	281	300
TPRG1	-6.708241674	2.25546E-12	down	3	7	2	91	587	145
TPRG1L	-1.025040536	1.25482E-15	down	749	749	668	1077	919	1143
TPRKB	0.893352452	3.17388E-06	up	903	933	795	368	339	295
TPRN	2.540812448	1.70578E-28	up	635	707	622	95	81	63
TPRX1	7.015696075	1.41236E-06	up	46	27	27	0	0	0
TPSAB1	-8.257600086	1.14568E-08	down	2	0	0	243	21	195
TPSB2	-8.288316078	8.40912E-09	down	0	2	0	223	22	211
TPST2	-1.281614418	1.13691E-13	down	292	340	324	530	554	559
TPT1	-2.475310282	3.4592E-33	down	13983	15138	14023	73665	49597	47883
TPX2	5.81952043	2.26308E-23	up	8095	8894	7922	126	150	29
TRA	-3.967362358	4.29631E-09	down	1	4	7	53	38	43
TRABD	0.969047794	4.95904E-12	up	776	915	839	337	281	300
TRABD2A	5.338695916	7.05943E-49	up	638	728	761	11	18	8
TRADD	-0.937909437	5.26447E-05	down	216	186	205	339	240	253
TRAF1	-4.294896645	1.29224E-14	down	37	41	43	1088	248	406
TRAF2	3.092699586	1.10545E-20	up	1652	1789	1665	199	138	88
TRAF3IP3	-5.854315796	2.36115E-12	down	1	0	5	104	53	96
TRAF4	3.379081217	7.42382E-13	up	1625	1641	1685	156	132	47
TRAF6	-0.648401356	0.001791108	down	338	402	373	461	306	483
TRAF7	2.063442595	4.8855E-57	up	3921	4048	4206	694	655	721
TRAFD1	1.16839945	1.33077E-17	up	1712	1820	1716	528	523	606
TRAIP	2.748111585	4.29871E-21	up	240	243	231	34	19	23
TRAK2	-2.109785692	9.86277E-25	down	805	824	730	2032	2318	2860
TRAM1L1	-7.501639152	4.07063E-08	down	0	1	0	66	41	32
TRANK1	-2.708297751	8.78854E-14	down	192	234	161	1116	477	1182
TRAP1	2.130405838	1.08889E-10	up	5390	6038	5353	763	1195	701
TRAPPC11	-1.803781916	1.14015E-25	down	279	305	249	737	548	792
TRAPPC3L	-4.800115875	0.030690289	down	0	0	0	5	3	3
TRAPPC6A	-1.195859303	0.000247443	down	64	92	71	156	116	96
TRAPPC6B	-1.520998386	1.87995E-16	down	362	452	383	989	636	830
TRAPPC9	-0.799558583	0.001106845	down	208	226	223	227	284	296
TRAT1	-5.448724392	0.00496958	down	0	0	0	4	6	7
TRB	-5.39170022	0.008816206	down	0	0	0	3	8	5

TRDMT1	-1.322842478	4.33127E-05	down	137	144	111	248	147	311
TREM1	-1.696378214	0.020620391	down	23	15	13	51	50	15
TREM2	-10.46031287	1.14E-12	down	0	0	0	388	66	119
TREML1	-5.580113599	0.003945221	down	0	0	0	5	4	10
TRERF1	1.237131283	5.2003E-09	up	535	584	532	205	153	140
TREX2	-4.548740514	0.000118846	down	4	5	2	153	7	31
TRG	-4.856669367	5.47307E-11	down	0	5	4	62	43	81
TRHDE	-7.779021416	1.03679E-10	down	0	2	0	107	55	154
TRIAP1	0.681354688	0.039419515	up	596	663	545	351	266	179
TRIB2	-7.142145556	1.0008E-102	down	11	10	14	1188	1224	1082
TRIB3	4.661033599	4.8338E-100	up	1365	1477	1313	51	33	33
TRIL	-9.667987489	2.48791E-13	down	0	0	0	79	134	100
TRIM11	1.471828763	5.94217E-18	up	744	752	691	218	147	199
TRIM13	-1.784045389	3.66537E-12	down	247	211	224	727	398	570
TRIM14	1.468290112	1.07188E-05	up	1314	1437	1233	311	214	512
TRIM16	2.438092734	1.78826E-19	up	1288	1400	1276	162	210	140
TRIM16L	3.147760566	4.59518E-41	up	1365	1408	1341	86	107	136
TRIM17	-5.148788111	0.045388275	down	0	0	0	2	10	1
TRIM2	-1.70125622	6.02718E-13	down	279	308	269	561	568	851
TRIM21	-0.924627338	0.001547832	down	173	285	229	400	237	293
TRIM22	-9.622849337	1.68003E-50	down	0	3	5	1860	892	1811
TRIM23	-1.414733002	1.9864E-10	down	218	264	205	483	315	513
TRIM24	1.093095885	0.005047666	up	996	1105	997	267	477	260
TRIM25	0.747124956	8.89342E-05	up	2343	2599	2377	902	1064	1110
TRIM28	0.77981685	6.20062E-07	up	3388	3529	3335	1700	1122	1455
TRIM29	7.735254287	9.75666E-48	up	5273	5743	5445	11	7	38
TRIM3	-0.74028236	0.021912162	down	301	311	306	489	217	405
TRIM32	0.865931841	0.00070221	up	796	777	731	316	205	389
TRIM33	1.041277389	7.30435E-05	up	2609	2743	2567	1113	575	1092
TRIM37	0.713421219	0.000998073	up	979	1200	996	386	454	526
TRIM38	-1.174955122	0.000721251	down	608	652	540	1217	516	1215
TRIM4	-1.838197873	1.44742E-11	down	225	217	229	761	381	592
TRIM5	-0.641565302	2.25161E-05	down	450	508	412	574	429	519
TRIM50	-5.27158461	0.00104357	down	0	0	1	14	6	10
TRIM52	-1.108712788	2.69383E-05	down	301	287	250	394	329	571
TRIM58	-6.725501166	1.51199E-05	down	0	0	0	11	16	14
TRIM59	2.482855789	2.25269E-06	up	692	805	705	150	30	109
TRIM61	-7.740548911	1.29257E-07	down	0	0	0	25	38	19
TRIM65	1.374903159	1.7705E-10	up	922	966	883	326	221	215
TRIM66	-1.455898746	1.74847E-06	down	156	186	180	401	208	425
TRIM68	-0.916623867	7.57185E-06	down	145	188	138	212	197	220
TRIM7	1.030984275	0.007081582	up	201	215	157	99	41	62
TRIM8	-1.546933132	1.36451E-10	down	504	611	521	988	1290	1068
TRIM9	-7.356968155	4.48828E-07	down	1	0	0	19	44	61
TRIML2	8.984011852	1.6715E-12	up	112	140	142	0	0	0
TRIO	1.733885227	1.33163E-11	up	6407	7086	6698	1225	1125	1990
TRIOBP	-1.109739462	2.10914E-12	down	803	964	936	1331	1358	1438
TRIP11	-0.595745671	0.011208286	down	828	1055	920	868	864	1277
TRIP13	7.285886183	1.1947E-163	up	4177	4799	4068	20	25	14
TRIP6	1.176704407	0.000510429	up	2692	2992	2880	1327	516	906
TRIQK	-0.909439838	1.19569E-05	down	417	530	417	531	588	689
TRIT1	1.573607428	4.62301E-28	up	768	900	829	209	172	216
TRMO	-0.81839527	0.0058392	down	149	163	149	257	173	149
TRMT1	0.659597584	3.59028E-05	up	755	949	774	415	342	355
TRMT10B	-2.063509829	3.16271E-21	down	80	98	79	255	210	300
TRMT10C	1.257462726	3.44144E-12	up	1097	1284	1067	365	349	304
TRMT2B	0.959500348	2.08951E-05	up	674	773	732	219	265	308
TRMT44	-0.623161905	0.00256346	down	132	176	147	175	151	171
TRMT6	0.97666977	7.30079E-08	up	468	604	511	219	171	181
TRMT61A	0.628293058	0.000152571	up	446	467	397	220	169	215
TRMU	0.653911884	0.000141682	up	684	661	675	329	297	286
TRNT1	0.676569827	0.003382864	up	798	939	810	318	408	394
TRO	-10.86724219	5.52433E-18	down	0	0	0	316	179	246
TROAP	5.695259545	1.62224E-13	up	1249	1352	1350	45	7	4
TROVE2	0.644596264	0.000642321	up	2114	2210	1894	1117	700	1036
TRPC1	-1.488939777	4.23266E-05	down	254	368	269	852	333	620
TRPC4	-8.109161996	6.30839E-09	down	1	0	0	122	38	56
TRPC6	-9.922756198	3.22085E-15	down	0	0	0	125	108	149
TRPM3	-8.819737922	3.50073E-10	down	0	0	0	89	27	66
TRPM4	-2.209336931	2.81265E-14	down	161	142	115	429	343	614
TRPM6	-2.266263031	1.58016E-05	down	10	22	14	37	53	65
TRPV2	-2.216937234	1.45642E-07	down	38	61	70	198	222	130
TRPV3	-5.375496856	8.85546E-05	down	1	0	2	19	56	10
TRPV4	-7.027337983	2.75331E-07	down	1	0	0	40	22	39
TRRAP	1.148622021	4.43605E-07	up	2255	2555	2357	668	631	1006
TRUB1	1.368208664	4.33875E-05	up	974	1036	970	228	357	218
TRUB2	1.379647688	0.000659669	up	1161	1178	1054	265	433	204
TSC22D1	-0.650851812	5.37777E-08	down	2853	3075	2845	3488	3134	3145
TSC22D3	-3.329006953	7.06724E-14	down	721	764	792	3062	4038	9291
TSC22D4	1.09467893	1.52744E-05	up	1290	1312	1297	386	506	389
TSEN2	0.990043403	3.31583E-05	up	406	447	458	132	166	168
TSEN54	1.867832217	4.45998E-17	up	550	542	589	137	96	96
TSGA10	-1.031128542	0.026356236	down	33	36	26	32	53	51
TSHR	-6.604729127	0.001217684	down	0	0	1	3	60	5
TSHZ1	-2.994720403	4.2533E-43	down	88	74	83	465	389	543
TSHZ2	-1.385273462	0.006234922	down	388	458	434	537	429	1452
TSHZ3	-9.780343302	9.31141E-15	down	0	1	0	185	233	249
TSKS	-2.393365462	0.002154271	down	7	3	6	15	27	17
TSLP	-5.052837697	1.53469E-09	down	2	4	0	59	46	35
TSN	0.705252178	2.70744E-06	up	2736	3082	2651	1448	976	1289
TSNARE1	-2.653172216	4.82846E-18	down	44	65	57	222	196	326
TSNAXIP1	-2.255119867	0.000681227	down	11	5	5	23	19	30
TSPAN1	1.526181501	0.008838067	up	90	149	110	46	10	32
TSPAN10	3.669083914	1.21067E-23	up	187	243	233	16	12	9
TSPAN11	-9.498208502	2.87448E-11	down	0	0	0	52	149	72
TSPAN13	1.989387587	0.000938796	up	1802	1847	1729	128	543	248
TSPAN14	-1.284298607	0.00707227	down	547	553	572	508	1388	912
TSPAN18	-2.055857687	0.000243075	down	195	197	234	315	1012	450
TSPAN19	6.911379781	1.71562E-07	up	68	59	55	0	0	1
TSPAN2	-7.406783821	1.91279E-19	down	9	8	3	1260	141	1107
TSPAN3	-1.704830526	8.14628E-08	down	1201	1311	1134	2433	3614	2213

TSPAN31	-1.328638512	9.84297E-23	down	357	370	331	647	594	645
TSPAN32	-6.740184264	2.39829E-07	down	2	0	0	67	60	24
TSPAN33	-1.744910561	0.001190481	down	85	61	70	312	93	125
TSPAN5	-1.850826215	2.07892E-11	down	52	71	52	155	115	180
TSPAN6	-9.739485903	1.42441E-16	down	0	0	2	346	581	257
TSPAN7	-8.94310158	7.60294E-58	down	6	5	1	1963	898	1399
TSPAN9	-1.578561192	3.8334E-07	down	555	578	569	1092	1516	938
TSPOAP1	-9.088664257	9.74115E-13	down	0	2	0	405	78	310
TSPYL1	-0.922612738	4.35945E-19	down	1015	1055	1020	1490	1233	1449
TSPYL4	-1.276419065	4.22545E-13	down	393	435	410	855	552	741
TSPYL5	-11.61918973	4.78887E-21	down	0	0	0	471	326	446
TSR1	2.035391292	1.24643E-36	up	3344	3666	3257	549	609	610
TSSC1	1.462451804	6.084E-05	up	637	700	611	175	212	106
TSTD1	-9.504961512	1.4994E-11	down	0	0	0	67	147	61
TSTD2	-0.782161552	1.31483E-05	down	404	393	344	440	449	502
TSTD3	-1.832125164	1.11675E-06	down	86	99	57	252	122	246
TTBK2	-0.911958761	0.005390863	down	673	803	761	1064	589	1389
TTC12	-2.045201264	1.34426E-14	down	77	64	73	205	169	259
TTC14	-1.429790843	1.74142E-06	down	777	917	693	1530	974	2119
TTC19	-0.742682418	7.25943E-05	down	434	528	430	545	570	526
TTC21A	-1.424662365	8.50181E-05	down	56	37	48	114	68	91
TTC21B	-0.730881208	0.002182915	down	341	323	306	365	298	490
TTC26	1.332193652	5.48106E-09	up	422	471	393	151	86	129
TTC27	0.608986643	0.002612053	up	552	647	598	330	265	241
TTC28	-0.760550625	0.000997695	down	648	717	645	793	598	1049
TTC33	1.044850408	4.63841E-09	up	1337	1408	1203	497	344	529
TTC38	1.176118267	0.002027165	up	1060	1175	1133	239	475	319
TTC39B	-3.002910822	8.19445E-10	down	70	68	39	468	148	417
TTC6	3.934187412	0.024972299	up	9	6	8	0	1	0
TTC7A	1.373107486	5.28976E-08	up	1347	1453	1340	353	276	517
TTC8	-0.70877906	0.00712731	down	298	322	270	461	246	338
TTC9	-0.646426027	0.038982324	down	108	144	108	157	88	159
TTC9B	4.554160237	0.035956524	up	8	3	7	0	0	0
TTC9C	2.038116815	1.51483E-23	up	1033	1087	930	176	134	221
TTF1	1.059918414	1.83653E-14	up	975	1057	904	374	278	352
TTF2	2.217953247	1.50981E-18	up	1536	1774	1574	217	191	342
TTI1	1.831547012	2.83594E-09	up	979	1103	1012	157	258	191
TTI2	0.689213547	0.032914317	up	292	306	271	181	108	96
TTK	5.526245829	3.3089E-73	up	1388	1504	1385	32	21	13
TTL	1.322751784	1.16154E-09	up	2214	2500	2169	552	712	669
TTLL1	-2.954382203	2.29485E-09	down	16	10	27	120	92	83
TTLL11	-3.271796242	1.07066E-14	down	47	30	34	255	152	372
TTLL12	1.767085653	3.38846E-05	up	1467	1573	1555	222	469	239
TTLL3	-2.663714575	1.52111E-06	down	17	11	26	75	52	122
TTLL4	1.092126096	7.22086E-08	up	508	588	578	194	194	167
TTLL5	1.273350142	4.85137E-11	up	794	942	873	235	270	256
TTLL6	2.748197804	0.000313662	up	50	56	60	5	1	12
TTLL7	-4.977328139	7.02794E-16	down	63	82	84	2300	408	2615
TTYH2	-4.364752043	1.31647E-34	down	9	24	17	240	233	253
TTYH3	2.521756896	2.0003E-102	up	4021	4326	4156	510	478	559
TUB	-8.284881843	3.01821E-31	down	1	0	4	404	288	427
TUBA1A	-1.801188714	5.77024E-21	down	948	895	911	2871	1804	2215
TUBA1B	2.135536438	2.89014E-38	up	29180	32962	28424	5357	3686	5718
TUBA1C	3.180480717	1.41367E-47	up	12246	13700	12300	1311	880	815
TUBA4A	3.393514865	4.21518E-68	up	6269	6866	6579	502	315	531
TUBA8	-2.803390716	0.003877315	down	10	22	8	20	131	32
TUBB3	3.853170229	1.73765E-06	up	693	827	863	30	75	6
TUBB4A	3.184654691	1.86999E-08	up	54	62	64	4	6	4
TUBB4B	2.626602359	9.0359E-19	up	11215	12501	10963	1375	1608	940
TUBD1	0.650296438	0.016852969	up	291	271	266	167	94	118
TUBE1	-1.65127867	3.34618E-08	down	214	174	151	499	267	457
TUBG1	2.263136272	4.21011E-07	up	1460	1500	1494	143	332	163
TUBG2	-0.659759763	0.00240457	down	395	436	438	477	365	594
TUBGCP3	1.517285809	1.99732E-16	up	1158	1132	1018	274	220	332
TUBGCP4	1.758896934	5.23987E-29	up	1510	1581	1483	346	250	371
TUBGCP6	-1.594562232	1.79641E-14	down	306	330	276	691	485	797
TUFM	0.864546332	0.003270353	up	3726	4325	3848	1574	1846	1148
TUFT1	3.083020233	1.9313E-30	up	858	921	817	73	47	100
TUSC1	-1.198517564	4.50003E-05	down	166	176	166	345	173	324
TUSC3	1.617351002	0.02136398	up	1540	1806	1512	144	708	207
TUSC5	-3.364319804	7.59827E-05	down	28	26	16	45	334	101
TVP23A	4.561410507	4.94535E-79	up	918	1084	974	40	21	29
TWF1	1.455317498	1.59576E-26	up	3734	4212	3606	1118	942	927
TWIST1	-2.436627176	1.23551E-06	down	74	71	68	480	126	238
TWIST2	-0.880045062	0.017032392	down	124	146	142	119	188	227
TWISTNB	0.596071987	0.018034969	up	892	887	803	541	290	398
TWSG1	-2.105549245	9.75206E-06	down	652	711	621	2392	770	3084
TXLNA	0.652915278	1.14686E-10	up	1876	1985	1825	901	760	913
TXLNG	1.854854348	4.74393E-05	up	2815	3063	2576	359	840	402
TXN	1.661348538	5.296E-14	up	7192	7774	6789	2184	1311	1427
TXN2	-0.751712578	0.004585694	down	851	898	776	1078	1129	780
TXNDC11	0.978721744	2.23184E-13	up	1313	1487	1313	561	450	472
TXNDC15	-1.998588876	1.43223E-13	down	432	498	490	1750	853	1497
TXNDC16	-1.347169862	4.57543E-05	down	205	203	199	370	226	520
TXNDC17	1.210153753	1.33954E-08	up	1277	1301	1198	491	347	325
TXNDC9	0.847911921	3.95684E-08	up	1057	1130	974	436	413	394
TXNIP	-4.133853901	1.70468E-15	down	1754	1864	1982	8592	27835	32204
TXNL4B	0.748260083	0.003139805	up	365	434	362	146	185	154
TXNRD1	1.918422817	1.0986E-12	up	13745	15069	13304	1939	2515	3428
TXNRD2	1.189570256	0.002654859	up	890	1085	903	263	410	199
TXNRD3	0.690179445	0.010652491	up	157	238	179	81	81	89
TYMP	-1.927079651	2.58467E-06	down	145	161	150	491	495	228
TYMS	3.2751047	1.09537E-16	up	1804	1802	1740	191	132	69
TYMSOS	3.448048707	3.69787E-05	up	27	31	34	3	2	1
TYRO3	2.329042654	0.001845009	up	2138	2371	2035	98	610	159
TYROBP	-12.47979784	7.42405E-21	down	0	0	0	1255	335	717
TYRP1	-5.513804389	1.67693E-08	down	3	3	5	47	242	47
TYSND1	1.285464585	0.013310022	up	1041	1120	1122	242	505	175
TYW5	-0.594868061	0.030196123	down	171	129	169	173	143	192
U2AF1L4	-1.883254484	1.36144E-12	down	72	72	74	245	161	169
U2AF2	1.026740822	3.66446E-10	up	3312	3631	3209	1064	1163	1296

U2SURP	1.237338652	1.57345E-13	up	5511	5955	5218	1807	1277	1984
UACA	-0.887497815	3.50452E-06	down	2134	2267	2182	3407	2096	3252
UAP1	1.549077039	3.78928E-09	up	3725	4101	3602	766	739	1282
UAP1L1	1.727901819	3.402E-14	up	1235	1305	1276	239	309	262
UBA1	1.584142253	1.81558E-12	up	10778	11847	10622	2109	2789	2904
UBA3	-0.65515818	1.53305E-06	down	915	1129	871	1151	977	1127
UBA52	-0.784223587	0.001544472	down	5681	5941	6123	9941	5528	6504
UBA6	0.607920638	2.20442E-07	up	1486	1511	1477	754	602	738
UBA7	-8.092584833	4.70811E-52	down	2	7	3	962	508	881
UBAC1	0.587183137	0.002318712	up	1058	1093	1123	532	540	469
UBALD1	0.672957483	0.000277752	up	343	361	362	176	127	175
UBAP1L	-3.148665788	3.37358E-12	down	14	16	13	86	59	129
UBAP2	1.01185185	0.000263614	up	780	846	804	211	302	336
UBAP2L	1.417982035	5.51303E-15	up	4393	5009	4421	1064	1176	1420
UBASH3B	4.773264497	1.3169E-16	up	2511	2560	2312	39	114	30
UBB	0.608771741	0.000930585	up	15154	16149	14873	7820	7358	6218
UBC	-0.858588977	1.95724E-08	down	9321	9929	9331	13973	9468	13710
UBE2B	-0.62891202	0.000372661	down	1173	1306	1147	1649	1082	1276
UBE2C	5.616361475	0.000430341	up	2063	2116	1954	66	24	0
UBE2D1	0.603154388	0.028008915	up	736	767	713	304	409	309
UBE2D2	-0.648048723	4.18479E-05	down	1211	1321	1091	1589	1184	1267
UBE2D4	-0.935601793	1.09616E-06	down	347	350	315	566	383	434
UBE2E3	-1.112423931	2.93692E-14	down	494	633	495	916	733	840
UBE2F	-0.961447346	1.74631E-05	down	158	158	142	244	204	185
UBE2G1	1.143171177	1.15759E-09	up	1966	2157	1901	558	631	740
UBE2G2	-0.673993778	0.000576812	down	1281	1432	1338	1340	1399	1849
UBE2I	0.778676486	1.85312E-06	up	3044	3145	3003	1486	974	1383
UBE2J1	-0.885693025	1.72117E-15	down	850	948	811	1175	1025	1225
UBE2L6	-12.51356334	3.11651E-23	down	0	0	0	1095	569	657
UBE2O	1.383025329	1.84566E-12	up	1120	1138	1083	339	310	258
UBE2Q2	-1.788257778	1.28883E-18	down	400	499	435	1110	831	1353
UBE2QL1	-10.17163646	7.66328E-15	down	0	0	0	224	92	145
UBE2S	3.257726921	2.10589E-24	up	2362	2761	2426	276	161	126
UBE2T	4.962514215	2.42485E-57	up	1372	1453	1421	47	30	20
UBE2V1	1.40225785	0.000239847	up	71	88	74	22	14	27
UBE3C	1.207781969	1.33565E-08	up	3596	3910	3645	941	1175	1290
UBE4B	0.886083566	7.43997E-05	up	2348	2702	2568	900	770	1276
UBFD1	1.688427038	2.17183E-16	up	2477	2799	2407	476	595	607
UBL3	-1.220726868	3.54941E-14	down	514	588	544	921	743	1074
UBL4A	0.901373605	1.4809E-08	up	1451	1529	1265	616	497	501
UBL5	-0.639276903	0.006708419	down	1170	1202	1069	1672	1088	1067
UBN1	1.311115359	6.10881E-06	up	1923	2401	2156	451	705	671
UBOX5	-1.334167645	0.000622343	down	58	113	77	205	105	136
UBQLN1	1.283512034	1.37117E-39	up	6516	7137	6123	2114	1687	1980
UBQLN2	0.607569743	0.018480097	up	1634	1769	1472	936	496	873
UBQLN4	1.346676649	4.87767E-10	up	1744	2095	1730	679	428	455
UBR3	-1.361006974	3.70984E-08	down	594	667	530	859	1127	1251
UBR4	0.998505573	5.42262E-05	up	6773	7577	7170	2077	2209	3378
UBXN1	-0.66665758	0.017342852	down	1190	1251	1099	1877	927	1244
UBXN10	-5.219626255	0.001234467	down	1	0	0	8	12	8
UBXN6	-0.863555478	2.14105E-06	down	878	970	922	1227	1249	1081
UBXN7	0.893492091	0.003061462	up	2139	2256	2168	599	782	1124
UCHL1	-11.33381887	2.55645E-19	down	0	0	0	310	392	297
UCHL3	1.151186376	7.39884E-06	up	839	912	850	317	299	210
UCHL5	2.111104561	7.47369E-33	up	2554	2931	2487	387	443	472
UCK2	2.809844486	9.34372E-39	up	2581	2680	2448	229	293	251
UCN2	5.520733595	7.42455E-08	up	68	56	67	3	0	0
UCP2	1.811961522	2.46427E-07	up	1845	1984	1859	342	511	272
UFC1	-0.950589799	3.09471E-05	down	989	1092	949	1829	1005	1373
UFL1	-1.322622422	6.75458E-10	down	511	528	457	762	803	1092
UFM1	-0.796449642	2.68872E-10	down	1146	1236	1144	1442	1276	1641
UFSP1	1.268194842	0.013657645	up	51	64	45	10	14	23
UFSP2	-2.929375987	1.38127E-16	down	124	128	156	981	407	875
UGCG	-0.799178098	0.020986781	down	535	514	470	651	768	430
UGDH	2.060504394	1.07994E-22	up	3163	3332	2954	521	406	695
UGGT1	2.126547053	2.15422E-24	up	5938	6232	5635	793	973	1114
UGP2	-0.64196903	0.000959861	down	3356	3600	3262	3604	4084	3533
UGT8	-5.639606441	0.003731768	down	0	0	0	7	3	10
UHMK1	1.028209066	0.008773184	up	4389	4714	4235	987	2084	1455
UHRF1	6.089464049	1.88307E-24	up	2008	2141	2040	41	19	5
UHRF1BP1	0.823321777	0.034428206	up	1228	1344	1199	333	675	468
ULBP1	5.198291748	2.63336E-09	up	77	66	64	0	2	2
ULBP2	4.284297465	2.48038E-18	up	175	249	189	8	11	3
ULBP3	2.176158169	0.000570971	up	151	152	126	18	38	9
ULK1	1.131094765	2.6955E-10	up	2019	2420	2244	752	559	871
ULK2	-2.294850443	1.21249E-45	down	133	152	137	501	425	548
ULK4	1.977125981	4.52464E-09	up	237	223	161	34	43	34
UMPS	1.369667569	1.0925E-12	up	1129	1330	1089	373	320	278
UNC119	0.651153641	0.000971278	up	546	533	466	237	239	220
UNC119B	-0.873195633	4.63517E-07	down	560	584	529	662	681	831
UNC13A	1.803784092	0.000268802	up	147	166	170	26	17	57
UNC13B	-1.015146887	7.75548E-05	down	654	773	644	764	1024	1157
UNC13C	-8.385573641	6.11444E-08	down	0	0	0	65	12	59
UNC13D	3.308192058	7.73367E-38	up	1889	2150	2038	200	95	147
UNC50	-0.661822129	0.004864706	down	585	711	577	861	487	779
UNC5A	3.305694296	0.032059527	up	44	28	18	0	6	0
UNC5B	-1.337672234	0.013746582	down	230	245	181	361	592	184
UNC5C	-11.65569476	1.28785E-19	down	0	0	0	280	493	473
UNC5CL	-2.014917782	0.002497077	down	7	8	14	28	16	41
UNG	1.804121112	0.00076911	up	1660	1924	1738	206	595	235
UPB1	-3.626053801	0.001005789	down	2	4	0	19	22	10
UPF3B	0.898427019	1.26403E-06	up	910	1008	890	366	276	435
UPK1A	3.060080881	0.03448095	up	14	17	15	0	0	4
UPK3B	4.660654852	5.3017E-09	up	53	71	54	2	1	2
UPP1	0.92969946	7.87283E-07	up	757	806	700	314	281	246
UPRT	-0.633609503	0.029762571	down	180	254	179	289	200	185
UQCR10	-1.101938166	0.011920437	down	738	956	813	1038	1838	822
UQCR11	-1.490319981	1.43827E-08	down	1002	1004	949	2043	2263	1524
UQCRB	-1.106633594	3.38592E-06	down	3973	4422	3717	7568	6118	4767
UQCRC1	0.94358006	0.014863319	up	5333	5659	5465	1633	2807	1480
URB1	1.096686709	0.002364389	up	1844	2026	1852	533	404	990

URB2	2.249156942	4.58325E-07	up	830	870	772	98	184	76
URM1	0.701124663	2.93956E-06	up	1320	1327	1254	580	567	553
USB1	1.710965398	1.2395E-19	up	1445	1501	1385	289	336	308
USF1	-0.854190594	0.000117367	down	534	550	575	923	549	682
USF3	-0.952226661	3.20129E-08	down	327	330	318	419	418	503
USH1C	6.947026631	1.77251E-56	up	988	1065	1037	12	1	5
USH1G	5.049206903	0.008247705	up	9	12	5	0	0	0
USH2A	7.349098943	1.77288E-24	up	1009	1057	1049	1	11	1
USHBP1	-6.457646673	6.50069E-17	down	3	4	0	89	147	195
USP1	1.441642484	7.943E-16	up	2777	3141	2642	884	553	821
USP10	1.180759955	1.1704E-07	up	2027	2169	1976	546	562	829
USP24	0.714397417	0.005978574	up	2889	3164	2820	979	1368	1456
USP27X	-1.873767606	6.46705E-05	down	36	26	56	106	71	137
USP30	-0.951441463	0.000181653	down	208	224	202	262	329	270
USP31	-0.667957752	0.013548281	down	440	455	397	548	319	612
USP32	1.055900611	8.42561E-06	up	1808	1940	1830	504	617	778
USP35	-0.632531999	0.041159099	down	276	353	286	336	222	460
USP36	0.625791754	0.001939958	up	1145	1196	1093	490	436	662
USP37	0.803916236	5.56681E-07	up	548	633	510	229	218	239
USP39	0.673051679	3.5674E-07	up	1306	1424	1327	689	497	631
USP4	-1.437625644	9.85054E-32	down	475	542	461	1033	809	1009
USP40	-0.928736115	3.11929E-07	down	452	508	398	737	521	583
USP42	0.684861438	0.004643958	up	565	550	551	324	204	214
USP43	5.569749245	1.52475E-14	up	108	135	156	1	1	4
USP44	-5.637968062	2.49262E-07	down	0	1	3	37	23	85
USP45	-0.975036808	0.000924294	down	162	168	189	188	239	296
USP46	-1.172909602	0.00103332	down	266	265	236	299	527	377
USP47	0.931202186	2.6181E-19	up	4229	4650	4293	1698	1412	1811
USP49	0.824796632	0.014616517	up	404	386	364	105	160	195
USP50	-4.837978251	0.033977275	down	0	0	0	4	5	2
USP51	-2.154788281	8.45165E-08	down	32	29	17	89	86	70
USP53	-2.127460534	8.58539E-08	down	872	989	894	2368	1633	4697
USP54	0.98875909	0.004627828	up	1082	1143	1024	347	244	589
USP7	0.78935213	6.66845E-06	up	4081	4085	3839	1662	1306	2004
USP9X	1.451639322	6.78776E-13	up	8295	9004	8479	1891	2055	2743
USP9Y	-12.12209457	0.004017139	down	0	0	0	803	0	1038
USPL1	-0.620953567	0.002063187	down	389	459	358	533	341	451
UST	-3.448232141	5.84905E-10	down	69	92	52	736	179	781
UTP11	1.308785922	2.54277E-18	up	1540	1753	1500	481	457	430
UTP14A	1.226187111	4.41754E-10	up	1019	1080	928	384	267	271
UTP14C	-1.371876064	3.95777E-12	down	295	355	266	545	467	674
UTP15	0.695102475	0.006369455	up	543	619	474	278	245	190
UTP18	2.570067629	4.31065E-19	up	2159	2570	2132	306	313	191
UTP20	1.839989647	2.6408E-18	up	1299	1527	1326	233	294	289
UTP4	1.725418141	1.4806E-08	up	633	662	640	100	165	145
UTP6	0.751394618	1.63874E-05	up	1311	1425	1180	666	482	508
UTRN	-2.460341031	1.41374E-20	down	787	842	828	2374	3417	3729
UTS2	-8.291597327	1.27965E-08	down	0	0	0	44	18	64
UTY	-10.77889623	0.011142786	down	0	0	0	339	0	387
UXS1	1.09028385	2.8385E-15	up	1142	1277	1140	456	354	379
VAC14	0.671862277	0.018999915	up	693	830	800	275	402	344
VAMP1	-1.286125236	0.000362043	down	187	200	184	438	177	396
VAMP2	-2.386128885	4.68615E-18	down	398	430	363	2059	976	1455
VAMP3	-1.438481886	4.27552E-16	down	1297	1397	1272	2878	1889	2946
VAMP4	-2.188243317	3.00576E-09	down	270	316	264	1207	459	1148
VAMP5	-6.57064	1.66569E-49	down	10	13	13	1255	609	595
VANGL1	1.412760522	2.12659E-18	up	1269	1310	1273	327	349	348
VANGL2	-7.618217581	1.39769E-08	down	1	0	1	142	111	25
VASH1	-4.903934231	3.45048E-89	down	40	47	49	856	987	1032
VASH2	2.997068405	0.000811159	up	428	372	355	4	22	78
VASN	2.151826573	0.00707227	up	31	23	15	3	4	4
VASP	1.435833515	0.000211083	up	4187	4406	4167	1832	638	958
VAT1	0.998474245	5.98603E-07	up	6105	6655	6127	1888	2150	2655
VAT1L	-12.05810258	2.19859E-21	down	0	0	0	813	377	509
VAV2	2.186808805	7.09747E-81	up	2373	2577	2435	404	362	386
VBP1	0.777099215	0.00055196	up	1609	1952	1521	626	771	682
VCAM1	-12.87360873	1.64097E-22	down	0	0	0	967	1361	540
VCAN	-15.64556091	5.06307E-37	down	0	1	0	17060	10083	12404
VCPKMT	-0.856959307	0.003158514	down	104	125	131	186	106	177
VDAC1	2.439570242	8.56649E-11	up	13429	14595	13351	1371	2531	1364
VDAC2	1.124947219	4.81452E-09	up	4906	5155	4990	1704	1740	1425
VDR	3.108282478	1.16934E-42	up	1194	1261	1065	115	98	76
VEGFB	-2.062915688	3.85739E-28	down	566	536	515	1741	1630	1413
VEGFC	-1.604266427	6.48518E-06	down	103	105	74	213	239	148
VENTX	-8.815956702	1.24058E-09	down	0	0	0	105	26	51
VEPH1	2.727005992	0.041693851	up	245	225	217	0	57	11
VGF	5.206235032	4.306E-05	up	34	48	22	1	0	1
VGLL3	-1.670667919	0.000326592	down	625	620	526	825	885	2342
VGLL4	-2.070544342	4.10243E-11	down	651	680	590	2623	1097	2130
VHL	1.372940055	1.13123E-10	up	1769	2028	1835	430	545	556
VILL	-6.01053749	3.28769E-18	down	0	3	6	175	133	104
VIM	-2.900498104	5.54769E-20	down	14098	15116	14223	103993	42232	89379
VIP	-6.920066746	2.40223E-09	down	0	0	2	61	43	70
VIPAS39	-0.720474636	0.00434205	down	269	414	269	367	309	435
VIPR1	-4.047805805	2.41545E-09	down	35	13	30	124	264	542
VIPR2	-1.680115685	0.004388921	down	114	92	118	356	77	335
VIT	-3.97748562	2.71032E-13	down	60	79	55	343	513	1322
VMA21	0.776400778	1.2043E-06	up	1477	1557	1275	701	514	577
VMAC	-2.510505442	2.17433E-09	down	15	17	22	60	66	93
VMO1	-5.105415281	4.26903E-06	down	1	0	5	94	27	32
VMP1	1.001343301	1.63829E-05	up	3427	3690	3355	1681	964	1106
VN1R1	-6.380707563	0.0002874	down	0	0	0	5	14	13
VNN1	-5.943215155	1.11475E-11	down	2	4	2	54	71	227
VNN2	-10.21193206	2.53771E-15	down	0	0	0	133	116	220
VNN3	-5.589722969	0.00256657	down	0	0	0	6	5	8
VPRBP	0.938588541	1.85411E-10	up	1291	1457	1240	470	481	521
VPS11	2.875918381	0.019652523	up	50	13	8	4	2	1
VPS13B	-0.899992287	0.000499177	down	713	816	696	881	749	1338
VPS13C	-1.284144724	1.80018E-06	down	988	1186	1047	1464	1598	2512
VPS13D	-0.890727903	0.004585694	down	1233	1394	1273	1189	1629	2297
VPS16	-1.075344243	8.66987E-05	down	145	192	168	243	285	218

VPS26A	0.810125013	3.25708E-13	up	3160	3350	2853	1377	1141	1278
VPS28	-1.887160461	4.22796E-14	down	406	460	439	1564	820	1089
VPS33A	0.836087202	1.28414E-06	up	602	701	568	235	243	263
VPS33B	0.779368513	0.000226583	up	455	478	435	238	144	189
VPS35	0.813839977	2.45572E-08	up	4125	4359	3936	1564	1632	1806
VPS36	-1.640178178	1.79475E-17	down	414	522	411	923	855	1203
VPS37B	0.849685237	1.53688E-06	up	915	1035	962	424	290	443
VPS39	-1.120366225	3.60199E-14	down	774	919	813	1267	1111	1498
VPS4B	-1.187967248	7.96071E-07	down	603	682	603	988	743	1352
VPS51	-1.042252757	9.52108E-07	down	896	950	830	1683	966	1308
VPS53	0.693011839	0.001576389	up	877	968	928	340	391	484
VPS72	0.800536897	2.57665E-05	up	1151	1257	1086	576	353	509
VPS8	-1.315439218	1.26081E-14	down	333	327	320	571	584	572
VPS9D1	-0.599029167	0.008565923	down	130	179	150	191	143	160
VRK1	2.117597582	4.00461E-17	up	717	901	661	158	115	99
VRK3	-0.676967028	0.000260026	down	234	276	249	294	289	275
VSIG10	1.043883098	4.10402E-09	up	917	918	883	288	318	327
VSIG2	-2.291635091	0.001997515	down	12	9	9	26	56	19
VSIG4	-13.89176828	1.12202E-23	down	0	0	0	1433	777	3915
VSNL1	-6.784129847	0.00062924	down	1	1	0	9	131	1
VSTM2L	-7.199318293	4.31813E-06	down	0	0	0	13	28	15
VSTM4	-13.63548164	5.17453E-28	down	0	0	0	1400	1859	1674
VTCN1	6.566963429	2.81561E-05	up	17	41	17	0	0	0
VTI1A	0.712847793	0.000241569	up	958	1072	874	373	425	452
VTI1B	-1.261255596	1.86172E-06	down	867	807	747	1281	1614	1175
VWA1	-5.97698864	4.52227E-43	down	11	19	12	810	648	402
VWA2	-6.400332717	0.024938573	down	0	0	0	0	16	16
VWA3B	-4.145079787	0.045029492	down	0	1	0	7	1	6
VWA5A	2.203431291	8.10159E-12	up	4249	4713	4308	890	369	832
VWA8	-1.007063205	0.00179578	down	347	345	329	384	607	441
VWC2	-7.791771145	0.000151897	down	0	0	0	17	62	2
VWDE	5.87149435	1.00687E-12	up	102	118	108	2	0	2
VWF	-10.59561141	2.30854E-72	down	14	9	4	5185	13579	10107
WAPL	0.760249589	4.84494E-08	up	2046	2105	1886	858	713	971
WARS	0.831067355	0.003223146	up	2392	2562	2293	796	1165	884
WAS	-4.753907608	4.58291E-23	down	6	4	8	144	90	115
WASF1	1.049748261	0.00102988	up	400	415	376	98	161	144
WASF3	-5.099321919	3.17299E-37	down	21	29	25	510	406	929
WASH1	-2.937985046	0.000386383	down	17	27	28	298	65	40
WBP1	-2.753571183	1.82862E-10	down	18	18	24	85	69	137
WBP11	1.154889155	6.44929E-09	up	2903	3073	2634	804	811	1133
WBP1L	-1.996693266	6.25268E-22	down	454	562	456	1219	1474	1434
WBP4	-1.673091294	3.4286E-13	down	243	308	275	595	485	801
WBSCR17	-10.36302209	4.3446E-16	down	0	0	0	146	198	166
WBSCR22	1.707222395	3.03966E-24	up	2613	2790	2445	654	550	500
WDCP	0.787860856	0.000692519	up	511	488	410	196	149	239
WDFY1	-1.536344184	1.99444E-16	down	516	654	523	1355	877	1279
WDFY2	-2.040056635	2.23787E-07	down	187	254	233	509	430	1051
WDFY3	-2.078530159	9.77228E-11	down	585	630	534	1444	1230	2625
WDFY4	-9.165258466	3.18874E-11	down	0	0	0	55	49	124
WDHD1	3.302595775	2.98194E-55	up	813	826	756	53	48	72
WDPCP	-1.310854358	0.000934234	down	54	88	51	84	123	127
WDR1	-0.747190094	0.0125183	down	4185	4382	3898	5879	2951	6310
WDR11	-1.038293703	4.56007E-07	down	562	633	561	780	711	1079
WDR12	1.04304626	5.07007E-09	up	865	983	786	306	310	286
WDR17	-8.991841179	1.27609E-11	down	0	0	0	49	64	86
WDR19	-1.470223194	0.00028262	down	373	421	365	583	479	1249
WDR24	0.699438638	0.000185422	up	322	309	306	153	116	143
WDR25	0.85148329	0.003982518	up	252	239	266	101	114	81
WDR27	-0.967327012	4.87209E-08	down	188	238	202	291	279	299
WDR3	2.307387666	6.7292E-15	up	2775	3067	2697	296	493	415
WDR31	-2.688045892	7.37616E-05	down	17	21	13	39	127	57
WDR33	0.877836051	5.85242E-16	up	2283	2447	2116	936	768	948
WDR34	2.953989876	1.49519E-39	up	2618	2864	2475	302	236	190
WDR36	1.097550224	5.60933E-07	up	1187	1314	1222	363	345	532
WDR37	-1.416117377	2.81489E-07	down	144	221	201	330	290	455
WDR4	2.249081475	4.54277E-31	up	421	468	453	67	58	76
WDR43	1.814667242	1.95875E-15	up	2102	2315	2022	360	483	442
WDR44	1.852060918	3.61421E-43	up	2062	2466	2056	467	356	476
WDR45B	0.67403045	1.44527E-09	up	2211	2249	2245	1074	900	1019
WDR48	-0.76832956	0.000155175	down	731	731	721	873	688	1103
WDR5	1.747453903	5.86991E-30	up	2106	2292	1914	486	435	410
WDR54	1.60215823	3.73312E-10	up	410	391	409	122	65	100
WDR55	0.846232364	1.20348E-09	up	898	1026	995	396	367	389
WDR62	4.538464874	8.15E-48	up	560	658	607	28	15	13
WDR66	1.034834832	0.013295021	up	110	115	106	32	25	59
WDR7	-1.593781374	1.21596E-16	down	180	256	214	451	433	504
WDR70	1.127413358	9.49564E-14	up	971	1110	1006	320	300	384
WDR72	5.857654098	4.78765E-19	up	210	196	170	1	5	1
WDR74	0.855132497	0.002702052	up	720	802	671	408	205	258
WDR76	2.585908805	2.15142E-29	up	808	801	774	81	80	122
WDR77	1.619778894	1.66179E-09	up	1754	1886	1670	374	493	341
WDR78	-1.175115948	0.001284772	down	26	30	32	55	38	49
WDR82	-0.849845015	4.25985E-12	down	1662	1831	1487	2246	1825	2317
WDR83	-0.787781089	0.00556246	down	97	124	86	132	94	152
WDR83OS	-1.02664705	2.4614E-14	down	645	726	717	1032	967	1018
WDR86	-6.808188942	3.60218E-06	down	1	0	2	92	5	157
WDR90	2.879822549	1.74576E-32	up	1232	1466	1507	159	90	162
WDR91	-1.038853748	0.000883751	down	248	330	275	448	258	555
WDSUB1	-1.772109089	5.26957E-09	down	73	87	60	217	120	202
WDTC1	-0.981810258	9.3092E-05	down	476	534	478	546	680	851
WEE1	1.122684942	0.000955728	up	1783	1933	1638	885	355	539
WFDC1	2.77290298	1.81098E-05	up	2241	2591	2294	544	103	118
WFDC2	-5.555523917	0.000319519	down	0	1	0	16	11	9
WFDC3	1.787479429	0.003271989	up	28	42	42	5	7	11
WFIKKN1	4.701678886	1.56929E-11	up	86	91	111	5	0	3
WFIKKN2	-4.294944167	0.008534613	down	3	0	0	29	5	10
WFS1	-1.283005905	4.46476E-07	down	678	676	622	1271	752	1445
WHRN	4.692877009	1.46014E-49	up	1139	1140	1279	21	39	37
WHSC1	2.070986063	3.2401E-35	up	3368	3567	3463	546	610	592
WIPF1	-0.928797703	0.013265913	down	1166	1298	1247	1945	857	2333

WIPF3	-4.370512357	5.07506E-08	down	6	1	7	106	67	36
WISP1	-10.33399052	1.1317E-15	down	0	0	0	245	127	140
WNK2	-2.455400592	0.010085441	down	14	40	21	41	198	30
WNK3	1.040640477	0.002292854	up	138	201	191	50	52	81
WNK4	4.292091869	8.20171E-06	up	36	37	37	0	1	3
WNT1	4.750582279	0.002551399	up	14	17	10	0	0	1
WNT10B	7.351266286	2.787E-105	up	3028	3631	3197	9	10	24
WNT11	-1.840536364	1.8741E-06	down	31	35	36	67	75	118
WNT2B	-2.10263901	5.58166E-06	down	41	25	39	106	66	156
WNT5B	-7.13324661	1.92444E-08	down	0	1	1	121	41	41
WNT7A	7.45112815	4.64401E-08	up	53	46	37	0	0	0
WNT7B	8.479780014	7.55786E-22	up	468	562	476	2	0	1
WNT9A	1.877209618	7.24147E-09	up	130	175	138	23	30	32
WRAP53	1.980900901	4.93897E-23	up	416	456	431	90	59	87
WRN	0.692972629	0.006403945	up	632	758	551	241	309	292
WSB1	-1.716758397	8.64476E-09	down	1551	1877	1798	4141	2546	5703
WSCD1	-6.473088492	1.10636E-14	down	0	4	1	74	103	134
WSCD2	-7.11223744	8.44268E-05	down	0	0	1	9	9	91
WTAP	-0.636170645	0.004962663	down	1250	1257	1178	1769	991	1360
WTIP	-2.951670825	2.3853E-13	down	128	120	128	785	336	1001
WWC1	8.002649162	1.77301E-16	up	2426	2618	2338	2	17	0
WWC3	-0.839836585	0.002390137	down	857	842	858	985	796	1507
WWOX	-0.728034946	0.047591676	down	181	144	140	234	108	217
WWP2	-1.781391213	0.000425415	down	621	734	636	2184	561	2271
WWTR1	-1.275995849	1.74511E-12	down	1850	1987	1799	3973	2423	3399
XAF1	-13.1092888	4.96123E-26	down	0	0	0	1534	949	1008
XAGE2	7.433743969	9.58169E-09	up	90	80	91	0	1	0
XCL2	-5.09008599	0.001671189	down	0	1	0	7	8	11
XDH	9.91214117	1.1317E-15	up	513	478	466	0	1	0
XG	-7.612640669	5.46315E-10	down	2	4	0	215	28	612
XK	3.150090641	5.85437E-15	up	1140	1287	1129	55	78	152
XKR6	-8.160936604	1.55034E-12	down	0	2	0	177	80	155
XKR8	-6.029327119	5.23312E-19	down	1	2	4	132	76	121
XPA	-0.901636633	0.00121275	down	332	364	302	545	281	518
XPC	-1.472391463	3.63114E-08	down	389	469	405	849	558	1111
XPNPEP1	1.079600177	2.54467E-09	up	2155	2283	2105	782	761	644
XPNPEP2	-9.080304957	5.68673E-09	down	1	0	0	26	118	269
XPNPEP3	-0.721626672	0.020301146	down	192	175	140	172	227	188
XPO1	1.724795253	1.12417E-15	up	11019	11921	11341	3109	2280	1988
XPO5	2.58458173	1.8121E-114	up	2600	2737	2542	324	276	335
XPO6	1.705843529	4.52782E-31	up	3441	3677	3400	864	731	693
XPO7	0.78934171	2.81831E-06	up	2937	3285	2991	1178	1092	1524
XPOT	1.72821675	2.40248E-78	up	6100	6462	5766	1378	1165	1393
XPR1	1.018359701	0.00440161	up	2797	2915	2659	613	1168	1105
XRCC2	5.789706706	6.25208E-72	up	818	913	850	10	15	8
XRCC3	1.920494003	1.41485E-12	up	341	341	311	49	69	67
XRCC4	0.766227756	0.012973432	up	545	511	507	263	233	154
XRCC6	1.020294788	1.3615E-14	up	8304	8965	7463	2806	2773	3052
XRRA1	-0.735074456	0.005501157	down	219	174	144	216	197	223
XXYLT1	0.906220859	5.29406E-05	up	403	422	358	146	162	137
XYLB	1.892952701	1.10597E-08	up	216	245	243	43	56	34
YARS	1.808635507	3.36332E-07	up	3030	3387	3102	686	808	401
YARS2	0.862427081	0.045990292	up	553	571	575	173	317	156
YBEY	0.667588118	0.00404716	up	324	377	301	129	152	164
YBX2	3.66988501	6.52771E-05	up	283	359	322	3	40	7
YBX3	-0.736293712	3.33565E-12	down	2942	3130	2917	3567	3354	3704
YDJC	2.737675686	1.74755E-28	up	582	618	555	80	59	48
YEATS2	0.814205602	7.78577E-09	up	1361	1544	1405	641	465	645
YEATS4	1.57253257	3.69653E-21	up	781	889	759	218	185	176
YES1	0.957471846	0.001688174	up	2248	2262	2108	583	952	850
YIF1A	0.716296943	0.002850929	up	1282	1222	1145	683	468	434
YIF1B	0.933212001	1.32413E-05	up	813	909	841	332	343	271
YIPF1	-1.003593051	1.27704E-05	down	250	217	253	338	359	326
YIPF3	-0.802672098	4.07366E-07	down	681	687	643	779	783	928
YKT6	1.044083888	1.64099E-12	up	3150	3350	3152	1037	1097	1179
YME1L1	0.744907993	1.07516E-12	up	4728	5168	4701	2055	1899	2231
YOD1	3.264231732	3.53412E-33	up	2783	3188	2876	161	198	294
YPEL1	-4.441116076	5.47192E-25	down	10	16	14	190	140	293
YPEL2	-3.616769914	1.04459E-23	down	173	162	179	1333	916	2316
YPEL3	-4.30850196	2.73825E-59	down	84	64	88	1308	810	1260
YPEL4	-1.974519357	0.013849369	down	14	17	41	126	24	60
YPEL5	-2.105709417	4.88246E-18	down	695	791	725	2618	1475	2766
YRDC	1.717498889	3.08075E-11	up	539	545	459	111	127	92
YTHDF1	1.034016319	1.90454E-07	up	1800	1711	1624	745	518	528
YWHAE	1.032797689	0.000388141	up	210	243	209	66	90	71
YWHAH	-1.504637686	0.000202257	down	1188	1301	1154	4031	1301	2190
YWHAQ	0.792703616	2.32482E-05	up	9988	11252	9786	4924	3086	4842
YWHAZ	0.76036468	8.64039E-10	up	19125	20538	17799	9253	6661	8305
YY1	1.737768842	2.65025E-43	up	3596	3742	3530	759	752	799
YY2	1.956973725	1.75151E-08	up	218	244	253	51	24	58
ZADH2	-1.355369313	5.49162E-11	down	283	314	273	463	549	557
ZAK	-1.417665746	1.67469E-09	down	1971	2196	1944	3039	4026	4430
ZBED1	4.310077335	0.014278344	up	5	10	15	0	0	1
ZBED2	4.891025235	0.013330584	up	11	7	5	0	0	0
ZBED4	1.374935389	2.88989E-17	up	640	734	662	197	184	175
ZBED6CL	1.273410506	4.99684E-05	up	161	153	156	60	30	50
ZBED8	-0.859211156	0.001793743	down	170	175	166	279	147	242
ZBED9	2.895558547	0.017089188	up	11	17	14	0	1	3
ZBP1	1.775668506	7.76763E-05	up	82	84	68	25	11	13
ZBTB10	-1.558851239	2.1509E-07	down	415	433	415	1108	519	1069
ZBTB11	0.947672783	0.000135086	up	1321	1423	1298	569	326	615
ZBTB14	-1.011376576	1.28708E-07	down	270	255	269	438	298	412
ZBTB16	-14.71669423	1.62857E-25	down	0	0	0	1608	1782	7377
ZBTB18	-0.684430714	0.03843414	down	281	300	261	222	346	383
ZBTB20	-1.988339604	2.12541E-23	down	647	679	702	1849	1511	2402
ZBTB25	0.883858756	3.23459E-07	up	449	567	492	219	160	203
ZBTB26	0.841048673	0.020223194	up	360	386	362	104	120	217
ZBTB33	1.57603236	6.0683E-29	up	1454	1802	1478	374	354	396
ZBTB4	-0.721975146	0.008202268	down	2226	2403	2209	3440	1682	3029
ZBTB43	1.957864344	6.80317E-28	up	1448	1531	1506	244	277	296
ZBTB44	0.680629897	0.008146961	up	2086	2124	2124	747	847	1216

ZBTB45	-0.994586314	0.000524651	down	103	87	72	140	96	138
ZBTB47	-2.579159732	3.46896E-30	down	434	406	400	1938	1233	2168
ZBTB49	-1.523083781	2.5548E-07	down	38	52	39	93	86	83
ZBTB6	0.673672852	0.020301146	up	640	612	597	254	197	382
ZBTB7B	0.645539838	0.004192921	up	1426	1503	1454	568	726	678
ZBTB7C	-4.211283233	8.33748E-21	down	13	17	13	288	122	164
ZC2HC1A	-2.208396907	2.06752E-16	down	91	130	94	408	240	394
ZC3H10	-1.02830689	0.001143107	down	113	100	89	199	107	137
ZC3H12A	1.112703602	0.003500679	up	326	355	343	178	80	83
ZC3H12B	-5.350969289	7.30925E-09	down	6	2	0	126	38	75
ZC3H13	-0.780237541	0.000176326	down	1243	1483	1247	1561	1260	2051
ZC3H18	1.079644519	1.30045E-12	up	1185	1248	1108	374	385	428
ZC3H3	0.715554648	0.001334609	up	181	195	174	91	64	84
ZC3H6	-1.109552753	8.77241E-07	down	241	285	224	377	297	481
ZC3H7B	-1.209425651	3.76168E-07	down	768	851	780	1039	1303	1577
ZC3H8	1.545635394	2.5858E-07	up	450	524	427	96	140	99
ZC3HAV1L	1.627997202	1.33007E-07	up	481	534	515	105	147	94
ZC3HC1	1.240515506	1.14618E-07	up	616	756	624	221	212	162
ZC4H2	-3.268532835	1.42577E-18	down	19	31	19	186	105	185
ZCCHC11	0.880668738	5.02375E-05	up	1222	1285	1145	393	442	571
ZCCHC14	1.147714409	3.96139E-05	up	2375	2564	2452	820	508	1078
ZCCHC18	-8.590910666	8.98673E-10	down	0	0	0	83	32	39
ZCCHC2	-1.414640994	7.98266E-08	down	221	219	229	523	280	485
ZCCHC24	-2.596481466	4.75563E-85	down	481	497	498	2165	2052	2115
ZCCHC5	-4.215421496	0.001507731	down	5	0	4	14	13	98
ZCCHC8	0.666223808	0.00068242	up	960	974	904	492	311	481
ZCWPW1	-2.400833171	5.87063E-10	down	38	27	20	110	81	131
ZCWPW2	-3.14101977	2.35177E-09	down	10	14	9	100	39	71
ZDHHC1	-4.406538943	1.74065E-20	down	13	10	3	142	108	143
ZDHHC11	3.531175271	7.84046E-12	up	414	555	497	11	32	46
ZDHHC12	1.10637656	0.000659	up	401	539	366	206	108	119
ZDHHC14	-0.910510094	4.96454E-05	down	159	155	145	230	202	180
ZDHHC15	-7.817770834	6.64391E-15	down	2	0	1	235	81	179
ZDHHC16	1.008549924	2.84082E-11	up	741	789	775	264	262	287
ZDHHC17	-1.376773936	9.91706E-11	down	498	654	568	1135	777	1285
ZDHHC18	1.13909516	8.44526E-13	up	698	712	689	219	222	235
ZDHHC21	-2.211394216	3.02531E-28	down	190	220	173	659	498	771
ZDHHC23	1.301370777	4.36262E-09	up	399	474	386	128	89	148
ZDHHC24	0.640691996	0.004279409	up	286	327	321	135	151	137
ZDHHC5	0.744160021	0.000257093	up	2600	2760	2566	962	1177	1197
ZDHHC6	0.767743849	1.21797E-07	up	1475	1577	1386	687	492	684
ZDHHC9	1.86471478	6.75439E-08	up	1648	1783	1665	224	426	323
ZEB1	-2.329805321	1.56908E-10	down	940	970	1005	3605	1851	5194
ZEB2	-4.921850071	2.8076E-139	down	115	130	125	2445	2758	2708
ZFAND2B	-1.210178637	7.02346E-07	down	205	247	206	447	250	396
ZFAND3	-1.021828177	4.65948E-06	down	959	864	914	1161	1151	1655
ZFAND4	-1.232902776	0.000264449	down	71	83	80	135	152	99
ZFAND5	-0.809084764	0.000256225	down	3351	3682	3478	3829	3641	5663
ZFC3H1	0.930082468	0.000960947	up	2700	3093	2928	1066	723	1505
ZFHX2	-5.240001539	1.82947E-08	down	0	3	1	36	37	33
ZFHX4	-5.795472152	2.8884E-11	down	2	5	1	57	65	194
ZFP14	-1.487359328	3.4507E-05	down	163	137	111	389	172	273
ZFP2	-8.147542775	6.92809E-09	down	1	0	0	119	31	73
ZFP28	-10.11703758	2.83963E-15	down	0	0	1	336	168	360
ZFP3	-1.485465916	8.30166E-09	down	92	98	87	155	179	215
ZFP36	-3.496492241	8.33305E-17	down	236	246	277	2639	2356	1035
ZFP36L1	-1.868037723	5.07893E-17	down	1198	1327	1303	3163	3734	2917
ZFP36L2	-1.973822406	4.4297E-41	down	1484	1455	1298	3825	3883	4077
ZFP37	-2.530837504	4.42802E-08	down	36	34	22	198	85	100
ZFP41	-1.089795569	1.78083E-05	down	88	90	71	121	109	147
ZFP64	0.9006892	1.41131E-06	up	382	434	420	169	124	180
ZFP69B	2.5433834	8.65514E-12	up	363	393	346	45	59	28
ZFP82	-0.588990464	0.015251505	down	150	134	149	183	139	143
ZFP90	-1.545635434	1.03794E-23	down	297	314	281	721	528	608
ZFP92	-6.373829065	5.18833E-05	down	0	2	0	10	72	27
ZFPM2	-8.968221972	1.71611E-30	down	2	2	0	585	381	466
ZFR2	5.120035047	0.009902557	up	4	9	14	0	0	0
ZFY	-10.61037388	0.012550869	down	0	0	0	304	0	342
ZFYVE1	-0.932380694	0.000723991	down	316	316	290	337	360	556
ZFYVE19	1.071262772	1.80693E-08	up	774	810	806	240	273	292
ZFYVE21	-1.259751954	0.000773937	down	839	966	895	2267	793	1643
ZFYVE28	-1.760030005	3.86571E-07	down	52	34	42	93	106	109
ZFYVE9	-0.94950586	2.80557E-06	down	413	415	359	508	459	667
ZGRF1	1.497839739	3.57E-08	up	264	328	263	68	53	95
ZHX2	-1.932229441	7.62702E-19	down	208	272	201	587	503	758
ZHX3	-1.206591173	0.007761855	down	903	1072	938	1052	1020	2759
ZIC2	7.74181891	1.60545E-12	up	197	197	209	0	2	0
ZIC5	10.01364356	1.11523E-15	up	277	304	225	0	0	0
ZIK1	-8.296640745	1.21614E-10	down	0	0	1	95	77	68
ZKSCAN3	-1.340193584	0.000117086	down	63	93	51	105	115	149
ZKSCAN4	-0.641066075	0.008358473	down	129	149	133	155	117	186
ZKSCAN8	-0.815129176	0.003263829	down	764	835	803	874	757	1396
ZMAT1	-6.253821314	1.28707E-24	down	7	17	6	850	222	582
ZMAT3	-1.872174603	1.74777E-14	down	333	364	335	849	662	1197
ZMAT5	-0.902999604	0.000107267	down	147	164	165	266	169	202
ZMIZ1	-0.92829911	2.50979E-05	down	894	1009	1054	1126	1352	1499
ZMYM6	-0.781851933	6.45316E-05	down	368	443	361	536	359	546
ZMYND11	-3.244691069	4.89188E-38	down	374	376	307	2553	1605	3052
ZMYND12	-7.826928371	4.81827E-08	down	0	0	0	41	29	19
ZMYND15	-3.591221715	0.000826457	down	0	15	4	68	53	36
ZMYND19	2.675218072	6.12956E-17	up	1192	1313	1163	123	176	101
ZNF10	-1.726061037	2.71994E-05	down	162	189	158	636	211	376
ZNF101	1.379030047	4.52542E-11	up	455	407	372	117	110	110
ZNF107	1.390427429	9.35803E-11	up	657	821	700	186	157	249
ZNF114	3.373996229	3.8336E-11	up	101	100	123	5	11	6
ZNF117	-3.553692916	2.22119E-13	down	189	194	213	1033	2606	1155
ZNF121	1.889552771	4.0859E-10	up	395	365	374	61	89	65
ZNF124	1.66521416	0.000194342	up	203	223	150	26	57	43
ZNF131	2.010911194	5.5977E-55	up	1937	2145	1949	388	336	338
ZNF132	-8.286392828	2.99931E-10	down	0	0	1	95	85	57
ZNF133	-1.098956512	7.9053E-07	down	206	277	203	420	269	359

ZNF135	-10.38722274	1.50117E-16	down	0	0	0	222	142	165
ZNF136	-1.128078917	8.87251E-05	down	135	171	132	300	155	232
ZNF14	-0.885571349	0.006580081	down	123	152	133	256	129	156
ZNF140	-0.927437953	0.002732014	down	253	234	228	459	259	258
ZNF141	-1.76658292	5.91894E-13	down	91	83	84	188	188	249
ZNF143	0.741181784	0.000109203	up	646	684	709	356	236	281
ZNF146	0.896313141	1.46896E-09	up	3514	3694	3153	1568	1110	1294
ZNF154	-6.503588826	1.99912E-11	down	5	0	1	79	201	98
ZNF16	-0.976537073	3.33544E-06	down	113	118	107	179	144	150
ZNF160	-1.142680219	1.66254E-08	down	451	472	427	830	511	800
ZNF165	2.627769887	5.76948E-09	up	90	109	95	18	8	8
ZNF17	-1.271121528	0.000719127	down	83	83	101	199	88	181
ZNF175	-0.694500679	0.003694396	down	160	236	165	235	174	236
ZNF18	-1.608050369	5.18414E-08	down	109	92	68	240	162	184
ZNF181	-1.506499168	2.37853E-09	down	195	217	159	501	291	369
ZNF19	-1.775115032	0.000352456	down	30	25	14	51	41	77
ZNF195	0.620931234	0.009945093	up	492	576	458	307	177	226
ZNF200	1.371148287	5.57315E-13	up	492	508	487	153	134	121
ZNF208	-1.856521204	0.000489146	down	76	89	61	92	174	312
ZNF211	-2.04347788	2.67775E-21	down	94	116	88	289	242	343
ZNF214	-1.329376366	1.07651E-05	down	47	59	41	96	86	79
ZNF215	-6.814225764	0.013112874	down	0	0	0	0	19	24
ZNF217	1.212878184	0.000734204	up	1661	1749	1703	323	615	605
ZNF219	-1.235556068	4.69916E-06	down	237	263	276	348	481	456
ZNF22	-0.744283484	2.00722E-08	down	589	605	535	692	636	729
ZNF221	2.101248311	2.29908E-10	up	143	133	153	20	25	26
ZNF223	-1.496491532	5.07108E-08	down	88	77	89	166	134	214
ZNF224	-1.086757996	1.32486E-06	down	348	459	460	745	475	709
ZNF225	-0.849961487	0.001044825	down	118	111	104	137	115	177
ZNF229	-7.762886576	1.15331E-10	down	2	0	0	60	114	132
ZNF232	1.172718238	0.014692745	up	340	301	320	170	47	95
ZNF235	-0.829793866	0.029535874	down	89	82	66	97	66	140
ZNF236	-0.653368364	0.016971687	down	176	179	145	164	157	239
ZNF239	1.245475208	9.0908E-10	up	363	381	339	104	113	106
ZNF248	-1.005756933	0.006646215	down	340	334	335	637	251	587
ZNF25	-4.226450561	1.47128E-29	down	62	56	68	1048	438	1053
ZNF250	-0.767239807	0.001246945	down	120	140	129	151	129	192
ZNF256	-9.545438012	5.78435E-14	down	0	0	0	117	86	91
ZNF257	-2.592869346	0.017443318	down	3	4	3	4	24	13
ZNF264	-0.928371277	8.9966E-06	down	269	252	273	350	297	434
ZNF266	-1.465499766	3.37726E-06	down	427	387	337	1032	459	812
ZNF267	1.227541881	1.54086E-09	up	589	652	517	218	156	160
ZNF268	-1.107487468	2.26573E-09	down	205	198	228	338	281	352
ZNF273	-0.806050822	0.03423174	down	145	201	162	143	189	296
ZNF274	-1.275141466	5.02673E-13	down	205	204	190	402	298	334
ZNF275	-0.758177171	0.003118243	down	285	338	301	336	289	491
ZNF276	1.010107128	2.32074E-07	up	1072	1134	1118	418	291	475
ZNF28	1.104199982	1.31919E-07	up	426	510	472	142	164	156
ZNF280C	1.277099214	3.82984E-07	up	243	286	235	74	56	95
ZNF280D	-1.884925704	1.65991E-19	down	370	405	421	1189	758	1229
ZNF281	2.234969831	4.71044E-29	up	1487	1719	1595	208	255	256
ZNF282	0.832014634	3.33431E-08	up	755	834	750	301	278	355
ZNF284	0.654231602	0.013805554	up	175	198	135	84	68	77
ZNF285	-2.129538558	3.26429E-08	down	30	22	19	77	77	66
ZNF286A	0.707101348	0.000692358	up	389	372	305	171	128	167
ZNF287	-9.404987247	4.66239E-14	down	1	0	0	192	147	181
ZNF300	1.335379813	7.73192E-10	up	692	736	671	243	186	162
ZNF302	-0.851921824	2.4397E-08	down	813	878	746	1218	839	1086
ZNF304	-1.030626221	8.2915E-07	down	183	194	155	299	200	278
ZNF32	-6.44678837	9.28512E-44	down	14	5	17	1030	624	617
ZNF329	-10.53221985	2.82264E-17	down	0	0	0	194	164	225
ZNF333	-3.378742408	5.46329E-31	down	52	57	30	339	294	393
ZNF334	-9.364255506	5.78553E-12	down	0	0	0	60	118	74
ZNF337	-0.659515704	0.005286448	down	530	624	551	723	439	773
ZNF34	-2.012599048	1.6984E-10	down	60	40	47	145	113	168
ZNF341	1.080500037	0.000338621	up	110	157	134	55	36	44
ZNF343	-1.290045807	5.14221E-05	down	190	146	122	316	182	310
ZNF345	-3.701358172	5.31647E-18	down	27	23	11	208	127	235
ZNF347	-11.20900211	1.82644E-19	down	0	0	0	288	273	369
ZNF350	-2.016717512	3.20335E-12	down	88	94	88	327	168	293
ZNF354A	-0.610095103	0.000208958	down	263	280	291	314	267	326
ZNF354B	-0.946117357	0.003292013	down	213	194	178	234	196	379
ZNF354C	-10.21684531	3.09849E-16	down	1	0	0	342	220	357
ZNF358	-1.87323369	1.70643E-17	down	338	400	350	1220	691	949
ZNF362	-3.245577343	8.73922E-23	down	80	87	91	673	338	762
ZNF365	-3.360245974	0.000402969	down	21	18	19	283	13	150
ZNF366	-9.83608641	2.3115E-12	down	1	0	0	98	361	213
ZNF367	3.259331251	3.15906E-47	up	858	963	847	75	70	52
ZNF382	-3.404775749	2.2223E-05	down	1	17	7	59	62	62
ZNF383	-0.805310572	5.43564E-05	down	235	245	189	303	225	306
ZNF385B	-6.035931075	0.000120455	down	1	2	0	15	6	122
ZNF385D	-11.5711192	9.20898E-17	down	0	1	0	858	225	1323
ZNF391	-4.129661412	6.20055E-16	down	8	10	7	74	126	105
ZNF394	-1.070135889	1.52079E-06	down	209	263	263	449	283	373
ZNF395	-0.726241882	0.001964649	down	869	911	895	955	831	1380
ZNF396	-1.389139638	0.000171147	down	51	36	39	90	55	93
ZNF397	-1.472480413	4.16866E-09	down	377	343	302	665	497	874
ZNF398	0.880564812	0.000201158	up	536	628	581	295	164	221
ZNF404	-6.312349412	4.12561E-11	down	1	2	0	58	51	60
ZNF407	-1.038302522	9.016E-06	down	158	149	133	212	172	262
ZNF408	0.737026192	0.000634166	up	255	331	302	147	101	132
ZNF414	-1.000206038	0.003018718	down	78	55	74	122	86	89
ZNF415	-10.65045849	8.29416E-18	down	0	0	0	228	192	211
ZNF418	-8.574630719	5.54251E-11	down	0	1	0	71	108	109
ZNF419	-0.721993278	0.030256956	down	102	129	132	139	166	115
ZNF423	-9.487199127	1.47137E-17	down	1	1	0	254	331	429
ZNF425	-2.578684564	0.000175363	down	14	9	11	88	23	38
ZNF426	-0.742457047	0.001400952	down	318	318	284	468	272	365
ZNF428	-2.2873862	5.56658E-21	down	140	165	141	685	410	462
ZNF429	-0.749291499	0.000111275	down	165	190	158	224	165	226
ZNF43	-9.382088777	2.68249E-13	down	0	0	0	77	91	92

ZNF431	1.126094541	0.001927953	up	538	554	502	109	204	198
ZNF432	-0.667691693	0.00431329	down	160	200	195	258	168	204
ZNF433	-2.706626185	7.13241E-13	down	15	18	24	103	67	97
ZNF438	-2.046869653	1.40832E-12	down	128	117	98	372	225	424
ZNF439	-8.879841352	3.05106E-12	down	0	1	0	153	100	109
ZNF44	-1.790623448	4.37371E-17	down	159	165	123	405	354	336
ZNF441	-2.558995126	6.87259E-12	down	97	88	73	542	217	343
ZNF443	-1.098526233	0.007539037	down	40	60	60	105	49	93
ZNF444	-0.961938076	0.001787888	down	162	197	215	317	170	320
ZNF446	-1.451683827	5.15363E-09	down	53	65	62	126	97	128
ZNF449	-1.321908499	3.76475E-09	down	187	188	156	279	290	373
ZNF454	-8.228774685	1.09478E-09	down	0	0	0	32	40	45
ZNF460	-1.12819029	0.008998902	down	37	29	24	62	37	42
ZNF461	-2.48500257	1.87822E-12	down	43	34	33	181	95	169
ZNF462	-1.371366107	0.008463144	down	96	101	100	84	184	274
ZNF467	-2.975057555	3.74599E-11	down	20	23	13	142	97	73
ZNF468	1.085053031	1.6537E-05	up	595	560	564	237	188	151
ZNF469	1.581888213	0.023906238	up	228	267	264	24	110	37
ZNF470	-9.742697922	7.65674E-15	down	0	1	0	252	162	247
ZNF471	-11.82903368	4.18058E-21	down	0	0	0	501	328	616
ZNF473	1.072301438	0.001270088	up	449	463	401	222	100	128
ZNF483	-11.21569393	1.78967E-17	down	0	0	0	473	154	330
ZNF484	-0.66215829	0.003095572	down	124	146	117	148	120	168
ZNF486	-8.864175467	1.36952E-11	down	0	0	0	58	68	55
ZNF488	4.301818503	9.34362E-18	up	161	153	157	9	6	2
ZNF490	-1.165880271	0.003290927	down	56	36	39	66	56	89
ZNF491	-4.479189344	3.30655E-13	down	3	4	4	51	47	77
ZNF492	2.604064975	2.4657E-07	up	56	78	65	7	10	6
ZNF493	-2.189932158	3.91948E-17	down	87	135	80	316	290	367
ZNF506	-2.37649324	7.51551E-20	down	73	98	73	320	316	255
ZNF511	-0.742566238	0.012273406	down	220	310	228	397	204	308
ZNF512B	0.624759014	0.046315256	up	401	456	365	138	213	204
ZNF513	-2.024453508	4.25705E-15	down	83	100	76	293	177	285
ZNF516	-4.096190364	1.48453E-43	down	33	32	32	460	270	465
ZNF517	-1.81322877	2.15934E-09	down	53	60	35	136	101	133
ZNF518A	-1.884825175	6.70235E-23	down	238	307	246	805	530	752
ZNF518B	-11.8888732	4.99307E-22	down	0	0	0	532	489	462
ZNF521	-1.62155168	3.10927E-06	down	173	183	177	253	429	470
ZNF524	-1.592150411	2.43792E-07	down	81	106	93	270	174	157
ZNF525	0.999145091	0.000693341	up	318	377	299	104	88	163
ZNF526	0.933110219	8.50813E-05	up	260	315	301	125	108	92
ZNF528	-10.89137541	2.0514E-17	down	0	0	0	308	147	304
ZNF529	0.652042205	1.54939E-06	up	716	752	669	347	276	346
ZNF530	1.567301446	1.60082E-09	up	210	223	204	54	55	43
ZNF532	-2.085671169	4.14072E-11	down	625	798	689	2494	1204	2783
ZNF536	-8.681450502	1.15474E-10	down	0	0	0	78	39	46
ZNF540	-3.318600771	1.44007E-13	down	32	30	29	320	107	235
ZNF541	-3.740724057	0.026455506	down	0	2	2	5	28	2
ZNF547	-2.387894048	2.68301E-05	down	15	21	16	108	48	39
ZNF549	-10.14929016	4.48763E-16	down	0	0	0	158	141	146
ZNF554	-1.221190036	0.000197313	down	65	59	41	89	77	108
ZNF555	-0.79520691	0.001655776	down	97	105	77	112	108	123
ZNF556	8.520000078	4.29906E-11	up	84	118	85	0	0	0
ZNF558	-0.888638959	1.95329E-07	down	245	269	269	399	289	347
ZNF559	-2.699504873	1.88035E-29	down	69	77	77	404	251	385
ZNF561	-1.352778639	2.44638E-06	down	387	423	368	912	436	826
ZNF563	-1.844237264	1.41515E-05	down	30	37	36	133	62	71
ZNF564	-2.104400246	6.10541E-13	down	60	93	63	282	175	204
ZNF568	-10.77498616	3.4475E-18	down	0	0	0	260	203	226
ZNF569	-1.451695836	1.7137E-05	down	66	116	96	209	184	142
ZNF57	0.929187671	0.001849654	up	151	168	122	65	53	46
ZNF570	-5.561029998	3.78866E-34	down	9	7	5	318	189	203
ZNF571	-1.233637724	0.008573519	down	70	55	29	95	60	105
ZNF572	-3.793545214	7.5804E-07	down	10	1	4	44	45	61
ZNF575	-3.054703072	1.74461E-07	down	7	11	6	71	31	41
ZNF577	-11.40564122	7.17733E-20	down	0	0	0	372	259	444
ZNF578	-8.280724205	2.38983E-08	down	0	0	0	22	61	35
ZNF580	-1.846815988	3.17754E-09	down	166	165	162	542	250	493
ZNF582	-7.144840626	3.75742E-10	down	0	0	2	73	52	78
ZNF583	-8.590101044	1.40939E-11	down	1	0	0	94	86	115
ZNF584	0.595137143	0.008068136	up	199	239	244	113	89	120
ZNF585A	-0.917366037	0.000143411	down	188	203	191	217	262	298
ZNF585B	-0.925947999	2.05418E-05	down	264	219	218	369	255	331
ZNF587	0.84698037	0.000837293	up	752	757	778	246	270	389
ZNF589	-0.799658937	0.032841632	down	363	315	304	354	263	617
ZNF595	-3.348065018	5.69749E-22	down	22	24	25	219	115	187
ZNF596	-2.619649504	3.25057E-18	down	28	24	23	118	93	118
ZNF597	1.045551596	2.71546E-05	up	316	301	284	106	76	131
ZNF598	2.035862726	4.73114E-45	up	1557	1749	1736	336	249	292
ZNF599	-2.018548668	1.78378E-06	down	26	23	13	66	57	55
ZNF606	-10.03575418	2.56558E-15	down	0	0	0	127	150	131
ZNF607	0.655468491	0.041296419	up	308	271	271	177	84	129
ZNF608	-10.4233818	8.44473E-16	down	0	1	0	229	427	374
ZNF610	-8.747093273	1.75583E-11	down	0	0	0	60	56	52
ZNF613	-1.809903232	4.81141E-05	down	54	39	52	179	68	124
ZNF615	-1.803857833	5.28118E-12	down	115	148	92	351	222	311
ZNF618	-0.681208361	0.015074016	down	337	339	319	458	242	452
ZNF619	-0.89688238	0.021395443	down	63	76	68	62	98	111
ZNF620	-3.256147568	1.31105E-07	down	15	9	8	62	40	120
ZNF622	1.689486771	3.0529E-29	up	1986	2166	2006	497	444	412
ZNF624	-2.232294674	2.25621E-11	down	35	32	37	95	119	132
ZNF625	-7.14914647	2.40532E-06	down	0	0	0	12	21	22
ZNF626	-10.29714043	3.37635E-16	down	0	0	0	149	140	206
ZNF627	-1.227946855	6.01022E-10	down	113	139	118	221	196	197
ZNF628	0.854870226	0.033406445	up	106	100	90	27	46	42
ZNF641	-1.013289909	9.58012E-07	down	448	446	462	632	517	811
ZNF649	-10.45287112	6.15448E-17	down	0	0	0	221	158	173
ZNF653	-1.514021673	0.006538501	down	39	40	22	114	42	52
ZNF654	-0.750505868	6.61646E-05	down	342	379	375	485	335	501
ZNF658	-2.036020146	1.81619E-05	down	19	46	34	135	79	74
ZNF66	3.760191247	0.007936423	up	16	29	12	0	0	3

ZNF662	-10.65344078	7.98161E-18	down	0	0	0	224	194	214
ZNF665	-4.131074814	2.28001E-05	down	9	1	2	33	30	91
ZNF667	-9.917001212	2.62315E-15	down	0	0	0	136	119	124
ZNF668	0.961063848	8.55337E-05	up	282	289	265	102	112	89
ZNF669	1.65543662	5.71681E-11	up	242	240	239	55	59	48
ZNF671	-9.467369859	4.21593E-12	down	0	0	0	67	131	72
ZNF675	1.58174668	3.87198E-16	up	430	438	369	97	86	111
ZNF676	-4.825974918	0.008699674	down	0	1	0	4	4	14
ZNF677	-9.549022389	1.13935E-13	down	0	0	0	97	109	85
ZNF681	-8.442595189	9.80454E-10	down	0	0	0	33	37	67
ZNF682	-9.643202758	4.86553E-14	down	0	0	0	136	84	96
ZNF687	1.268351066	4.64314E-09	up	893	946	855	254	204	342
ZNF688	-2.159557252	2.59058E-08	down	49	40	71	217	160	135
ZNF69	-9.080937789	2.39374E-12	down	0	0	0	62	74	75
ZNF695	4.468918581	5.71793E-09	up	50	80	58	3	2	1
ZNF696	-0.730384556	0.001327735	down	116	147	111	170	126	145
ZNF699	-1.130031123	7.48051E-05	down	101	95	101	139	125	201
ZNF700	-0.769232414	0.023273058	down	221	244	213	408	190	234
ZNF704	-2.028693872	7.75384E-15	down	715	702	664	1801	1505	2785
ZNF708	-0.938475266	8.73079E-05	down	151	167	149	181	202	251
ZNF709	-1.721705609	0.000705868	down	26	25	22	79	28	68
ZNF71	-1.653271105	4.12007E-11	down	46	52	52	120	98	118
ZNF710	0.629090463	0.005976817	up	331	314	327	185	130	134
ZNF711	-4.485984894	1.01857E-18	down	16	23	11	410	134	266
ZNF713	1.340169089	0.004009778	up	84	115	93	20	38	22
ZNF714	2.614647965	2.07481E-32	up	577	608	590	65	54	88
ZNF716	5.058928811	0.005914793	up	7	10	9	0	0	0
ZNF718	-9.988176111	5.87299E-15	down	0	0	0	128	98	176
ZNF727	-4.802145456	2.35894E-16	down	8	7	4	75	130	168
ZNF728	-8.448682357	3.88132E-10	down	0	0	0	40	35	63
ZNF730	-5.742872348	0.000104863	down	0	0	1	14	12	15
ZNF737	-1.571727281	6.27333E-07	down	87	85	87	140	169	238
ZNF747	-0.902737435	3.42272E-05	down	125	148	152	216	151	201
ZNF749	0.942794218	0.018017117	up	118	198	176	44	69	66
ZNF750	-5.080236213	0.021353218	down	0	0	0	5	6	2
ZNF75A	-1.172320549	3.20483E-10	down	145	156	134	267	203	228
ZNF75D	-1.380272899	1.06243E-13	down	188	235	167	369	329	390
ZNF76	-1.666390302	5.69418E-19	down	172	207	161	463	322	438
ZNF763	-4.839048272	3.68536E-13	down	6	5	0	79	74	69
ZNF765	0.991642179	0.024449197	up	145	173	146	40	38	89
ZNF766	-0.641224804	0.023182253	down	359	315	273	431	238	396
ZNF770	0.897092774	7.96865E-13	up	2111	2241	2107	857	799	803
ZNF771	-3.315864634	6.71161E-15	down	21	12	15	140	76	130
ZNF772	-3.00073359	5.05751E-15	down	19	28	34	157	173	126
ZNF773	-5.967975305	2.71032E-13	down	2	3	0	58	68	95
ZNF774	1.909145519	1.93348E-10	up	158	222	209	42	37	32
ZNF776	-0.864199023	0.009721113	down	201	230	240	204	323	331
ZNF781	-9.809227947	1.42524E-14	down	0	0	0	138	83	135
ZNF782	-1.219695292	0.020768432	down	57	64	50	93	43	153
ZNF784	-2.163825232	2.69983E-12	down	40	36	31	115	89	139
ZNF788	-7.792800013	2.21934E-12	down	2	0	0	123	88	106
ZNF790	-2.906443295	1.00145E-19	down	23	35	22	129	137	157
ZNF791	-0.847167815	0.000959042	down	377	433	452	569	376	688
ZNF792	-1.790234448	0.006947605	down	34	37	35	29	102	125
ZNF793	-10.33221867	1.51382E-15	down	0	0	0	165	108	240
ZNF804A	7.377599397	2.48019E-11	up	148	181	139	1	0	1
ZNF805	-1.626879379	9.52694E-14	down	96	91	83	229	177	188
ZNF813	1.520390737	0.002040341	up	175	181	164	20	50	57
ZNF821	-0.931916979	0.000992647	down	61	81	70	111	72	106
ZNF823	-1.002362819	0.017477167	down	88	95	56	156	67	122
ZNF827	-3.566344122	1.5417E-34	down	48	49	46	445	270	505
ZNF829	-9.800248389	1.16705E-14	down	0	0	0	116	92	144
ZNF83	-2.734807811	5.84574E-11	down	287	290	248	2025	604	1381
ZNF835	-7.756245737	6.45389E-08	down	0	0	0	40	26	19
ZNF836	-1.200218644	3.19084E-06	down	98	86	89	159	114	177
ZNF837	-2.592301853	0.001027015	down	3	5	4	26	11	15
ZNF84	-0.866826246	3.13285E-05	down	499	544	461	832	529	599
ZNF844	-10.17485659	5.41129E-16	down	0	0	1	266	326	282
ZNF845	0.712578672	0.001497242	up	259	250	266	103	109	124
ZNF846	-3.341350286	9.68234E-20	down	50	70	73	523	266	626
ZNF850	-8.090188997	3.96549E-09	down	0	0	0	27	38	41
ZNF853	-9.775155732	9.95036E-15	down	0	1	0	238	158	281
ZNF860	3.912499859	7.39745E-51	up	713	725	634	27	27	44
ZNF862	-2.054348326	1.90575E-15	down	135	143	164	368	393	546
ZNF879	-2.653101524	8.24314E-16	down	26	22	27	130	84	125
ZNF880	-10.47803359	1.18004E-14	down	0	0	0	92	226	231
ZNF883	-2.755706356	0.000151221	down	12	11	23	105	88	27
ZNF891	0.819683431	0.00068445	up	183	227	187	82	63	96
ZNF90	-4.98129852	4.2634E-06	down	3	1	0	22	20	49
ZNF92	1.159567919	1.87527E-09	up	492	441	407	146	131	150
ZNF93	-5.454770822	1.00963E-06	down	1	0	3	66	42	18
ZNF98	-6.771815373	2.55636E-05	down	0	0	0	14	19	9
ZNF99	-5.663438489	0.00351525	down	0	0	0	4	5	11
ZNRF3	-0.935153398	0.019840638	down	251	229	235	473	168	356
ZP3	5.319780128	7.86422E-44	up	528	593	511	15	10	4
ZPR1	0.705988602	1.3119E-06	up	925	936	887	439	378	380
ZRANB1	-1.146860848	5.60484E-06	down	869	763	801	1025	1231	1570
ZSCAN12	0.660857864	0.001467224	up	557	666	484	266	203	300
ZSCAN18	-12.65239019	2.58857E-23	down	0	0	0	1159	490	928
ZSCAN23	-9.392577642	6.2549E-11	down	0	0	0	70	39	161
ZSCAN26	-1.604639195	1.60965E-12	down	186	205	196	487	299	497
ZSCAN30	-1.630074999	4.98569E-22	down	173	183	192	456	343	412
ZSCAN31	-1.951276108	7.80742E-08	down	64	60	32	174	117	139
ZSWIM1	1.153526344	0.002037618	up	311	265	280	70	119	80
ZSWIM3	1.330591975	4.12104E-07	up	161	199	196	48	48	61
ZSWIM4	1.954272748	1.65894E-22	up	465	456	505	93	83	86
ZSWIM5	0.982204531	0.003931188	up	209	276	268	128	76	68
ZSWIM6	0.586297974	0.001811712	up	761	664	681	366	271	368
ZSWIM7	-2.239318644	1.16579E-11	down	89	91	76	302	337	208
ZSWIM8	-0.994430721	0.000192163	down	707	745	757	910	808	1434
ZW10	1.539635647	1.48578E-21	up	1002	1076	920	228	232	271

ZWILCH	2.89655955	5.93455E-38	up	1326	1522	1255	165	122	104
ZWINT	5.052644344	8.13714E-30	up	2678	2903	2531	104	41	31
ZXDA	-1.442648409	1.3292E-05	down	76	82	68	204	99	139
ZXDB	1.861908106	1.38678E-17	up	988	1132	1018	257	142	221
ZYG11A	6.988523303	8.33404E-12	up	566	586	570	0	9	0
ZYG11B	-1.026676189	0.000482281	down	704	735	718	847	1272	951
