# Supplementary material for: Multi-omics analysis reveals NNMT as a master metabolic regulator of metastasis in esophageal squamous cell carcinoma
Source: NPJ Precis Oncol. 2024 Jan 30;8:24. doi: 10.1038/s41698-024-00509-w (PMC10828394; doi:10.1038/s41698-024-00509-w)
Supplement: Supplementary file 2 — Reporting Summary [file 41698_2024_509_MOESM2_ESM.pdf]

nature portfolio

Corresponding author(s): Mantang Qiu

Last updated by author(s): Nov 18, 2023

Reporting Summary

Nature Portfolio wishes to improve the reproducibility of the work that we publish. This form provides structure for consistency and transparency in reporting. For further information on Nature Portfolio policies, see our Editorial Policies and the Editorial Policy Checklist.

Statistics

For all statistical analyses, confirm that the following items are present in the figure legend, table legend, main text, or Methods section.

n/a	Confirmed	
☐	☒	The exact sample size (*n*) for each experimental group/condition, given as a discrete number and unit of measurement
☐	☒	A statement on whether measurements were taken from distinct samples or whether the same sample was measured repeatedly
☐	☒	The statistical test(s) used AND whether they are one- or two-sided<br>*Only common tests should be described solely by name; describe more complex techniques in the Methods section.*
☒	☐	A description of all covariates tested
☐	☒	A description of any assumptions or corrections, such as tests of normality and adjustment for multiple comparisons
☐	☒	A full description of the statistical parameters including central tendency (e.g. means) or other basic estimates (e.g. regression coefficient) AND variation (e.g. standard deviation) or associated estimates of uncertainty (e.g. confidence intervals)
☐	☒	For null hypothesis testing, the test statistic (e.g. *F*, *t*, *r*) with confidence intervals, effect sizes, degrees of freedom and *P* value noted<br>*Give P values as exact values whenever suitable.*
☒	☐	For Bayesian analysis, information on the choice of priors and Markov chain Monte Carlo settings
☒	☐	For hierarchical and complex designs, identification of the appropriate level for tests and full reporting of outcomes
☒	☐	Estimates of effect sizes (e.g. Cohen's *d*, Pearson's *r*), indicating how they were calculated

Our web collection on statistics for biologists contains articles on many of the points above.

Software and code

Policy information about availability of computer code

Data collection	Cell Ranger (Version 3.0.2) for 10x Genomics data
Data analysis	Custom scripts for analyzing data are available upon reasonable request. Softwares used in this study: R software (Version 4.0.5), Seurat package (Version 4.3.0).

For manuscripts utilizing custom algorithms or software that are central to the research but not yet described in published literature, software must be made available to editors and reviewers. We strongly encourage code deposition in a community repository (e.g. GitHub). See the Nature Portfolio guidelines for submitting code & software for further information.

Data

Policy information about availability of data

All manuscripts must include a data availability statement. This statement should provide the following information, where applicable:

- Accession codes, unique identifiers, or web links for publicly available datasets
- A description of any restrictions on data availability
- For clinical datasets or third party data, please ensure that the statement adheres to our policy

The raw sequence data reported in this paper have been deposited in the Genome Sequence Archive of the Beijing Institute of Genomics (BIG) Data Center, BIG, Chinese Academy of Sciences, under accession code HRA003417 and are publicly accessible at http://bigd.big.ac.cn/gsa-human. Custom scripts for analyzing data are available upon reasonable request.

Research involving human participants, their data, or biological material

Policy information about studies with human participants or human data. See also policy information about sex, gender (identity/presentation), and sexual orientation and race, ethnicity and racism.

Reporting on sex and gender	Sex or gender was not considered in the study design.
Reporting on race, ethnicity, or other socially relevant groupings	Race and ethnicity data were not collected.
Population characteristics	Population characteristics in this study were provided in detail in Supplementary tables.
Recruitment	ESCC patients who underwent surgery were recruited in this study. None of the patients had been treated with chemotherapy, radiation, or any other anti-tumor medicines prior to tumor resection.
Ethics oversight	This study was approved by the Ethics Committee Board of Peking University People's Hospital and informed consent was obtained from each patient.

Note that full information on the approval of the study protocol must also be provided in the manuscript.

Field-specific reporting

Please select the one below that is the best fit for your research. If you are not sure, read the appropriate sections before making your selection.

☒ Life sciences ☐ Behavioural & social sciences ☐ Ecological, evolutionary & environmental sciences

For a reference copy of the document with all sections, see nature.com/documents/nr-reporting-summary-flat.pdf

Life sciences study design

All studies must disclose on these points even when the disclosure is negative.

Sample size	Five ESCC patients were recruited for single cell RNA analysis and seventy-one ESCC patients were recruited for metabolomics analysis. The sample size was determined by the maximum available at the clinical time point. No prior calculation was performed.
Data exclusions	There is no data that were excluded from analyses.
Replication	Given the small number of available specimens, single cell transcriptome were not technical replicated on individual samples.
Randomization	Participants were not chosen randomly. Since our work focused on the different single cell landscape between ESCC patients with or without lymph node metastasis, thus the experiments were not randomized.
Blinding	All samples from ESCC patients were performed by laboratory personnel who were without prior knowledge of the participant groups. The integration of all patients samples were performed without bias about expected results.

Reporting for specific materials, systems and methods

We require information from authors about some types of materials, experimental systems and methods used in many studies. Here, indicate whether each material, system or method listed is relevant to your study. If you are not sure if a list item applies to your research, read the appropriate section before selecting a response.

Materials & experimental systems

n/a	Involved in the study	
☐	☒	Antibodies
☐	☒	Eukaryotic cell lines
☒	☐	Palaeontology and archaeology
☐	☒	Animals and other organisms
☒	☐	Clinical data
☒	☐	Dual use research of concern
☒	☐	Plants

Methods

n/a	Involved in the study	
☒	☐	ChIP-seq
☒	☐	Flow cytometry
☒	☐	MRI-based neuroimaging

Antibodies

Antibodies used	NNMT antibody (ab119758, mouse, abcam, Cambridge, UK); E-cadherin antibody (ab40772, rabbit, abcam, Cambridge, UK); N-cadherin antibody (ab76011, rabbit, abcam, Cambridge, UK); β-caterin antibody (ab32572, rabbit, abcam, Cambridge, UK); H3K4me3 antibody (ab8580, rabbit, abcam, Cambridge, UK); METTL14 antibody (ab309096, rabbit, abcam, Cambridge, UK); IGF2BP1 antibody (8482, rabbit, Cell Signaling Technology, Danvers, MA, USA); m6A antibody (202003, mouse, Synaptic Systems, Goettingen, SN, Germany); β-actin antibody (66009-1-Ig, mouse, Proteintech, Rosemont, PA, USA).
Validation	All the the species and application of primary antibody were valadated. The information of antibody have been provided.

Eukaryotic cell lines

Policy information about cell lines and Sex and Gender in Research

Cell line source(s)	The Eca-109 and KYSE-30 cell lines were purchased from the Cell library of Shanghai Institutes for Biological Sciences, Chinese Academy of Sciences (Shanghai, China).
Authentication	All cell lines were identified by short tandem repeat (STR) analysis.
Mycoplasma contamination	All the cell lines tested negative for mycoplasma.
Commonly misidentified lines (See ICLAC register)	not applicable

Animals and other research organisms

Policy information about studies involving animals; ARRIVE guidelines recommended for reporting animal research, and Sex and Gender in Research

Laboratory animals	Five-week-old athymic BALB/c mice.
Wild animals	not applicable
Reporting on sex	not applicable
Field-collected samples	not applicable
Ethics oversight	This study was conducted in accordance with the Guide for the Care and Use of Laboratory Animals of the National Institutes of Health. The study protocol was approved by the Committee on the Ethics of Animal Experiments of Nanjing Medical University.

Note that full information on the approval of the study protocol must also be provided in the manuscript.

Plants

Seed stocks	not applicable
Novel plant genotypes	not applicable
Authentication	not applicable
